# Supplementary material for: Unified metal-free intermolecular Heck-type sulfonylation, cyanation, amination, amidation of alkenes by thianthrenation
Source: Nat Commun. 2024 Jan 15;15:529. doi: 10.1038/s41467-024-44746-w (PMC10789743; doi:10.1038/s41467-024-44746-w)
Supplement: Supplementary file 2 — Supplementary Information [file 41467_2024_44746_MOESM2_ESM.pdf]

Supplementary Information

for

Unified Metal-Free Regioselective Heck-Type Sulfonylation, Cyanation, Amination, Amidation of Alkenes by Thianthrenation

Ming-Shang Liu,[1] Hai-Wu Du,[1] Huan Meng,[1] Ying Xie,[3] and Wei Shu[*,1,2,3]

[1]*Shenzhen Grubbs Institute and Department of Chemistry, Guangming Advanced Research Institute, Southern University of Science and Technology, Shenzhen 518055, Guangdong, P. R. China*
[2]*State Key Laboratory of Elemento-Organic Chemistry, Nankai University, Tianjin 300071, P. R. China*
[3]*College of Chemistry and Environmental Engineering, Sichuan University of Science and Engineering, 643000, Zigong, P. R. China*

*E-mail: shuw@sustech.edu.cn

Table of Contents

1. Supplementary Methods S2
2. Supplementary Tables S3
3. Supplementary Notes S6
3.1 Synthesis of Materials S6
3.2 General Procedure for the Alkenes S15
3.3 Gram Scale Synthesis S16
3.4 Characterization of Cross-Coupling Products S18
3.5 Mechanistic Study S52
3.6 Crystallographic Data S57
3.7 Copies of ^{1}H, ^{13}C and ^{19}F NMR Spectra S60
4. Supplementary References S171

1. Supplementary Methods

General remarks: Unless otherwise noted, all reactions of substrates preparation were conducted in flame-dried glassware under a nitrogen atmosphere using anhydrous solvent were re-distilled according to *Purification of Laboratory Chemicals* (Fifth Edition). Commercially available reagents were used without further purification. Thin layer chromatography (TLC) was performed using Jiangyou TLC silica gel plates HSG F_{254} and visualized using UV light, anisaldehyde or potassium permanganate. Flash column chromatography was performed over silica gel (300-400 mesh). ^{1}H and ^{13}C NMR spectra were recorded in $CDCl_3$, unless otherwise noted, on a Bruker AVANCE 600 MHz or a Bruker AVANCE 500 MHz or a Bruker AVANCE 400 MHz spectrometer. Chemical shifts in ^{1}H NMR spectra were reported in parts per million (ppm) on the δ scale from an internal standard of residual chloroform (7.26 ppm). Data for ^{1}H NMR were reported as follows: chemical shift, multiplicity (s = singlet, d = doublet, t = triplet, q = quartet, m = multiplet, br = broad), coupling constant in Herts (Hz) and integration. Data for ^{13}C NMR spectra were reported in terms of chemical shift in ppm from the central peak of $CDCl_3$ (77.16 ppm). HRMS experiments were performed on a Thermo Scientific Q Exactive.

Materials and methods: Unless otherwise noted, commercial reagents were purchased from Energy Chemical Limited, J&K, Adamas-beta®, TCI, Aladdin, MERYER, Macklin Reagent, Alfa Aesar, Bidepharm and used directly without further purification. DCE and CH_3CN were distilled over CaH_2 and stored under nitrogen atmosphere. Thianthrene was purchased from MERYER.

2. Supplementary Tables

Supplementary Table 1. Evaluation of solvent for sulfonylation

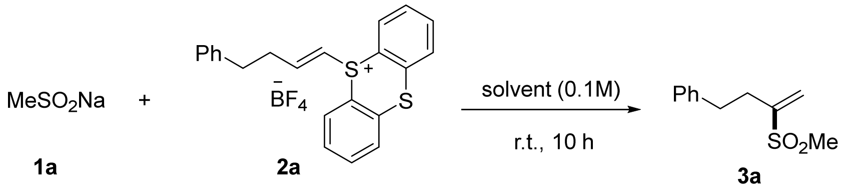

entry	solvent	conversion of **2a**	yield of **3a**[a]
1	DCE (0.1 M)	>95%	88% (83%)
2	DMF (0.1 M)	>95%	62%
3	DMA (0.1 M)	>95%	52%
4	THF (0.1 M)	>95%	75%
5	CH_3CN (0.1 M)	90%	56%
6	DCM (0.1 M)	>95%	78%
7	DMSO (0.1 M)	>95%	65%
8	DME (0.1 M)	>95%	77%
9	toluene (0.1 M)	>95%	76%

[a] The reaction was conducted using sodium methanesulfinate **1a** (15.6 mg, 0.15 mmol, 1.5 equiv), (*E*)-5-(4-phenylbut-1-en-1-yl)-5*H*-thianthren-5-ium tetrafluoroborate **2a** (43.4 mg, 0.10 mmol, 1.0 equiv) for 10 h. The reaction was run in 1.0 mL solvent at room temperature. Yield was determined by ^{1}H NMR of the crude mixture using mesitylene (12.0 mg, 0.10 mmol, 1.0 equiv) as internal standard. Isolated yield after flash chromatography is shown in the parentheses. N.D. = not detected.

Supplementary Table 2. Evaluation of additives for cyanation

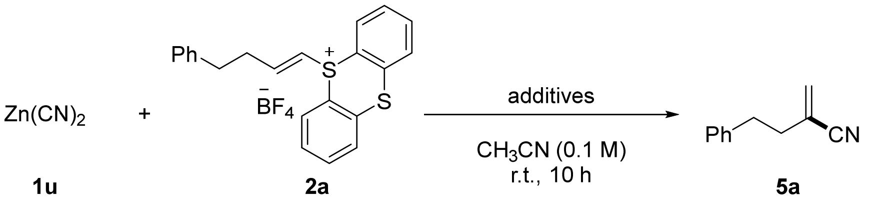

entry	additives	Conversion of **2a**	yield of **5a**[a]
1	-	20%	N.D.
2	^{n}Bu$_4$NF (1.0 equiv)	53%	N.D.

3	KI (1.0 equiv)	13%	N.D.
4	KBr (1.0 equiv)	48%	7%
5	KCl (1.0 equiv)	34%	10%
6	NaF (1.0 equiv)	13%	N.D.
7	KF (1.0 equiv)	50%	35%
8	CsF (1.0 equiv)	64%	27%
9	KF (2.0 equiv)	88%	50%
10	KF (3.0 equiv)	>95%	77% (70%)
11	KF (4.0 equiv)	>95%	73%

[a] The reaction was conducted using zinc cyanide **1u** (17.6 mg, 0.15 mmol, 1.5 equiv), (*E*)-5-(4-phenylbut-1-en-1-yl)-5*H*-thianthren-5-ium tetrafluoroborate **2a** (43.4 mg, 0.10 mmol, 1.0 equiv) and additives for 10 h. The reaction was run in 1.0 mL CH_3CN at room temperature. Yield was determined by ^1H NMR of the crude mixture using mesitylene (12.0 mg, 0.10 mmol, 1.0 equiv) as internal standard. Isolated yield after flash chromatography is shown in the parentheses. N.D. = not detected.

Supplementary Table 3. Evaluation of solvent for cyanation

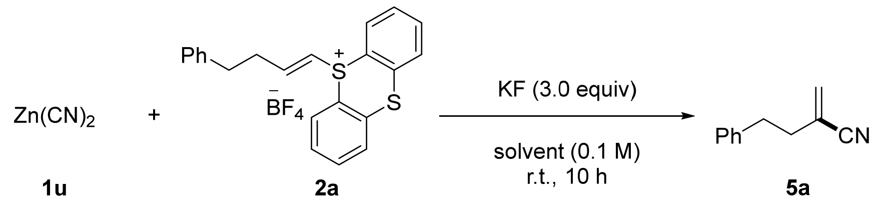

entry	solvent	Conversion of **2a**	yield of **5a**[a]
1	CH_3CN	>95%	77% (70%)
2	DCM	>95%	8%
3	DCE	>95%	7%
4	THF	>95%	trace
5	DMF	>95%	31%
6	DMSO	>95%	15%
7	toluene	75%	11%

[a] The reaction was conducted using zinc cyanide **1u** (17.6 mg, 0.15 mmol, 1.5 equiv), (*E*)-5-(4-phenylbut-1-en-1-yl)-5*H*-thianthren-5-ium tetrafluoroborate **2a** (43.4 mg, 0.10 mmol, 1.0 equiv) and KF (17.4 mg, 0.3 mmol, 3.0 equiv) for 10 h. The reaction was run in 1.0 mL solvent at room temperature. Yield was determined by ^1H NMR of the crude mixture using mesitylene (12.0 mg, 0.10 mmol, 1.0 equiv) as internal

standard. Isolated yield after flash chromatography is shown in the parentheses. N.D. = not detected.

Supplementary Table 4. Evaluation of base for amination

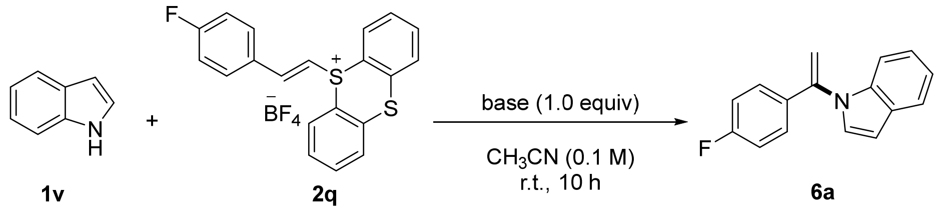

entry	base	Conversion of **2q**	yield of **6a**[a]
1	K_2CO_3 (1.0 equiv)	>95%	84% (82%)
2	Cs_2CO_3 (1.0 equiv)	>95%	69%
3	Na_2CO_3 (1.0 equiv)	25%	N.D.
4	K_3PO_4 (1.0 equiv)	>95%	59%
5	KOH (1.0 equiv)	>95%	62%

[a] The reaction was conducted using indole **1v** (17.6 mg, 0.15 mmol, 1.5 equiv), (*E*)-5-(4-fluorostyryl)-5*H*-thianthren-5-ium tetrafluoroborate **2q** (42.4 mg, 0.10 mmol, 1.0 equiv) and base (0.1 mmol, 1.0 equiv) for 10 h. The reaction was run in 1.0 mL CH_3CN at room temperature. Yield was determined by ^{1}H NMR of the crude mixture using mesitylene (12.0 mg, 0.10 mmol, 1.0 equiv) as internal standard. Isolated yield after flash chromatography is shown in the parentheses. N.D. = not detected.

Supplementary Table 5. Evaluation of solvent for amination

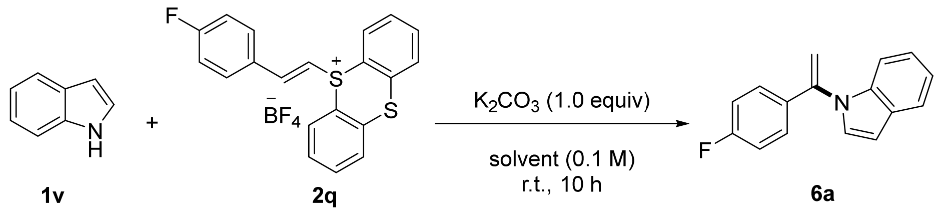

entry	solvent	Conversion of **2q**	yield of **6a**[a]
1	CH_3CN (0.1 M)	>95%	84% (82%)
2	DCM (0.1 M)	79%	32%
3	DCE (0.1 M)	86%	40%
4	THF (0.1 M)	>95%	61%
5	DMF (0.1 M)	>95%	36%
6	toluene (0.1 M)	58%	18%

[a] The reaction was conducted using indole **1v** (17.6 mg, 0.15 mmol, 1.5 equiv), (*E*)-5-(4-fluorostyryl)-5*H*-thianthren-5-ium tetrafluoroborate **2q** (42.4 mg, 0.10 mmol, 1.0 equiv) and K_2CO_3 (13.8 mg, 0.1 mmol, 1.0 equiv) for 10 h. The reaction was run in 1.0 mL solvent at room temperature. Yield was determined by ^{1}H NMR of the crude mixture using mesitylene (12.0 mg, 0.10 mmol, 1.0 equiv) as internal standard. Isolated yield after flash chromatography is shown in the parentheses. N.D. = not detected.

3. Supplementary Notes

3.1 Synthesis of Materials

$MeSO_2Na$

1a

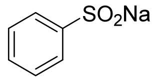

1b

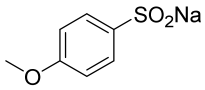

1c

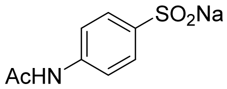

1d

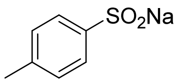

1e

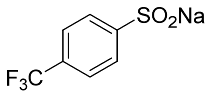

1f

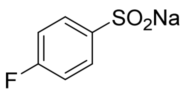

1g

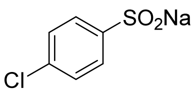

1h

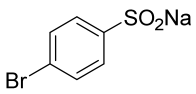

1i

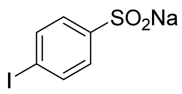

1j

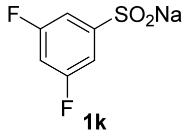

1k

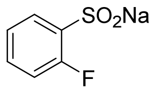

1l

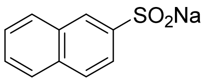

1m

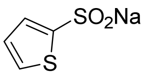

1n

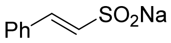

1o

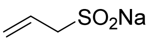

1p

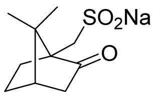

1q

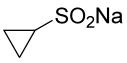

1r

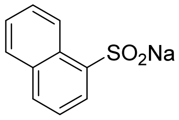

1s

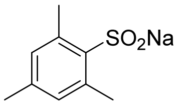

1t

$Zn(CN)_2$

1u

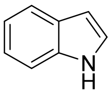

1v

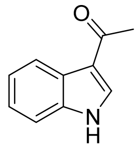

1w

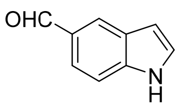

1x

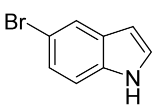

1y

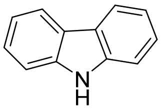

1z

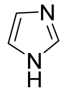

1aa

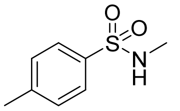

1ab

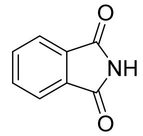

1ac

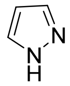

1ad

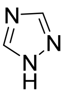

1ae

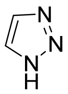

1af

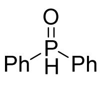

1ag

1a, **1b**, **1e**, **1g**, **1h**, **1u**, **1v**, **1w**, **1x**, **1y**, **1z**, **1aa**, **1ab**, **1ac**, **1ad**, **1ae**, **1af**, **1ag** were purchased from Alfa Aesar, Energy Chemical, Bidepharm, Adamas, Aladdin and TCI.

1c, **1d**, **1f**, **1i**, **1j**, **1k**, **1l**, **1m**, **1n**, **1o**, **1p**, **1q**, **1r**, **1s** and **1t** were synthesized following reported method.[1-3]

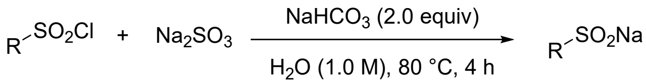

Sodium sulfite (1.25 g, 10.0 mmol, 2.0 equiv), sodium bicarbonate (0.84 g, 10.0 mmol, 2.0 equiv) and the corresponding aryl sulfonyl chloride (5.0 mmol, 1.0 equiv) were dissolved in distilled water (5.0 mL). The reaction mixture was stirred for 4 h at 80 °C. After cooling to rt, water was removed by lyophilization overnight. The white residue was extracted with ethanol (20.0 mL) to obtain the desired aryl sulfinate as white crystalline powder.

Sodium 4-acetamidobenzenesulfinate (1d, CAS: 15898-43-8)

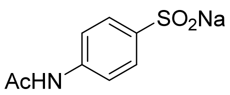

4-Acetamidobenzenesulfonyl chloride was used for the reaction to afford **1d** as white solid (0.33 g, 75% yield, 2.0 mmol scale). **^{1}H NMR** (400 MHz, D_2O) δ 7.63 – 7.57 (m, 2H), 7.53 – 7.47 (m, 2H), 2.13 (s, 3H). The characterization data are consistent with literature.[2]

Sodium 4-bromobenzenesulfinate (1i, CAS: 34176-08-4)

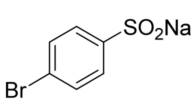

4-Bromobenzenesulfonyl chloride was used for the reaction to afford **1i** as white solid (0.40 g, 82% yield, 2.0 mmol scale). **^{1}H NMR** (400 MHz, D_2O) δ 7.71 – 7.65 (m, 1H), 7.55 – 7.48 (m, 1H). The characterization data are consistent with literature.[1]

Sodium 4-iodobenzenesulfinate (1j, CAS: 61404-98-6)

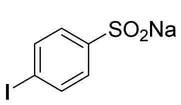

4-Iodobenzenesulfonyl chloride was used for the reaction to afford **1l** as white solid (0.36 g, 62% yield, 2.0 mmol scale). **^{1}H NMR** (400 MHz, D_2O) δ 7.90 (d, J = 8.3 Hz, 2H), 7.38 (d, J = 8.3 Hz, 2H). The characterization data are consistent with literature.[2]

Sodium naphthalene-2-sulfinate (1m, CAS: 63735-42-2)

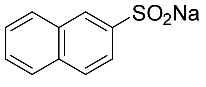

Naphthalene-2-sulfonyl chloride was used for the reaction to afford **1m** as white solid (0.34 g, 79% yield, 2.0 mmol scale). **^{1}H NMR** (600 MHz, D_2O) δ 8.05 (s, 1H), 7.97 (s, 2H), 7.91 (s, 2H), 7.69 (s, 1H), 7.55 (s, 2H). The characterization data are consistent with literature.[1-3]

Sodium (*E*)-2-phenylethene-1-sulfinate (1r, CAS: 130665-83-7)

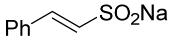

(*E*)-2-phenylethene-1-sulfonyl chloride was used for the reaction to afford **1r** as white solid (0.69 g, 73% yield, 5.0 mmol scale). **^{1}H NMR** (400 MHz, D2O) δ 7.61 – 7.52 (m, 2H), 7.48 – 7.37 (m, 3H), 7.01 (d, *J* = 16.1 Hz, 1H), 6.86 (d, *J* = 16.1 Hz, 1H). The characterization data are consistent with literature.[3]

Sodium 2,4,6-trimethylbenzenesulfinate (1t, CAS: 50827-54-8)

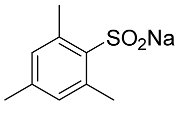

2,4,6-Trimethylbenzenesulfonyl chloride was used for the reaction to afford **1r** as white solid (0.34 g, 82% yield, 2.0 mmol scale). **^{1}H NMR** (400 MHz, D2O) δ 7.01 (s, 1H), 6.88 (s, 2H), 2.54 (s, 6H), 2.23 (s, 3H). The characterization data are consistent with literature.[3]

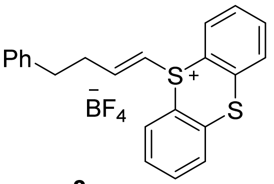

2a

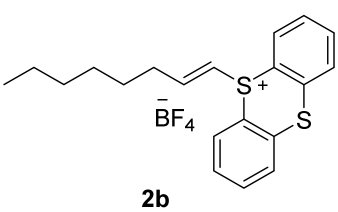

2b

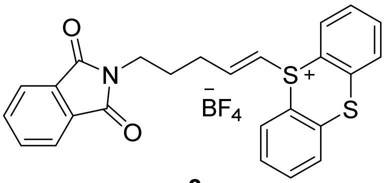

2c

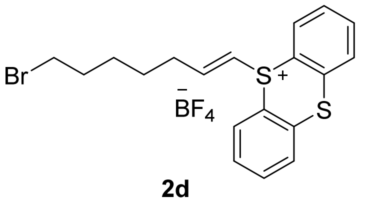

2d

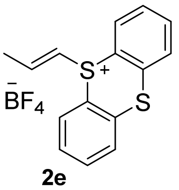

2e

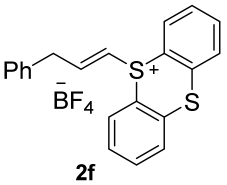

2f

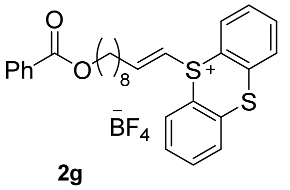

2g

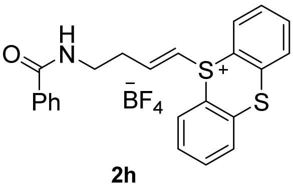

2h

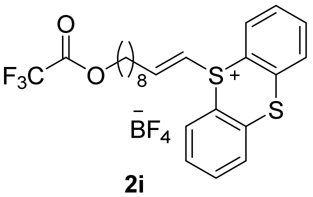

2i

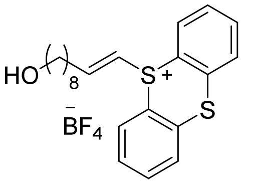

2j

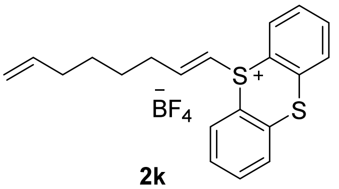

2k

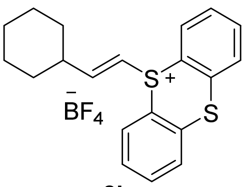

2l

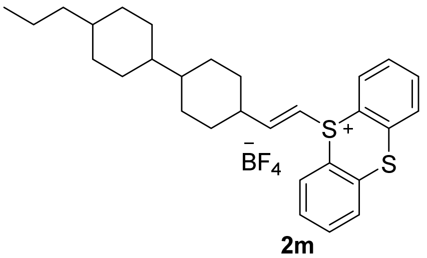

2m

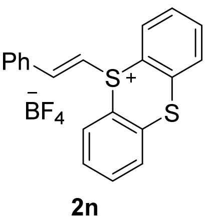

2n

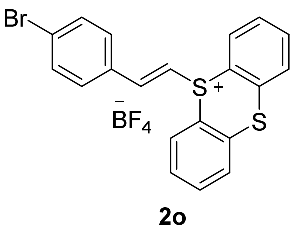

2o

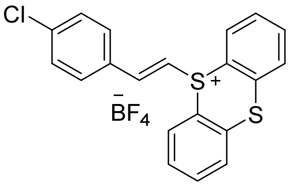

2p

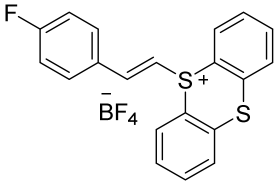

2q

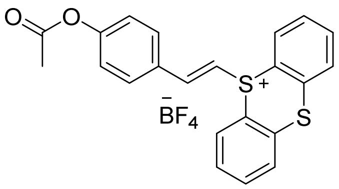

2r

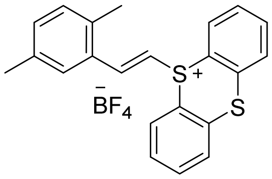

2s

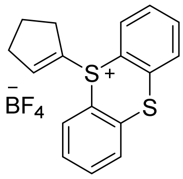

2t

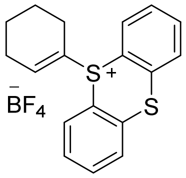

2u

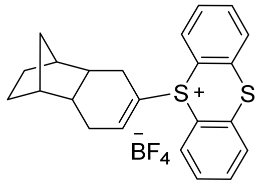

2v

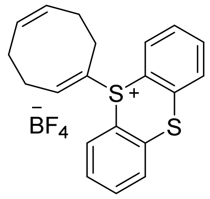

2w

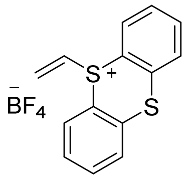

2x

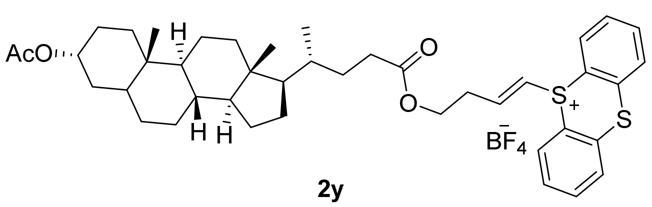

2y

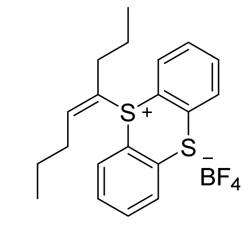

2z

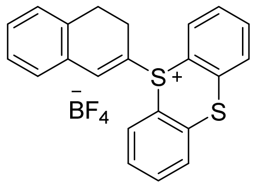

2aa

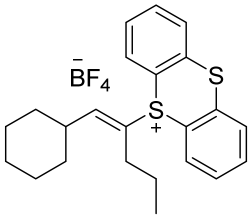

2bb

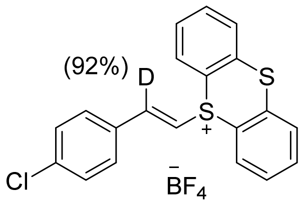

2cc

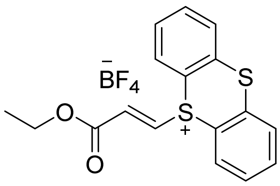

2dd

Alkenyl thianthren-5-ium tetrafluoroborate were synthesized following reported procedure.[4-8]

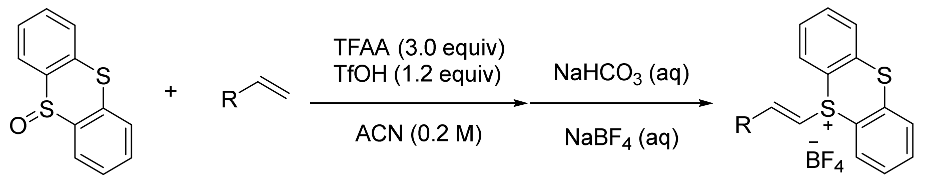

Under nitrogen atmosphere, a 50 mL Schlenk vial equipped with a magnetic stir bar was charged with alkene (2.0 mmol, 1.0 equiv), thianthrene 5-oxide (464 mg, 2.0 mmol, 1.0 equiv), and MeCN (10 mL, c = 0.20 M). After cooling to 0°C, trifluoroacetic anhydride (1260.0 mg, 6.0 mmol, 3.0 equiv) was added dropwise within 2 minutes, followed by dropwise addition of TfOH (360.0 mg, 2.4 mmol, 1.2 equiv.) within 2 minutes. After stirring the lilac mixture at 0 °C for 1h followed by stirring for 1h at room temperature, the resulting mixture was concentrated under reduced pressure, and subsequently diluted with CH_2Cl_2 (30 mL). The saturated aqueous $NaHCO_3$ solution (stir 10 minute, 3x30 mL) pour into CH_2Cl_2 solution, and the layers were separated with separatory funnel. Then, aqueous $NaBF_4$ solution (5x30 mL, 5 % w/w, stir 10 minute) pour into CH_2Cl_2 solution, and the layers were separated with separatory funnel. The CH_2Cl_2 layer was dried over Na_2SO_4, filtered, and the solvent was removed under reduced pressure. The residue was purified by chromatography on silica gel eluting with MeOH/DCM = 0-10% to afford the alkenyl thianthrenium salt.

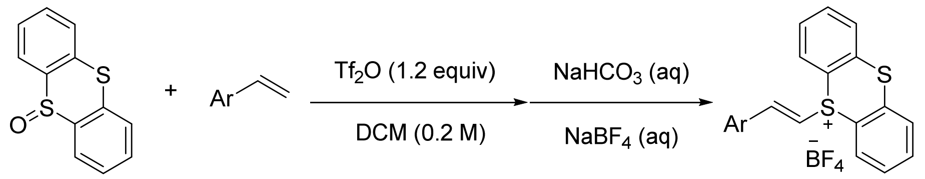

Under nitrogen atmosphere, a 50 mL Schlenk vial equipped with a magnetic stir bar was charged with styrene (2.0 mmol, 1.00 equiv), thianthrene 5-oxide (464.0 mg, 2.0 mmol, 1.0 equiv), and DCM (10 mL, c = 0.20 M). After cooling to -40°C, triflic anhydride (677.1 mg, 2.4 mmol, 1.2 equiv) was added dropwise to the reaction. After stirring the lilac mixture at -40 °C for 30 min followed by stirring for 1h at 0 °C. The resulting mixture was stirred at room temperature 2 h, then it was poured onto a saturated aqueous $NaHCO_3$ solution (stir 10 minute, 3x30 mL) and the layers were separated with separatory funnel. Then, aqueous $NaBF_4$ solution (5x30 mL, 5 % w/w, stir 10 minute) pour into CH_2Cl_2 solution, and the layers were separated with separatory funnel. The CH_2Cl_2 layer was dried over Na_2SO_4, filtered, and the solvent was removed under reduced pressure. The residue was purified by chromatography on

silica gel eluting with MeOH/DCM = 0-10% to afford the alkenyl thianthrenium salt.

(*E*)-5-(5-(1,3-Dioxoisoindolin-2-yl)pent-1-en-1-yl)-5*H*-thianthren-5-ium tetra-fluoroborate (2c, CAS: 2411696-52-9)

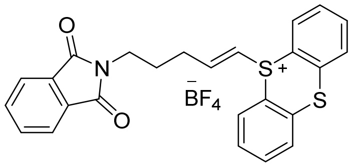

2-(Pent-4-en-1-yl)isoindoline-1,3-dione was used for the reaction to afford **2c** as white solid (1.47 g, 57% yield, 5.0 mmol scale). **^{1}H NMR** (400 MHz, $CDCl_3$) δ 8.39 (dd, *J* = 7.8, 1.2 Hz, 2H), 7.81 (ddd, *J* = 11.6, 6.6, 2.2 Hz, 4H), 7.75 – 7.64 (m, 6H), 7.33 – 7.21 (m, 1H), 6.64 (d, *J* = 14.8 Hz, 1H), 3.60 (t, *J* = 6.8 Hz, 2H), 2.31 (q, *J* = 6.9 Hz, 2H), 1.80 (p, *J* = 7.0 Hz, 2H). The characterization data are consistent with literature.[4]

(*E*)-5-(4-Benzamidobut-1-en-1-yl)-5*H*-thianthren-5-ium tetrafluoroborate (2h, CAS: 2411696-66-5)

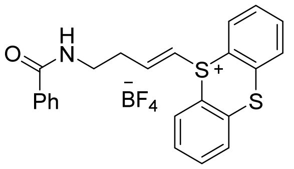

N-(But-3-en-1-yl)benzamide was used for the reaction to afford **2h** as white solid (0.54 g, 57% yield, 2.0 mmol scale). **^{1}H NMR** (400 MHz, $CDCl_3$) δ 8.20 (dd, *J* = 7.9, 1.1 Hz, 2H), 7.77 – 7.70 (m, 4H), 7.65 (td, *J* = 7.7, 1.2 Hz, 2H), 7.57 (td, *J* = 7.7, 1.3 Hz, 2H), , 7.47 – 7.39 (m, 2H), 7.33 (t, *J* = 7.5 Hz, 2H), 6.66 (d, *J* = 14.7 Hz, 1H), 3.62 (dt, *J* = 6.0, 5.7 Hz, 2H), 2.66 (dt, *J* = 6.0, 5.7 Hz, 2H). The characterization data are consistent with literature.[4]

(*E*)-5-(4-Bromostyryl)-5*H*-thianthren-5-ium tetrafluoroborate (2o, CAS: 2829324-90-3)

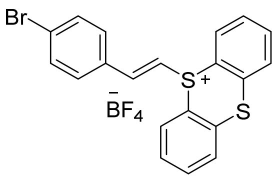

4-Bromostyrene was used for the reaction to afford **2o** as lightly yellow solid (0.96 g, 66% yield, 3.0 mmol scale). **^{1}H NMR** (400 MHz, $CDCl_3$) δ 8.40 (d, *J* = 7.7 Hz, 2H), 8.05 (d, *J* = 15.0 Hz, 1H), 7.85 (d, *J* = 7.8 Hz, 2H), 7.74 (t, *J* = 7.6 Hz, 2H), 7.67 (t, *J* = 7.6 Hz, 2H), 7.45 (d, *J* = 7.2 Hz, 2H), 7.37 (d, *J* = 8.5 Hz, 2H), 7.12 (d, *J* = 15.0 Hz, 1H). The characterization data are consistent with literature.[8]

(*E*)-5-(4-Chlorostyryl)-5*H*-thianthren-5-ium tetrafluoroborate (2p, CAS: 2829324-86-7)

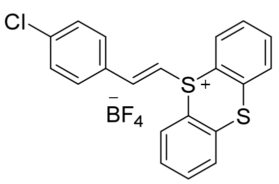

4-Chlorostyrene was used for the reaction to afford **2p** as lightly yellow solid (0.51 g, 58% yield, 2.0 mmol scale). **^{1}H NMR** (400 MHz, $CDCl_3$) δ 8.41 (d, *J* = 7.8 Hz, 2H), 8.08 (d, *J* = 15.1 Hz, 1H), 7.85 (dd, *J* = 7.8, 1.2 Hz, 2H), 7.74 (t, *J* = 7.2

Hz, 2H), 7.67 (t, J = 7.6 Hz, 2H), 7.45 (d, J = 8.5 Hz, 2H), 7.29 (d, J = 8.4 Hz, 2H), 7.10 (d, J = 15.1 Hz, 1H). The characterization data are consistent with literature.[8]

(*E*)-5-(4-Fluorostyryl)-5*H*-thianthren-5-ium tetrafluoroborate (2q, CAS: 2829324-84-5)

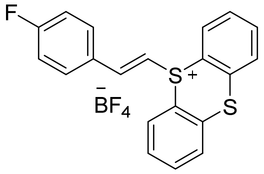

4-Fluorostyrene was used for the reaction to afford **2q** as lightly yellow solid (1.55 g, 73% yield, 5.0 mmol scale). **^{1}H NMR** (400 MHz, $CDCl_3$) δ 8.47 – 8.39 (m, 2H), 8.16 (d, J = 15.1 Hz, 1H), 7.85 (dd, J = 7.8, 1.3 Hz, 2H), 7.74 (td, J = 7.8, 1.3 Hz, 2H), 7.68 (td, J = 7.8, 1.3 Hz, 2H), 7.58 – 7.51 (m, 2H), 7.10 – 6.99 (m, 3H). The characterization data are consistent with literature.[8]

(*E*)-5-(4-Acetoxystyryl)-5*H*-thianthren-5-ium tetrafluoroborate (2r)

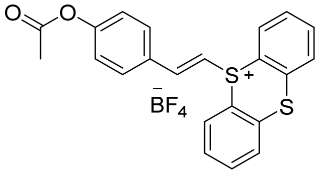

4-Acetoxystyrene was used for the reaction to afford **2r** as lightly yellow solid (0.81 g, 58% yield, 3.0 mmol scale). **^{1}H NMR** (400 MHz, $CDCl_3$) δ 8.42 (d, J = 7.0 Hz, 2H), 8.11 (d, J = 15.1 Hz, 1H), 7.84 (dd, J = 7.8, 1.3 Hz, 2H), 7.73 (td, J = 7.7, 1.2 Hz, 2H), 7.67 (td, J = 7.7, 1.2 Hz, 2H), 7.52 (d, J = 7.8 Hz, 2H), 7.12 – 7.02 (m, 3H), 2.27 (s, 3H);

^{13}C NMR (101 MHz, $CDCl_3$) δ 168.9, 153.7, 150.8, 135.8, 134.5, 134.0, 130.6, 130.5, 130.2, 129.8, 122.6, 120.9, 106.2, 21.2;

^{19}F NMR (376 MHz, $CDCl_3$) δ -150.05, -150.10;

HRMS-ESI (m/z) $[M\text{-}BF_4]^+$ calc'd for $C_{22}H_{17}O_2S_2^+$, 377.0664, found 377.0663.

(*E*)-5-(2,5-Dimethylstyryl)-5*H*-thianthren-5-ium tetrafluoroborate (2s)

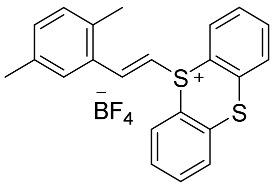

2,5-Dimethylstyrene was used for the reaction to afford **2s** as lightly yellow solid (0.60 g, 46% yield, 3.0 mmol scale). **^{1}H NMR** (400 MHz, $CDCl_3$) δ 8.48 (d, J = 7.8 Hz, 2H), 8.33 (dd, J = 15.0, 3.0 Hz, 1H), 7.84 (d, J = 7.1 Hz, 2H), 7.73 (t, J = 7.1 Hz, 2H), 7.68 (t, J = 7.6 Hz, 2H), 7.16 (s, 1H), 7.13 – 7.00 (m, 3H), 2.40 (s, 3H), 2.24 (s, 3H);

^{13}C NMR (101 MHz, $CDCl_3$) δ 149.7, 136.7, 136.1, 135.7, 134.4, 134.2, 133.2, 131.4, 130.9, 130.5, 130.1, 128.9, 127.8, 127.2, 121.3, 106.3, 20.9, 19.3;

^{19}F NMR (376 MHz, $CDCl_3$) δ -150.23, -150.29;

HRMS-ESI (m/z) $[M\text{-}BF_4]^+$ calc'd for $C_{22}H_{19}S_2^+$, 347.0923, found 347.0920.

5-((*E*)-4-(((4*R*)-4-((3*R*,8*R*,9*S*,10*S*,13*R*,14*S*,17*R*)-3-Acetoxy-10,13-dimethylhexa-decahydro-1*H*-cyclopenta[*a*]phenanthren-17-yl)pentanoyl)oxy)but-1-en-1-yl)-5*H*-thianthren-5-ium tetrafluoroborate (2y, CAS: 2700216-11-9)

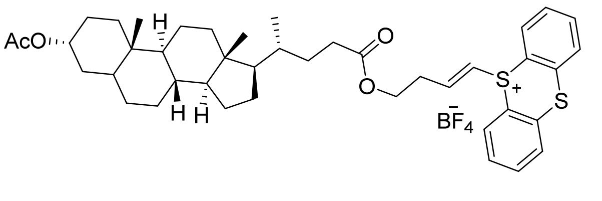

But-3-en-1-yl (4*R*)-4-((3*R*,8*R*,9*S*,10*S*,13*R*,14*S*,17*R*)-3-acetoxy-10,13-di-methylhexadecahydro-1*H*-cyclo-penta[*a*]phenanthren-17-yl)penta-noate was used for the reaction to afford **2y** as white solid (0.68 g, 44% yield, 2.0 mmol scale). **^{1}H NMR** (400 MHz, $CDCl_3$) δ 8.41 (d, *J* = 7.6 Hz, 2H), 7.83 (dd, *J* = 7.7, 1.4 Hz, 2H), 7.76 – 7.65 (m, 4H), 7.24 – 7.17 (m, 1H), 6.65 (d, *J* = 14.8 Hz, 1H), 4.72 (dt, *J* = 11.2, 6.5 Hz, 2H), 4.12 (t, *J* = 6.3 Hz, 2H), 2.60 (q, *J* = 6.0 Hz, 2H), 2.25 – 2.18 (m, 1H), 2.14 – 2.06 (m, 2H), 2.03 (s, 3H), 1.98 – 1.91 (m, 2H), 1.87 – 1.75 (m, 4H), 1.71 – 1.63 (m, 2H), 1.55 – 1.50 (m, 1H), 1.47 – 1.33 (m, 6H), 1.27 – 1.15 (m, 4H), 1.10 – 0.99 (m, 5H), 0.93 (s, 3H), 0.85 (d, *J* = 6.5 Hz, 3H), 0.63 (s, 3H). The characterization data are consistent with literature.[4]

(*Z*)-5-(Oct-4-en-4-yl)-5*H*-thianthren-5-ium tetrafluoroborate (2z, CAS: 2813345-90-1)

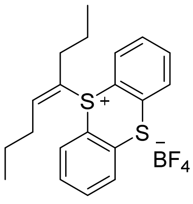

(*E*)-Oct-4-ene was used for the reaction to afford **2z** as white solid (0.63 g, 76% yield, 2.0 mmol scale). **^{1}H NMR** (400 MHz, $CDCl_3$) δ 8.24 (d, *J* = 7.2 Hz, 2H), 7.77 – 7.63 (m, 6H), 6.34 (t, *J* = 7.6 Hz, 1H), 2.68 (q, *J* = 7.4 Hz, 2H), 2.14 (t, *J* = 7.4 Hz, 2H), 1.49 (h, *J* = 7.4 Hz, 2H), 1.35 (h, *J* = 7.4 Hz, 2H), 0.99 (t, *J* = 7.4 Hz, 3H), 0.75 (t, *J* = 7.4 Hz, 3H). The characterization data are consistent with literature.[4]

5-(3,4-Dihydronaphthalen-2-yl)-5*H*-thianthren-5-ium tetrafluoroborate (2aa)

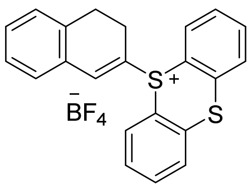

1,2-Dihydronaphthalene was used for the reaction to afford **2aa** as white solid (0.90 g, 69% yield, 3.0 mmol scale). **^{1}H NMR** (400 MHz, $CDCl_3$) δ 8.49 (d, *J* = 7.7 Hz, 2H), 7.80 (q, *J* = 7.8, 7.1 Hz, 4H), 7.75 (t, *J* = 7.3 Hz, 2H), 7.23 (d, *J* = 7.5 Hz, 1H), 7.15 (t, *J* = 7.5 Hz, 1H), 7.07 (d, *J* = 7.4 Hz, 1H), 7.02 (d, *J* = 7.5 Hz, 1H), 6.58 (s, 1H), 2.91 (t, *J* = 8.1 Hz, 2H), 2.35 (t, *J* = 8.1 Hz, 2H);

^{13}C NMR (101 MHz, $CDCl_3$) δ 137.7, 136.4, 135.7, 134.9, 134.8, 131.2, 130.7, 130.4,

129.9, 128.9, 128.0, 127.4, 121.5, 117.3, 27.8, 24.1;

^{19}F NMR (376 MHz, $CDCl_3$) δ -150.92, -150.97;

HRMS-ESI (m/z) $[M-BF_4]^+$ calc'd for $C_{22}H_{17}S_2^+$, 345.0766, found 345.0765.

(*E*)-5-(1-Cyclohexylpent-1-en-2-yl)-5*H*-thianthren-5-ium tetrafluoroborate (2bb)

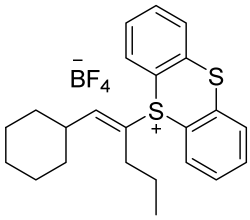

(*Z*)-Pent-1-en-1-ylcyclohexane was used for the reaction to afford **2bb** as white solid (0.71 g, 78% yield, 2.0 mmol scale, rr > 20:1).

^{1}H NMR (500 MHz, $CDCl_3$) δ 8.45 – 8.37 (m, 2H), 7.84 – 7.75 (m, 4H), 7.73 – 7.68 (m, 2H), 5.22 (d, *J* = 10.2 Hz, 1H), 2.32 – 2.21 (m, 1H), 2.21 – 2.08 (m, 2H), 1.68 – 1.55 (m, 3H), 1.53 – 1.45 (m, 2H), 1.31 – 1.02 (m, 5H), 0.98 – 0.88 (m, 2H), 0.81 (t, *J* = 7.3 Hz, 3H);

^{13}C NMR (126 MHz, $CDCl_3$) δ 146.3, 136.75, 136.73,135.9, 135.8, 134.83, 134.76, 130.3, 130.04, 129.98, 121.7, 117.4, 117.3, 39.4, 31.9, 30.3, 25.4, 25.1, 21.9, 13.7;

^{19}F NMR (471 MHz, $CDCl_3$) δ -151.57, -151.62;

HRMS-ESI (m/z) $[M-BF_4]^+$ calc'd for $C_{23}H_{27}S_2^+$, 367.1549, found 367.1551.

(*E*)-5-(2-(4-Chlorophenyl)vinyl-2-*d*)-5*H*-thianthren-5-ium tetrafluoroborate (2cc)

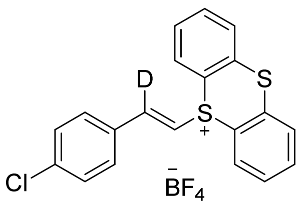

1-Chloro-4-(vinyl-1-d)benzene was used for the reaction to afford **2cc** as white solid (0.82 g, 46% yield, 4.0 mmol scale, D: 95%).

^{1}H NMR (500 MHz, $CDCl_3$) δ 8.48 (d, *J* = 7.9 Hz, 3H), 7.84 (dd, *J* = 7.9, 1.2 Hz, 2H), 7.74 (td, *J* = 7.7, 1.2 Hz, 3H), 7.67 (t, *J* = 7.7 Hz, 3H), 7.45 (d, *J* = 8.5 Hz, 2H), 7.28 (d, *J* = 8.5 Hz, 2H), 7.13 (s, 1H);

^{13}C NMR (126 MHz, $CDCl_3$) δ 150.0, 138.5, 135.8, 134.5, 134.3, 130.7, 130.5, 130.4, 130.2, 129.5, 120.9, 107.0;

^{19}F NMR (471 MHz, $CDCl_3$) δ -149.91, -149.96;

HRMS-ESI (m/z) $[M-BF_4]^+$ calc'd for $C_{20}H_{13}DClS_2^+$, 354.0283, found 354.0284.

(*E*)-5-(3-Ethoxy-3-oxoprop-1-en-1-yl)-5*H*-thianthren-5-ium tetrafluoroborate (2dd)

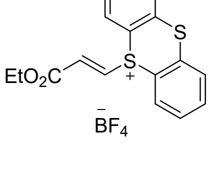

(*E*)-Ethyl 3-(4,4,5,5-tetramethyl-1,3,2-dioxaborolan-2-yl) acrylate was used for the reaction to afford **2dd** as white solid (0.69 g, 34%

yield, 5.0 mmol scale).

^{1}H NMR (500 MHz, $CDCl_3$) δ 8.52 (d, *J* = 7.1 Hz, 2H), 7.87 (d, *J* = 7.8 Hz, 2H), 7.80 (dd, *J* = 7.8, 7.4 Hz, 2H), 7.74 (dd, *J* = 7.4, 7.1 Hz, 2H), 7.38 (d, *J* = 14.7 Hz, 1H), 6.65 (d, *J* = 14.7 Hz, 1H), 4.19 (q, *J* = 7.1 Hz, 2H), 1.26 (t, *J* = 7.1 Hz, 3H);

^{13}C NMR (126 MHz, $CDCl_3$) δ 161.8, 137.0, 136.4, 135.5, 135.3, 130.8, 130.4, 127.0, 118.1, 62.8, 14.0;

^{19}F NMR (471 MHz, $CDCl_3$) δ -149.84, -149.90;

HRMS-ESI (m/z) $[M-BF_4]^+$ calc'd for $C_{17}H_{15}O_2S_2^+$, 315.0508, found 315.0509.

3.2 General Procedure for the Alkenes

Standard Procedure A:

Sodium sulfinate (1.5 equiv) and vinyl thianthrenium salt (1.0 equiv) were placed in a 10.0 mL Schlenk tube which equipped with a magnetic stir bar. After back-filled with nitrogen (this process was repeated three times), DCE (0.1 M) was added. The vial was sealed and at room temperature with stirring until TLC indicated the complete consumption of thianthrene (typically 10 h). The reaction mixture was evaporated and purified directly by column chromatography to afford the product.

Standard Procedure B:

Sodium sulfinate (1.5 equiv) and vinyl thianthrenium salt (1.0 equiv) were placed in a 10.0 mL Schlenk tube which equipped with a magnetic stir bar. After back-filled with nitrogen (this process was repeated three times), DCE (0.1 M) was added. The vial was sealed and at 50 ºC with stirring until TLC indicated the complete consumption of thianthrene (typically 10 h). The reaction mixture was evaporated and purified directly by column chromatography to afford the product.

Standard Procedure C:

$Zn(CN)_2$ (1.5 equiv), KF (3.0 equiv) and vinyl thianthrenium salt (1.0 equiv) were placed in a 10.0 mL Schlenk tube which equipped with a magnetic stir bar. After back-filled with nitrogen (this process was repeated three times), CH_3CN (0.1 M) was added. The vial was sealed and at room temperature with stirring until TLC indicated the complete consumption of thianthrene (typically 10 h). The reaction mixture was evaporated and purified directly by column chromatography to afford the product.

Standard Procedure D:

$Zn(CN)_2$ (1.5 equiv), KF (3.0 equiv) and vinyl thianthrenium salt (1.0 equiv) were placed in a 10.0 mL Schlenk tube which equipped with a magnetic stir bar. After back-filled with nitrogen (this process was repeated three times), CH_3CN (0.1 M) was added. The vial was sealed and at 50 °C with stirring until TLC indicated the complete consumption of thianthrene (typically 10 h). The reaction mixture was evaporated and purified directly by column chromatography to afford the product.

Standard Procedure E:

Nucleophile (1.5 equiv), K_2CO_3 (1.0 equiv) and vinyl thianthrenium salt (1.0 equiv) were placed in a 10.0 mL Schlenk tube which equipped with a magnetic stir bar. After back-filled with nitrogen (this process was repeated three times), CH_3CN (0.1 M) was added. The vial was sealed and at room temperature with stirring until TLC indicated the complete consumption of thianthrene (typically 10 h). The reaction mixture was evaporated and purified directly by column chromatography to afford the product.

Standard Procedure F:

Nucleophile (1.5 equiv), Cs_2CO_3 (1.0 equiv) and vinyl thianthrenium salt (1.0 equiv) were placed in a 10.0 mL Schlenk tube which equipped with a magnetic stir bar. After back-filled with nitrogen (this process was repeated three times), CH_3CN (0.1 M) was added. The vial was sealed and at room temperature with stirring until TLC indicated the complete consumption of thianthrene (typically 10 h). The reaction mixture was evaporated and purified directly by column chromatography to afford the product.

3.3 Gram Scale Synthesis

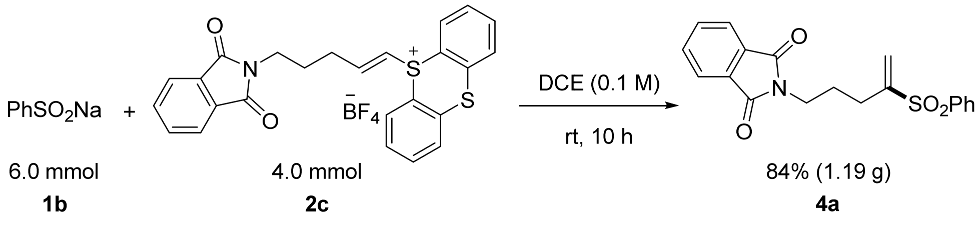

Sodium benzene sulfinate **1b** (985.0 mg, 6.0 mmol, 1.5 equiv), and (*E*)-5-(5-(1,3-dioxoisoindolin-2-yl)pent-1-en-1-yl)-5*H*-thianthren-5-ium tetrafluoroborate **2c** (2069.4 mg, 4.0 mmol, 1.0 equiv) were placed in 100 mL Schlenk flask equipped with a magnetic stirring bar. After backfilled with nitrogen (this process was repeated three times), 40.0 mL DCE was added. The flask was sealed at room temperature with stirring for 10 h. The reaction mixture was filtrated and washed with DCM (3 x 20.0 mL). The organic phase was purified directly by column chromatography (EA: PE = 0-20%) to afford **4a** as white solid (1193.1 mg, 84% yield).

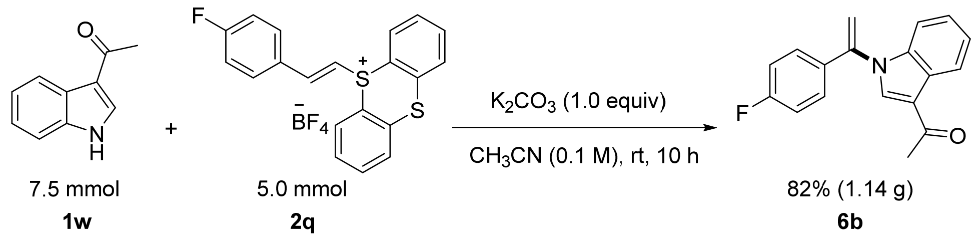

3-Acetylindole **1w** (1208.7 mg, 7.5 mmol, 1.5 equiv), (*E*)-5-(4 -fluorostyryl)-5*H*-thianthren-5-ium tetrafluoroborate **2q** (2121.3 mg, 5.0 mmol, 1.0 equiv) and K_2CO_3 (691.1 mg, mmol, 1.0 equiv) were placed in 100 mL Schlenk flask equipped with a magnetic stirring bar. After backfilled with nitrogen (this process was repeated three times), 50.0 mL CH_3CN was added. The flask was sealed at room temperature with stirring for 10 h. The reaction mixture was filtrated and washed with DCM (3 x 20.0 mL). The organic phase was purified directly by column chromatography (EA: PE = 0-20%) to afford **6b** as lightly yellow solid (1145.3 mg, 82% yield).

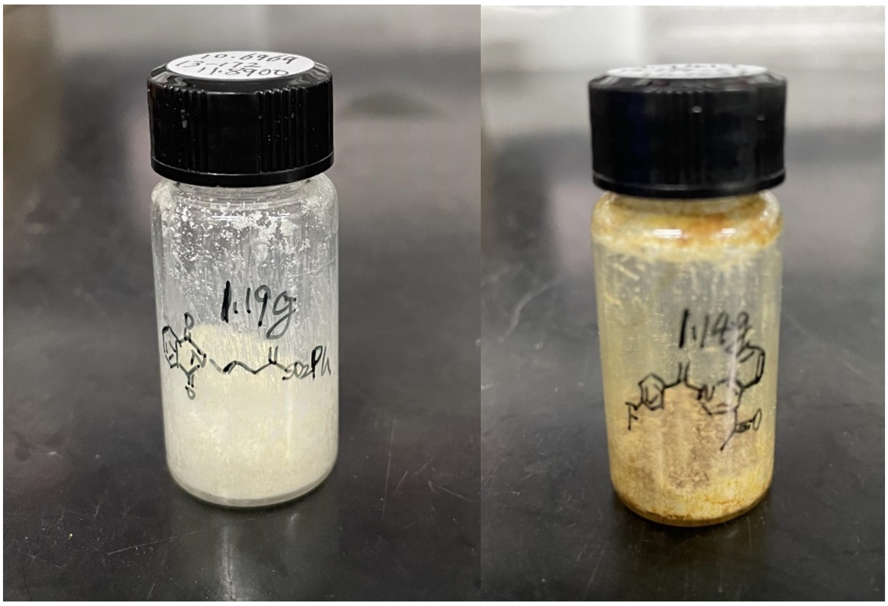

3.4 Characterization of Cross-Coupling Products

(3-(Methylsulfonyl)but-3-en-1-yl)benzene (3a)

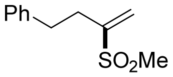

Following the Standard Procedure A, the reaction of Sodium methyl sulfinate **1a** (15.6 mg, 0.15 mmol, 1.5 equiv) and (*E*)-5-(4-phenylbut-1-en-1-yl)-5*H*-thianthren-5-ium tetrafluoroborate **2a** (43.4 mg, 0.10 mmol, 1.0 equiv) for 10 h afforded product after flash chromatography EA: PE = 0-20% as white solid **3a** (17.5 mg, 83% yield).

^{1}H NMR (400 MHz, $CDCl_3$) δ 7.35 – 7.28 (m, 2H), 7.25 – 7.19 (m, 3H), 6.29 (d, *J* = 1.1 Hz, 1H), 5.75 (d, *J* = 1.1 Hz, 1H), 2.95 (t, *J* = 7.8 Hz, 2H), 2.85 (s, 3H), 2.76 (t, *J* = 7.8 Hz, 2H);

^{13}C NMR (101 MHz, $CDCl_3$) δ 149.5, 140.0, 128.7, 128.6, 126.7, 124.7, 40.8, 34.2, 31.6;

HRMS-ESI (m/z) $[M+H]^+$ calc'd for $C_{11}H_{15}O_2S^+$, 211.0787, found 211.0785.

2-(Methylsulfonyl)oct-1-ene (3b)

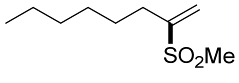

Following the Standard Procedure A, the reaction of Sodium methyl sulfinate **1a** (15.6 mg, 0.15 mmol, 1.5 equiv) and (*E*)-5-(oct-1-en-1-yl)-5*H*-thianthren-5-ium tetrafluoroborate **2b** (41.4 mg, 0.10 mmol, 1.0 equiv) for 10 h afforded product after flash chromatography EA: PE = 0-20% as colorless oil **3b** (14.1 mg, 74% yield).

^{1}H NMR (600 MHz, $CDCl_3$) δ 6.26 (s, 1H), 5.76 (s, 1H), 2.90 (s, 3H), 2.43 (t, *J* = 7.8

Hz, 2H), 1.61 (p, J = 7.4 Hz, 2H), 1.43 – 1.35 (m, 2H), 1.34 – 1.27 (m, 4H), 0.89 (t, J = 6.9 Hz, 3H);

^{13}C NMR (151 MHz, $CDCl_3$) δ 150.6, 123.6, 41.0, 31.6, 29.8, 28.8, 27.8, 22.7, 14.2;

HRMS-ESI (m/z) $[M+H]^+$ calc'd for $C_9H_{19}O_2S^+$, 191.1100, found 191.1100.

2-(4-(Methylsulfonyl)pent-4-en-1-yl)isoindoline-1,3-dione (3c)

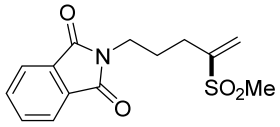

Following the Standard Procedure A, the reaction of Sodium methyl sulfinate **1a** (15.6 mg, 0.15 mmol, 1.5 equiv) and (E)-5-(5-(1,3-dioxoisoindolin-2-yl)pent-1-en-1-yl)-5H-thianthren-5-ium tetrafluoroborate **2c** (51.7 mg, 0.10 mmol, 1.0 equiv) for 10 h afforded product after flash chromatography EA: PE = 0-20% as white solid **3c** (25.7 mg, 88% yield).

^{1}H NMR (400 MHz, $CDCl_3$) δ 7.87 – 7.81 (m, 2H), 7.72 (m, 2H), 6.30 (s, 1H), 5.86 (s, 1H), 3.76 (t, J = 7.1 Hz, 2H), 2.91 (s, 3H), 2.51 (t, J = 7.4 Hz, 2H), 2.02 (tt, J = 7.4, 7.1 Hz, 2H);

^{13}C NMR (101 MHz, $CDCl_3$) δ 168.4, 149.3, 134.3, 132.1, 124.5, 123.5, 41.1, 37.1, 27.1, 26.7;

HRMS-ESI (m/z) $[M+H]^+$ calc'd for $C_{14}H_{16}NO_4S^+$, 294.0795, found 294.0792.

7-Bromo-2-(methylsulfonyl)hept-1-ene (3d)

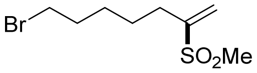

Following the Standard Procedure A, the reaction of Sodium methyl sulfinate **1a** (15.6 mg, 0.15 mmol, 1.5 equiv) and (E)-5-(7-bromohept-1-en-1-yl)-5H-thianthren-5-ium tetrafluoroborate **2d** (47.9 mg, 0.10 mmol, 1.0 equiv) for 10 h afforded product after flash chromatography EA: PE = 0-20% as colorless oil **3d** (19.6 mg, 77% yield).

^{1}H NMR (400 MHz, $CDCl_3$) δ 6.27 (s, 1H), 5.77 (s, 1H), 3.42 (t, J = 6.6 Hz, 2H), 2.91 (s, 3H), 2.46 (t, J = 7.6 Hz, 2H), 1.91 (p, J = 6.8 Hz, 2H), 1.71 – 1.62 (m, 2H), 1.59 – 1.49 (m, 2H);

^{13}C NMR (101 MHz, $CDCl_3$) δ 150.2, 124.0, 41.0, 33.6, 32.4, 29.7, 27.6, 27.1;

HRMS-ESI (m/z) $[M+H]^+$ calc'd for $C_8H_{16}BrO_2S^+$, 255.0049, found 255.0047.

2-(Methylsulfonyl)prop-1-ene (3e)

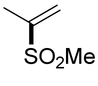

Following the Standard Procedure A, the reaction of Sodium methyl

sulfinate **1a** (15.6 mg, 0.15 mmol, 1.5 equiv) and (*E*)-5-(prop-1-en-1-yl)-5*H*-thianthren-5-ium tetrafluoroborate **2e** (34.4 mg, 0.10 mmol, 1.0 equiv) for 10 h afforded product after flash chromatography EA: PE = 0-20% as colorless oil **3e** (6.3 mg, 52% yield).

^{1}H NMR (400 MHz, $CDCl_3$) δ 6.18 (s, 1H), 5.76 (q, *J* = 1.6 Hz, 1H), 2.91 (s, 3H), 2.18 (t, *J* = 1.6 Hz, 3H);

^{13}C NMR (101 MHz, $CDCl_3$) δ 145.9, 125.0, 40.2, 16.8;

HRMS-ESI (m/z) $[M+H]^+$ calc'd for $C_4H_9O_2S^+$, 121.0318, found 121.0319.

(2-(Methylsulfonyl)allyl)benzene (3f)

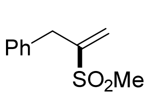

Following the Standard Procedure A, the reaction of Sodium methyl sulfinate **1a** (15.6 mg, 0.15 mmol, 1.5 equiv) and (*E*)-5-(3-phenylprop-1-en-1-yl)-5*H*-thianthren-5-ium tetrafluoroborate **2f** (42.0 mg, 0.10 mmol, 1.0 equiv) for 10 h afforded product after flash chromatography EA: PE = 0-20% as colorless oil **3f** (10.8 mg, 55% yield).

^{1}H NMR (400 MHz, $CDCl_3$) δ 7.36 (dd, *J* = 8.1, 6.5 Hz, 2H), 7.32 – 7.28 (m, 1H), 7.27 – 7.24 (m, 2H), 6.31 (s, 1H), 5.67 (s, 1H), 3.81 (s, 2H), 2.68 (s, 3H);

^{13}C NMR (101 MHz, $CDCl_3$) δ 150.4, 136.0, 129.4, 129.1, 127.6, 126.2, 42.1, 36.8;

HRMS-ESI (m/z) $[M+H]^+$ calc'd for $C_{10}H_{13}O_2S^+$, 197.0631, found 197.0631.

9-(Methylsulfonyl)dec-9-en-1-yl benzoate (3g)

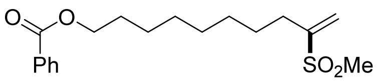

Following the Standard Procedure A, the reaction of Sodium methyl sulfinate **1a** (15.6 mg, 0.15 mmol, 1.5 equiv) and (*E*)-5-(10-(benzoyloxy)dec-1-en-1-yl)-5*H*-thianthren-5-ium tetrafluoro -borate **2g** (56.2 mg, 0.10 mmol, 1.0 equiv) for 10 h afforded product after flash chromatography EA: PE = 0-20% as colorless oil **3g** (24.5 mg, 72% yield).

^{1}H NMR (400 MHz, $CDCl_3$) δ 8.07 – 8. 01 (m, 2H), 7.58 – 7.52 (m, 1H), 7.47 – 7.40 (m, 2H), 6.25 (s, 1H), 5.75 (s, 1H), 4.31 (t, *J* = 6.6 Hz, 2H), 2.89 (s, 3H), 2.43 (t, *J* = 7.9 Hz, 2H), 1.77 (tt, *J* = 6.9, 6.6 Hz, 2H), 1.62 (tt, *J* = 7.9, 7.3 Hz, 2H), 1.47 – 1.35 (m, 8H);

^{13}C NMR (101 MHz, $CDCl_3$) δ 166.8, 150.5, 133.0, 130.6, 129.6, 128.5, 123.7, 65.1, 41.0, 29.7, 29.3, 29.2, 29.1, 28.8, 27.8, 26.1;

HRMS-ESI (m/z) $[M+H]^+$ calc'd for $C_{18}H_{27}O_4S^+$, 339.1625, found 339.1622.

N-(3-(Methylsulfonyl)but-3-en-1-yl)benzamide (3h)

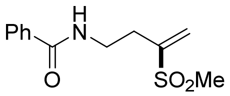

Following the Standard Procedure A, the reaction of Sodium methyl sulfinate **1a** (15.6 mg, 0.15 mmol, 1.5 equiv) and (*E*)-5-(4-benzamidobut-1-en-1-yl)-5*H*-thianthren-5-ium tetrafluoroborate **2h** (47.7 mg, 0.10 mmol, 1.0 equiv) for 10 h afforded product after flash chromatography EA: PE = 0-20% as white solid **3h** (17.9 mg, 71% yield).

^{1}H NMR (400 MHz, $CDCl_3$) δ 7.80 – 7.74 (m, 2H), 7.53 – 7.46 (m, 1H), 7.45 – 7.38 (m, 2H), 6.81 (bs, 1H), 6.34 (s, 1H), 5.90 (s, 1H), 3.74 (dt, J = 6.5, 6.2 Hz, 2H), 2.97 (s, 3H), 2.81 (t, J = 6.5 Hz, 2H);

^{13}C NMR (101 MHz, $CDCl_3$) δ 167.8, 147.6, 134.1, 131.8, 128.8, 127.0, 126.9, 40.9, 39.2, 30.1;

HRMS-ESI (m/z) $[M+H]^+$ calc'd for $C_{12}H_{16}NO_3S^+$, 254.0845, found 254.0843.

9-(Methylsulfonyl)dec-9-en-1-yl 2,2,2-trifluoroacetate (3i)

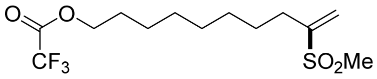

Following the Standard Procedure A, the reaction of Sodium methyl sulfinate **1a** (15.6 mg, 0.15 mmol, 1.5 equiv) and (*E*)-5-(10-(2,2,2-trifluoroacetoxy)dec-1-en-1-yl)-5*H*-thianthren-5-ium tetrafluoroborate **2i** (55.4 mg, 0.10 mmol, 1.0 equiv) for 10 h afforded product after flash chromatography EA: PE = 0-20% as colorless oil **3i** (22.9 mg, 69% yield).

^{1}H NMR (400 MHz, $CDCl_3$) δ 6.25 (s, 1H), 5.75 (s, 1H), 4.34 (t, J = 6.6 Hz, 2H), 2.90 (s, 3H), 2.43 (t, J = 7.7 Hz, 2H), 1.74 (tt, J = 7.2, 6.6 Hz, 2H), 1.61 (tt, J = 7.7, 7.0 Hz, 2H), 1.43 – 1.31 (m, 8H);

^{13}C NMR (101 MHz, $CDCl_3$) δ 157.7 (q, J = 42.0 Hz), 150.5, 123.7, 114.7 (q, J = 285.7 Hz), 68.3, 41.0, 29.7, 29.2, 29.01, 28.99, 28.2, 27.8, 25.6;

^{19}F NMR (376 MHz, $CDCl_3$) δ -75.13;

HRMS-ESI (m/z) $[M+H]^+$ calc'd for $C_{13}H_{22}F_3O_4S^+$, 331.1185, found 331.1183.

9-(Methylsulfonyl)dec-9-en-1-ol (3j)

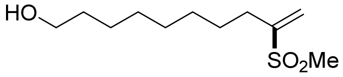

Following the Standard Procedure A, the reaction of Sodium methyl sulfinate **1a** (15.6 mg, 0.15 mmol, 1.5 equiv) and (*E*)-5-(10-hydroxydec-1-en-1-yl)-5*H*-thianthren-5-ium tetrafluoroborate **2j** (45.8 mg, 0.10 mmol, 1.0 equiv) for 10 h afforded product after flash chromatography EA: PE = 0-20% as white solid **3j** (17.5 mg, 75% yield).

^{1}H NMR (400 MHz, $CDCl_3$) δ 6.25 (s, 1H), 5.75 (s, 1H), 3.63 (t, J = 6.6 Hz, 2H),

2.90 (s, 3H), 2.42 (t, J = 7.6 Hz, 2H), 1.65 – 1.51 (m, 4H), 1.43 – 1.29 (m, 8H);
^{13}C NMR (101 MHz, $CDCl_3$) δ 150.5, 123.7, 63.1, 41.0, 32.8, 29.7, 29.3, 29.0, 27.8, 25.8;
HRMS-ESI (m/z) $[M+H]^+$ calc'd for $C_{11}H_{23}O_3S^+$, 235.1362, found 235.1360.

2-(Methylsulfonyl)octa-1,7-diene (3k)

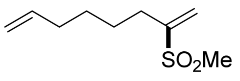

Following the Standard Procedure A, the reaction of Sodium methyl sulfinate **1a** (15.6 mg, 0.15 mmol, 1.5 equiv) and (*E*)-5-(octa-1,7-dien-1-yl)-5*H*-thianthren-5-ium tetrafluoroborate **2k** (41.2 mg, 0.10 mmol, 1.0 equiv) for 10 h afforded product after flash chromatography EA: PE = 0-20% as colorless oil **3k** (13.1 mg, 70% yield).
^{1}H NMR (400 MHz, $CDCl_3$) δ 6.26 (s, 1H), 5.79 (ddt, J = 17.0, 10.2, 6.7 Hz, 1H), 5.76 (s, 1H), 5.02 (dd, J = 17.0, 1.8 Hz, 1H), 4.97 (dd, J = 10.2, 1.8 Hz, 1H), 2.90 (s, 3H), 2.44 (t, J = 7.7 Hz, 2H), 2.10 (dt, J = 7.0, 6.7 Hz, 2H), 1.64 (tt, J = 8.3, 7.0 Hz, 2H), 1.49 (tt, J = 8.3, 7.7 Hz, 2H);
^{13}C NMR (101 MHz, $CDCl_3$) δ 150.4, 138.3, 123.8, 115.1, 41.0, 33.5, 29.6, 28.3, 27.2;
HRMS-ESI (m/z) $[M+Na]^+$ calc'd for $C_9H_{16}NaO_2S^+$, 211.0763, found 211.0764.

(1-(Methylsulfonyl)vinyl)cyclohexane (3l)

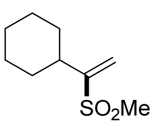

Following the Standard Procedure A, the reaction of Sodium methyl sulfinate **1a** (15.6 mg, 0.15 mmol, 1.5 equiv) and (*E*)-5-(2-cyclohexylvinyl)-5*H*-thianthren-5-ium tetrafluoroborate **2l** (41.2 mg, 0.10 mmol, 1.0 equiv) for 24 h afforded product after flash chromatography EA: PE = 0-20% as colorless oil **3l** (15.3 mg, 81% yield).
^{1}H NMR (400 MHz, $CDCl_3$) δ 6.27 (s, 1H), 5.79 (s, 1H), 2.90 (s, 3H), 2.46 (tt, J = 11.5, 3.4 Hz, 1H), 1.98 (ddt, J = 11.5, 3.3, 1.7 Hz, 2H), 1.82 (dt, J = 12.9, 3.3 Hz, 2H), 1.73 (ddt, J = 12.9, 3.4, 1.7 Hz, 1H), 1.41 – 1.19 (m, 5H);
^{13}C NMR (101 MHz, $CDCl_3$) δ 156.3, 123.1, 41.8, 38.8, 33.9, 26.6, 25.8;
HRMS-ESI (m/z) $[M+H]^+$ calc'd for $C_9H_{17}O_2S^+$, 189.0944, found 189.0944.

4-(1-(Methylsulfonyl)vinyl)-4'-propyl-1,1'-bi(cyclohexane) (3m)

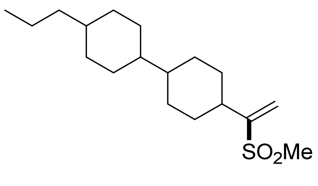

Following the Standard Procedure A, the reaction of Sodium methyl sulfinate **1a** (15.6 mg, 0.15 mmol, 1.5

equiv) and (*E*)-5-(2-(4'-propyl-[1,1'-bi(cyclohexan)]-4-yl) vinyl)-5*H*-thianthren-5-ium tetrafluoroborate **2m** (53.7 mg, 0.10 mmol, 1.0 equiv) for 24 h afforded product after flash chromatography EA: PE = 0-20% as white solid **3m** (26.5 mg, 85% yield).

^{1}H NMR (400 MHz, $CDCl_3$) δ 6.26 (s, 1H), 5.78 (s, 1H), 2.90 (s, 3H), 2.41 (tt, *J* = 12.0, 3.3 Hz, 1H), 2.08 – 1.98 (m, 2H), 1.86 – 1.64 (m, 6H), 1.35 – 1.21 (m, 4H), 1.19 – 1.08 (m, 6H), 1.07 – 0.91 (m, 3H), 0.90 – 0.77 (m, 5H);

^{13}C NMR (101 MHz, $CDCl_3$) δ 156.2, 122.9, 43.3, 42.8, 41.8, 39.9, 38.9, 37.7, 34.0, 33.6, 30.13, 30.06, 20.2, 14.5;

HRMS-ESI (m/z) $[M+H]^+$ calc'd for $C_{18}H_{33}O_2S^+$, 313.2196, found 313.2192.

(1-(Methylsulfonyl)vinyl)benzene (3n)

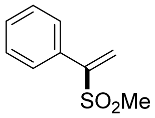

Following the Standard Procedure A, the reaction of Sodium methyl sulfinate **1a** (15.6 mg, 0.15 mmol, 1.5 equiv) and (*E*)-5-styryl-5*H*-thianthren-5-ium tetrafluoroborate **2n** (40.6 mg, 0.10 mmol, 1.0 equiv) for 10 h afforded product after flash chromatography EA: PE = 0-20% as white solid **3n** (13.0 mg, 71% yield).

^{1}H NMR (600 MHz, $CDCl_3$) δ 7.63 (d, *J* = 7.5 Hz, 2H), 7.47 – 7.41 (m, 3H), 6.54 (s, 1H), 6.02 (s, 1H), 2.80 (s, 3H);

^{13}C NMR (151 MHz, $CDCl_3$) δ 150.2, 132.6, 130.0, 129.0, 128.8, 126.1, 40.8;

HRMS-ESI (m/z) $[M+H]^+$ calc'd for $C_9H_{11}O_2S^+$, 183.0474, found 183.0473.

1-Bromo-4-(1-(methylsulfonyl)vinyl)benzene (3o)

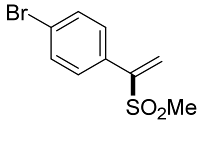

Following the Standard Procedure A, the reaction of Sodium methyl sulfinate **1a** (15.6 mg, 0.15 mmol, 1.5 equiv) and (*E*)-5-(4-bromostyryl)-5*H*-thianthren-5-ium tetrafluoroborate **2o** (48.5 mg, 0.10 mmol, 1.0 equiv) for 10 h afforded product after flash chromatography EA: PE = 0-20% as light yellow solid **3o** (22.7 mg, 87% yield).

^{1}H NMR (600 MHz, $CDCl_3$) δ 7.59 – 7.54 (m, 2H), 7.53 – 7.48 (m, 2H), 6.55 (s, 1H), 6.03 (s, 1H), 2.80 (s, 3H);

^{13}C NMR (151 MHz, $CDCl_3$) δ 149.3, 132.3, 131.5, 130.3, 126.6, 124.6, 40.8;

HRMS-ESI (m/z) $[M+H]^+$ calc'd for $C_9H_{10}BrO_2S^+$, 260.9579, found 260.9577.

1-Chloro-4-(1-(methylsulfonyl)vinyl)benzene (3p)

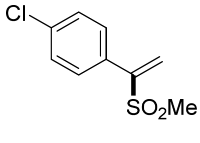

Following the Standard Procedure A, the reaction of Sodium methyl

sulfinate **1a** (15.6 mg, 0.15 mmol, 1.5 equiv) and (*E*)-5-(4-chlorostyryl)-5*H*-thianthren-5-ium tetrafluoroborate **2p** (44.1 mg, 0.10 mmol, 1.0 equiv) for 10 h afforded product after flash chromatography EA: PE = 0-20% as white solid **3p** (13.2 mg, 61% yield).

^{1}H NMR (400 MHz, $CDCl_3$) δ 7.58 (d, *J* = 8.5 Hz, 2H), 7.41 (d, *J* = 8.5 Hz, 2H), 6.55 (s, 1H), 6.03 (s, 1H), 2.80 (s, 3H);

^{13}C NMR (101 MHz, $CDCl_3$) δ 149.2, 136.4, 131.0, 130.1, 129.3, 126.6, 40.8;

HRMS-ESI (m/z) $[M+H]^+$ calc'd for $C_9H_{10}ClO_2S^+$, 217.0085, found 217.0082.

1-Fluoro-4-(1-(methylsulfonyl)vinyl)benzene (3q)

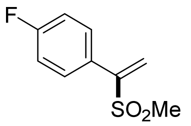

Following the Standard Procedure A, the reaction of Sodium methyl sulfinate **1a** (15.6 mg, 0.15 mmol, 1.5 equiv) and (*E*)-5-(4-fluorostyryl)-5*H*-thianthren-5-ium tetrafluoroborate **2q** (42.4 mg, 0.10 mmol, 1.0 equiv) for 10 h afforded product after flash chromatography EA: PE = 0-20% as white solid **3q** (16.8 mg, 84% yield).

^{1}H NMR (400 MHz, $CDCl_3$) δ 7.67 – 7.58 (m, 2H), 7.16 – 7.07 (m, 2H), 6.54 (s, 1H), 6.01 (s, 1H), 2.79 (s, 3H);

^{13}C NMR (101 MHz, $CDCl_3$) δ 163.8 (d, J = 250.7 Hz), 149.3, 130.9 (d, J = 8.4 Hz), 128.6 (d, J = 3.5 Hz), 126.3, 116.2 (d, J = 21.8 Hz), 40.7;

^{19}F NMR (376 MHz, $CDCl_3$) δ -110.61;

HRMS-ESI (m/z) $[M+H]^+$ calc'd for $C_9H_{10}FO_2S^+$, 201.0380, found 201.0378.

4-(1-(Methylsulfonyl)vinyl)phenyl acetate (3r)

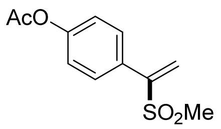

Following the Standard Procedure A, the reaction of Sodium methyl sulfinate **1a** (15.6 mg, 0.15 mmol, 1.5 equiv) and (*E*)-5-(4-acetoxystyryl)-5*H*-thianthren-5-ium tetrafluoroborate **2r** (46.4 mg, 0.10 mmol, 1.0 equiv) for 10 h afforded product after flash chromatography EA: PE = 0-20% as white solid **3r** (22.2 mg, 92% yield).

^{1}H NMR (600 MHz, $CDCl_3$) δ 7.68 – 7.63 (m, 2H), 7.18 – 7.14 (m, 2H), 6.54 (s, 1H), 6.02 (s, 1H), 2.81 (s, 3H), 2.32 (s, 3H);

^{13}C NMR (151 MHz, $CDCl_3$) δ 169.3, 152.0, 149.4, 130.1, 130.0, 126.4, 122.3, 40.8, 21.3;

HRMS-ESI (m/z) $[M+H]^+$ calc'd for $C_{11}H_{13}O_4S^+$, 241.0529, found 241.0526.

1,4-Dimethyl-2-(1-(methylsulfonyl)vinyl)benzene (3s)

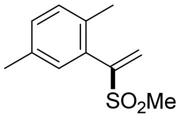

Following the Standard Procedure B, the reaction of Sodium methyl sulfinate **1a** (15.6 mg, 0.15 mmol, 1.5 equiv) and (*E*)-5-(2,5-dimethylstyryl)-5*H*-thianthren-5-ium tetrafluoroborate **2s** (43.4 mg, 0.10 mmol, 1.0 equiv) for 10 h at 50 °C afforded product after flash chromatography EA: PE = 0-20% as white solid **3s** (13.8 mg, 66% yield).

^{1}H NMR (400 MHz, $CDCl_3$) δ 7.20 – 7.12 (m, 3H), 6.67 (s, 1H), 5.87 (s, 1H), 2.80 (s, 3H), 2.33 (s, 3H), 2.31 (s, 3H);

^{13}C NMR (101 MHz, $CDCl_3$) δ 149.6, 135.6, 134.3, 131.7, 130.82, 130.80, 130.6, 127.7, 40.0, 21.0, 19.7;

HRMS-ESI (m/z) $[M+H]^+$ calc'd for $C_{11}H_{15}O_2S^+$, 211.0787, found 211.0786.

1-(Methylsulfonyl)cyclopent-1-ene (3t)

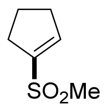

Following the Standard Procedure B, the reaction of Sodium methyl sulfinate **1a** (15.6 mg, 0.15 mmol, 1.5 equiv) and 5-(cyclopent-1-en-1-yl)-5*H*-thianthren-5-ium tetrafluoroborate **2t** (37.0 mg, 0.10 mmol, 1.0 equiv) for 36 h at 50 ºC afforded product after flash chromatography EA: PE = 0-20% as white solid **3t** (13.0 mg, 89% yield).

^{1}H NMR (400 MHz, $CDCl_3$) δ 6.75 (p, *J* = 2.2, Hz, 1H), 2.93 (s, 3H), 2.72 (ddt, *J* = 10.3, 7.4, 2.3 Hz, 2H), 2.59 (tq, *J* = 7.7, 2.6 Hz, 2H), 2.19 – 2.10 (m, 2H);

^{13}C NMR (101 MHz, $CDCl_3$) δ 144.1, 144.0, 41.4, 33.1, 31.3, 23.9;

HRMS-ESI (m/z) $[M+H]^+$ calc'd for $C_6H_{11}O_2S^+$, 147.0474, found 147.0474.

1-(Methylsulfonyl)cyclohex-1-ene (3u)

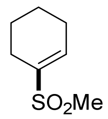

Following the Standard Procedure B, the reaction of Sodium methyl sulfinate **1a** (15.6 mg, 0.15 mmol, 1.5 equiv) and 5-(cyclohex-1-en-1-yl)-5*H*-thianthren-5-ium tetrafluoroborate **2u** (38.4 mg, 0.10 mmol, 1.0 equiv) for 36 h at 50 ºC afforded product after flash chromatography EA: PE = 0-20% as white solid **3u** (9.4 mg, 59% yield).

^{1}H NMR (400 MHz, $CDCl_3$) δ 6.94 (tt, *J* = 3.7, 1.8 Hz, 1H), 2.86 (s, 3H), 2.38 (dt, *J* = 6.2, 1.8 Hz, 2H), 2.28 (dt, *J* = 6.2, 3.7 Hz, 2H), 1.82 – 1.73 (m, 2H), 1.71 – 1.61 (m, 2H);

^{13}C NMR (101 MHz, $CDCl_3$) δ 139.5, 139.1, 40.5, 25.5, 23.4, 22.0, 20.9;

HRMS-ESI (m/z) $[M+H]^+$ calc'd for $C_7H_{13}O_2S^+$, 161.0631, found 161.0630.

6-(Methylsulfonyl)-1,2,3,4,4a,5,8,8a-octahydro-1,4-methanonaphthalene (3v)

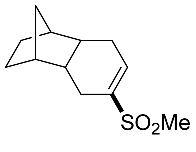

Following the Standard Procedure B, the reaction of Sodium methyl sulfinate **1a** (15.6 mg, 0.15 mmol, 1.5 equiv) and 5-(1,2,3,4,4a,5,8,8a-octahydro-1,4-methanonaphthalen-6-yl)-5*H*-thianthren-5-ium tetrafluoroborate **2v** (45.0 mg, 0.10 mmol, 1.0 equiv) for 36 h at 50 ºC afforded product after flash chromatography EA: PE = 0-20% as white solid **3v** (19.1 mg, 84% yield).

^{1}H NMR (400 MHz, $CDCl_3$) δ 7.09 – 6.99 (m, 1H), 2.85 (s, 3H), 2.79 – 2.68 (m, 1H), 2.62 – 2.49 (m, 1H), 2.07 (s, 1H), 2.03 (s, 1H), 1.83 – 1.72 (m, 2H), 1.70 – 1.64 (m, 1H), 1.61 – 1.55 (m, 4H), 1.26 – 1.21 (m, 2H), 1.12 (d, *J* = 10.4 Hz, 1H);
^{13}C NMR (151 MHz, $CDCl_3$) δ 140.8, 140.4, 43.7, 43.2, 43.1, 42.5, 40.9, 33.5, 29.6, 29.5, 29.1, 27.1;
HRMS-ESI (m/z) $[M+H]^+$ calc'd for $C_{12}H_{19}O_2S^+$, 227.1100, found 227.1099.

(1*E*,5*Z*)-1-(methylsulfonyl)cycloocta-1,5-diene (3w)

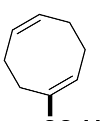

SO2Me

Following the Standard Procedure B, the reaction of Sodium methyl sulfinate **1a** (15.6 mg, 0.15 mmol, 1.5 equiv) and 5-((1*E*,5*Z*)-cycloocta-1,5-dien-1-yl)-5*H*-thianthren-5-ium tetrafluoroborate **2w** (41.0 mg, 0.10 mmol, 1.0 equiv) for 36 h at 50 °C afforded product after flash chromatography EA: PE = 0-20% as colorless oil **3w** (11.9 mg, 64% yield).
^{1}H NMR (400 MHz, $CDCl_3$) δ 6.96 (t, *J* = 6.1 Hz, 1H), 5.61 – 5.56 (m, 2H), 2.89 – 2.81 (m, 5H), 2.62 – 2.51 (m, 4H), 2.45 (q, *J* = 6.1 Hz, 2H);
^{13}C NMR (101 MHz, $CDCl_3$) δ 140.9, 140.5, 128.4, 128.2, 41.2, 28.8, 28.0, 26.0, 25.8;
HRMS-ESI (m/z) $[M+H]^+$ calc'd for $C_9H_{15}O_2S^+$, 187.0787, found 187.0787.

(Vinylsulfonyl)benzene (3x)

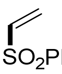

SO2Ph

Following the Standard Procedure A, the reaction of sodium benzene sulfinate **1a** (24.6 mg, 0.15 mmol, 1.5 equiv) and 5-vinyl-5*H*-thianthren-5-ium tetrafluoroborate **2x** (33.0 mg, 0.10 mmol, 1.0 equiv) for 10 h afforded product after flash chromatography EA: PE = 0-20% as white solid **3x** (11.9 mg, 71% yield).
^{1}H NMR (400 MHz, $CDCl_3$) δ 7.93 – 7.87 (m, 2H), 7.67 – 7.61 (m, 1H), 7.59 – 7.51 (m, 2H), 6.66 (dd, *J* = 16.6, 9.8 Hz, 1H), 6.46 (d, *J* = 16.6 Hz, 1H), 6.04 (d, *J* = 9.8 Hz, 1H);
^{13}C NMR (101 MHz, $CDCl_3$) δ 139.7, 138.6, 133.8, 129.5, 128.1, 127.9;
HRMS-ESI (m/z) $[M+H]^+$ calc'd for $C_8H_9O_2S^+$, 169.0318, found 169.0317.

(Prop-1-en-2-ylsulfonyl)benzene (3y)

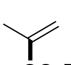

SO2Ph

Following the Standard Procedure A, the reaction of sodium benzene sulfinate **1a** (24.6 mg, 0.15 mmol, 1.5 equiv) and

(*E*)-5-(prop-1-en-1-yl)-5*H*-thianthren-5-ium tetrafluoroborate **2e** (34.4 mg, 0.10 mmol, 1.0 equiv) for 10 h afforded product after flash chromatography EA: PE = 0-20% as colorless oil **3y** (16.0 mg, 88% yield).

^{1}H NMR (400 MHz, $CDCl_3$) δ 7.94 – 7.85 (m, 2H), 7.69 – 7.60 (m, 1H), 7.58 – 7.50 (m, 2H), 6.28 (s, 1H), 5.71 (q, *J* = 1.6 Hz, 1H), 1.96 (dd, *J* = 1.6, 1.0 Hz, 3H);

^{13}C NMR (101 MHz, $CDCl_3$) δ 146.4, 138.6, 133.6, 129.3, 128.4, 124.4, 16.5;

HRMS-ESI (m/z) $[M+H]^+$ calc'd for $C_9H_{11}O_2S^+$, 183.0474, found 183.0473.

3-(Methylsulfonyl)but-3-en-1-yl (4*R*)-4-((3*R*,8*R*,9*S*,10*S*,13*R*,14*S*,17*R*)-3-acetoxy-10,13-dimethylhexadecahydro-1*H*-cyclopenta[*a*]phenanthren-17-yl)pentanoate (3z)

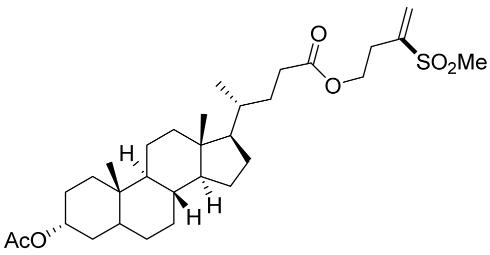

Following the Standard Procedure A, the reaction of Sodium methyl sulfinate **1a** (15.6 mg, 0.15 mmol, 1.5 equiv) and 5-((*E*)-4-(((4*R*)-4-((3*R*,8*R*,9*S*,10*S*,13*R*,14*S*,17*R*)-3-acetoxy-10,13-dimethylhexadecahydro-1H-cyclopenta[a]phenanthren-17-yl)pentanoyl)oxy)but-1-en-1-yl)-5*H*-thianthren-5-ium tetrafluoroborate **2y** (77.5 mg, 0.10 mmol, 1.0 equiv) for 10 h afforded product after flash chromatography EA: PE = 0-20% as white solid **3z** (38.8 mg, 70% yield).

^{1}H NMR (400 MHz, $CDCl_3$) δ 6.35 (s, 1H), 5.87 (s, 1H), 4.71 (tt, *J* = 11.1, 4.7 Hz, 1H), 4.33 (t, *J* = 6.6 Hz, 2H), 2.94 (s, 3H), 2.79 (t, *J* = 6.6 Hz, 2H), 2.34 (ddd, *J* = 15.3, 10.1, 5.1 Hz, 1H), 2.21 (ddd, *J* = 15.7, 9.6, 6.4 Hz, 1H), 2.02 (s, 3H), 1.98 – 1.91 (m, 1H), 1.89 – 1.73 (m, 5H), 1.72 – 1.64 (m, 1H), 1.60 – 1.49 (m, 2H), 1.48 – 1.19 (m, 11H), 1.08 (dddd, *J* = 29.1, 24.5, 14.9, 11.6 Hz, 6H), 0.92 (s, 3H), 0.90 (d, *J* = 6.9 Hz, 3H), 0.63 (s, 3H);

^{13}C NMR (101 MHz, $CDCl_3$) δ 174.1, 170.8, 146.8, 126.3, 74.5, 61.6, 56.6, 56.1, 42.9, 42.0, 41.1, 40.5, 40.3, 35.9, 35.5, 35.2, 34.7, 32.4, 31.3, 31.1, 29.4, 28.3, 27.1, 26.8, 26.4, 24.3, 23.5, 21.6, 21.0, 18.4, 12.2;

HRMS-ESI (m/z) $[M+Na]^+$ calc'd for $C_{31}H_{50}NaO_6S^+$, 573.3220, found 573.3216.

Ethyl 3-(methylsulfonyl)acrylate (3aa)

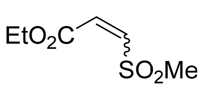

Following the Standard Procedure A, the reaction of sodium methyl sulfinate **1a** (15.6 mg, 0.15 mmol, 1.5 equiv) and

(*E*)-5-(3-ethoxy-3-oxoprop-1-en-1-yl)-5*H*-thianthren-5-ium tetrafluoroborate **2dd** (40.2 mg, 0.10 mmol, 1.0 equiv) for 10 h at room temperature afforded product after flash chromatography EA: PE = 0-30% as colorless oil **3aa** (9.8 mg, 55% yield, Z/E = 4:1).

^{1}H NMR (500 MHz, $CDCl_3$) δ 6.68 (d, *J* = 11.6 Hz, 1H), 6.59 (d, *J* = 11.6 Hz, 1H), 4.32 (q, *J* = 7.2 Hz, 2H), 3.17 (s, 3H), 1.35 (t, *J* = 7.2 Hz, 3H).

^{13}C NMR (126 MHz, $CDCl_3$) δ 163.3, 138.1, 133.9, 62.4, 43.8, 14.1.

HRMS-ESI (m/z) $[M+H]^+$ calc'd for $C_6H_{11}O_4S^+$, 179.0373, found 179.0374.

2-(4-(Phenylsulfonyl)pent-4-en-1-yl)isoindoline-1,3-dione (4a)

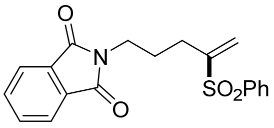

Following the Standard Procedure A, the reaction of sodium benzene sulfinate **1b** (24.6 mg, 0.15 mmol, 1.5 equiv) and (*E*)-5-(5-(1,3-dioxoisoindolin-2-yl)pent-1-en-1-yl)-5*H*-thianthren-5-ium tetrafluoroborate **2a** (51.7 mg, 0.10 mmol, 1.0 equiv) for 10 h afforded product after flash chromatography EA: PE = 0-20% as white solid **4a** (29.7 mg, 84% yield).

^{1}H NMR (600 MHz, $CDCl_3$) δ 7.87 – 7.83 (m, 2H), 7.81 (dd, *J* = 5.4, 3.0 Hz, 2H), 7.71 (dd, *J* = 5.5, 3.0 Hz, 2H), 7.63 – 7.57 (m, 1H), 7.51 (t, *J* = 7.8 Hz, 2H), 6.40 (s, 1H), 5.83 (s, 1H), 3.61 (t, *J* = 7.2 Hz, 2H), 2.29 (t, *J* = 7.7 Hz, 2H), 1.85 (p, *J* = 7.4 Hz, 2H);

^{13}C NMR (151 MHz, $CDCl_3$) δ 168.3, 149.5, 138.7, 134.2, 133.7, 132.1, 129.4, 128.4, 123.7, 123.4, 37.0, 26.7, 26.6;

HRMS-ESI (m/z) $[M+H]^+$ calc'd for $C_{19}H_{18}NO_4S^+$, 356.0951, found 356.0948.

2-(4-((4-Methoxyphenyl)sulfonyl)pent-4-en-1-yl)isoindoline-1,3-dione (4b)

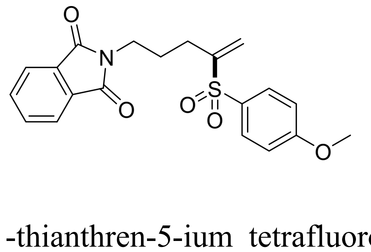

Following the Standard Procedure A, the reaction of sodium 4-methoxybenzenesulfinate **1c** (29.1 mg, 0.15 mmol, 1.5 equiv) and (*E*)-5-(5-(1,3-dioxoisoindolin-2-yl)pent-1-en-1-yl)-5*H*-thianthren-5-ium tetrafluoroborate **2a** (51.7 mg, 0.10 mmol, 1.0 equiv) for 10 h afforded product after flash chromatography EA: PE = 0-20% as white solid **4b** (32.8 mg, 85% yield).

^{1}H NMR (600 MHz, $CDCl_3$) δ 7.86 – 7.79 (m, 2H), 7.78 (dd, *J* = 8.8, 3.7 Hz, 2H),

7.73 – 7.68 (m, 2H), 6.96 (dd, J = 8.8, 2.9 Hz, 2H), 6.33 (s, 1H), 5.76 (s, 1H), 3.86 (s, 3H), 3.62 (t, J = 7.3 Hz, 2H), 2.29 (t, J = 7.8 Hz, 2H), 1.84 (tt, J = 7.8, 7.3 Hz, 2H);

^{13}C NMR (151 MHz, $CDCl_3$) δ 168.3, 163.8, 150.0, 134.2, 132.1, 130.6, 130.1, 123.4, 122.8, 114.6, 55.8, 37.1, 26.6;

HRMS-ESI (m/z) $[M+H]^+$ calc'd for $C_{20}H_{20}NO_5S^+$, 386.1057, found 386.1054.

***N*-(4-((5-(1,3-Dioxoisoindolin-2-yl)pent-1-en-2-yl)sulfonyl)phenyl)acetamide (4c)**

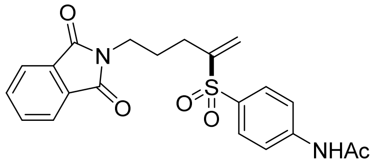

Following the Standard Procedure A, the reaction of sodium 4-acetamidobenzenesulfinate **1d** (33.2 mg, 0.15 mmol, 1.5 equiv) and (*E*)-5-(5-(1,3-dioxoiso-indolin-2-yl)pent-1-en-1-yl)-5*H*-thianthren-5-ium tetrafluoroborate **2a** (51.7 mg, 0.10 mmol, 1.0 equiv) for 24 h afforded product after flash chromatography EA: PE = 0-20% as white solid **4c** (39.7 mg, 96% yield).

^{1}H NMR (600 MHz, $CDCl_3$) δ 8.07 – 7.94 (bs, 1H), 7.81 (dd, J = 5.5, 3.0 Hz, 2H), 7.75 (dd, J = 9.0, 2.3 Hz, 2H), 7.72 – 7.65 (m, 4H), 6.35 (s, 1H), 5.81 (d, J = 2.0 Hz, 1H), 3.62 (t, J = 7.2 Hz, 2H), 2.28 (t, J = 7.7 Hz, 2H), 2.20 (s, 3H), 1.85 (p, J = 7.5 Hz, 2H);

^{13}C NMR (151 MHz, $CDCl_3$) δ 169.1, 168.4, 149.6, 143.2, 134.2, 132.0, 129.7, 123.5, 123.4, 119.5, 37.1, 26.7, 26.6, 24.8;

HRMS-ESI (m/z) $[M+H]^+$ calc'd for $C_{21}H_{21}N_2O_5S^+$, 413.1166, found 413.1163.

2-(4-Tosylpent-4-en-1-yl)isoindoline-1,3-dione (4d)

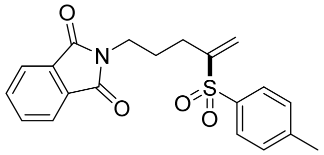

Following the Standard Procedure A, the reaction of Sodium *p*-toluenesulfinate **1e** (26.7 mg, 0.15 mmol, 1.5 equiv) and (*E*)-5-(5-(1,3-dioxoisoindolin-2-yl)pent-1-en-1-yl)-5*H*-thianthren-5-ium tetrafluoroborate **2a** (51.7 mg, 0.10 mmol, 1.0 equiv) for 10 h afforded product after flash chromatography EA: PE = 0-20% as white solid **4d** (30.3 mg, 82% yield).

^{1}H NMR (400 MHz, $CDCl_3$) δ 7.82 (dd, J = 5.4, 3.0 Hz, 2H), 7.74 – 7.69 (m, 4H), 7.29 (d, J = 8.0 Hz, 2H), 6.37 (s, 1H), 5.79 (s, 1H), 3.61 (t, J = 7.1 Hz, 2H), 2.41 (s, 3H), 2.28 (t, J = 7.8 Hz, 2H), 1.84 (tt, J = 7.8, 7.1 Hz, 2H);

^{13}C NMR (101 MHz, $CDCl_3$) δ 168.3, 149.7, 144.7, 135.7, 134.2, 132.1, 130.0, 128.4, 123.4, 123.2, 37.1, 26.7, 26.6, 21.7;

HRMS-ESI (m/z) $[M+H]^+$ calc'd for $C_{20}H_{20}NO_4S^+$, 370.1108, found 370.1105.

2-(4-((4-(Trifluoromethyl)phenyl)sulfonyl)pent-4-en-1-yl)isoindoline-1,3-dione (4e)

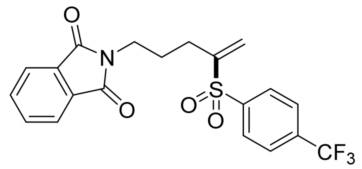

Following the Standard Procedure A, the reaction of sodium 4-(trifluoromethyl)benzenesulfinate **1f** (34.8 mg, 0.15 mmol, 1.5 equiv) and (*E*)-5-(5-(1,3-dioxo-isoindolin-2-yl)pent-1-en-1-yl)-5*H*-thianthren-5-ium tetrafluoroborate **2a** (51.7 mg, 0.10 mmol, 1.0 equiv) for 24 h afforded product after flash chromatography EA: PE = 0-20% as white solid **4e** (25.9 mg, 61% yield).

^{1}H NMR (600 MHz, $CDCl_3$) δ 7.99 (d, *J* = 8.1 Hz, 2H), 7.82 (dd, *J* = 5.4, 3.1 Hz, 2H), 7.78 (d, *J* = 8.1 Hz, 2H), 7.72 (dd, *J* = 5.5, 3.0 Hz, 2H), 6.47 (s, 1H), 5.92 (s, 1H), 3.65 (t, *J* = 7.1 Hz, 2H), 2.28 (t, *J* = 7.7 Hz, 2H), 1.88 (p, *J* = 7.4 Hz, 2H);

^{13}C NMR (151 MHz, $CDCl_3$) δ 168.3, 149.0, 142.5, 135.4 (q, *J* = 33.2 Hz), 134.3, 132.0, 129.0, 126.6 (q, *J* = 3.8 Hz), 125.1, 123.4, 123.2 (q, *J* = 273.2 Hz), 37.0, 26.8, 26.5;

^{19}F NMR (565 MHz, $CDCl_3$) δ -63.17;

HRMS-ESI (m/z) $[M+H]^+$ calc'd for $C_{20}H_{17}F_3NO_4S^+$, 424.0825, found 424.0821.

2-(4-((4-Fluorophenyl)sulfonyl)pent-4-en-1-yl)isoindoline-1,3-dione (4f)

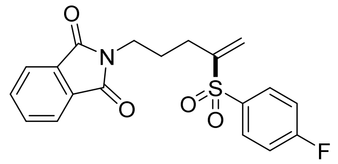

Following the Standard Procedure A, the reaction of sodium 4-fluorobenzenesulfinate **1g** (27.3 mg, 0.15 mmol, 1.5 equiv) and (*E*)-5-(5-(1,3-dioxoisoindolin-2-yl)pent-1-en-1-yl)-5*H*-thianthren-5-ium tetrafluoroborate **2a** (51.7 mg, 0.10 mmol, 1.0 equiv) for 10 h afforded product after flash chromatography EA: PE = 0-20% as white solid **4f** (30.6 mg, 82% yield).

^{1}H NMR (400 MHz, $CDCl_3$) δ 7.88 – 7.79 (m, 4H), 7.71 (dd, *J* = 5.4, 3.1 Hz, 2H), 7.21 – 7.13 (m, 2H), 6.39 (s, 1H), 5.83 (s, 1H), 3.63 (t, *J* = 7.1 Hz, 2H), 2.28 (t, *J* = 7.8 Hz, 2H), 1.92 – 1.81 (m, 2H);

^{13}C NMR (101 MHz, $CDCl_3$) δ 168.3, 165.9 (d, *J* = 256.4 Hz), 149.4, 134.8 (d, *J* = 3.2 Hz), 134.2, 132.0, 131.2 (d, *J* = 9.5 Hz), 123.9, 123.4, 116.7 (d, *J* = 22.6 Hz), 37.0, 26.6, 26.5;

^{19}F NMR (376 MHz, $CDCl_3$) δ -103.53;

HRMS-ESI (m/z) $[M+H]^+$ calc'd for $C_{19}H_{17}FNO_4S^+$, 374.0857, found 374.0854.

2-(4-((4-Chlorophenyl)sulfonyl)pent-4-en-1-yl)isoindoline-1,3-dione (4g)

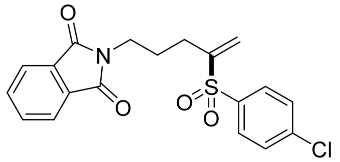

Following the Standard Procedure A, the reaction of sodium 4-chlorobenzenesulfinate **1h** (29.8 mg, 0.15 mmol, 1.5 equiv) and (*E*)-5-(5-(1,3-dioxoisoindolin-2-yl) pent-1-en-1-yl)-5*H*-thianthren-5-ium tetrafluoroborate **2a** (51.7 mg, 0.10 mmol, 1.0 equiv) for 10 h afforded product after flash chromatography EA: PE = 0-20% as white solid **4g** (33.6 mg, 86% yield).

^{1}H NMR (400 MHz, $CDCl_3$) δ 7.83 (dd, *J* = 5.5, 3.1 Hz, 2H), 7.80 – 7.76 (m, 2H), 7.72 (dd, *J* = 5.5, 3.0 Hz, 2H), 7.51 – 7.46 (m, 2H), 6.41 (s, 1H), 5.86 (s, 1H), 3.64 (t, *J* = 7.1 Hz, 2H), 2.28 (t, *J* = 7.7 Hz, 2H), 1.87 (p, *J* = 7.4 Hz, 2H);

^{13}C NMR (101 MHz, $CDCl_3$) δ 168.3, 149.3, 140.5, 137.3, 134.2, 132.0, 129.9, 129.7, 124.2, 123.4, 37.0, 26.7, 26.5;

HRMS-ESI (m/z) $[M+H]^+$ calc'd for $C_{19}H_{17}ClNO_4S^+$, 390.0561, found 390.0558.

2-(4-((4-Bromophenyl)sulfonyl)pent-4-en-1-yl)isoindoline-1,3-dione (4h)

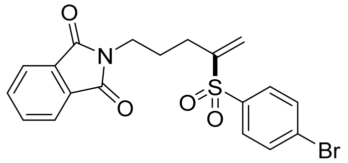

Following the Standard Procedure A, the reaction of sodium 4-bromobenzenesulfinate **1i** (36.5 mg, 0.15 mmol, 1.5 equiv) and (*E*)-5-(5-(1,3-dioxoisoindolin-2-yl) pent-1-en-1-yl)-5*H*-thianthren-5-ium tetrafluoroborate **2a** (51.7 mg, 0.10 mmol, 1.0 equiv) for 24 h afforded product after flash chromatography EA: PE = 0-20% as white solid **4h** (34.0 mg, 78% yield).

^{1}H NMR (400 MHz, $CDCl_3$) δ 7.84 (dd, *J* = 5.4, 3.0 Hz, 2H), 7.75 – 7.69 (m, 4H), 7.68 – 7.63 (m, 2H), 6.42 (s, 1H), 5.86 (s, 1H), 3.65 (t, *J* = 7.1 Hz, 2H), 2.28 (t, *J* = 7.7 Hz, 2H), 1.88 (tt, *J* = 7.7, 7.1 Hz, 2H);

^{13}C NMR (101 MHz, $CDCl_3$) δ 168.3, 149.2, 137.8, 134.2, 132.7, 132.0, 129.9, 129.1, 124.2, 123.4, 37.0, 26.6, 26.5;

HRMS-ESI (m/z) $[M+H]^+$ calc'd for $C_{19}H_{17}BrNO_4S^+$, 434.0056, found 434.0049.

2-(4-((4-Iodophenyl)sulfonyl)pent-4-en-1-yl)isoindoline-1,3-dione (4i)

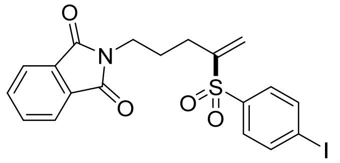

Following the Standard Procedure A, the reaction of sodium 4-iodobenzenesulfinate **1j** (43.5 mg, 0.15 mmol, 1.5 equiv) and (*E*)-5-(5-(1,3-dioxoisoindolin-2-yl)pent-1 -en-1-yl)-5*H*-thianthren-5-ium tetrafluoroborate **2a** (51.7

mg, 0.10 mmol, 1.0 equiv) for 24 h afforded product after flash chromatography EA: PE = 0-20% as white solid **4i** (38.2 mg, 79% yield).

^{1}H NMR (400 MHz, $CDCl_3$) δ 7.89 – 7.85 (m, 2H), 7.83 (dd, *J* = 5.4, 3.1 Hz, 2H), 7.73 (dd, *J* = 5.5, 3.1 Hz, 2H), 7.57 – 7.53 (m, 2H), 6.41 (s, 1H), 5.85 (s, 1H), 3.64 (t, *J* = 7.1 Hz, 2H), 2.28 (t, *J* = 7.7 Hz, 2H), 1.87 (tt, *J* = 7.7, 7.1 Hz, 2H);

^{13}C NMR (101 MHz, $CDCl_3$) δ 168.3, 149.3, 138.7, 138.5, 134.3, 132.1, 129.8, 124.2, 123.5, 101.7, 37.0, 26.7, 26.5;

HRMS-ESI (m/z) $[M+H]^+$ calc'd for $C_{19}H_{17}INO_4S^+$, 481.9917, found 481.9912.

2-(4-((3,5-Difluorophenyl)sulfonyl)pent-4-en-1-yl)isoindoline-1,3-dione (4j)

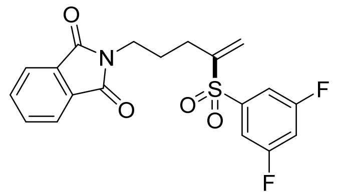

Following the Standard Procedure A, the reaction of sodium 3,5-difluorobenzenesulfinate **1k** (30.0 mg, 0.15 mmol, 1.5 equiv) and (*E*)-5-(5-(1,3-dioxoisoindolin-2-yl) pent-1-en-1-yl)-5*H*-thianthren-5-ium tetrafluoroborate **2a** (51.7 mg, 0.10 mmol, 1.0 equiv) for 24 h afforded product after flash chromatography EA: PE = 0-20% as white solid **4j** (29.6 mg, 76% yield).

^{1}H NMR (600 MHz, $CDCl_3$) δ 7.83 (dd, *J* = 5.5, 3.1 Hz, 2H), 7.72 (dd, *J* = 5.5, 3.1 Hz, 2H), 7.43 – 7.35 (m, 2H), 7.09 – 7.02 (m, 1H), 6.46 (s, 1H), 5.94 (s, 1H), 3.66 (t, *J* = 7.1 Hz, 2H), 2.30 (t, *J* = 7.7 Hz, 2H), 1.90 (tt, *J* = 7.7, 7.1 Hz, 2H);

^{13}C NMR (151 MHz, $CDCl_3$) δ 168.4, 163.0 (dd, *J* = 255.8, 11.4 Hz), 148.7, 142.4 (t, *J* = 8.0 Hz), 134.3, 132.0, 125.4, 123.5, 111.9 (dd, *J* = 21.7, 6.4 Hz), 109.5 (t, *J* = 25.0 Hz), 37.0, 26.7, 26.5;

^{19}F NMR (376 MHz, $CDCl_3$) δ -104.85;

HRMS-ESI (m/z) $[M+H]^+$ calc'd for $C_{19}H_{16}F_2NO_4S^+$, 392.0763, found 392.0760.

2-(4-((2-Fluorophenyl)sulfonyl)pent-4-en-1-yl)isoindoline-1,3-dione (4k)

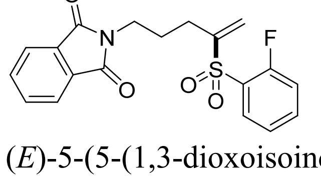

Following the Standard Procedure A, the reaction of sodium 2-fluorobenzenesulfinate **1l** (27.3 mg, 0.15 mmol, 1.5 equiv) and (*E*)-5-(5-(1,3-dioxoisoindolin-2-yl)pent-1-en-1-yl)-5*H*-thianthren-5-ium tetra -fluoroborate **2a** (51.7 mg, 0.10 mmol, 1.0 equiv) for 10 h afforded product after flash chromatography EA: PE = 0-20% as white solid **4k** (22.4 mg, 60% yield).

^{1}H NMR (400 MHz, $CDCl_3$) δ 7.95 (t, *J* = 7.7 Hz, 1H), 7.86 – 7.79 (m, 2H), 7.76 – 7.69 (m, 2H), 7.65 – 7.58 (m, 1H), 7.30 (t, *J* = 7.7 Hz, 1H), 7.17 (t, *J* = 7.7 Hz, 1H),

6.50 (s, 1H), 5.96 (s, 1H), 3.63 (t, *J* = 7.1 Hz, 2H), 2.31 (t, *J* = 7.7 Hz, 2H), 1.88 (tt, *J* = 7.7, 7.1 Hz, 2H);

^{13}C NMR (101 MHz, $CDCl_3$) δ 168.3, 159.7 (d, *J* = 258.1 Hz), 148.8, 136.4 (d, *J* = 8.5 Hz), 134.2, 132.1, 131.1, 126.7 (d, *J* = 13.8 Hz), 125.5 (d, *J* = 1.9 Hz), 124.8 (d, *J* = 3.9 Hz), 123.4, 117.5 (d, *J* = 21.1 Hz), 37.0, 26.7, 26.6;

^{19}F NMR (376 MHz, $CDCl_3$) δ -107.33;

HRMS-ESI (m/z) $[M+H]^+$ calc'd for $C_{19}H_{17}FNO_4S^+$, 374.0857, found 374.0855.

2-(4-(Naphthalen-2-ylsulfonyl)pent-4-en-1-yl)isoindoline-1,3-dione (4l)

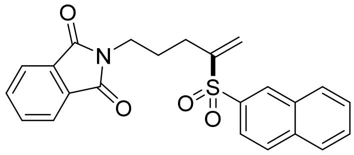

Following the Standard Procedure A, the reaction of sodium naphthalene-2-sulfinate **1m** (32.1 mg, 0.15 mmol, 1.5 equiv) and (*E*)-5-(5-(1,3-dioxoisoindolin-2-yl)pent-1-en-1-yl)-5*H*-thianthren-5-ium tetrafluoroborate **2a** (51.7 mg, 0.10 mmol, 1.0 equiv) for 10 h afforded product after flash chromatography EA: PE = 0-20% as white solid **4l** (32.1 mg, 79% yield).

^{1}H NMR (400 MHz, $CDCl_3$) δ 8.47 (d, *J* = 1.9 Hz, 1H), 7.96 (dd, *J* = 11.3, 8.3 Hz, 2H), 7.90 (d, *J* = 8.0 Hz, 1H), 7.79 – 7.74 (m, 3H), 7.71 – 7.59 (m, 4H), 6.47 (s, 1H), 5.87 (s, 1H), 3.61 (t, *J* = 7.1 Hz, 2H), 2.33 (t, *J* = 7.7 Hz, 2H), 1.88 (tt, *J* = 7.7, 7.1 Hz, 2H);

^{13}C NMR (101 MHz, $CDCl_3$) δ 168.3, 149.5, 135.5, 135.4, 134.1, 132.3, 132.0, 130.3, 129.7, 129.6, 129.4, 128.1, 127.8, 123.9, 123.4, 123.0, 37.0, 26.7, 26.5;

HRMS-ESI (m/z) $[M+H]^+$ calc'd for $C_{23}H_{20}NO_4S^+$, 406.1108, found 406.1103.

2-(4-(Thiophen-2-ylsulfonyl)pent-4-en-1-yl)isoindoline-1,3-dione (4m)

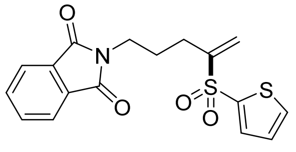

Following the Standard Procedure A, the reaction of sodium thiophene-2-sulfinate **1n** (25.5 mg, 0.15 mmol, 1.5 equiv) and (*E*)-5-(5-(1,3-dioxoisoindolin-2-yl)pent-1-en-1-yl)-5*H*-thianthren-5-ium tetrafluoroborate **2a** (51.7 mg, 0.10 mmol, 1.0 equiv) for 10 h afforded product after flash chromatography EA: PE = 0-20% as white solid **4m** (32.1 mg, 89% yield).

^{1}H NMR (400 MHz, $CDCl_3$) δ 7.83 (dd, *J* = 5.6, 2.2 Hz, 2H), 7.72 (dd, *J* = 5.6, 2.2 Hz, 2H), 7.68 (dd, *J* = 4.9, 1.3 Hz, 1H), 7.65 (p, *J* = 1.9 Hz, 1H), 7.11 (dd, *J* = 4.9, 3.6 Hz, 1H), 6.40 (d, *J* = 2.9 Hz, 1H), 5.84 – 5.76 (m, 1H), 3.66 (t, *J* = 7.2 Hz, 2H), 2.41 (t, *J* = 7.8 Hz, 2H), 1.94 – 1.83 (m, 2H);

^{13}C NMR (101 MHz, $CDCl_3$) δ 168.4, 150.1, 140.0, 134.7, 134.5, 134.2, 132.1, 128.1, 123.5, 123.4, 37.1, 26.8, 26.7;

HRMS-ESI (m/z) $[M+H]^+$ calc'd for $C_{17}H_{16}NO_4S_2^+$, 362.0515, found 362.0511.

(*E*)-2-(4-(Styrylsulfonyl)pent-4-en-1-yl)isoindoline-1,3-dione (4n)

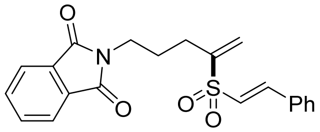

Following the Standard Procedure A, the reaction of sodium (*E*)-2-phenylethene-1-sulfinate **1o** (28.5 mg, 0.15 mmol, 1.5 equiv) and (*E*)-5-(5-(1,3-dioxoisoindolin-2-yl) pent-1-en-1-yl)-5*H*-thianthren-5-ium tetrafluoroborate **2a** (51.7 mg, 0.10 mmol, 1.0 equiv) for 10 h afforded product after flash chromatography EA: PE = 0-20% as white solid **4n** (32.1 mg, 84% yield).

^{1}H NMR (400 MHz, $CDCl_3$) δ 7.78 (dd, *J* = 5.4, 3.1 Hz, 2H), 7.69 (dd, *J* = 5.5, 3.0 Hz, 2H), 7.58 (d, *J* = 15.5 Hz, 1H), 7.53 – 7.48 (m, 2H), 7.45 – 7.37 (m, 3H), 6.72 (d, *J* = 15.5 Hz, 1H), 6.35 (s, 1H), 5.86 (s, 1H), 3.72 (t, *J* = 7.1 Hz, 2H), 2.51 – 2.40 (m, 2H), 2.08 – 1.95 (m, 2H);

^{13}C NMR (101 MHz, $CDCl_3$) δ 168.4, 149.0, 145.0, 134.1, 132.5, 132.1, 131.5, 129.2, 128.8, 124.7, 124.3, 123.4, 37.1, 26.7, 26.6;

HRMS-ESI (m/z) $[M+H]^+$ calc'd for $C_{21}H_{20}NO_4S^+$, 382.1108, found 382.1103.

2-(4-(Allylsulfonyl)pent-4-en-1-yl)isoindoline-1,3-dione (4o)

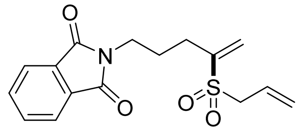

Following the Standard Procedure A, the reaction of sodium prop-2-ene-1-sulfinate **1p** (19.2 mg, 0.15 mmol, 1.5 equiv) and (*E*)-5-(5-(1,3-dioxoisoindolin-2-yl)pent-1-en-1-yl)-5*H* -thianthren-5-ium tetrafluoroborate **2a** (51.7 mg, 0.10 mmol, 1.0 equiv) for 10 h afforded product after flash chromatography EA: PE = 0-20% as white solid **4o** (20.7 mg, 65% yield).

^{1}H NMR (600 MHz, $CDCl_3$) δ 7.85 (dd, *J* = 5.5, 2.9 Hz, 2H), 7.73 (dd, *J* = 5.5, 2.9 Hz, 2H), 6.23 (s, 1H), 5.93 (s, 1H), 5.81 (ddd, *J* = 17.1, 10.2, 7.4 Hz, 1H), 5.40 (d, *J* = 10.2 Hz, 1H), 5.35 (d, *J* = 17.1 Hz, 1H), 3.75 (t, *J* = 7.2 Hz, 2H), 3.71 (d, *J* = 7.4 Hz, 2H), 2.46 (t, *J* = 7.7 Hz, 2H), 2.00 (tt, *J* = 7.7, 7.2 Hz, 2H);

^{13}C NMR (151 MHz, $CDCl_3$) δ 168.4, 147.2, 134.2, 132.1, 126.5, 124.8, 124.6, 123.5, 57.7, 37.1, 27.5, 26.7;

HRMS-ESI (m/z) $[M+H]^+$ calc'd for $C_{16}H_{18}NO_4S^+$, 320.0951, found 320.0949.

2-(4-((((1*R*,4*S*)-7,7-Dimethyl-2-oxobicyclo[2.2.1]heptan-1-yl)methyl)sulfonyl)pent-4-en-1-yl)isoindoline-1,3-dione (4p)

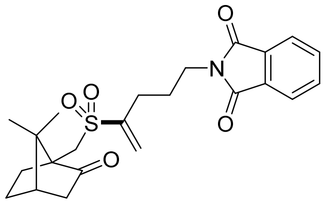

Following the Standard Procedure A, the reaction of sodium ((1*R*,4*S*)-7,7-dimethyl-2-oxobicyclo[2.2.1]heptan-1-yl)methanesulfinate **1q** (35.7 mg, 0.15 mmol, 1.5 equiv) and (*E*)-5-(5-(1,3-dioxoisoindolin-2-yl)pent-1-en-1-yl)-5*H*-thianthren-5-ium tetrafluoroborate **2a** (51.7 mg, 0.10 mmol, 1.0 equiv) for 10 h afforded product after flash chromatography EA: PE = 0-20% as white solid **4p** (30.1 mg, 70% yield).

^{1}H NMR (400 MHz, $CDCl_3$) δ 7.84 (dt, *J* = 6.4, 3.2 Hz, 2H), 7.72 (dd, *J* = 5.4, 3.0 Hz, 2H), 6.27 (s, 1H), 5.86 (s, 1H), 3.80 (t, *J* = 7.1 Hz, 2H), 3.47 (d, *J* = 14.8 Hz, 1H), 2.77 (d, *J* = 14.8 Hz, 1H), 2.62 (q, *J* = 7.9 Hz, 2H), 2.58 – 2.48 (m, 1H), 2.32 (dt, *J* = 18.5, 3.9 Hz, 1H), 2.13 – 1.98 (m, 4H), 1.89 (d, *J* = 18.5 Hz, 1H), 1.67 (ddd, *J* = 14.0, 9.3, 4.6 Hz, 1H), 1.42 (ddd, *J* = 13.1, 9.4, 3.9 Hz, 1H), 1.10 (s, 3H), 0.86 (s, 3H);

^{13}C NMR (101 MHz, $CDCl_3$) δ 214.8, 168.5, 150.2, 134.1, 132.2, 124.6, 123.4, 58.8, 49.5, 48.5, 42.7, 42.6, 37.2, 27.3, 27.2, 26.7, 24.5, 20.0, 19.9;

HRMS-ESI (m/z) $[M+H]^+$ calc'd for $C_{23}H_{28}NO_5S^+$, 430.1683, found 430.1680.

2-(4-(Cyclopropylsulfonyl)pent-4-en-1-yl)isoindoline-1,3-dione (4q)

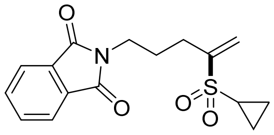

Following the Standard Procedure A, the reaction of sodium cyclopropanesulfinate **1r** (19.2 mg, 0.15 mmol, 1.5 equiv) and (*E*)-5-(5-(1,3-dioxoisoindolin-2-yl)pent-1-en-1-yl)-5*H*-thianthren-5-ium tetrafluoroborate **2a** (51.7 mg, 0.10 mmol, 1.0 equiv) for 10 h afforded product after flash chromatography EA: PE = 0-20% as white solid **4q** (28.6 mg, 90% yield).

^{1}H NMR (400 MHz, $CDCl_3$) δ 7.86 – 7.81 (m, 2H), 7.75 – 7.68 (m, 2H), 6.21 (s, 1H), 5.79 (s, 1H), 3.76 (t, *J* = 7.1 Hz, 2H), 2.59 – 2.49 (m, 2H), 2.36 (ddd, *J* = 8.0, 4.8, 1.4 Hz, 1H), 2.09 – 1.94 (m, 2H), 1.24 – 1.19 (m, 2H), 1.09 – 0.99 (m, 2H);

^{13}C NMR (101 MHz, $CDCl_3$) δ 168.4, 149.1, 134.2, 132.1, 123.6, 123.4, 37.2, 29.6, 27.3, 26.7, 5.6;

HRMS-ESI (m/z) $[M+H]^+$ calc'd for $C_{16}H_{18}NO_4S^+$, 320.0951, found 320.0949.

1-((1-Phenylvinyl)sulfonyl)naphthalene (4r)

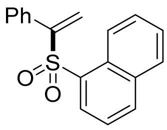

Following the Standard Procedure A, the reaction of sodium naphthalene-1-sulfinate **1s** (32.1 mg, 0.15 mmol, 1.5 equiv) and (*E*)-5-styryl-5*H*-thianthren-5-ium tetrafluoroborate **2a** (40.6 mg, 0.10 mmol, 1.0 equiv) for 10 h afforded product after flash chromatography EA: PE = 0-20% as white solid **4r** (22.2 mg, 75% yield).

^{1}H NMR (400 MHz, $CDCl_3$) δ 8.60 (d, *J* = 8.6 Hz, 1H), 8.13 (dd, *J* = 7.3, 1.3 Hz, 1H), 8.01 (dd, *J* = 8.3, 1.3 Hz, 1H), 7.91 – 7.85 (m, 1H), 7.64 (ddt, *J* = 8.5, 6.9, 1.3 Hz, 1H), 7.57 (tt, *J* = 7.0, 1.2 Hz, 1H), 7.42 (t, *J* = 7.8 Hz, 1H), 7.22 (ddt, *J* = 8.6, 6.0, 3.1 Hz, 1H), 7.17 – 7.09 (m, 4H), 6.77 (s, 1H), 6.05 (s, 1H);

^{13}C NMR (101 MHz, $CDCl_3$) δ 151.6, 135.3, 134.0, 133.0, 132.5, 131.7, 129.3, 129.2, 128.9, 128.8, 128.4, 128.2, 127.0, 125.9, 124.4, 124.3;

HRMS-ESI (m/z) $[M+H]^+$ calc'd for $C_{18}H_{15}O_2S^+$, 295.0787, found 295.0784.

1,3,5-Trimethyl-2-((1-phenylvinyl)sulfonyl)benzene (4s)

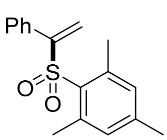

Following the Standard Procedure B, the reaction of sodium 2,4,6-trimethylbenzenesulfinate **1t** (30.9 mg, 0.15 mmol, 1.5 equiv) and (*E*)-5-styryl-5*H*-thianthren-5-ium tetrafluoroborate **2a** (40.6 mg, 0.10 mmol, 1.0 equiv) for 10 h at 50 ºC afforded product after flash chromatography EA: PE = 0-20% as white solid **4s** (24.5 mg, 86% yield)

^{1}H NMR (600 MHz, $CDCl_3$) δ 7.34 – 7.29 (m, 1H), 7.26 – 7.19 (m, 4H), 6.84 (s, 2H), 6.44 (s, 1H), 5.88 (s, 1H), 2.45 (s, 6H), 2.25 (s, 3H);

^{13}C NMR (151 MHz, $CDCl_3$) δ 152.0, 143.5, 140.8, 132.8, 132.1, 131.6, 129.3, 129.2, 128.3, 123.9, 22.7, 21.1;

HRMS-ESI (m/z) $[M+H]^+$ calc'd for $C_{17}H_{19}O_2S^+$, 287.1100, found 287.1096.

2-Methylene-4-phenylbutanenitrile (5a)

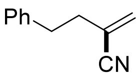

Following the Standard Procedure C, the reaction of $Zn(CN)_2$ **1u** (17.6 mg, 0.15 mmol, 1.5 equiv), KF (17.4 mg, 0.30 mmol, 3.0 equiv) and (*E*)-5-(4-phenylbut-1-en-1-yl)-5*H*-thianthren-5-ium tetrafluoroborate **2a** (43.4 mg, 0.10 mmol, 1.0 equiv) for 10 h afforded product after flash chromatography EA: PE = 0-5% as colorless oil **5a** (11.0 mg, 70% yield).

^{1}H NMR (400 MHz, $CDCl_3$) δ 7.31 (dd, *J* = 8.1, 6.6 Hz, 2H), 7.25 – 7.21 (m, 1H),

7.21 – 7.17 (m, 2H), 5.83 (s, 1H), 5.64 (s, 1H), 2.89 (t, J = 7.3 Hz, 2H), 2.57 (t, J = 7.3 Hz, 2H);

^{13}C NMR (101 MHz, $CDCl_3$) δ 139.7, 131.1, 128.7, 128.6, 126.6, 122.5, 118.7, 36.6, 34.0;

HRMS-ESI (m/z) $[M+H]^+$ calc'd for $C_{11}H_{12}N^+$, 158.0964, found 158.0965.

2-Cyclohexylacrylonitrile (5b)

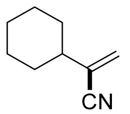

Following the Standard Procedure C, the reaction of $Zn(CN)_2$ **1u** (17.6 mg, 0.15 mmol, 1.5 equiv), KF (17.4 mg, 0.30 mmol, 3.0 equiv) and (*E*)-5-(2-cyclohexylvinyl)-5*H*-thianthren-5-ium tetrafluoroborate **2l** (41.2 mg, 0.10 mmol, 1.0 equiv) for 10 h afforded product after flash chromatography EA: PE = 0-5% as colorless oil **5b** (7.1 mg, 53% yield).

^{1}H NMR (400 MHz, $CDCl_3$) δ 5.79 (s, 1H), 5.68 (s, 1H), 2.20 – 2.10 (m, 1H), 1.90 – 1.78 (m, 4H), 1.74 – 1.65 (m, 1H), 1.37 – 1.13 (m, 5H);

^{13}C NMR (101 MHz, $CDCl_3$) δ 129.2, 128.1, 118.6, 42.7, 31.4, 25.9, 25.7;

HRMS-ESI (m/z) $[M+H]^+$ calc'd for $C_9H_{14}N^+$, 136.1121, found 136.1125.

5-(1,3-Dioxoisoindolin-2-yl)-2-methylenepentanenitrile (5c)

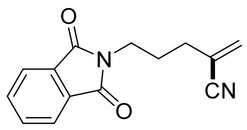

Following the Standard Procedure C, the reaction of $Zn(CN)_2$ **1u** (17.6 mg, 0.15 mmol, 1.5 equiv), KF (17.4 mg, 0.30 mmol, 3.0 equiv) and (*E*)-5-(5-(1,3-dioxoisoindolin-2-yl)pent-1-en-1-yl) -5*H*-thianthren-5-ium tetrafluoroborate **2c** (51.7 mg, 0.10 mmol, 1.0 equiv) for 10 h afforded product after flash chromatography EA: PE = 0-10% as colorless oil **5c** (18.4 mg, 77% yield).

^{1}H NMR (400 MHz, $CDCl_3$) δ 7.85 (dd, J = 5.4, 3.1 Hz, 2H), 7.73 (dd, J = 5.5, 3.0 Hz, 2H), 5.89 (s, 1H), 5.80 (s, 1H), 3.73 (t, J = 6.8 Hz, 2H), 2.32 (t, J = 7.7 Hz, 2H), 1.96 (tt, J = 7.7, 6.8 Hz, 2H);

^{13}C NMR (101 MHz, $CDCl_3$) δ 168.4, 134.2, 132.1, 131.3, 123.5, 121.9, 118.4, 36.8, 32.0, 26.7;

HRMS-ESI (m/z) $[M+H]^+$ calc'd for $C_{14}H_{13}N_2O_2^+$, 241.0972, found 241.0970.

9-Cyanodec-9-en-1-yl benzoate (5d)

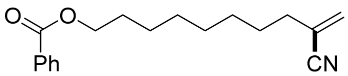

Following the Standard Procedure C, the reaction of $Zn(CN)_2$ **1u** (17.6 mg, 0.15 mmol, 1.5 equiv), KF (17.4 mg, 0.30 mmol, 3.0 equiv) and (*E*)-5-(10-(benzoyloxy)dec-1-en-1-yl)-5*H*-thianthren-5-ium tetrafluoroborate **2g** (56.2 mg, 0.10 mmol, 1.0 equiv) for 10 h afforded product after flash chromatography EA: PE = 0-10% as colorless oil **5d** (18.7 mg, 66% yield).

^{1}H NMR (400 MHz, $CDCl_3$) δ 8.07 – 8.02 (m, 2H), 7.58 – 7.52 (m, 1H), 7.47 – 7.41 (m, 2H), 5.82 (s, 1H), 5.69 (s, 1H), 4.31 (t, *J* = 6.6 Hz, 2H), 2.24 (t, *J* = 7.6 Hz, 2H), 1.77 (tt, *J* = 6.9, 6.6 Hz, 2H), 1.56 (tt, *J* = 7.6, 6.5 Hz, 2H), 1.49 – 1.29 (m, 8H);

^{13}C NMR (101 MHz, $CDCl_3$) δ 166.8, 133.0, 130.6, 130.2, 129.7, 128.5, 123.5, 118.9, 65.2, 34.7, 29.2, 28.8, 28.6, 27.6, 26.1;

HRMS-ESI (m/z) $[M+H]^+$ calc'd for $C_{18}H_{24}NO_2^+$, 286.1802, found 286.1801.

10-Hydroxy-2-methylenedecanenitrile (5e)

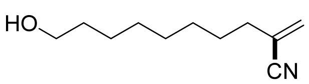

Following the Standard Procedure C, the reaction of $Zn(CN)_2$ **1u** (17.6 mg, 0.15 mmol, 1.5 equiv), KF (17.4 mg, 0.30 mmol, 3.0 equiv) and (*E*)-5-(10-hydroxydec-1-en-1-yl)-5*H*-thianthren-5-ium tetrafluoroborate **2j** (45.8 mg, 0.10 mmol, 1.0 equiv) for 10 h afforded product after flash chromatography EA: PE = 0-10% as colorless oil **5e** (11.8 mg, 65% yield).

^{1}H NMR (400 MHz, $CDCl_3$) δ 5.83 (s, 1H), 5.69 (s, 1H), 3.64 (t, *J* = 6.6 Hz, 2H), 2.24 (t, *J* = 7.6 Hz, 2H), 1.60 – 1.52 (m, 4H), 1.38 – 1.29 (m, 8H);

^{13}C NMR (101 MHz, $CDCl_3$) δ 130.3, 123.5, 118.9, 63.1, 34.7, 32.9, 29.4, 29.3, 28.6, 27.6, 25.8;

HRMS-ESI (m/z) $[M+H]^+$ calc'd for $C_{11}H_{20}NO^+$, 182.1539, found 182.1539.

7-Bromo-2-methyleneheptanenitrile (5f)

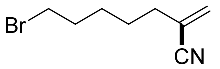

Following the Standard Procedure C, the reaction of $Zn(CN)_2$ **1u** (17.6 mg, 0.15 mmol, 1.5 equiv), KF (17.4 mg, 0.30 mmol, 3.0 equiv) and (*E*)-5-(7-bromohept-1-en-1-yl)-5*H*-thianthren-5-ium tetrafluoroborate **2d** (47.9 mg, 0.10 mmol, 1.0 equiv) for 10 h afforded product after flash chromatography EA: PE = 0-5% as colorless oil **5f** (16.9 mg, 84% yield).

^{1}H NMR (400 MHz, $CDCl_3$) δ 5.85 (s, 1H), 5.72 (s, 1H), 3.41 (t, *J* = 6.8 Hz, 2H), 2.28 (t, *J* = 7.5 Hz, 2H), 1.89 (tt, *J* = 7.4, 6.8 Hz, 2H), 1.60 (tt, *J* = 7.5, 7.0 Hz, 2H),

1.49 (tt, $J = 7.0, 6.8$ Hz, 2H);

^{13}C NMR (101 MHz, $CDCl_3$) δ 130.6, 123.1, 118.7, 34.6, 33.5, 32.4, 27.3, 26.9;

HRMS-ESI (m/z) $[M+H]^+$ calc'd for $C_8H_{13}BrN^+$, 202.0226, found 202.0223.

2-Methyleneoct-7-enenitrile (5g)

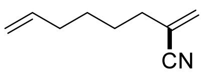

Following the Standard Procedure C, the reaction of $Zn(CN)_2$ **1u** (17.6 mg, 0.15 mmol, 1.5 equiv), KF (17.4 mg, 0.30 mmol, 3.0 equiv) and (*E*)-5-(octa-1,7-dien-1-yl)-5*H*-thianthren-5-ium tetrafluoroborate **2k** (41.2 mg, 0.10 mmol, 1.0 equiv) for 10 h afforded product after flash chromatography EA: PE = 0-5% as colorless oil **5g** (6.4 mg, 47% yield).

^{1}H NMR (400 MHz, $CDCl_3$) δ 5.83 (s, 1H), 5.79 (ddt, $J = 17.2, 10.2, 7.2$ Hz, 1H), 5.70 (s, 1H), 5.01 (dd, $J = 17.2, 1.9$ Hz, 1H), 4.97 (dd, $J = 10.2, 1.9$ Hz, 1H), 2.26 (t, $J = 7.5$ Hz, 2H), 2.08 (dt, $J = 7.2, 7.0$ Hz, 2H), 1.63 – 1.54 (m, 2H), 1.48 – 1.39 (m, 2H);

^{13}C NMR (101 MHz, $CDCl_3$) δ 138.4, 130.3, 123.4, 118.9, 115.0, 34.6, 33.4, 28.0, 27.1;

HRMS-ESI (m/z) $[M+H]^+$ calc'd for $C_9H_{14}N^+$, 136.1121, found 136.1122.

1,2,3,4,4a,5,8,8a-Octahydro-1,4-methanonaphthalene-6-carbonitrile (5h)

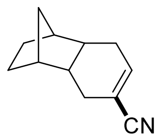

Following the Standard Procedure D, the reaction of $Zn(CN)_2$ **1u** (17.6 mg, 0.15 mmol, 1.5 equiv), KF (17.4 mg, 0.30 mmol, 3.0 equiv) and 5-(1,2,3,4,4a,5,8,8a-octahydro-1,4-methanonaphthalen-6-yl)-5*H*-thianthren-5-ium tetrafluoroborate **2v** (45.0 mg, 0.10 mmol, 1.0 equiv) at 50 °C for 10 h afforded product after flash chromatography EA: PE = 0-5% as colorless oil **5h** (13.2 mg, 76% yield).

^{1}H NMR (400 MHz, $CDCl_3$) δ 6.85 – 6.77 (m, 1H), 2.49 – 2.37 (m, 2H), 2.00 (s, 2H), 1.77 – 1.67 (m, 2H), 1.62 – 1.53 (m, 5H), 1.28 – 1.19 (m, 2H), 1.09 (d, $J = 10.3$ Hz, 1H);

^{13}C NMR (151 MHz, $CDCl_3$) δ 147.2, 119.6, 113.7, 43.2, 43.0, 42.7, 42.2, 33.4, 30.0, 29.7, 29.6, 29.0;

HRMS-ESI (m/z) $[M+H]^+$ calc'd for $C_{12}H_{16}N^+$, 174.1277, found 174.1278.

(*Z*)-2-Propylhex-2-enenitrile (5i)

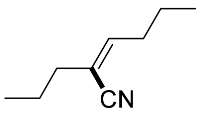

Following the Standard Procedure D, the reaction of $Zn(CN)_2$ **1u** (17.6 mg, 0.15 mmol, 1.5 equiv), KF (17.4 mg, 0.30 mmol, 3.0 equiv) and (*Z*)-5-(oct-4-en-4-yl)-5*H*-thianthren-5-ium tetrafluoroborate **2z** (41.4 mg, 0.10 mmol, 1.0 equiv) at 50 °C for 10 h afforded product after flash chromatography EA: PE = 0-5% as colorless oil **5i** (6.3 mg, 46% yield, Z/E = 1.6:1).

^{1}H NMR (400 MHz, $CDCl_3$) δ 6.35 (t, *J* = 7.6 Hz, 0.43H), 6.13 (t, *J* = 7.7 Hz, 0.57H), 2.33 (q, *J* = 7.5 Hz, 1.1H), 2.21 – 2.11 (m, 2.9H), 1.63 – 1.53 (m, 1.9H), 1.47 (dq, *J* = 14.7, 7.4 Hz, 2.1H), 1.00 – 0.88 (m, 6.0H);

^{13}C NMR (101 MHz, $CDCl_3$) δ 147.8, 118.0, 114.8, 36.3, 30.5, 22.1, 21.4, 13.7, 13.2;

HRMS-ESI (m/z) $[M+H]^+$ calc'd for $C_9H_{16}N^+$, 138.1277, found 138.1277.

2-(2,5-Dimethylphenyl)acrylonitrile (5j)

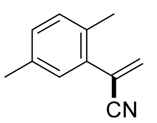

Following the Standard Procedure D, the reaction of $Zn(CN)_2$ **1u** (17.6 mg, 0.15 mmol, 1.5 equiv), KF (17.4 mg, 0.30 mmol, 3.0 equiv) and (*E*)-5-(2,5-dimethylstyryl)-5*H*-thianthren-5-ium tetrafluoroborate **2s** (43.4 mg, 0.10 mmol, 1.0 equiv) for 24 h at 50 °C afforded product after flash chromatography EA: PE = 0-5% as colorless oil **5j** (11.0 mg, 70% yield).

^{1}H NMR (600 MHz, $CDCl_3$) δ 7.11 (s, 1H), 7.11 (s, 1H), 7.07 (s, 1H), 6.23 (d, *J* = 0.5 Hz, 1H), 5.98 (d, *J* = 0.5 Hz, 1H), 2.40 (s, 3H), 2.33 (s, 3H);

^{13}C NMR (151 MHz, $CDCl_3$) δ 136.2, 133.6, 132.8, 131.0, 130.4, 129.7, 123.5, 118.2, 20.9, 19.7;

HRMS-ESI (m/z) $[M+H]^+$ calc'd for $C_{11}H_{12}N^+$, 158.0964, found 158.0965.

1-(1-(4-Fluorophenyl)vinyl)-1*H*-indole (6a)

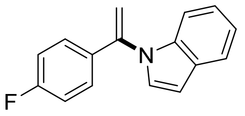

Following the Standard Procedure E, the reaction of indole **1v** (17.6 mg, 0.15 mmol, 1.5 equiv), K_2CO_3 (13.8 mg, 0.10 mmol, 1.0 equiv) and (*E*)-5-(4-fluorostyryl)-5*H*-thianthren-5-ium tetrafluoroborate **2q** (42.4 mg, 0.10 mmol, 1.0 equiv) for 10 h afforded product after flash chromatography EA: PE = 0-10% as light yellow solid **6a** (19.4 mg, 82% yield).

^{1}H NMR (400 MHz, $CDCl_3$) δ 7.65 – 7.59 (m, 1H), 7.25 (dt, *J* = 8.5, 6.1 Hz, 2H), 7.13 (d, *J* = 3.3 Hz, 1H), 7.12 – 7.05 (m, 3H), 7.00 (t, *J* = 8.6 Hz, 2H), 6.59 (d, *J* = 3.3 Hz, 1H), 5.49 (s, 1H), 5.32 (s, 1H);

^{13}C NMR (101 MHz, $CDCl_3$) δ 163.4 (d, *J* = 249.1 Hz), 144.2, 136.4, 133.3 (d, *J* =

3.2 Hz), 129.5, 128.9 (d, *J* = 8.3 Hz), 128.7, 122.2, 121.2, 120.4, 115.8 (d, *J* = 21.8 Hz), 112.0, 108.0 (d, *J* = 1.4 Hz), 103.4;

^{19}F NMR (376 MHz, $CDCl_3$) δ -112.05;

HRMS-ESI (m/z) $[M+H]^+$ calc'd for $C_{16}H_{13}FN^+$, 238.1027, found 238.1025.

1-(1-(1-(4-Fluorophenyl)vinyl)-1*H*-indol-3-yl)ethan-1-one (6b)

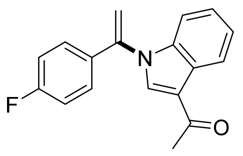

Following the Standard Procedure E, the reaction of 3-acetylindole **1w** (23.9 mg, 0.15 mmol, 1.5 equiv), K_2CO_3 (13.8 mg, 0.10 mmol, 1.0 equiv) and (*E*)-5-(4-fluorostyryl)-5*H*-thianthren-5-ium tetrafluoroborate **2q** (42.4 mg, 0.10 mmol, 1.0 equiv) for 10 h afforded product after flash chromatography EA: PE = 0-20% as light yellow solid **6b** (22.3 mg, 80% yield).

^{1}H NMR (400 MHz, $CDCl_3$) δ 8.37 (dt, *J* = 8.3, 1.0 Hz, 1H), 7.76 (s, 1H), 7.25 – 7.19 (m, 3H), 7.14 (ddd, *J* = 8.3, 7.0, 1.3 Hz, 1H), 7.06 – 6.93 (m, 3H), 5.67 (s, 1H), 5.42 (s, 1H), 2.50 (s, 3H);

^{13}C NMR (101 MHz, $CDCl_3$) δ 193.5, 163.7 (d, *J* = 250.3 Hz), 143.4, 137.3, 135.1, 132.0 (d, *J* = 3.4 Hz), 128.6 (d, *J* = 8.4 Hz), 126.6, 123.9, 123.2, 122.9, 118.6, 116.1 (d, *J* = 22.0 Hz), 112.0, 110.6 (d, *J* = 1.5 Hz), 27.9;

^{19}F NMR (376 MHz, $CDCl_3$) δ -110.89;

HRMS-ESI (m/z) $[M+H]^+$ calc'd for $C_{18}H_{15}FNO^+$, 280.1132, found 280.1130.

1-(1-(4-Fluorophenyl)vinyl)-1*H*-indole-5-carbaldehyde (6c)

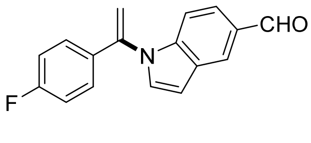

Following the Standard Procedure E, the reaction of indole-5-carboxaldehyde **1x** (21.8 mg, 0.15 mmol, 1.5 equiv), K_2CO_3 (13.8 mg, 0.10 mmol, 1.0 equiv) and (*E*)-5-(4-fluorostyryl)-5*H*-thianthren-5-ium tetrafluoroborate **2q** (42.4 mg, 0.10 mmol, 1.0 equiv) for 10 h afforded product after flash chromatography EA: PE = 0-20% as light yellow solid **6c** (21.6 mg, 81% yield).

^{1}H NMR (400 MHz, $CDCl_3$) δ 10.03 (s, 1H), 8.18 (s, 1H), 7.67 (dd, *J* = 8.7, 1.6 Hz, 1H), 7.24 (dd, *J* = 9.1, 4.5 Hz, 3H), 7.18 (d, *J* = 8.7 Hz, 1H), 7.07 – 7.01 (m, 2H), 6.77 (d, *J* = 4.5 Hz, 1H), 5.65 (s, 1H), 5.41 (s, 1H);

^{13}C NMR (101 MHz, $CDCl_3$) δ 192.4, 163.6 (d, *J* = 249.8 Hz), 143.6, 139.7, 132.6 (d, *J* = 3.3 Hz), 130.6, 130.2, 129.2, 128.7 (d, *J* = 8.4 Hz), 126.2, 122.7, 116.0 (d, *J* =

21.8 Hz), 112.3, 109.6 (d, J = 1.5 Hz), 105.1;

^{19}F NMR (376 MHz, $CDCl_3$) δ -111.24;

HRMS-ESI (m/z) $[M+H]^+$ calc'd for $C_{17}H_{13}FNO^+$, 266.0976, found 266.0975.

5-Bromo-1-(1-(4-fluorophenyl)vinyl)-1*H*-indole (6d)

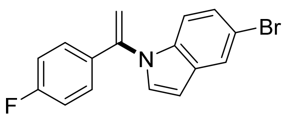

Following the Standard Procedure E, the reaction of 5-bromoindole **1y** (29.4 mg, 0.15 mmol, 1.5 equiv), K_2CO_3 (13.8 mg, 0.10 mmol, 1.0 equiv) and (*E*)-5-(4-fluorostyryl)-5*H*-thianthren-5-ium tetrafluoroborate **2q** (42.4 mg, 0.10 mmol, 1.0 equiv) for 10 h afforded product after flash chromatography EA: PE = 0-10% as light yellow solid **6d** (24.0 mg, 76% yield).

^{1}H NMR (400 MHz, $CDCl_3$) δ 7.77 (d, J = 1.9 Hz, 1H), 7.26 – 7.22 (m, 2H), 7.19 – 7.15 (m, 2H), 7.07 – 6.99 (m, 2H), 6.93 (d, J = 8.8 Hz, 1H), 6.56 (d, J = 3.3 Hz, 1H), 5.54 (s, 1H), 5.33 (s, 1H);

^{13}C NMR (101 MHz, $CDCl_3$) δ 163.5 (d, J = 249.6 Hz), 143.9, 135.1, 132.9 (d, J = 3.3 Hz), 131.1, 129.8, 128.8 (d, J = 8.3 Hz), 125.1, 123.6, 115.9 (d, J = 21.9 Hz), 113.7, 113.4, 108.5 (d, J = 1.4 Hz), 102.9;

^{19}F NMR (376 MHz, $CDCl_3$) δ -111.58;

HRMS-ESI (m/z) $[M+H]^+$ calc'd for $C_{16}H_{12}BrFN^+$, 316.0132, found 316.0129.

9-(1-(4-Fluorophenyl)vinyl)-9*H*-carbazole (6e)

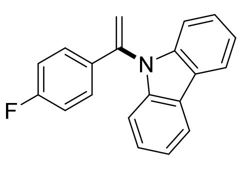

Following the Standard Procedure E, the reaction of carbazole **1z** (25.1 mg, 0.15 mmol, 1.5 equiv), K_2CO_3 (13.8 mg, 0.10 mmol, 1.0 equiv) and (*E*)-5-(4-fluorostyryl)-5*H*-thianthren-5 -ium tetrafluoroborate **2q** (42.4 mg, 0.10 mmol, 1.0 equiv) for 10 h afforded product after flash chromatography EA: PE = 0-10% as white solid **6e** (23.3 mg, 81% yield).

^{1}H NMR (400 MHz, $CDCl_3$) δ 8.14 (d, J = 7.7 Hz, 2H), 7.40 – 7.33 (m, 2H), 7.30 – 7.21 (m, 6H), 6.98 (t, J = 8.6 Hz, 2H), 6.02 (s, 1H), 5.58 (s, 1H);

^{13}C NMR (101 MHz, $CDCl_3$) δ 163.4 (d, J = 249.1 Hz), 141.9, 140.8, 132.6 (d, J = 3.3 Hz), 128.2 (d, J = 8.3 Hz), 126.0, 123.6, 120.4, 120.1, 115.9 (d, J = 21.8 Hz), 112.7 (d, J = 1.7 Hz), 110.9;

^{19}F NMR (376 MHz, $CDCl_3$) δ -112.15;

HRMS-ESI (m/z) $[M+H]^+$ calc'd for $C_{20}H_{15}FN^+$, 288.1183, found 288.1177.

1-(1-(4-Fluorophenyl)vinyl)-1*H*-imidazole (6f)

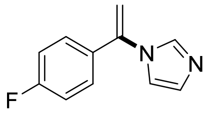

Following the Standard Procedure E, the reaction of imidazole **1aa** (10.2 mg, 0.15 mmol, 1.5 equiv), K_2CO_3 (13.8 mg, 0.10 mmol, 1.0 equiv) and (*E*)-5-(4-fluorostyryl)-5*H*-thianthren-5-ium tetrafluoroborate **2q** (42.4 mg, 0.10 mmol, 1.0 equiv) for 10 h afforded product after flash chromatography EA: PE = 0-20% as colorless oil **6f** (15.8 mg, 84% yield).

^{1}H NMR (400 MHz, $CDCl_3$) δ 7.63 (s, 1H), 7.36 – 7.28 (m, 2H), 7.14 (s, 1H), 7.12 – 7.04 (m, 2H), 7.00 (s, 1H), 5.28 (s, 1H), 5.27 (s, 1H);

^{13}C NMR (101 MHz, $CDCl_3$) δ 163.7 (d, *J* = 250.3 Hz), 142.4, 137.2, 131.9 (d, *J* = 3.4 Hz), 129.9, 129.3 (d, *J* = 8.4 Hz), 119.4, 116.0 (d, *J* = 22.0 Hz), 106.5 (d, *J* = 1.3 Hz);

^{19}F NMR (376 MHz, $CDCl_3$) δ -110.90;

HRMS-ESI (m/z) $[M+H]^+$ calc'd for $C_{11}H_{10}FN_2^+$, 189.0823, found 189.0821.

***N*-(1-(4-Fluorophenyl)vinyl)-*N*,4-dimethylbenzenesulfonamide (6g)**

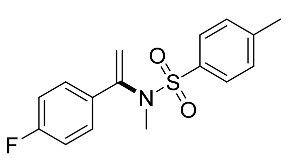

Following the Standard Procedure E, the reaction of *N*-methyl-*p*-toluenesulfonamide **1ab** (27.8 mg, 0.15 mmol, 1.5 equiv), K_2CO_3 (13.8 mg, 0.10 mmol, 1.0 equiv) and (*E*)-5-(4-fluorostyryl)-5*H*-thianthren-5-ium tetrafluoroborate **2q** (42.4 mg, 0.10 mmol, 1.0 equiv) for 10 h afforded product after flash chromatography EA: PE = 0-20% as light yellow solid **6g** (19.4 mg, 64% yield).

^{1}H NMR (400 MHz, $CDCl_3$) δ 7.74 – 7.67 (m, 2H), 7.51 – 7.44 (m, 2H), 7.35 – 7.30 (m, 2H), 7.05 – 6.98 (m, 2H), 5.35 (s, 1H), 4.77 (s, 1H), 3.04 (s, 3H), 2.45 (s, 3H);

^{13}C NMR (101 MHz, $CDCl_3$) δ 163.3 (d, *J* = 248.3 Hz), 147.5, 143.9, 134.5, 133.8 (d, *J* = 3.3 Hz), 129.6, 128.4 (d, *J* = 8.3 Hz), 128.1, 115.4 (d, *J* = 21.7 Hz), 110.8, 38.5, 21.7;

^{19}F NMR (376 MHz, $CDCl_3$) δ -112.99;

HRMS-ESI (m/z) $[M+H]^+$ calc'd for $C_{16}H_{17}FNO_2S^+$, 306.0959, found 306.0957.

2-(1-(4-Fluorophenyl)vinyl)isoindoline-1,3-dione (6h)

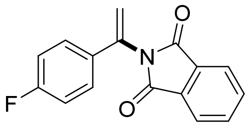

Following the Standard Procedure E, the reaction of phthalimide **1ac** (22.1 mg, 0.15 mmol, 1.5 equiv), K_2CO_3 (13.8 mg, 0.10 mmol, 1.0 equiv) and (*E*)-5-(4-fluorostyryl)-5*H*-thianthren-5-ium tetrafluoroborate **2q** (42.4 mg, 0.10 mmol, 1.0 equiv) for 10 h afforded product after flash chromatography EA: PE = 0-20% as white solid **6h** (21.5 mg, 80% yield).

^{1}H NMR (400 MHz, $CDCl_3$) δ 7.93 (dd, *J* = 5.5, 3.1 Hz, 2H), 7.79 (dd, *J* = 5.5, 3.1 Hz, 2H), 7.36 (ddt, *J* = 8.1, 5.1, 2.5 Hz, 2H), 7.06 – 6.98 (m, 2H), 5.93 (s, 1H), 5.43 (s, 1H);

^{13}C NMR (101 MHz, $CDCl_3$) δ 167.2, 163.2 (d, *J* = 248.7 Hz), 136.4, 134.6, 131.9, 131.8 (d, *J* = 3.3 Hz), 127.5 (d, *J* = 8.4 Hz), 124.0, 116.1, 115.8 (d, *J* = 21.9 Hz);

^{19}F NMR (376 MHz, $CDCl_3$) δ -112.51;

HRMS-ESI (m/z) $[M+H]^+$ calc'd for $C_{16}H_{11}FNO_2^+$, 268.0768, found 268.0768.

1-(1-(4-Fluorophenyl)vinyl)-1*H*-imidazole (6i)

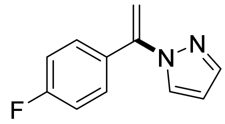

Following the Standard Procedure E, the reaction of pyrazole **1ad** (10.2 mg, 0.15 mmol, 1.5 equiv), K_2CO_3 (13.8 mg, 0.10 mmol, 1.0 equiv) and (*E*)-5-(4-fluorostyryl)-5*H*-thianthren-5-ium tetrafluoroborate **2q** (42.4 mg, 0.10 mmol, 1.0 equiv) for 10 h afforded product after flash chromatography EA: PE = 0-20% as colorless oil **6i** (12.4 mg, 66% yield).

^{1}H NMR (500 MHz, $CDCl_3$) δ 7.68 (d, *J* = 1.5 Hz, 1H), 7.50 (d, *J* = 2.4 Hz, 1H), 7.39 – 7.33 (m, 2H), 7.12 – 7.05 (m, 2H), 6.37 (dd, *J* = 2.4, 1.5 Hz, 1H), 5.55 (s, 1H), 5.16 (s, 1H).

^{13}C NMR (126 MHz, $CDCl_3$) δ 163.4 (d, *J* = 249.2 Hz), 145.1, 141.2, 132.1, 130.0 (d, *J* = 8.4 Hz), 129.7, 115.7 (d, *J* = 21.8 Hz), 106.8, 105.1.

^{19}F NMR (471 MHz, $CDCl_3$) δ -111.82;

HRMS-ESI (m/z) $[M+H]^+$ calc'd for $C_{11}H_{10}FN_2^+$, 189.0823, found 189.0822.

1-(1-(4-Fluorophenyl)vinyl)-1H-1,2,4-triazole (6j)

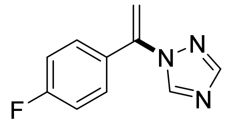

Following the Standard Procedure E, the reaction of 1,2,4-triazole **1ae** (10.4 mg, 0.15 mmol, 1.5 equiv), K_2CO_3 (13.8 mg, 0.10 mmol, 1.0 equiv) and (*E*)-5-(4-fluorostyryl)-5*H*-thianthren-5-ium tetrafluoroborate **2q** (42.4 mg, 0.10 mmol, 1.0 equiv) for 10 h afforded product after

flash chromatography EA: PE = 0-20% as colorless oil **6j** (13.3 mg, 70% yield).

^{1}H NMR (500 MHz, $CDCl_3$) δ 8.18 (s, 1H), 8.07 (s, 1H), 7.35 (dd, *J* = 8.1, 5.6 Hz, 2H), 7.12 (dd, *J* = 8.5, 8.1 Hz, 2H), 5.67 (s, 1H), 5.35 (s, 1H);

^{13}C NMR (126 MHz, $CDCl_3$) δ163.7 (d, *J* = 250.5 Hz), 152.6, 143.3, 142.0, 130.6, 129.8 (d, *J* = 8.5 Hz), 116.1 (d, *J* = 22.0 Hz), 107.9;

^{19}F NMR (471 MHz, $CDCl_3$) δ -110.46;

HRMS-ESI (m/z) $[M+H]^+$ calc'd for $C_{10}H_9FN_3^+$, 190.0775, found. 190.0776.

Following the Standard Procedure E, the reaction of 1,2,3-triazole **1af** (10.4 mg, 0.15 mmol, 1.5 equiv), K_2CO_3 (13.8 mg, 0.10 mmol, 1.0 equiv) and (*E*)-5-(4-fluorostyryl)-5*H*-thianthren-5-ium tetrafluoroborate **2q** (42.4 mg, 0.10 mmol, 1.0 equiv) for 10 h afforded product after flash chromatography EA: PE = 0-30% as colorless oil **6k** (6.8 mg, 36% yield) and colorless oil **6k'** (5.3 mg, 28% yield).

2-(1-(4-Fluorophenyl)vinyl)-2*H*-1,2,3-triazole (6k)

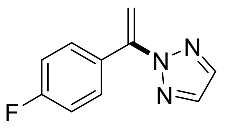

^{1}H NMR (500 MHz, $CDCl_3$) δ 7.78 (s, 2H), 7.46 – 7.35 (m, 2H), 7.16 – 7.06 (m, 2H), 5.90 (s, 1H), 5.34 (s, 1H);

^{13}C NMR (126 MHz, $CDCl_3$) δ 163.5 (d, *J* = 249.3 Hz), 145.2, 135.6, 131.0, 130.3 (d, *J* = 8.4 Hz), 115.6 (d, *J* = 21.9 Hz), 107.5;

^{19}F NMR (471 MHz, $CDCl_3$) δ -111.74;

HRMS-ESI (m/z) $[M+H]^+$ calc'd for $C_{10}H_9FN_3^+$, 190.0775, found 190.0775.

1-(1-(4-Fluorophenyl)vinyl)-1*H*-1,2,3-triazole (6k')

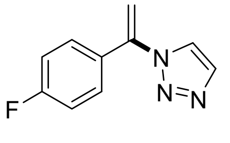

^{1}H NMR (500 MHz, $CDCl_3$) δ 7.78 (s, 1H), 7.62 (s, 1H), 7.34 – 7.28 (m, 2H), 7.09 (t, *J* = 8.6 Hz, 2H), 5.76 (s, 1H), 5.50 (s, 1H);

^{13}C NMR (126 MHz, $CDCl_3$) δ 163.7 (d, *J* = 250.6 Hz), 142.2, 133.9, 130.9, 129.4 (d, *J* = 8.5 Hz), 124.0, 116.1 (d, *J* = 22.0 Hz), 109.5;

^{19}F NMR (471 MHz, CDCl3) δ -110.55;

HRMS-ESI (m/z) $[M+H]^+$ calc'd for $C_{10}H_9FN_3^+$, 190.0775, found 190.0775.

5-Bromo-1-(1-(4-chlorophenyl)vinyl)-1*H*-indole (6l)

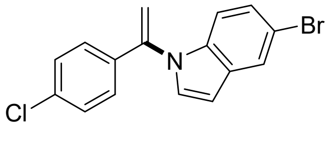

Following the Standard Procedure E, the reaction of 5-bromoindole **1y** (29.4 mg, 0.15 mmol, 1.5 equiv), K_2CO_3 (13.8 mg, 0.10 mmol, 1.0 equiv) and (*E*)-5-(4-chlorostyryl)-5*H*-thianthren-5-ium tetrafluoroborate **2p** (44.1 mg, 0.10 mmol,

1.0 equiv) for 10 h afforded product after flash chromatography EA: PE = 0-10% as light yellow solid **6l** (25.9 mg, 78% yield).

^{1}H NMR (400 MHz, $CDCl_3$) δ 7.78 (s, 1H), 7.32 (dd, *J* = 8.7, 1.8 Hz, 2H), 7.22 – 7.15 (m, 4H), 6.93 (d, *J* = 8.7 Hz, 1H), 6.57 (d, *J* = 3.3 Hz, 1H), 5.59 (s, 1H), 5.38 (s, 1H);

^{13}C NMR (101 MHz, $CDCl_3$) δ 143.8, 135.5, 135.2, 135.1, 131.1, 129.8, 129.1, 128.3, 125.2, 123.7, 113.8, 113.3, 109.2, 103.0;

HRMS-ESI (m/z) $[M+H]^+$ calc'd for $C_{16}H_{12}BrClN^+$, 331.9836, found 331.9835.

5-Bromo-1-(1-(4-bromophenyl)vinyl)-1*H*-indole (6m)

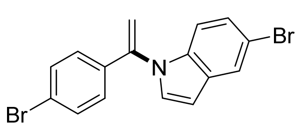

Following the Standard Procedure E, the reaction of 5-bromoindole **1y** (29.4 mg, 0.15 mmol, 1.5 equiv), K_2CO_3 (13.8 mg, 0.10 mmol, 1.0 equiv) and (*E*)-5-(4-bromostyryl) -5*H*-thianthren-5-ium tetrafluoroborate **2o** (48.5 mg, 0.10 mmol, 1.0 equiv) for 10 h afforded product after flash chromatography EA: PE = 0-10% as light yellow solid **6m** (31.0 mg, 82% yield).

^{1}H NMR (400 MHz, $CDCl_3$) δ 7.77 (t, *J* = 2.1 Hz, 1H), 7.53 – 7.43 (m, 2H), 7.25 – 7.10 (m, 4H), 6.93 (dd, *J* = 8.8, 2.1 Hz, 1H), 6.56 (t, *J* = 2.6 Hz, 1H), 5.60 (d, *J* = 1.9 Hz, 1H), 5.38 (d, *J* = 1.9 Hz, 1H);

^{13}C NMR (101 MHz, $CDCl_3$) δ 143.9, 135.6, 135.1, 132.1, 131.1, 129.8, 128.5, 125.2, 123.72, 123.67, 113.8, 113.3, 109.3, 103.1;

HRMS-ESI (m/z) $[M+H]^+$ calc'd for $C_{16}H_{12}Br_2N^+$, 375.9331, found 375.9329.

5-Bromo-1-(3,4-dihydronaphthalen-1-yl)-1*H*-indole (6n)

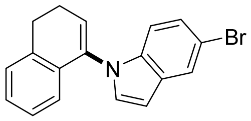

Following the Standard Procedure E, the reaction of 5-bromoindole **1y** (29.4 mg, 0.15 mmol, 1.5 equiv), K_2CO_3 (13.8 mg, 0.10 mmol, 1.0 equiv) and 5-(3,4-dihydronaphthalen -2-yl)-5*H*-thianthren-5-ium tetrafluoroborate **2aa** (43.2 mg, 0.10 mmol, 1.0 equiv) for 10 h afforded product after flash chromatography EA: PE = 0-10% as light yellow solid **6n** (26.5 mg, 82% yield).

^{1}H NMR (400 MHz, $CDCl_3$) δ 7.79 (d, *J* = 1.9 Hz, 1H), 7.25 – 7.17 (m, 4H), 7.04 (dt, *J* = 7.8, 3.0 Hz, 2H), 6.58 (d, *J* = 3.0 Hz, 1H), 6.48 (d, *J* = 7.8 Hz, 1H), 6.19 (t, *J* = 4.6 Hz, 1H), 2.97 (t, *J* = 8.1 Hz, 2H), 2.57 (td, *J* = 8.1, 4.6 Hz, 2H);

^{13}C NMR (101 MHz, $CDCl_3$) δ 136.4, 136.3, 135.6, 131.9, 130.3, 130.0, 128.4, 127.9, 126.9, 125.7, 124.8, 123.4, 123.1, 113.3, 112.8, 102.1, 27.6, 22.9;

HRMS-ESI (m/z) $[M+H]^+$ calc'd for $C_{18}H_{15}BrN^+$, 324.0382, found 324.0381.

1-(4-Phenylbut-1-en-2-yl)-1*H*-imidazole (6o)

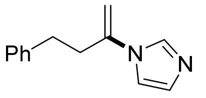

Following the Standard Procedure F, the reaction of imidazole **1aa** (10.2 mg, 0.15 mmol, 1.5 equiv), Cs_2CO_3 (32.6 mg, 0.10 mmol, 1.0 equiv) and (*E*)-5-(4-phenylbut-1-en-1-yl)-5*H*-thianthren-5-ium tetrafluoroborate **2a** (43.4 mg, 0.10 mmol, 1.0 equiv) for 10 h afforded product after flash chromatography EA: PE = 0-20% as colorless oil **6o** (11.8 mg, 60% yield).

^{1}H NMR (400 MHz, $CDCl_3$) δ 7.70 (s, 1H), 7.32 – 7.27 (m, 2H), 7.24 – 7.19 (m, 1H), 7.16 – 7.09 (m, 4H), 5.07 (s, 1H), 4.78 (s, 1H), 2.80 (s, 4H);

^{13}C NMR (101 MHz, $CDCl_3$) δ 142.1, 140.2, 135.3, 130.1, 128.7, 128.4, 126.5, 117.2, 103.9, 35.9, 33.4;

HRMS-ESI (m/z) $[M+H]^+$ calc'd for $C_{13}H_{15}N_2^+$, 199.1230, found 199.1228.

1-(1-Cyclohexylvinyl)-1*H*-imidazole (6p)

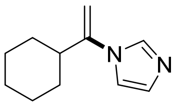

Following the Standard Procedure F, the reaction of imidazole **1aa** (10.2 mg, 0.15 mmol, 1.5 equiv), Cs_2CO_3 (32.6 mg, 0.10 mmol, 1.0 equiv) and (*E*)-5-(5-(1,3-dioxoisoindolin-2-yl)pent-1-en-1-yl)-5*H*-thianthren-5-ium tetrafluoroborate **2l** (41.2 mg, 0.10 mmol, 1.0 equiv) for 10 h afforded product after flash chromatography EA: PE = 0-20% as colorless oil **6p** (13.0 mg, 74% yield).

^{1}H NMR (600 MHz, $CDCl_3$) δ 7.61 (s, 1H), 7.08 (s, 1H), 7.04 (s, 1H), 5.05 (s, 1H), 4.85 (s, 1H), 2.32 (tt, J = 11.5, 3.2 Hz, 1H), 1.86 – 1.77 (m, 4H), 1.75 – 1.69 (m, 1H), 1.36 – 1.26 (m, 2H), 1.24 – 1.13 (m, 3H);

^{13}C NMR (151 MHz, $CDCl_3$) δ 148.8, 135.8, 129.6, 117.9, 103.8, 42.2, 31.5, 26.4, 26.2;

HRMS-ESI (m/z) $[M+H]^+$ calc'd for $C_{11}H_{17}N_2^+$, 177.1386, found 177.1385.

1-(Octa-1,7-dien-2-yl)-1*H*-imidazole (6q)

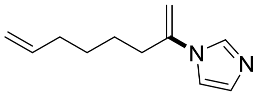

Following the Standard Procedure F, the reaction of imidazole **1aa** (10.2 mg, 0.15 mmol, 1.5 equiv), Cs_2CO_3 (32.6 mg, 0.10 mmol, 1.0 equiv) and (*E*)-5-(5-(1,3-dioxoisoindolin-2-yl)pent-1-en-1-yl)-5*H* -thianthren-5-ium tetrafluoroborate **2k** (41.2 mg, 0.10 mmol, 1.0 equiv) for 10 h

afforded product after flash chromatography EA: PE = 0-20% as colorless oil **6q** (11.5 mg, 65% yield).

^{1}H NMR (600 MHz, $CDCl_3$) δ 7.68 (s, 1H), 7.10 (s, 1H), 7.09 (s, 1H), 5.77 (ddt, *J* = 16.9, 10.2, 6.7 Hz, 1H), 5.07 (s, 1H), 4.99 (dd, *J* = 16.9, 1.8 Hz, 1H), 4.95 (dd, *J* = 10.2, 1.8 Hz, 1H), 4.80 (s, 1H), 2.50 (t, *J* = 7.4 Hz, 2H), 2.06 (dt, *J* = 7.2, 6.7 Hz, 2H), 1.51 (tt, *J* = 7.4, 7.0 Hz, 2H), 1.44 (tt, *J* = 7.2, 7.0 Hz, 2H);

^{13}C NMR (151 MHz, $CDCl_3$) δ 142.9, 138.4, 135.3, 129.9, 117.2, 115.0, 103.1, 33.9, 33.4, 28.3, 26.4;

HRMS-ESI (m/z) $[M+H]^+$ calc'd for $C_{11}H_{17}N_2^+$, 177.1386, found 177.1385.

(*E*)-1-(1-Cyclohexylpent-1-en-1-yl)-1*H*-imidazole (6r)

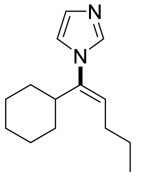

Following the Standard Procedure E, the reaction of imidazole **1aa** (10.2 mg, 0.15 mmol, 1.5 equiv), Cs_2CO_3 (32.6 mg, 0.10 mmol, 1.0 equiv) and (*E*)-5-(4-fluorostyryl)-5*H*-thianthren-5-ium tetrafluoroborate **2bb** (45.4 mg, 0.10 mmol, 1.0 equiv) for 10 h afforded product after flash chromatography EA: PE = 0-10% as colorless oil **6r** (15.9 mg, 73% yield, rr > 15:1).

^{1}H NMR (500 MHz, $CDCl_3$) δ 7.33 (s, 1H), 7.09 (s, 1H), 6.80 (s, 1H), 5.45 (t, *J* = 7.3 Hz, 1H), 2.15 (tt, *J* = 11.6, 3.2 Hz, 1H), 1.80 – 1.68 (m, 6H), 1.32 (tq, *J* = 7.4, 7.2 Hz, 2H), 1.28 – 1.17 (m, 3H), 1.15 – 1.02 (m, 3H), 0.82 (t, *J* = 7.4 Hz, 3H);

^{13}C NMR (126 MHz, $CDCl_3$) δ 141.2, 137.3, 128.9, 125.7, 119.9, 44.6, 31.4, 28.8, 26.3, 26.1, 22.8, 13.7;

HRMS-ESI (m/z) $[M+H]^+$ calc'd for $C_{14}H_{23}N_2^+$, 219.1856, found 219.1857.

1-(Cyclohex-1-en-1-yl)-1*H*-imidazole (6s)

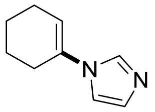

Following the Standard Procedure F, the reaction of imidazole **1aa** (10.2 mg, 0.15 mmol, 1.5 equiv), Cs_2CO_3 (32.6 mg, 0.10 mmol, 1.0 equiv) and (*E*)-5-(5-(1,3-dioxoisoindolin-2-yl)pent-1-en-1-yl)-5*H*-thianthren-5-ium tetrafluoroborate **2u** (42.4 mg, 0.10 mmol, 1.0 equiv) for 10 h afforded product after flash chromatography EA: PE = 0-20% as colorless oil **6s** (8.5 mg, 57% yield).

^{1}H NMR (400 MHz, $CDCl_3$) δ 7.65 (s, 1H), 7.08 (s, 1H), 7.06 (s, 1H), 5.83 (tt, *J* = 3.9, 1.6 Hz, 1H), 2.46 – 2.39 (m, 2H), 2.23 – 2.15 (m, 2H), 1.87 – 1.78 (m, 2H), 1.71 – 1.62 (m, 2H);

^{13}C NMR (101 MHz, $CDCl_3$) δ 134.6, 133.9, 129.5, 116.7, 116.5, 27.5, 24.2, 22.5, 21.8;

HRMS-ESI (m/z) $[M+H]^+$ calc'd for $C_9H_{13}N_2^+$, 149.1073, found 149.1073.

1-((1*E*,5*Z*)-Cycloocta-1,5-dien-1-yl)-1*H*-imidazole (6t)

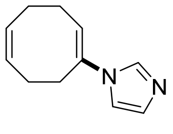

Following the Standard Procedure F, the reaction of imidazole **1aa** (10.2 mg, 0.15 mmol, 1.5 equiv), Cs_2CO_3 (32.6 mg, 0.10 mmol, 1.0 equiv) and (*E*)-5-(5-(1,3-dioxoisoindolin-2-yl)pent-1-en-1-yl)-5*H*-thianthren-5-ium tetrafluoro-borate **2w** (42.4 mg, 0.10 mmol, 1.0 equiv) for 10 h afforded product after flash chromatography EA: PE = 0-20% as colorless oil **6t** (9.9 mg, 57% yield).

^{1}H NMR (400 MHz, $CDCl_3$) δ 7.56 (s, 1H), 7.04 (s, 1H), 7.00 (s, 1H), 5.74 (t, *J* = 6.7 Hz, 1H), 5.61 (t, *J* = 4.1 Hz, 2H), 2.81 – 2.73 (m, 2H), 2.53 – 2.41 (m, 6H);

^{13}C NMR (101 MHz, $CDCl_3$) δ 136.1, 136.0, 129.3, 128.9, 127.8, 121.8, 118.1, 32.1, 27.7, 26.6, 25.7;

HRMS-ESI (m/z) $[M+H]^+$ calc'd for $C_{11}H_{15}N_2^+$, 175.1230, found 175.1229.

5-((3,5-Dimethylphenoxy)methyl)-3-(1-(4-fluorophenyl)vinyl)oxazolidin-2-one (6u)

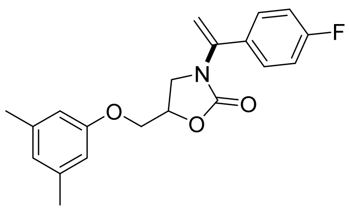

Following the Standard Procedure E, the reaction of Metaxalone (33.2 mg, 0.15 mmol, 1.5 equiv), K_2CO_3 (13.8 mg, 0.10 mmol, 1.0 equiv) and (*E*)-5-(4-fluorostyryl)-5*H*-thianthren-5-ium tetrafluoro-borate **2q** (42.4 mg, 0.10 mmol, 1.0 equiv) for 10 h afforded product after flash chromatography EA: PE = 0-50% as white solid **6u** (27.9 mg, 82% yield).

^{1}H NMR (600 MHz, $CDCl_3$) δ 7.44 – 7.39 (m, 2H), 7.09 – 7.01 (m, 2H), 6.67 (s, 1H), 6.56 (s, 2H), 5.23 (d, *J* = 0.6 Hz, 1H), 5.20 (d, *J* = 0.6 Hz, 1H), 4.90 (ddt, *J* = 9.0, 5.5, 4.7 Hz, 1H), 4.20 (dd, *J* = 10.2, 4.7 Hz, 1H), 4.14 (dd, *J* = 10.2, 4.7 Hz, 1H), 3.92 (dd, *J* = 9.1, 9.0 Hz, 1H), 3.79 (dd, *J* = 9.1, 5.5 Hz, 1H), 2.30 (s, 6H);

^{13}C NMR (151 MHz, $CDCl_3$) δ 163.2 (d, *J* = 248.4 Hz), 158.2, 155.2, 142.2, 139.7, 132.0 (d, *J* = 3.2 Hz), 128.7 (d, *J* = 8.3 Hz), 123.7, 115.6 (d, *J* = 21.8 Hz), 112.5, 106.9, 70.9, 68.1, 48.5, 21.6;

^{19}F NMR (376 MHz, $CDCl_3$) δ -112.62.

HRMS-ESI (m/z) $[M+H]^+$ calc'd for $C_{20}H_{21}FNO_3^+$, 342.1500, found 342.1498.

1-(1-(4-Fluorophenyl)vinyl)-2-(((3-methyl-4-(2,2,2-trifluoroethoxy)pyridin-2-yl)-methyl)sulfinyl)-1*H*-benzo[*d*]imidazole (6v)

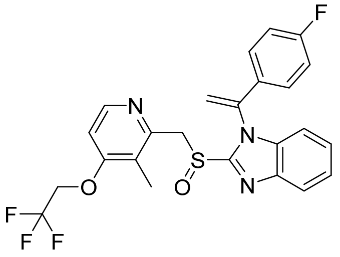

Following the Standard Procedure E, the reaction of Lansoprazole (55.4 mg, 0.15 mmol, 1.5 equiv), K_2CO_3 (13.8 mg, 0.10 mmol, 1.0 equiv) and (*E*)-5-(4-fluorostyryl)-5*H*-thianthren-5-ium tetrafluoro -borate **2q** (42.4 mg, 0.10 mmol, 1.0 equiv) for 10 h afforded product after flash chromatography EA: PE = 0-50% as white solid **6v** (34.3 mg, 70% yield).

^{1}H NMR (400 MHz, $CDCl_3$) δ 8.12 (d, *J* = 5.6 Hz, 1H), 7.88 (d, *J* = 8.1 Hz, 1H), 7.38 – 7.31 (m, 1H), 7.30 – 7.26 (m, 1H), 7.26 – 7.19 (m, 2H), 7.07 (d, *J* = 8.1 Hz, 1H), 7.03 – 6.97 (m, 1H), 6.57 (d, *J* = 5.7 Hz, 1H), 6.11 – 6.00 (m, 1H), 5.70 (s, 1H), 5.20 (d, *J* = 14.0 Hz, 1H), 4.95 (d, *J* = 14.0 Hz, 1H), 4.35 (q, *J* = 7.8 Hz, 2H), 2.33 (s, 3H);

^{13}C NMR (101 MHz, $CDCl_3$) δ 163.7 (d, *J* = 250.5 Hz), 161.8, 154.2, 152.1, 148.0, 142.2, 139.5, 136.0, 131.3 (d, *J* = 3.3 Hz), 128.2 (d, *J* = 8.5 Hz), 125.4, 124.0, 123.1, 123.0 (q, *J* = 277.9 Hz), 121.5, 116.1 (d, *J* = 22.0 Hz), 115.6, 111.9, 105.7, 65.5 (q, *J* = 36.4 Hz), 57.8, 11.1;

^{19}F NMR (376 MHz, $CDCl_3$) δ -73.79, -110.75;

HRMS-ESI (m/z) $[M+H]^+$ calc'd for $C_{24}H_{20}F_4N_3O_2S^+$, 490.1207, found 490.1206.

***N*-(2-(1-(1-(4-Fluorophenyl)vinyl)-5-methoxy-1*H*-indol-3-yl)ethyl)acetamide (6w)**

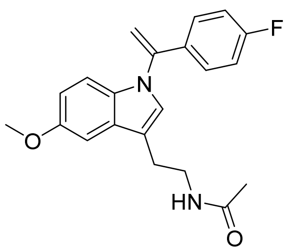

Following the Standard Procedure E, the reaction of Melatonin (34.8 mg, 0.15 mmol, 1.5 equiv), K_2CO_3 (13.8 mg, 0.10 mmol, 1.0 equiv) and (*E*)-5-(4-fluorostyryl)-5*H*-thianthren-5-ium tetrafluoroborate **2q** (42.4 mg, 0.10 mmol, 1.0 equiv) for 10 h afforded product after flash chromatography EA: PE = 0-50% as white solid **6w** (26.4 mg, 75% yield).

^{1}H NMR (400 MHz, $CDCl_3$) δ 7.28 – 7.20 (m, 2H), 7.04 – 6.97 (m, 3H), 6.95 – 6.91 (m, 2H), 6.73 (dd, *J* = 9.0, 2.4 Hz, 1H), 5.64 (bs, 1H), 5.36 (s, 1H), 5.22 (s, 1H), 3.82 (s, 3H), 3.55 (dt, *J* = 6.9, 6.2 Hz, 2H), 2.90 (t, *J* = 6.9 Hz, 2H), 1.91 (s, 3H);

^{13}C NMR (101 MHz, $CDCl_3$) δ 170.1, 163.4 (d, *J* = 249.2 Hz), 154.5, 144.1, 133.4 (d,

J = 3.2 Hz), 132.0, 129.5, 129.1 (d, J = 8.3 Hz), 126.9, 115.7 (d, J = 21.8 Hz), 113.6, 113.0, 112.4, 106.7, 100.9, 56.0, 39.8, 25.4, 23.5;

^{19}F NMR (376 MHz, $CDCl_3$) δ -111.90;

HRMS-ESI (m/z) $[M+H]^+$ calc'd for $C_{21}H_{22}FN_2O_2^+$, 353.1660, found 353.1656.

Diphenyl(4-phenylbut-1-en-2-yl)phosphine oxide (7a)

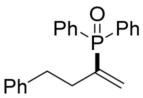

Following the Standard Procedure E, the reaction of diphenylphosphineoxide **1ag** (30.3 mg, 0.15 mmol, 1.5 equiv), K_2CO_3 (13.8 mg, 0.10 mmol, 1.0 equiv) and (*E*)-5-(4-fluorostyryl)-5*H*-thianthren-5-ium tetrafluoroborate **2a** (43.4 mg, 0.10 mmol, 1.0 equiv) for 10 h afforded product after flash chromatography EA: PE = 0-50% as white solid **7a** (22.8 mg, 69% yield).

^{1}H NMR (500 MHz, $CDCl_3$) δ 7.73 – 7.67 (m, 4H), 7.58 – 7.52 (m, 2H), 7.47 (dddd, J = 8.3, 5.5, 2.9, 1.4 Hz, 4H), 7.23 (dd, J = 8.1, 6.6 Hz, 2H), 7.18 – 7.13 (m, 1H), 7.11 – 7.06 (m, 2H), 5.97 (dd, J = 42.3, 1.1 Hz, 1H), 5.69 (dd, J = 20.7, 1.1 Hz, 1H), 2.84 – 2.75 (m, 2H), 2.67 – 2.58 (m, 2H);

^{13}C NMR (126 MHz, $CDCl_3$) δ 143.3 (d, J = 91.6 Hz), 141.1, 132.1, 132.0 (d, J = 5.7 Hz), 131.8, 131.0, 129.6 (d, J = 9.3 Hz), 128.7 (d, J = 11.9 Hz), 128.5 (d, J = 13.1 Hz), 126.2, 34.6 (d, J = 5.4 Hz), 33.5 (d, J = 10.8 Hz);

^{31}P NMR (202 MHz, $CDCl_3$) δ 31.31;

HRMS-ESI (m/z) $[M+H]^+$ calc'd for $C_{22}H_{22}PO^+$, 333.1403, found 333.1402.

3.5 Mechanistic Study

One-Pot Experiments

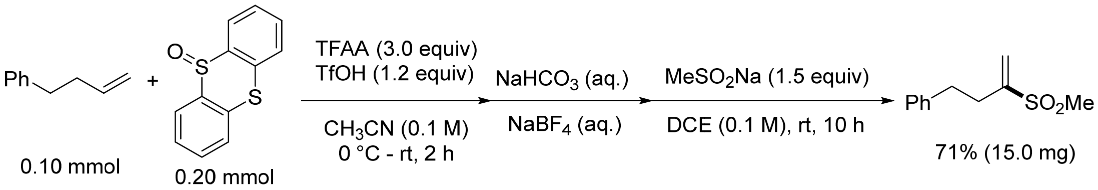

Under the nitrogen atmosphere, to a 10.0 mL borosilicate vial equipped with a magnetic stir bar was charged with thianthrene 5-oxide (46.5 mg, 0.2 mmol, 2.0 equiv), 4-phenyl-1-butene (13.2 mg, 0.1 mmol, 1.0 equiv) and CH_3CN (1.0 mL). Then, trifluoroacetic anhydride (31.5 mg, 0.30 mmol, 3.0 equiv) and

trifluoromethanesulfonic (18.0 mg, 0.12 mmol, 1.2 equiv) were added at 0 °C. The resulting mixture was stirred at 0 °C for 1 h. After stirring at room temperature for 1 h, the solvent was evaporated followed by the addition of DCM (10.0 mL), washed with saturated aqueous $NaHCO_3$ solution (2 x 10.0 mL), then the CH_2Cl_2 solution was washed with aqueous $NaBF_4$ solution (2 x 10.0 mL, 5 % w/w). The DCM solvent was evaporated followed by the addition of sodium methanesulfinate **1a** (15.6 mg, 0.15 mmol, 1.5 equiv) and DCE (1.0 mL). The resulting mixture was stirred at room temperature for 10 h. Evaporation of solvent and flash chromatography (EA: PE = 0-10%) afforded **3a** as white solid (15.0 mg, 71% yield).

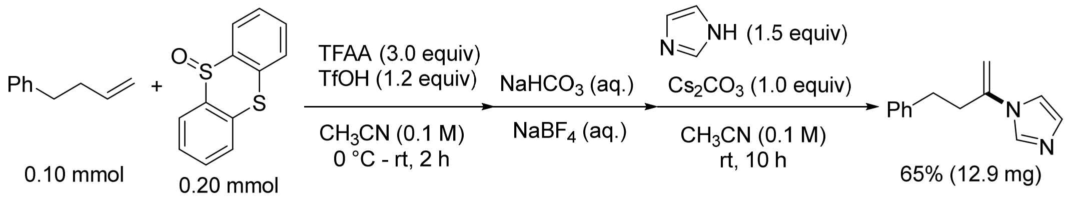

Under the nitrogen atmosphere, to a 10.0 mL borosilicate vial equipped with a magnetic stir bar was charged with thianthrene 5-oxide (46.5 mg, 0.2 mmol, 2.0 equiv), 4-phenyl- 1-butene (13.2 mg, 0.1 mmol, 1.0 equiv) and CH_3CN (1.0 mL). Then, trifluoroacetic anhydride (31.5 mg, 0.30 mmol, 3.0 equiv) and trifluoromethanesulfonic (18.0 mg, 0.12 mmol, 1.2 equiv) were added at 0 °C. The resulting mixture was stirred at 0 °C for 1 h. After stirring at room temperature for 1 h, the solvent was evaporated followed by the addition of DCM (10.0 mL), washed with saturated aqueous $NaHCO_3$ solution (2 x 10.0 mL), then the CH_2Cl_2 solution was washed with aqueous $NaBF_4$ solution (2 x 10.0 mL, 5 % w/w). The DCM solvent was evaporated followed by the addition of imidazole **1aa** (10.2 mg, 0.15 mmol, 1.5 equiv), K_2CO_3 (13.8 mg, 0.10 mmol, 1.0 equiv) and CH_3CN (1.0 mL). The resulting mixture was stirred at room temperature for 10 h. Evaporation of solvent and flash chromatography (EA: PE = 0-20%) afforded **6l** as colorless oil (12.9 mg, 65% yield).

Probe of Radical Experiments

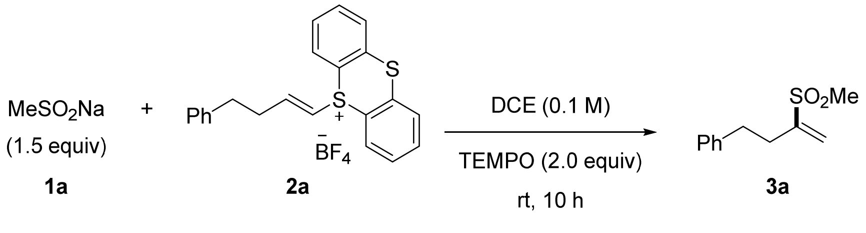

Following the Standard Procedure A, the reaction of sodium methanesulfinate **1a** (15.6 mg, 0.15 mmol, 1.5 equiv), (*E*)-5-(4-phenylbut-1-en-1-yl)-5*H*-thianthren-5-ium tetrafluoroborate **2a** (43.4 mg, 0.10 mmol, 1.0 equiv) and TEMPO (30.5 mg, 0.20 mmol, 2.0 equiv) in DCE (1.0 mL) for 10 h. The conversion of **2a** >95%, and the yield of **3a** 72% yield. Conversion and yield were determined by ^{1}H NMR of the crude mixture using mesitylene (12.0 mg, 0.10 mmol, 1.0 equiv) as internal standard.

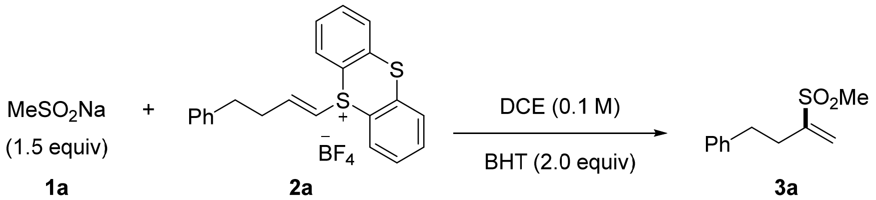

Following the Standard Procedure A, the reaction of sodium methanesulfinate **1a** (15.6 mg, 0.15 mmol, 1.5 equiv), (*E*)-5-(4-phenylbut-1-en-1-yl)-5*H*-thianthren-5-ium tetrafluoroborate **2a** (43.4 mg, 0.10 mmol, 1.0 equiv) and BHT (44.1 mg, 0.20 mmol, 2.0 equiv) in DCE (1.0 mL) for 10 h. The conversion of **2a** >95%, and the yield of **3a** 83% yield. Conversion and yield were determined by ^{1}H NMR of the crude mixture using mesitylene (12.0 mg, 0.10 mmol, 1.0 equiv) as internal standard.

Assignment of Reaction Intermediate

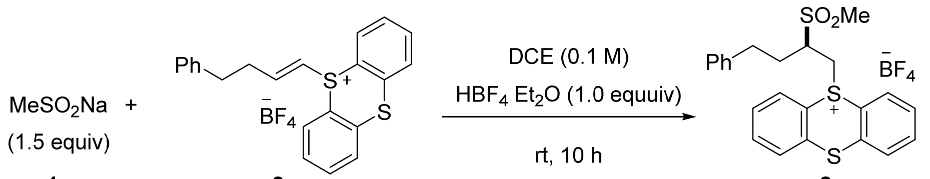

Under the nitrogen atmosphere, to a 10.0 mL Schlenk tube equipped with a magnetic stir bar was charged with (*E*)-5-(4-phenylbut-1-en-1-yl)-5*H*-thianthren-5-ium tetrafluoroborate **2a** (43.4 mg, 0.1 mmol, 1.0 equiv), sodium methanesulfinate **1a** (15.6 mg, 0.15 mmol, 1.5 equiv) and $HBF_4 \cdot Et_2O$ (16.2 mg, 0.1 mmol, 1.0 equiv). After addition of DCE (1.0 mL), the reaction stirred at room temperature for 10 h. The reaction mixture was evaporated and washed with Et_2O (3 x 10.0 mL). The residual solid was dissolved in DCM (10.0 mL) and filtrated, the filtrate was evaporated to afford **8a** (22.1 mg, 43% yield).

^{1}H NMR (400 MHz, $CDCl_3$) δ 8.33 (d, *J* = 7.8 Hz, 1H), 8.09 – 7.99 (m, 1H), 7.83 (dd, *J* = 7.9, 1.0 Hz, 1H), 7.77 – 7.69 (m, 3H), 7.69 – 7.56 (m, 2H), 7.25 – 7.15 (m, 3H), 7.08 – 7.03 (m, 2H), 4.31 (dd, *J* = 13.8, 9.6 Hz, 1H), 4.11 (dd, *J* = 13.8, 4.4 Hz, 1H),

3.25 – 3.16 (m, 1H), 3.12 (s, 3H), 2.81 (dt, *J* = 14.3, 7.2 Hz, 1H), 2.71 (dt, *J* = 14.3, 7.2 Hz, 1H), 2.44 – 2.33 (m, 1H), 2.03 (dq, *J* = 15.2, 7.2 Hz, 1H);

^{13}C NMR (101 MHz, $CDCl_3$) δ 138.9, 136.1, 135.9, 135.8, 135.2, 134.7, 130.6, 130.1, 129.0, 128.6, 126.8, 118.8, 117.3, 58.3, 40.4, 38.0, 32.0, 29.8;

^{19}F NMR (376 MHz, $CDCl_3$) δ -150.36, 150.41;

HRMS-ESI (m/z) $[M-BF_4]^+$ calc'd for $C_{23}H_{23}O_2S_3^+$, 427.0855, found 427.0853.

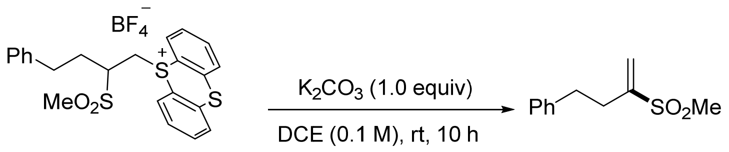

Under the nitrogen atmosphere, to a 10.0 mL Schlenk tube equipped with a magnetic stir bar was charged with 5-(2-(methylsulfonyl)-4-phenylbutyl)-5*H*-thianthren-5-ium trifluoromethanesulfonate **8a** (57.7 mg, 0.1 mmol, 1.0 equiv) and K_2CO_3 (13.8 mg, 0.1 mmol, 1.0 equiv). After addition of DCE (1.0 mL), the reaction stirred at room temperature for 10 h. The reaction mixture was purified directly by column chromatography (EA: PE = 0-20%) to afford **3a** as white solid (13.3 mg, 63% yield).

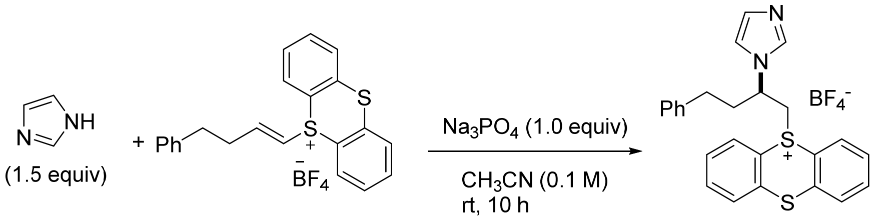

Under the nitrogen atmosphere, to a 10.0 mL Schlenk tube equipped with a magnetic stir bar was charged with (*E*)-5-(4-phenylbut-1-en-1-yl)-5*H*-thianthren-5-ium tetrafluoroborate **2a** (86.8 mg, 0.2 mmol, 1.0 equiv), imidazole **1aa** (20.4 mg, 0.3 mmol, 1.5 equiv) and Na_3PO_4 (32.8 mg, 0.2 mmol, 1.0 equiv). After addition of CH_3CN (2.0 mL), the reaction stirred at room temperature for 10 h. The reaction mixture was evaporated and washed with Et_2O (3 x 10.0 mL). The residual solid was dissolved in DCM (10.0 mL) and filtrated, the filtrate was evaporated to afford **8b** (98.3 mg, 98% yield).

^{1}H NMR (600 MHz, $CDCl_3$) δ 8.31 (d, *J* = 7.9 Hz, 1H), 7.80 (d, *J* = 7.8 Hz, 1H), 7.74 – 7.66 (m, 2H), 7.61 (dt, *J* = 10.8, 7.8 Hz, 2H), 7.51 (d, *J* = 7.9 Hz, 1H), 7.47 (s, 1H), 7.39 (t, *J* = 7.7 Hz, 1H), 7.16 (t, *J* = 7.5 Hz, 2H), 7.10 (t, *J* = 7.7 Hz, 1H), 6.98 (d, *J* =

7.5 Hz, 2H), 6.83 (s, 1H), 6.77 (s, 1H), 5.07 (tt, *J* = 6.7, 3.2 Hz, 1H), 4.68 (dd, *J* = 13.2, 3.2 Hz, 1H), 3.87 (dd, *J* = 13.2, 3.2 Hz, 1H), 2.48 – 2.41 (m, 1H), 2.25 – 2.16 (m, 2H), 1.95 (tt, *J* = 14.0, 6.7 Hz, 1H);

^{13}C NMR (101 MHz, $CDCl_3$) δ 139.6, 136.8, 136.1, 135.9, 134.6, 134.3, 133.3, 131.5, 130.41, 130.38, 130.36, 130.2, 128.6, 128.4, 126.4, 118.7, 117.1, 115.7, 54.2, 46.4, 37.1, 31.5;

^{19}F NMR (376 MHz, $CDCl_3$) δ -150.49, 150.55;

HRMS-ESI (m/z) $[M\text{-}BF_4]^+$ calc'd for $C_{25}H_{23}N_2S_2^+$, 415.1297, found 415.1290.

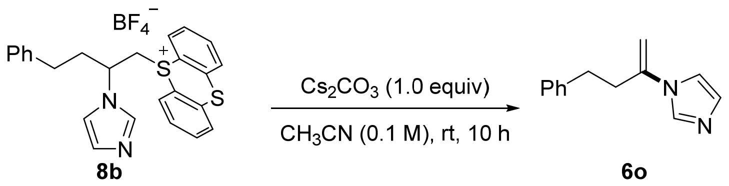

Under the nitrogen atmosphere, to a 10.0 mL Schlenk tube equipped with a magnetic stir bar was charged with 5-(2-(1*H*-imidazol-1-yl)-4-phenylbutyl)-5*H*-thianthren -5-ium tetrafluoroborate **8b** (50.2 mg, 0.1 mmol, 1.0 equiv) and Cs_2CO_3 (32.6 mg, 0.1 mmol, 1.0 equiv). After addition of CH_3CN (1.0 mL), the reaction stirred at room temperature for 10 h. The reaction mixture was purified directly by column chromatography (EA: PE = 0-20%) to afford **6o** as colorless oil (11.3 mg, 57% yield).

Deuterated Substrate Experiment

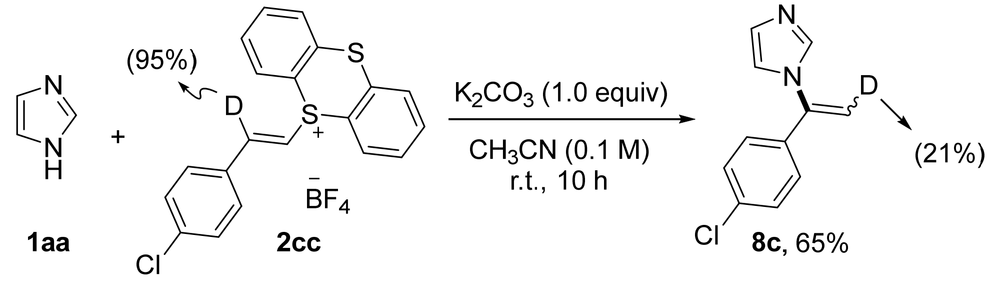

Following the Standard Procedure E, the reaction of sodium methanesulfinate **1aa** (10.2 mg, 0.15 mmol, 1.5 equiv), (*E*)-5-(2-(4-chlorophenyl)vinyl-2-*d*)-5*H*-thianthren-5-ium tetrafluoroborate **2cc** (44.2 mg, 0.10 mmol, 1.0 equiv) and K_2CO_3 (13.8 mg, 0.10 mmol, 1.0 equiv) in CH_3CN (1.0 mL) for 10 h. The reaction mixture was evaporated and afforded product after flash chromatography EA: PE = 0-10% as light-yellow oil **8c** (13.4 mg, 65% yield).

^{1}H NMR (500 MHz, $CDCl_3$) δ 7.64 (s, 1H), 7.48 – 7.33 (m, 2H), 7.29 – 7.23 (m, 2H), 7.14 (s, 1H), 7.00 (s, 1H), 5.39 – 5.26 (m, 2H).

^{13}C NMR (126 MHz, $CDCl_3$) δ 142.3, 137.2, 136.0, 134.2, 129.9, 129.2, 128.6, 119.4, 107.2.

HRMS-ESI (m/z) $[M+H]^+$ calc'd for $C_{11}H_{10}ClN_2^+$, 205.0527, found 205.0526.

‘’

3.6 Crystallographic Data

X-Ray Crystallographic Data for 4k (CCDC 2241239)

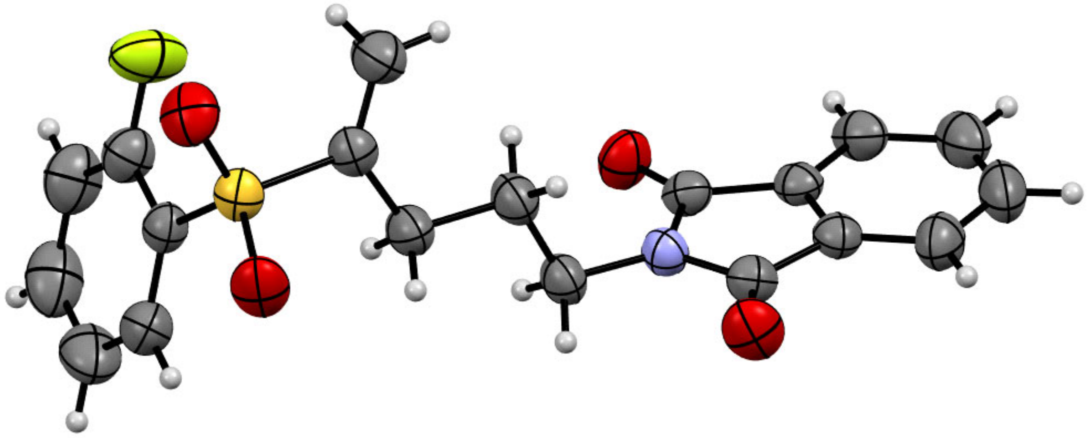

Supplementary Table 6. Crystal data and structure refinement for **4k** (LMS12209B2_0m)

Identification code	LMS12209B2_0m
Empirical formula	$C_{19}H_{16}FNO_4S$
Formula weight	373.39
Temperature/K	267.0
Crystal system	monoclinic
Space group	P2/n
a/Å	7.0846(7)
b/Å	12.2015(12)
c/Å	23.936(2)
α/°	90
β/°	98.375(4)
γ/°	90
Volume/Å^3	2047.1(3)

Z	4
ρ_{calc}g/cm^3	1.212
μ/mm^{-1}	1.676
F(000)	776.0
Crystal size/mm^3	0.02 × 0.01 × 0.01
Radiation	CuKα (λ = 1.54178)
2Θ range for data collection/°	7.244 to 144.838
Index ranges	$-8 \leq h \leq 8$, $-14 \leq k \leq 15$, $-29 \leq l \leq 22$
Reflections collected	20764
Independent reflections	4021 [R_{int} = 0.0498, R_{sigma} = 0.0344]
Data/restraints/parameters	4021/0/235
Goodness-of-fit on F^2	1.198
Final R indexes [I>=2σ (I)]	R_1 = 0.0588, wR_2 = 0.2023
Final R indexes [all data]	R_1 = 0.0629, wR_2 = 0.2059
Largest diff. peak/hole / e Å^{-3}	0.38/-0.28

X-Ray Crystallographic Data for 6g (CCDC 2241240)

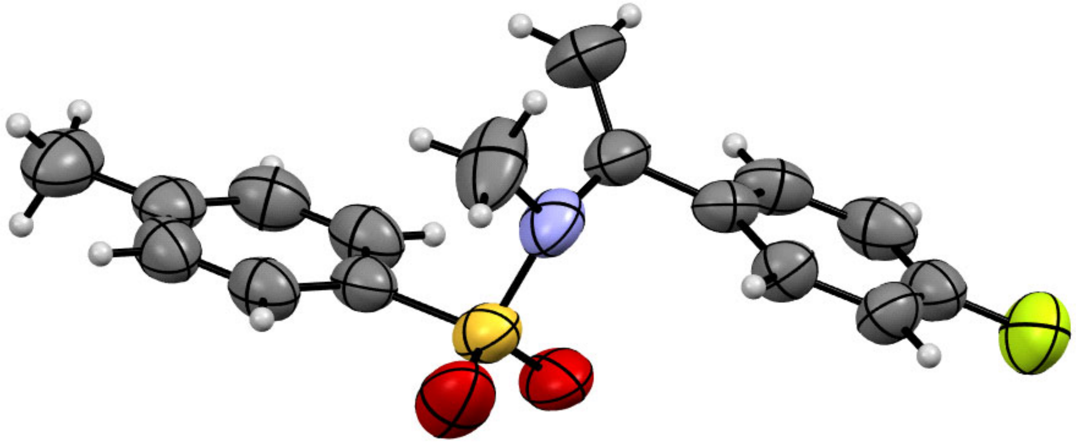

Supplementary Table 7. Crystal data and structure refinement for **6g** (lms129c06_0ma)

Identification code	lms129c06_0ma
Empirical formula	$C_{16}H_{16}FNO_2S$
Formula weight	305.36

Temperature/K	298.0
Crystal system	monoclinic
Space group	Cc
a/Å	17.2716(14)
b/Å	11.7864(14)
c/Å	7.4972(7)
α/°	90
β/°	92.385(6)
γ/°	90
Volume/Å^3	1524.9(3)
Z	4
ρ_{calc}g/cm^3	1.330
μ/mm^{-1}	2.019
F(000)	640.0
Crystal size/mm^3	0.02 × 0.01 × 0.01
Radiation	CuKα (λ = 1.54178)
2Θ range for data collection/°	9.086 to 144.248
Index ranges	-21 ≤ h ≤ 21, -14 ≤ k ≤ 14, -9 ≤ l ≤ 8
Reflections collected	12375
Independent reflections	2802 [R_{int} = 0.0543, R_{sigma} = 0.0408]
Data/restraints/parameters	2802/2/192
Goodness-of-fit on F^2	1.055
Final R indexes [I>=2σ (I)]	R_1 = 0.0349, wR_2 = 0.0890
Final R indexes [all data]	R_1 = 0.0375, wR_2 = 0.0913
Largest diff. peak/hole / e Å^{-3}	0.20/-0.13
Flack parameter	0.116(9)

3.7 Copies of ^{1}H, ^{13}C and ^{19}F NMR Spectra

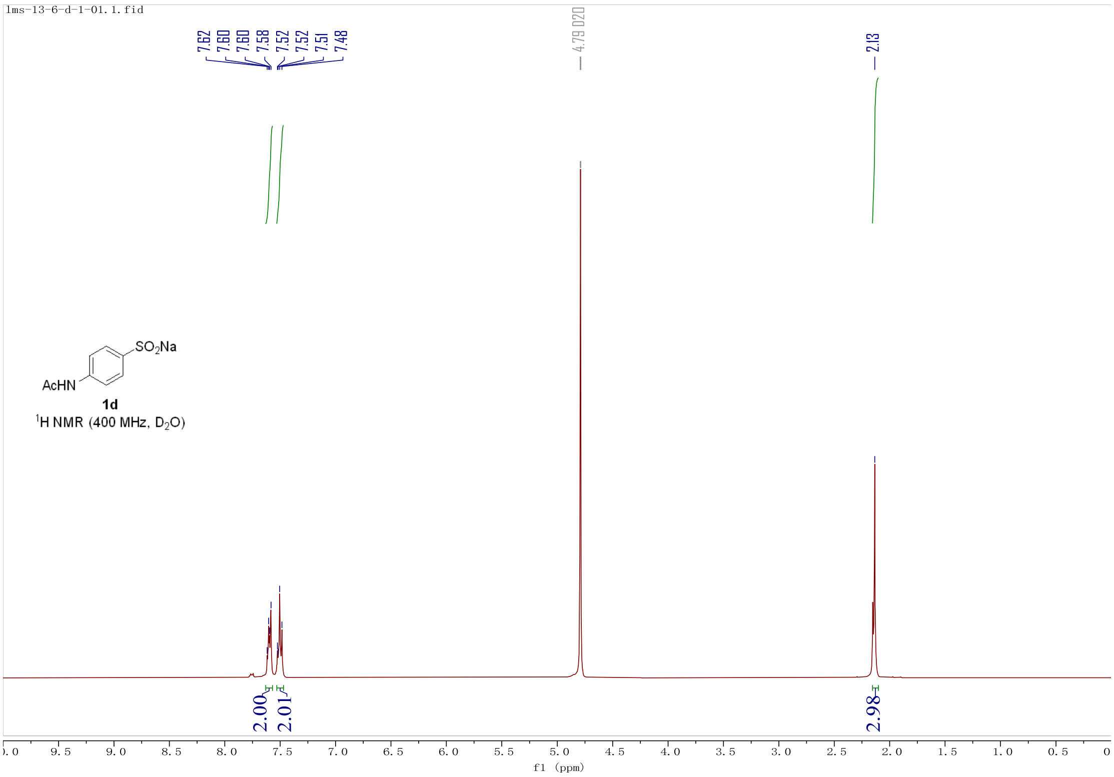

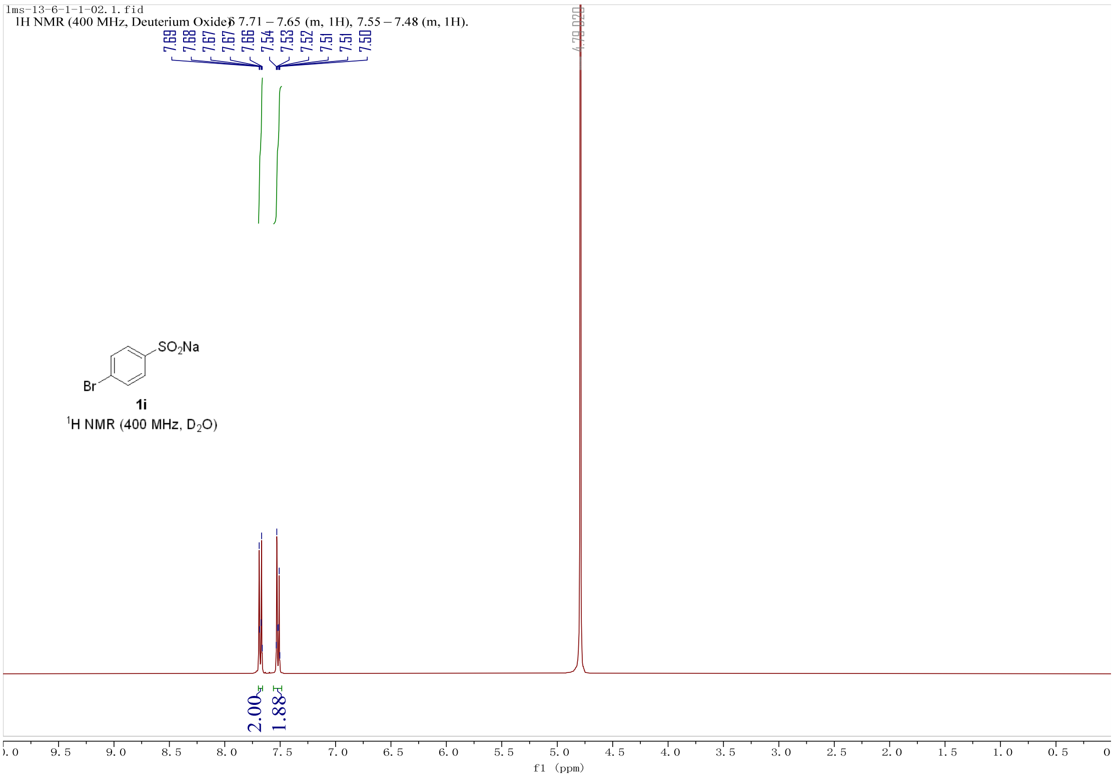

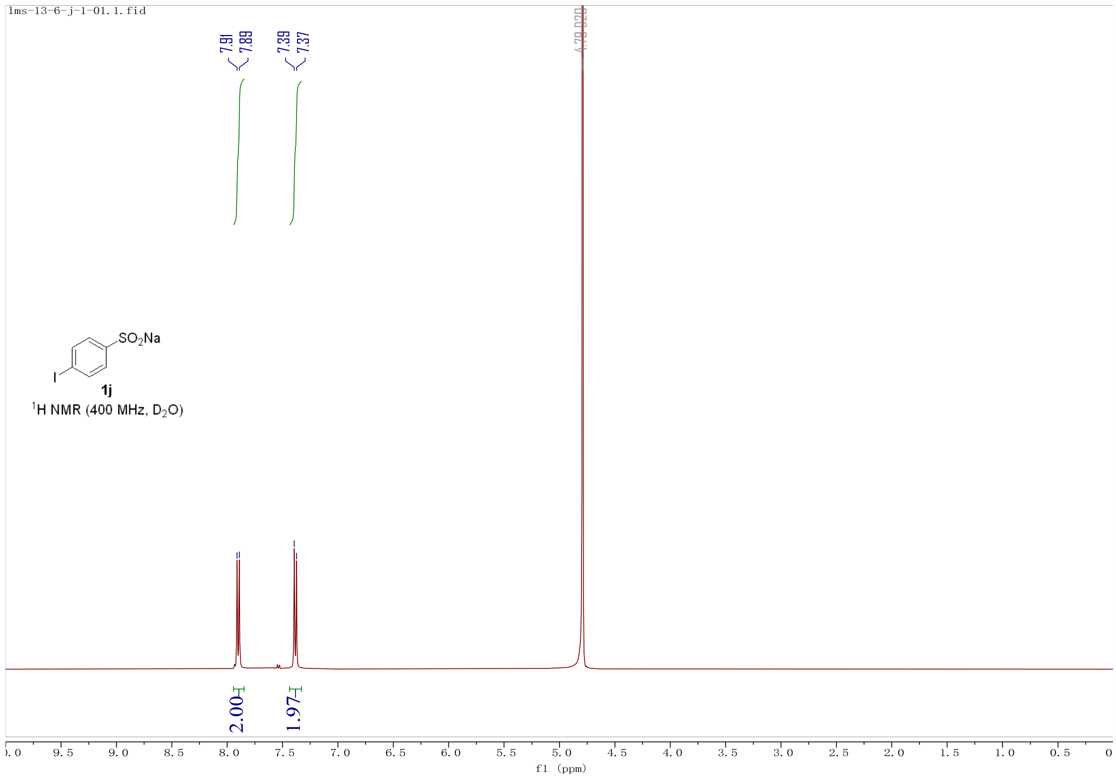

1j

^{1}H NMR (400 MHz, D_2O)

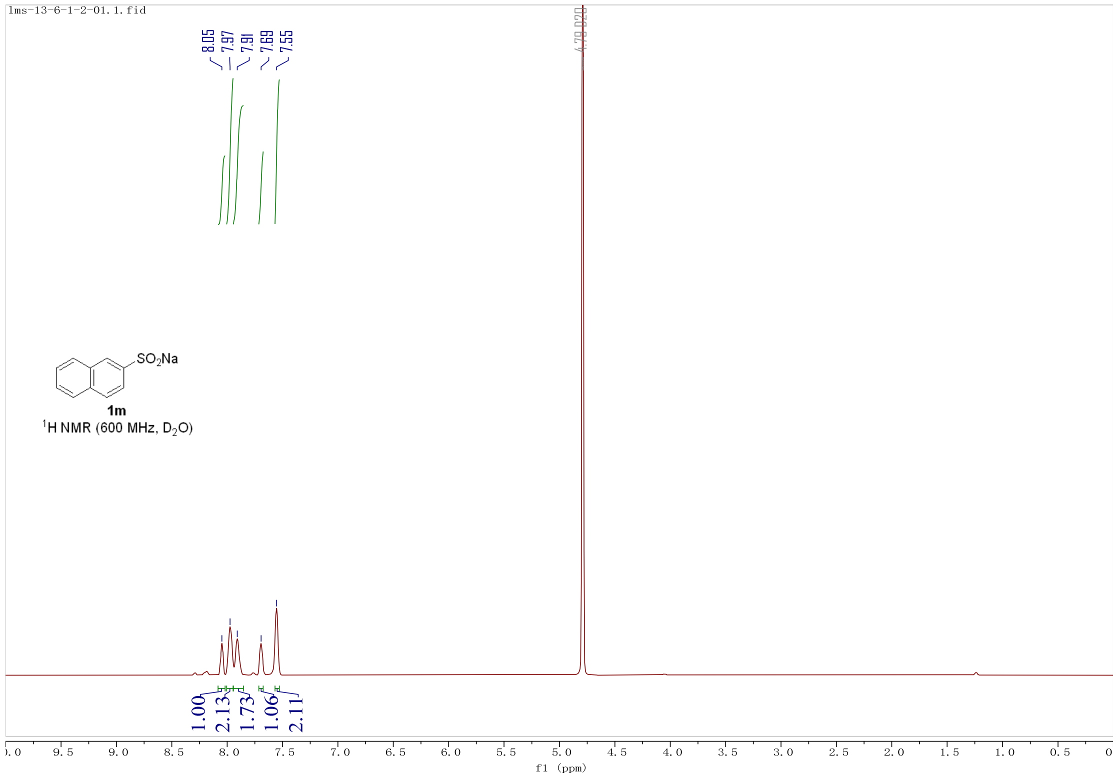

1m

^{1}H NMR (600 MHz, D_2O)

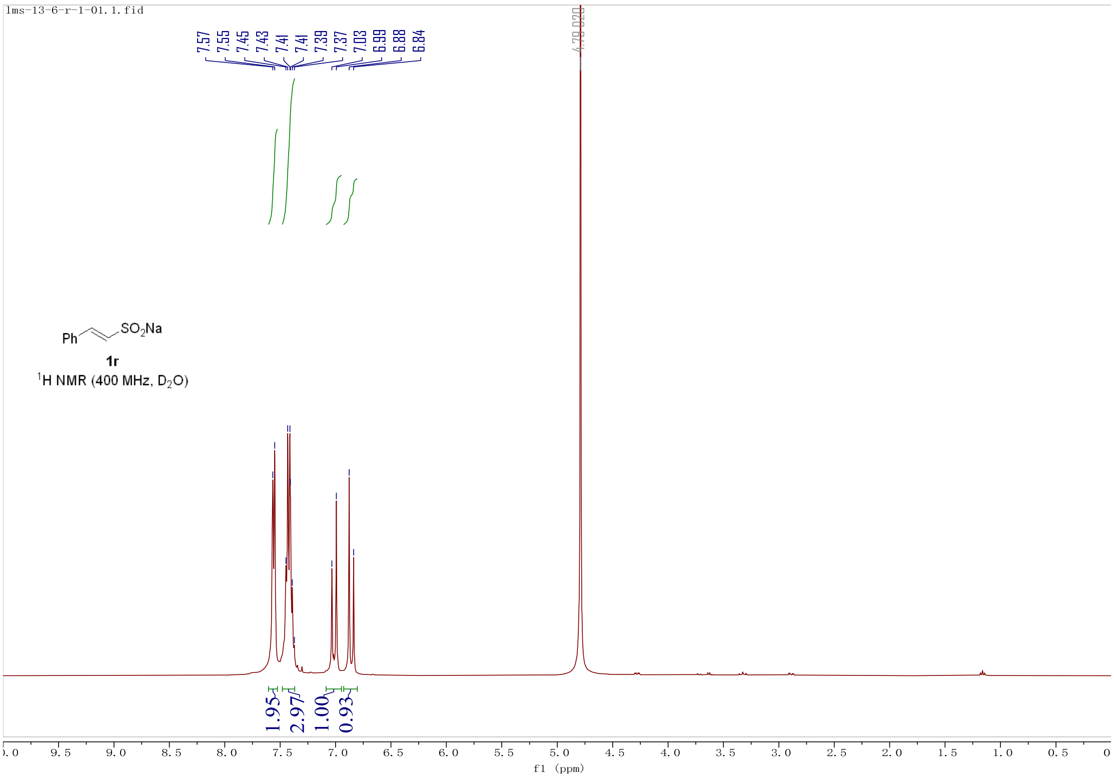
lms-13-6-r-1-01.1.fid
7.57
7.55
7.45
7.43
7.41
7.41
7.39
7.37
7.03
6.99
6.88
6.84
4.79 D2O
Ph
SO2Na
1r
1H NMR (400 MHz, D2O)
1.95
2.97
1.00
0.93
f1 (ppm)

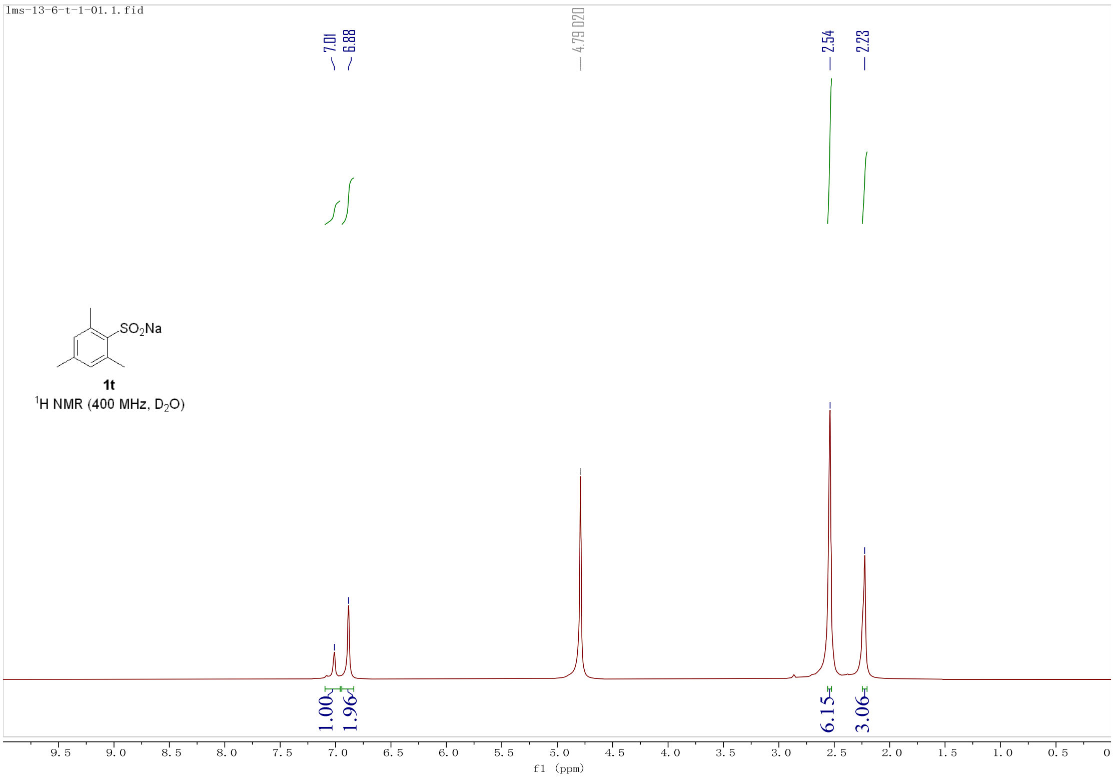
lms-13-6-t-1-01.1.fid
7.01
6.88
4.79 D2O
2.54
2.23
SO2Na
1t
1H NMR (400 MHz, D2O)
1.00
1.96
6.15
3.06
f1 (ppm)

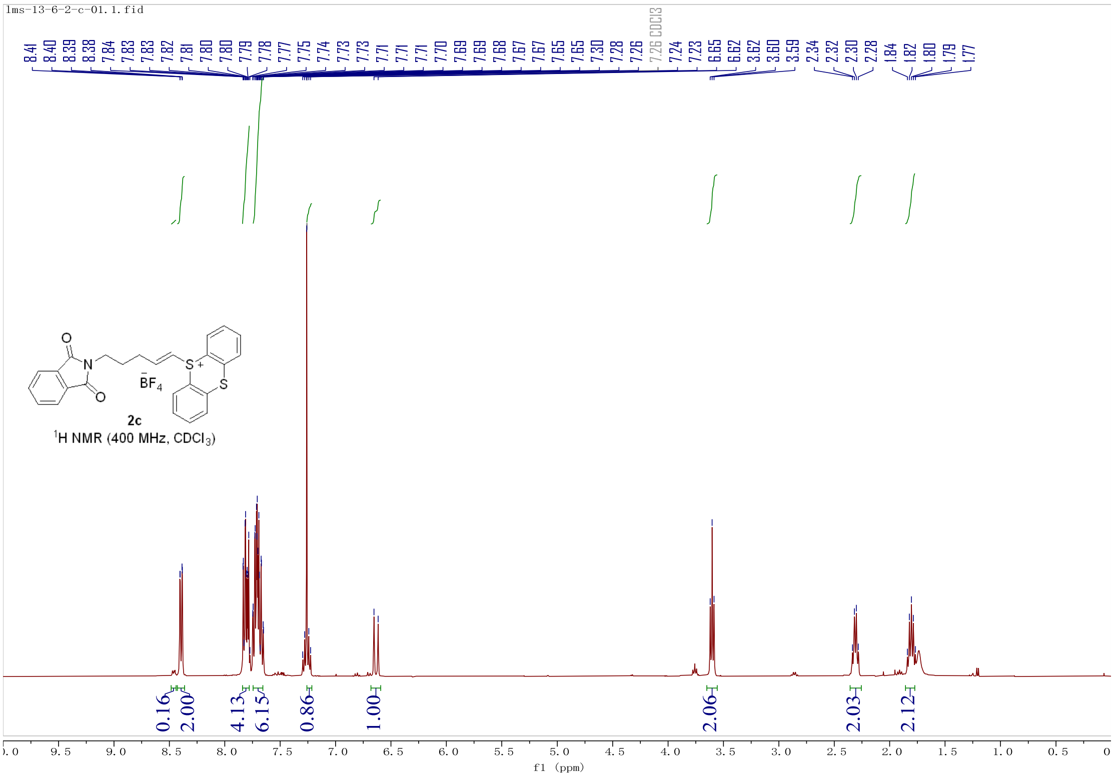
lms-13-6-2-c-01.1.fid
8.41 8.40 8.39 8.38 7.84 7.83 7.83 7.82 7.81 7.80 7.80 7.79 7.78 7.77 7.75 7.74 7.73 7.73 7.71 7.71 7.71 7.70 7.69 7.69 7.68 7.67 7.67 7.65 7.65 7.30 7.28 7.26 7.26 CDCl3 7.24 7.23 6.65 6.62 3.62 3.60 3.59 2.34 2.32 2.30 2.28 1.84 1.82 1.80 1.79 1.77
$\bar{B}F_4$
2c
1H NMR (400 MHz, $CDCl_3$)
0.16 2.00 4.13 6.15 0.86 1.00 2.06 2.03 2.12
f1 (ppm)

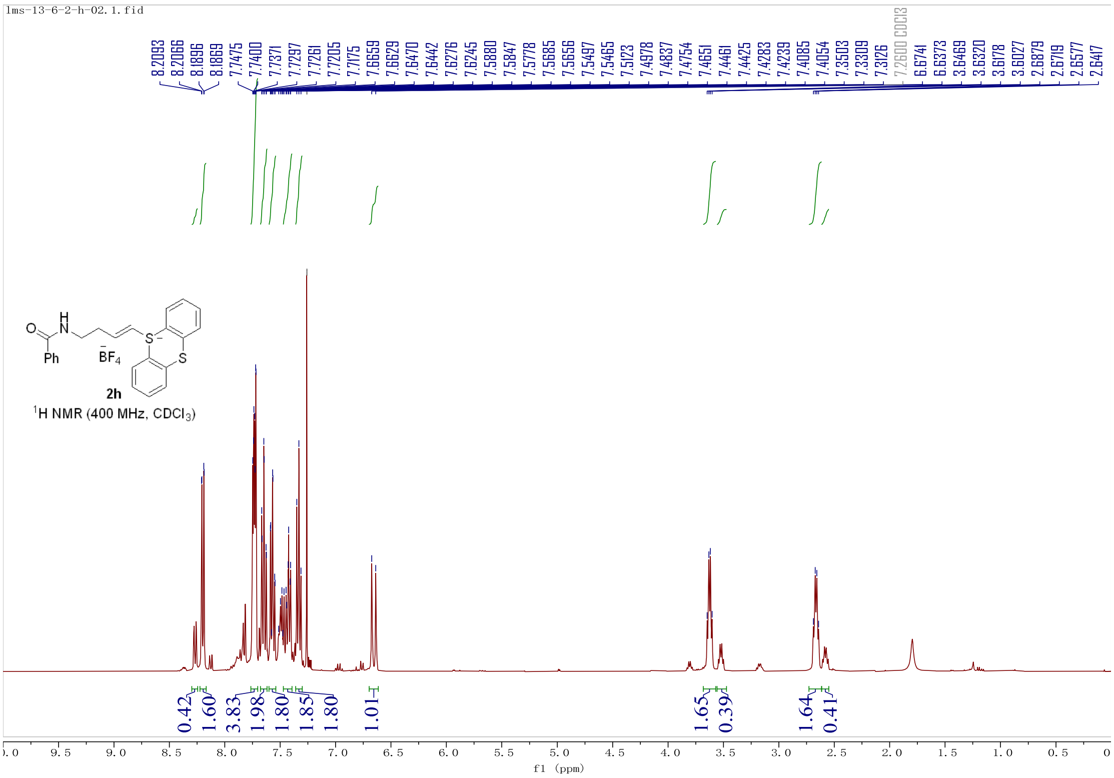
lms-13-6-2-h-02.1.fid
8.2093 8.2066 8.1896 8.1869 7.7475 7.7400 7.7371 7.7297 7.7261 7.7205 7.7175 7.6659 7.6629 7.6470 7.6442 7.6276 7.6245 7.5880 7.5847 7.5778 7.5685 7.5656 7.5497 7.5465 7.5123 7.4978 7.4837 7.4754 7.4651 7.4461 7.4425 7.4283 7.4239 7.4085 7.4054 7.3503 7.3309 7.3126 7.2600 CDCl3 6.6741 6.6373 3.6469 3.6320 3.6178 3.6027 2.6879 2.6719 2.6577 2.6417
Ph
$\bar{B}F_4$
2h
1H NMR (400 MHz, $CDCl_3$)
0.42 1.60 3.83 1.98 1.80 1.85 1.80 1.01 1.65 0.39 1.64 0.41
f1 (ppm)

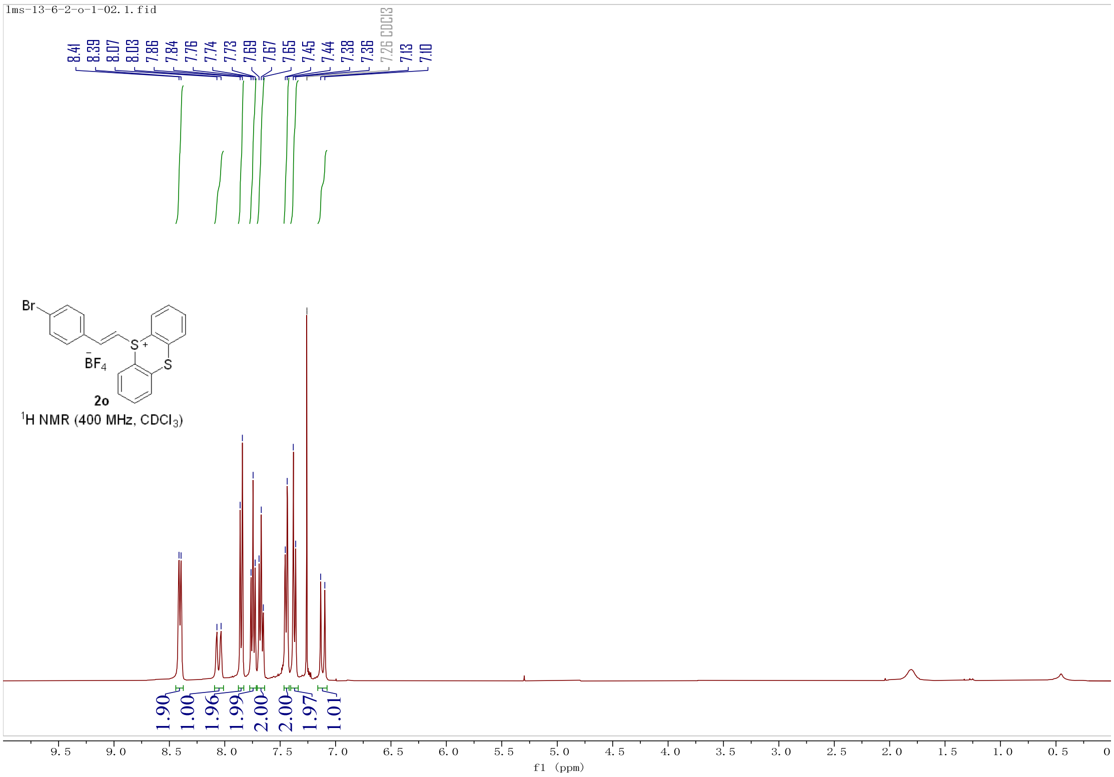
lms-13-6-2-o-1-02.1.fid
8.41
8.39
8.07
8.03
7.86
7.84
7.76
7.74
7.73
7.69
7.67
7.65
7.45
7.44
7.38
7.36
7.26 CDCl3
7.13
7.10
Br
S+
S
$\bar{B}F_4$
2o
^{1}H NMR (400 MHz, $CDCl_3$)
1.90
1.00
1.96
1.99
2.00
2.00
1.97
1.01
9.5
9.0
8.5
8.0
7.5
7.0
6.5
6.0
5.5
5.0
4.5
4.0
3.5
3.0
2.5
2.0
1.5
1.0
0.5
0
f1 (ppm)

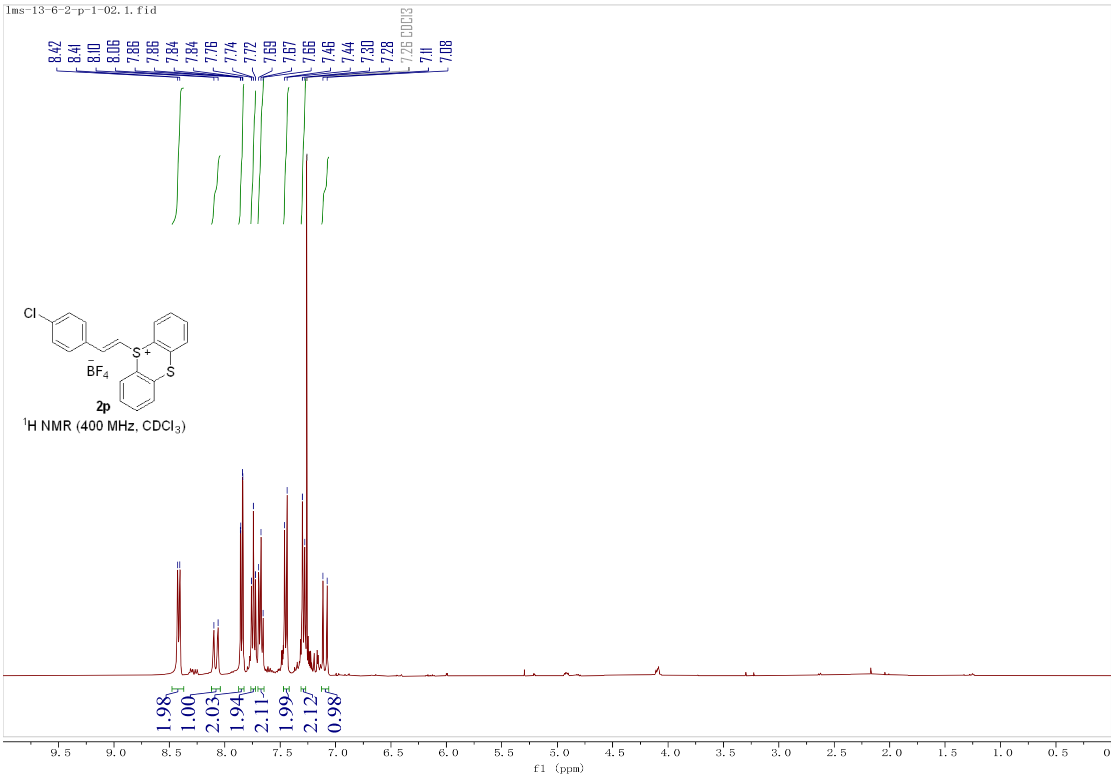
lms-13-6-2-p-1-02.1.fid
8.42
8.41
8.10
8.06
7.86
7.86
7.84
7.84
7.76
7.74
7.72
7.69
7.67
7.66
7.46
7.44
7.30
7.28
7.26 CDCl3
7.11
7.08
Cl
S+
S
$\bar{B}F_4$
2p
^{1}H NMR (400 MHz, $CDCl_3$)
1.98
1.00
2.03
1.94
2.11
1.99
2.12
0.98
9.5
9.0
8.5
8.0
7.5
7.0
6.5
6.0
5.5
5.0
4.5
4.0
3.5
3.0
2.5
2.0
1.5
1.0
0.5
0
f1 (ppm)

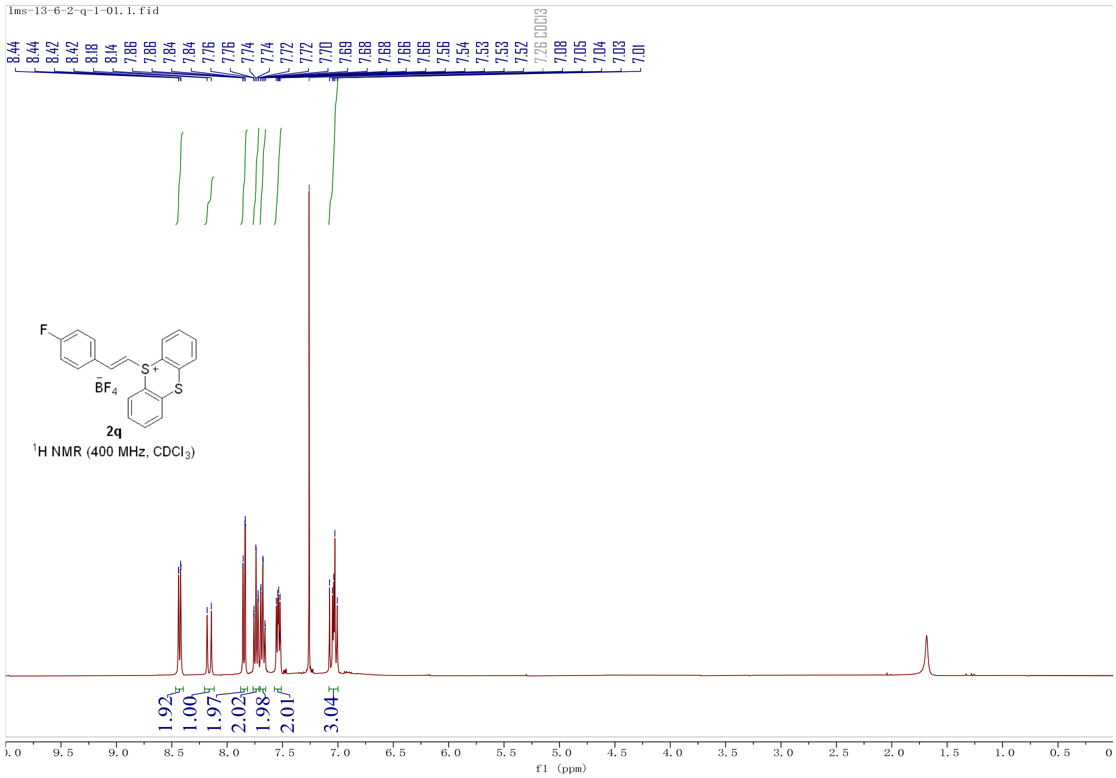
lms-13-6-2-q-1-01.1.fid
8.44 8.44 8.42 8.42 8.18 8.14 7.86 7.86 7.84 7.84 7.76 7.76 7.74 7.74 7.72 7.70 7.69 7.68 7.68 7.66 7.66 7.56 7.54 7.53 7.53 7.52 7.26 CDCl3 7.08 7.05 7.04 7.03 7.01
F
BF4
S+
S
2q
1H NMR (400 MHz, CDCl3)
1.92 1.00 1.97 2.02 1.98 2.01 3.04
f1 (ppm)

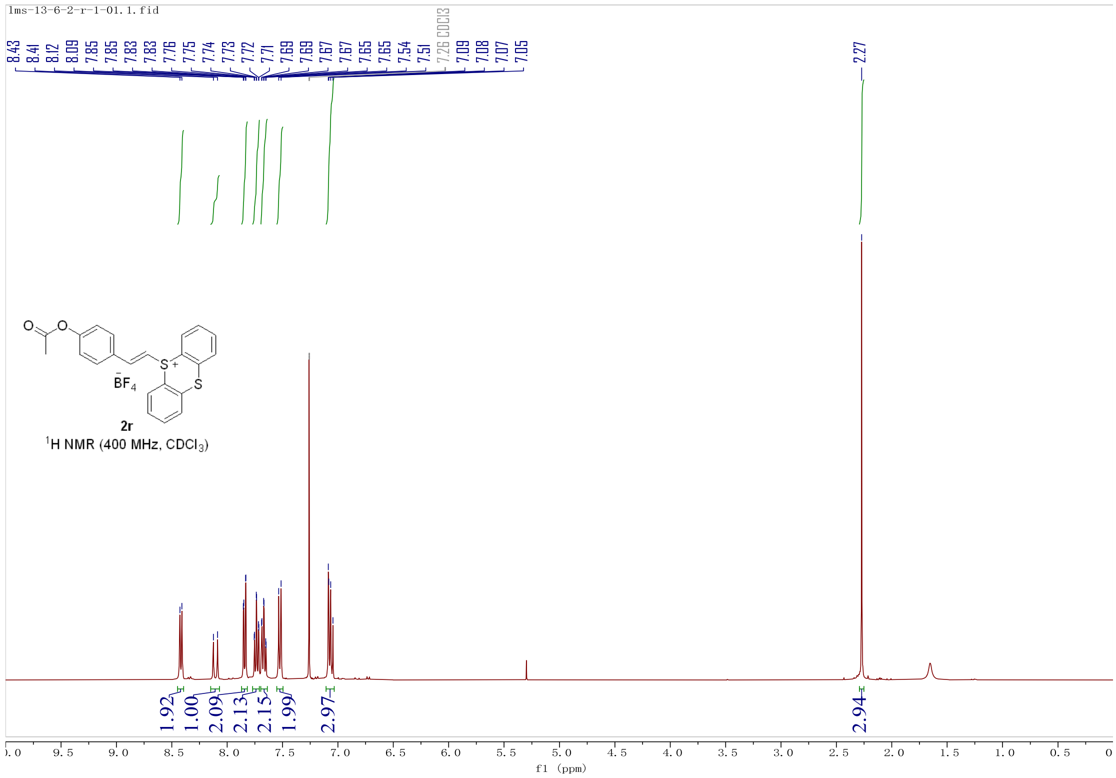
lms-13-6-2-r-1-01.1.fid
8.43 8.41 8.12 8.09 7.85 7.85 7.83 7.83 7.76 7.75 7.74 7.73 7.72 7.71 7.69 7.67 7.67 7.65 7.65 7.54 7.51 7.26 CDCl3 7.09 7.08 7.07 7.05 2.27
O
O
BF4
S+
S
2r
1H NMR (400 MHz, CDCl3)
1.92 1.00 2.09 2.13 2.15 1.99 2.97 2.94
f1 (ppm)

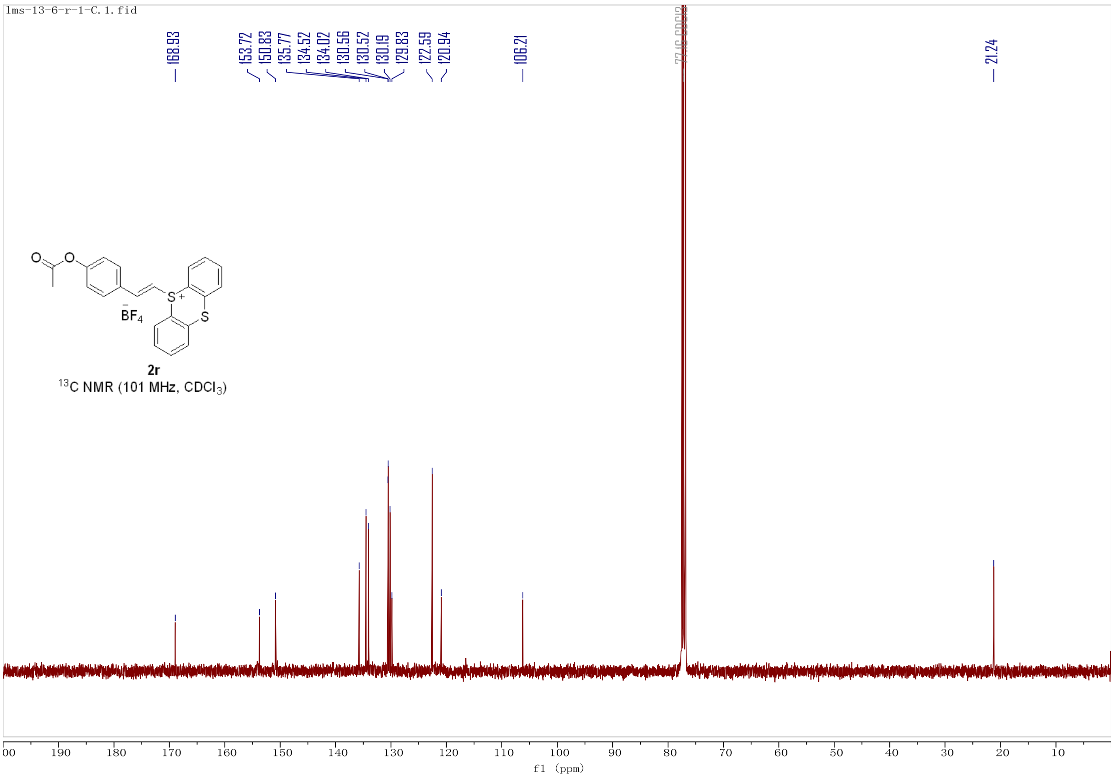
lms-13-6-r-1-C.1.fid
168.93
153.72
150.83
135.77
134.52
134.02
130.56
130.52
130.19
129.83
122.59
120.94
106.21
21.24
2r
13C NMR (101 MHz, CDCl3)
f1 (ppm)

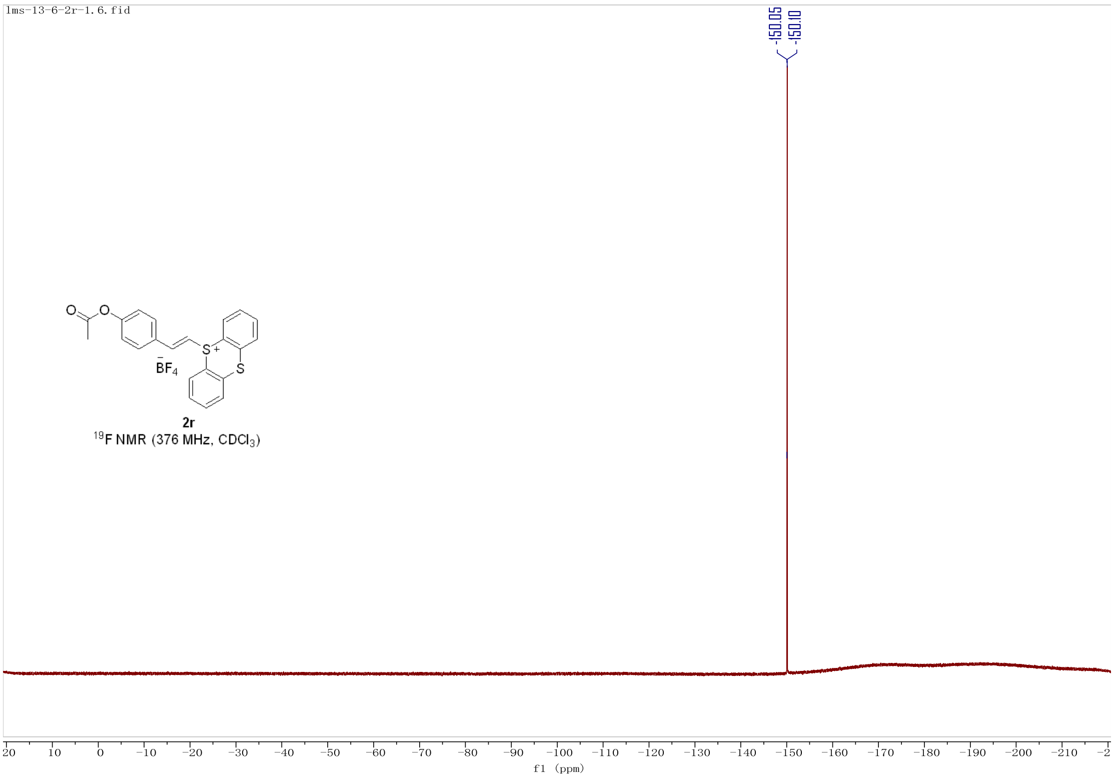
lms-13-6-2r-1.6.fid
-150.05
-150.10
2r
19F NMR (376 MHz, CDCl3)
f1 (ppm)

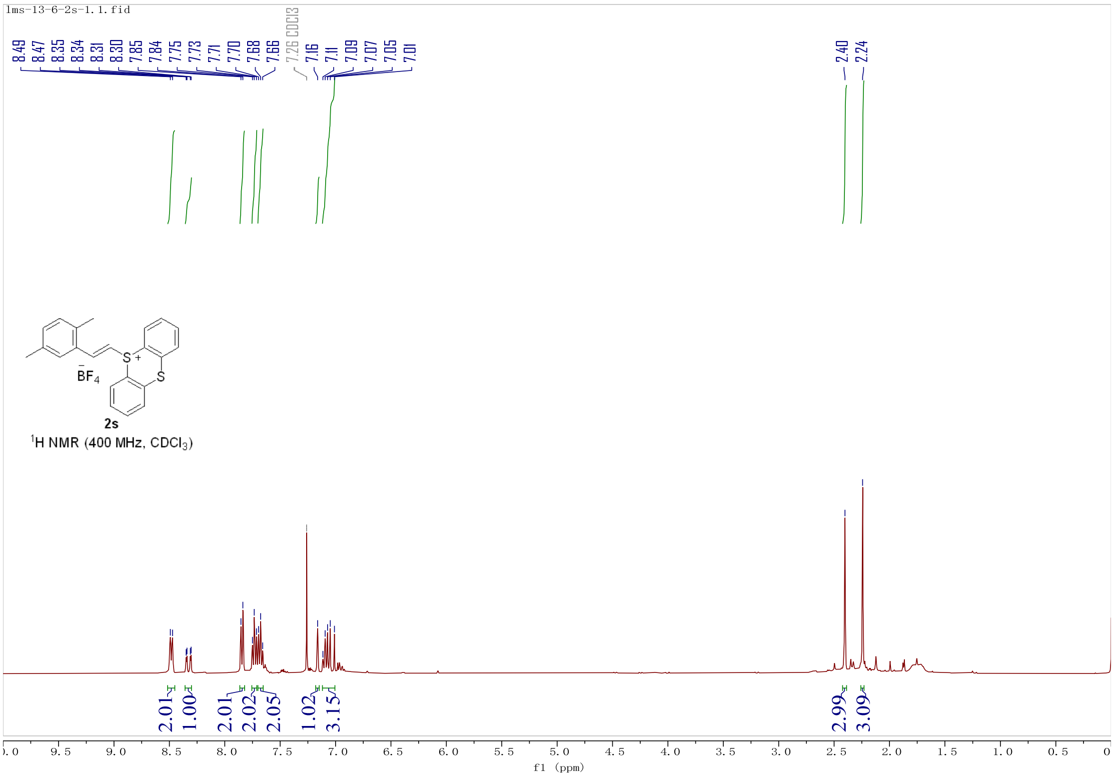

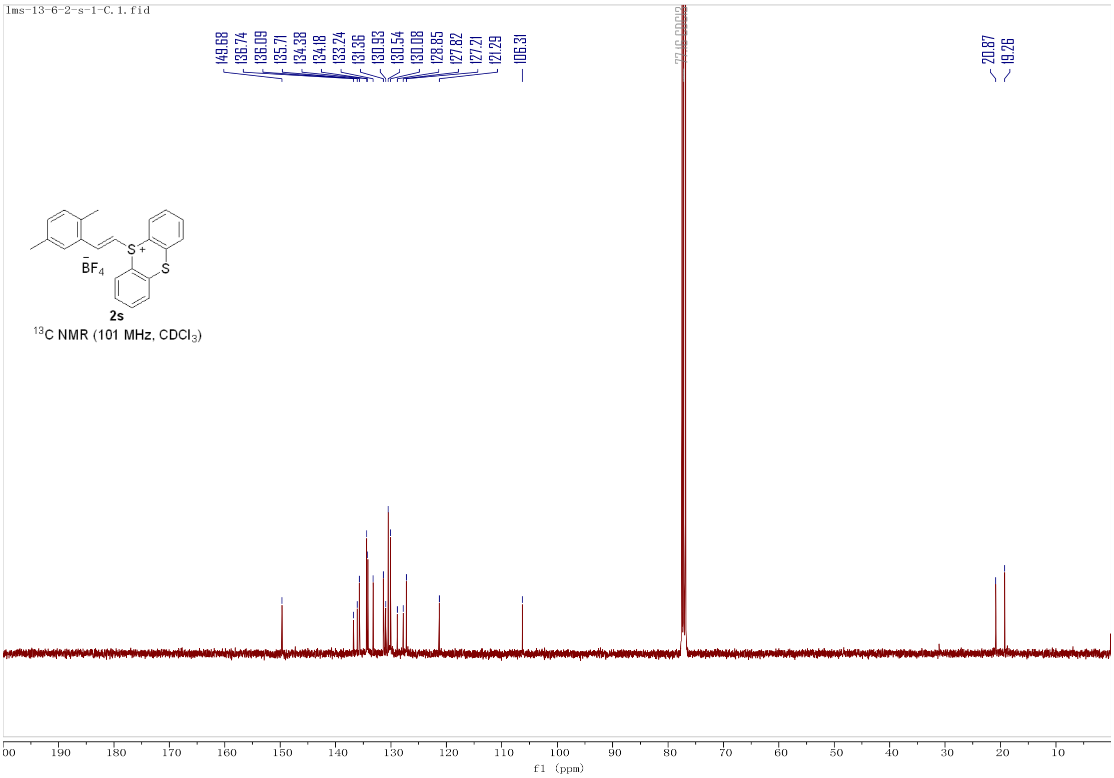

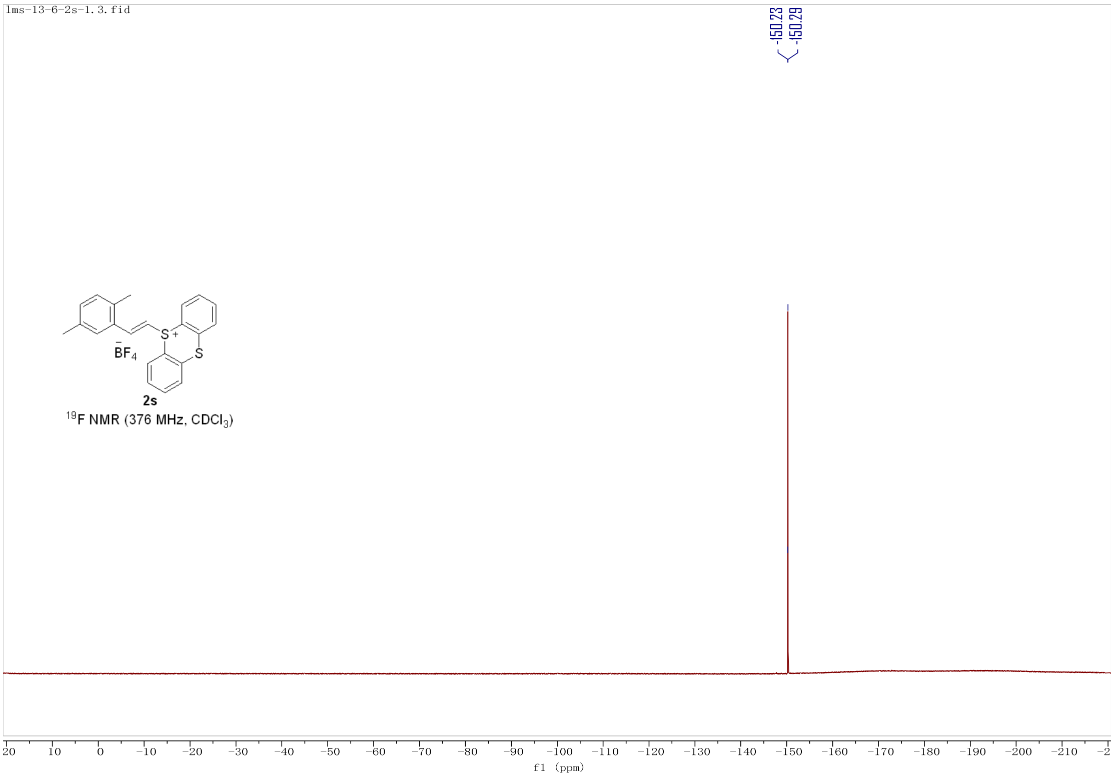
lms-13-6-2s-1.3.fid
-150.23
-150.29
BF4
2s
19F NMR (376 MHz, CDCl3)
20 10 0 −10 −20 −30 −40 −50 −60 −70 −80 −90 −100 −110 −120 −130 −140 −150 −160 −170 −180 −190 −200 −210
f1 (ppm)

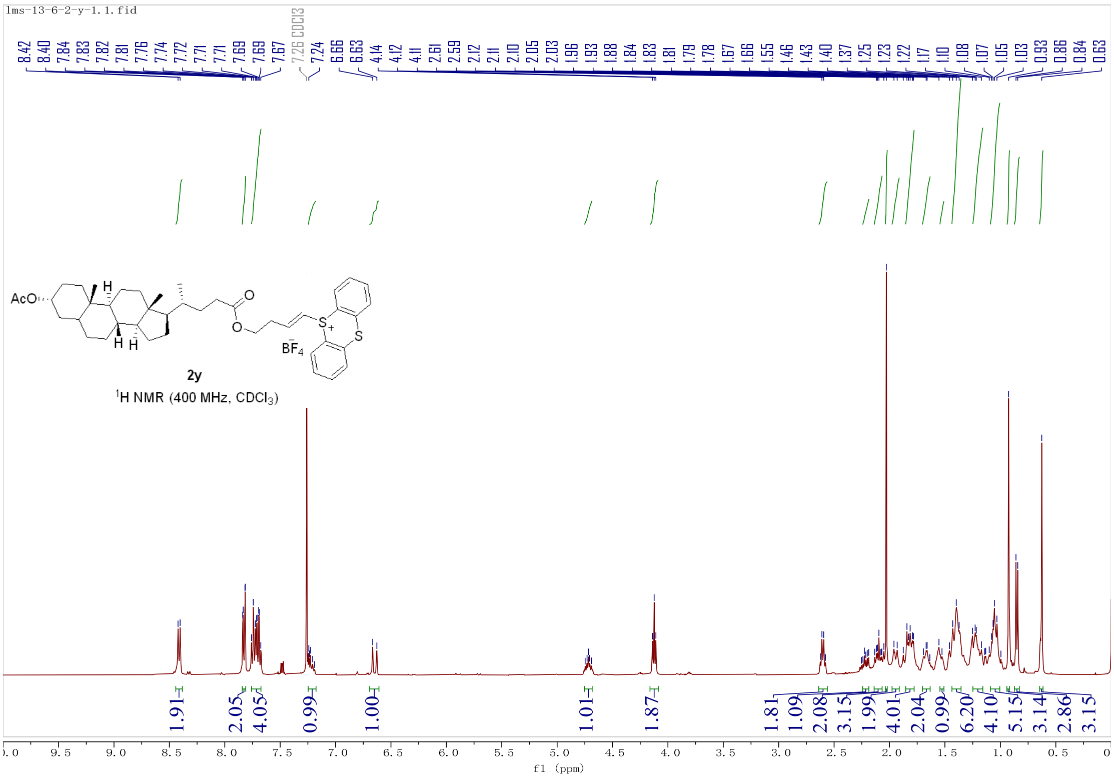
lms-13-6-2-y-1.1.fid
8.42 8.40 7.84 7.83 7.82 7.81 7.76 7.74 7.72 7.71 7.71 7.69 7.69 7.67
7.26 CDCl3 7.24 6.66 6.63 4.14 4.12 4.11 2.61 2.59 2.12 2.11 2.10 2.05 2.03 1.96 1.93 1.88 1.84 1.83 1.81 1.79 1.78 1.67 1.66 1.55 1.46 1.43 1.40 1.37 1.25 1.23 1.22 1.17 1.10 1.08 1.07 1.05 1.03 0.93 0.86 0.84 0.63
AcO
H
H H
O
O
S
S
BF4
2y
1H NMR (400 MHz, CDCl3)
1.91 2.05 4.05 0.99 1.00 1.01 1.87 1.81 1.09 2.08 3.15 1.99 4.01 2.04 0.99 6.20 4.10 5.15 3.14 2.86 3.15
10.0 9.5 9.0 8.5 8.0 7.5 7.0 6.5 6.0 5.5 5.0 4.5 4.0 3.5 3.0 2.5 2.0 1.5 1.0 0.5 0
f1 (ppm)

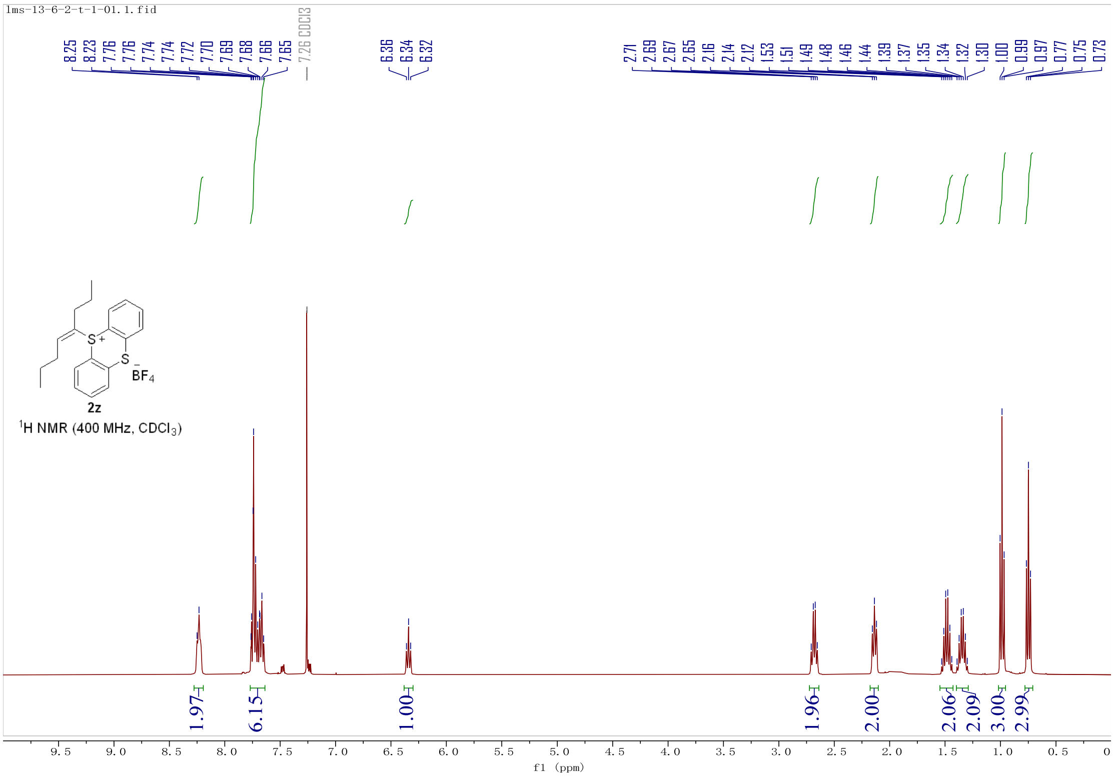
lms-13-6-2-t-1-01.1.fid
8.25 8.23 7.76 7.76 7.74 7.74 7.72 7.70 7.69 7.68 7.66 7.65
7.26 CDCl3
6.36 6.34 6.32
2.71 2.69 2.67 2.65 2.16 2.14 2.12 1.53 1.51 1.49 1.48 1.46 1.44 1.39 1.37 1.35 1.34 1.32 1.30 1.00 0.99 0.97 0.77 0.75 0.73
S+
S
BF4
2z
1H NMR (400 MHz, CDCl3)
1.97 6.15 1.00 1.96 2.00 2.06 2.09 3.00 2.99
9.5 9.0 8.5 8.0 7.5 7.0 6.5 6.0 5.5 5.0 4.5 4.0 3.5 3.0 2.5 2.0 1.5 1.0 0.5 0
f1 (ppm)

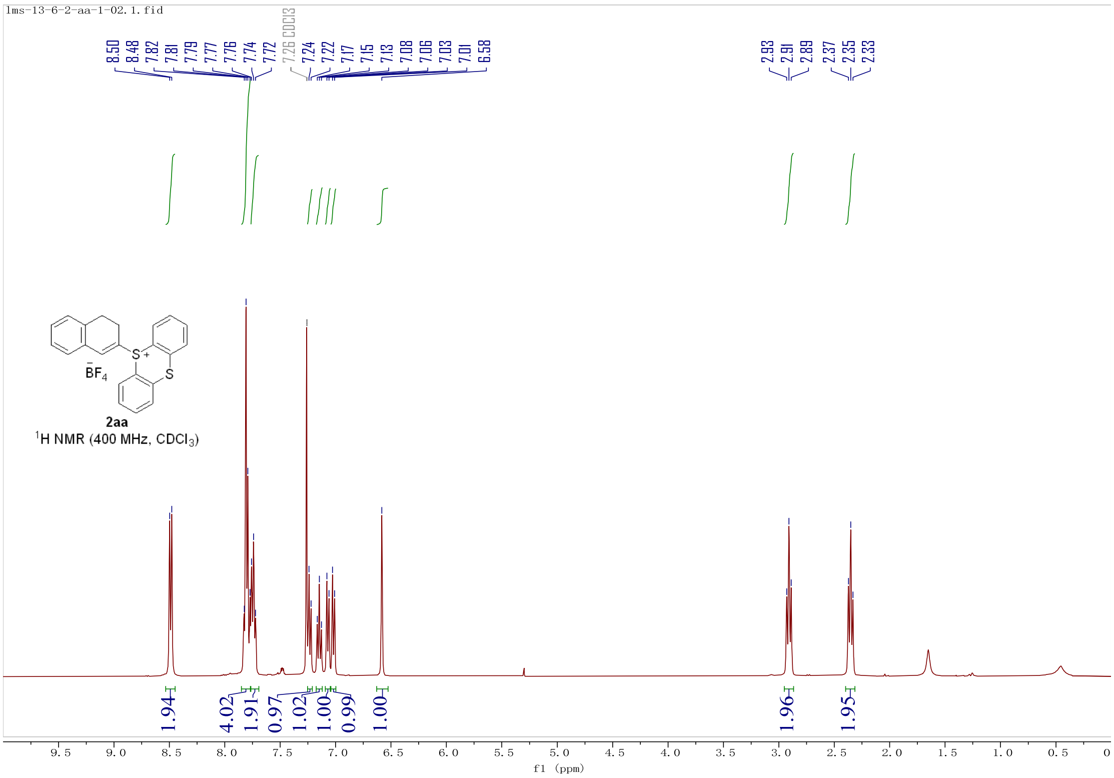
lms-13-6-2-aa-1-02.1.fid
8.50 8.48 7.82 7.81 7.79 7.77 7.76 7.74 7.72
7.26 CDCl3
7.24 7.22 7.17 7.15 7.13 7.08 7.06 7.03 7.01 6.58
2.93 2.91 2.89 2.37 2.35 2.33
S+
S
BF4
2aa
1H NMR (400 MHz, CDCl3)
1.94 4.02 1.91 0.97 1.02 1.00 0.99 1.00 1.96 1.95
9.5 9.0 8.5 8.0 7.5 7.0 6.5 6.0 5.5 5.0 4.5 4.0 3.5 3.0 2.5 2.0 1.5 1.0 0.5 0
f1 (ppm)

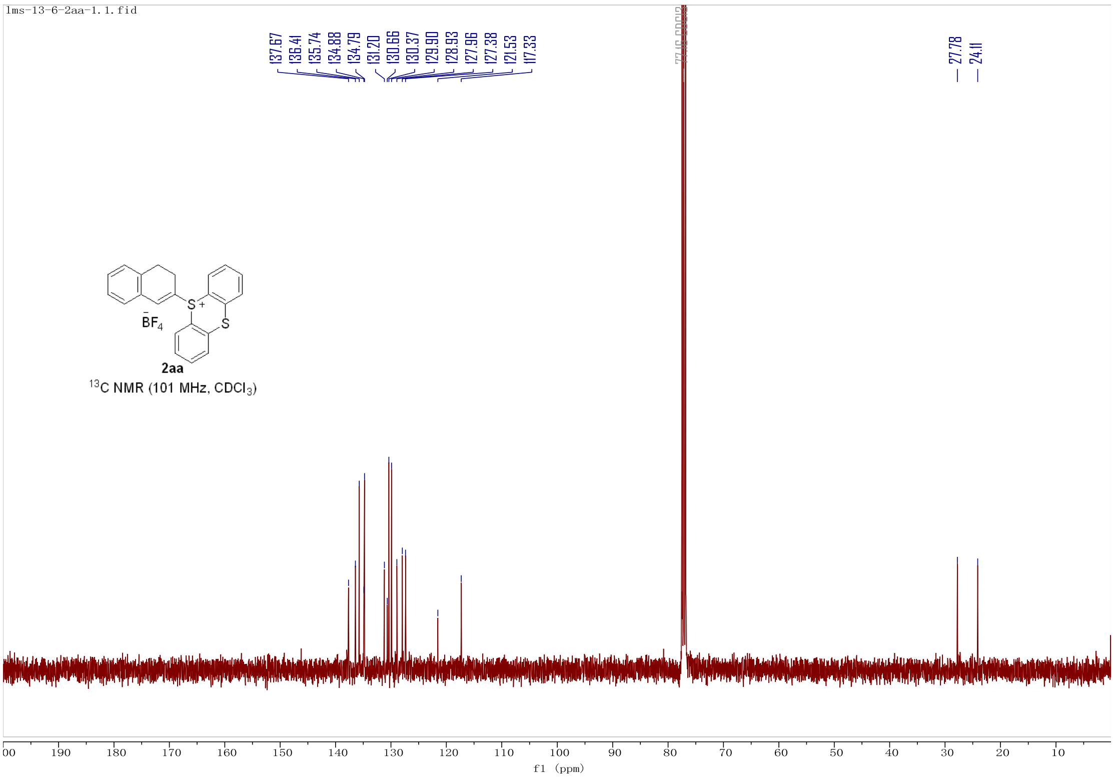
lms-13-6-2aa-1.1.fid
137.67
136.41
135.74
134.88
134.79
131.20
130.66
130.37
129.90
128.93
127.96
127.38
121.53
117.33
27.78
24.11
2aa
^{13}C NMR (101 MHz, CDCl$_3$)
f1 (ppm)

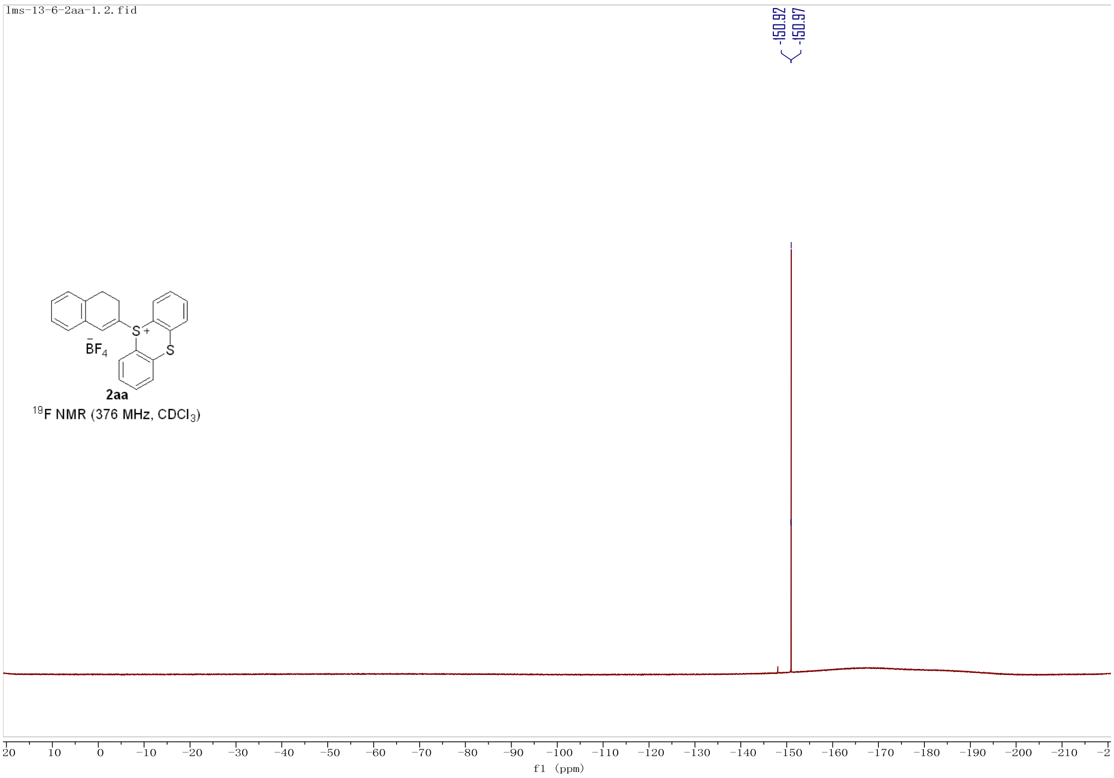
lms-13-6-2aa-1.2.fid
-150.92
-150.97
2aa
^{19}F NMR (376 MHz, CDCl$_3$)
f1 (ppm)

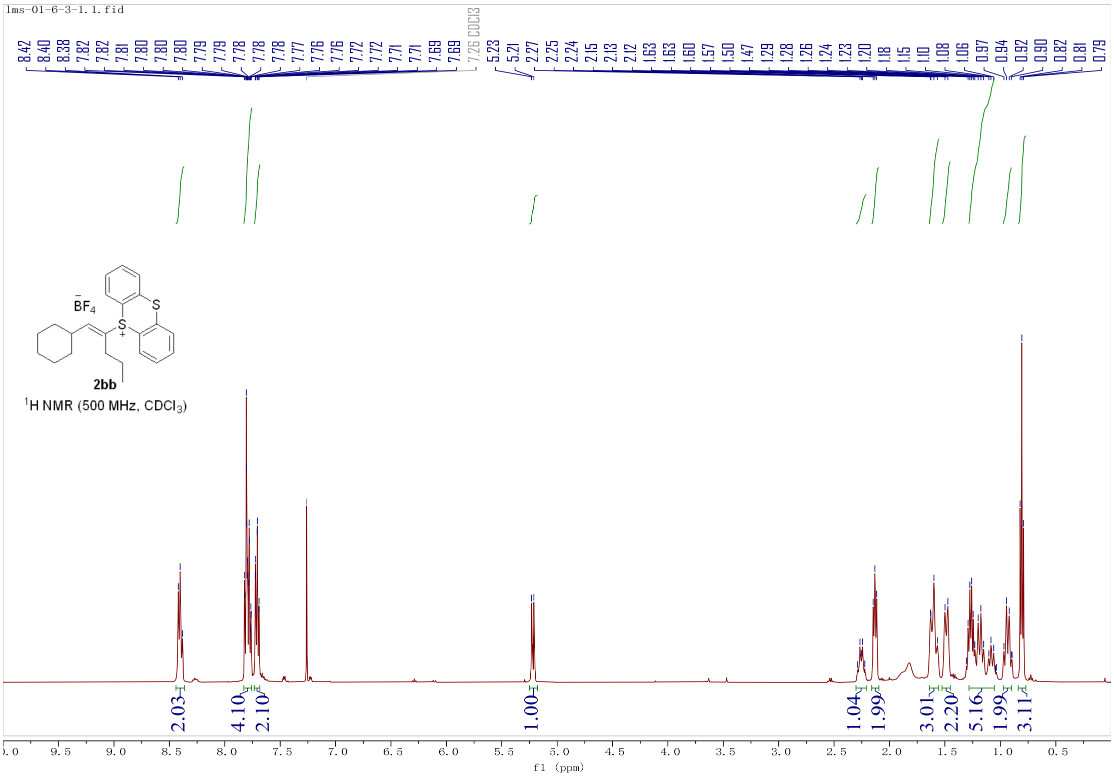

lms-01-6-3-1.1.fid
8.42 8.40 8.38 7.82 7.82 7.81 7.80 7.80 7.79 7.79 7.78 7.78 7.78 7.77 7.76 7.76 7.72 7.72 7.71 7.71 7.69 7.69
7.26 CDCl3
5.23 5.21 2.27 2.25 2.24 2.15 2.13 2.12 1.63 1.63 1.60 1.57 1.50 1.47 1.29 1.28 1.26 1.24 1.23 1.20 1.18 1.15 1.10 1.08 1.06 0.97 0.94 0.92 0.90 0.82 0.81 0.79
2.03 4.10 2.10 1.00 1.04 1.99 3.01 2.20 5.16 1.99 3.11
2bb
1H NMR (500 MHz, CDCl3)
f1 (ppm)

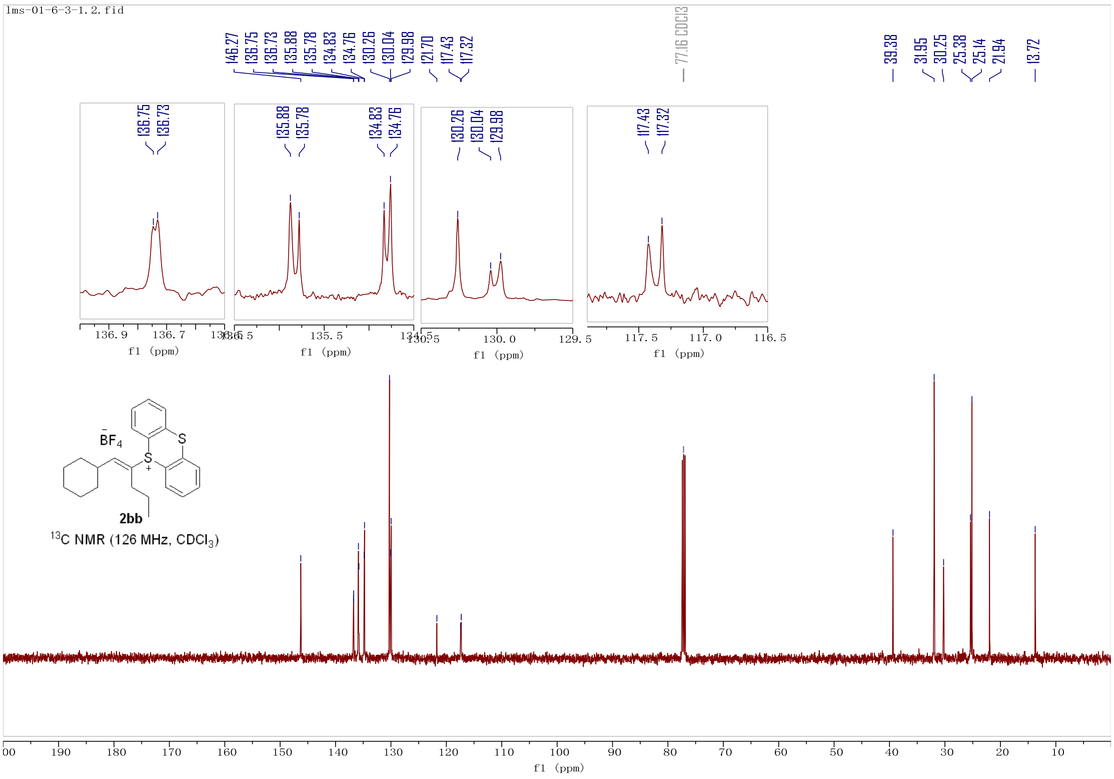

lms-01-6-3-1.2.fid
146.27 136.75 136.73 135.88 135.78 134.83 134.76 130.26 130.04 129.98 121.70 117.43 117.32
77.16 CDCl3
39.38 31.95 30.25 25.38 25.14 21.94 13.72
2bb
13C NMR (126 MHz, CDCl3)
f1 (ppm)

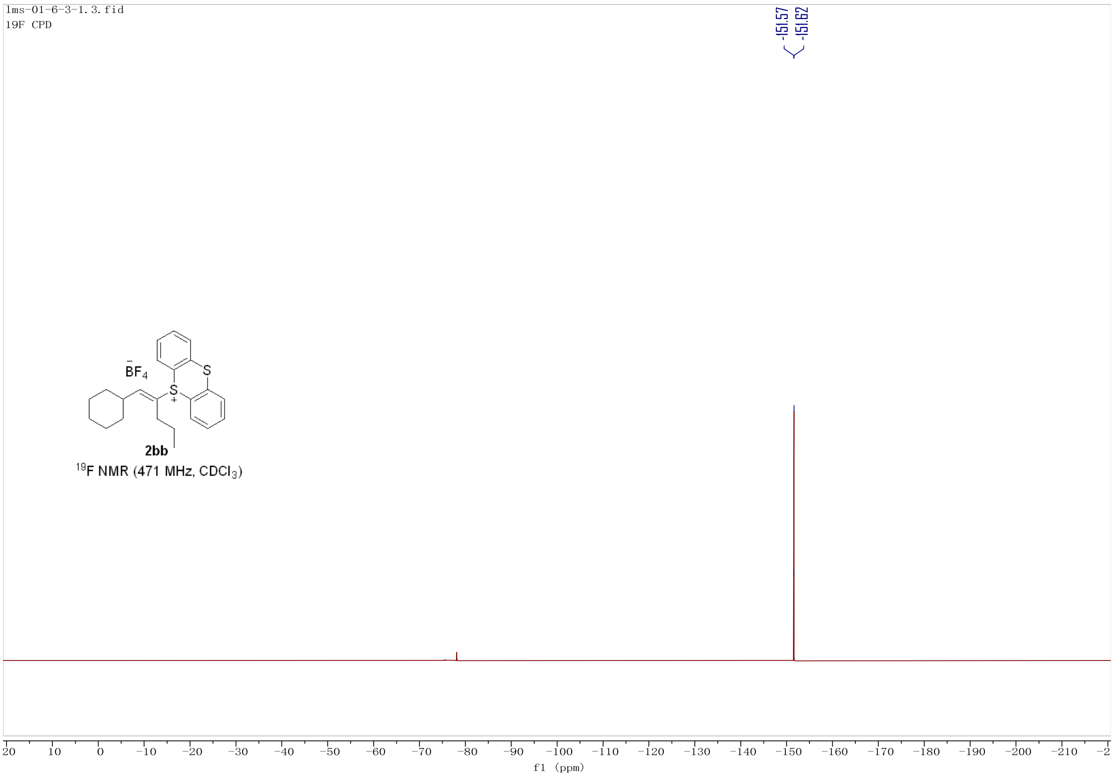
lms-01-6-3-1.3.fid
19F CPD
-151.57
-151.62
BF4
S
S
+
2bb
19F NMR (471 MHz, CDCl3)
f1 (ppm)

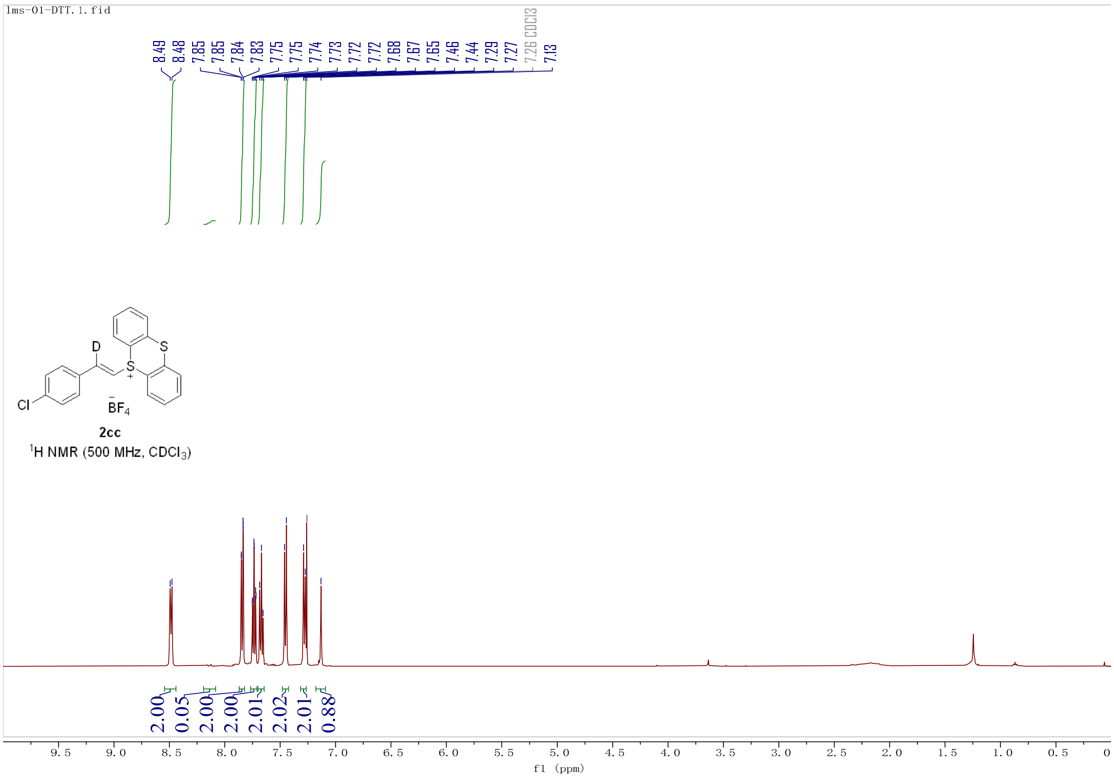
lms-01-DTT.1.fid
8.49
8.48
7.85
7.84
7.83
7.75
7.75
7.74
7.73
7.72
7.72
7.68
7.67
7.65
7.46
7.44
7.29
7.27
7.26 CDCl3
7.13
D
S
S
+
Cl
BF4
2cc
1H NMR (500 MHz, CDCl3)
2.00
0.05
2.00
2.00
2.01
2.02
2.01
0.88
f1 (ppm)

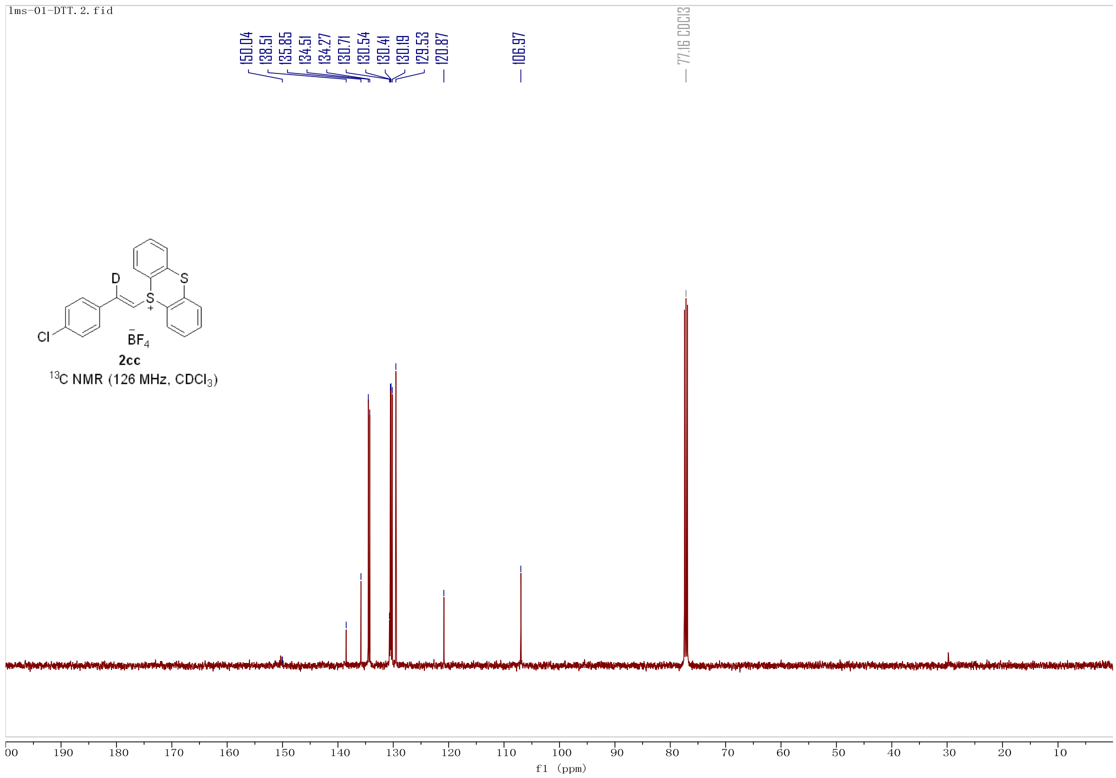

2cc

^{13}C NMR (126 MHz, $CDCl_3$)

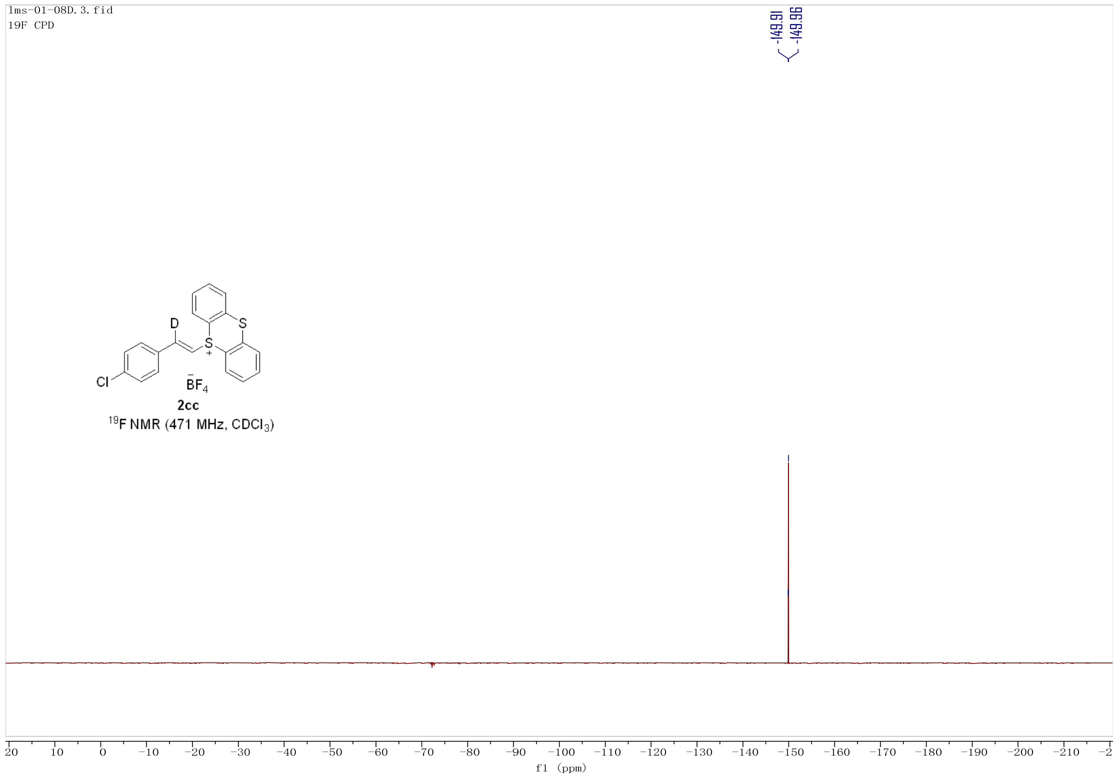

2cc

^{19}F NMR (471 MHz, $CDCl_3$)

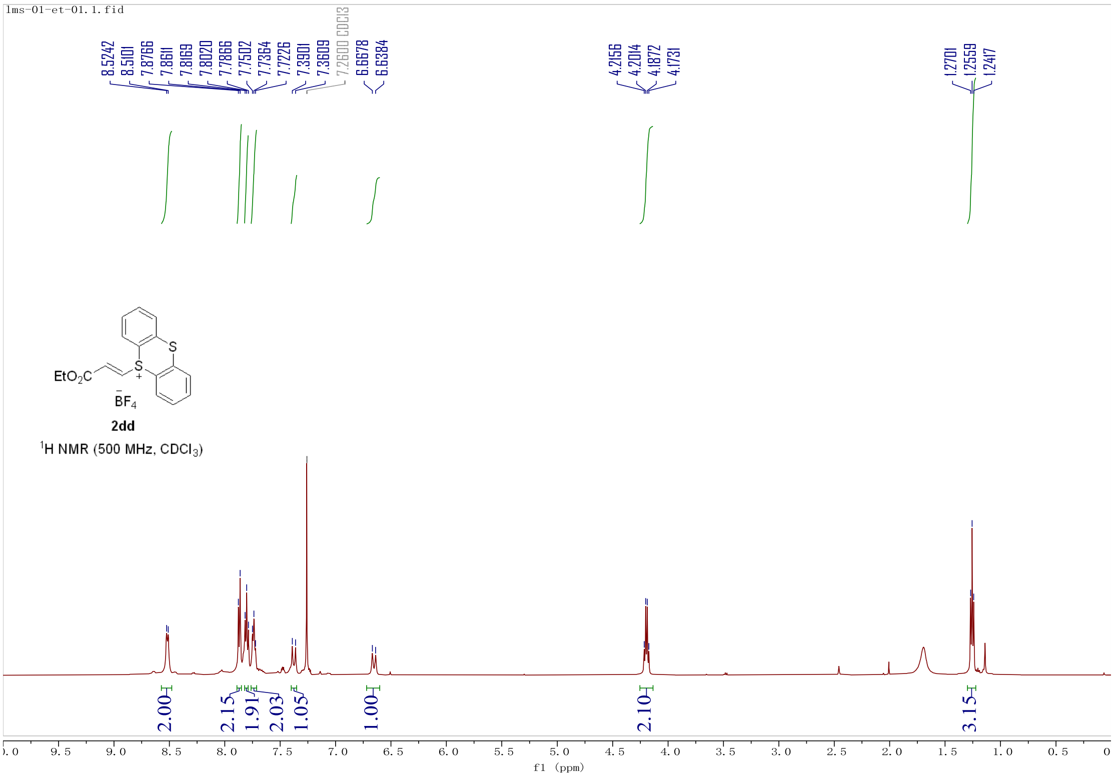
lms-01-et-01.1.fid
8.5242
8.5101
7.8766
7.8611
7.8169
7.8020
7.7866
7.7502
7.7364
7.7226
7.3901
7.3609
7.2600 CDCl3
6.6678
6.6384
4.2156
4.2014
4.1872
4.1731
1.2701
1.2559
1.2417
EtO2C
S
S
+
BF4
2dd
1H NMR (500 MHz, CDCl3)
2.00
2.15
1.91
2.03
1.05
1.00
2.10
3.15
f1 (ppm)

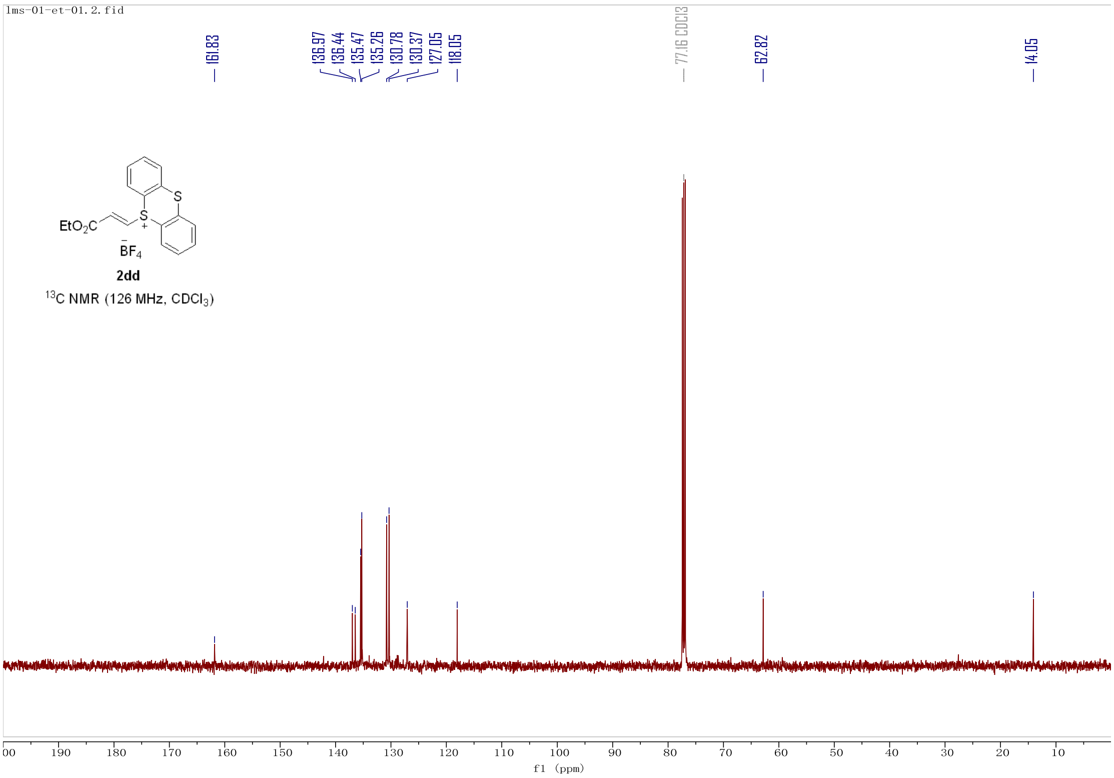
lms-01-et-01.2.fid
161.83
136.97
136.44
135.47
135.26
130.78
130.37
127.05
118.05
77.16 CDCl3
62.82
14.05
EtO2C
S
S
+
BF4
2dd
13C NMR (126 MHz, CDCl3)
f1 (ppm)

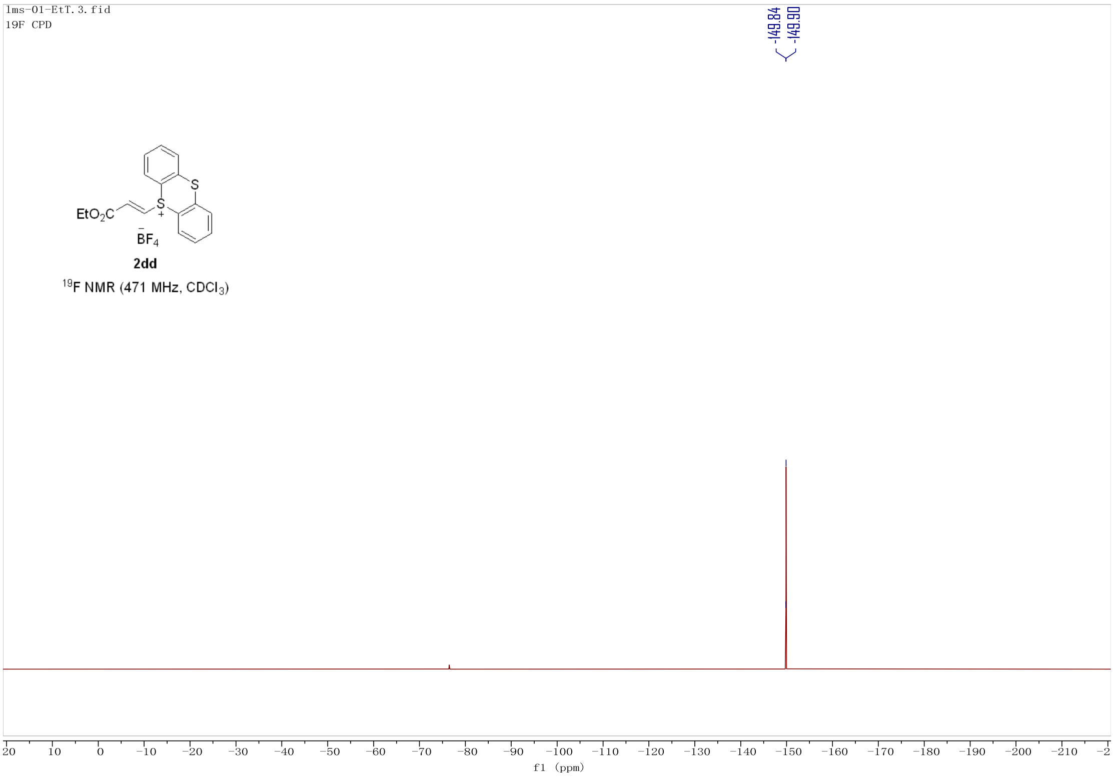
lms-01-EtT.3.fid
19F CPD
-149.84
-149.90
EtO2C
BF4
2dd
19F NMR (471 MHz, CDCl3)
f1 (ppm)

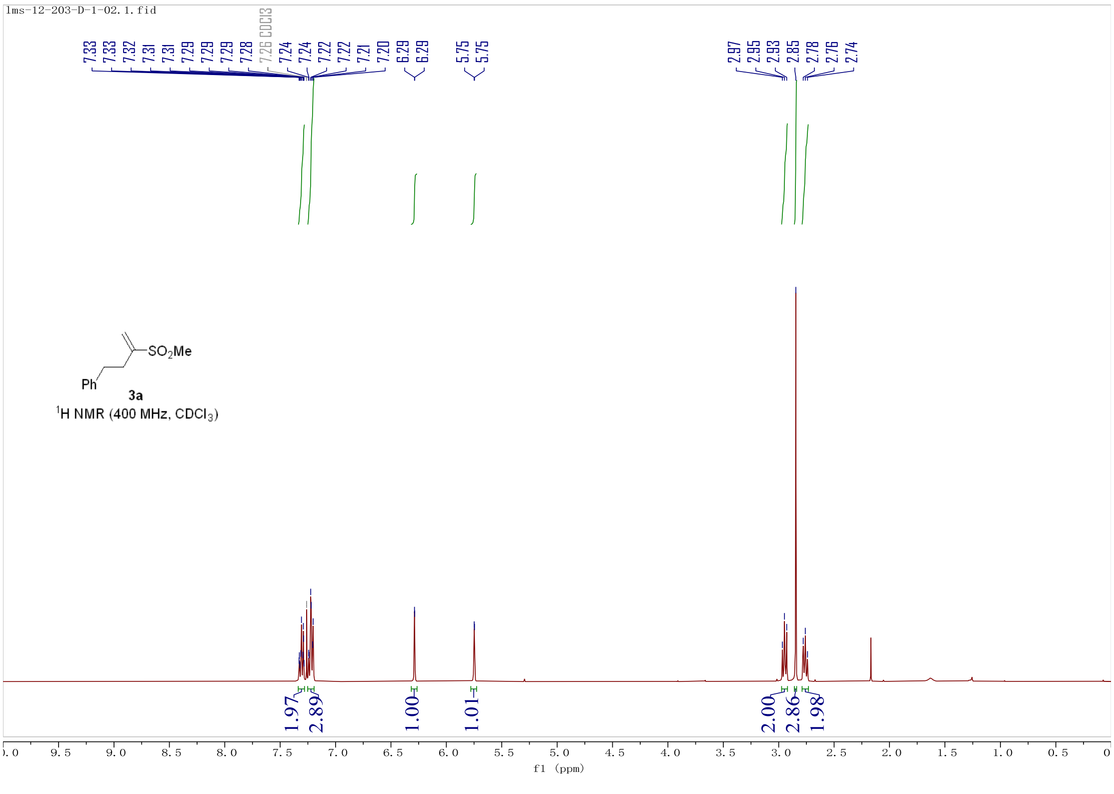
lms-12-203-D-1-02.1.fid
7.33
7.33
7.32
7.31
7.31
7.29
7.29
7.29
7.28
7.26 CDCl3
7.24
7.24
7.22
7.22
7.21
7.20
6.29
6.29
5.75
5.75
2.97
2.95
2.93
2.85
2.78
2.76
2.74
SO2Me
Ph
3a
1H NMR (400 MHz, CDCl3)
1.97
2.89
1.00
1.01
2.00
2.86
1.98
f1 (ppm)

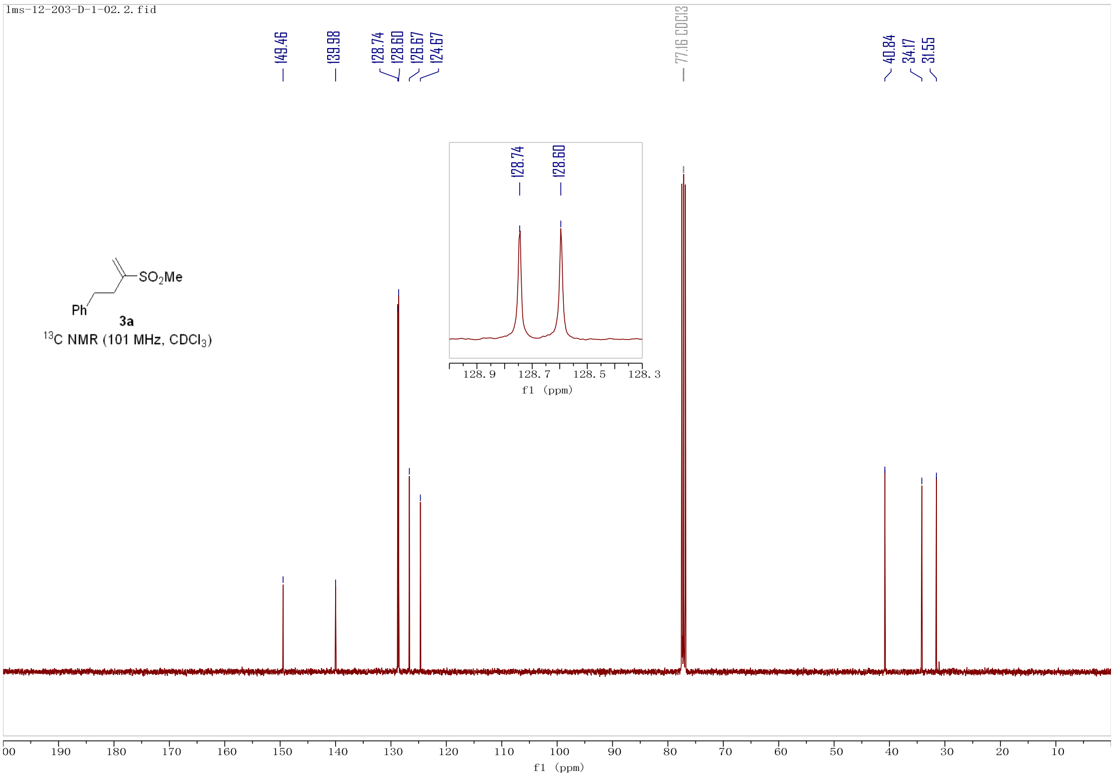
lms-12-203-D-1-02.2.fid
149.46
139.98
128.74
128.60
126.67
124.67
77.16 CDCl3
40.84
34.17
31.55
SO2Me
Ph
3a
13C NMR (101 MHz, CDCl3)
f1 (ppm)

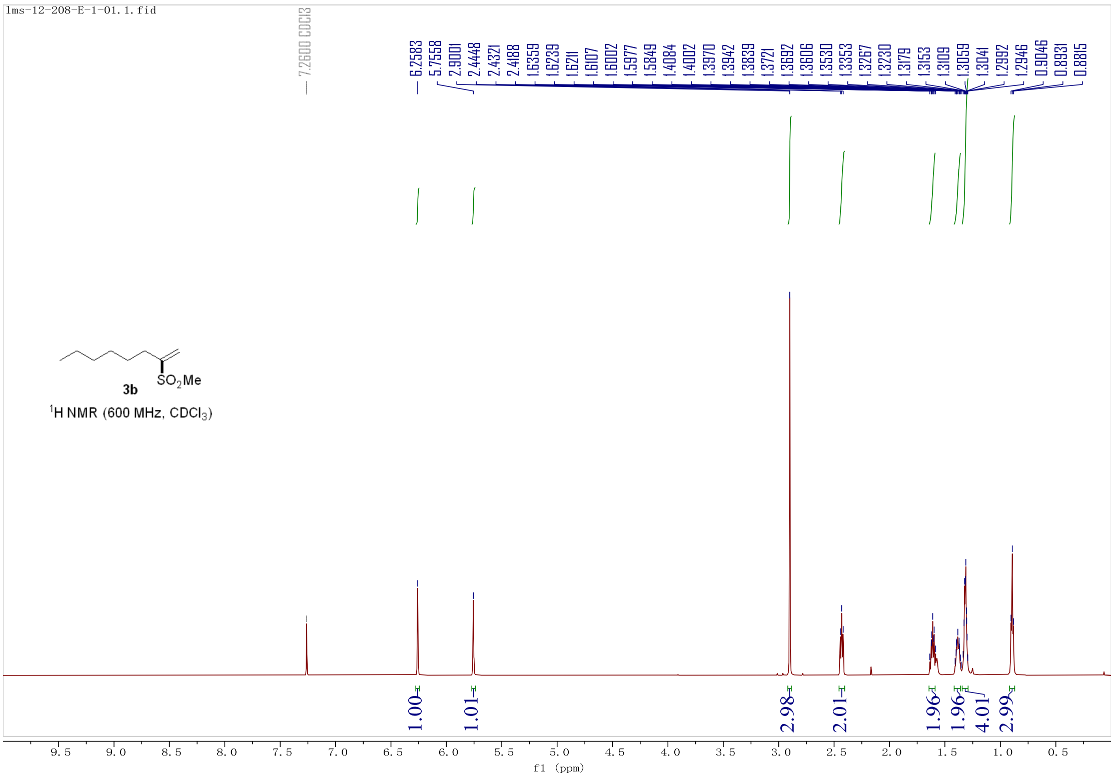
lms-12-208-E-1-01.1.fid
7.2600 CDCl3
SO2Me
3b
1H NMR (600 MHz, CDCl3)
1.00
1.01
2.98
2.01
1.96
1.96
4.01
2.99
f1 (ppm)

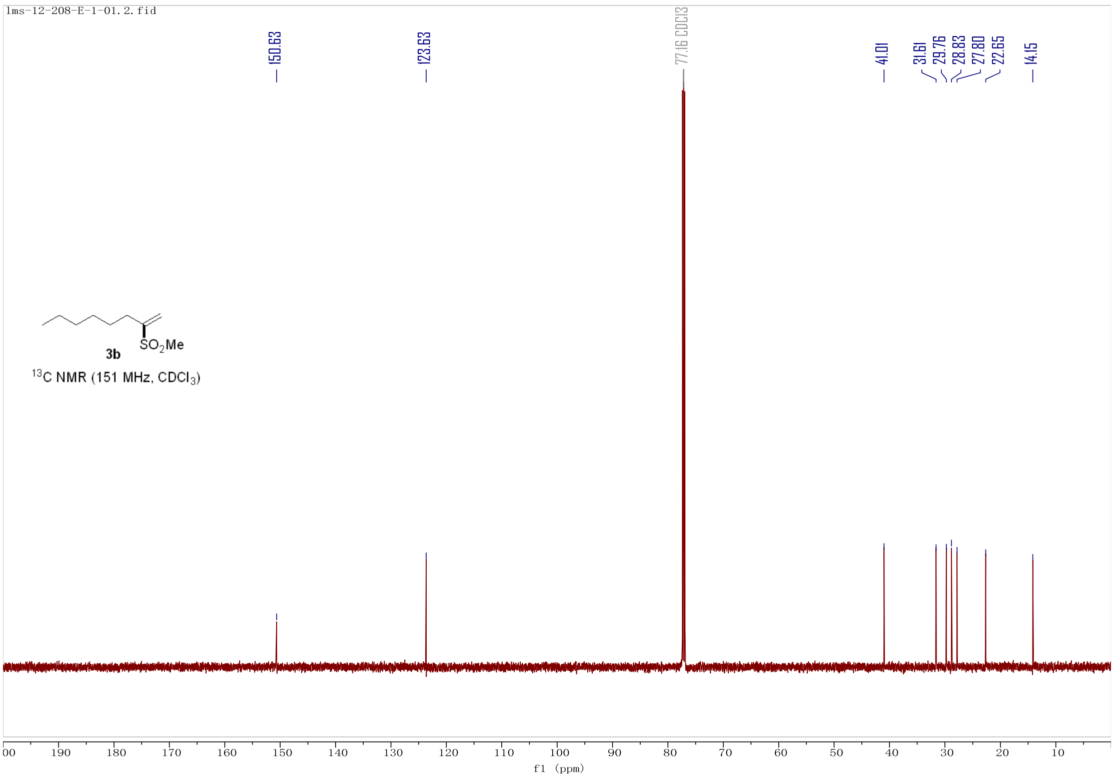
lms-12-208-E-1-01.2.fid
150.63
123.63
77.16 CDCl3
41.01
31.61
29.76
28.83
27.80
22.65
14.15
SO2Me
3b
13C NMR (151 MHz, CDCl3)
f1 (ppm)

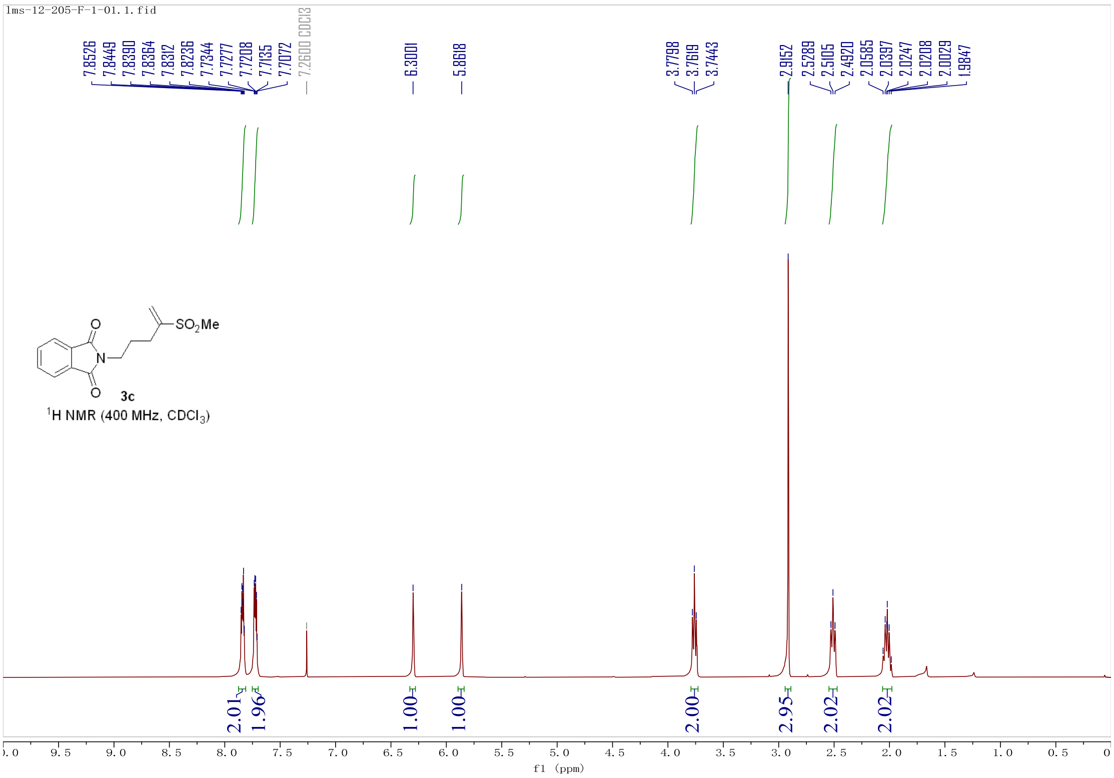
lms-12-205-F-1-01.1.fid
7.8526
7.8449
7.8390
7.8364
7.8312
7.8236
7.7344
7.7277
7.7208
7.7135
7.7072
7.2600 CDCl3
6.3001
5.8618
3.7798
3.7619
3.7443
2.9152
2.5289
2.5105
2.4920
2.0585
2.0397
2.0247
2.0208
2.0029
1.9847
SO2Me
O
N
O
3c
1H NMR (400 MHz, CDCl3)
2.01
1.96
1.00
1.00
2.00
2.95
2.02
2.02
f1 (ppm)

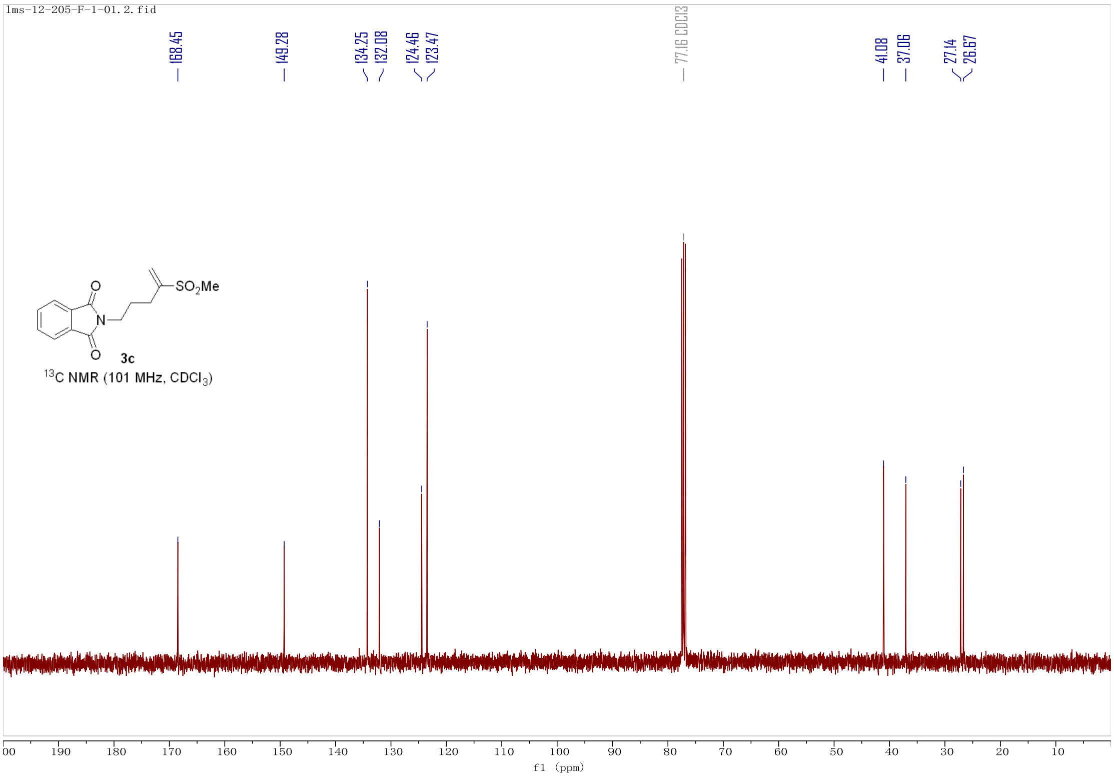
lms-12-205-F-1-01.2.fid
168.45
149.28
134.25
132.08
124.46
123.47
77.16 CDCl3
41.08
37.06
27.14
26.67
3c
13C NMR (101 MHz, CDCl3)
f1 (ppm)

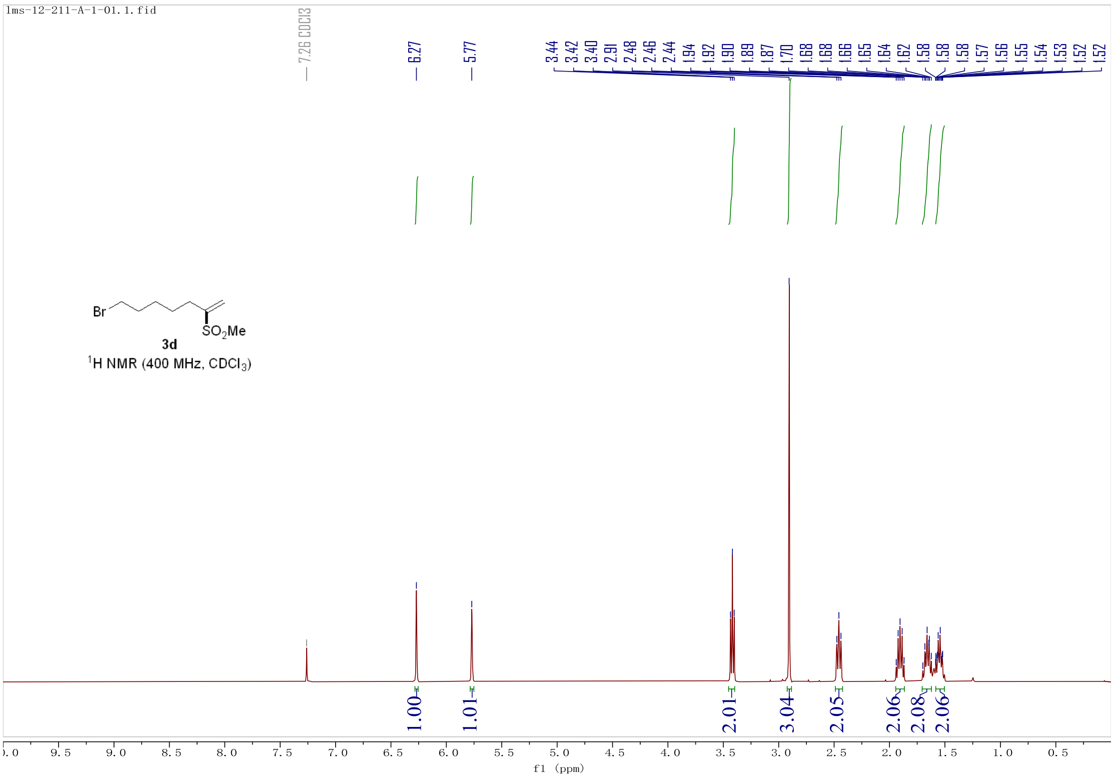
lms-12-211-A-1-01.1.fid
7.26 CDCl3
6.27
5.77
Br
SO2Me
3d
1H NMR (400 MHz, CDCl3)
1.00
1.01
2.01
3.04
2.05
2.06
2.08
2.06
f1 (ppm)

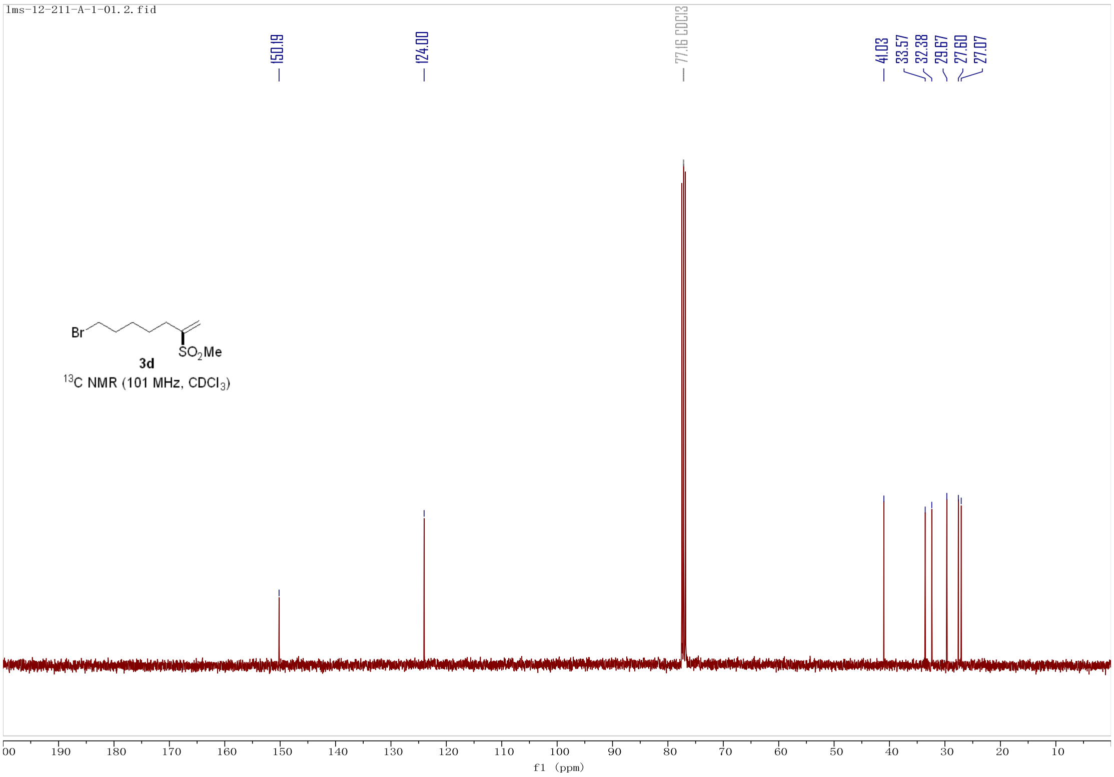
lms-12-211-A-1-01.2.fid
150.19
124.00
77.16 CDCl3
41.03
33.57
32.38
29.67
27.60
27.07
Br
SO2Me
3d
13C NMR (101 MHz, CDCl3)
f1 (ppm)

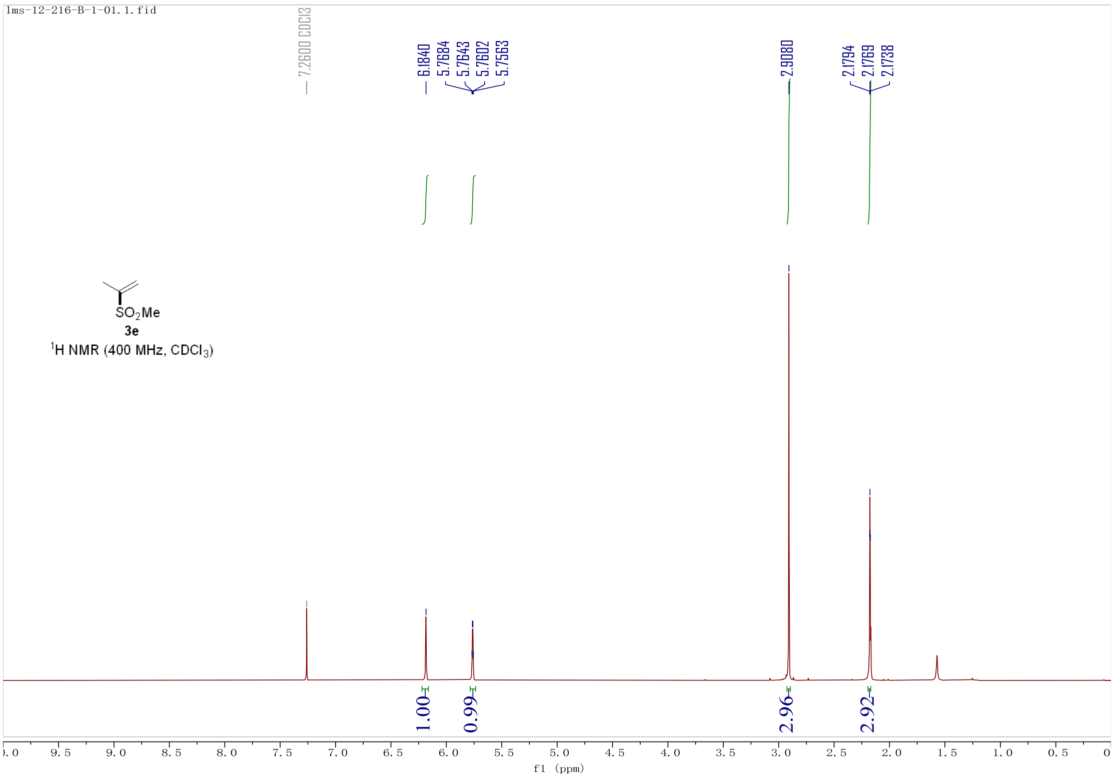
lms-12-216-B-1-01.1.fid
7.2600 CDCl3
6.1840
5.7684
5.7643
5.7602
5.7563
2.9080
2.1794
2.1769
2.1738
SO2Me
3e
1H NMR (400 MHz, CDCl3)
1.00
0.99
2.96
2.92
f1 (ppm)

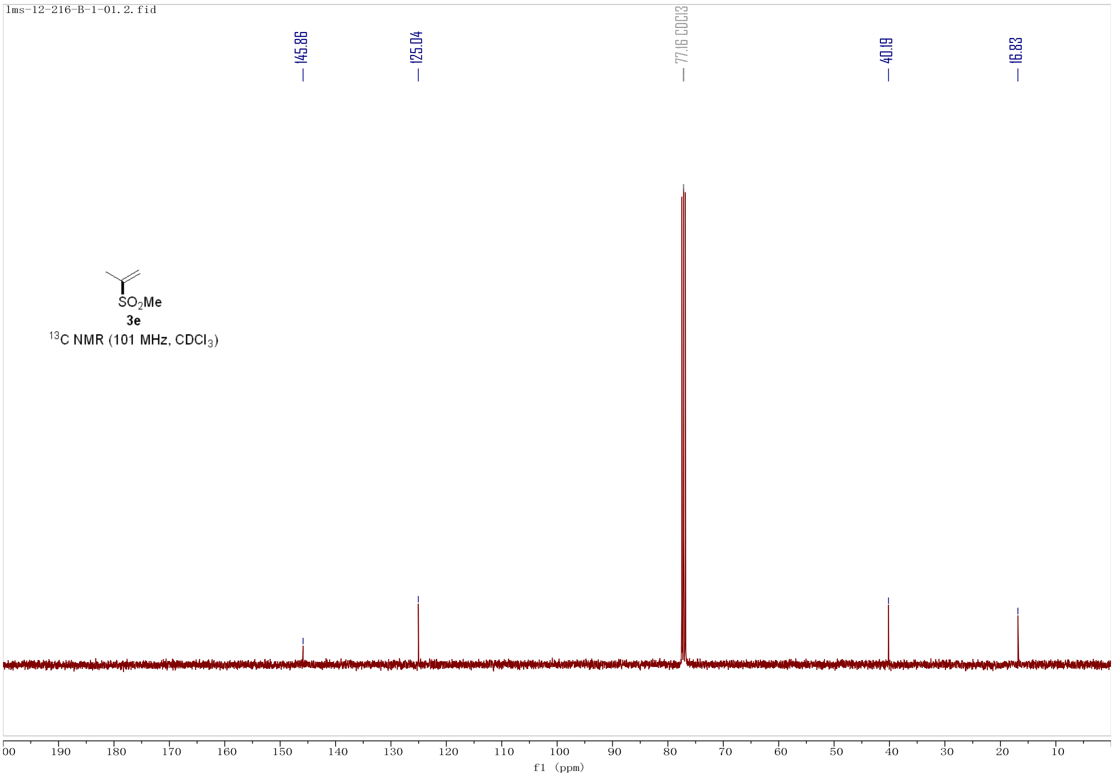
lms-12-216-B-1-01.2.fid
145.86
125.04
77.16 CDCl3
40.19
16.83
SO2Me
3e
13C NMR (101 MHz, CDCl3)
f1 (ppm)

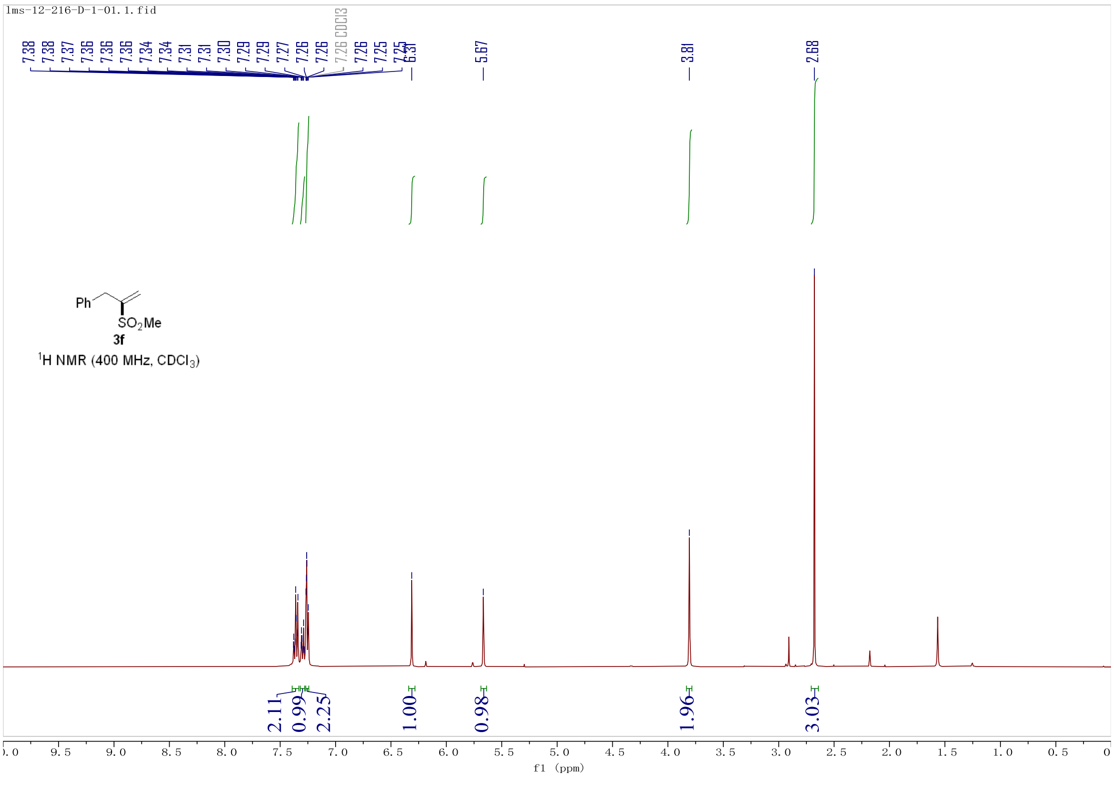
lms-12-216-D-1-01.1.fid
6.31
5.67
3.81
2.68
Ph
SO2Me
3f
1H NMR (400 MHz, CDCl3)
2.11
0.99
2.25
1.00
0.98
1.96
3.03
f1 (ppm)

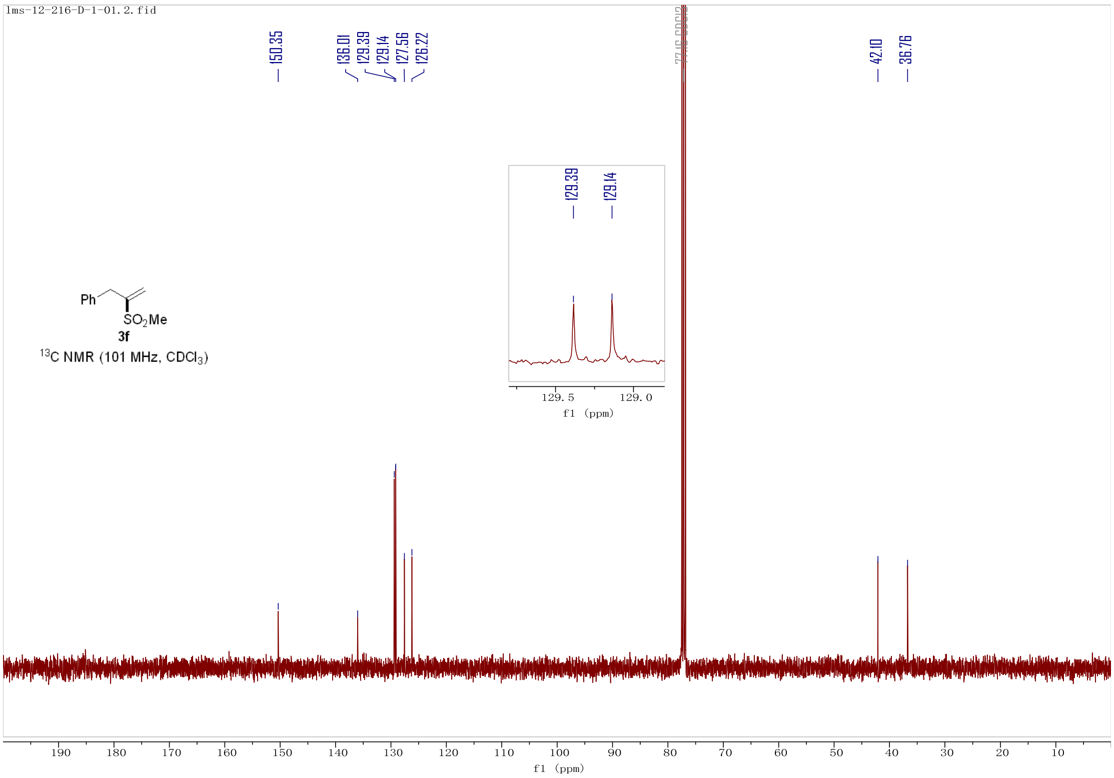
lms-12-216-D-1-01.2.fid
150.35
136.01
129.39
129.14
127.56
126.22
42.10
36.76
129.39
129.14
129.5
129.0
f1 (ppm)
Ph
SO2Me
3f
13C NMR (101 MHz, CDCl3)
190
180
170
160
150
140
130
120
110
100
90
80
70
60
50
40
30
20
10
f1 (ppm)

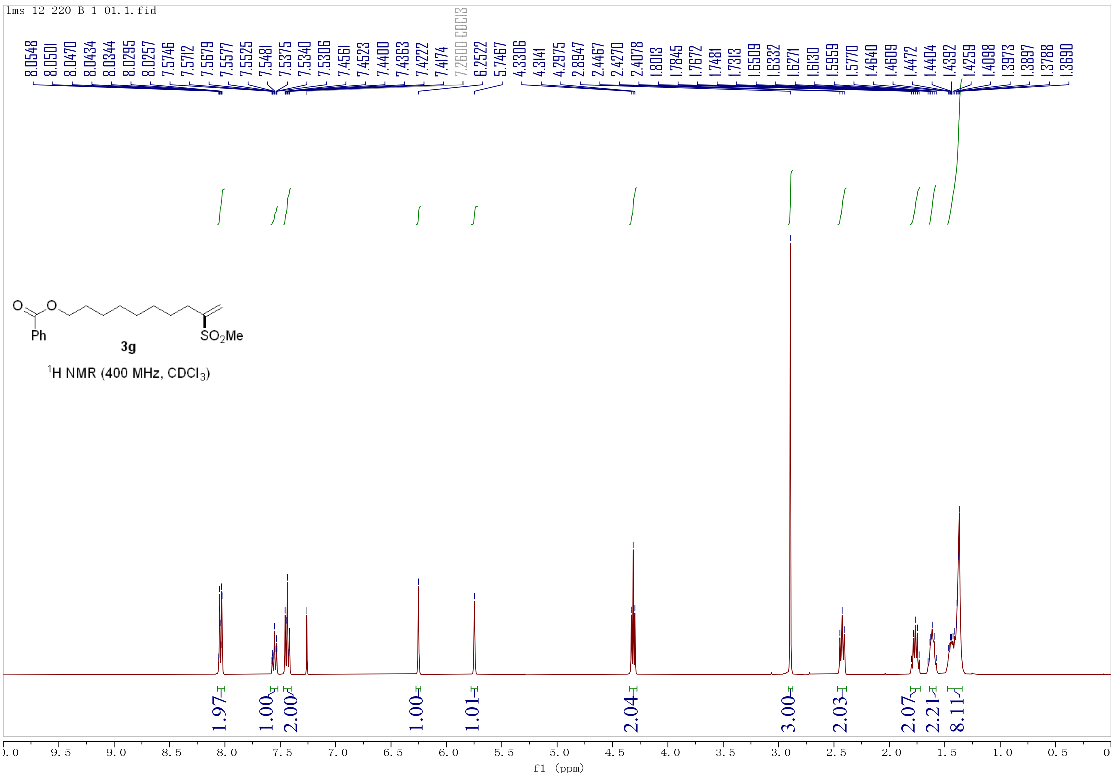
lms-12-220-B-1-01.1.fid
8.0548
8.0501
8.0470
8.0434
8.0344
8.0295
8.0257
7.5746
7.5712
7.5679
7.5577
7.5525
7.5481
7.5375
7.5340
7.5306
7.4561
7.4523
7.4400
7.4363
7.4222
7.4174
7.2600 CDCl3
6.2522
5.7467
4.3306
4.3141
4.2975
2.8947
2.4467
2.4270
2.4078
1.8013
1.7845
1.7672
1.7481
1.7313
1.6509
1.6332
1.6271
1.6130
1.5959
1.5770
1.4640
1.4609
1.4472
1.4404
1.4392
1.4259
1.4098
1.3973
1.3897
1.3788
1.3690
O
O
Ph
3g
SO2Me
1H NMR (400 MHz, CDCl3)
1.97
1.00
2.00
1.00
1.01
2.04
3.00
2.03
2.07
2.21
8.11
9.5
9.0
8.5
8.0
7.5
7.0
6.5
6.0
5.5
5.0
4.5
4.0
3.5
3.0
2.5
2.0
1.5
1.0
0.5
0
f1 (ppm)

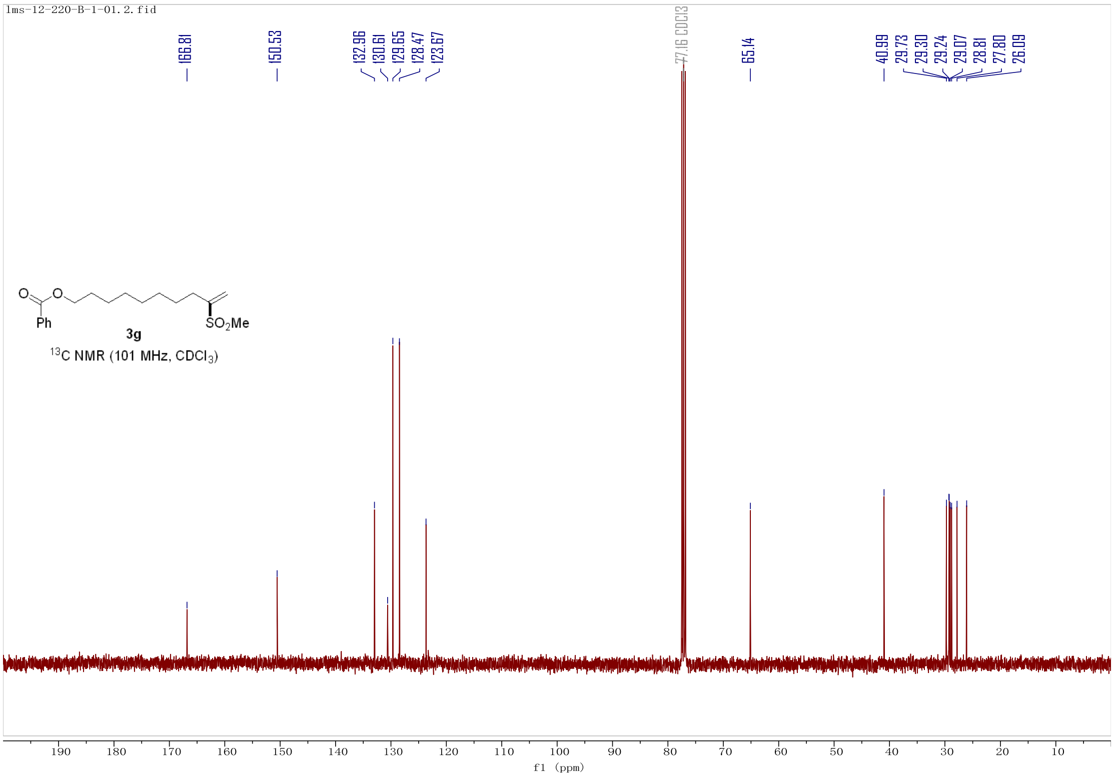
lms-12-220-B-1-01.2.fid
166.81
150.53
132.96
130.61
129.65
128.47
123.67
77.16 CDCl3
65.14
40.99
29.73
29.30
29.24
29.07
28.81
27.80
26.09
Ph
SO2Me
3g
13C NMR (101 MHz, CDCl3)
f1 (ppm)

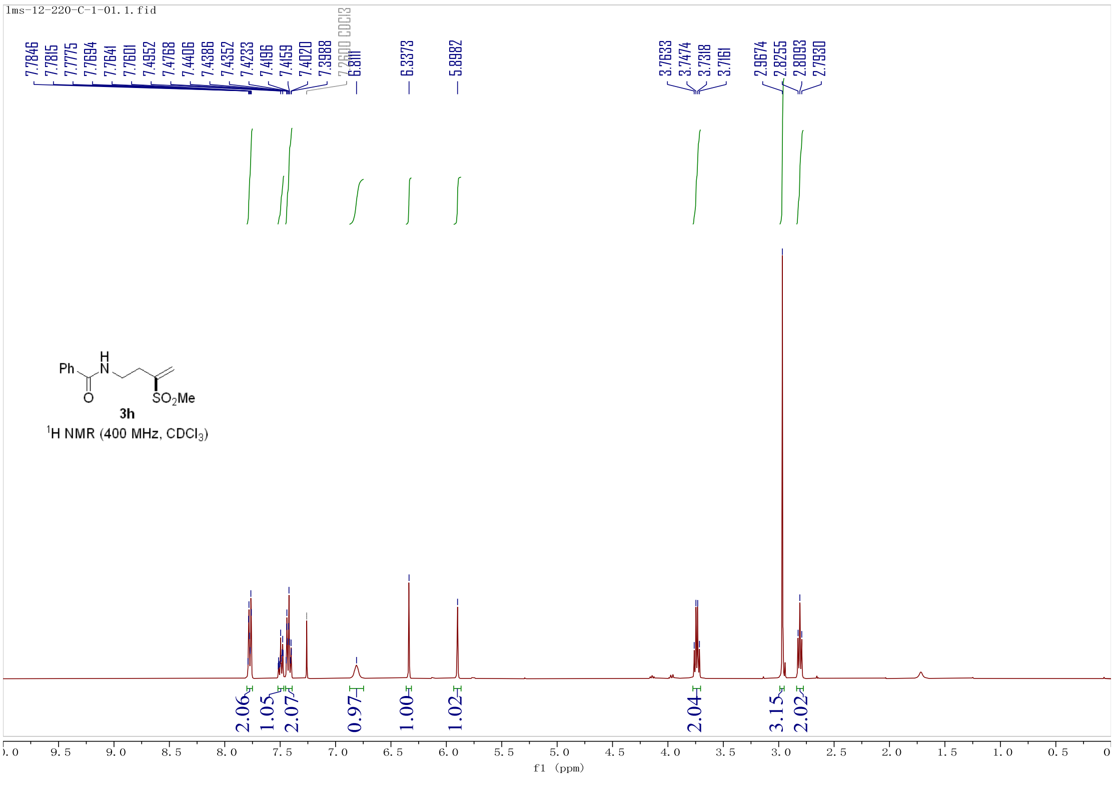
lms-12-220-C-1-01.1.fid
7.7846
7.7815
7.7775
7.7694
7.7641
7.7601
7.4952
7.4768
7.4406
7.4386
7.4352
7.4233
7.4196
7.4159
7.4020
7.3988
6.8111
6.3373
5.8982
3.7633
3.7474
3.7318
3.7161
2.9674
2.8255
2.8093
2.7930
Ph
H
N
O
SO2Me
3h
1H NMR (400 MHz, CDCl3)
2.06
1.05
2.07
0.97
1.00
1.02
2.04
3.15
2.02
f1 (ppm)

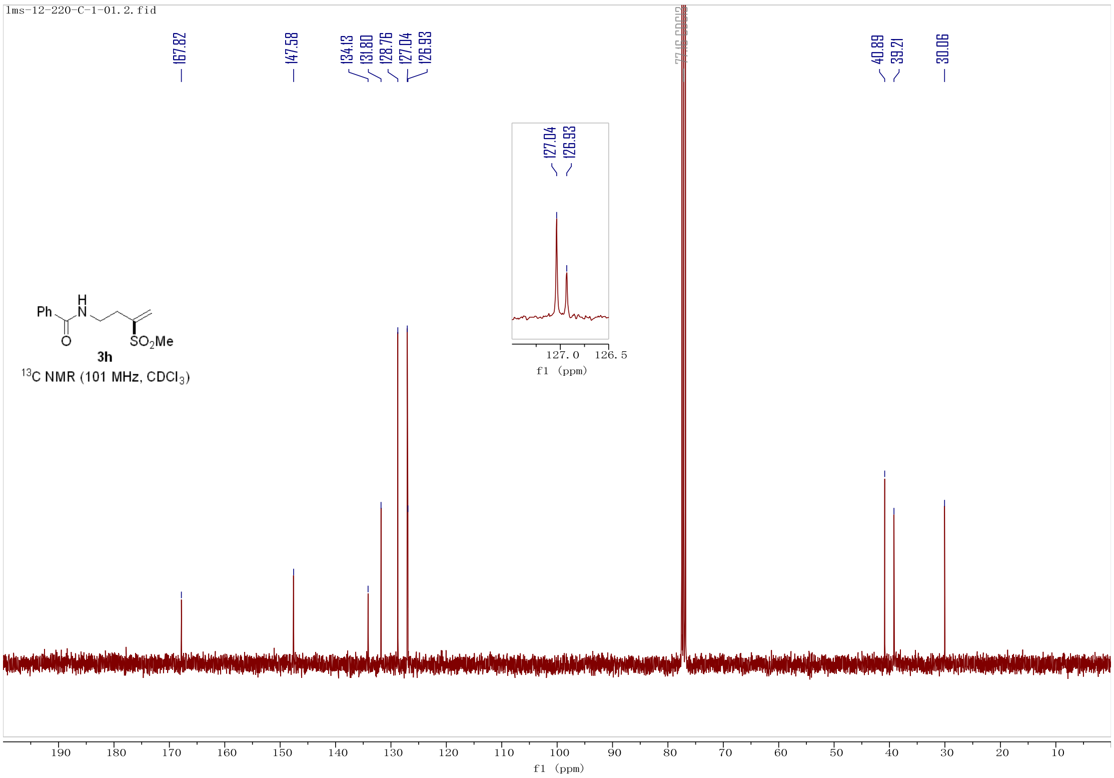
lms-12-220-C-1-01.2.fid
167.82
147.58
134.13
131.80
128.76
127.04
126.93
40.89
39.21
30.06
127.04
126.93
127.0
126.5
f1 (ppm)
Ph
H
N
O
SO2Me
3h
13C NMR (101 MHz, CDCl3)
190
180
170
160
150
140
130
120
110
100
90
80
70
60
50
40
30
20
10
f1 (ppm)

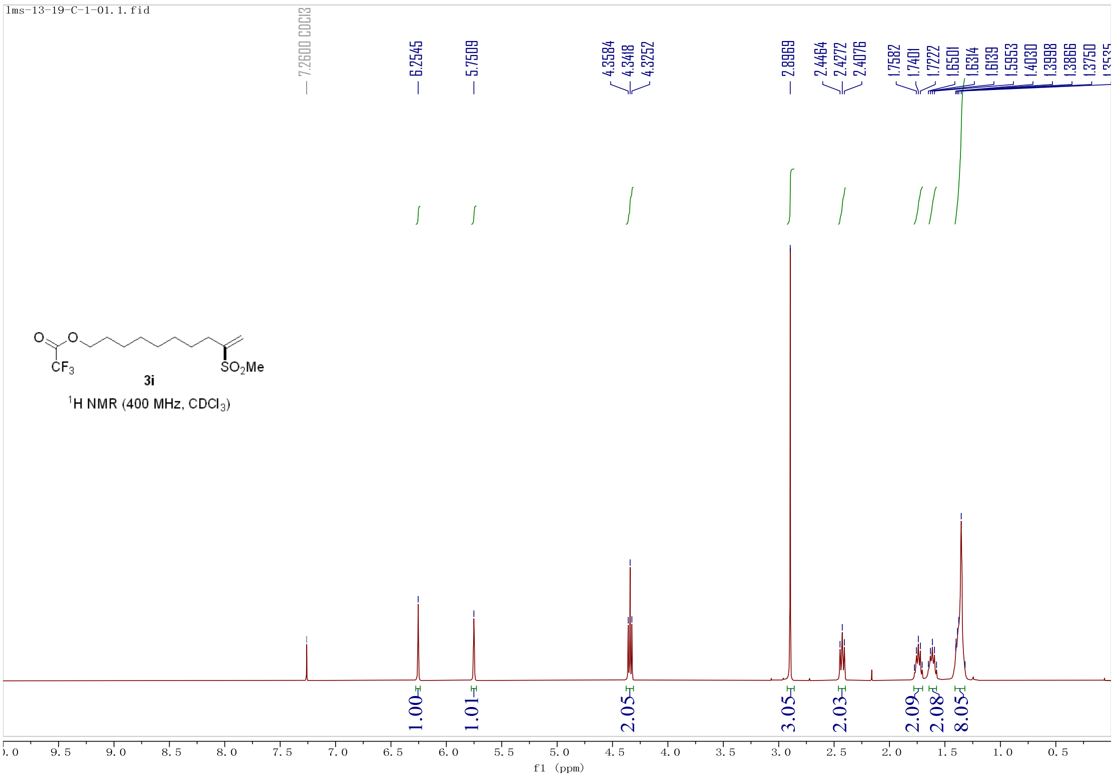
lms-13-19-C-1-01.1.fid
7.2600 CDCl3
6.2545
5.7509
4.3584
4.3418
4.3252
2.8969
2.4464
2.4272
2.4076
1.7582
1.7401
1.7222
1.6501
1.6314
1.6139
1.5953
1.4030
1.3998
1.3866
1.3750
O
O
CF3
SO2Me
3i
1H NMR (400 MHz, CDCl3)
1.00
1.01
2.05
3.05
2.03
2.09
2.08
8.05
9.5
9.0
8.5
8.0
7.5
7.0
6.5
6.0
5.5
5.0
4.5
4.0
3.5
3.0
2.5
2.0
1.5
1.0
0.5
f1 (ppm)

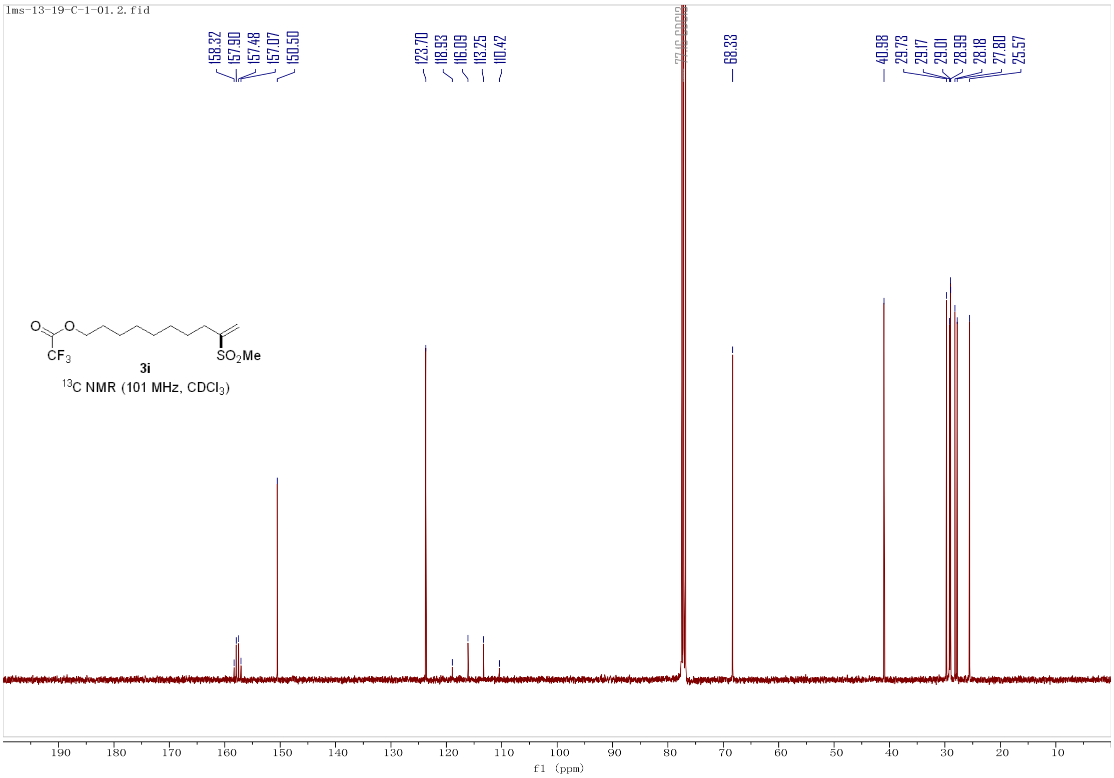
lms-13-19-C-1-01.2.fid
158.32
157.90
157.48
157.07
150.50
123.70
118.93
116.09
113.25
110.42
68.33
40.98
29.73
29.17
29.01
28.99
28.18
27.80
25.57
O
O
CF3
SO2Me
3i
13C NMR (101 MHz, CDCl3)
190
180
170
160
150
140
130
120
110
100
90
80
70
60
50
40
30
20
10
f1 (ppm)

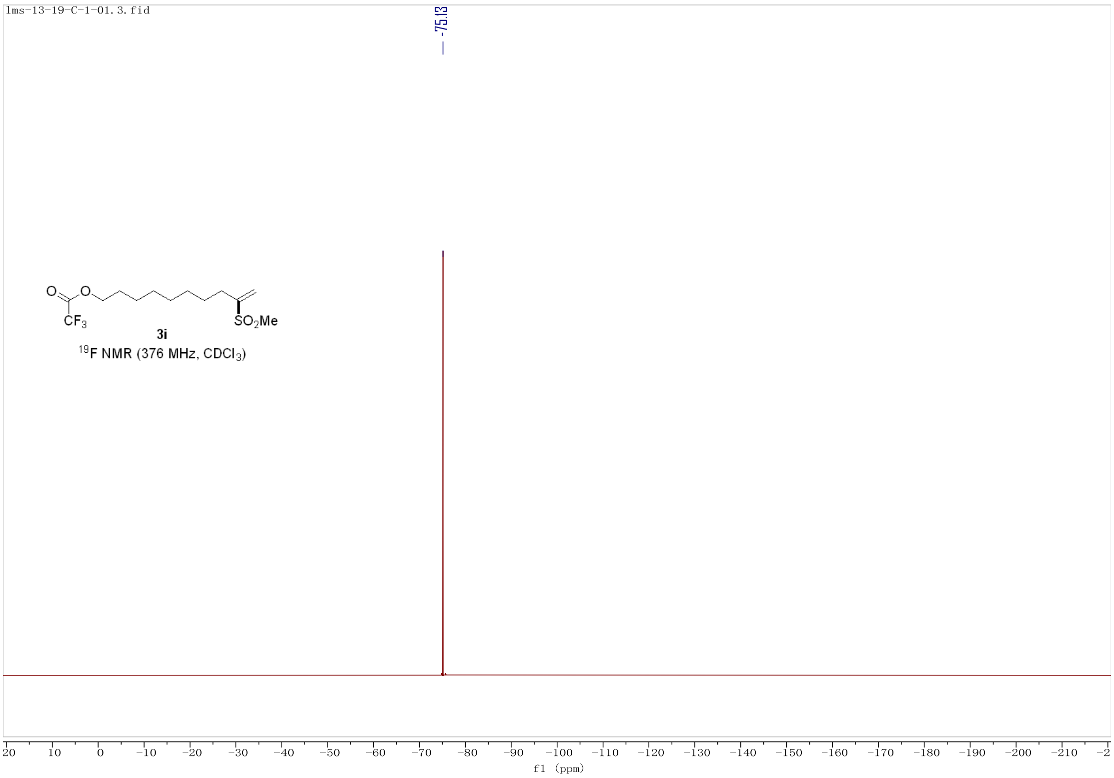
lms-13-19-C-1-01.3.fid
-75.13
O
O
CF3
SO2Me
3i
19F NMR (376 MHz, CDCl3)
20
10
0
−10
−20
−30
−40
−50
−60
−70
−80
−90
−100
−110
−120
−130
−140
−150
−160
−170
−180
−190
−200
−210
f1 (ppm)

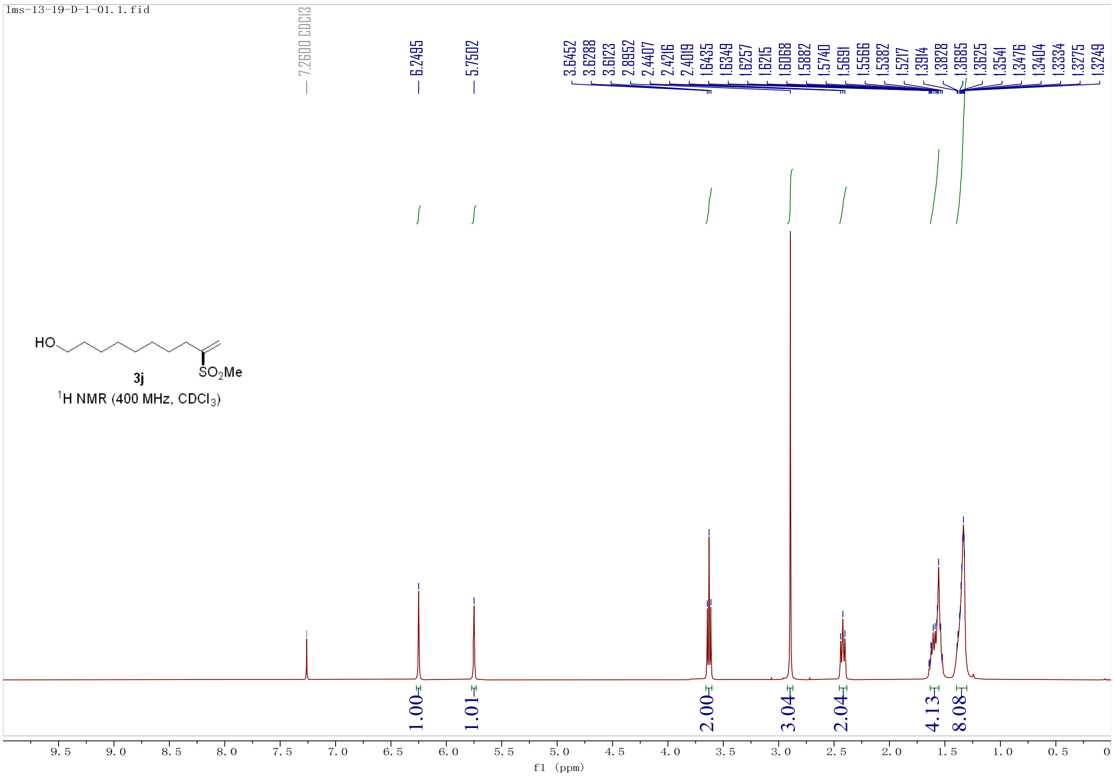
lms-13-19-D-1-01.1.fid
7.2600 CDCl3
6.2495
5.7502
3.6452
3.6288
3.6123
2.8952
2.4407
2.4216
2.4019
1.6435
1.6349
1.6257
1.6215
1.6068
1.5882
1.5740
1.5691
1.5566
1.5382
1.5217
1.3914
1.3828
1.3685
1.3625
1.3541
1.3476
1.3404
1.3334
1.3275
1.3249
HO
3j
SO2Me
1H NMR (400 MHz, CDCl3)
1.00
1.01
2.00
3.04
2.04
4.13
8.08
f1 (ppm)

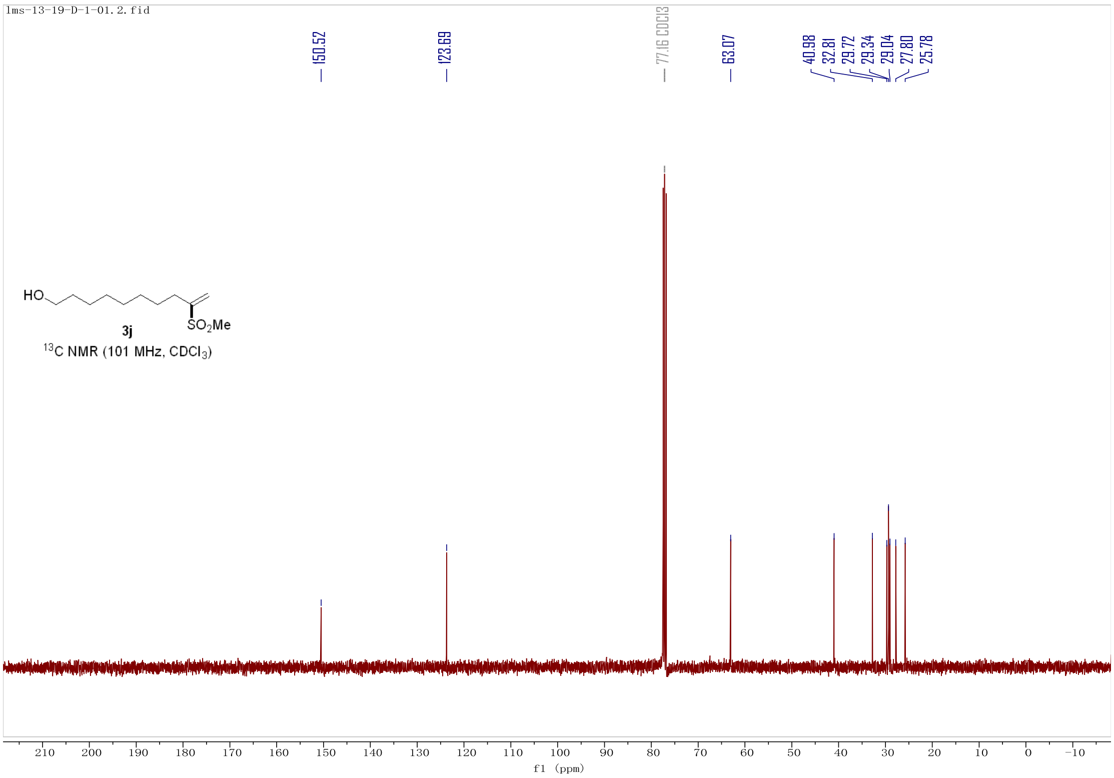
lms-13-19-D-1-01.2.fid
150.52
123.69
77.16 CDCl3
63.07
40.98
32.81
29.72
29.34
29.04
27.80
25.78
HO
3j
SO2Me
13C NMR (101 MHz, CDCl3)
f1 (ppm)

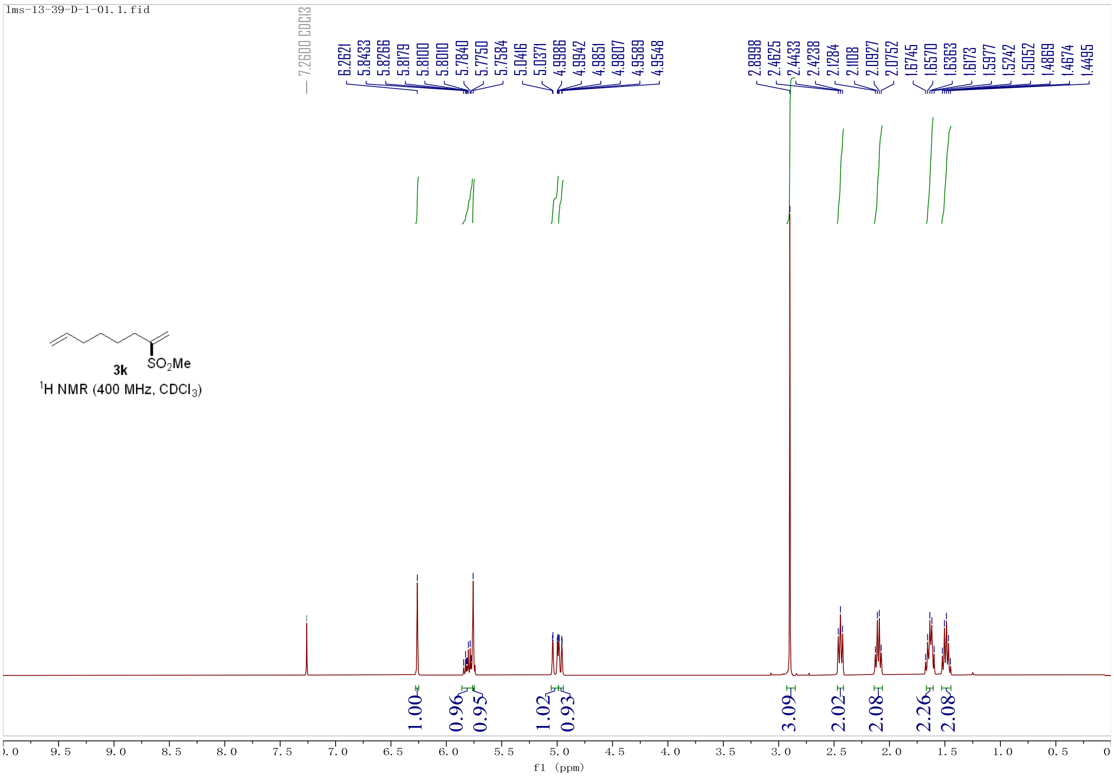
lms-13-39-D-1-01.1.fid
7.2600 CDCl3
6.2621
5.8433
5.8266
5.8179
5.8100
5.8010
5.7840
5.7750
5.7584
5.0416
5.0371
4.9986
4.9942
4.9851
4.9807
4.9589
4.9548
2.8998
2.4625
2.4433
2.4238
2.1284
2.1108
2.0927
2.0752
1.6745
1.6570
1.6363
1.6173
1.5977
1.5242
1.5052
1.4869
1.4674
1.4495
3k
SO2Me
1H NMR (400 MHz, CDCl3)
1.00
0.96
0.95
1.02
0.93
3.09
2.02
2.08
2.26
2.08
f1 (ppm)

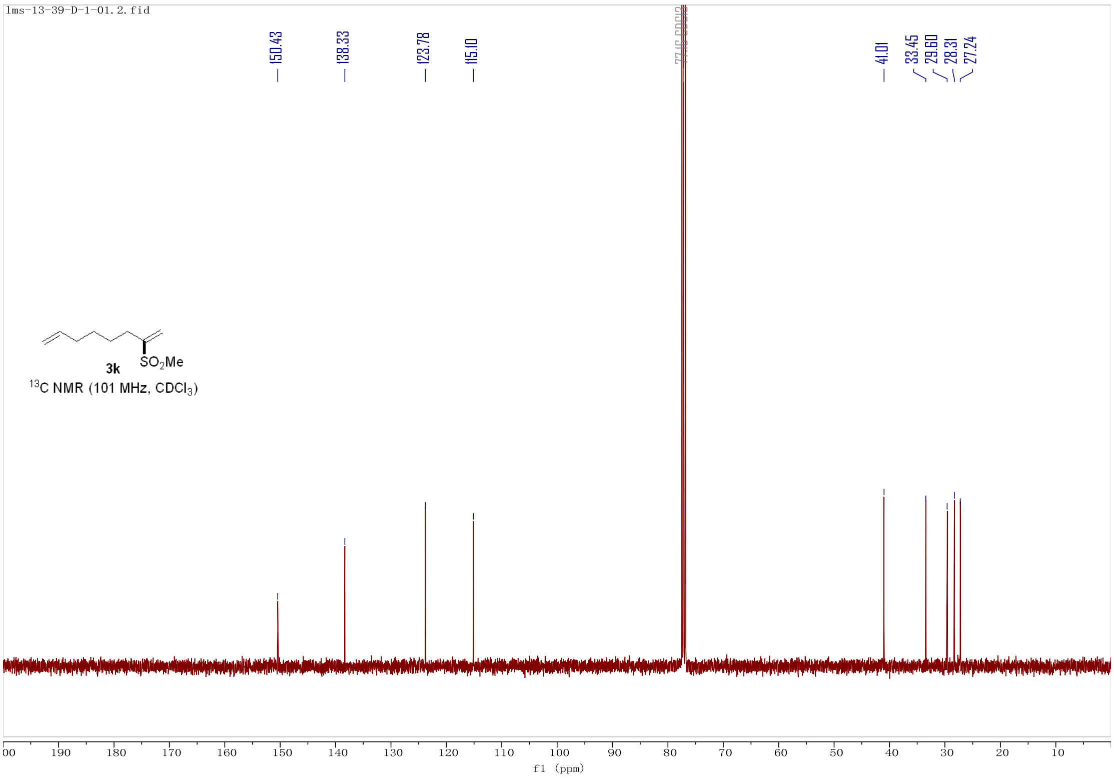
lms-13-39-D-1-01.2.fid
150.43
138.33
123.78
115.10
41.01
33.45
29.60
28.31
27.24
3k
SO2Me
13C NMR (101 MHz, CDCl3)
f1 (ppm)

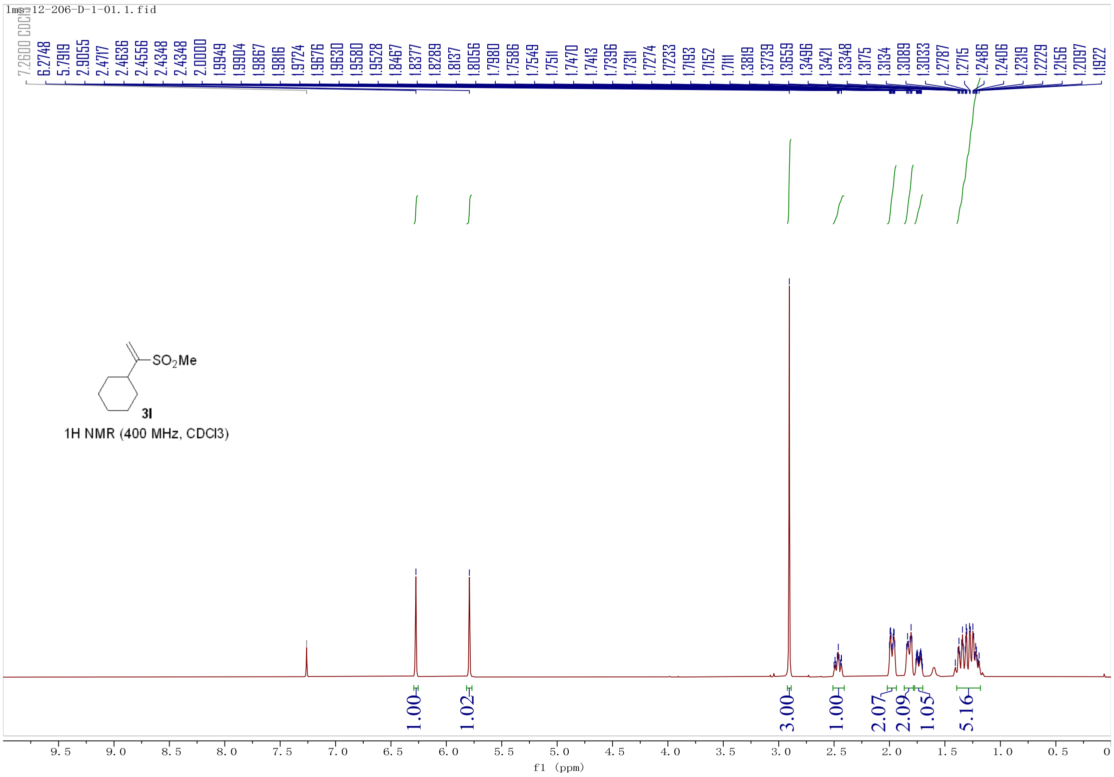
lms-12-206-D-1-01.1.fid
7.2600 CDCl3
6.2748
5.7919
2.9055
2.4717
2.4636
2.4556
2.4348
2.4348
2.0000
1.9949
1.9904
1.9867
1.9816
1.9724
1.9676
1.9630
1.9580
1.9528
1.8467
1.8377
1.8289
1.8137
1.8056
1.7980
1.7586
1.7549
1.7511
1.7470
1.7413
1.7396
1.7311
1.7274
1.7233
1.7193
1.7152
1.7111
1.3819
1.3739
1.3659
1.3496
1.3421
1.3348
1.3175
1.3134
1.3089
1.3033
1.2787
1.2715
1.2486
1.2406
1.2319
1.2229
1.2156
1.2097
1.1922
SO2Me
3l
1H NMR (400 MHz, CDCl3)
1.00
1.02
3.00
1.00
2.07
2.09
1.05
5.16
f1 (ppm)

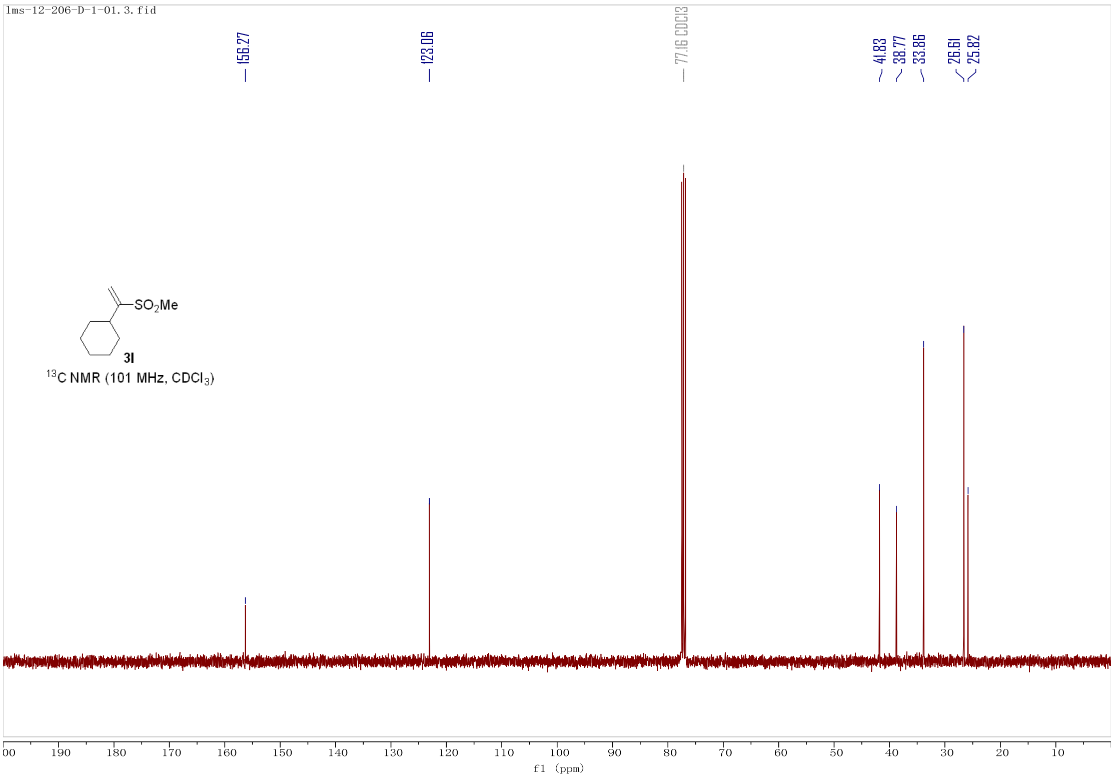
lms-12-206-D-1-01.3.fid
156.27
123.06
77.16 CDCl3
41.83
38.77
33.86
26.61
25.82
SO2Me
3l
13C NMR (101 MHz, CDCl3)
f1 (ppm)

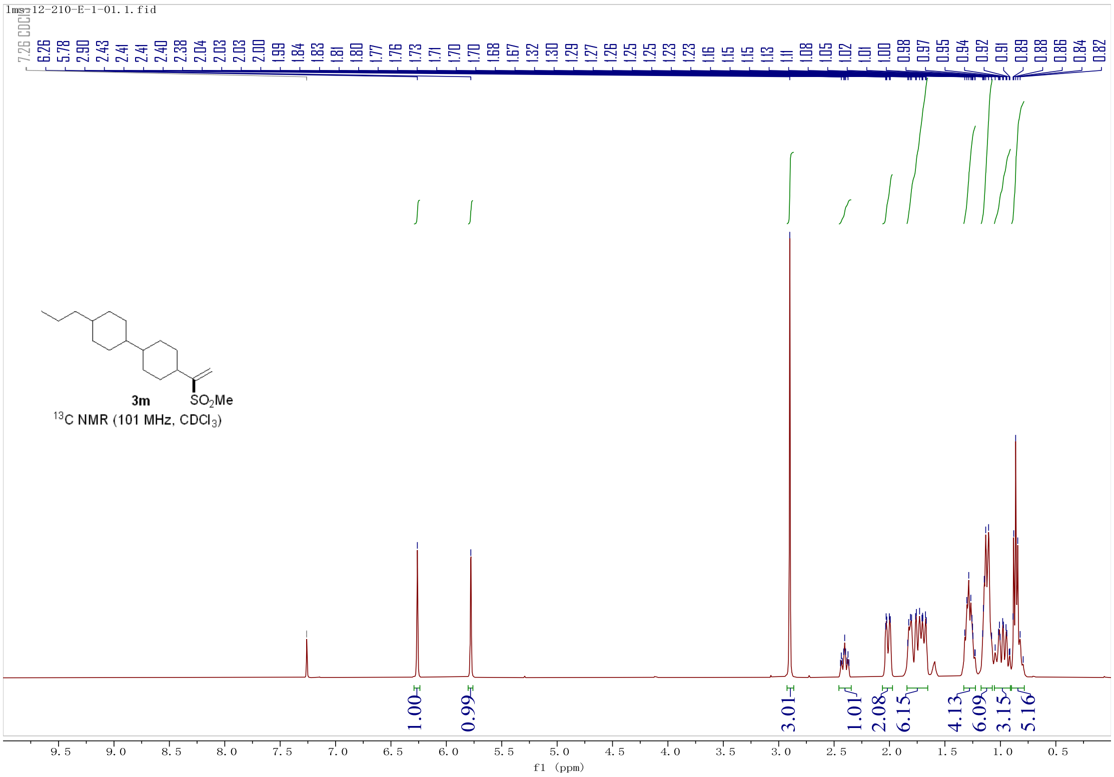
lms-12-210-E-1-01.1.fid
7.26 CDCl3
6.26
5.78
2.90
2.43
2.41
2.41
2.40
2.38
2.04
2.03
2.03
2.00
1.99
1.84
1.83
1.81
1.80
1.77
1.76
1.73
1.71
1.70
1.70
1.68
1.67
1.32
1.30
1.29
1.27
1.26
1.25
1.25
1.23
1.23
1.16
1.15
1.15
1.13
1.11
1.08
1.05
1.02
1.01
1.00
0.98
0.97
0.95
0.94
0.92
0.91
0.89
0.88
0.86
0.84
0.82
3m
SO2Me
13C NMR (101 MHz, CDCl3)
1.00
0.99
3.01
1.01
2.08
6.15
4.13
6.09
3.15
5.16
f1 (ppm)

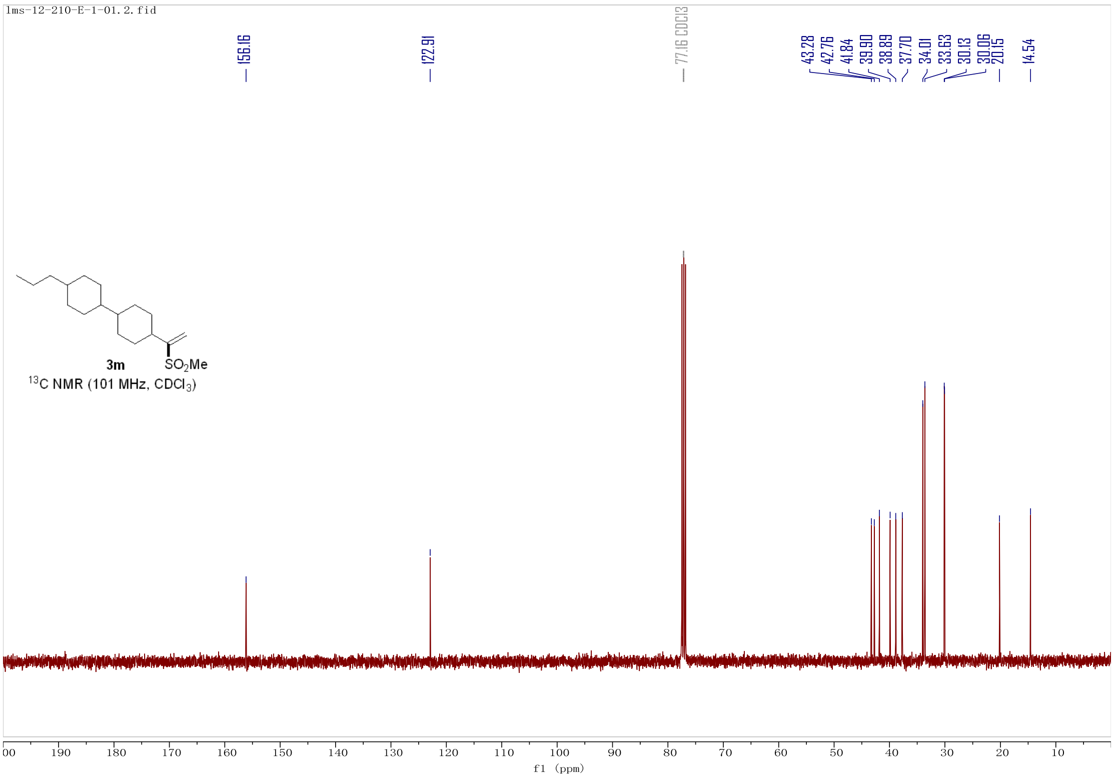
lms-12-210-E-1-01.2.fid
156.16
122.91
77.16 CDCl3
43.28
42.76
41.84
39.90
38.89
37.70
34.01
33.63
30.13
30.06
20.15
14.54
3m
SO2Me
13C NMR (101 MHz, CDCl3)
f1 (ppm)

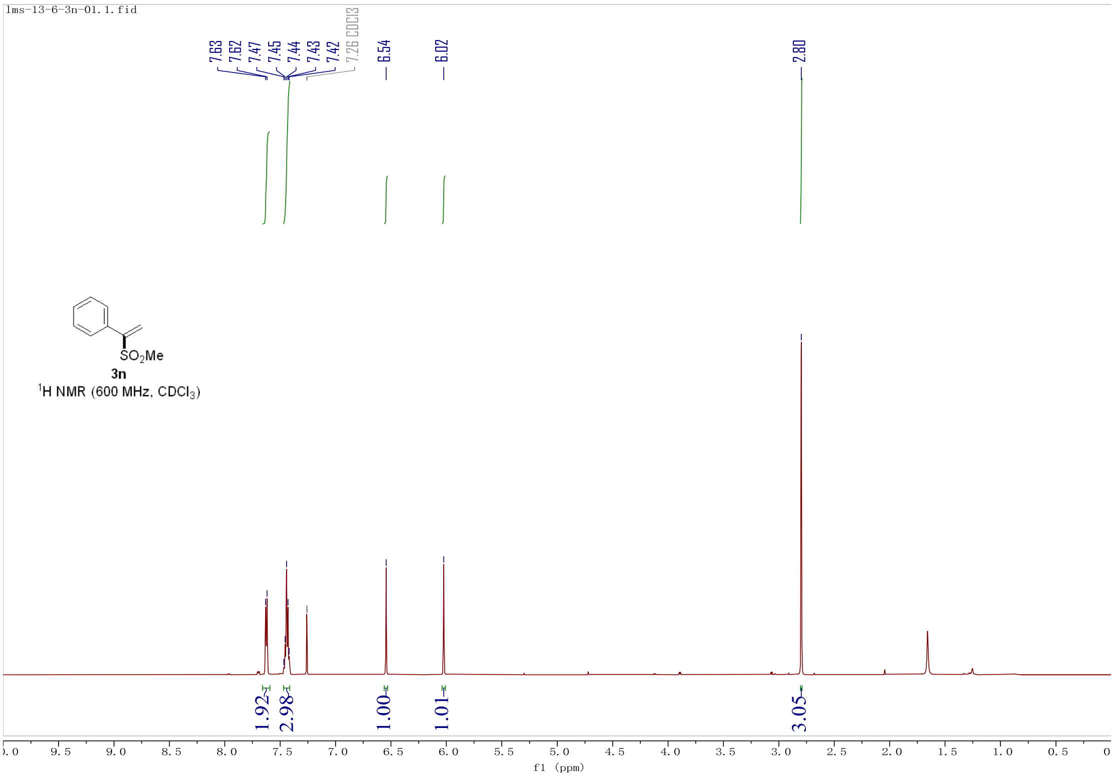
lms-13-6-3n-01.1.fid
7.63
7.62
7.47
7.45
7.44
7.43
7.42
7.26 CDCl3
6.54
6.02
2.80
SO2Me
3n
1H NMR (600 MHz, CDCl3)
1.92
2.98
1.00
1.01
3.05
f1 (ppm)

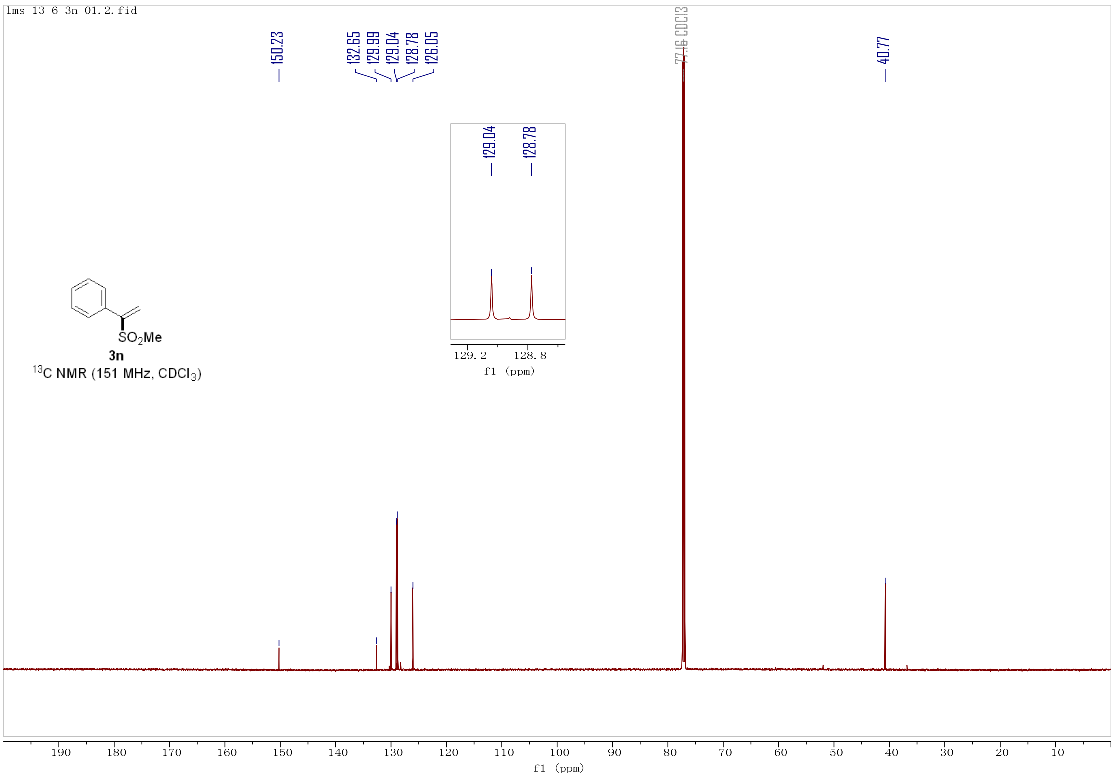
lms-13-6-3n-01.2.fid
150.23
132.65
129.99
129.04
128.78
126.05
77.16 CDCl3
40.77
129.04
128.78
129.2
128.8
f1 (ppm)
SO2Me
3n
13C NMR (151 MHz, CDCl3)
f1 (ppm)

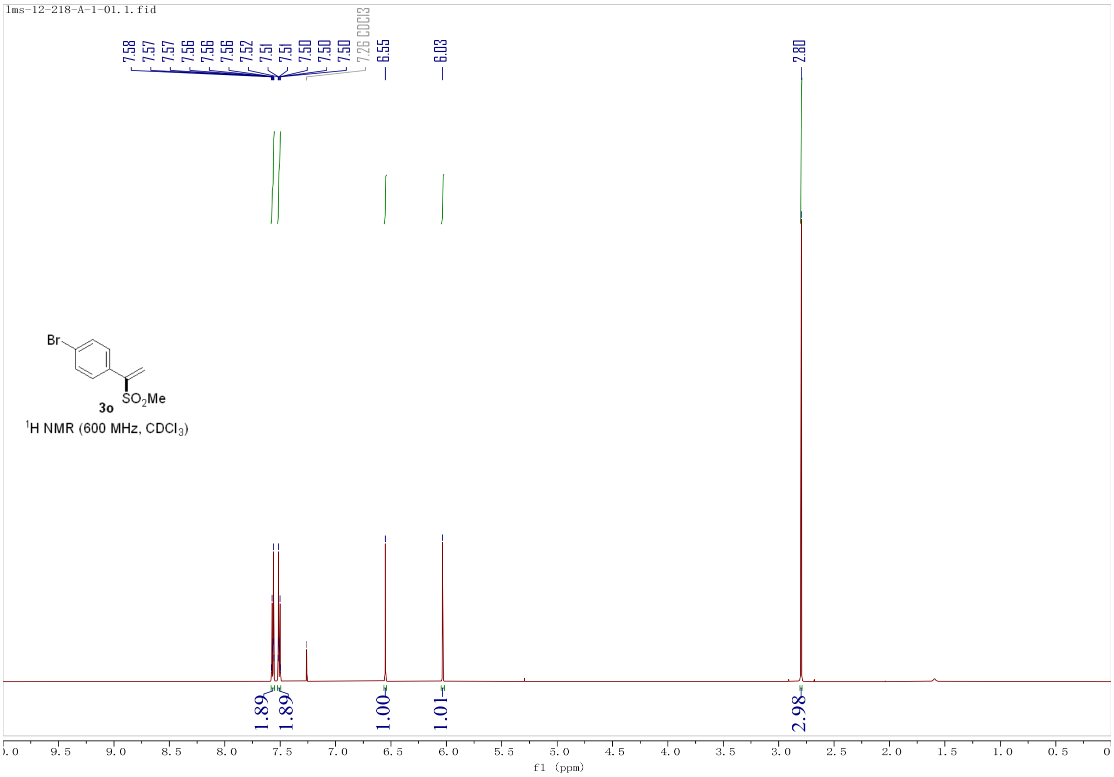
lms-12-218-A-1-01.1.fid
7.58 7.57 7.57 7.56 7.56 7.56 7.52 7.51 7.51 7.50 7.50 7.50
7.26 CDCl3
6.55
6.03
2.80
Br
3o
SO2Me
1H NMR (600 MHz, CDCl3)
1.89
1.89
1.00
1.01
2.98
f1 (ppm)

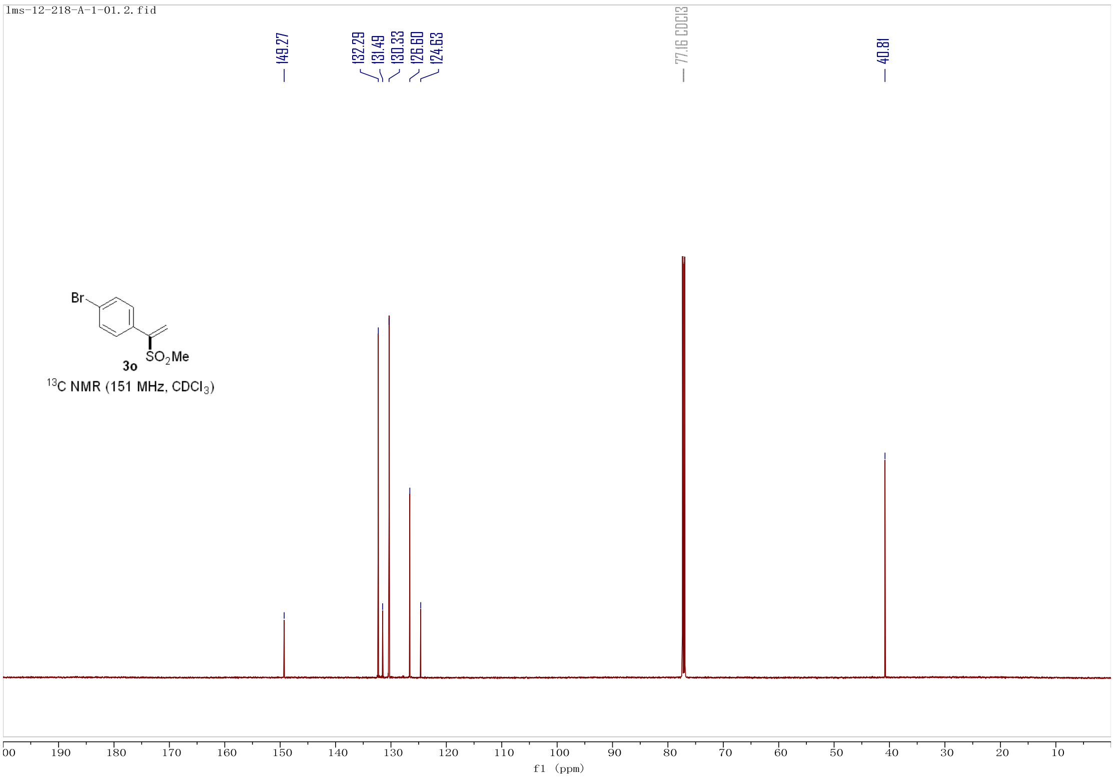
lms-12-218-A-1-01.2.fid
149.27
132.29 131.49 130.33 126.60 124.63
77.16 CDCl3
40.81
Br
3o
SO2Me
13C NMR (151 MHz, CDCl3)
f1 (ppm)

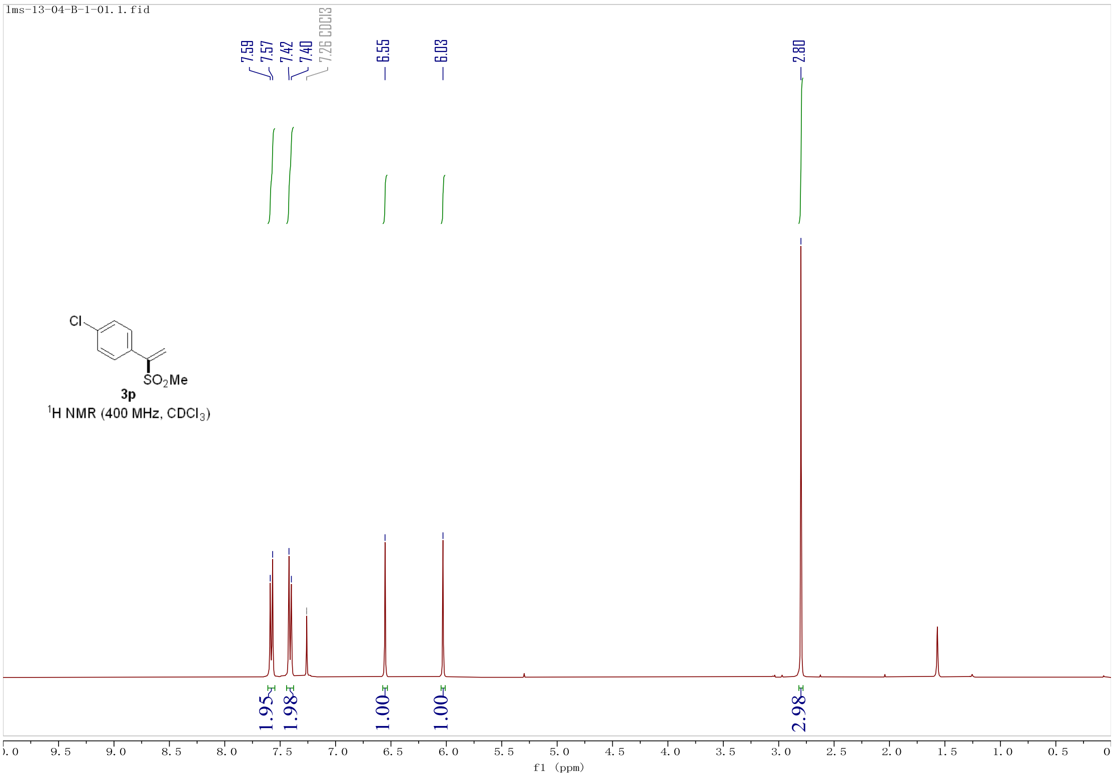
lms-13-04-B-1-01.1.fid
7.59
7.57
7.42
7.40
7.26 CDCl3
6.55
6.03
2.80
Cl
SO2Me
3p
1H NMR (400 MHz, CDCl3)
1.95
1.98
1.00
1.00
2.98
f1 (ppm)

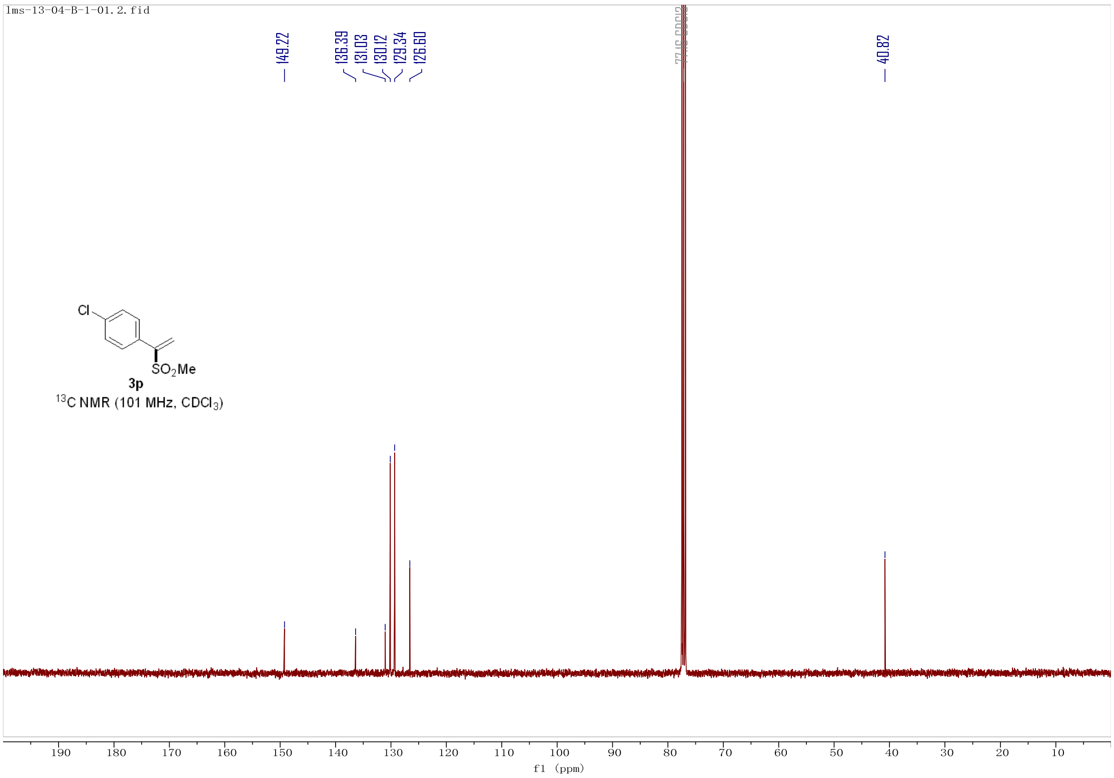
lms-13-04-B-1-01.2.fid
149.22
136.39
131.03
130.12
129.34
126.60
40.82
Cl
SO2Me
3p
13C NMR (101 MHz, CDCl3)
f1 (ppm)

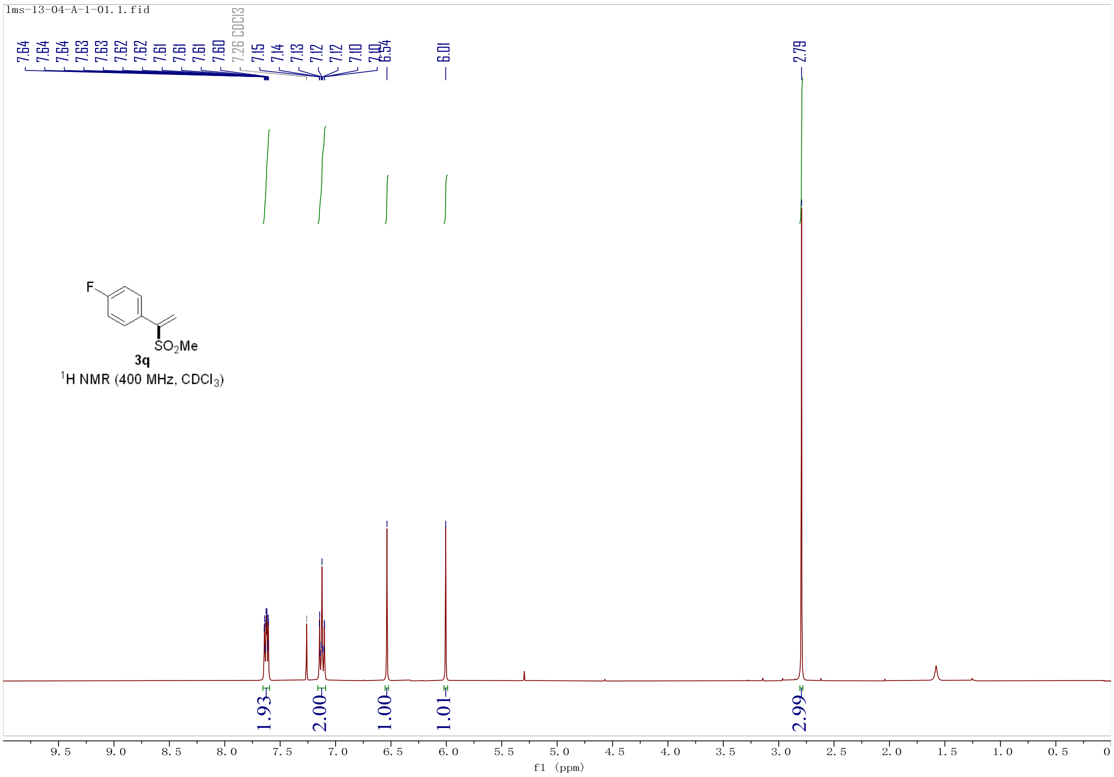
lms-13-04-A-1-01.1.fid
7.64
7.64
7.64
7.63
7.63
7.62
7.62
7.61
7.61
7.61
7.60
7.26 CDCl3
7.15
7.14
7.13
7.12
7.12
7.10
7.10
6.54
6.01
2.79
3q
^{1}H NMR (400 MHz, $CDCl_3$)
1.93
2.00
1.00
1.01
2.99
f1 (ppm)

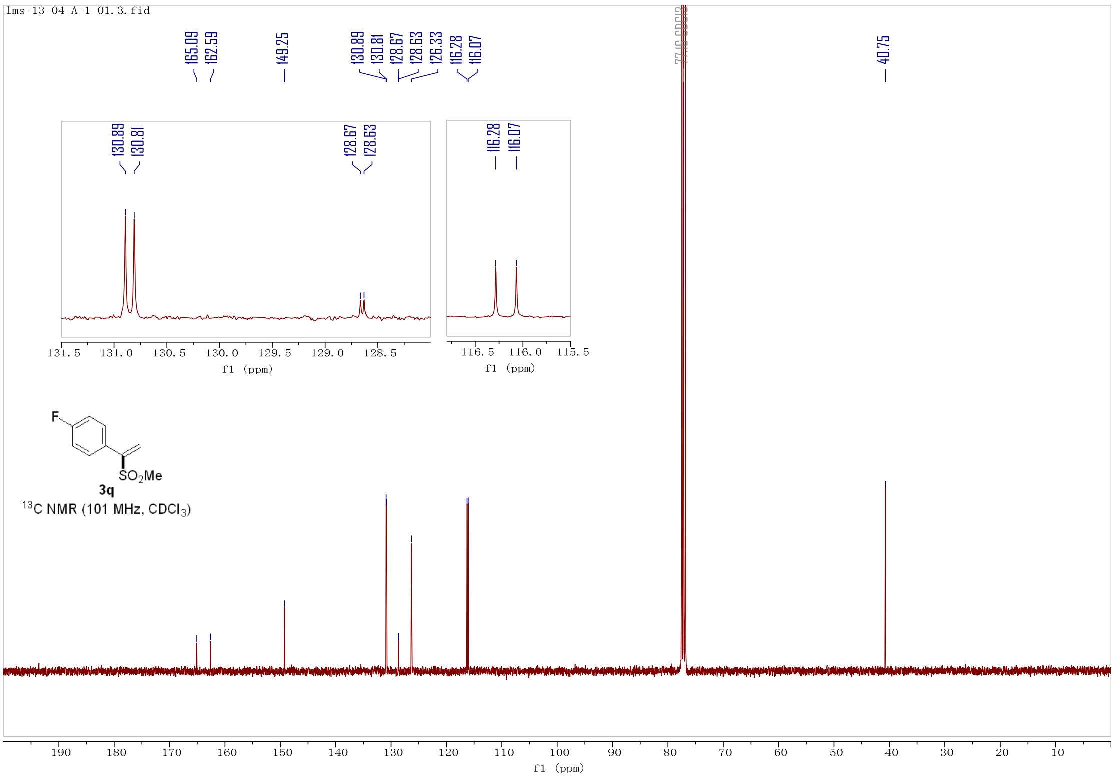
lms-13-04-A-1-01.3.fid
165.09
162.59
149.25
130.89
130.81
128.67
128.63
126.33
116.28
116.07
40.75
f1 (ppm)
3q
^{13}C NMR (101 MHz, $CDCl_3$)

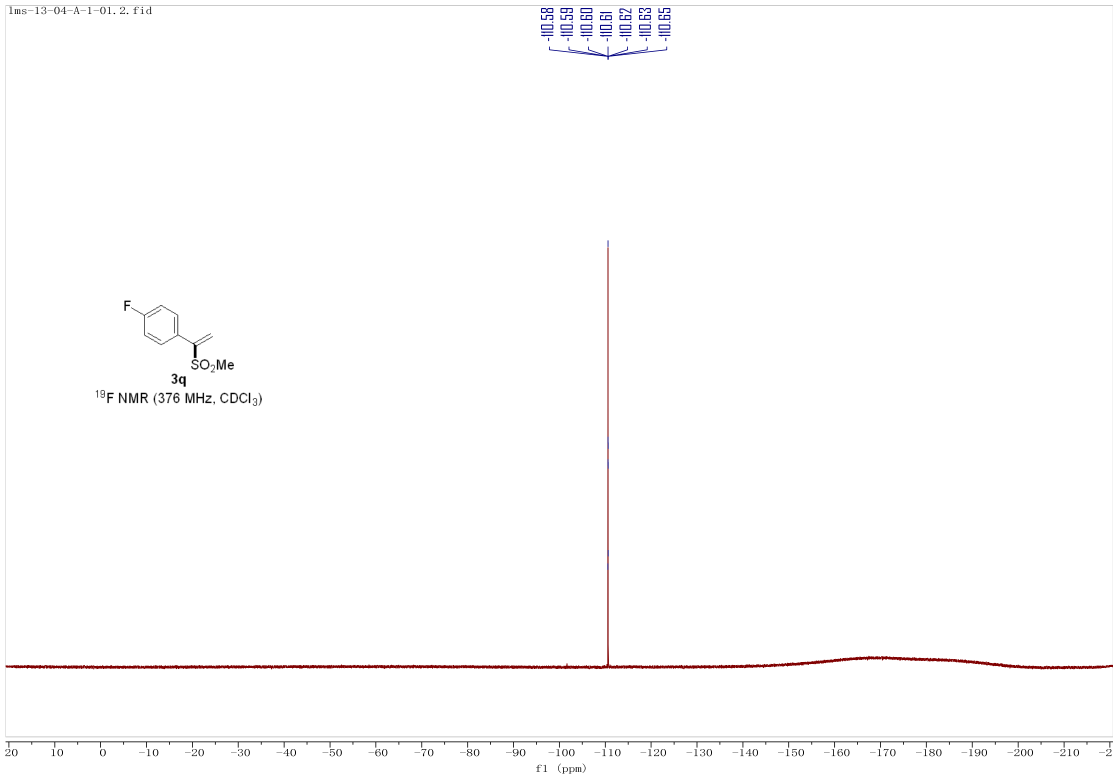
lms-13-04-A-1-01.2.fid
-110.58
-110.59
-110.60
-110.61
-110.62
-110.63
-110.65
F
SO2Me
3q
19F NMR (376 MHz, CDCl3)
f1 (ppm)

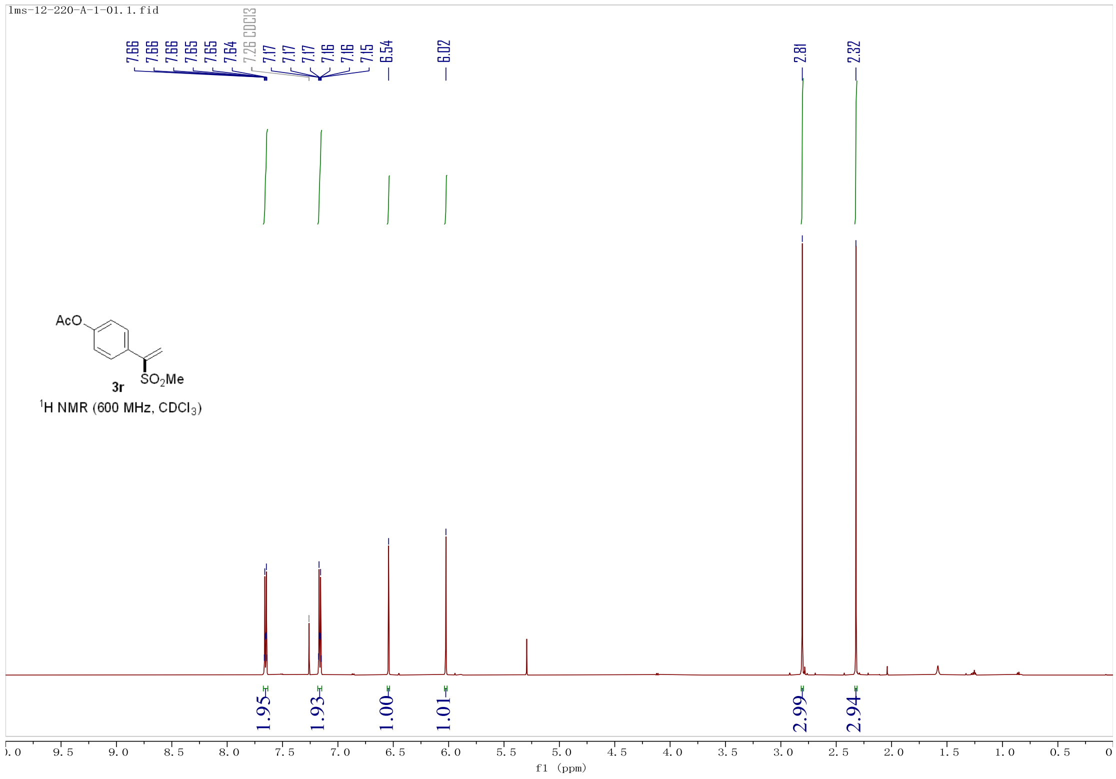
lms-12-220-A-1-01.1.fid
7.66
7.66
7.66
7.65
7.65
7.64
7.26 CDCl3
7.17
7.17
7.17
7.16
7.16
7.15
6.54
6.02
2.81
2.32
AcO
SO2Me
3r
1H NMR (600 MHz, CDCl3)
1.95
1.93
1.00
1.01
2.99
2.94
f1 (ppm)

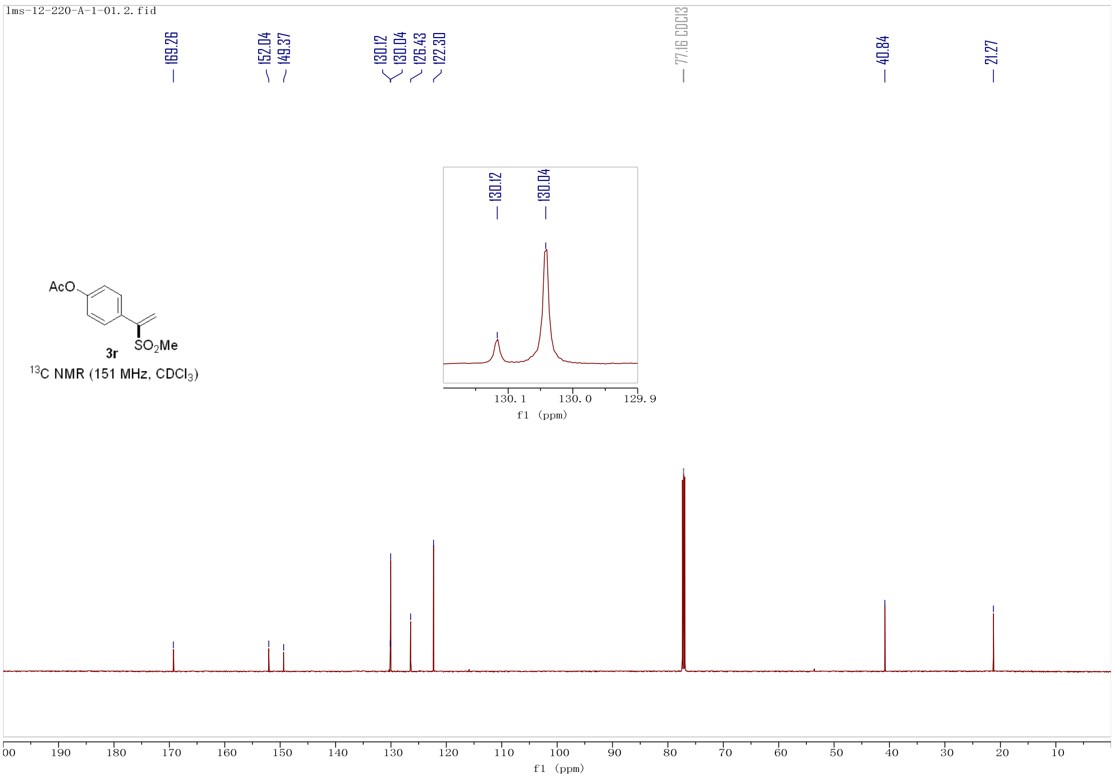
lms-12-220-A-1-01.2.fid
169.26
152.04
149.37
130.12
130.04
126.43
122.30
77.16 CDCl3
40.84
21.27
AcO
3r
SO2Me
13C NMR (151 MHz, CDCl3)
f1 (ppm)

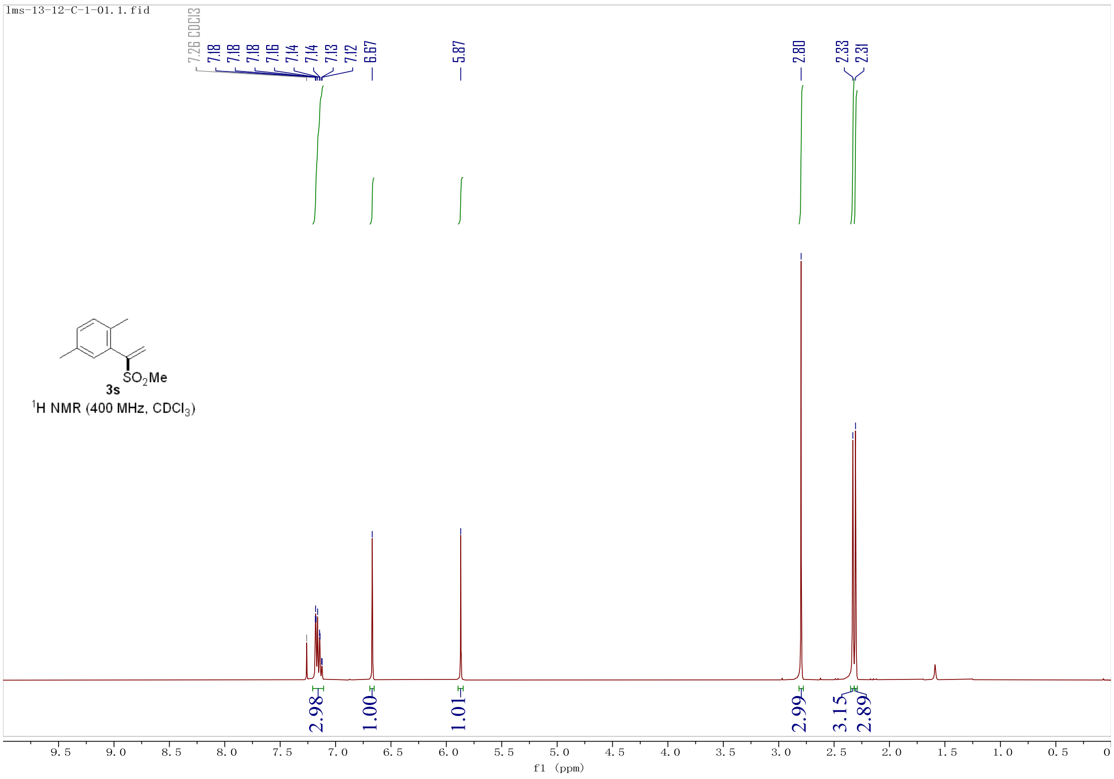
lms-13-12-C-1-01.1.fid
7.26 CDCl3
7.18
7.18
7.18
7.16
7.14
7.14
7.13
7.12
6.67
5.87
2.80
2.33
2.31
3s
SO2Me
1H NMR (400 MHz, CDCl3)
2.98
1.00
1.01
2.99
3.15
2.89
f1 (ppm)

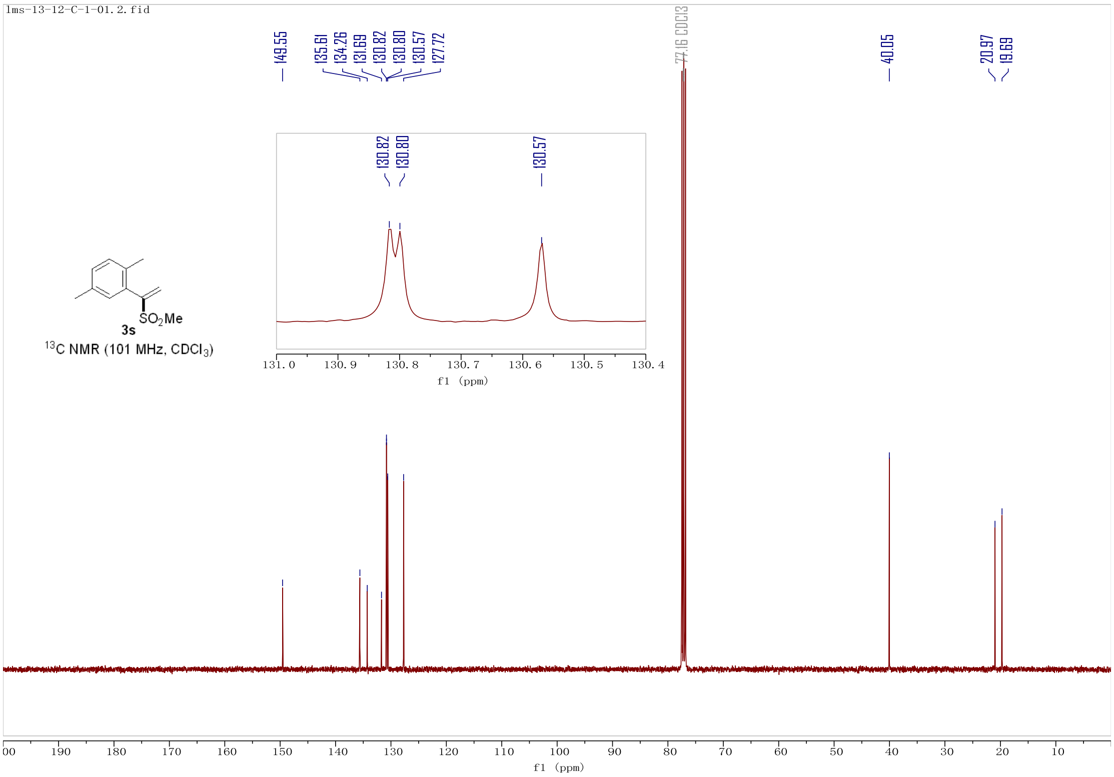
lms-13-12-C-1-01.2.fid
149.55
135.61
134.26
131.69
130.82
130.80
130.57
127.72
77.16 CDCl3
40.05
20.97
19.69
SO2Me
3s
13C NMR (101 MHz, CDCl3)
f1 (ppm)

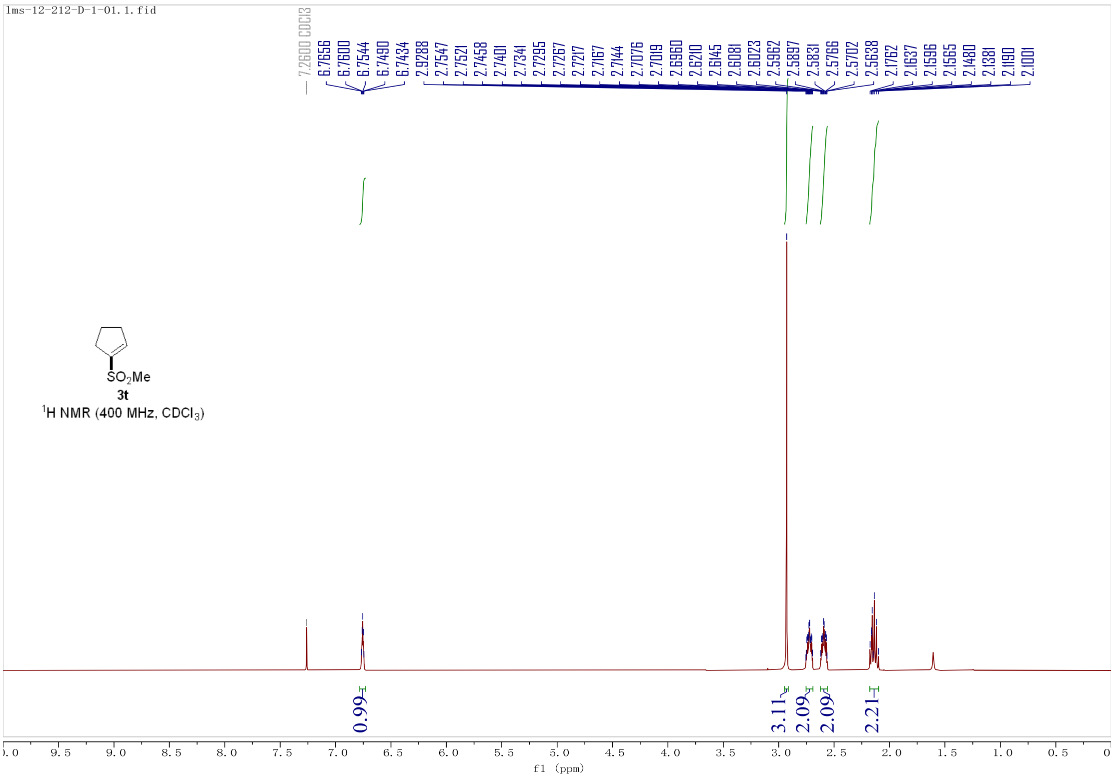
lms-12-212-D-1-01.1.fid
7.2600 CDCl3
6.7656
6.7600
6.7544
6.7490
6.7434
2.9288
2.7547
2.7521
2.7458
2.7401
2.7341
2.7295
2.7267
2.7217
2.7167
2.7144
2.7076
2.7019
2.6960
2.6210
2.6145
2.6081
2.6023
2.5962
2.5897
2.5831
2.5766
2.5702
2.5638
2.1762
2.1637
2.1596
2.1565
2.1480
2.1381
2.1190
2.1001
SO2Me
3t
1H NMR (400 MHz, CDCl3)
0.99
3.11
2.09
2.09
2.21
f1 (ppm)

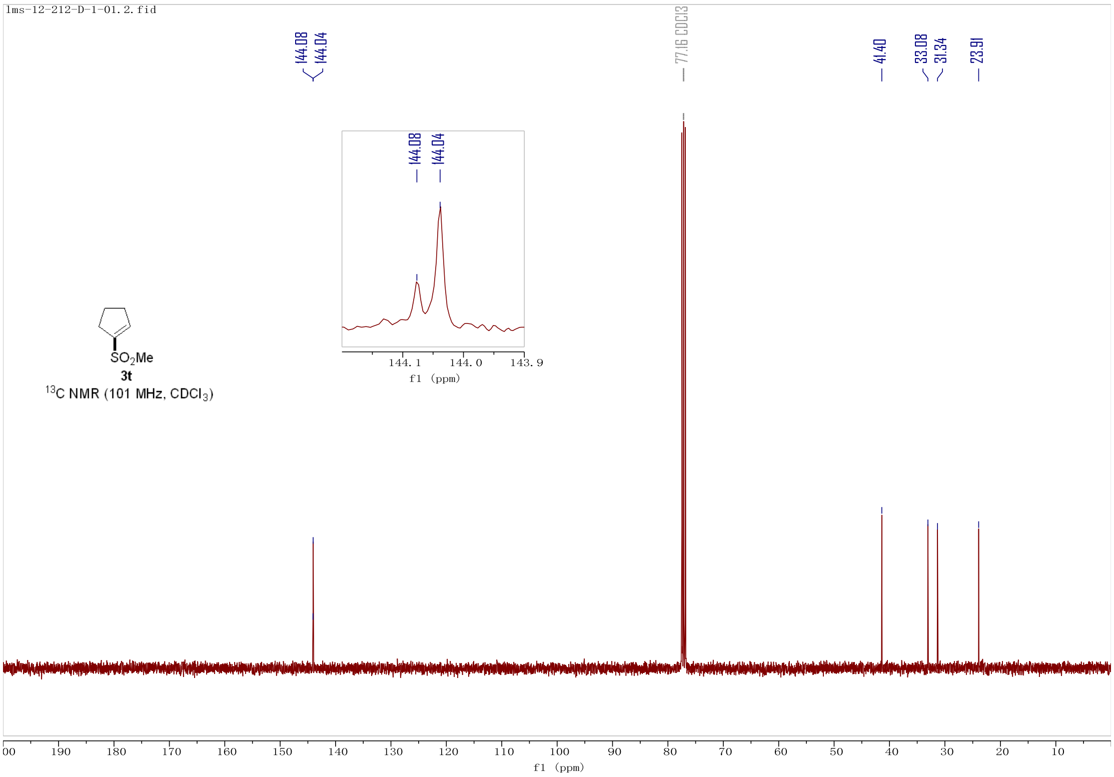
lms-12-212-D-1-01.2.fid
144.08
144.04
77.16 CDCl3
41.40
33.08
31.34
23.91
SO2Me
3t
13C NMR (101 MHz, CDCl3)
f1 (ppm)

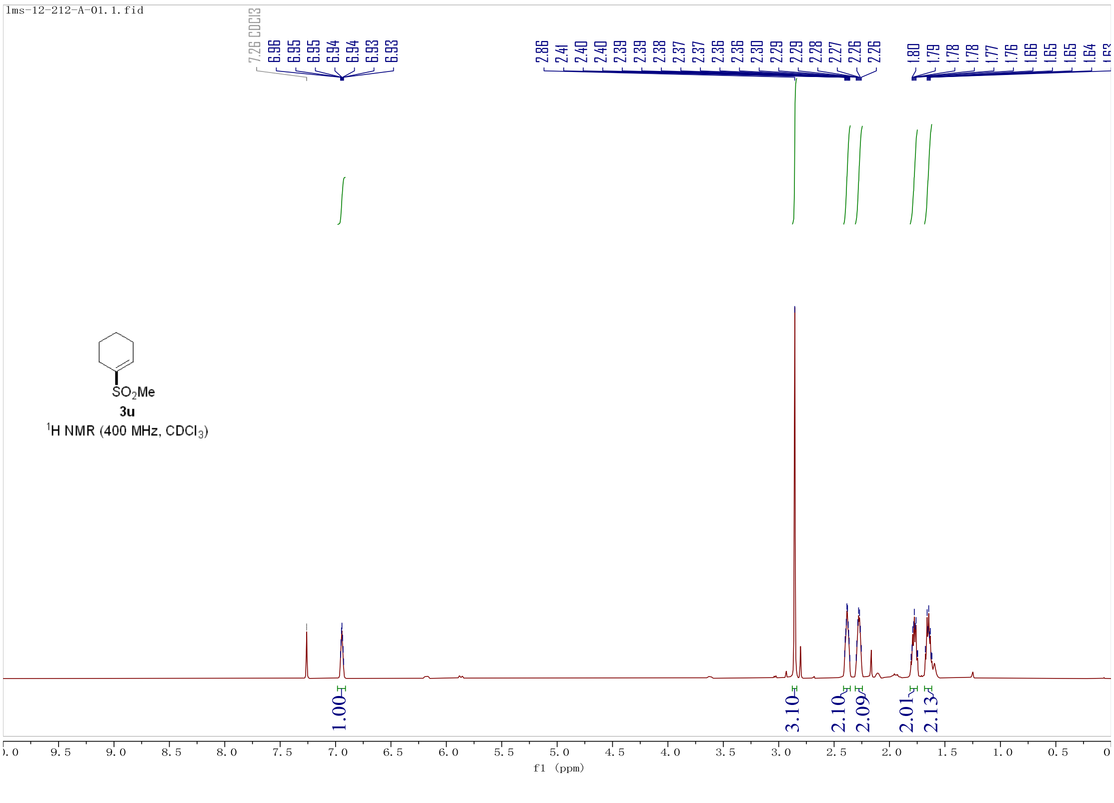
lms-12-212-A-01.1.fid
7.26 CDCl3
6.96
6.95
6.95
6.94
6.94
6.93
6.93
2.86
2.41
2.40
2.40
2.39
2.39
2.38
2.37
2.37
2.36
2.36
2.30
2.29
2.29
2.28
2.27
2.26
2.26
1.80
1.79
1.78
1.78
1.77
1.76
1.66
1.65
1.65
1.64
SO2Me
3u
1H NMR (400 MHz, CDCl3)
1.00
3.10
2.10
2.09
2.01
2.13
f1 (ppm)

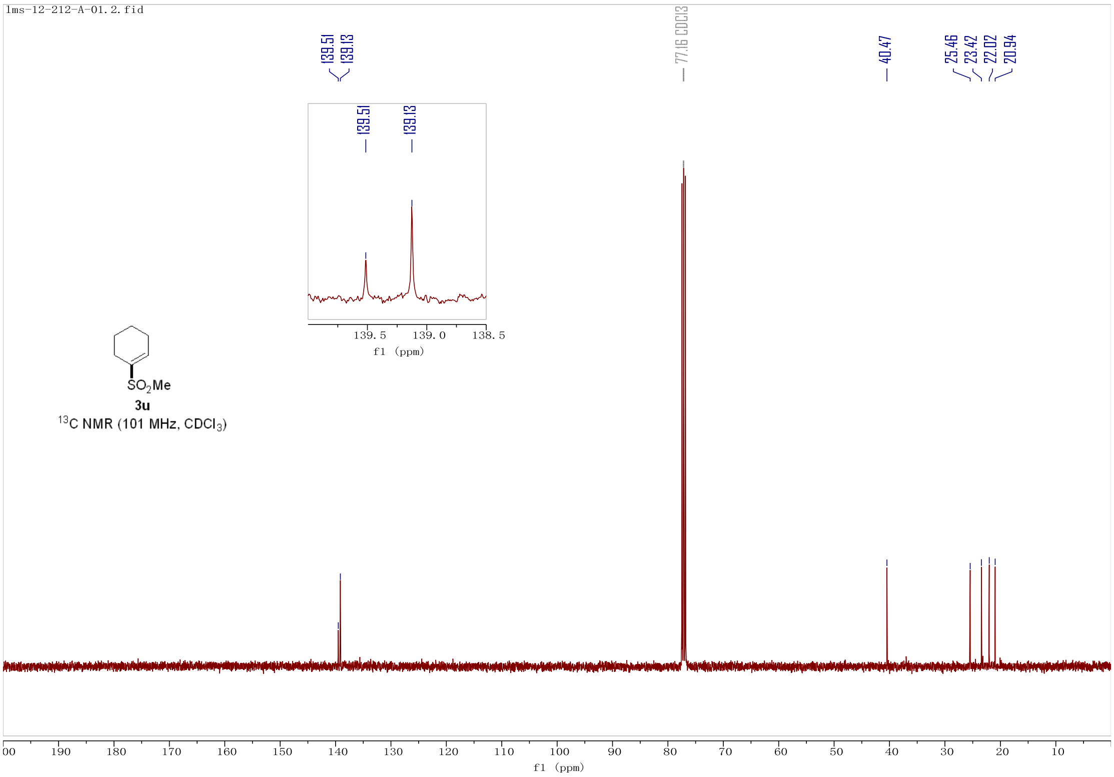
lms-12-212-A-01.2.fid
139.51
139.13
77.16 CDCl3
40.47
25.46
23.42
22.02
20.94
SO2Me
3u
13C NMR (101 MHz, CDCl3)
f1 (ppm)

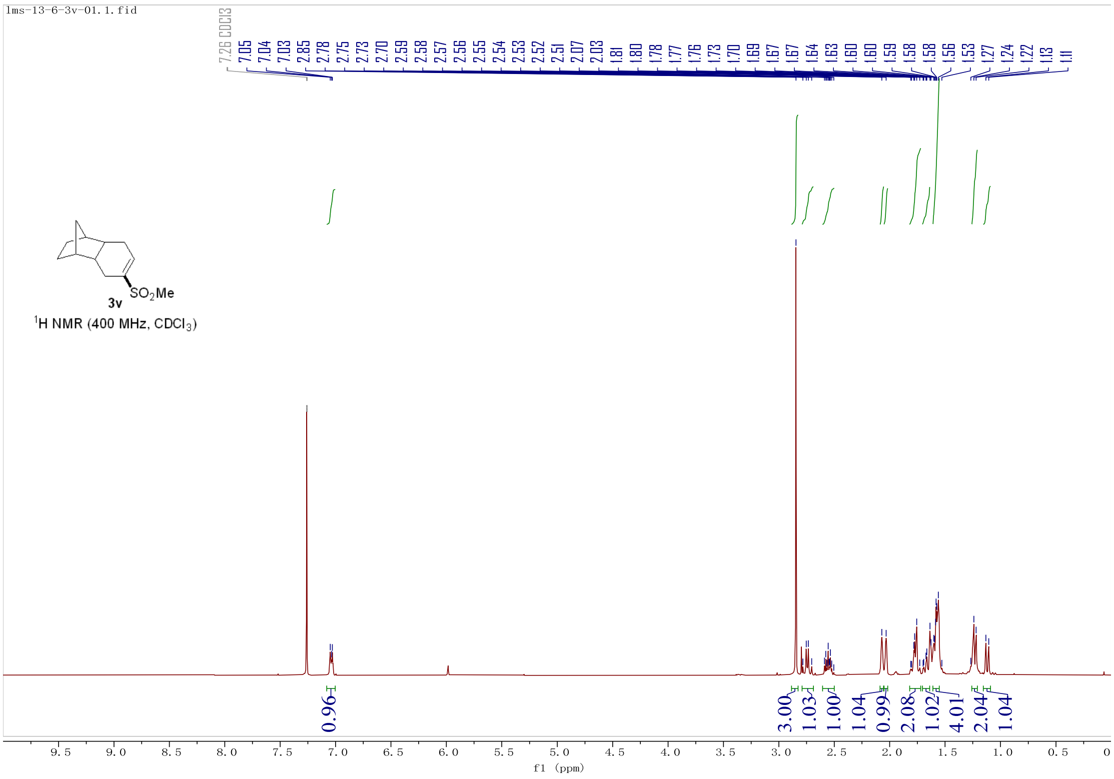
lms-13-6-3v-01.1.fid
7.26 CDCl3
7.05
7.04
7.03
2.85
2.78
2.75
2.73
2.70
2.59
2.58
2.57
2.56
2.55
2.54
2.53
2.52
2.51
2.07
2.03
1.81
1.80
1.78
1.77
1.76
1.73
1.70
1.69
1.67
1.67
1.64
1.63
1.60
1.60
1.59
1.58
1.58
1.56
1.53
1.27
1.24
1.22
1.13
1.11
SO2Me
3v
1H NMR (400 MHz, CDCl3)
0.96
3.00
1.03
1.00
1.04
0.99
2.08
1.02
4.01
2.04
1.04
f1 (ppm)

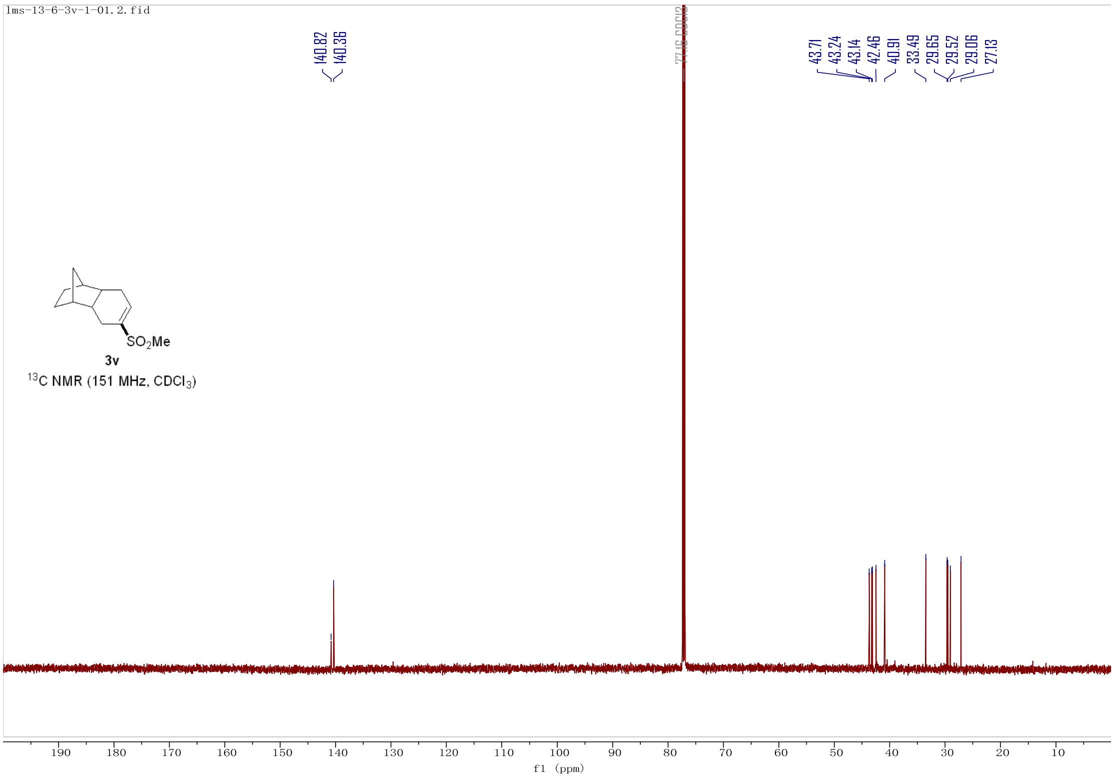
lms-13-6-3v-1-01.2.fid
140.82
140.36
77.16 CDCl3
43.71
43.24
43.14
42.46
40.91
33.49
29.65
29.52
29.06
27.13
SO2Me
3v
13C NMR (151 MHz, CDCl3)
f1 (ppm)

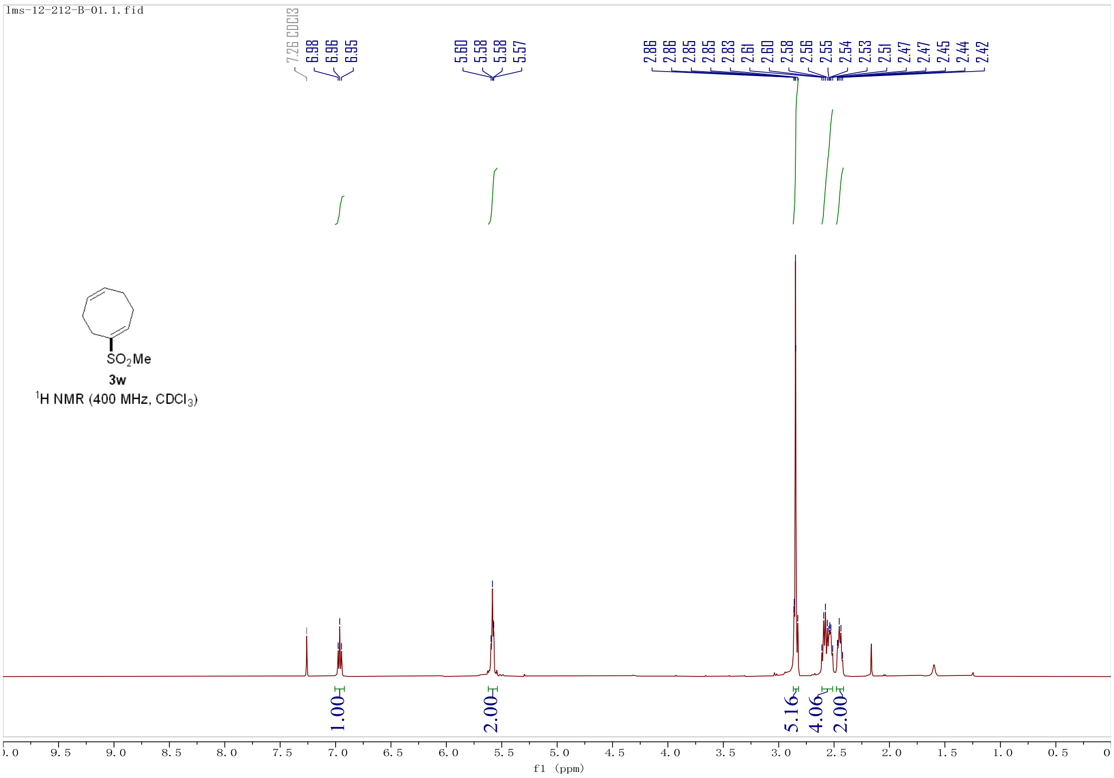
lms-12-212-B-01.1.fid
7.26 CDCl3
6.98
6.96
6.95
5.60
5.58
5.58
5.57
2.86
2.86
2.85
2.83
2.61
2.60
2.58
2.56
2.55
2.54
2.53
2.51
2.47
2.47
2.45
2.44
2.42
SO2Me
3w
1H NMR (400 MHz, CDCl3)
1.00
2.00
5.16
4.06
2.00
f1 (ppm)

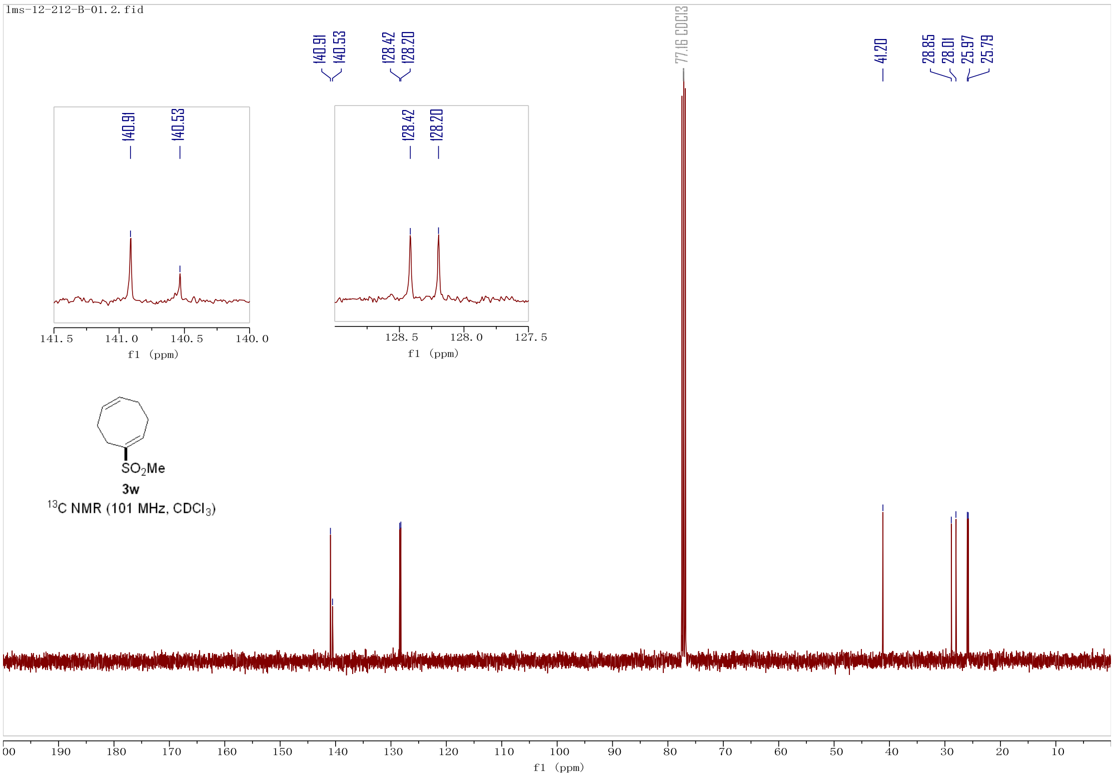
lms-12-212-B-01.2.fid
140.91
140.53
128.42
128.20
77.16 CDCl3
41.20
28.85
28.01
25.97
25.79
SO2Me
3w
13C NMR (101 MHz, CDCl3)
f1 (ppm)

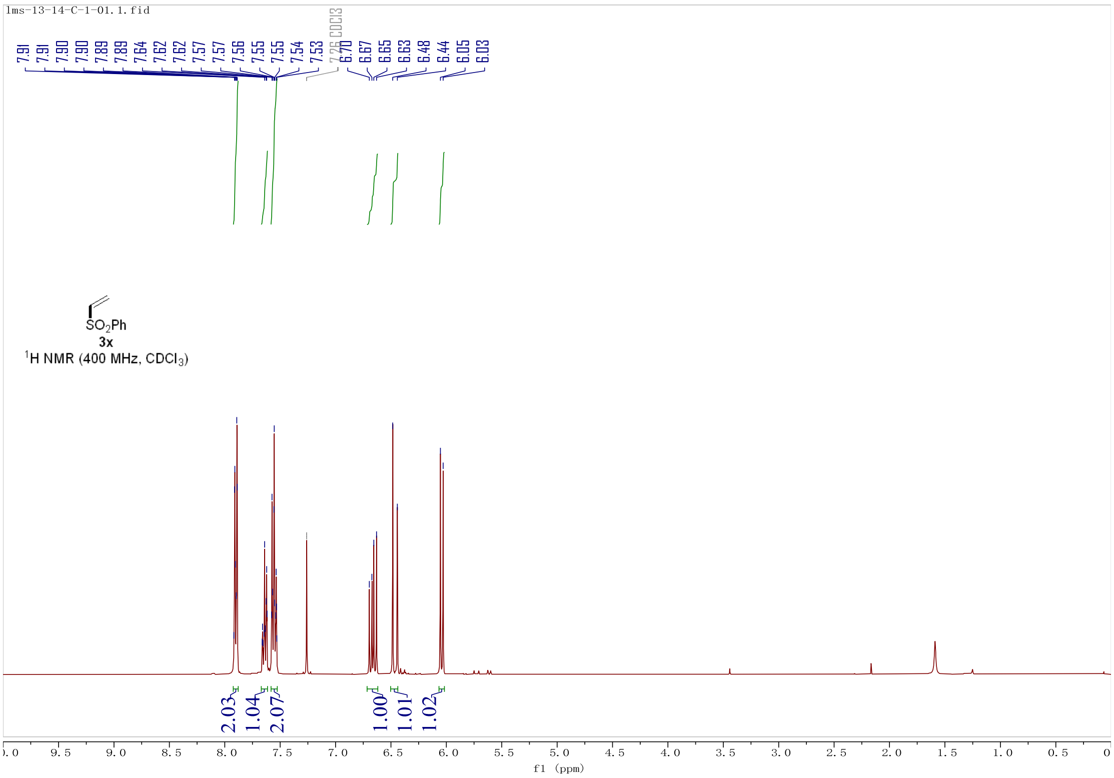
lms-13-14-C-1-01.1.fid
7.91
7.91
7.90
7.89
7.89
7.64
7.62
7.62
7.57
7.56
7.55
7.55
7.54
7.53
7.26 CDCl3
6.70
6.67
6.65
6.63
6.48
6.44
6.05
6.03
SO2Ph
3x
1H NMR (400 MHz, CDCl3)
2.03
1.04
2.07
1.00
1.01
1.02
f1 (ppm)

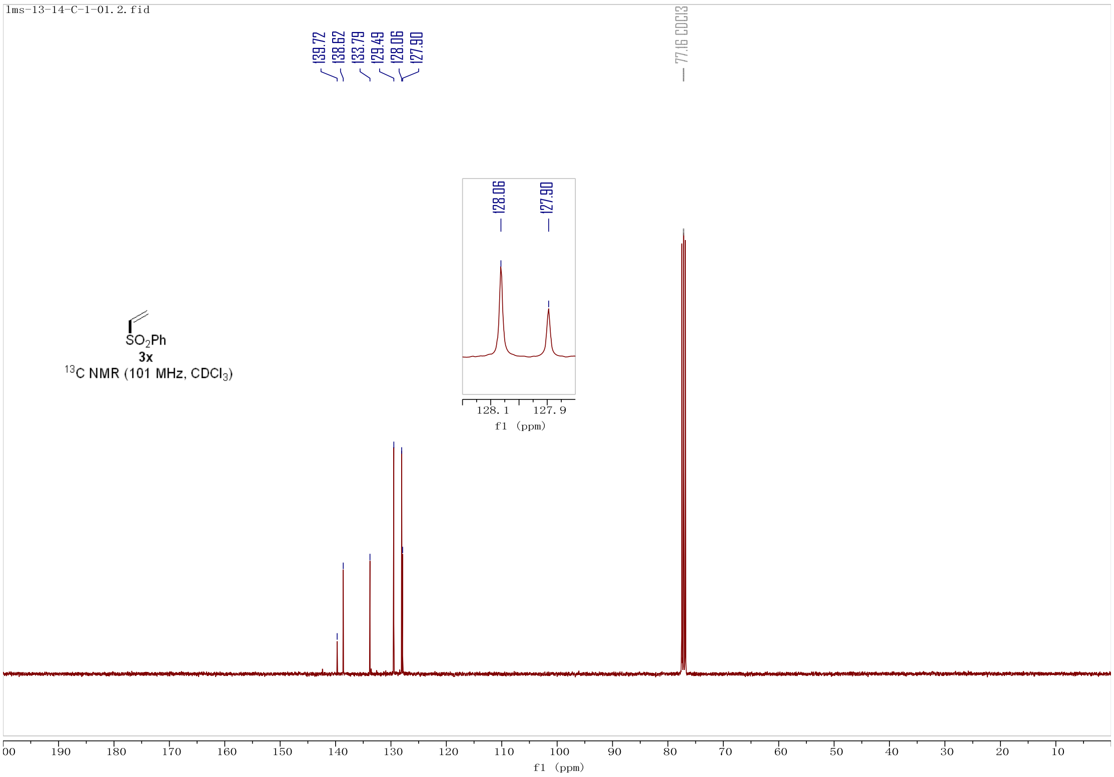
lms-13-14-C-1-01.2.fid
139.72
138.62
133.79
129.49
128.06
127.90
77.16 CDCl3
SO2Ph
3x
13C NMR (101 MHz, CDCl3)
f1 (ppm)

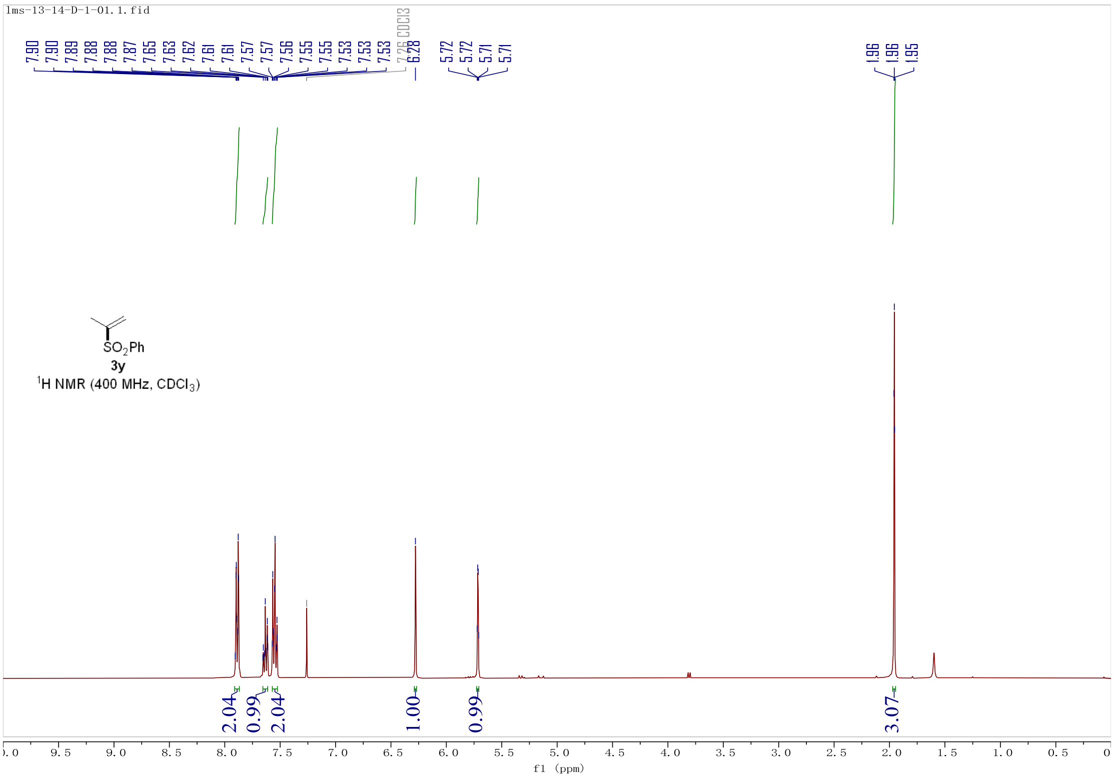
lms-13-14-D-1-01.1.fid
7.26 CDCl3
6.28
5.72
5.72
5.71
5.71
1.96
1.96
1.95
SO2Ph
3y
1H NMR (400 MHz, CDCl3)
2.04
0.99
2.04
1.00
0.99
3.07
f1 (ppm)

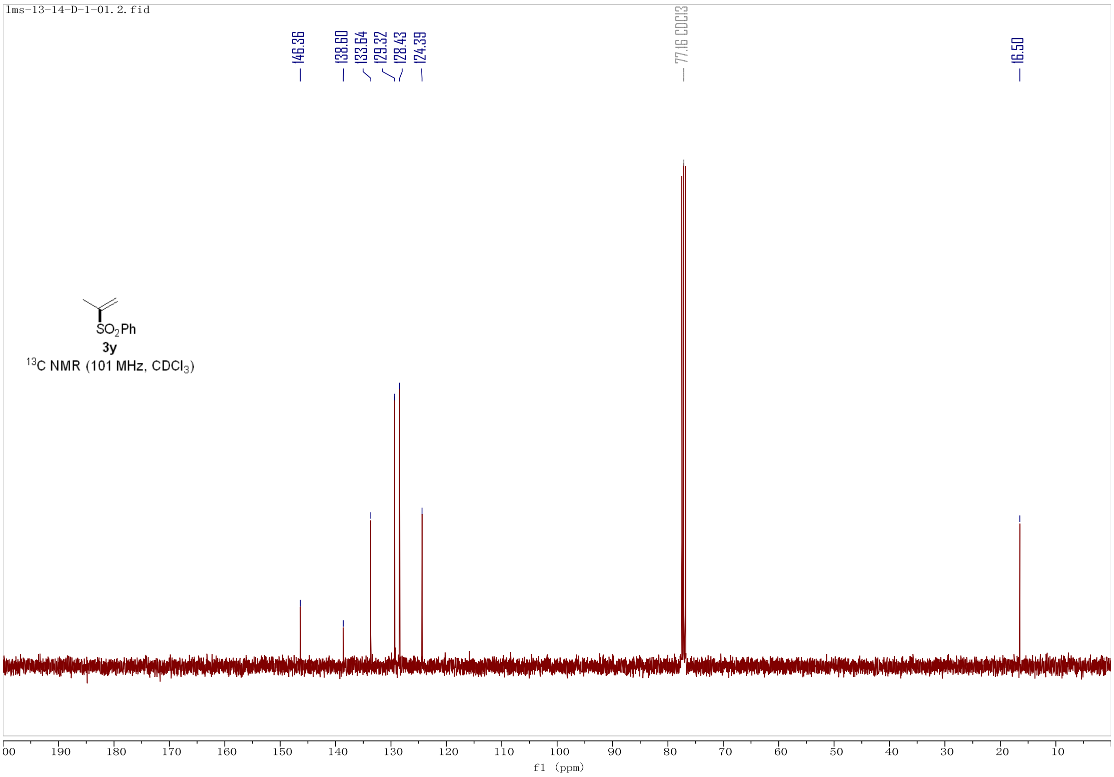

lms-13-14-D-1-01.2.fid
146.36
138.60
133.64
129.32
128.43
124.39
77.16 CDCl3
16.50
SO2Ph
3y
13C NMR (101 MHz, CDCl3)
f1 (ppm)

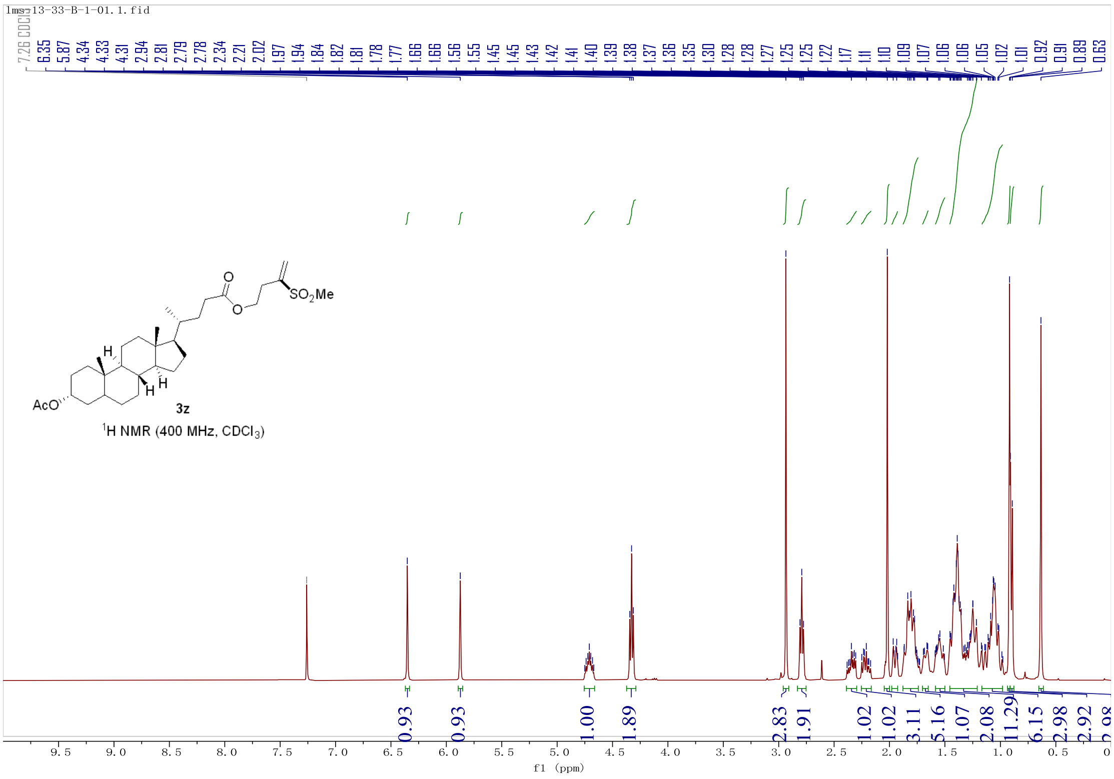

lms-13-33-B-1-01.1.fid
7.26 CDCl3
6.35
5.87
4.34
4.33
4.31
2.94
2.81
2.79
2.78
2.34
2.21
2.02
1.97
1.94
1.84
1.82
1.81
1.78
1.77
1.66
1.66
1.56
1.55
1.45
1.45
1.43
1.42
1.41
1.40
1.39
1.38
1.37
1.36
1.35
1.30
1.28
1.28
1.27
1.25
1.25
1.22
1.17
1.11
1.10
1.09
1.07
1.06
1.06
1.05
1.02
1.01
0.92
0.91
0.89
0.63
SO2Me
AcO
H
H
H
3z
1H NMR (400 MHz, CDCl3)
0.93
0.93
1.00
1.89
2.83
1.91
1.02
1.02
3.11
5.16
1.07
2.08
11.29
6.15
2.98
2.92
f1 (ppm)

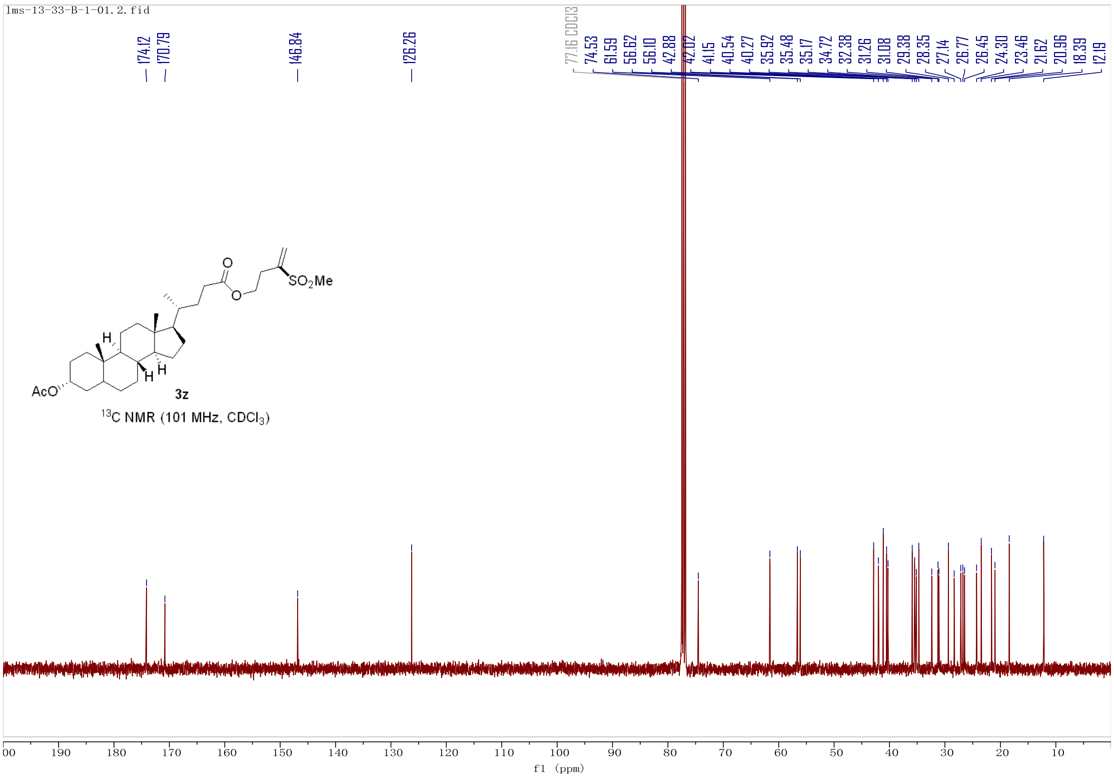
lms-13-33-B-1-01.2.fid
174.12
170.79
146.84
126.26
77.16 CDCl3
74.53
61.59
56.62
56.10
42.88
42.02
41.15
40.54
40.27
35.92
35.48
35.17
34.72
32.38
31.26
31.08
29.38
28.35
27.14
26.77
26.45
24.30
23.46
21.62
20.96
18.39
12.19
O
SO2Me
H
H
H
AcO
3z
13C NMR (101 MHz, CDCl3)
f1 (ppm)

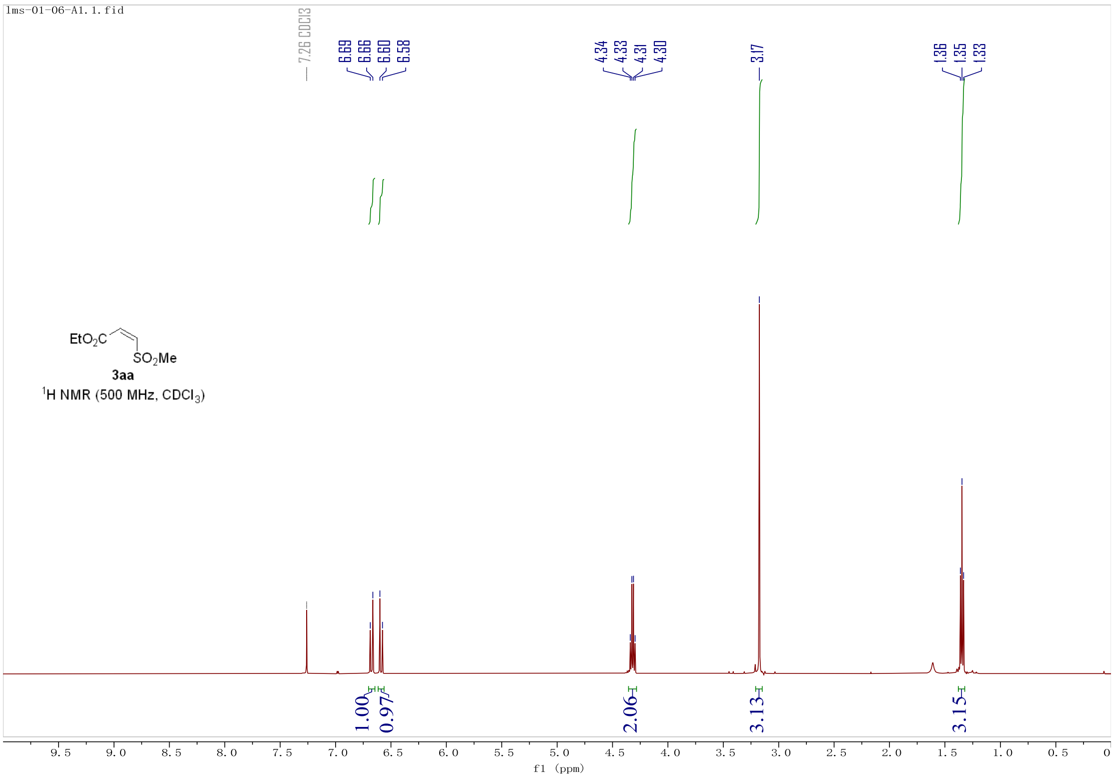
lms-01-06-A1.1.fid
7.26 CDCl3
6.69
6.66
6.60
6.58
4.34
4.33
4.31
4.30
3.17
1.36
1.35
1.33
EtO2C
SO2Me
3aa
1H NMR (500 MHz, CDCl3)
1.00
0.97
2.06
3.13
3.15
f1 (ppm)

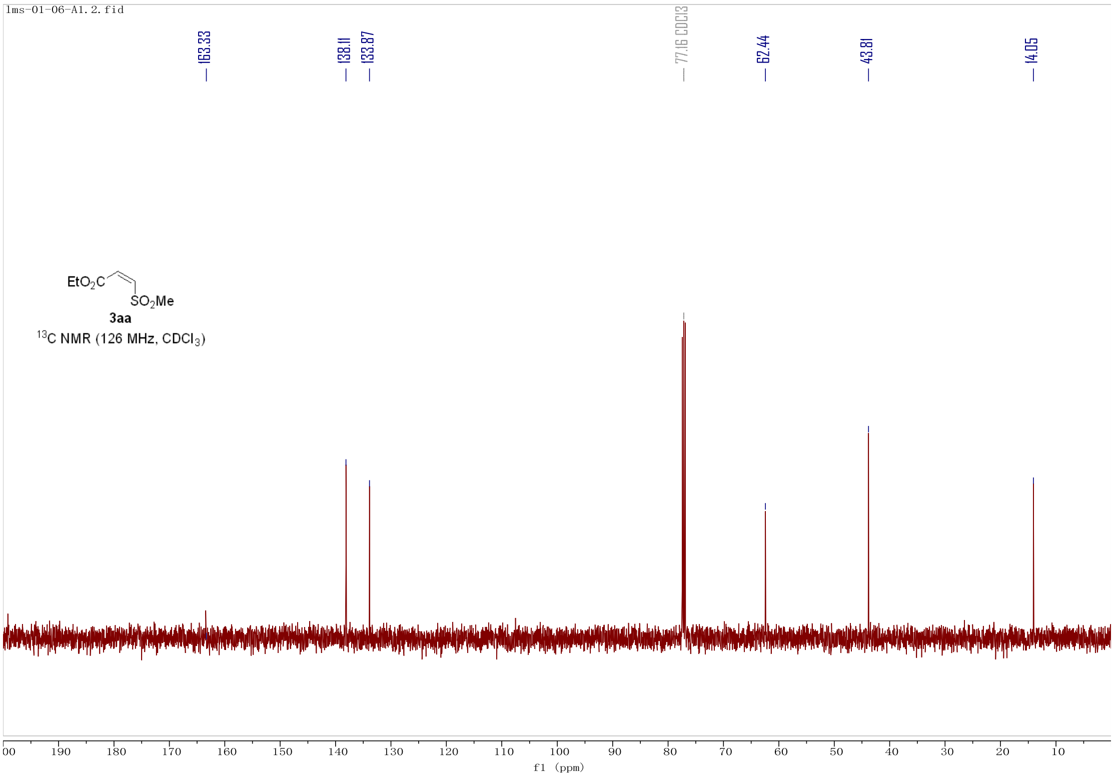
lms-01-06-A1.2.fid
163.33
138.11
133.87
77.16 CDCl3
62.44
43.81
14.05
EtO2C
SO2Me
3aa
13C NMR (126 MHz, CDCl3)
f1 (ppm)

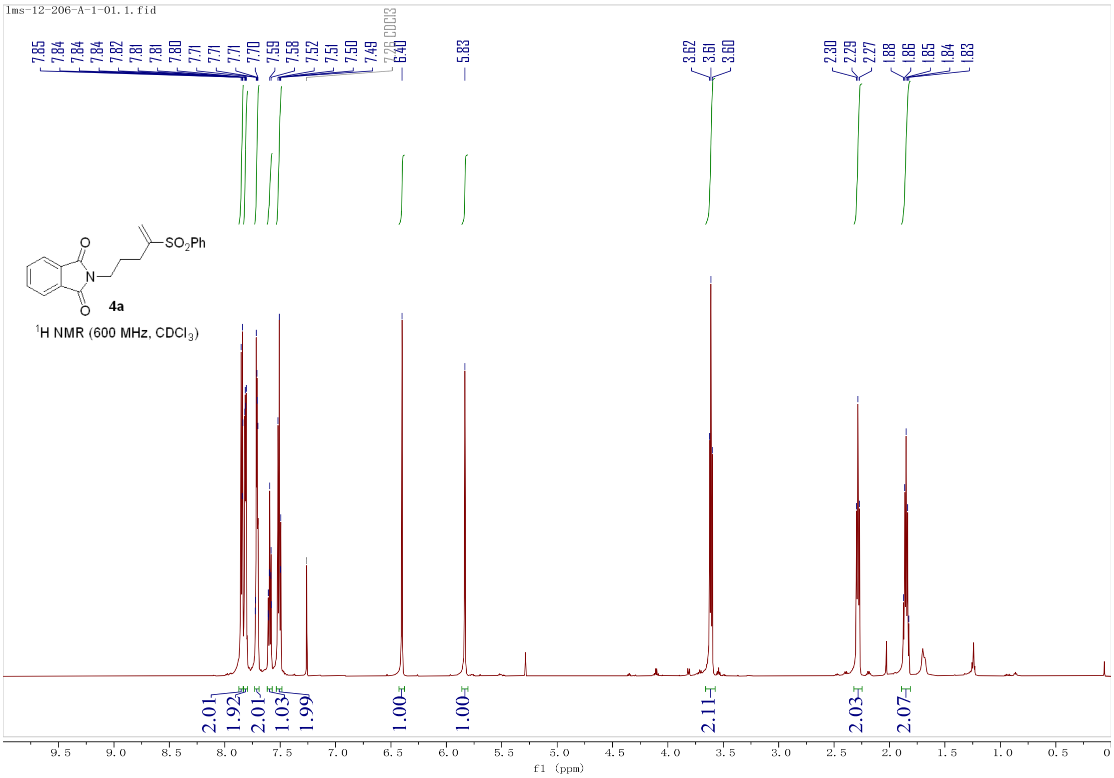
lms-12-206-A-1-01.1.fid
7.85
7.84
7.84
7.84
7.82
7.81
7.81
7.80
7.71
7.71
7.71
7.70
7.59
7.58
7.52
7.51
7.50
7.49
7.26 CDCl3
6.40
5.83
3.62
3.61
3.60
2.30
2.29
2.27
1.88
1.86
1.85
1.84
1.83
SO2Ph
4a
1H NMR (600 MHz, CDCl3)
2.01
1.92
2.01
1.03
1.99
1.00
1.00
2.11
2.03
2.07
f1 (ppm)

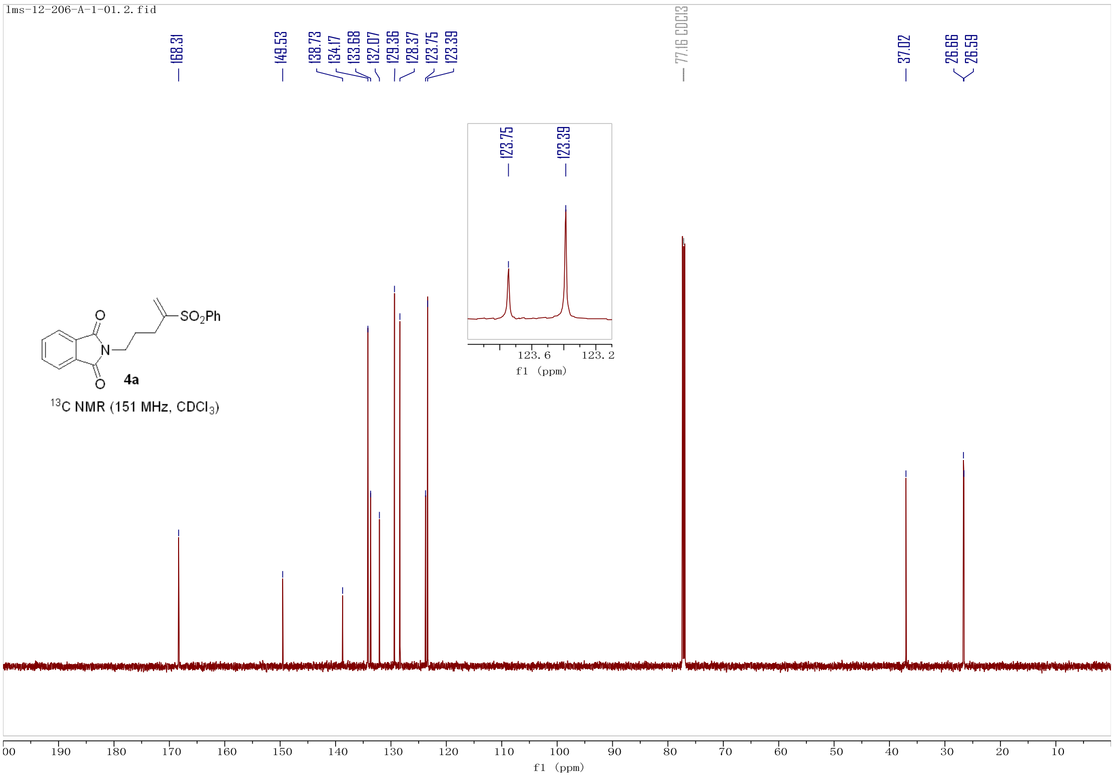
lms-12-206-A-1-01.2.fid
168.31
149.53
138.73
134.17
133.68
132.07
129.36
128.37
123.75
123.39
77.16 CDCl3
37.02
26.66
26.59
123.75
123.39
123.6
123.2
f1 (ppm)
SO2Ph
O
N
O
4a
13C NMR (151 MHz, CDCl3)
f1 (ppm)

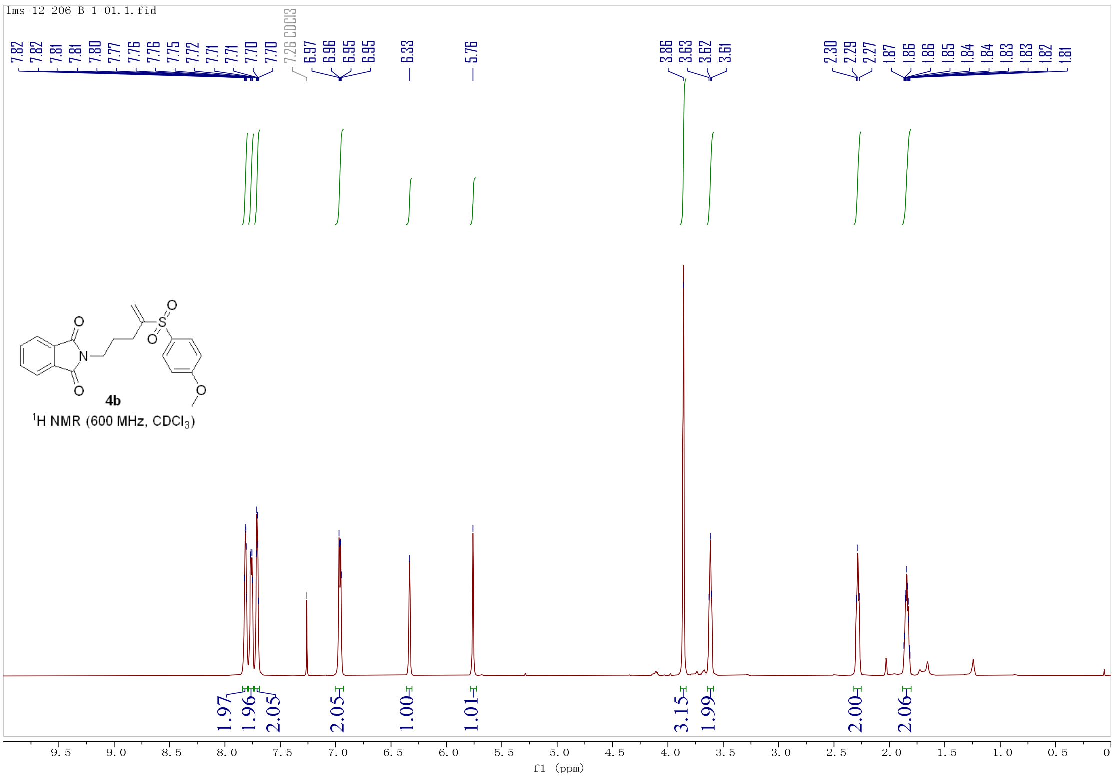
lms-12-206-B-1-01.1.fid
7.82
7.82
7.81
7.81
7.80
7.77
7.76
7.76
7.75
7.72
7.71
7.71
7.70
7.70
7.26 CDCl3
6.97
6.96
6.95
6.95
6.33
5.76
3.86
3.63
3.62
3.61
2.30
2.29
2.27
1.87
1.86
1.86
1.85
1.84
1.84
1.83
1.83
1.82
1.81
4b
1H NMR (600 MHz, CDCl3)
1.97
1.96
2.05
2.05
1.00
1.01
3.15
1.99
2.00
2.06
f1 (ppm)

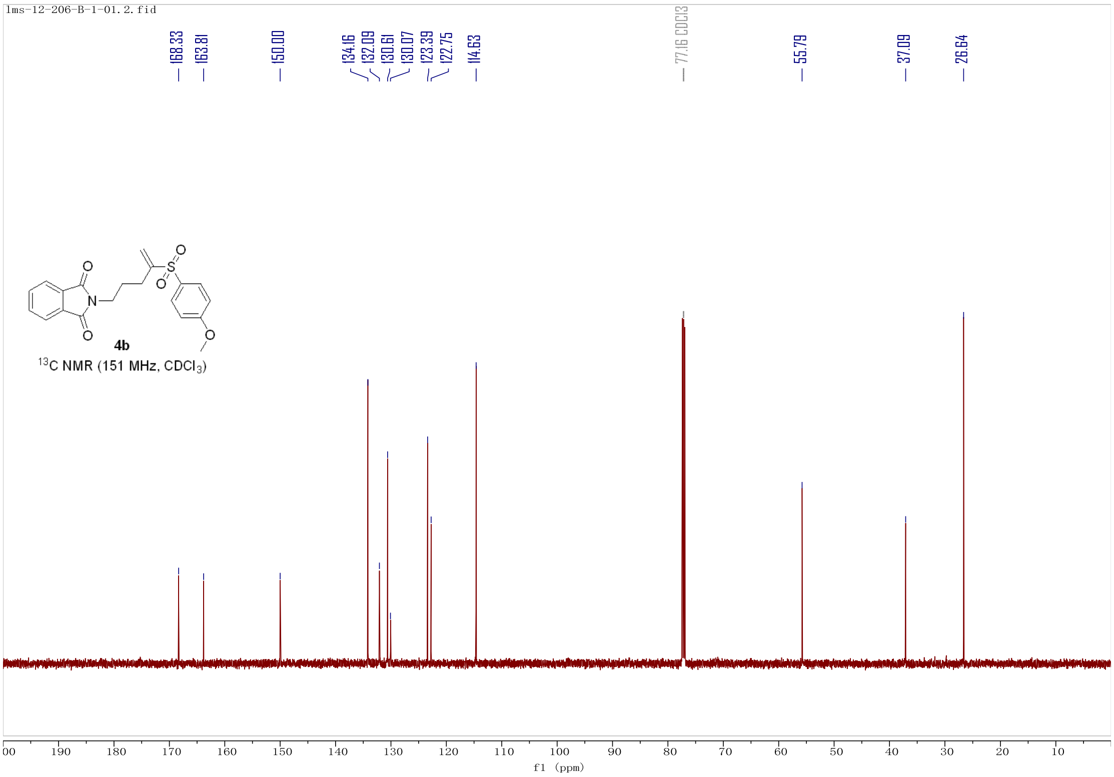
lms-12-206-B-1-01.2.fid
168.33
163.81
150.00
134.16
132.09
130.61
130.07
123.39
122.75
114.63
77.16 CDCl3
55.79
37.09
26.64
4b
^{13}C NMR (151 MHz, CDCl$_3$)
f1 (ppm)

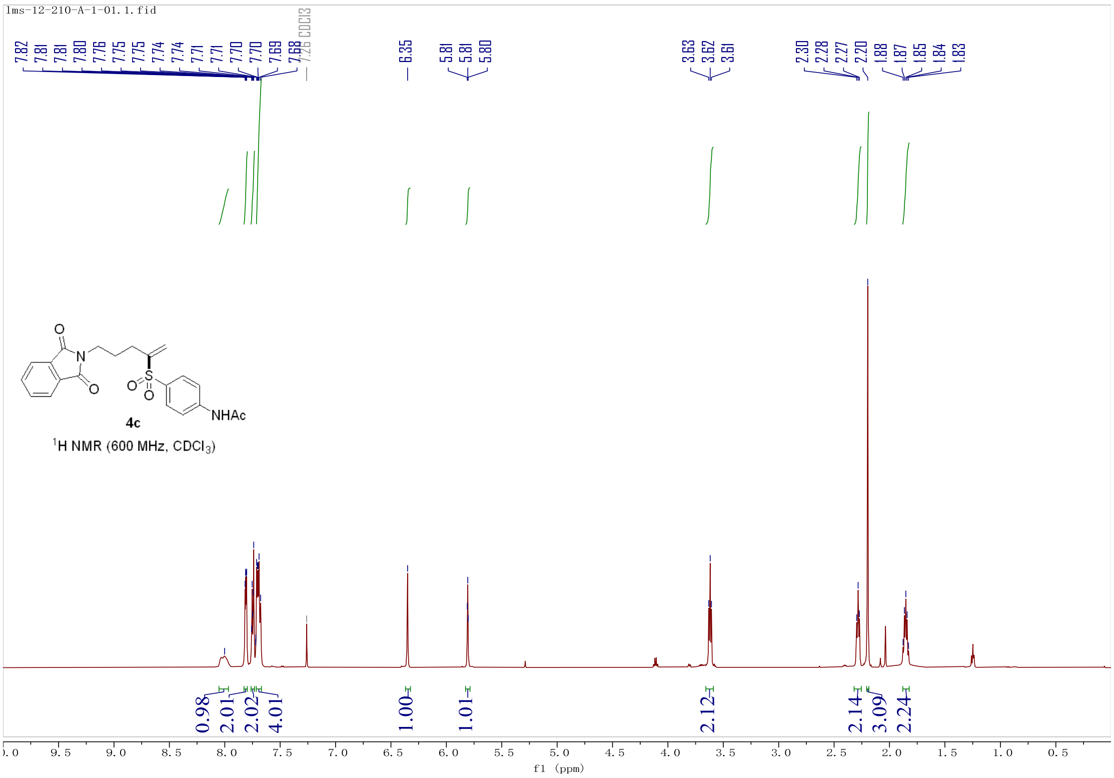
lms-12-210-A-1-01.1.fid
7.82 7.81 7.81 7.80 7.76 7.75 7.75 7.74 7.74 7.71 7.71 7.70 7.70 7.69 7.68
7.26 CDCl3
6.35
5.81 5.81 5.80
3.63 3.62 3.61
2.30 2.28 2.27 2.20
1.88 1.87 1.85 1.84 1.83
NHAc
4c
^{1}H NMR (600 MHz, CDCl$_3$)
0.98 2.01 2.02 4.01 1.00 1.01 2.12 2.14 3.09 2.24
f1 (ppm)

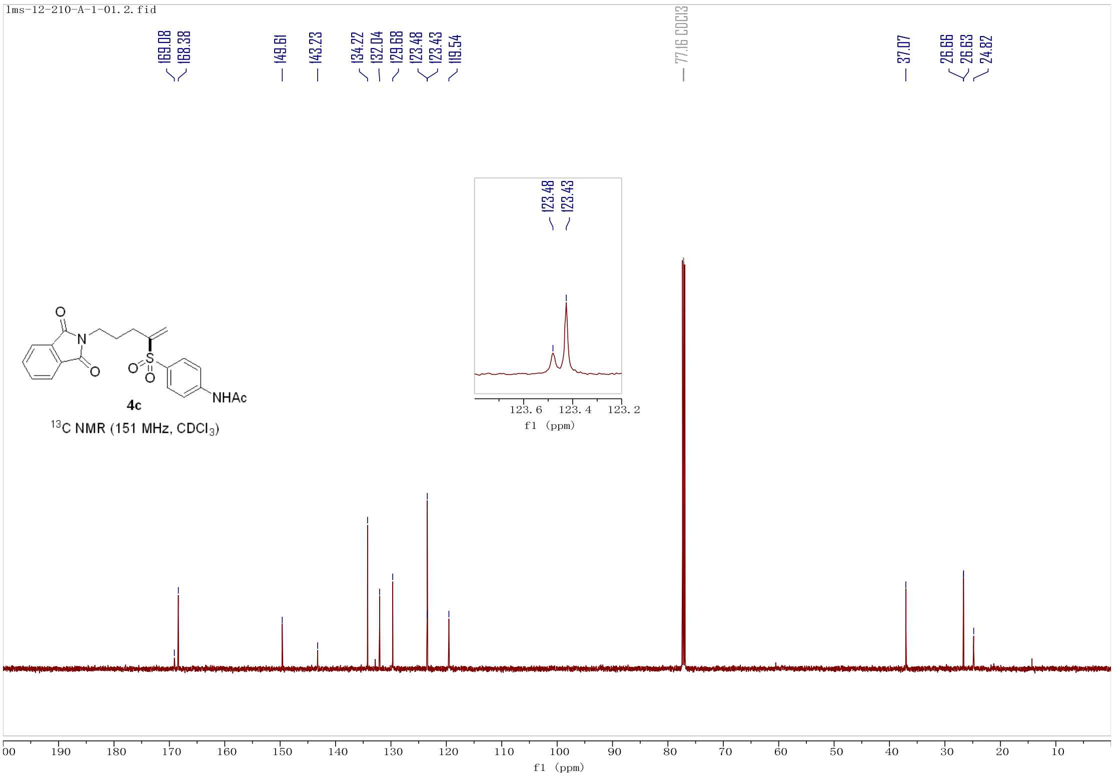
lms-12-210-A-1-01.2.fid
169.08
168.38
149.61
143.23
134.22
132.04
129.68
123.48
123.43
119.54
77.16 CDCl3
37.07
26.66
26.63
24.82
4c
13C NMR (151 MHz, CDCl3)
NHAc
f1 (ppm)

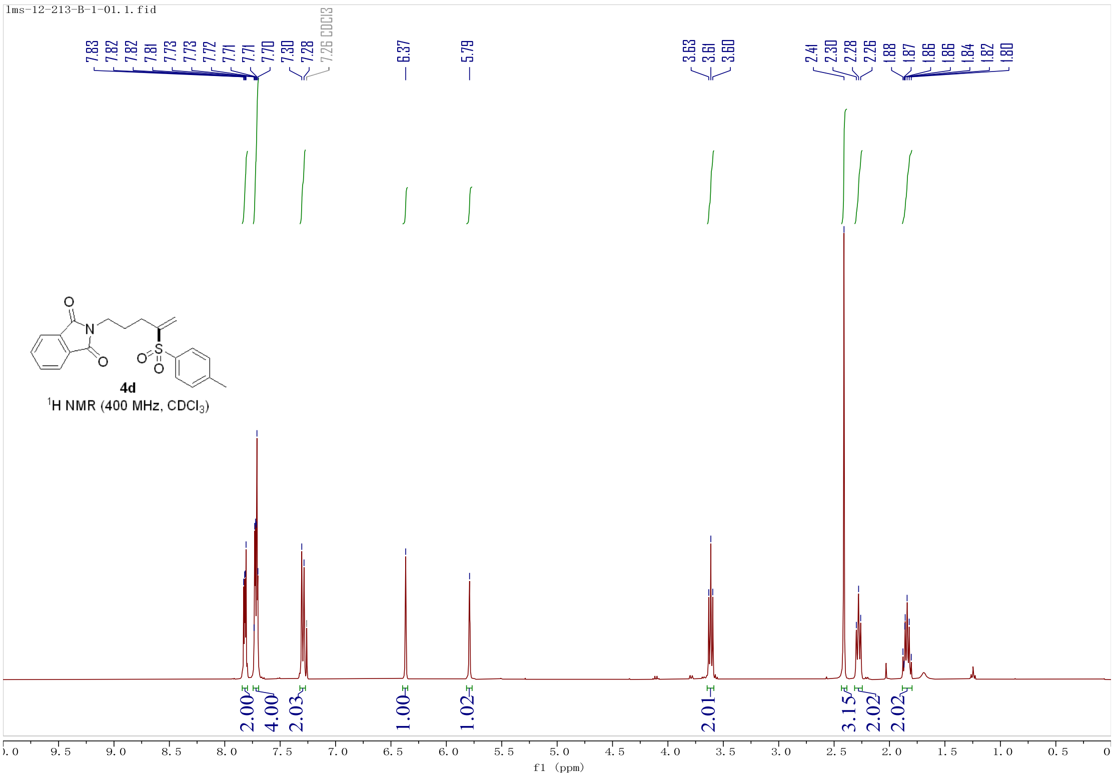
lms-12-213-B-1-01.1.fid
7.83
7.82
7.82
7.81
7.73
7.73
7.72
7.71
7.71
7.70
7.30
7.28
7.26 CDCl3
6.37
5.79
3.63
3.61
3.60
2.41
2.30
2.28
2.26
1.88
1.87
1.86
1.86
1.84
1.82
1.80
4d
1H NMR (400 MHz, CDCl3)
2.00
4.00
2.03
1.00
1.02
2.01
3.15
2.02
2.02
f1 (ppm)

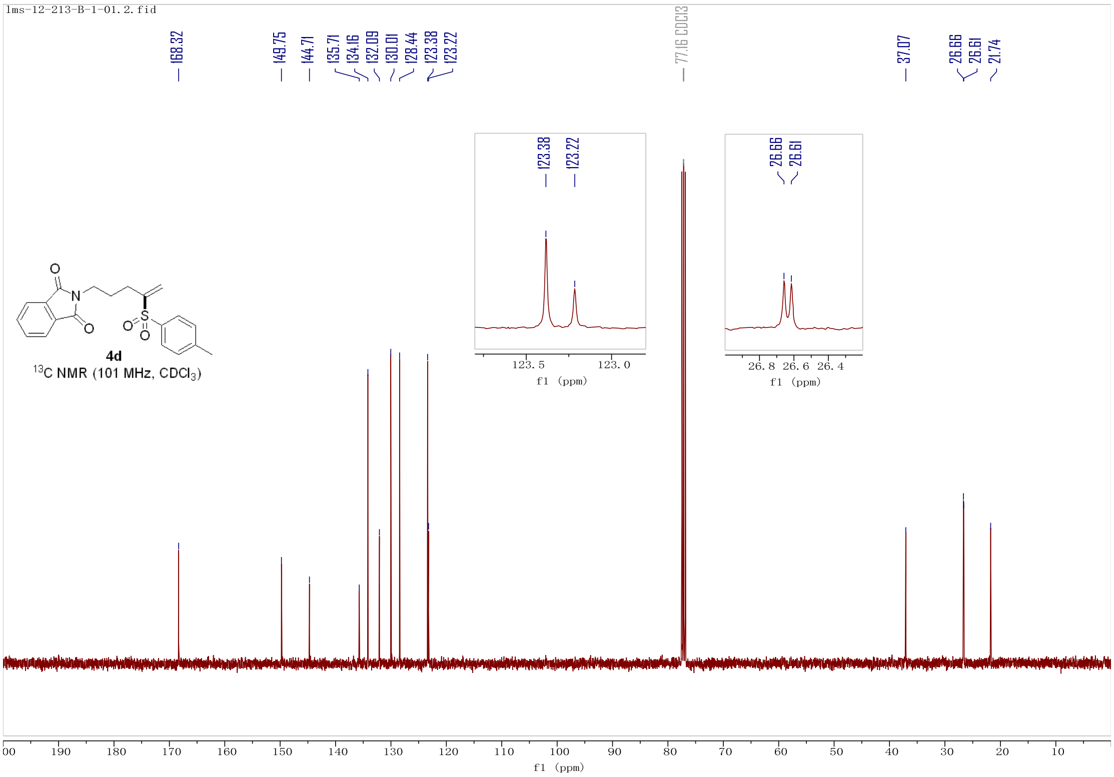
lms-12-213-B-1-01.2.fid
168.32
149.75
144.71
135.71
134.16
132.09
130.01
128.44
123.38
123.22
77.16 CDCl3
37.07
26.66
26.61
21.74
4d
^{13}C NMR (101 MHz, $CDCl_3$)
f1 (ppm)

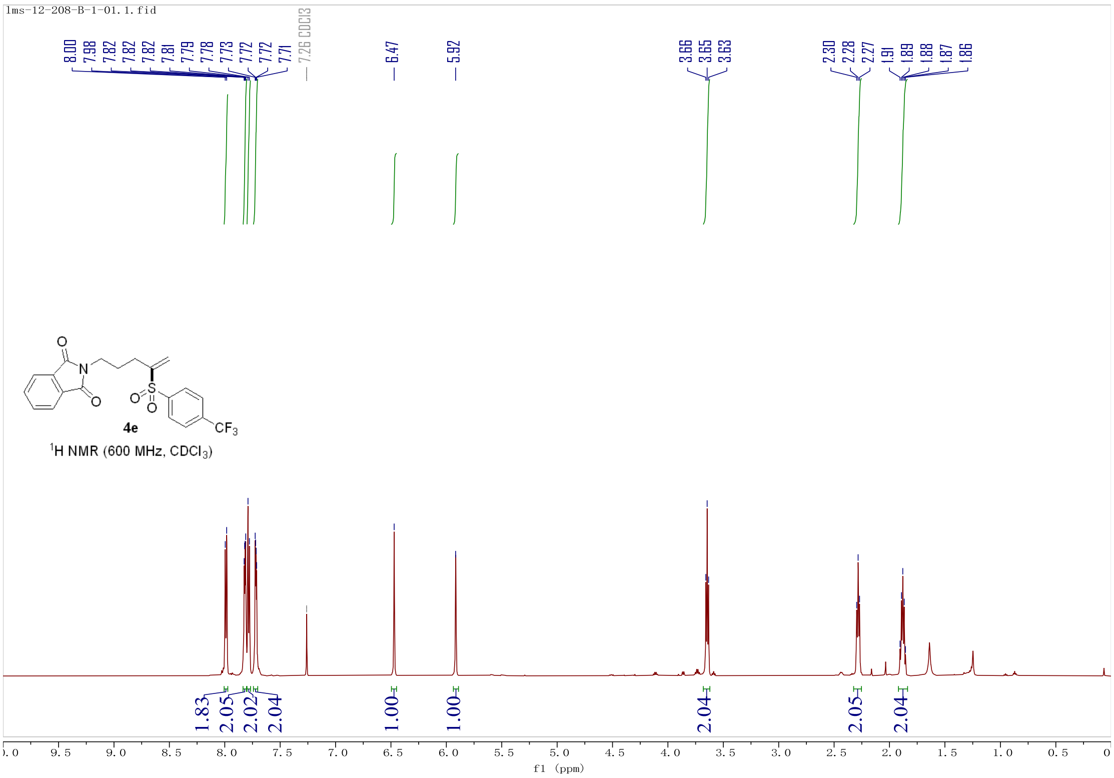
lms-12-208-B-1-01.1.fid
8.00
7.98
7.82
7.82
7.82
7.81
7.79
7.78
7.73
7.72
7.72
7.71
7.26 CDCl3
6.47
5.92
3.66
3.65
3.63
2.30
2.28
2.27
1.91
1.89
1.88
1.87
1.86
4e
^{1}H NMR (600 MHz, $CDCl_3$)
1.83
2.05
2.02
2.04
1.00
1.00
2.04
2.05
2.04
f1 (ppm)

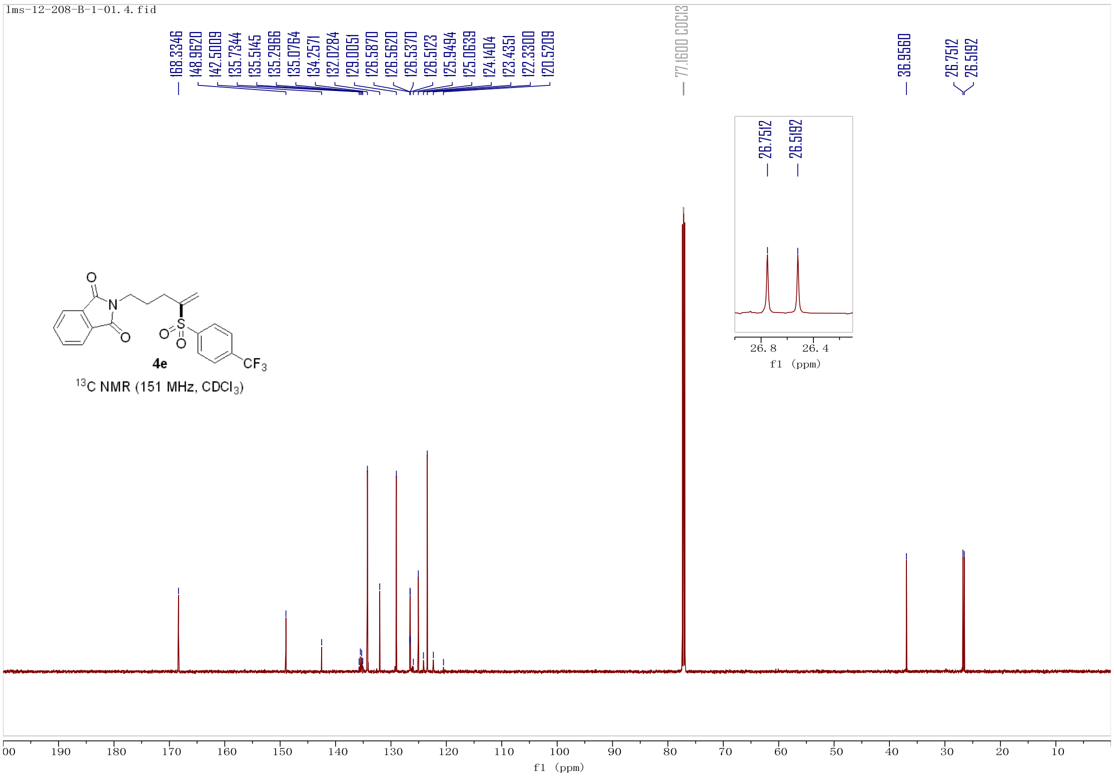
lms-12-208-B-1-01.4.fid
168.3346
148.9620
142.5009
135.7344
135.5145
135.2966
135.0764
134.2571
132.0284
129.0051
126.5870
126.5620
126.5370
126.5123
125.9494
125.0639
124.1404
123.4351
122.3300
120.5209
77.1600 CDCl3
36.9560
26.7512
26.5192
4e
13C NMR (151 MHz, CDCl3)
26.8
26.4
f1 (ppm)
00
190
180
170
160
150
140
130
120
110
100
90
80
70
60
50
40
30
20
10
f1 (ppm)

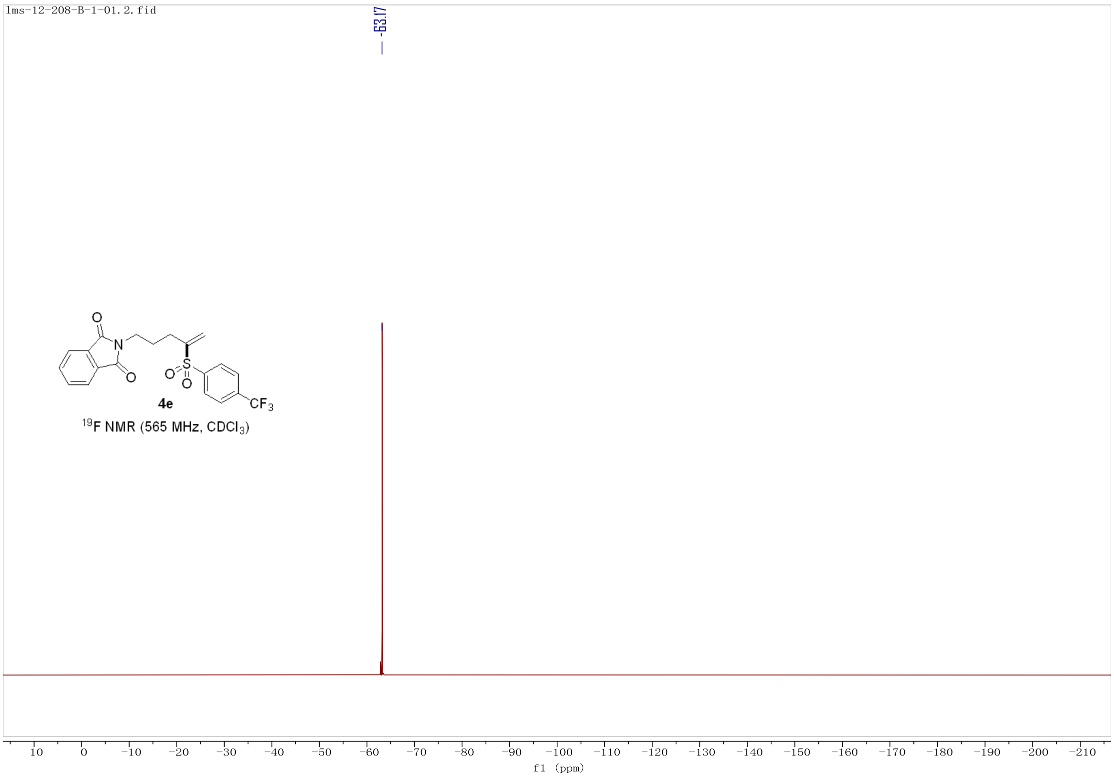
lms-12-208-B-1-01.2.fid
-63.17
4e
19F NMR (565 MHz, CDCl3)
10
0
-10
-20
-30
-40
-50
-60
-70
-80
-90
-100
-110
-120
-130
-140
-150
-160
-170
-180
-190
-200
-210
f1 (ppm)

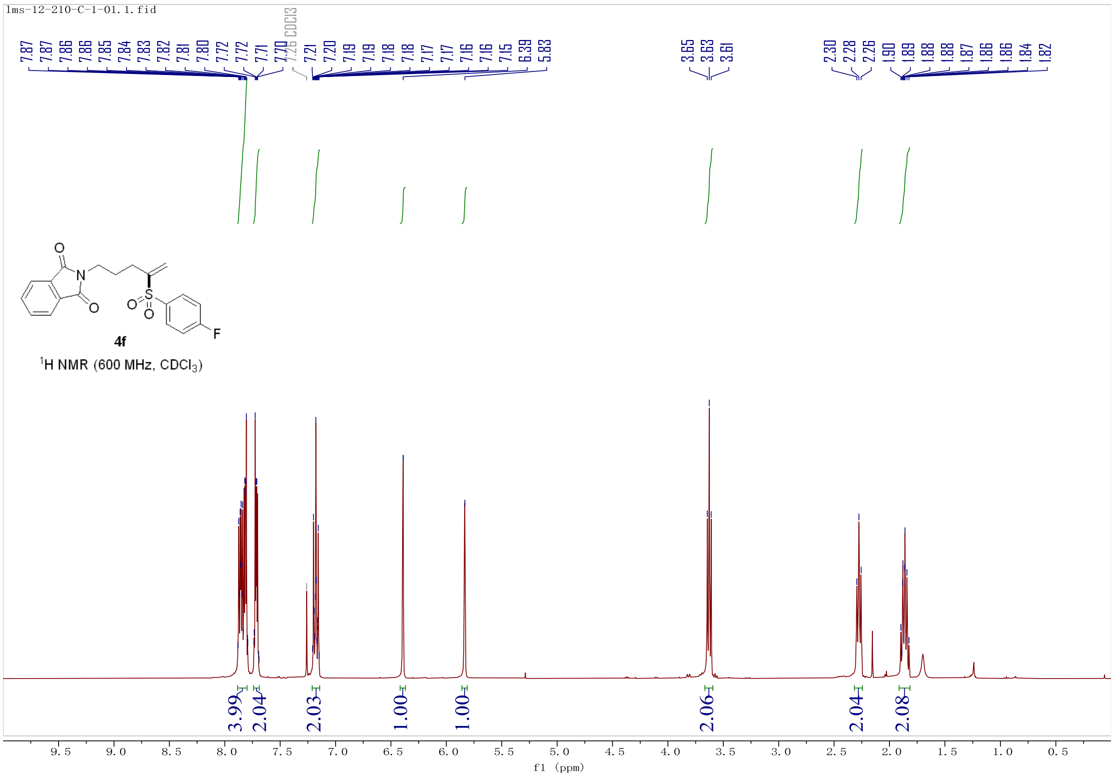
lms-12-210-C-1-01.1.fid
7.87 7.87 7.86 7.86 7.85 7.84 7.83 7.82 7.81 7.80 7.72 7.72 7.71 7.70 7.26 CDCl3 7.21 7.20 7.19 7.19 7.18 7.18 7.17 7.17 7.16 7.16 7.15 6.39 5.83
3.65 3.63 3.61
2.30 2.28 2.26 1.90 1.89 1.88 1.88 1.87 1.86 1.86 1.84 1.82
4f
^{1}H NMR (600 MHz, CDCl$_3$)
3.99 2.04 2.03 1.00 1.00 2.06 2.04 2.08
f1 (ppm)

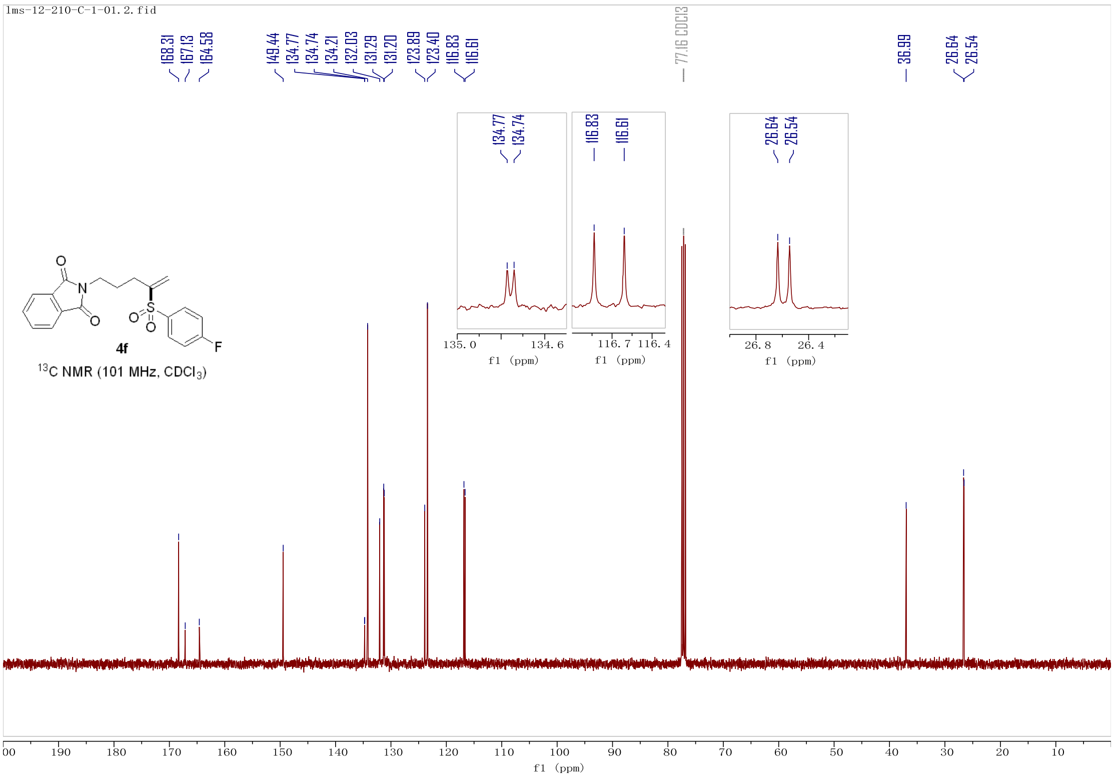
lms-12-210-C-1-01.2.fid
168.31 167.13 164.58 149.44 134.77 134.74 134.21 132.03 131.29 131.20 123.89 123.40 116.83 116.61
77.16 CDCl3
36.99 26.64 26.54
134.77 134.74
116.83 116.61
26.64 26.54
4f
^{13}C NMR (101 MHz, CDCl$_3$)
f1 (ppm)

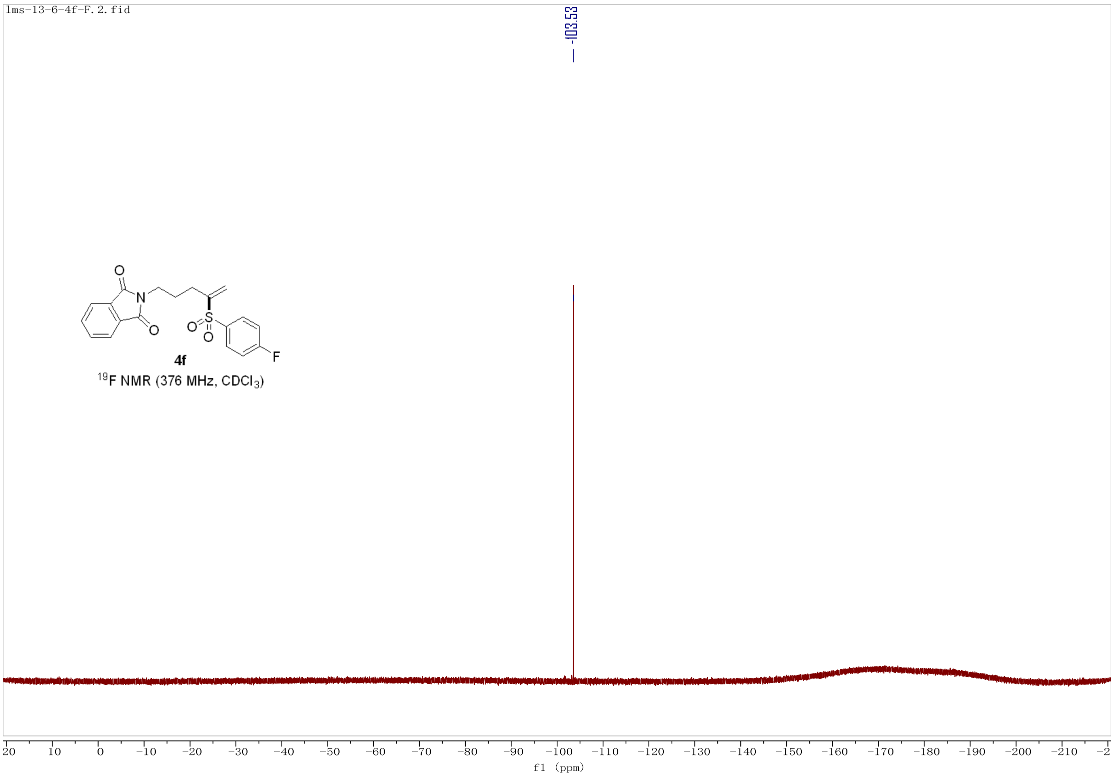
lms-13-6-4f-F.2.fid
-103.53
4f
^{19}F NMR (376 MHz, CDCl$_3$)
f1 (ppm)

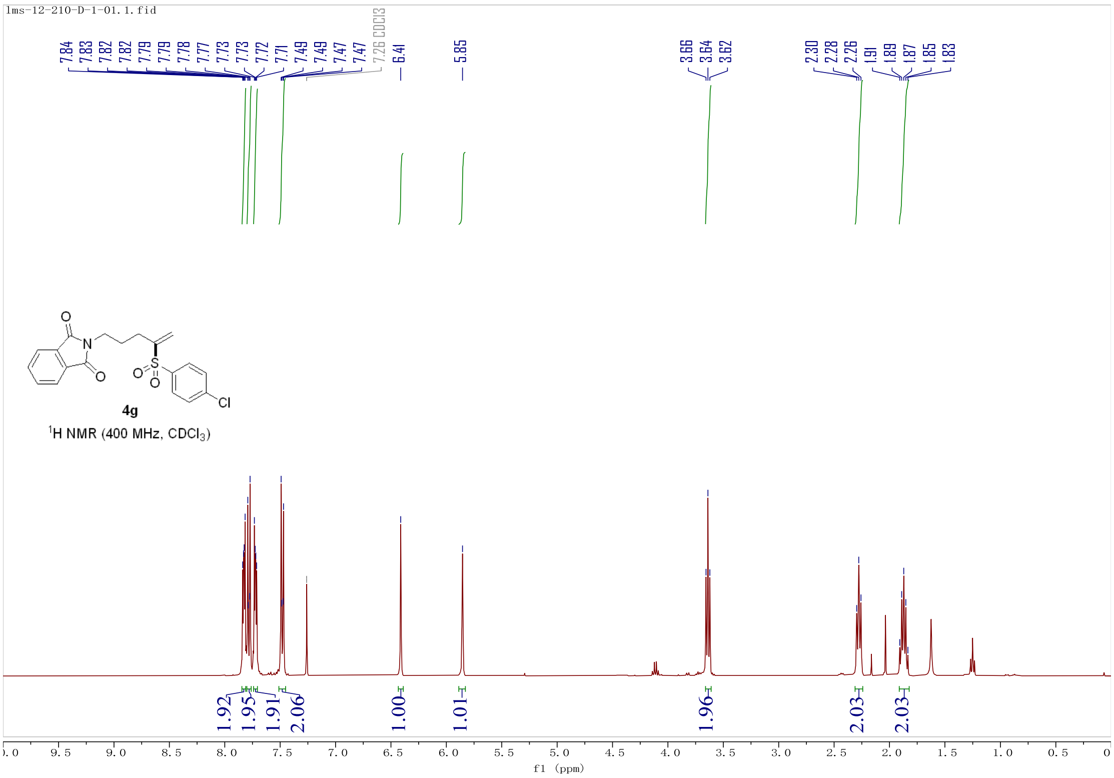
lms-12-210-D-1-01.1.fid
7.84 7.83 7.82 7.82 7.79 7.79 7.78 7.77 7.73 7.73 7.72 7.71 7.49 7.49 7.47 7.47 7.26 CDCl3 6.41 5.85
3.66 3.64 3.62
2.30 2.28 2.26 1.91 1.89 1.87 1.85 1.83
4g
^{1}H NMR (400 MHz, CDCl$_3$)
1.92 1.95 1.91 2.06 1.00 1.01 1.96 2.03 2.03
f1 (ppm)

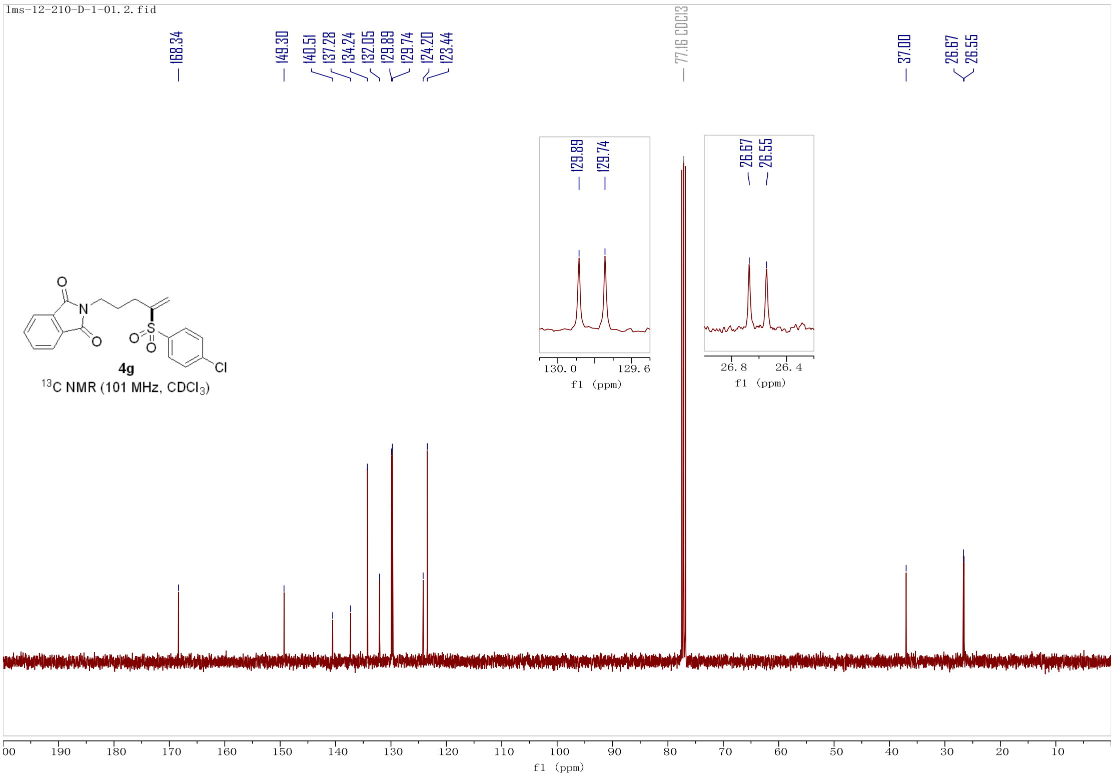
lms-12-210-D-1-01.2.fid
168.34
149.30
140.51
137.28
134.24
132.05
129.89
129.74
124.20
123.44
77.16 CDCl3
37.00
26.67
26.55
4g
^{13}C NMR (101 MHz, CDCl$_3$)
f1 (ppm)

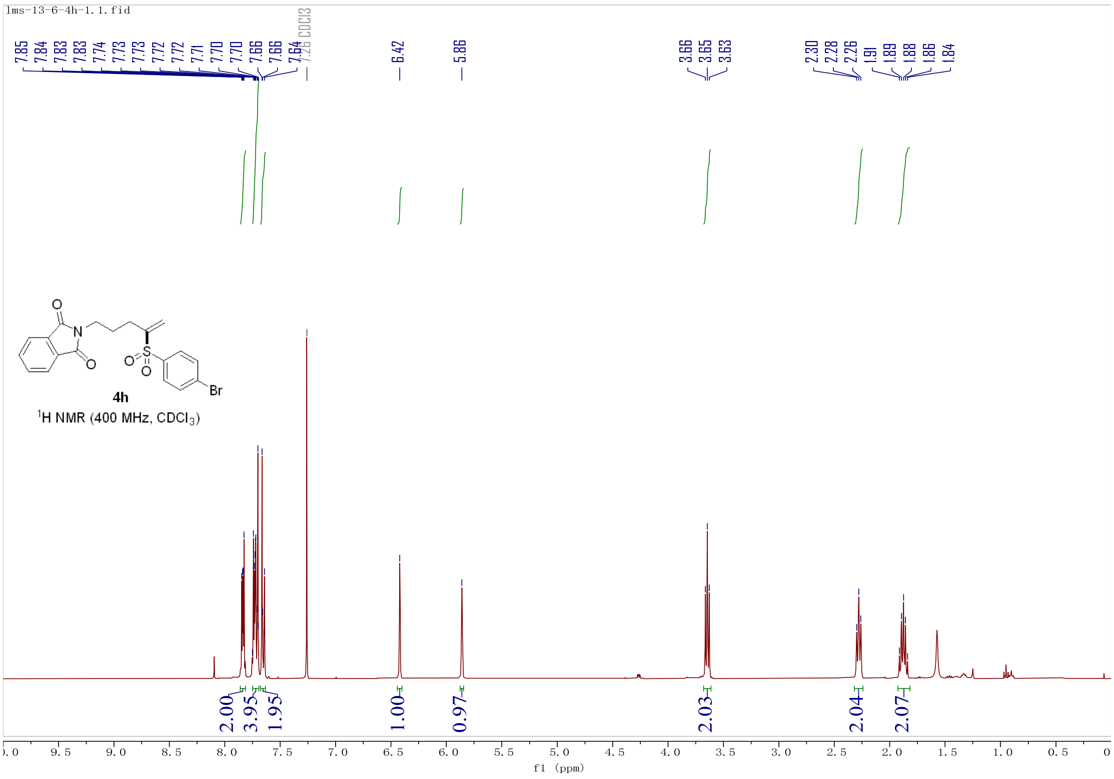
lms-13-6-4h-1.1.fid
7.85 7.84 7.83 7.83 7.74 7.73 7.73 7.72 7.72 7.71 7.70 7.70 7.66 7.66 7.64
7.26 CDCl3
6.42
5.86
3.66 3.65 3.63
2.30 2.28 2.26 1.91 1.89 1.88 1.86 1.84
4h
^{1}H NMR (400 MHz, CDCl$_3$)
2.00 3.95 1.95 1.00 0.97 2.03 2.04 2.07
f1 (ppm)

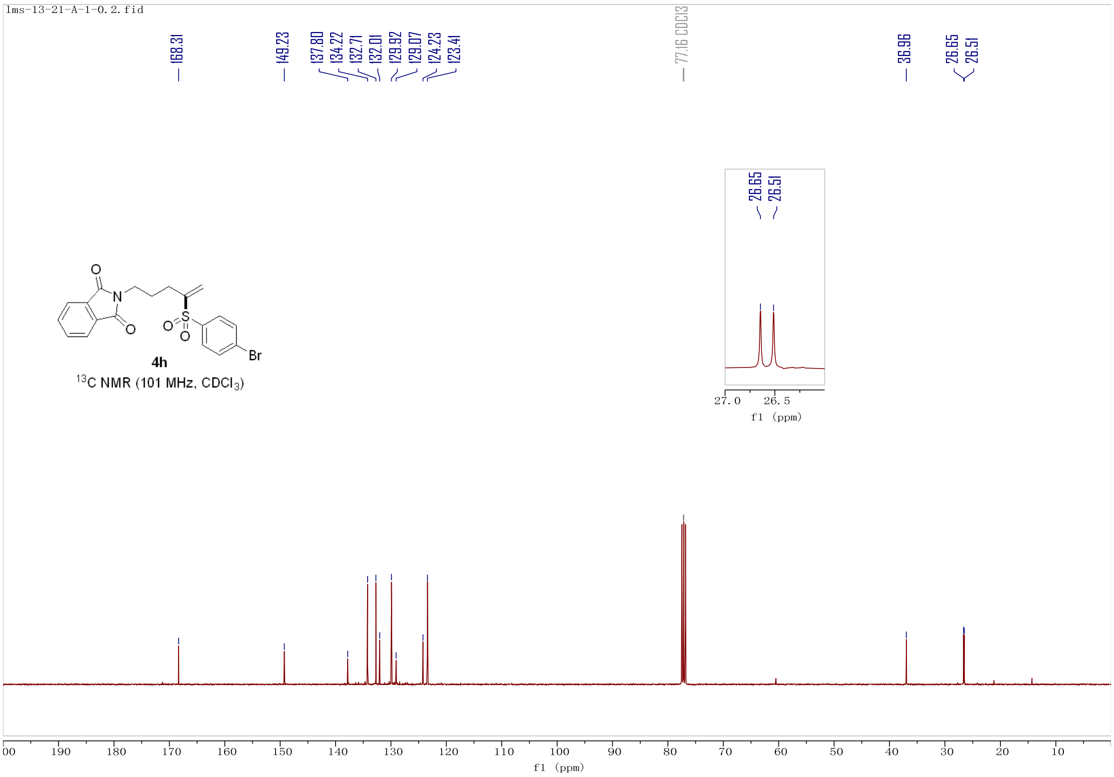
lms-13-21-A-1-0.2.fid
168.31
149.23
137.80
134.22
132.71
132.01
129.92
129.07
124.23
123.41
77.16 CDCl3
36.96
26.65
26.51
Br
4h
^{13}C NMR (101 MHz, $CDCl_3$)
f1 (ppm)

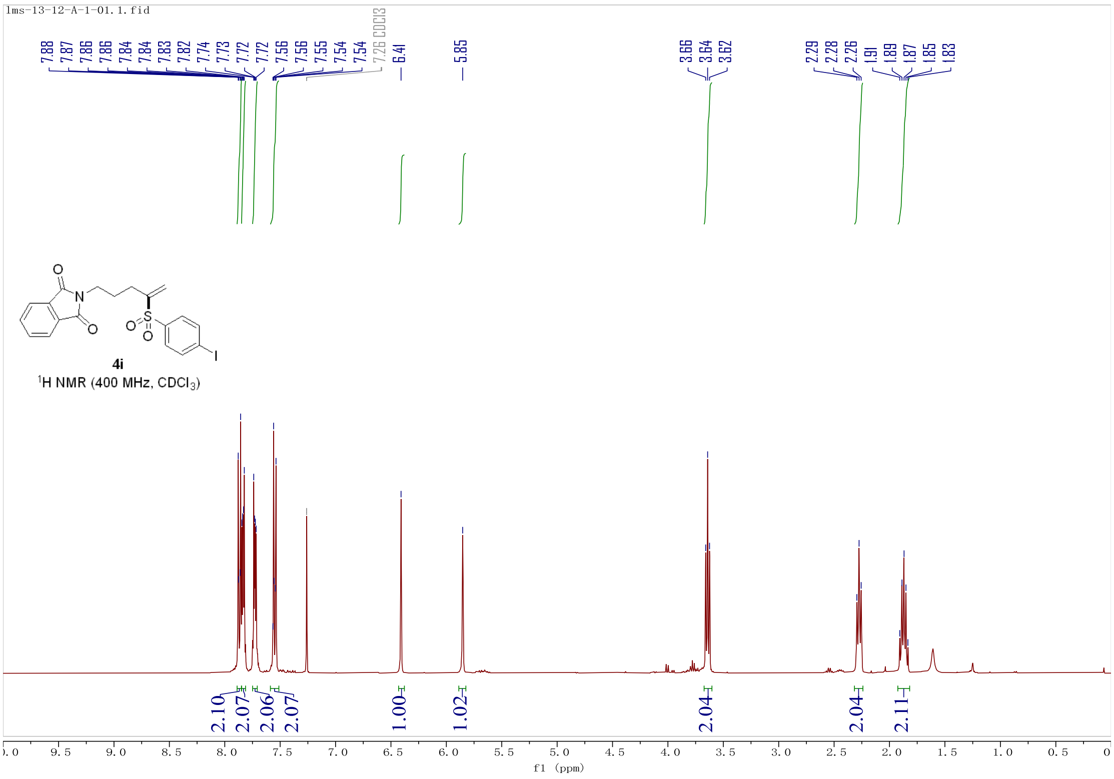
lms-13-12-A-1-01.1.fid
7.88
7.87
7.86
7.84
7.84
7.83
7.82
7.74
7.73
7.72
7.72
7.56
7.56
7.55
7.54
7.54
7.26 CDCl3
6.41
5.85
3.66
3.64
3.62
2.29
2.28
2.26
1.91
1.89
1.87
1.85
1.83
4i
^{1}H NMR (400 MHz, $CDCl_3$)
2.10
2.07
2.06
2.07
1.00
1.02
2.04
2.04
2.11
f1 (ppm)

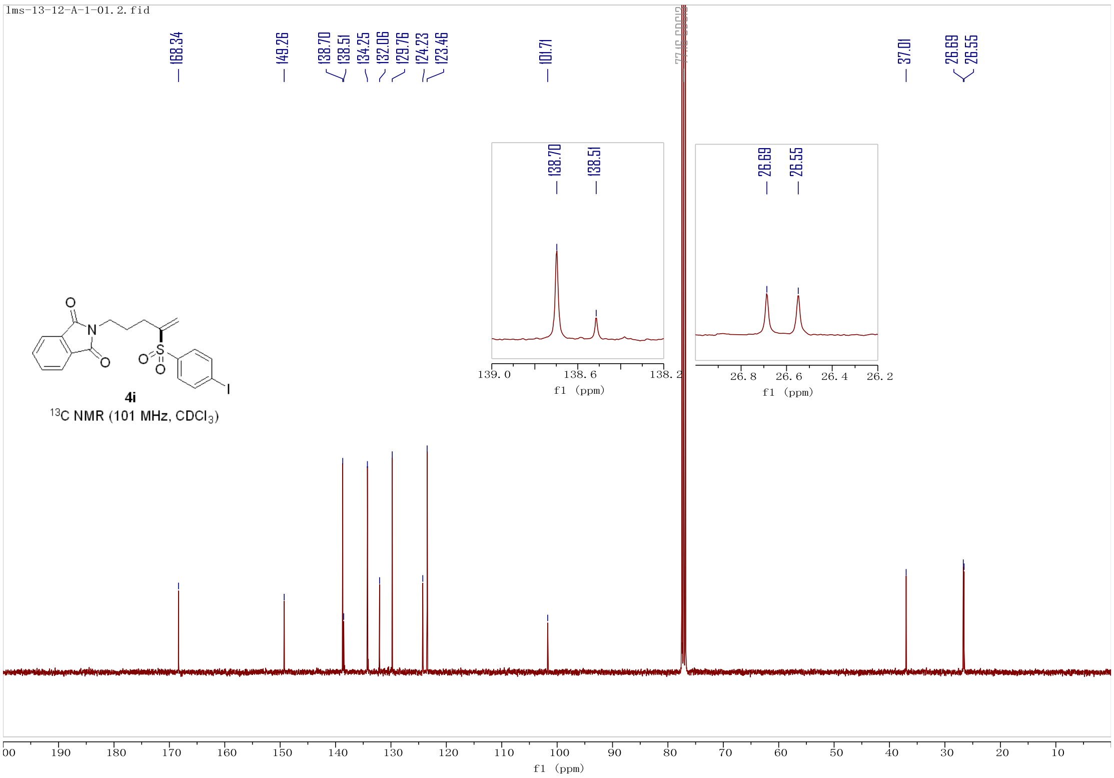
lms-13-12-A-1-01.2.fid
168.34
149.26
138.70
138.51
134.25
132.06
129.76
124.23
123.46
101.71
77.16 CDCl3
37.01
26.69
26.55
4i
^{13}C NMR (101 MHz, CDCl$_3$)
f1 (ppm)

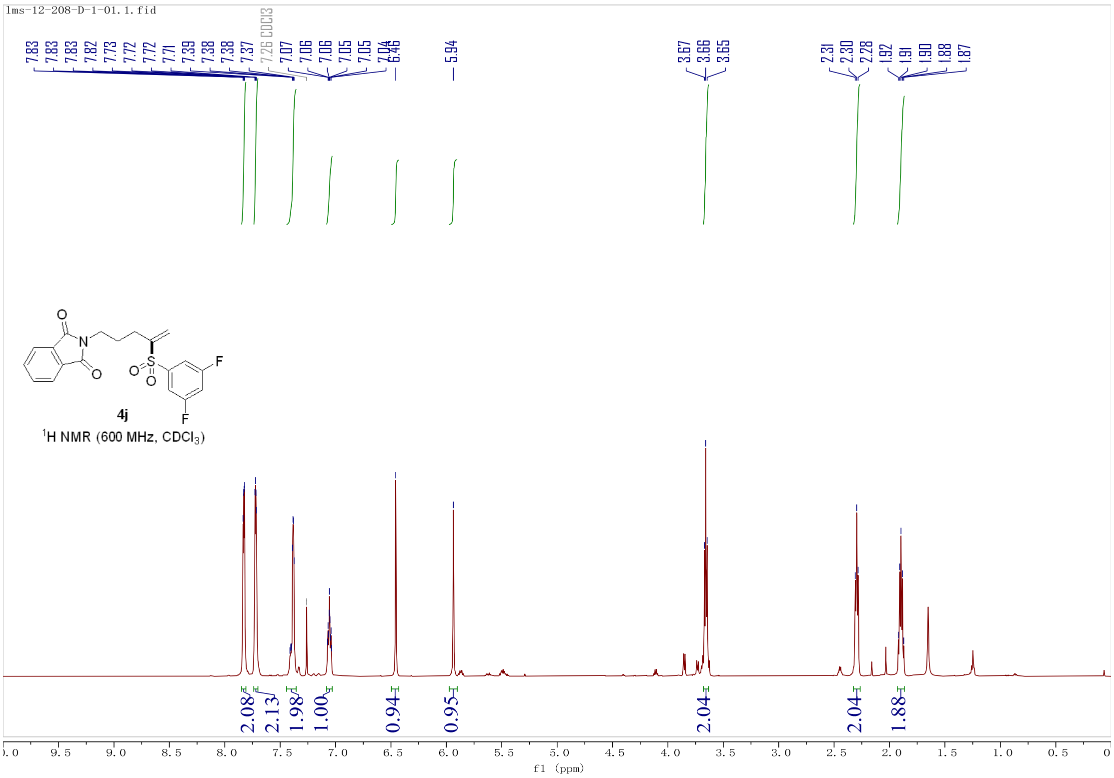
lms-12-208-D-1-01.1.fid
7.83
7.83
7.83
7.82
7.73
7.72
7.72
7.71
7.39
7.38
7.38
7.37
7.26 CDCl3
7.07
7.06
7.06
7.05
7.05
7.04
6.46
5.94
3.67
3.66
3.65
2.31
2.30
2.28
1.92
1.91
1.90
1.88
1.87
4j
^{1}H NMR (600 MHz, CDCl$_3$)
2.08
2.13
1.98
1.00
0.94
0.95
2.04
2.04
1.88
f1 (ppm)

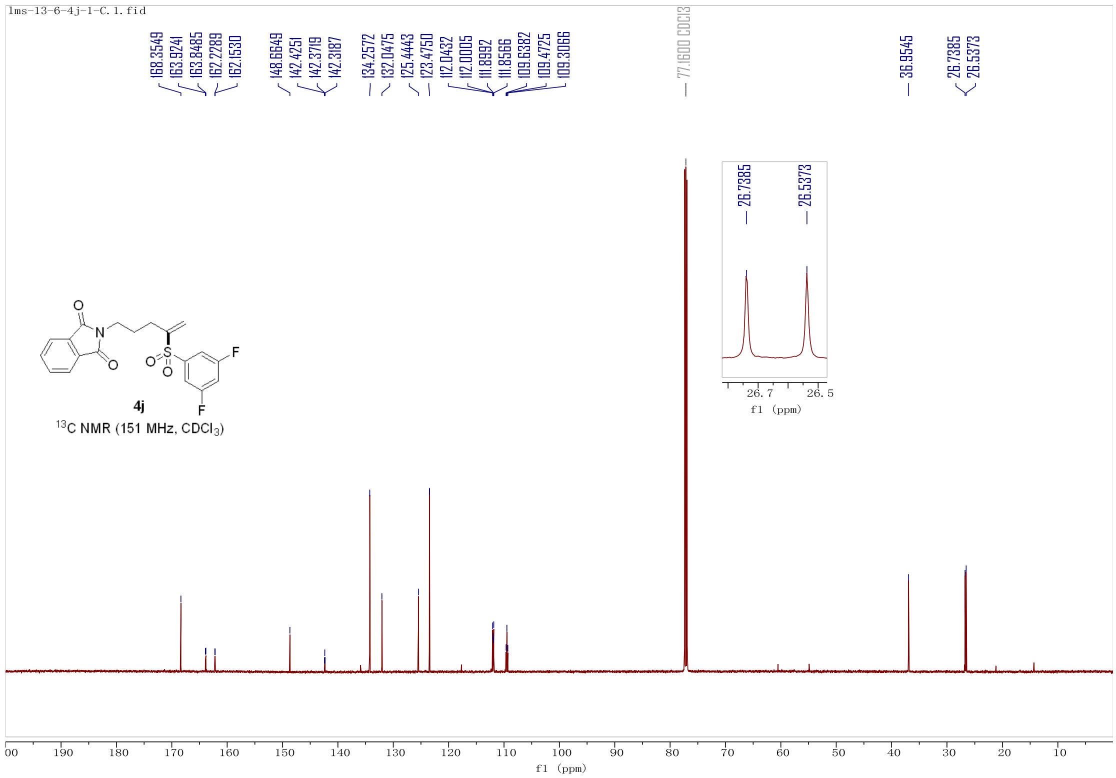
lms-13-6-4j-1-C.1.fid
168.3549
163.9241
163.8485
162.2289
162.1530
148.6649
142.4251
142.3719
142.3187
134.2572
132.0475
125.4443
123.4750
112.0432
112.0005
111.8992
111.8566
109.6382
109.4725
109.3066
77.1600 CDCl3
36.9545
26.7385
26.5373
4j
^{13}C NMR (151 MHz, CDCl$_3$)
f1 (ppm)

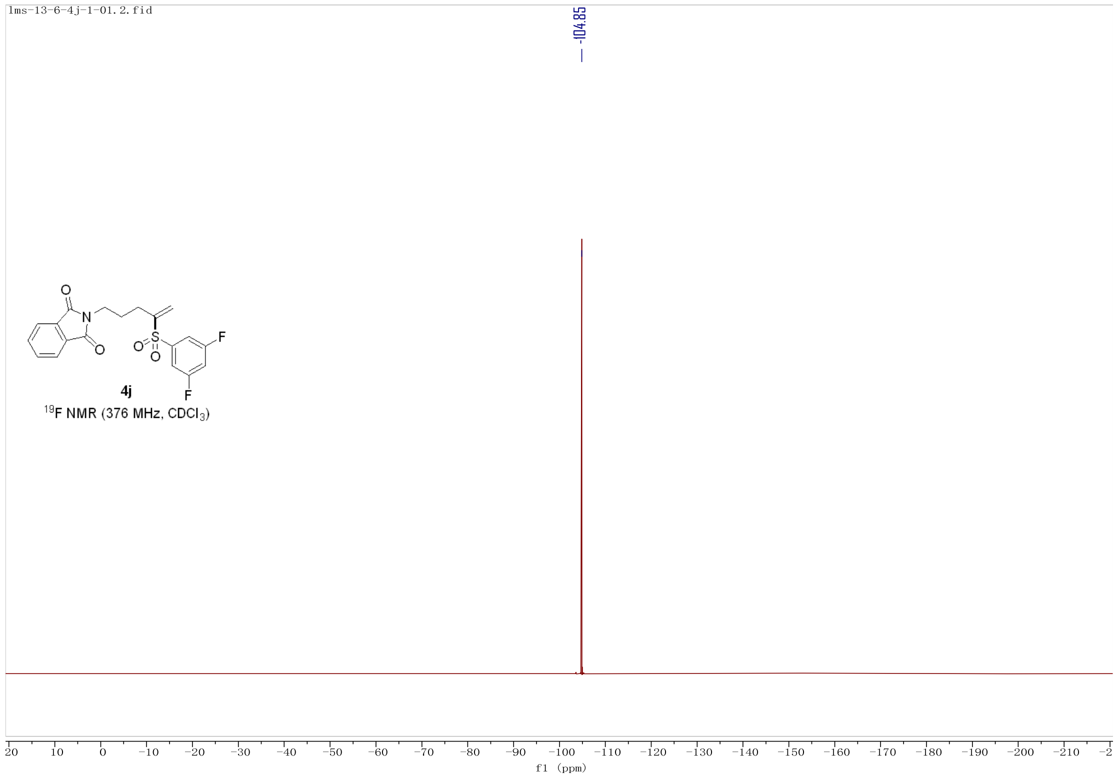
lms-13-6-4j-1-01.2.fid
-104.85
4j
^{19}F NMR (376 MHz, CDCl$_3$)
f1 (ppm)

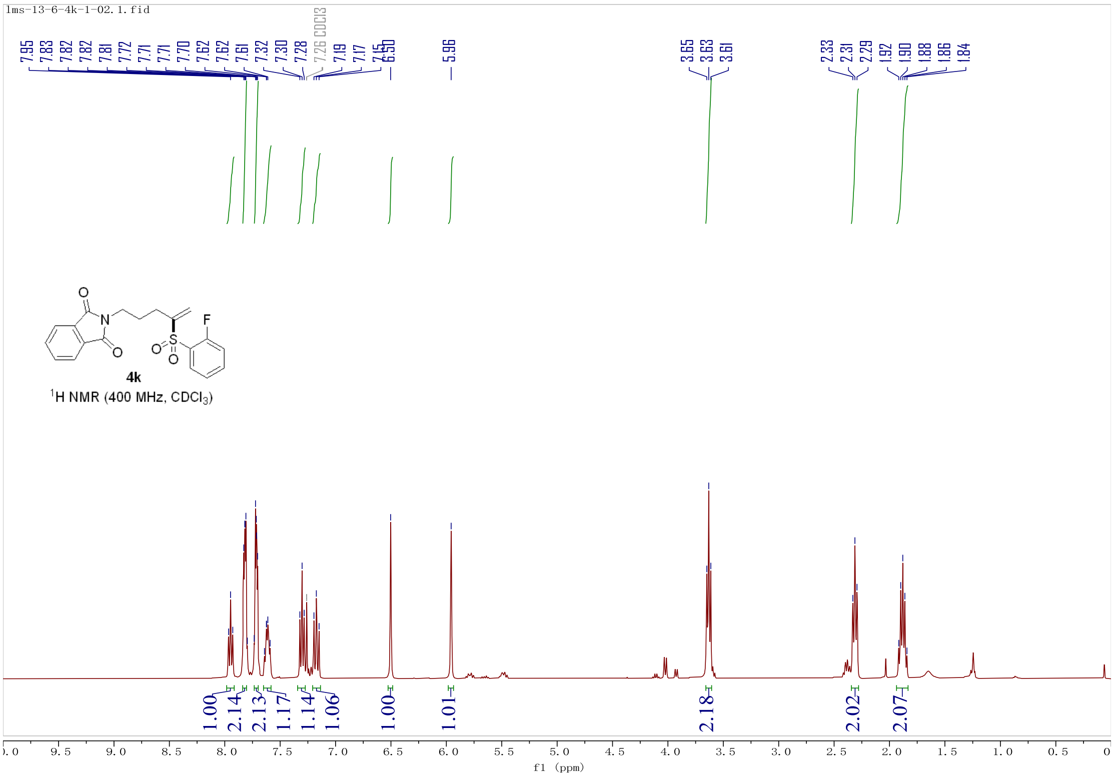
lms-13-6-4k-1-02.1.fid
7.95 7.83 7.82 7.82 7.81 7.72 7.71 7.71 7.70 7.62 7.62 7.61 7.32 7.30 7.28 7.26 CDCl3 7.19 7.17 7.15 6.50
5.96
3.65 3.63 3.61
2.33 2.31 2.29 1.92 1.90 1.88 1.86 1.84
O
N
O
F
O
S
O
4k
^{1}H NMR (400 MHz, $CDCl_3$)
1.00 2.14 2.13 1.17 1.14 1.06 1.00 1.01 2.18 2.02 2.07
9.5 9.0 8.5 8.0 7.5 7.0 6.5 6.0 5.5 5.0 4.5 4.0 3.5 3.0 2.5 2.0 1.5 1.0 0.5 0
f1 (ppm)

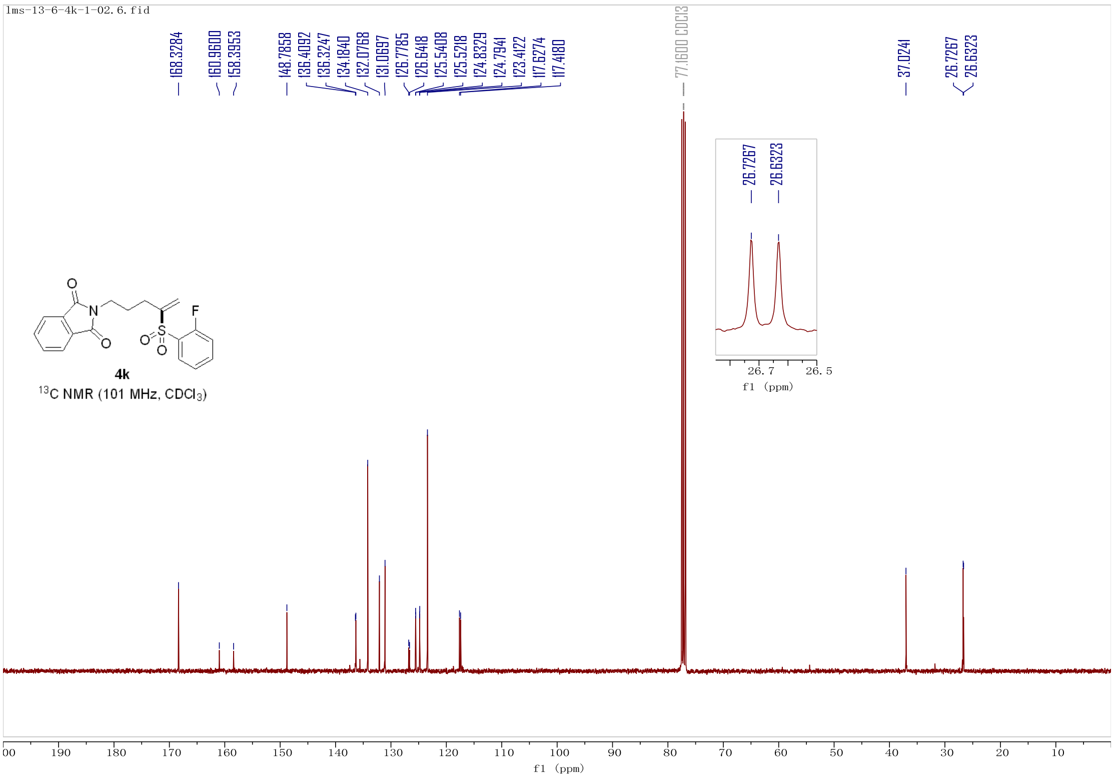
lms-13-6-4k-1-02.6.fid
168.3284
160.9600 158.3953
148.7858 136.4092 136.3247 134.1840 132.0768 131.0697 126.7785 126.6418 125.5408 125.5218 124.8329 124.7941 123.4122 117.6274 117.4180
77.1600 CDCl3
37.0241
26.7267 26.6323
26.7267
26.6323
26.7 26.5
f1 (ppm)
O
N
O
F
O
S
O
4k
^{13}C NMR (101 MHz, $CDCl_3$)
190 180 170 160 150 140 130 120 110 100 90 80 70 60 50 40 30 20 10
f1 (ppm)

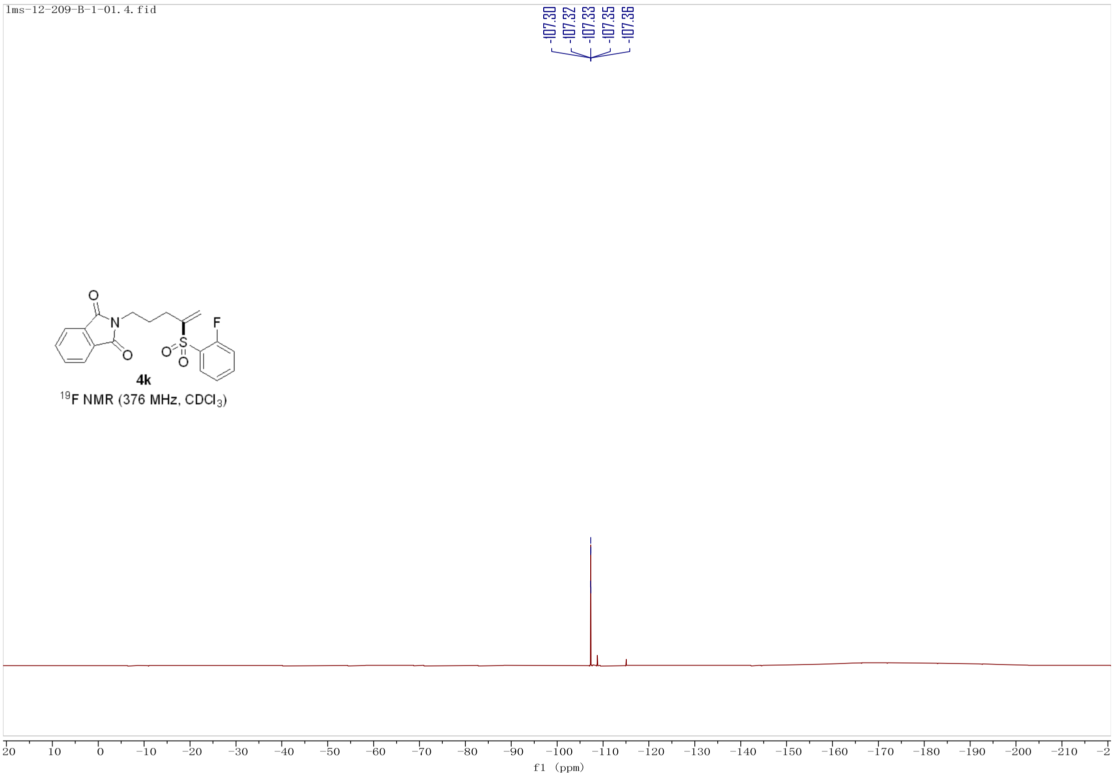
lms-12-209-B-1-01.4.fid
-107.30
-107.32
-107.33
-107.35
-107.36
F
O
N
S
4k
^{19}F NMR (376 MHz, $CDCl_3$)
f1 (ppm)

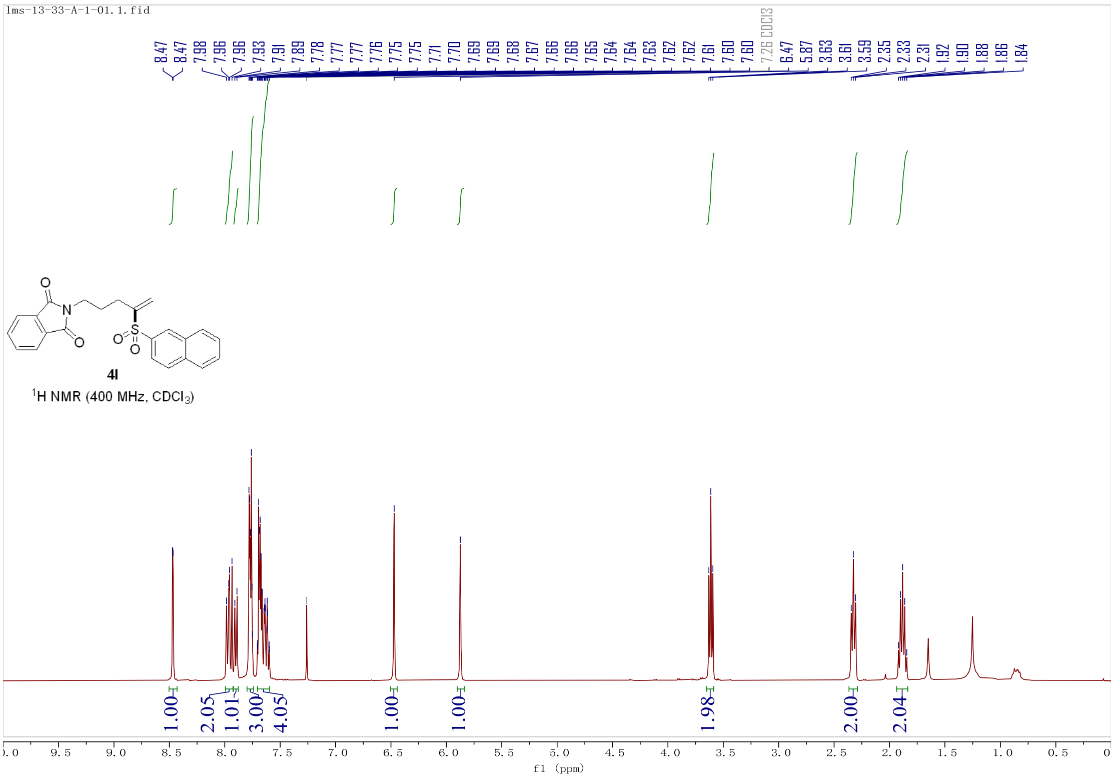
lms-13-33-A-1-01.1.fid
8.47
8.47
7.98
7.96
7.93
7.91
7.89
7.78
7.77
7.77
7.76
7.75
7.75
7.71
7.70
7.69
7.69
7.68
7.67
7.66
7.65
7.64
7.64
7.63
7.62
7.62
7.61
7.60
7.60
7.26 CDCl3
6.47
5.87
3.63
3.61
3.59
2.35
2.33
2.31
1.92
1.90
1.88
1.86
1.84
O
N
S
4l
^{1}H NMR (400 MHz, $CDCl_3$)
1.00
2.05
1.01
3.00
4.05
1.00
1.00
1.98
2.00
2.04
f1 (ppm)

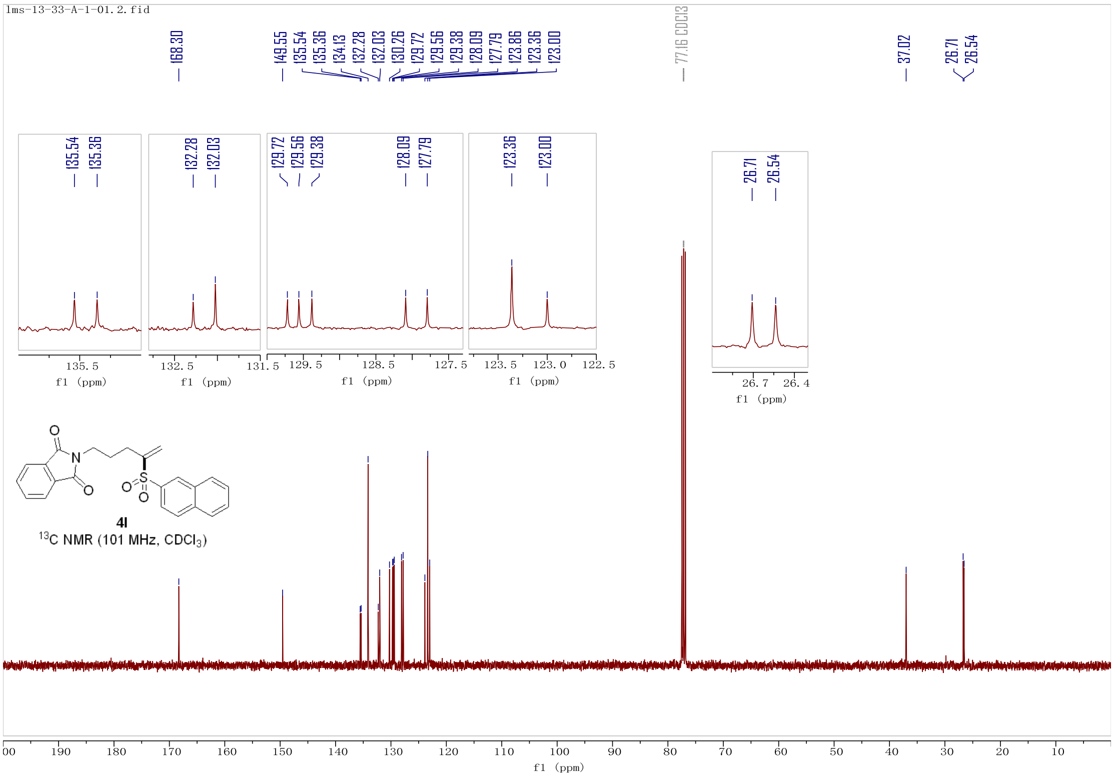
lms-13-33-A-1-01.2.fid
168.30
149.55
135.54
135.36
134.13
132.28
132.03
130.26
129.72
129.56
129.38
128.09
127.79
123.86
123.36
123.00
77.16 CDCl3
37.02
26.71
26.54
4l
13C NMR (101 MHz, CDCl3)
f1 (ppm)

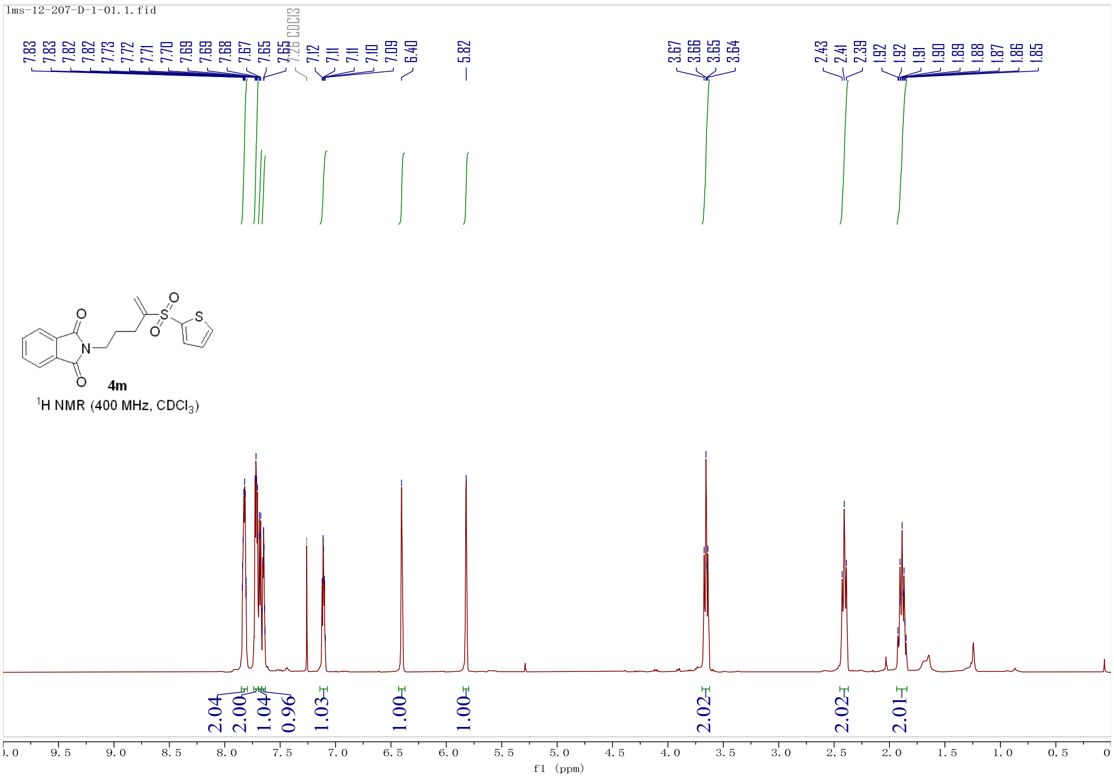
lms-12-207-D-1-01.1.fid
7.83
7.83
7.82
7.82
7.73
7.72
7.71
7.70
7.69
7.69
7.68
7.67
7.65
7.26 CDCl3
7.12
7.11
7.11
7.10
7.09
6.40
5.82
3.67
3.66
3.65
3.64
2.43
2.41
2.39
1.92
1.92
1.91
1.90
1.89
1.88
1.87
1.86
1.85
2.04
2.00
1.04
0.96
1.03
1.00
1.00
2.02
2.02
2.01
4m
1H NMR (400 MHz, CDCl3)
f1 (ppm)

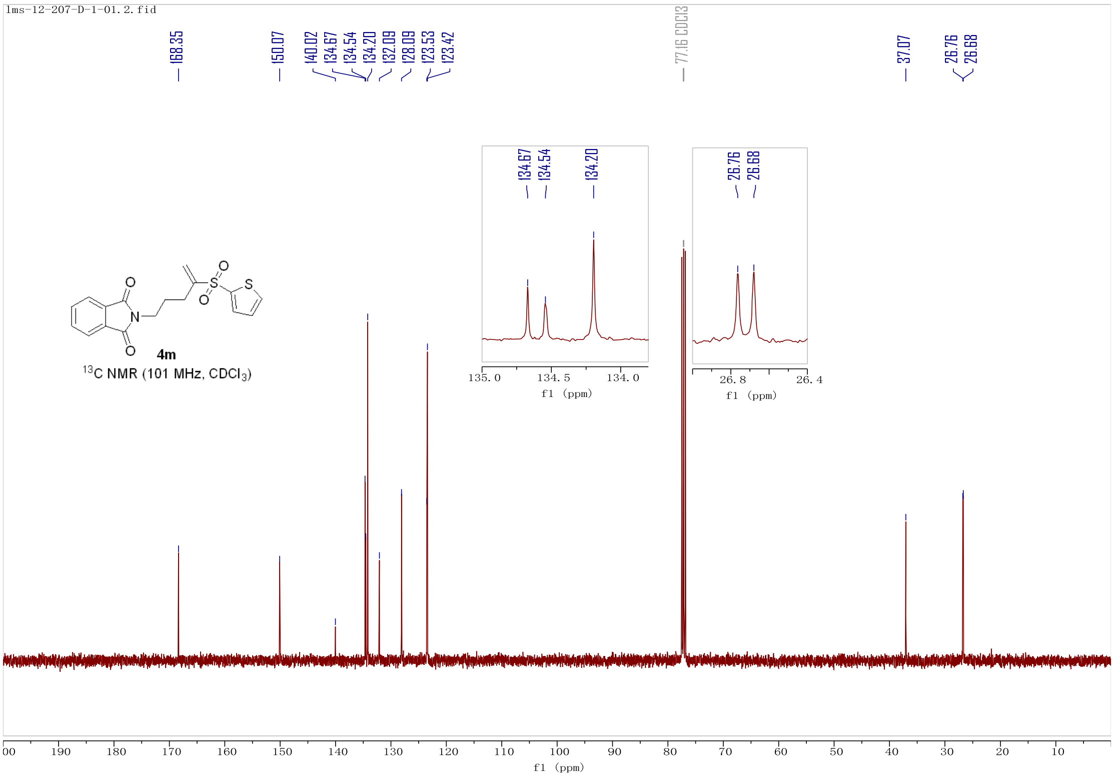
lms-12-207-D-1-01.2.fid
168.35
150.07
140.02
134.67
134.54
134.20
132.09
128.09
123.53
123.42
77.16 CDCl3
37.07
26.76
26.68
4m
13C NMR (101 MHz, CDCl3)
f1 (ppm)

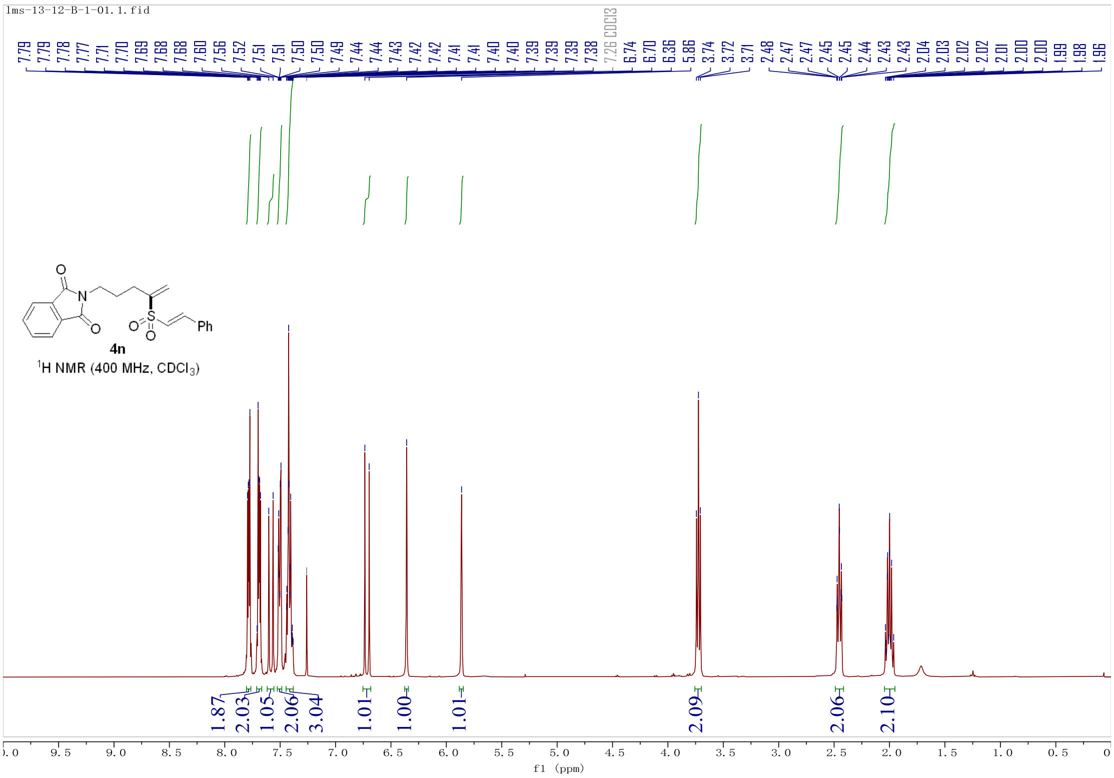
lms-13-12-B-1-01.1.fid
7.26 CDCl3
3.74
3.72
3.71
4n
Ph
1H NMR (400 MHz, CDCl3)
1.87
2.03
1.05
2.06
3.04
1.01
1.00
1.01
2.09
2.06
2.10
f1 (ppm)

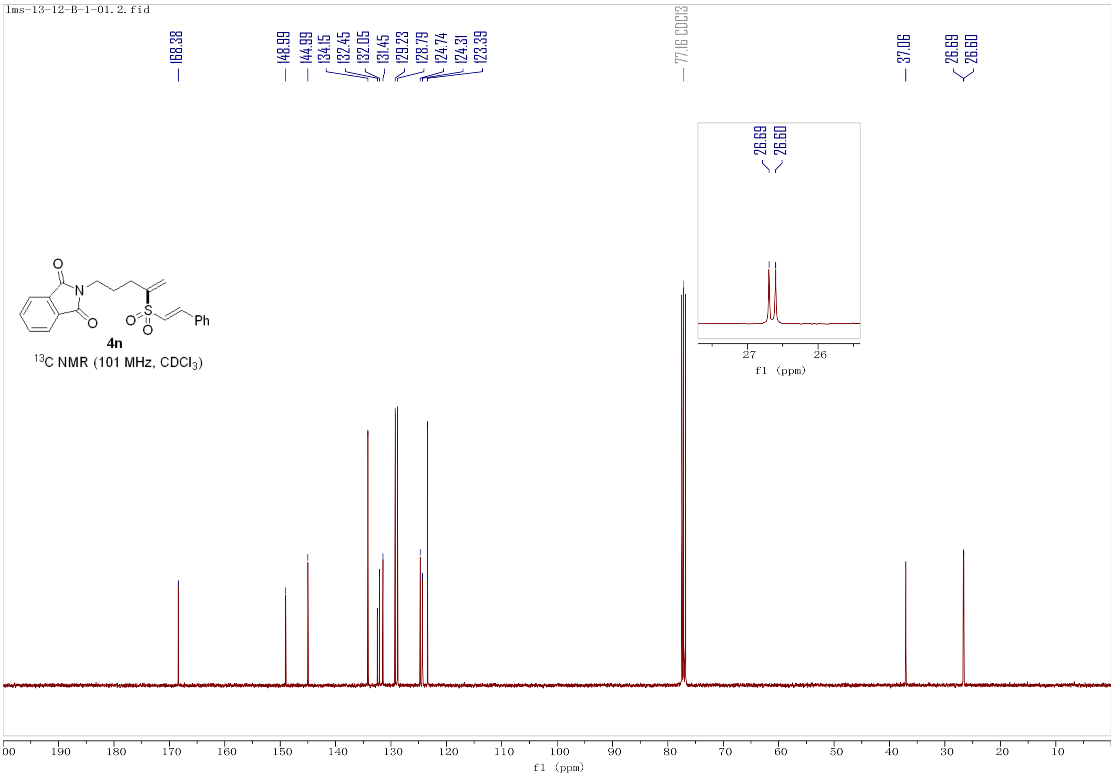
lms-13-12-B-1-01.2.fid
168.38
148.99
144.99
134.15
132.45
132.05
131.45
129.23
128.79
124.74
124.31
123.39
77.16 CDCl3
37.06
26.69
26.60
4n
13C NMR (101 MHz, CDCl3)
f1 (ppm)

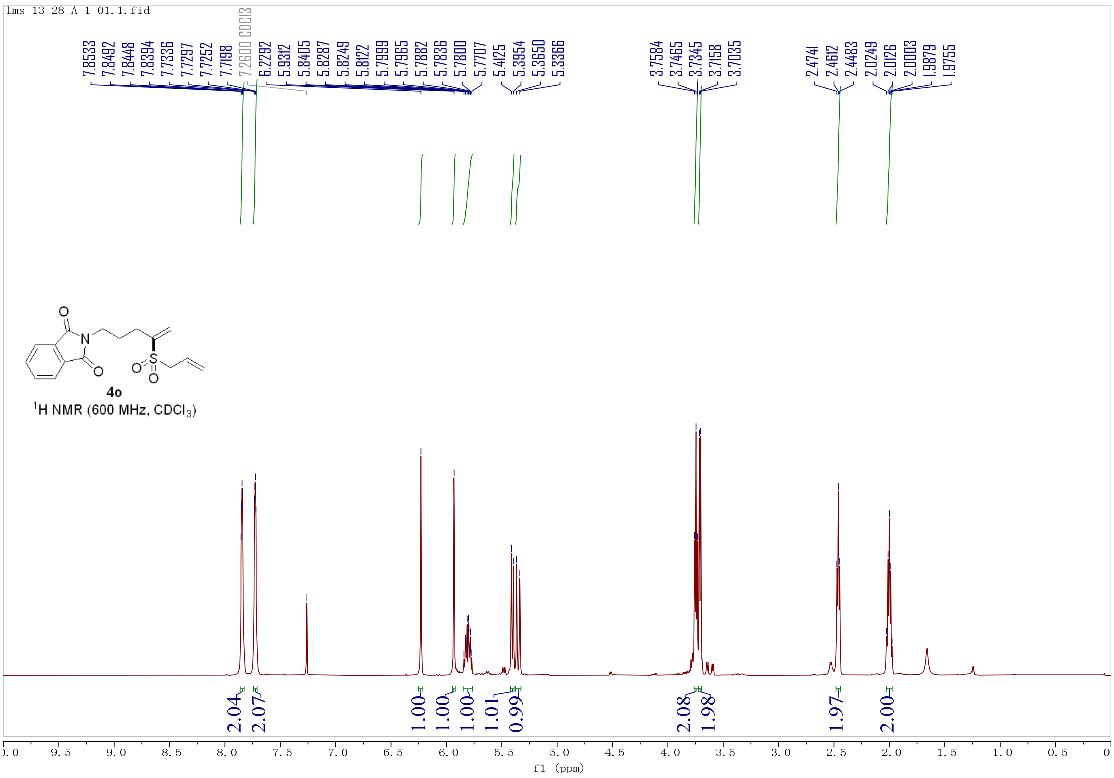
lms-13-28-A-1-01.1.fid
7.8533
7.8492
7.8448
7.8394
7.7336
7.7297
7.7252
7.7198
7.2600 CDCl3
6.2292
5.9312
5.8405
5.8287
5.8249
5.8122
5.7999
5.7965
5.7882
5.7836
5.7800
5.7707
5.4125
5.3954
5.3650
5.3366
3.7584
3.7465
3.7345
3.7158
3.7035
2.4741
2.4612
2.4483
2.0249
2.0126
2.0003
1.9879
1.9755
4o
1H NMR (600 MHz, CDCl3)
2.04
2.07
1.00
1.00
1.00
1.01
0.99
2.08
1.98
1.97
2.00
f1 (ppm)

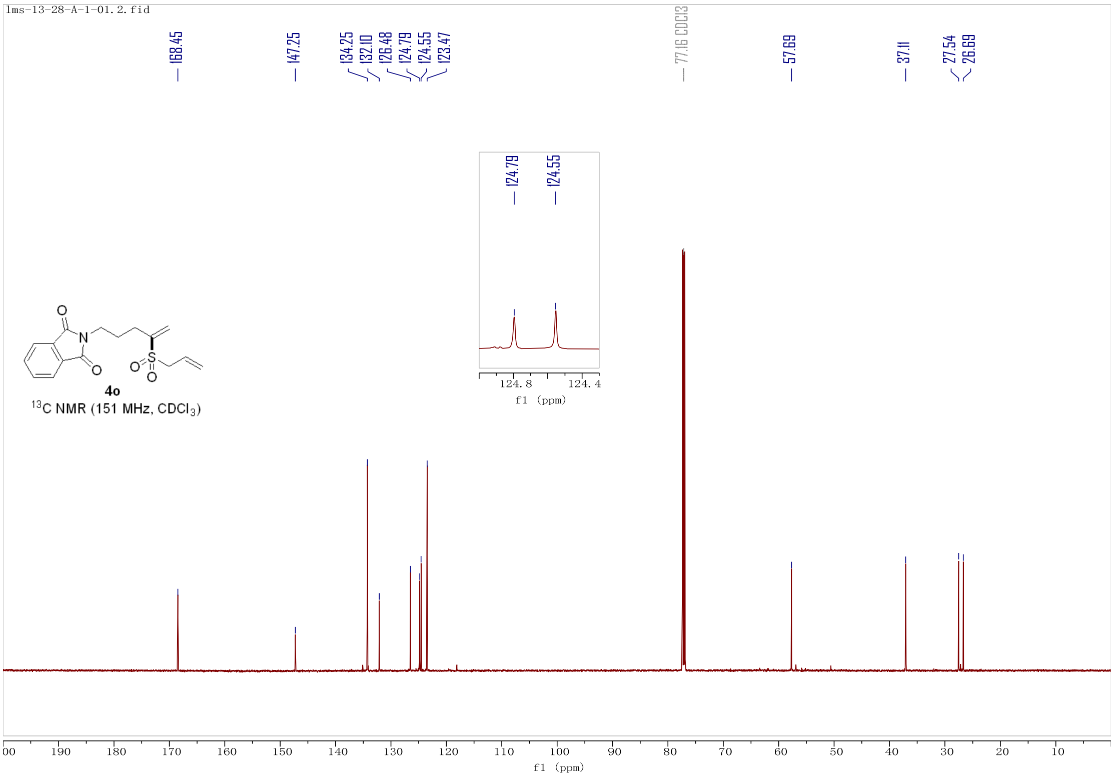
lms-13-28-A-1-01.2.fid
168.45
147.25
134.25
132.10
126.48
124.79
124.55
123.47
77.16 CDCl3
57.69
37.11
27.54
26.69
4o
13C NMR (151 MHz, CDCl3)
f1 (ppm)

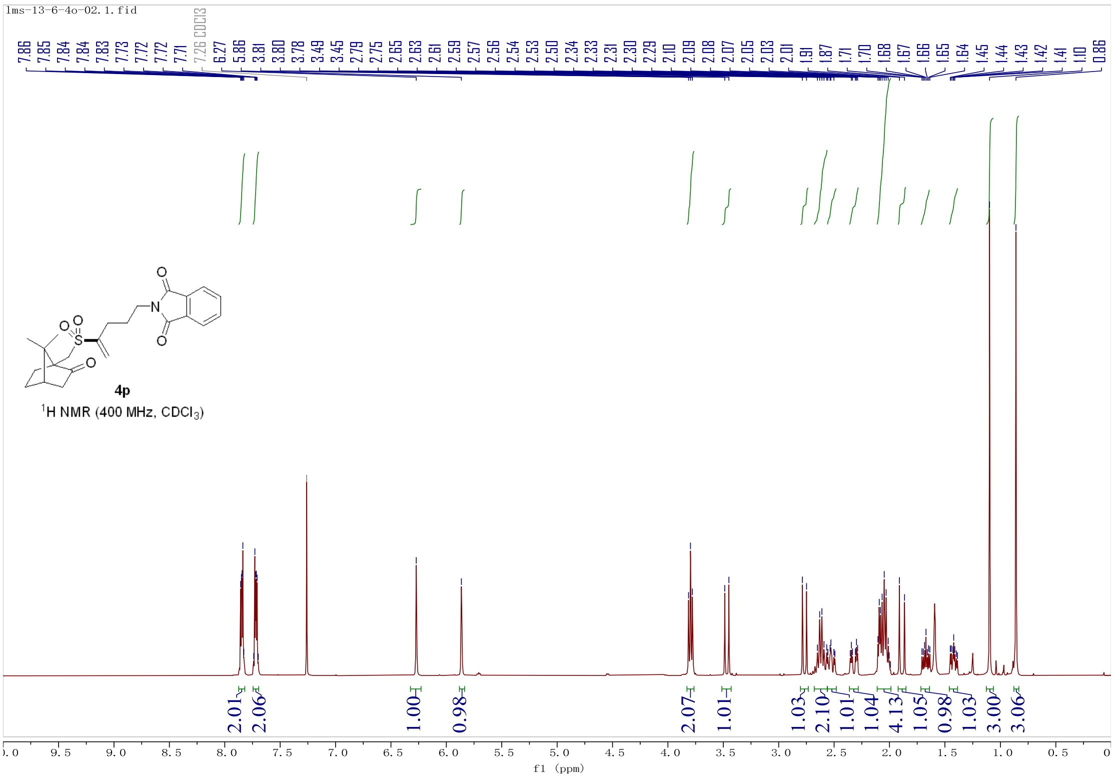
lms-13-6-4o-02.1.fid
4p
1H NMR (400 MHz, CDCl3)
f1 (ppm)

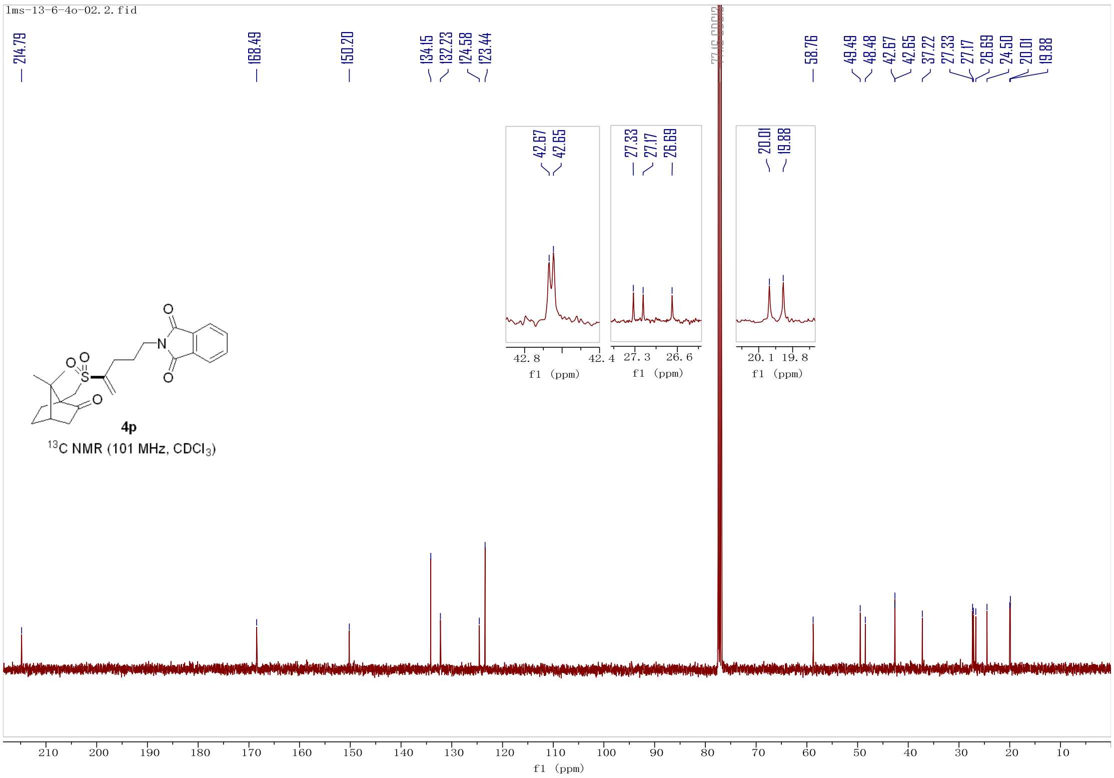
lms-13-6-4o-02.2.fid
214.79
168.49
150.20
134.15
132.23
124.58
123.44
77.16 CDCl3
58.76
49.49
48.48
42.67
42.65
37.22
27.33
27.17
26.69
24.50
20.01
19.88
4p
^{13}C NMR (101 MHz, CDCl$_3$)
f1 (ppm)

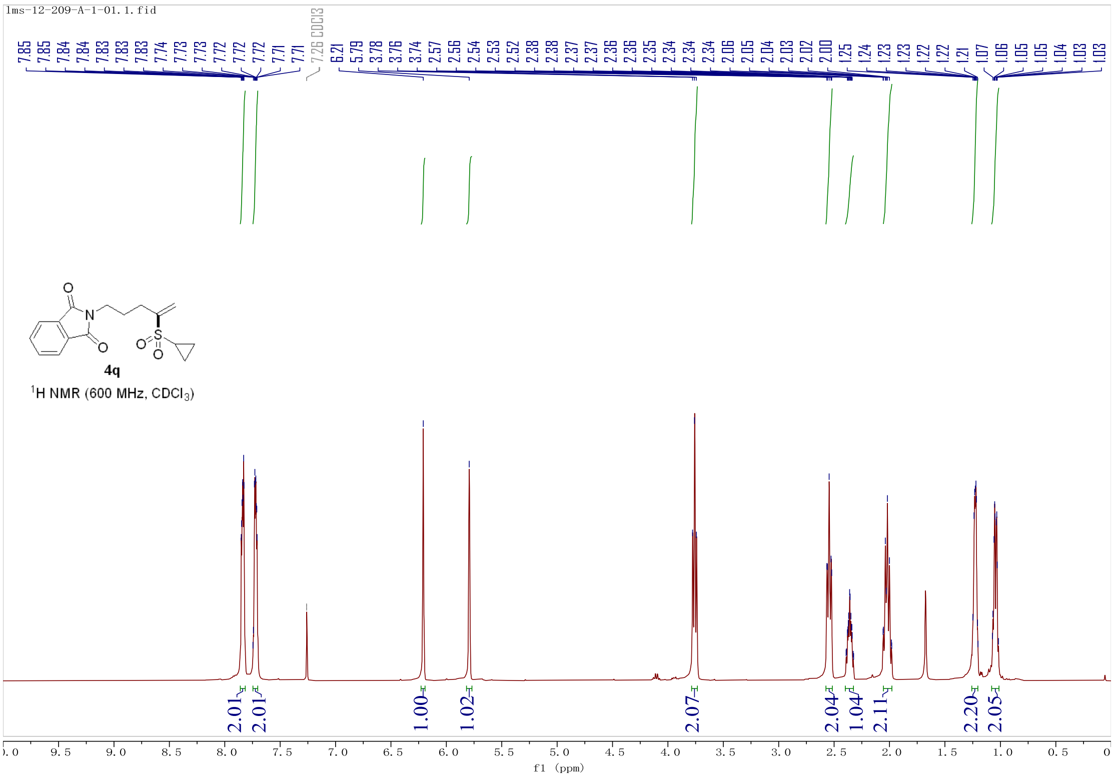
lms-12-209-A-1-01.1.fid
7.26 CDCl3
4q
^{1}H NMR (600 MHz, CDCl$_3$)
2.01
2.01
1.00
1.02
2.07
2.04
1.04
2.11
2.20
2.05
f1 (ppm)

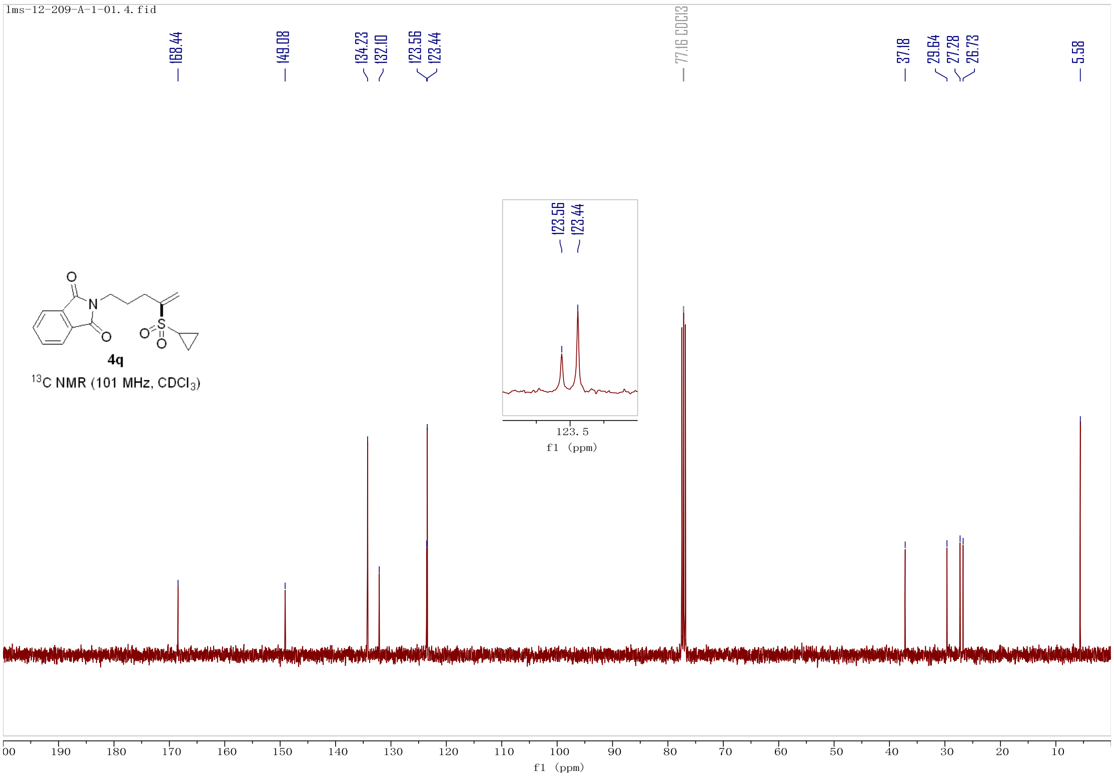

lms-12-209-A-1-01.4.fid
168.44
149.08
134.23
132.10
123.56
123.44
77.16 CDCl3
37.18
29.64
27.28
26.73
5.58
4q
13C NMR (101 MHz, CDCl3)
123.56
123.44
123.5
f1 (ppm)
00 190 180 170 160 150 140 130 120 110 100 90 80 70 60 50 40 30 20 10
f1 (ppm)

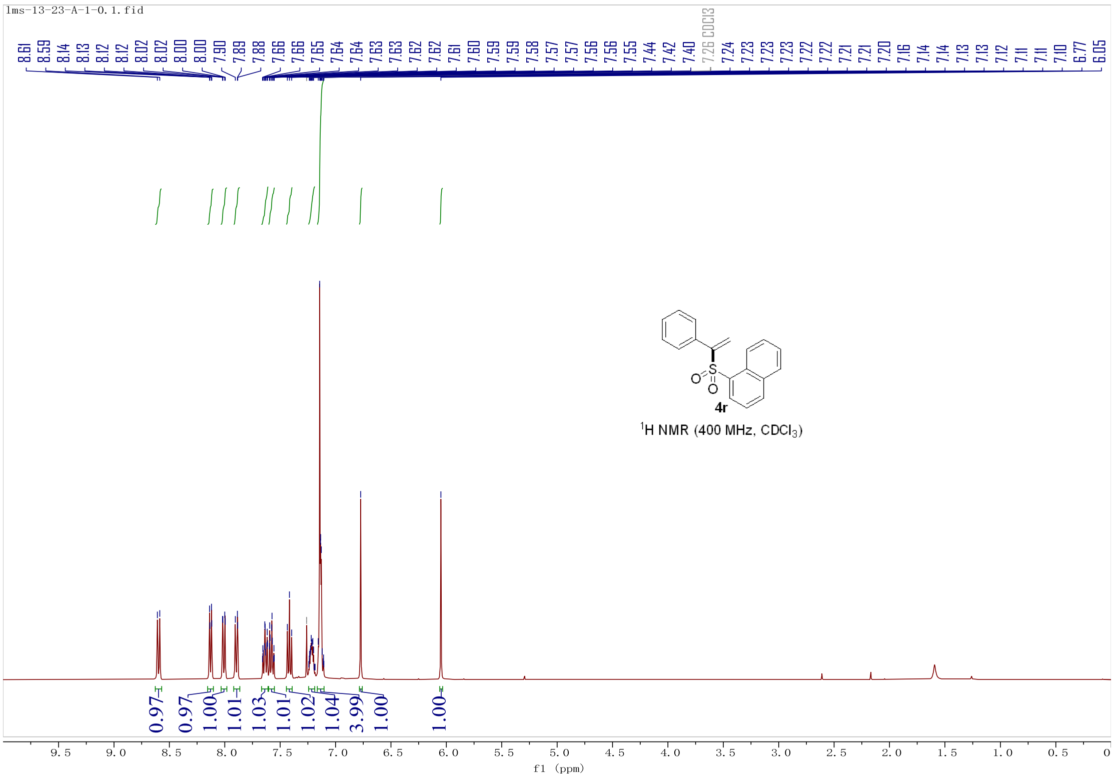

lms-13-23-A-1-0.1.fid
8.61 8.59 8.14 8.13 8.12 8.12 8.02 8.02 8.00 8.00 7.90 7.89 7.88 7.66 7.65 7.64 7.64 7.63 7.63 7.62 7.62 7.61 7.60 7.59 7.59 7.58 7.57 7.57 7.56 7.56 7.55 7.44 7.42 7.40 7.26 CDCl3 7.24 7.23 7.23 7.23 7.22 7.22 7.21 7.21 7.20 7.16 7.14 7.14 7.13 7.13 7.12 7.11 7.11 7.10 6.77 6.05
4r
1H NMR (400 MHz, CDCl3)
0.97 0.97 1.00 1.01 1.03 1.01 1.02 1.04 3.99 1.00 1.00
9.5 9.0 8.5 8.0 7.5 7.0 6.5 6.0 5.5 5.0 4.5 4.0 3.5 3.0 2.5 2.0 1.5 1.0 0.5 0
f1 (ppm)

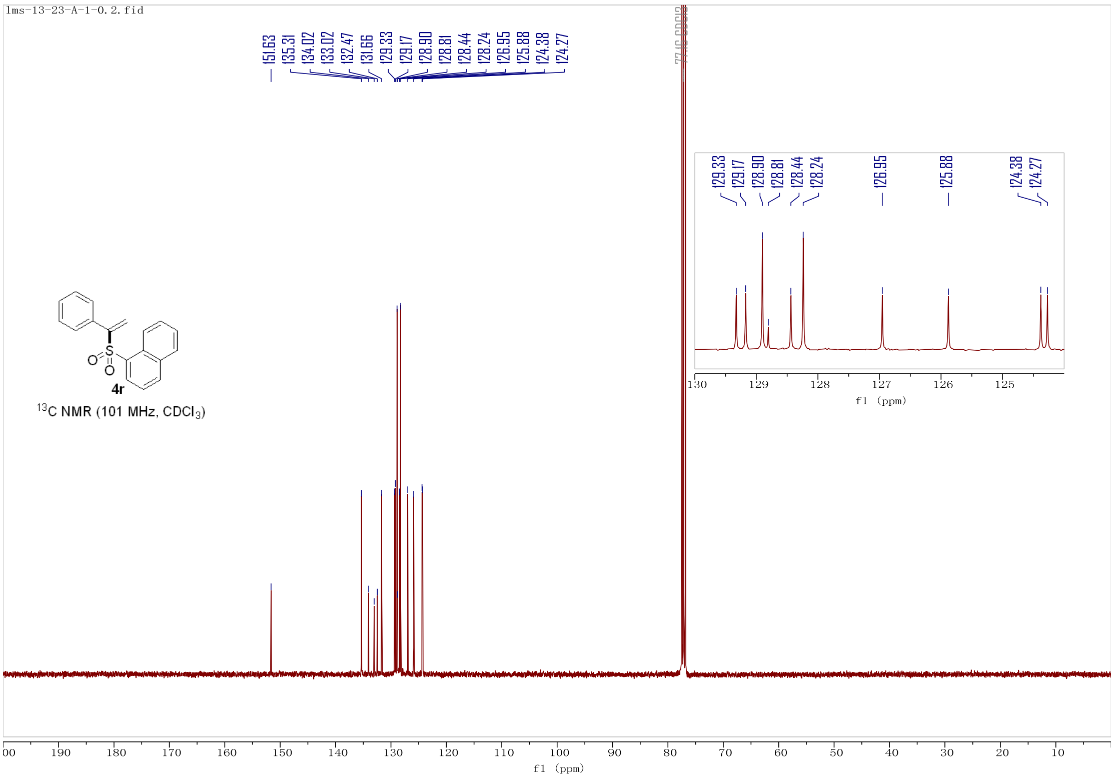
lms-13-23-A-1-0.2.fid
151.63
135.31
134.02
133.02
132.47
131.66
129.33
129.17
128.90
128.81
128.44
128.24
126.95
125.88
124.38
124.27
4r
13C NMR (101 MHz, CDCl3)
f1 (ppm)

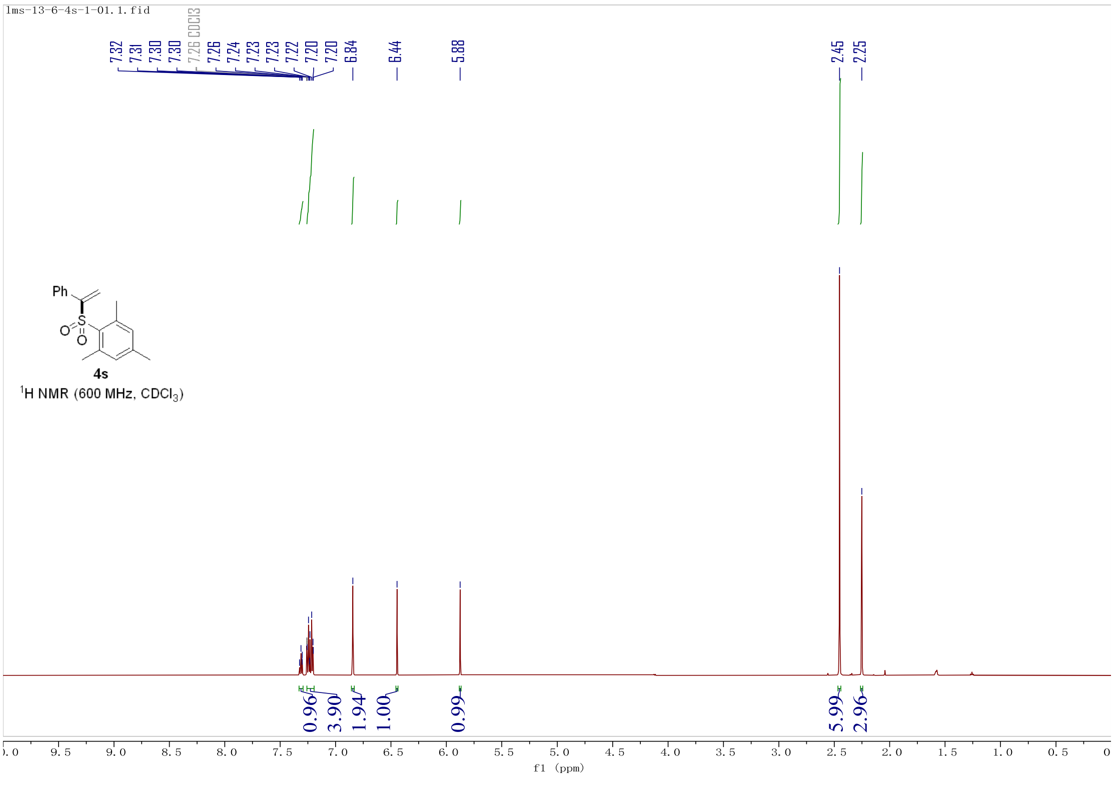
lms-13-6-4s-1-01.1.fid
7.32
7.31
7.30
7.30
7.26 CDCl3
7.26
7.24
7.23
7.23
7.22
7.20
7.20
6.84
6.44
5.88
2.45
2.25
Ph
4s
1H NMR (600 MHz, CDCl3)
0.96
3.90
1.94
1.00
0.99
5.99
2.96
f1 (ppm)

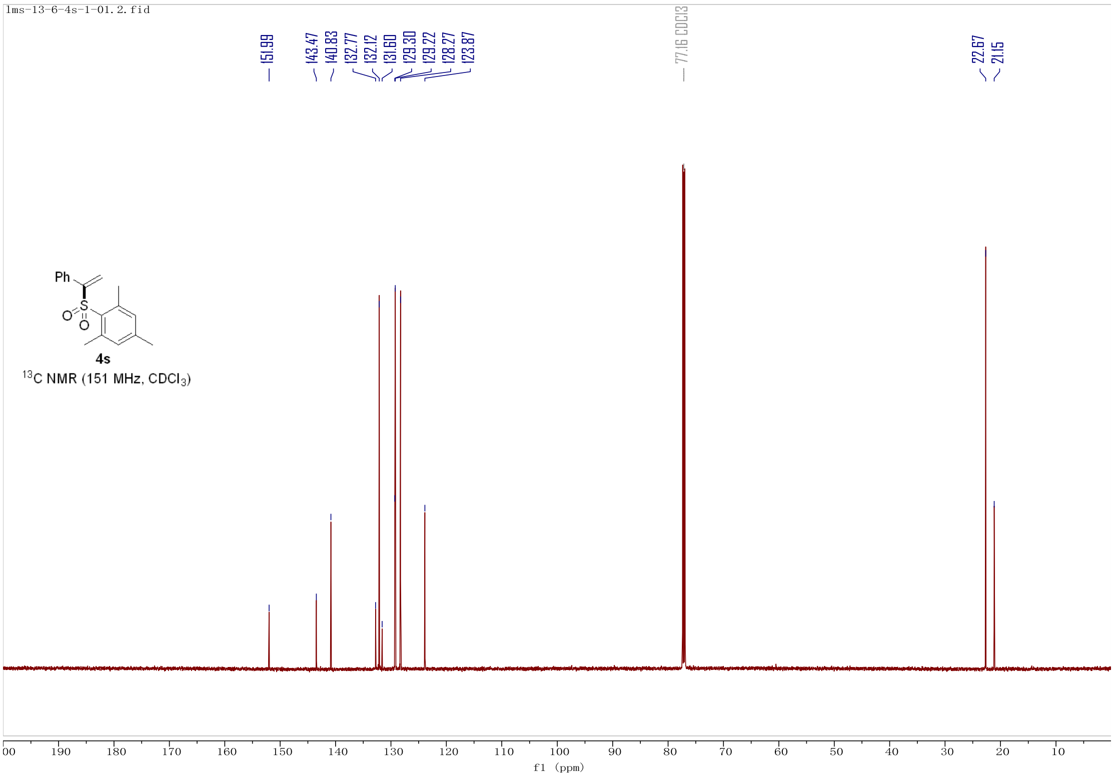
lms-13-6-4s-1-01.2.fid
151.99
143.47
140.83
132.77
132.12
131.60
129.30
129.22
128.27
123.87
77.16 CDCl3
22.67
21.15
Ph
S
O
O
4s
13C NMR (151 MHz, CDCl3)
f1 (ppm)

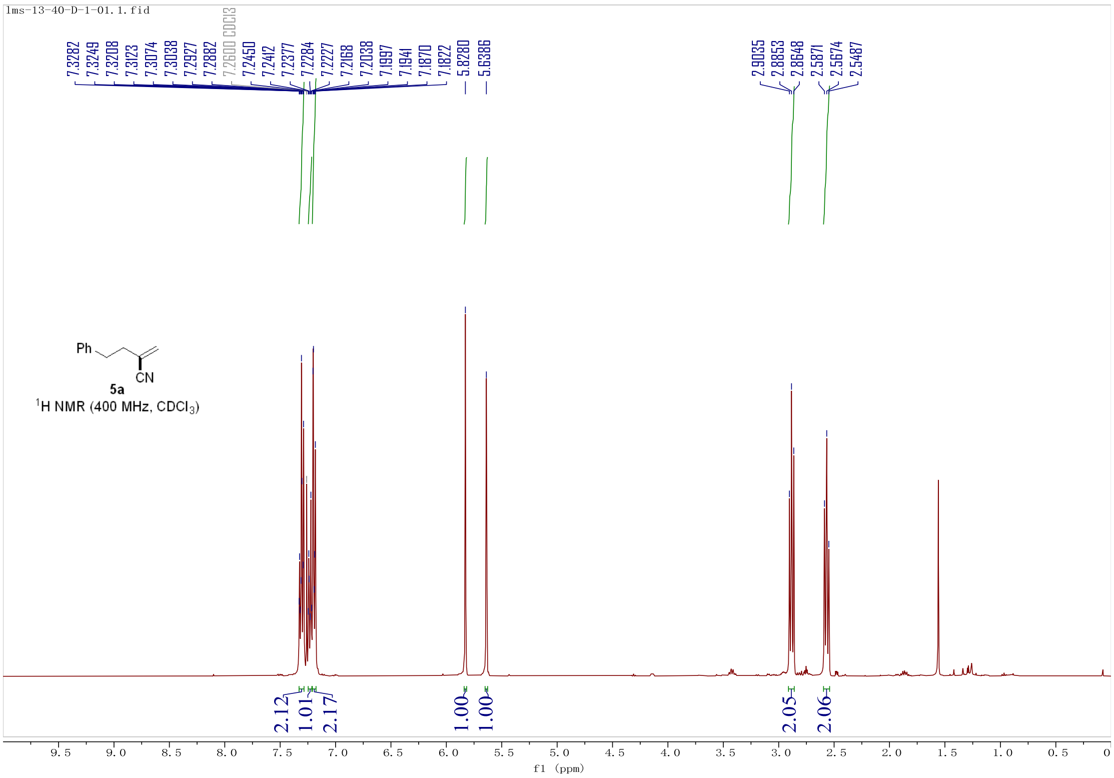
lms-13-40-D-1-01.1.fid
7.3282
7.3249
7.3208
7.3123
7.3074
7.3038
7.2927
7.2882
7.2600 CDCl3
7.2450
7.2412
7.2377
7.2284
7.2227
7.2168
7.2038
7.1997
7.1941
7.1870
7.1822
5.8280
5.6386
2.9035
2.8853
2.8648
2.5871
2.5674
2.5487
Ph
CN
5a
1H NMR (400 MHz, CDCl3)
2.12
1.01
2.17
1.00
1.00
2.05
2.06
f1 (ppm)

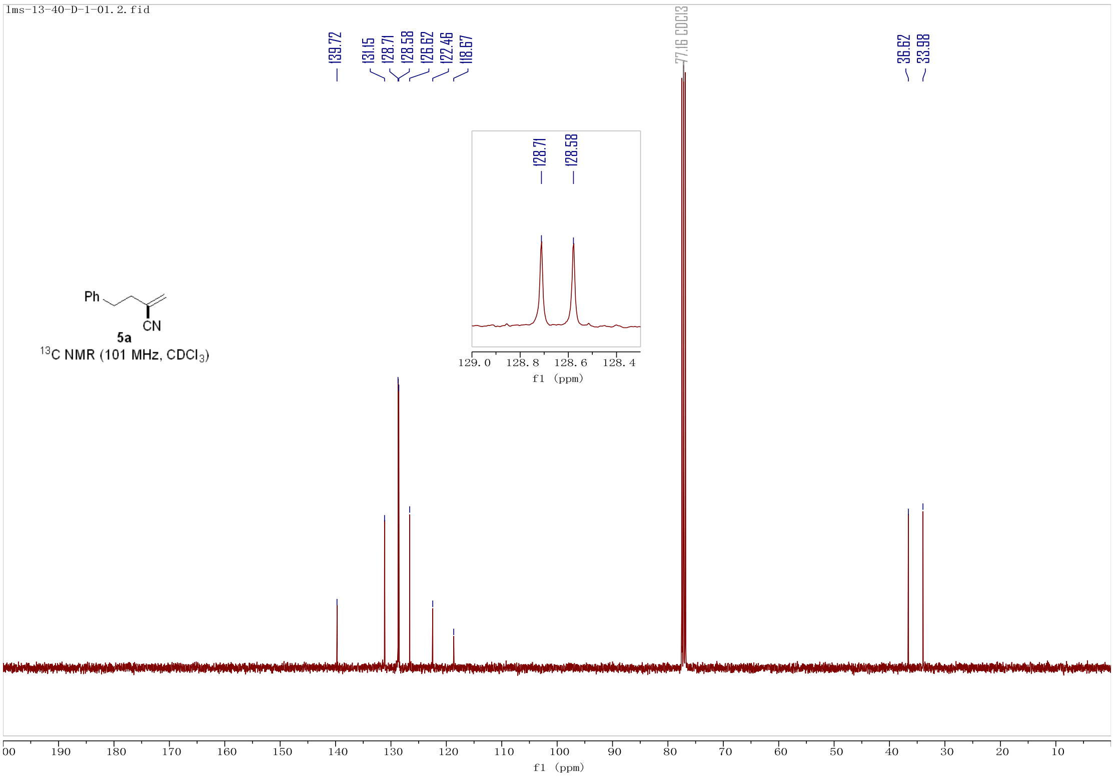
lms-13-40-D-1-01.2.fid
139.72
131.15
128.71
128.58
126.62
122.46
118.67
77.16 CDCl3
36.62
33.98
Ph
CN
5a
^{13}C NMR (101 MHz, CDCl$_3$)
129.0 128.8 128.6 128.4
f1 (ppm)

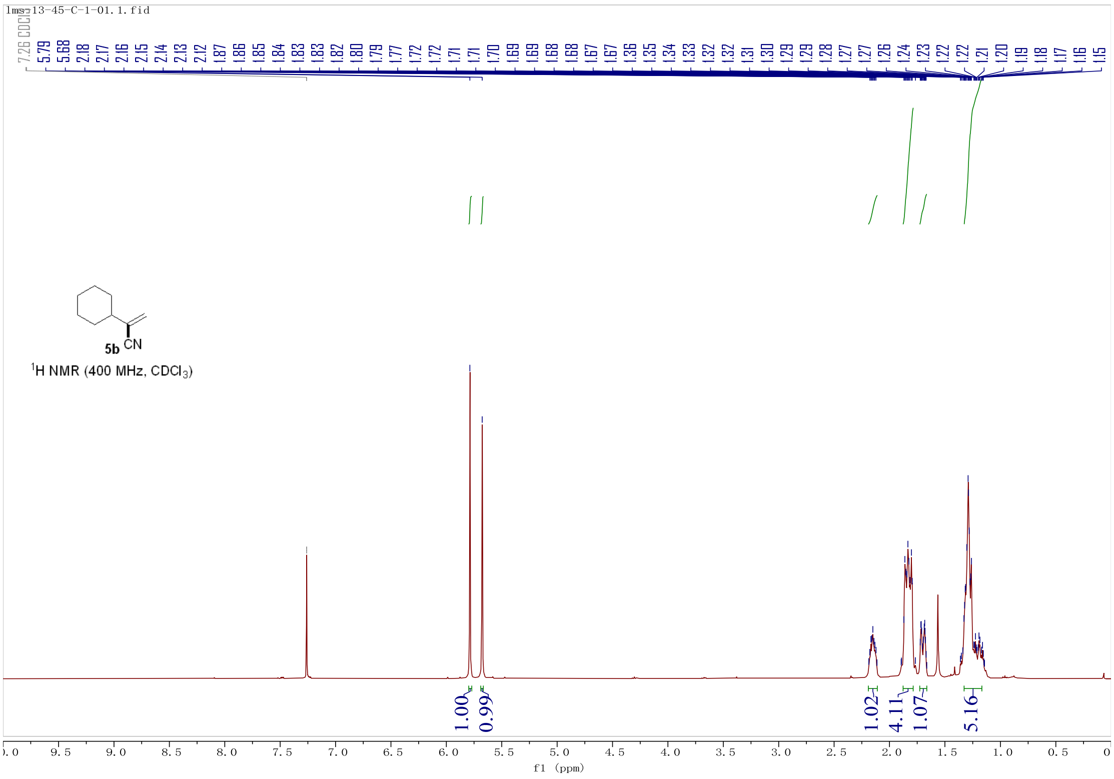
lms-13-45-C-1-01.1.fid
7.26 CDCl3
5.79
5.68
CN
5b
^{1}H NMR (400 MHz, CDCl$_3$)
1.00
0.99
1.02
4.11
1.07
5.16
f1 (ppm)

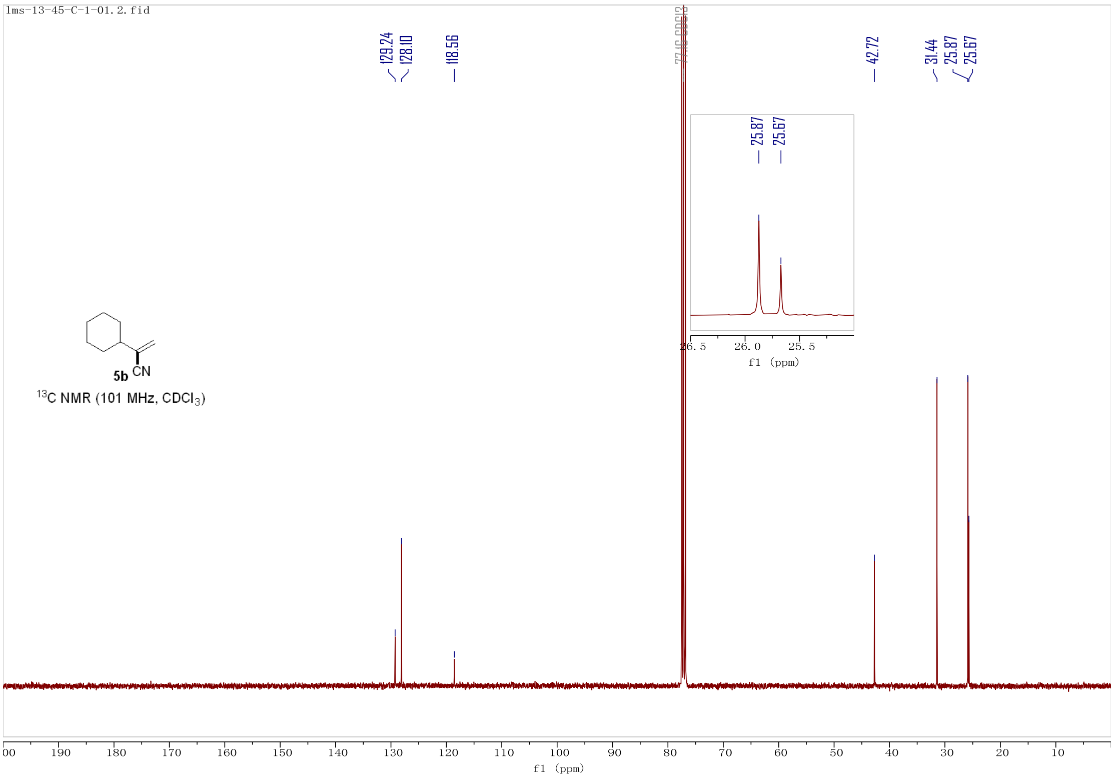
lms-13-45-C-1-01.2.fid
129.24
128.10
118.56
42.72
31.44
25.87
25.67
5b
CN
^{13}C NMR (101 MHz, CDCl$_3$)
f1 (ppm)

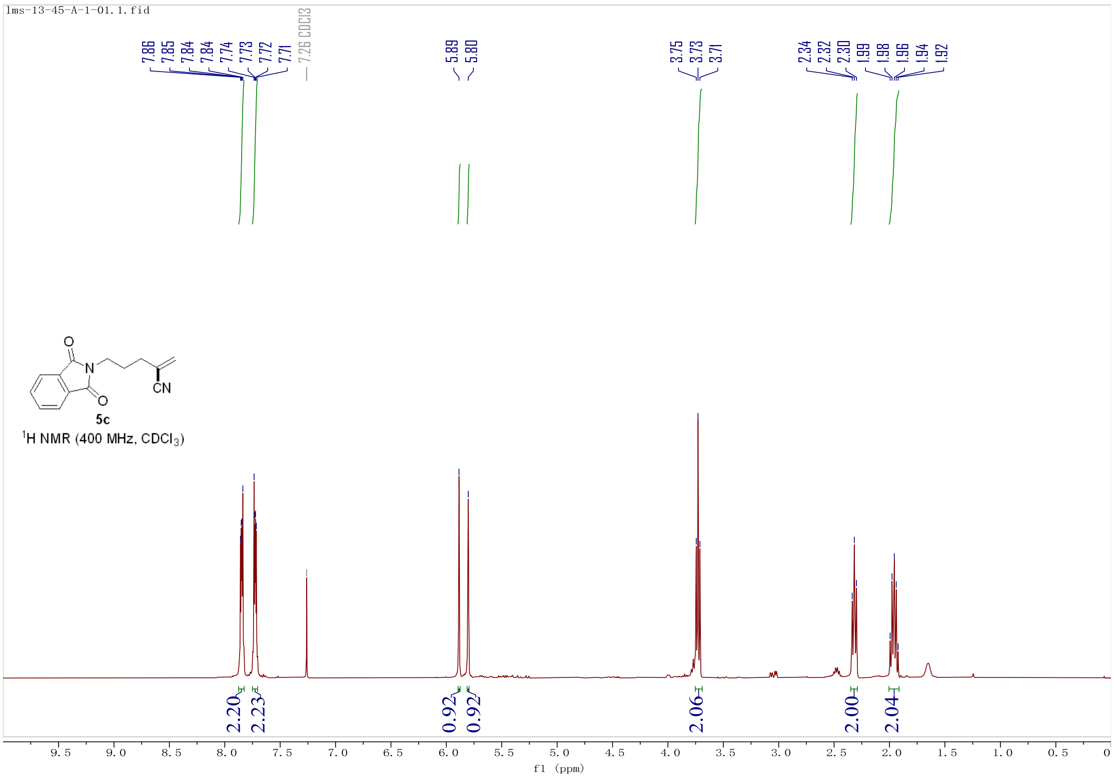
lms-13-45-A-1-01.1.fid
7.86
7.85
7.84
7.84
7.74
7.73
7.72
7.71
7.26 CDCl3
5.89
5.80
3.75
3.73
3.71
2.34
2.32
2.30
1.99
1.98
1.96
1.94
1.92
5c
CN
^{1}H NMR (400 MHz, CDCl$_3$)
2.20
2.23
0.92
0.92
2.06
2.00
2.04
f1 (ppm)

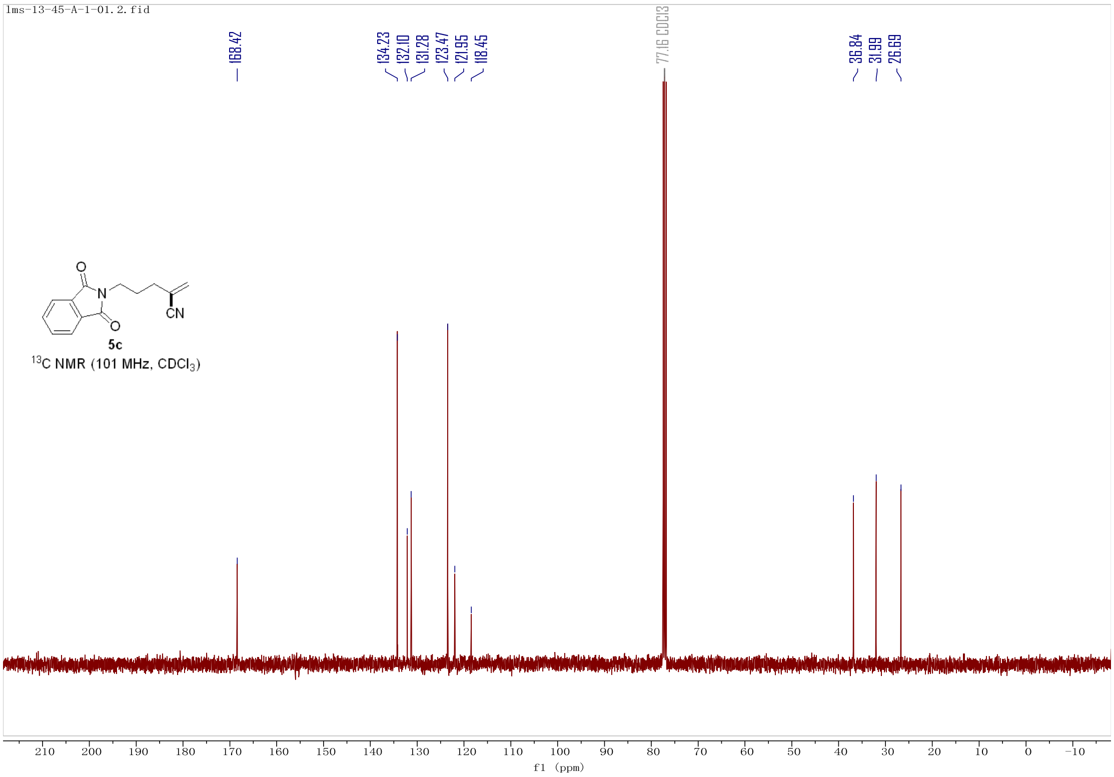
lms-13-45-A-1-01.2.fid
168.42
134.23
132.10
131.28
123.47
121.95
118.45
77.16 CDCl3
36.84
31.99
26.69
5c
^{13}C NMR (101 MHz, CDCl$_3$)
f1 (ppm)

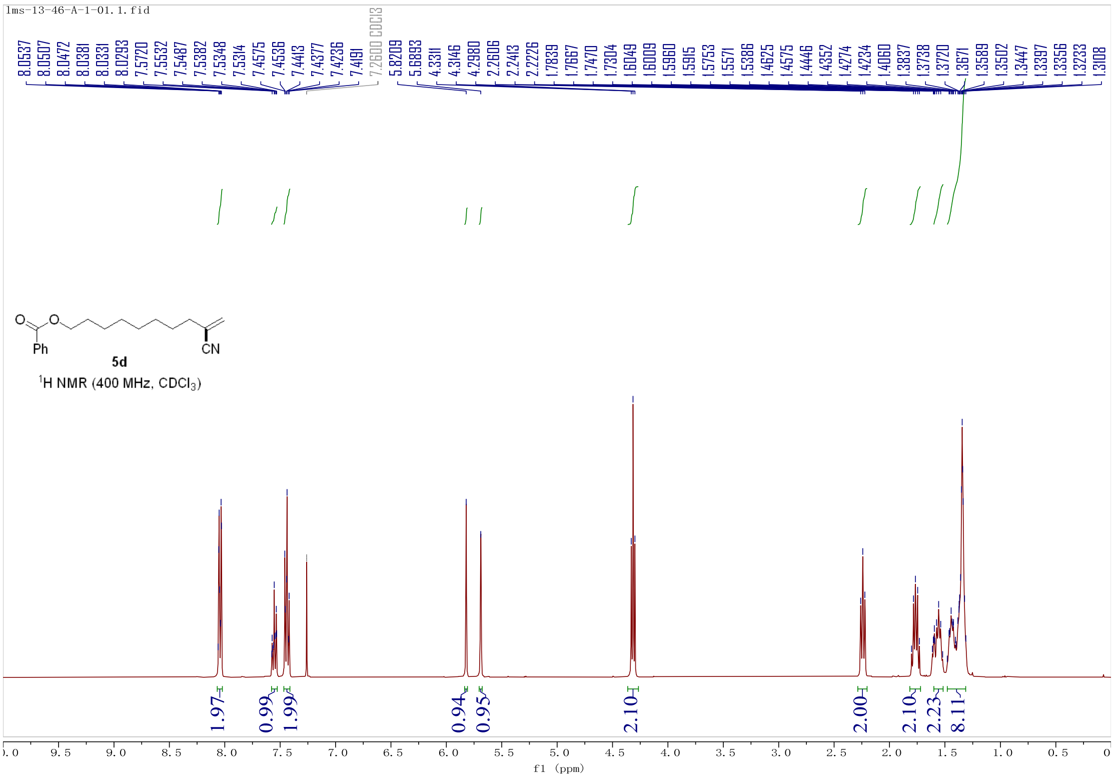
lms-13-46-A-1-01.1.fid
8.0537
8.0507
8.0472
8.0381
8.0331
8.0293
7.5720
7.5532
7.5487
7.5382
7.5348
7.5314
7.4575
7.4536
7.4413
7.4377
7.4236
7.4191
7.2600 CDCl3
5.8209
5.6893
4.3311
4.3146
4.2980
2.2606
2.2413
2.2226
1.7839
1.7667
1.7470
1.7304
1.6049
1.6009
1.5960
1.5915
1.5753
1.5571
1.5386
1.4625
1.4575
1.4446
1.4352
1.4274
1.4234
1.4060
1.3837
1.3738
1.3720
1.3671
1.3589
1.3502
1.3447
1.3397
1.3356
1.3233
1.3108
5d
^{1}H NMR (400 MHz, CDCl$_3$)
1.97
0.99
1.99
0.94
0.95
2.10
2.00
2.10
2.23
8.11
f1 (ppm)

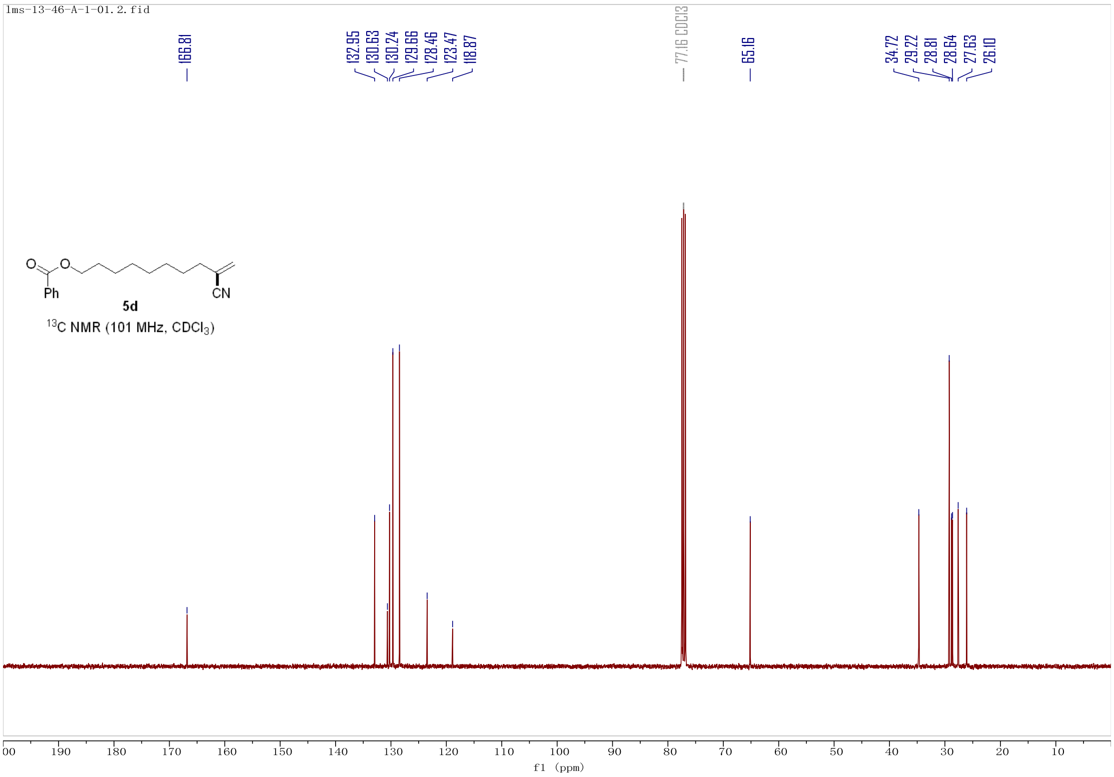
lms-13-46-A-1-01.2.fid
166.81
132.95
130.63
130.24
129.66
128.46
123.47
118.87
77.16 CDCl3
65.16
34.72
29.22
28.81
28.64
27.63
26.10
5d
13C NMR (101 MHz, CDCl3)
f1 (ppm)

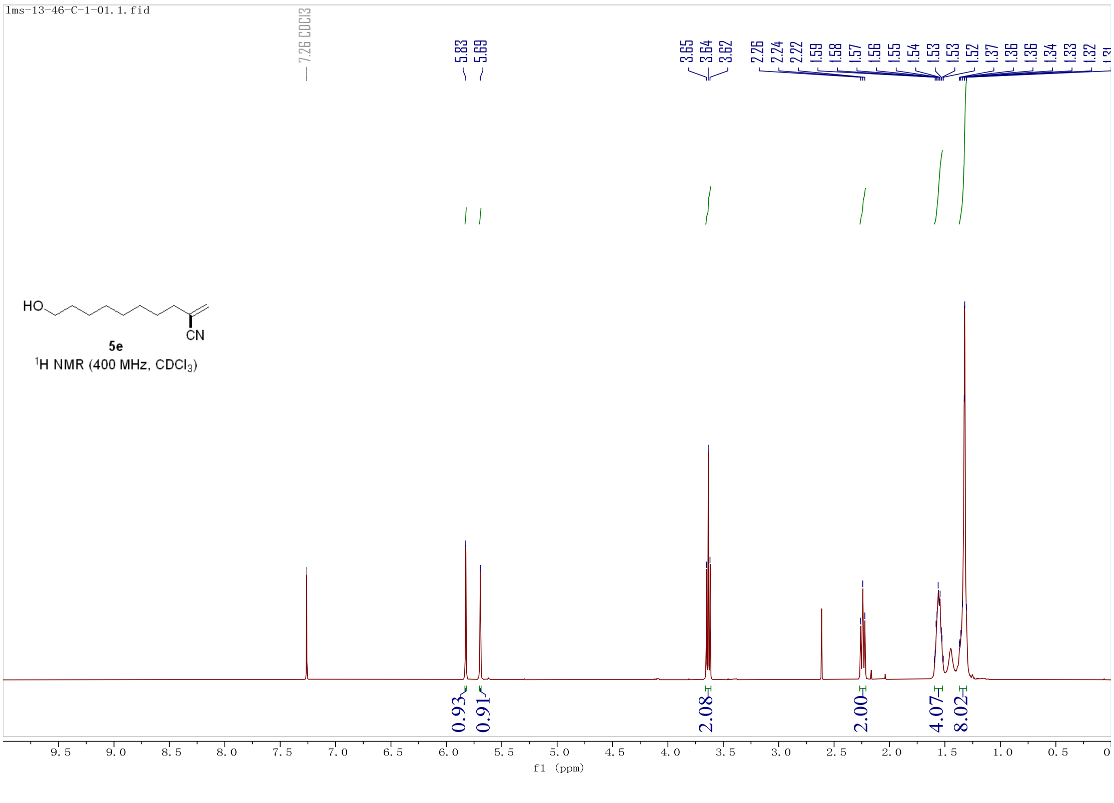
lms-13-46-C-1-01.1.fid
7.26 CDCl3
5.83
5.69
3.65
3.64
3.62
2.26
2.24
2.22
1.59
1.58
1.57
1.56
1.55
1.54
1.53
1.53
1.52
1.37
1.36
1.36
1.34
1.33
1.32
HO
CN
5e
1H NMR (400 MHz, CDCl3)
0.93
0.91
2.08
2.00
4.07
8.02
f1 (ppm)

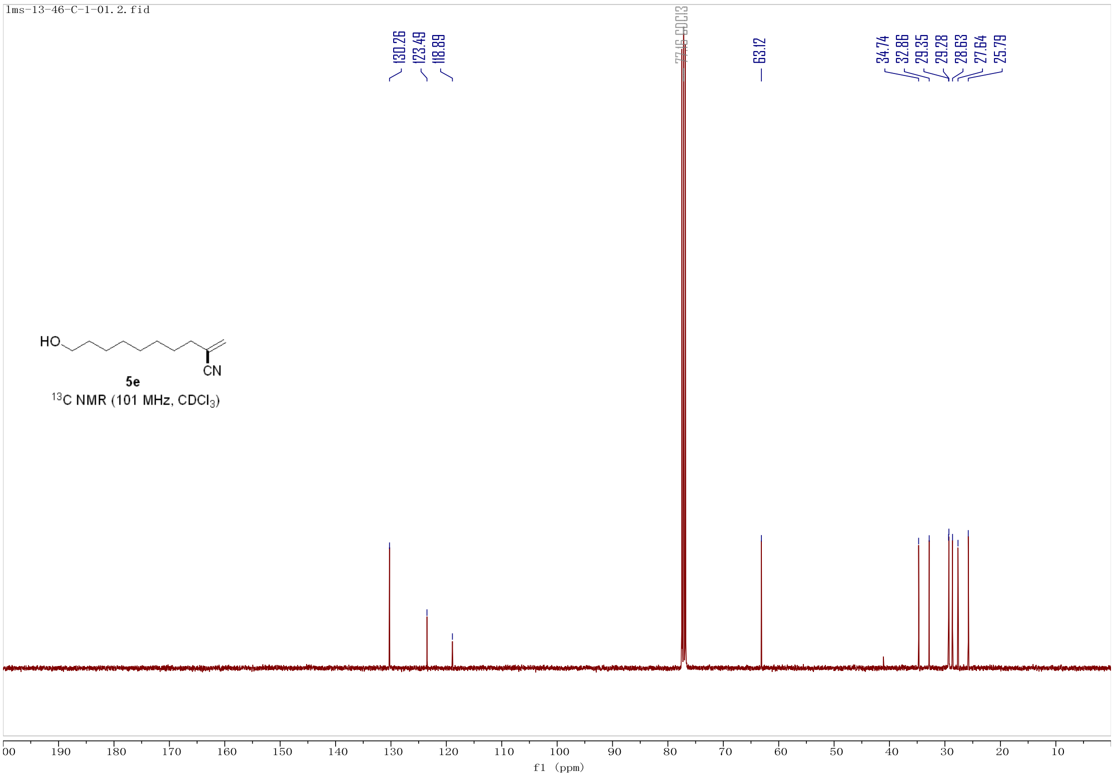
lms-13-46-C-1-01.2.fid
130.26
123.49
118.89
77.16 CDCl3
63.12
34.74
32.86
29.35
29.28
28.63
27.64
25.79
HO
CN
5e
13C NMR (101 MHz, CDCl3)
f1 (ppm)

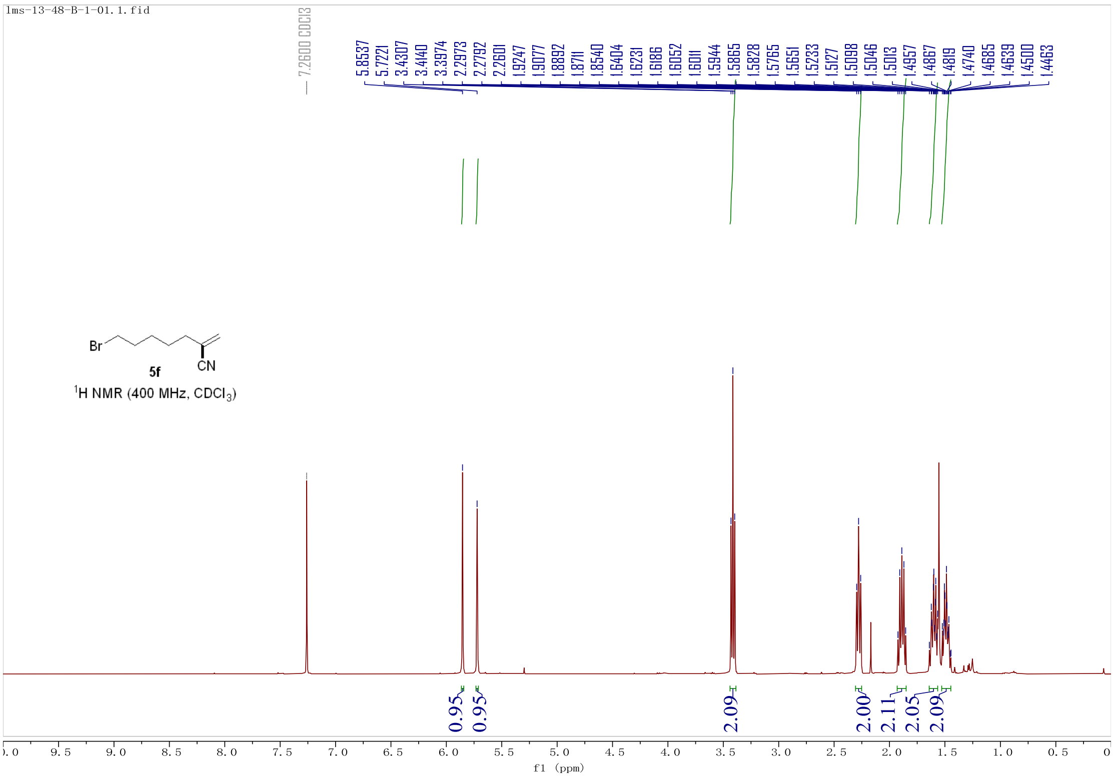
lms-13-48-B-1-01.1.fid
7.2600 CDCl3
5.8537
5.7221
3.4307
3.4140
3.3974
2.2973
2.2792
2.2601
1.9247
1.9077
1.8892
1.8711
1.8540
1.6404
1.6231
1.6186
1.6052
1.6011
1.5944
1.5865
1.5828
1.5765
1.5651
1.5233
1.5127
1.5098
1.5046
1.5013
1.4957
1.4867
1.4819
1.4740
1.4685
1.4639
1.4500
1.4463
Br
CN
5f
1H NMR (400 MHz, CDCl3)
0.95
0.95
2.09
2.00
2.11
2.05
2.09
f1 (ppm)

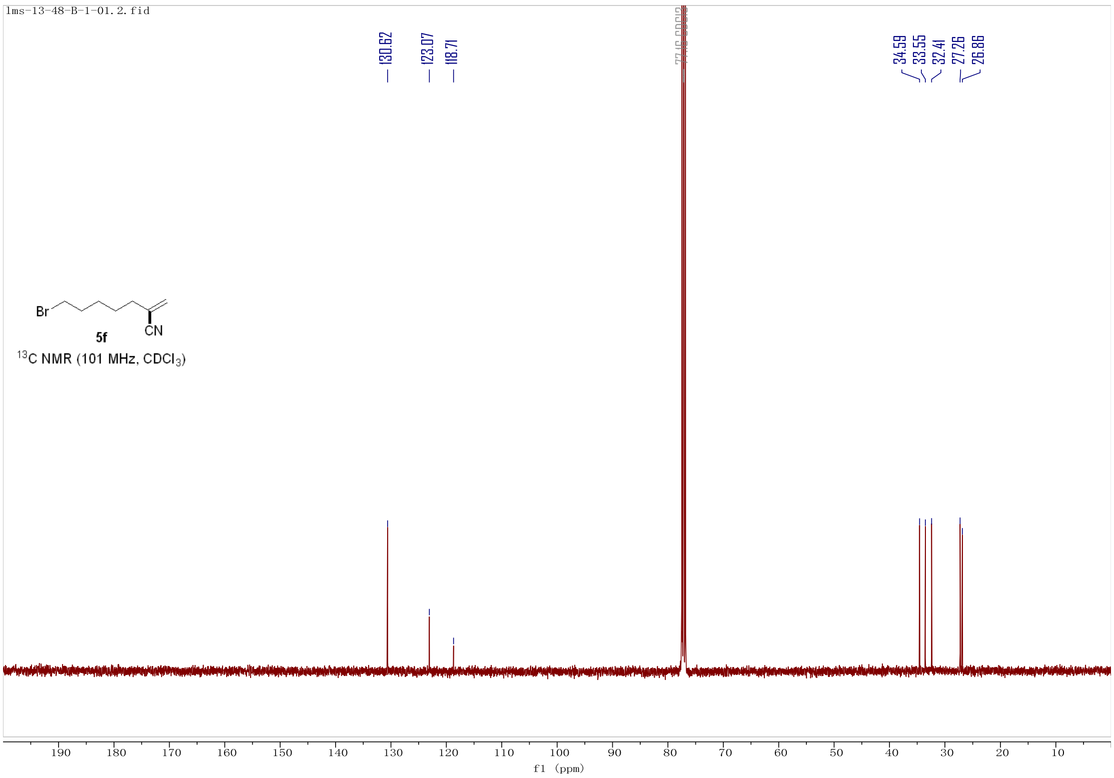
lms-13-48-B-1-01.2.fid
130.62
123.07
118.71
34.59
33.55
32.41
27.26
26.86
Br
CN
5f
^{13}C NMR (101 MHz, CDCl$_3$)
f1 (ppm)

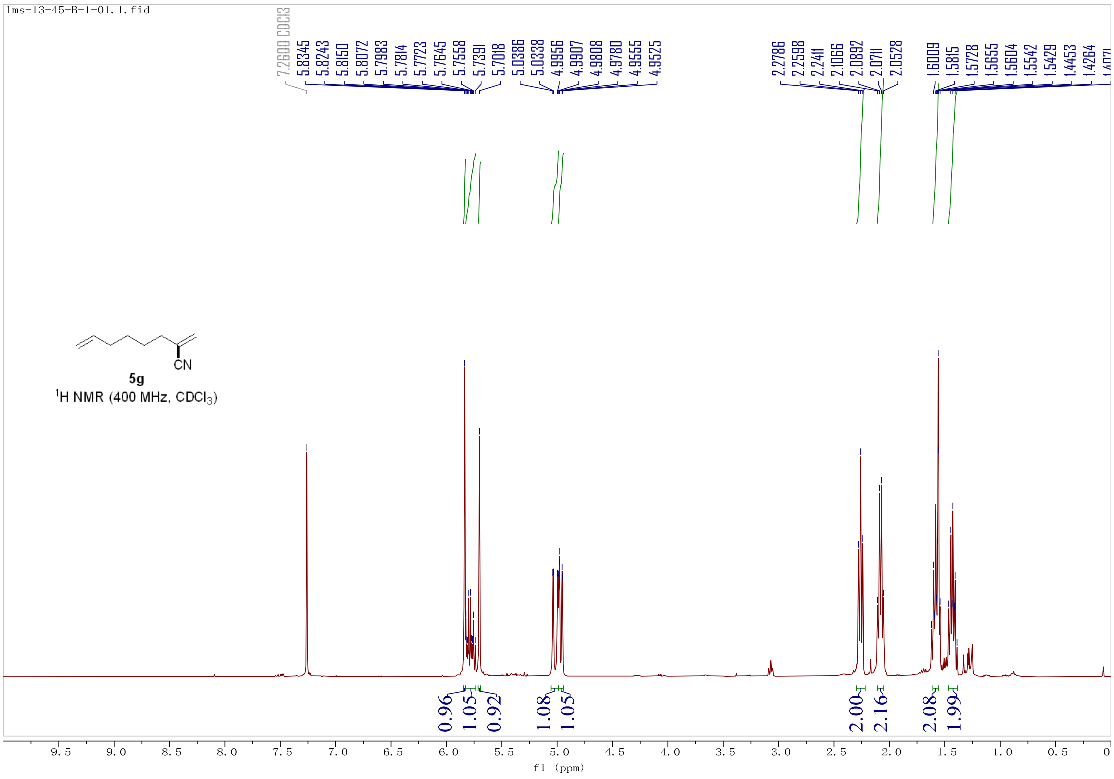
lms-13-45-B-1-01.1.fid
7.2600 CDCl3
5.8345
5.8243
5.8150
5.8072
5.7983
5.7814
5.7723
5.7645
5.7558
5.7391
5.7018
5.0386
5.0338
4.9956
4.9907
4.9808
4.9780
4.9555
4.9525
2.2786
2.2598
2.2411
2.1066
2.0892
2.0711
2.0528
1.6009
1.5815
1.5728
1.5655
1.5604
1.5542
1.5429
1.4453
1.4264
CN
5g
^{1}H NMR (400 MHz, CDCl$_3$)
0.96
1.05
0.92
1.08
1.05
2.00
2.16
2.08
1.99
f1 (ppm)

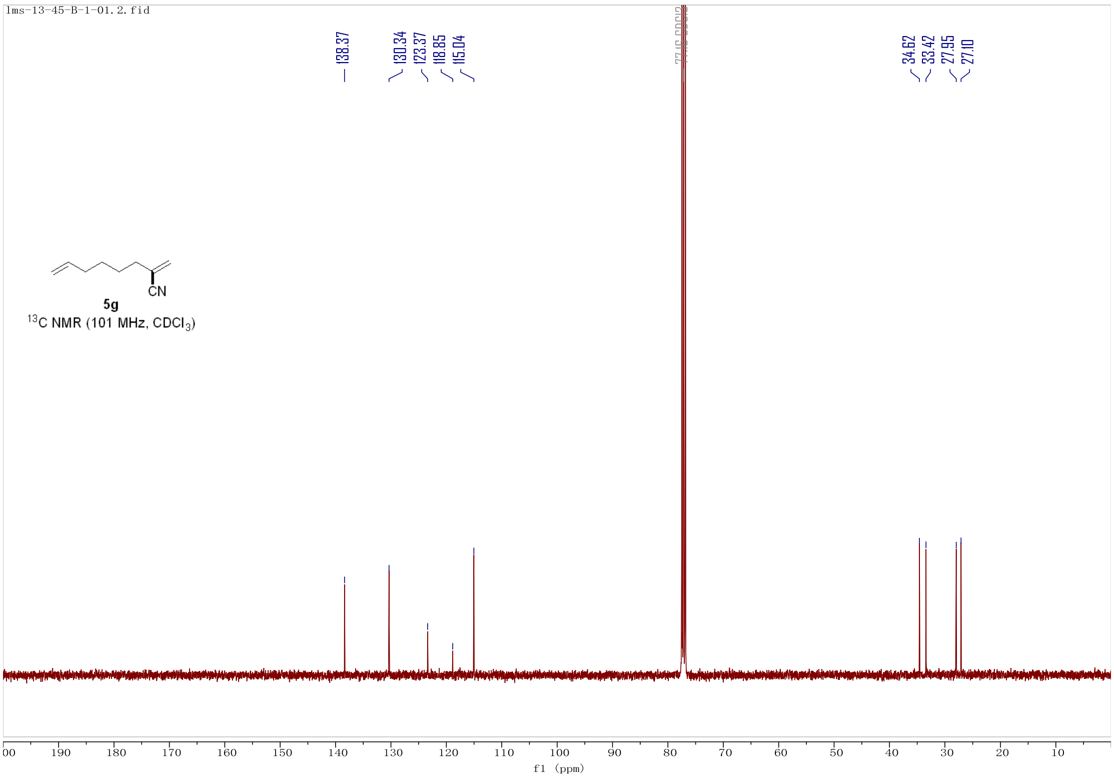
lms-13-45-B-1-01.2.fid
138.37
130.34
123.37
118.85
115.04
34.62
33.42
27.95
27.10
5g
^{13}C NMR (101 MHz, CDCl$_3$)
f1 (ppm)

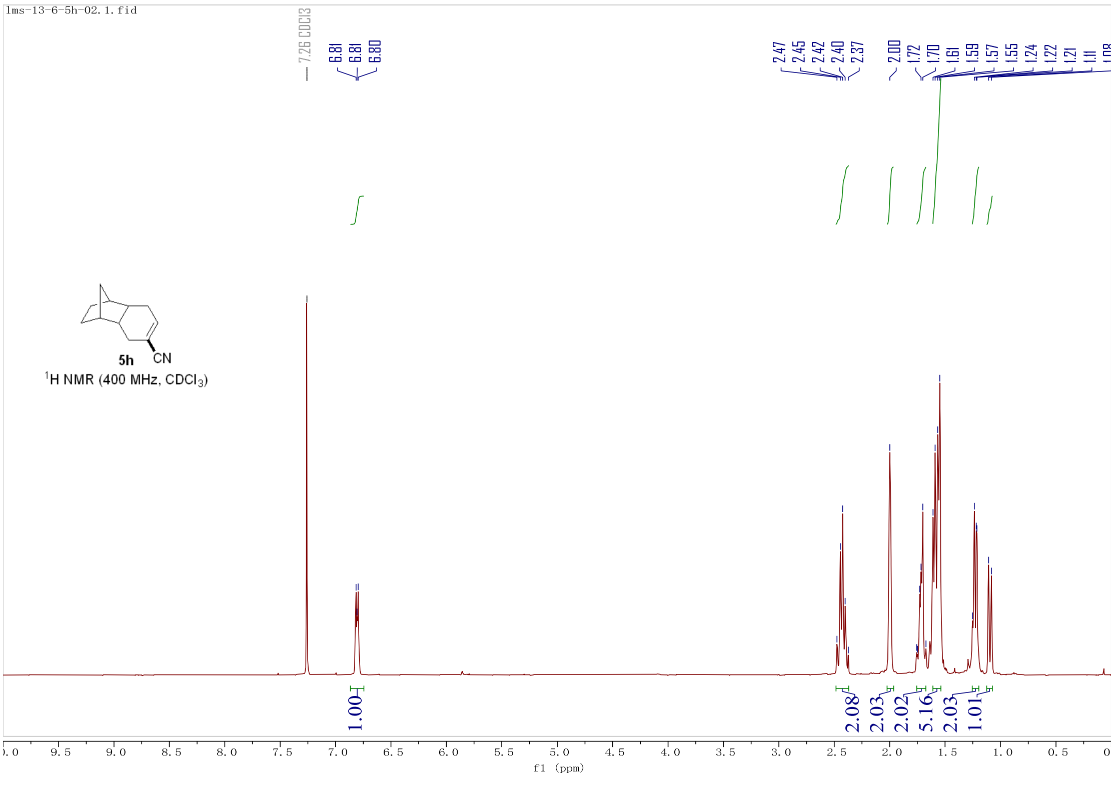
lms-13-6-5h-02.1.fid
7.26 CDCl3
6.81
6.81
6.80
2.47
2.45
2.42
2.40
2.37
2.00
1.72
1.70
1.61
1.59
1.57
1.55
1.24
1.22
1.21
1.11
5h
^{1}H NMR (400 MHz, CDCl$_3$)
1.00
2.08
2.03
2.02
5.16
2.03
1.01
f1 (ppm)

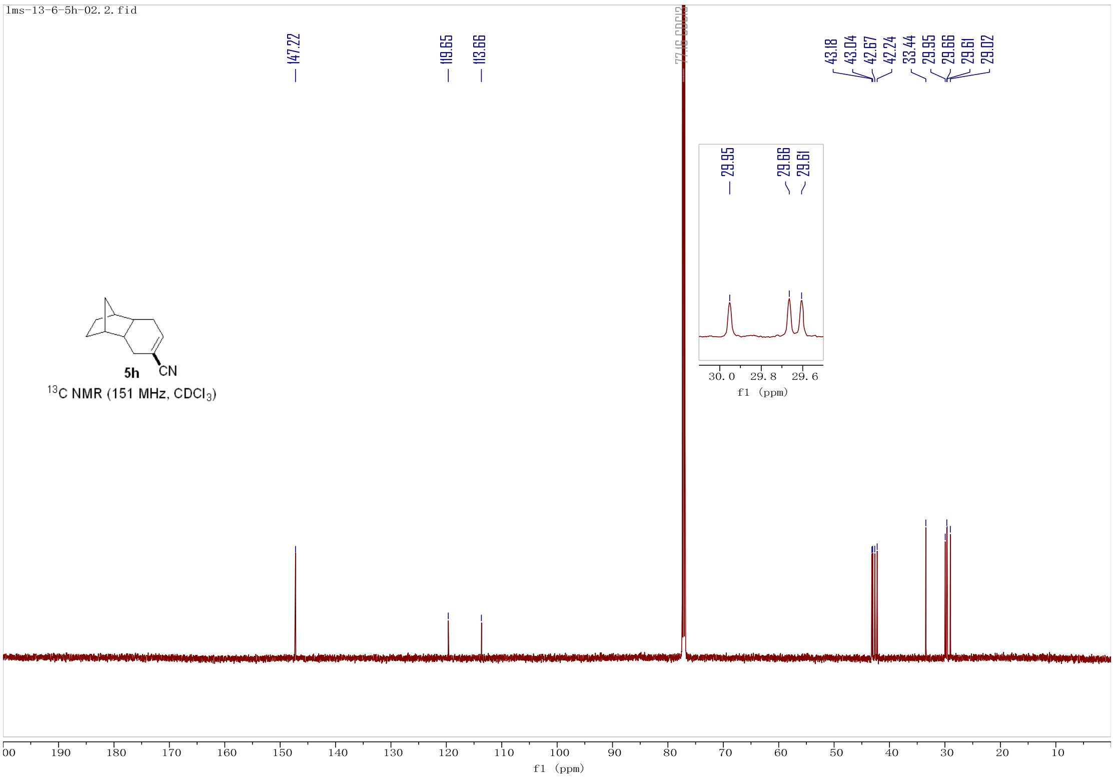
lms-13-6-5h-02.2.fid
147.22
119.65
113.66
43.18
43.04
42.67
42.24
33.44
29.95
29.66
29.61
29.02
5h
CN
^{13}C NMR (151 MHz, CDCl$_3$)
f1 (ppm)

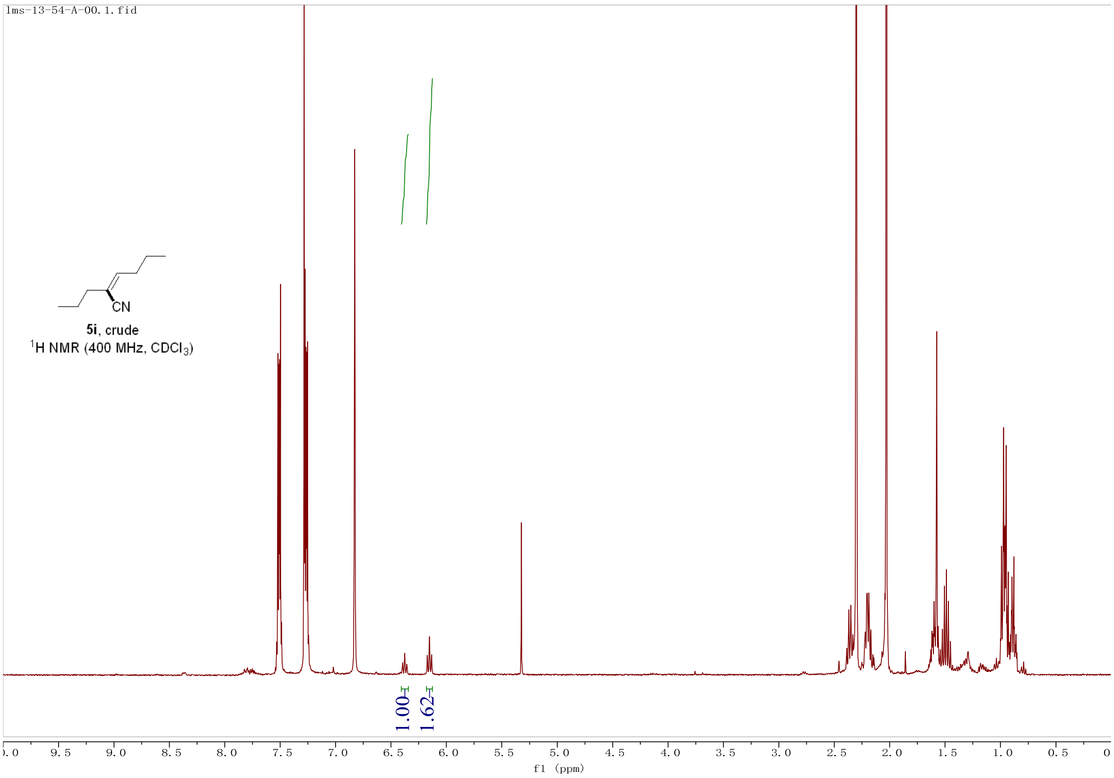
lms-13-54-A-00.1.fid
CN
5i, crude
^{1}H NMR (400 MHz, CDCl$_3$)
1.00
1.62
f1 (ppm)

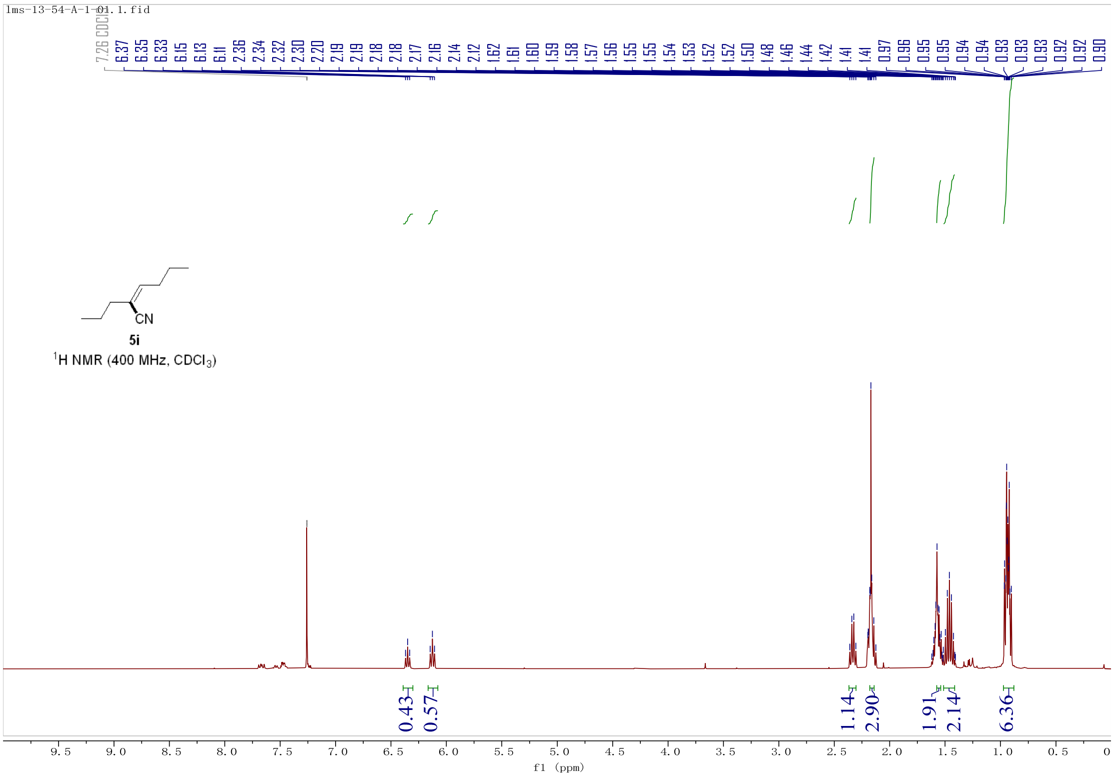
lms-13-54-A-1-01.1.fid
7.26 CDCl3
6.37 6.35 6.33 6.15 6.13 6.11 2.36 2.34 2.32 2.30 2.20 2.19 2.19 2.18 2.18 2.17 2.16 2.14 2.12 1.62 1.61 1.60 1.59 1.58 1.57 1.56 1.55 1.55 1.54 1.53 1.52 1.52 1.50 1.48 1.46 1.44 1.42 1.41 1.41 0.97 0.96 0.95 0.95 0.94 0.94 0.93 0.93 0.93 0.93 0.92 0.92 0.90
CN
5i
^{1}H NMR (400 MHz, $CDCl_3$)
0.43 0.57 1.14 2.90 1.91 2.14 6.36
f1 (ppm)

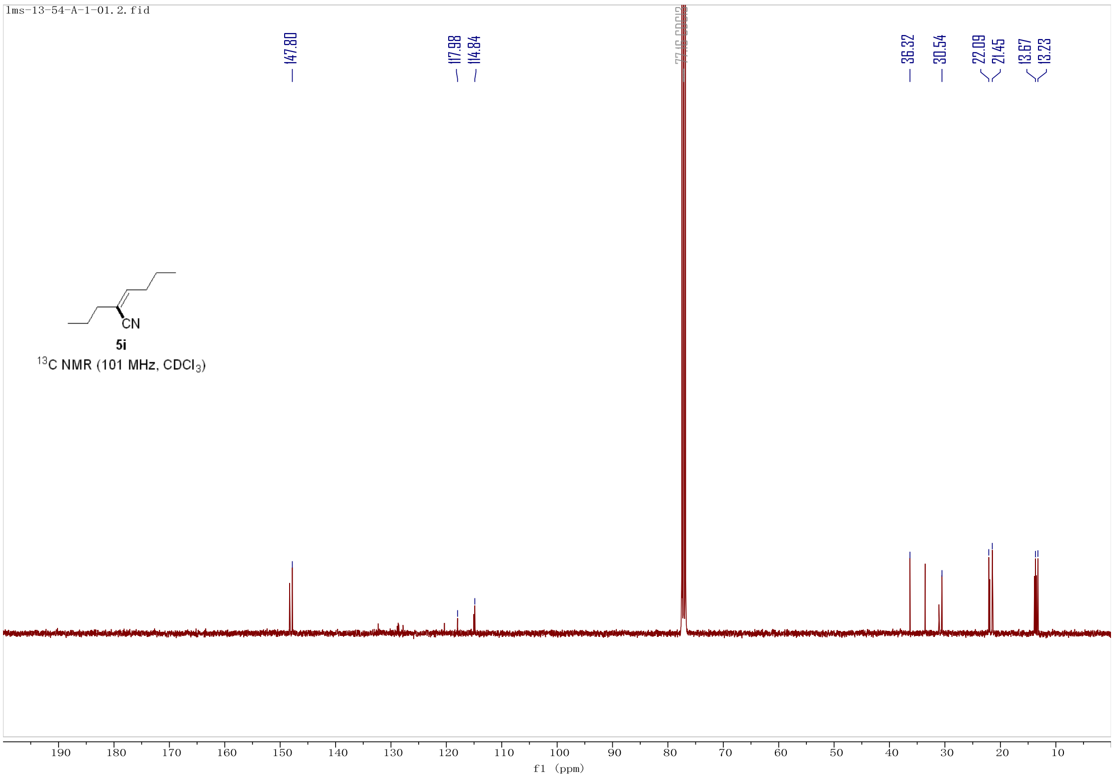
lms-13-54-A-1-01.2.fid
147.80
117.98 114.84
77.16 CDCl3
36.32 30.54 22.09 21.45 13.67 13.23
CN
5i
^{13}C NMR (101 MHz, $CDCl_3$)
f1 (ppm)

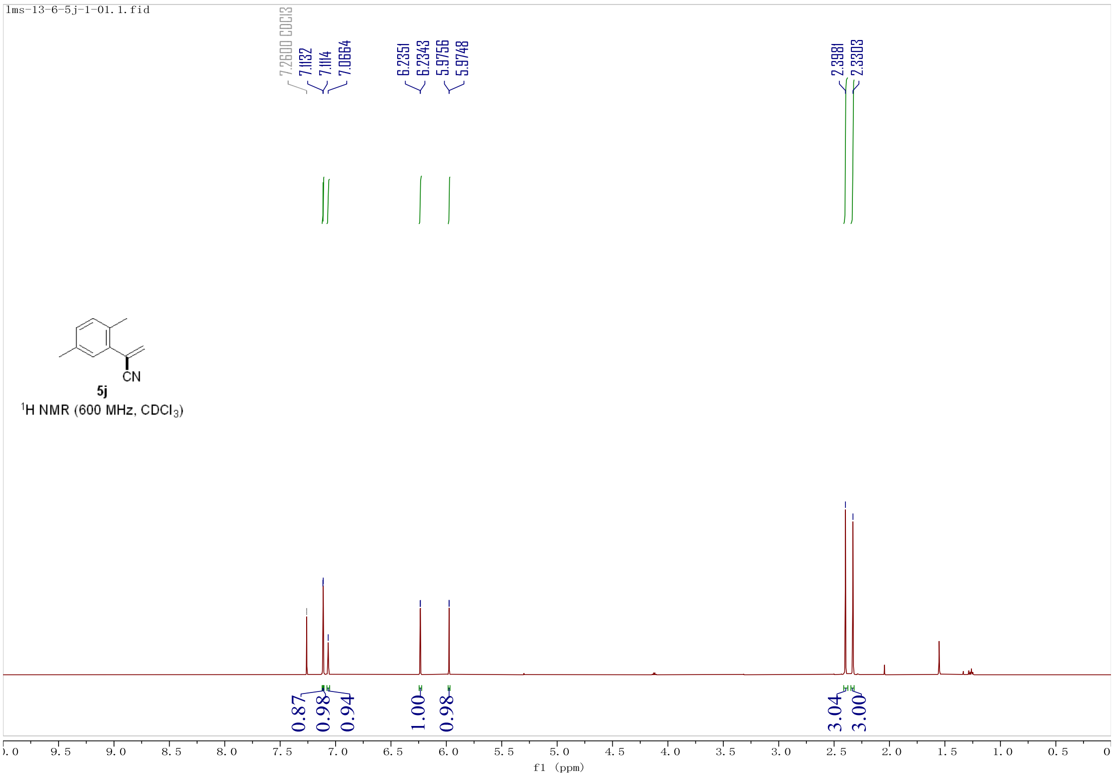

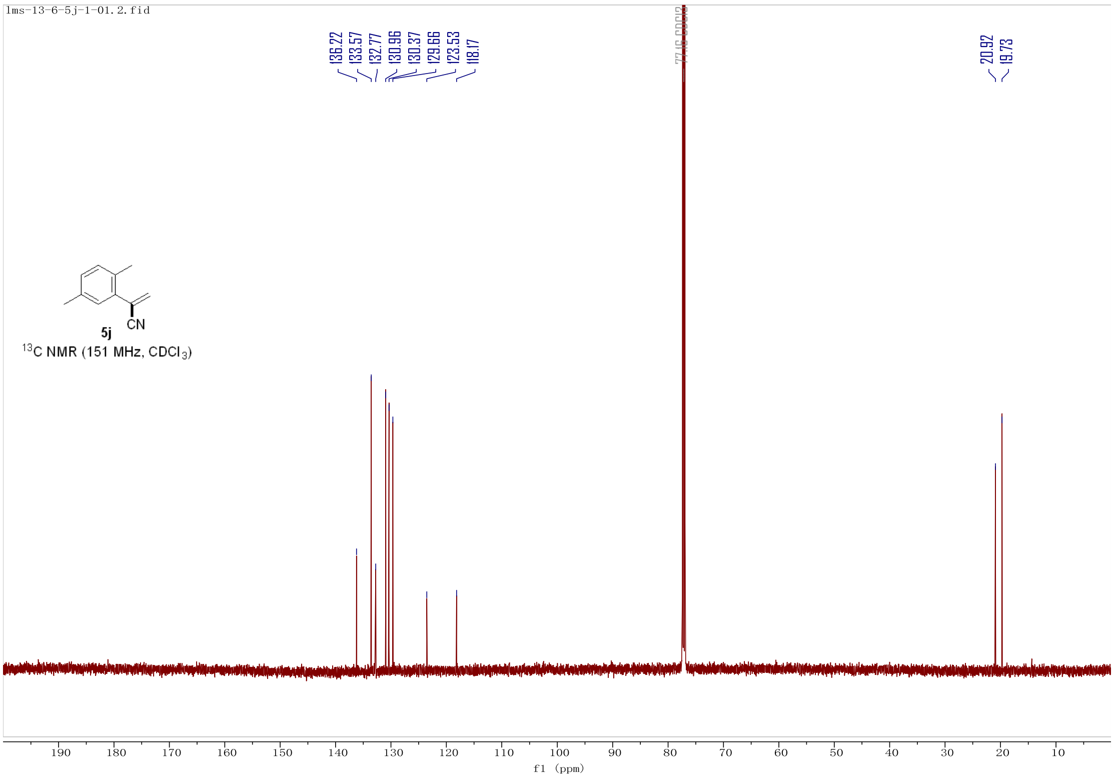

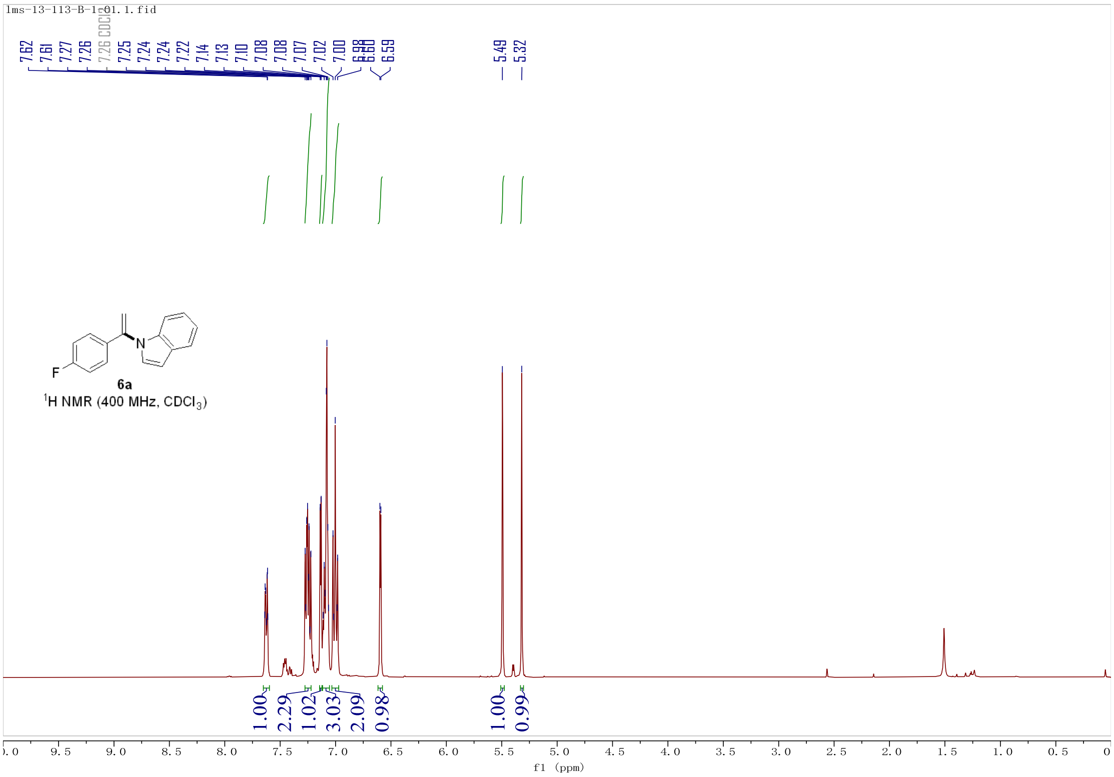

lms-13-113-B-1-01.1.fid
7.62 7.61 7.27 7.26 7.26 CDCl3 7.25 7.24 7.24 7.22 7.14 7.13 7.10 7.08 7.08 7.07 7.02 7.00 6.98 6.60 6.59
5.49 5.32
6a
1H NMR (400 MHz, CDCl3)
1.00 2.29 1.02 3.03 2.09 0.98 1.00 0.99
f1 (ppm)

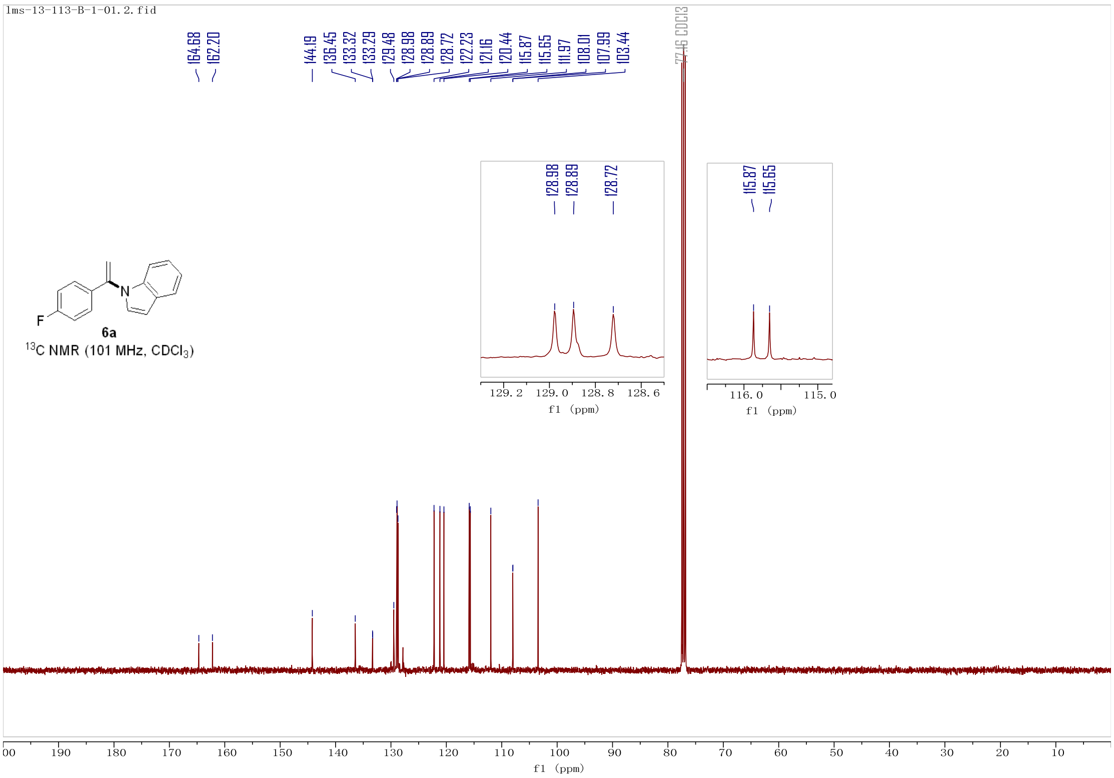

lms-13-113-B-1-01.2.fid
164.68 162.20 144.19 136.45 133.32 133.29 129.48 128.98 128.89 128.72 122.23 121.16 120.44 115.87 115.65 111.97 108.01 107.99 103.44
77.16 CDCl3
128.98 128.89 128.72
115.87 115.65
6a
13C NMR (101 MHz, CDCl3)
f1 (ppm)

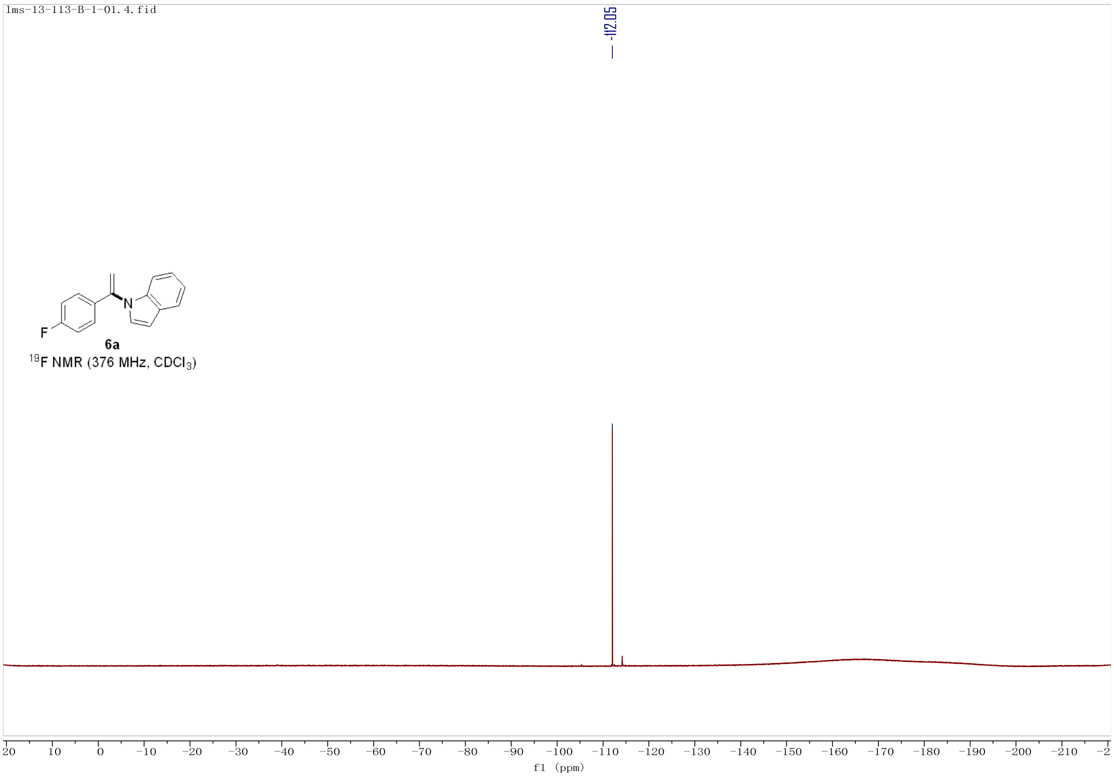
lms-13-113-B-1-01.4.fid
-112.05
6a
19F NMR (376 MHz, CDCl3)
f1 (ppm)

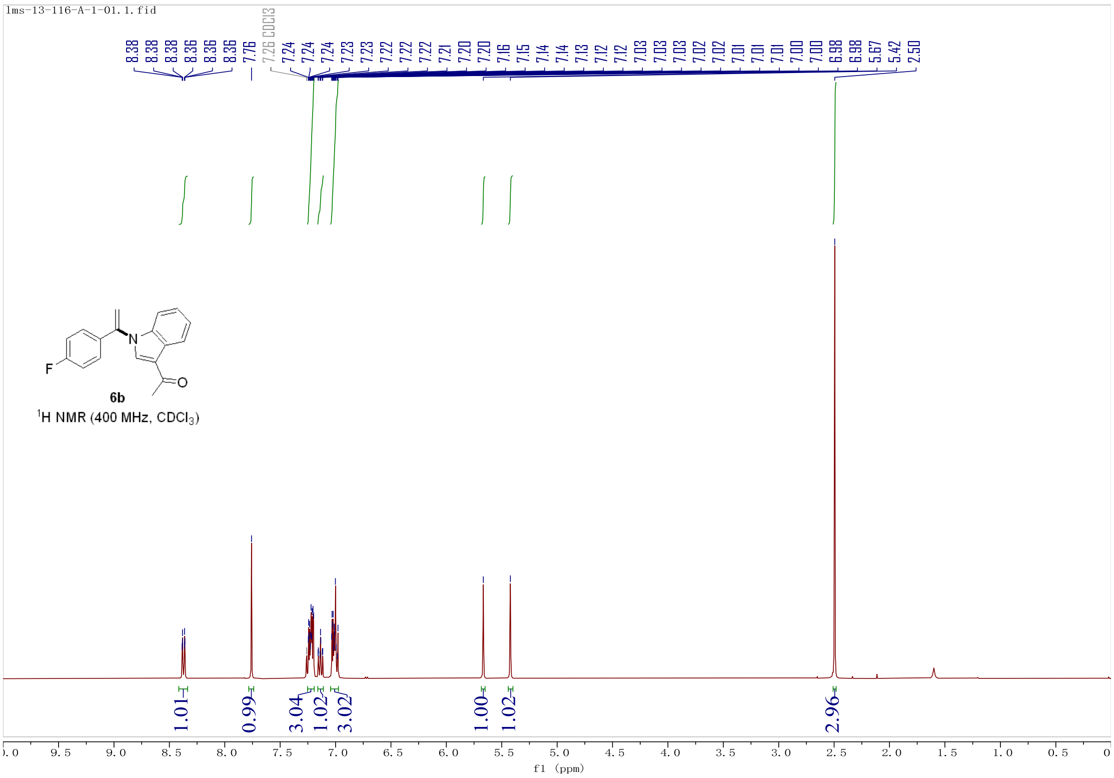
lms-13-116-A-1-01.1.fid
8.38 8.38 8.38 8.36 8.36 8.36 7.76 7.26 CDCl3 7.24 7.24 7.24 7.23 7.23 7.22 7.22 7.22 7.21 7.20 7.20 7.16 7.15 7.14 7.14 7.13 7.12 7.12 7.12 7.03 7.03 7.02 7.02 7.01 7.01 7.01 7.00 7.00 6.98 6.98 5.67 5.42 2.50
6b
1H NMR (400 MHz, CDCl3)
1.01 0.99 3.04 1.02 3.02 1.00 1.02 2.96
f1 (ppm)

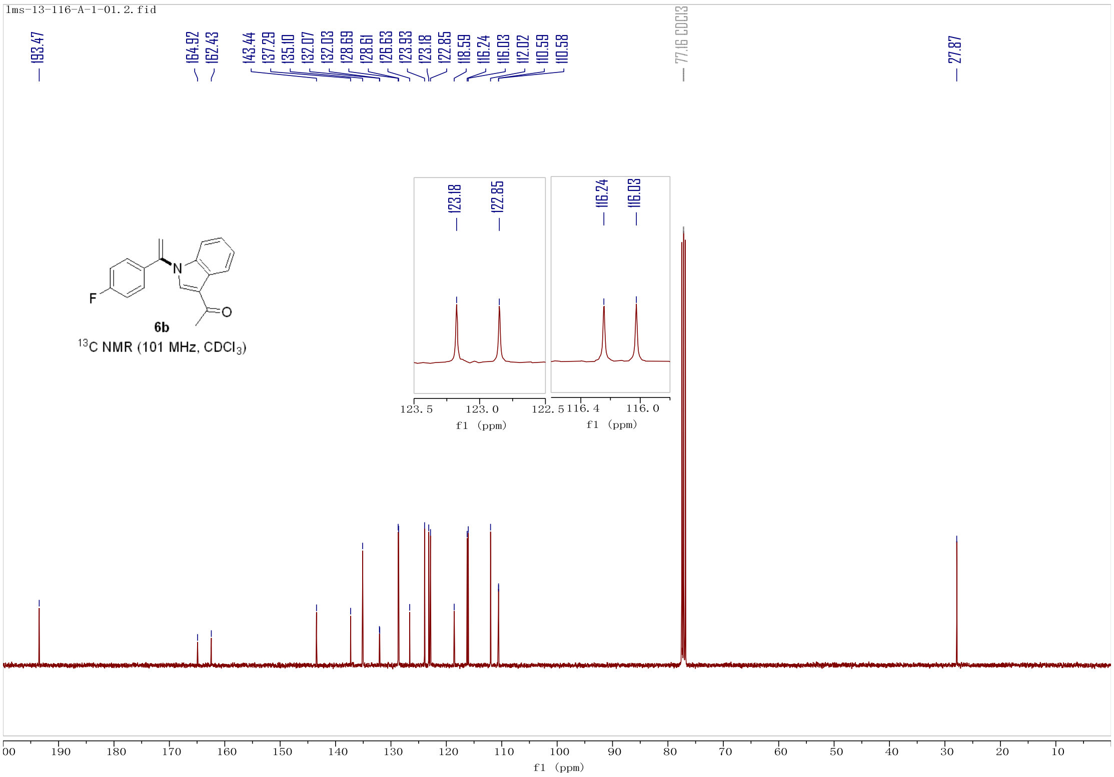
lms-13-116-A-1-01.2.fid
193.47
164.92
162.43
143.44
137.29
135.10
132.07
132.03
128.69
128.61
126.63
123.93
123.18
122.85
118.59
116.24
116.03
112.02
110.59
110.58
77.16 CDCl3
27.87
6b
13C NMR (101 MHz, CDCl3)
f1 (ppm)

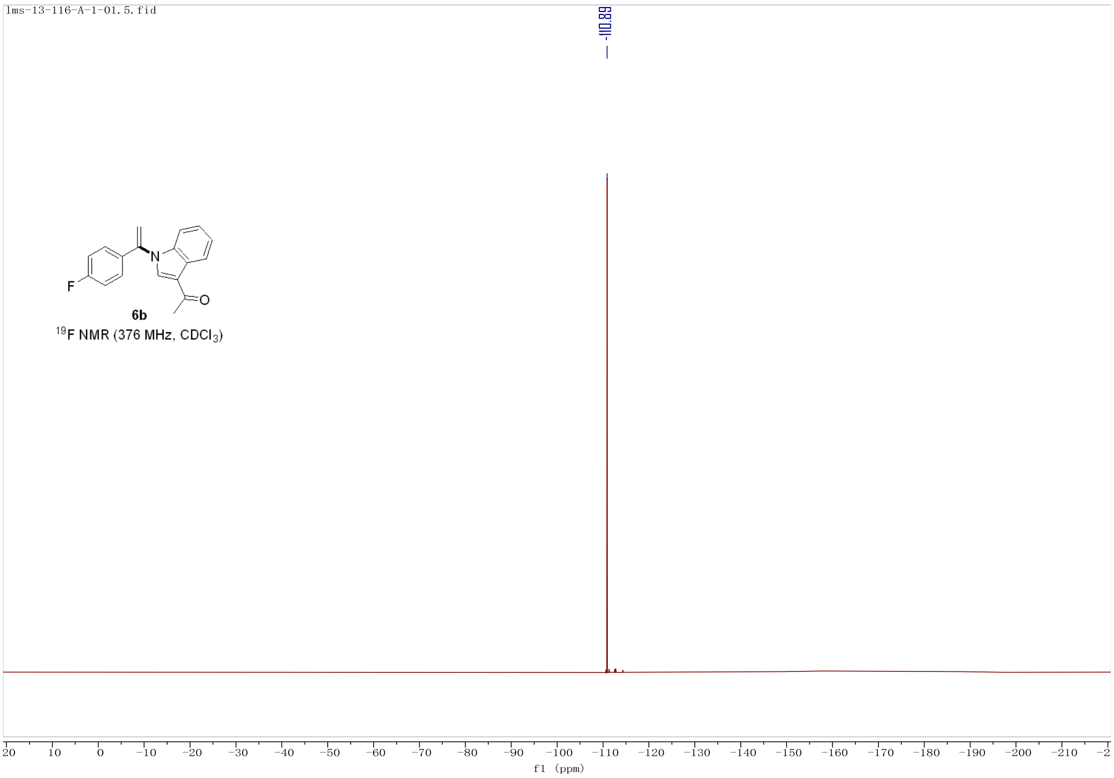
lms-13-116-A-1-01.5.fid
-110.89
6b
19F NMR (376 MHz, CDCl3)
f1 (ppm)

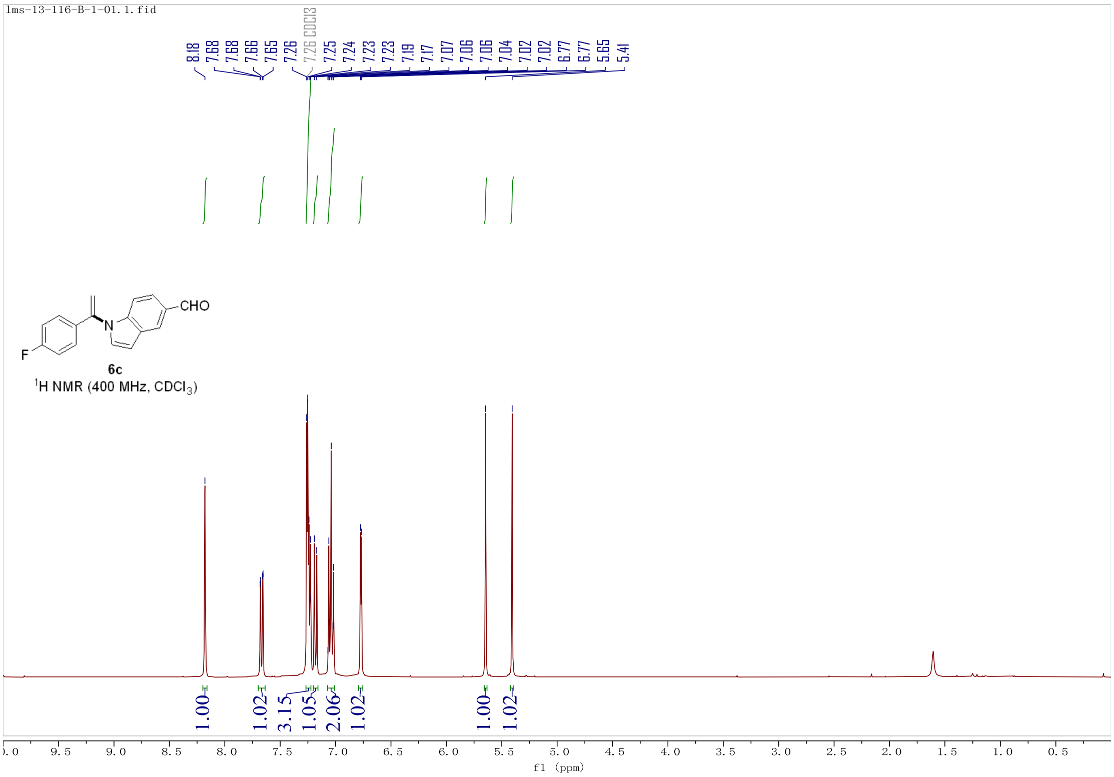
lms-13-116-B-1-01.1.fid
8.18
7.68
7.68
7.66
7.65
7.26
7.26 CDCl3
7.25
7.24
7.23
7.23
7.19
7.17
7.07
7.06
7.06
7.04
7.02
7.02
6.77
6.77
5.65
5.41
CHO
N
F
6c
1H NMR (400 MHz, CDCl3)
1.00
1.02
3.15
1.05
2.06
1.02
1.00
1.02
f1 (ppm)

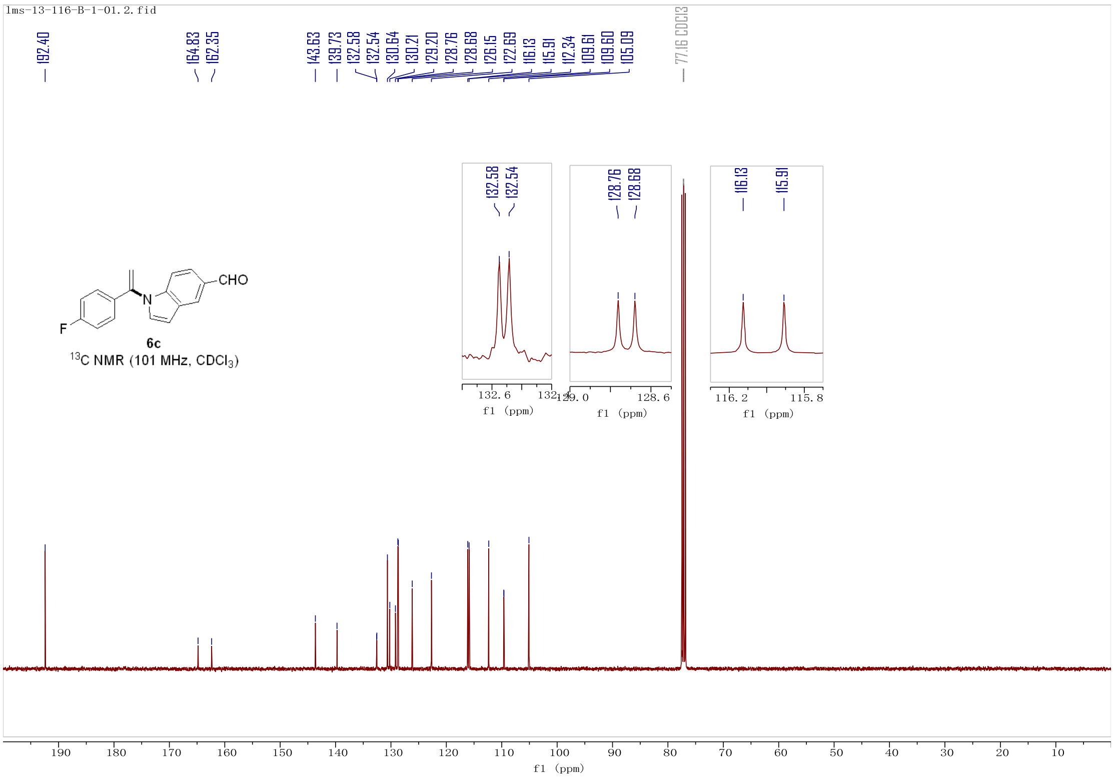
lms-13-116-B-1-01.2.fid
192.40
164.83
162.35
143.63
139.73
132.58
132.54
130.64
130.21
129.20
128.76
128.68
126.15
122.69
116.13
115.91
112.34
109.61
109.60
105.09
77.16 CDCl3
CHO
N
F
6c
13C NMR (101 MHz, CDCl3)
f1 (ppm)

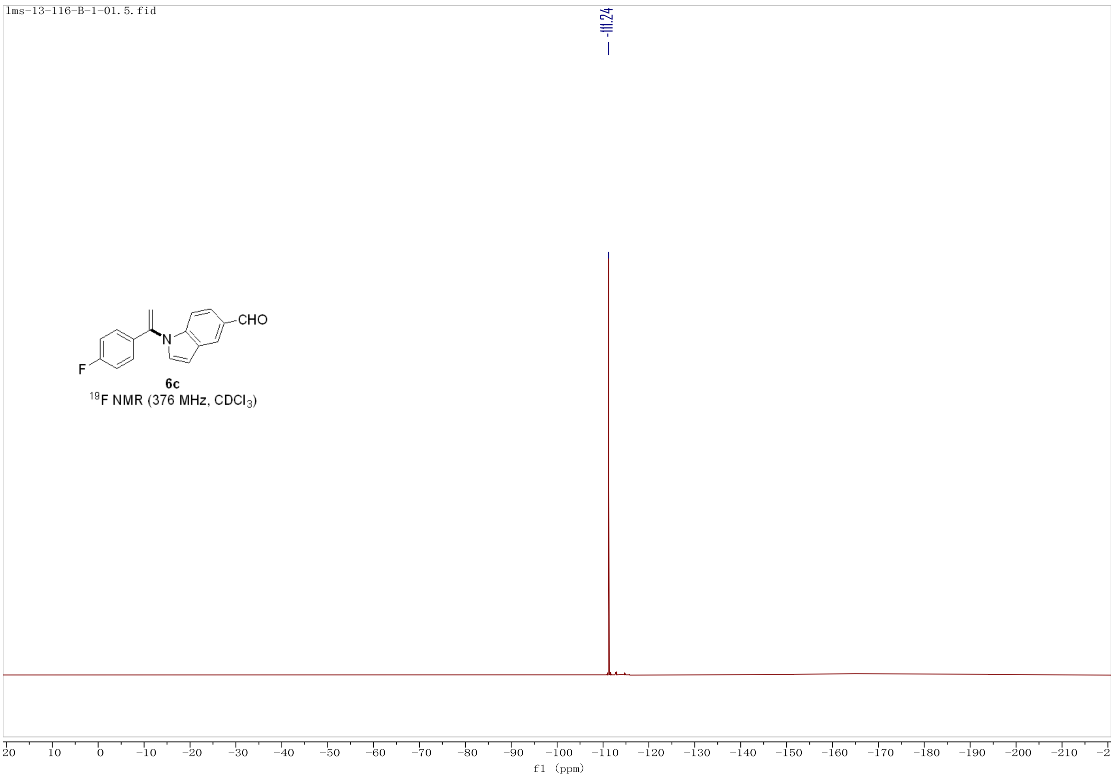
lms-13-116-B-1-01.5.fid
-111.24
CHO
N
F
6c
^{19}F NMR (376 MHz, CDCl$_3$)
f1 (ppm)

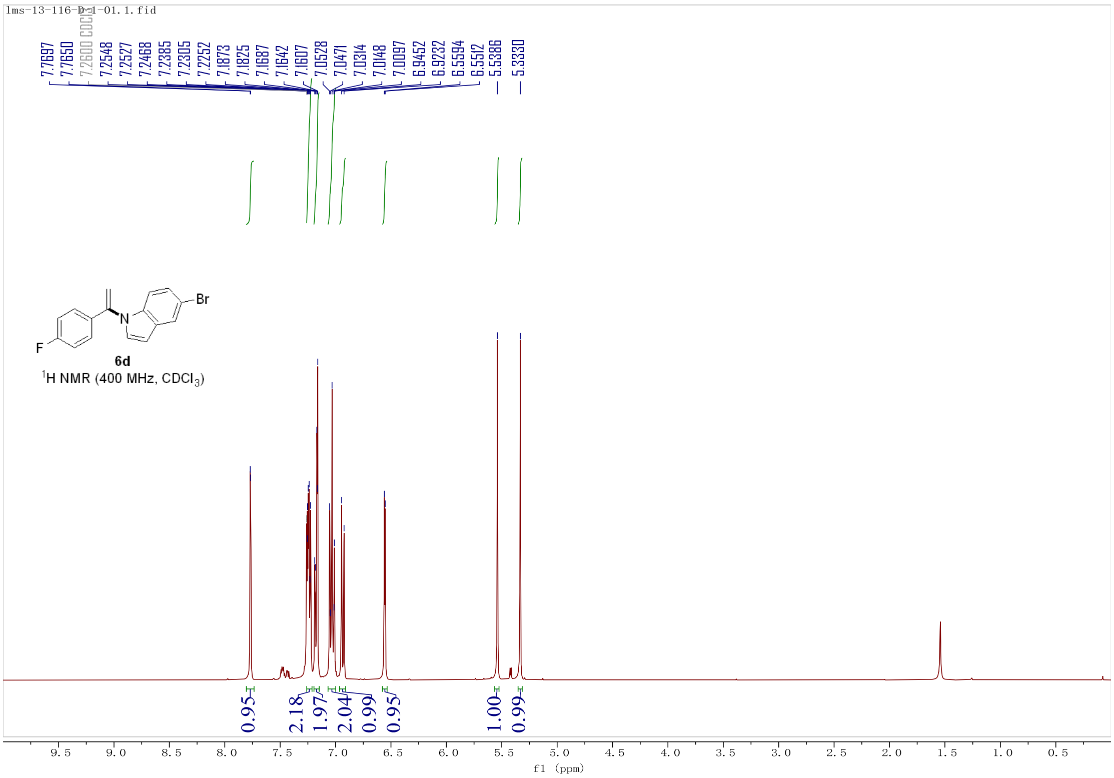
lms-13-116-D-1-01.1.fid
7.7697
7.7650
7.2600 CDCl3
7.2548
7.2527
7.2468
7.2385
7.2305
7.2252
7.1873
7.1825
7.1687
7.1642
7.1607
7.0528
7.0471
7.0314
7.0148
7.0097
6.9452
6.9232
6.5594
6.5512
5.5386
5.3330
Br
N
F
6d
^{1}H NMR (400 MHz, CDCl$_3$)
0.95
2.18
1.97
2.04
0.99
0.95
1.00
0.99
f1 (ppm)

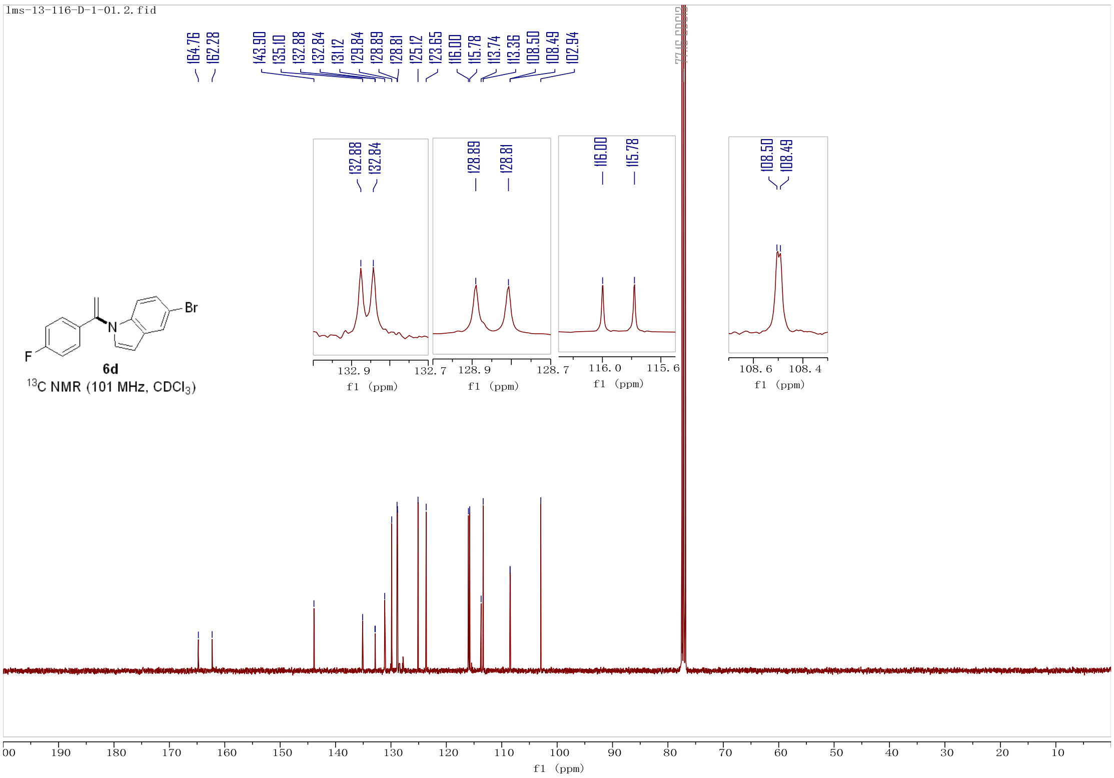
lms-13-116-D-1-01.2.fid
164.76
162.28
143.90
135.10
132.88
132.84
131.12
129.84
128.89
128.81
125.12
123.65
116.00
115.78
113.74
113.36
108.50
108.49
102.94
Br
N
F
6d
^{13}C NMR (101 MHz, CDCl$_3$)
f1 (ppm)

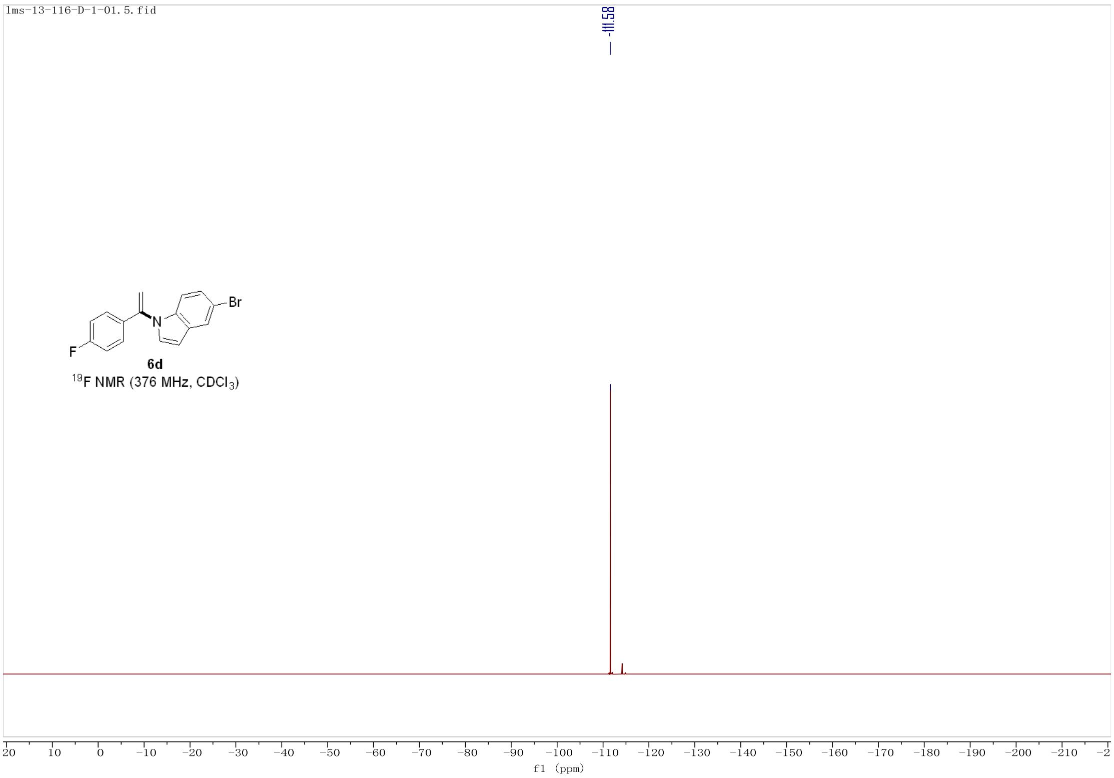
lms-13-116-D-1-01.5.fid
-111.58
Br
N
F
6d
^{19}F NMR (376 MHz, CDCl$_3$)
f1 (ppm)

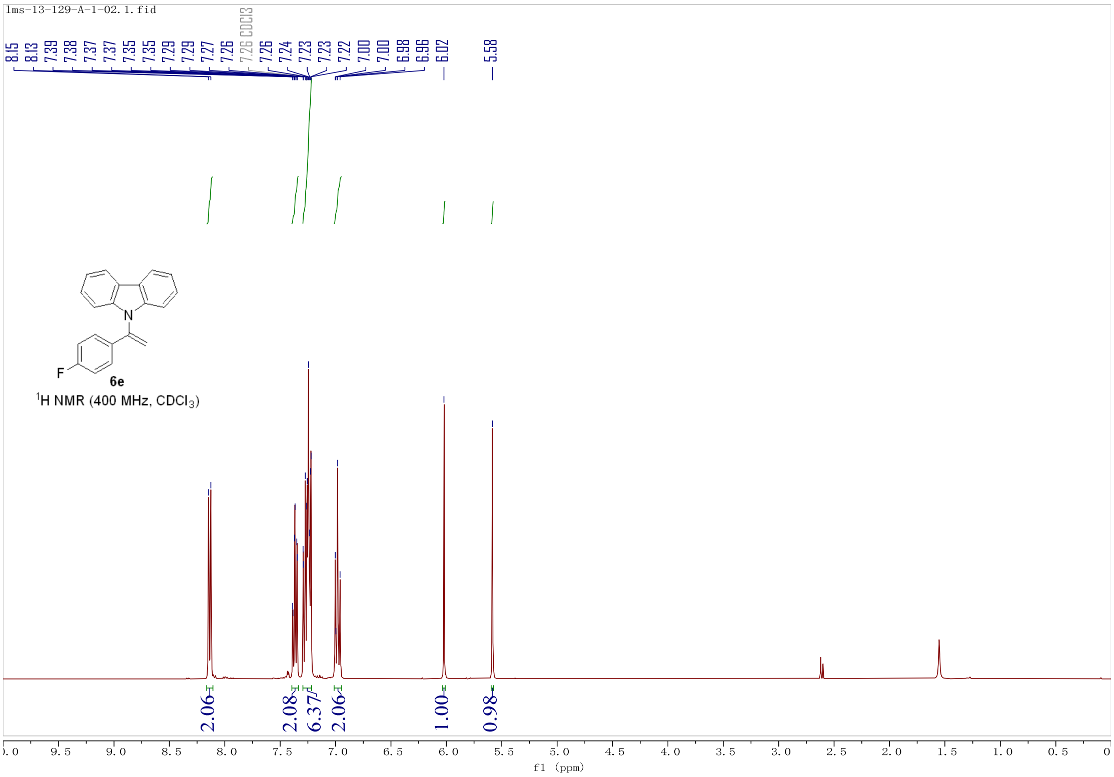
lms-13-129-A-1-02.1.fid
8.15 8.13 7.39 7.38 7.37 7.37 7.35 7.35 7.29 7.29 7.27 7.26 7.26 CDCl3 7.26 7.24 7.23 7.23 7.23 7.22 7.00 7.00 6.98 6.96 6.02 5.58
F
N
6e
1H NMR (400 MHz, CDCl3)
2.06 2.08 6.37 2.06 1.00 0.98
f1 (ppm)

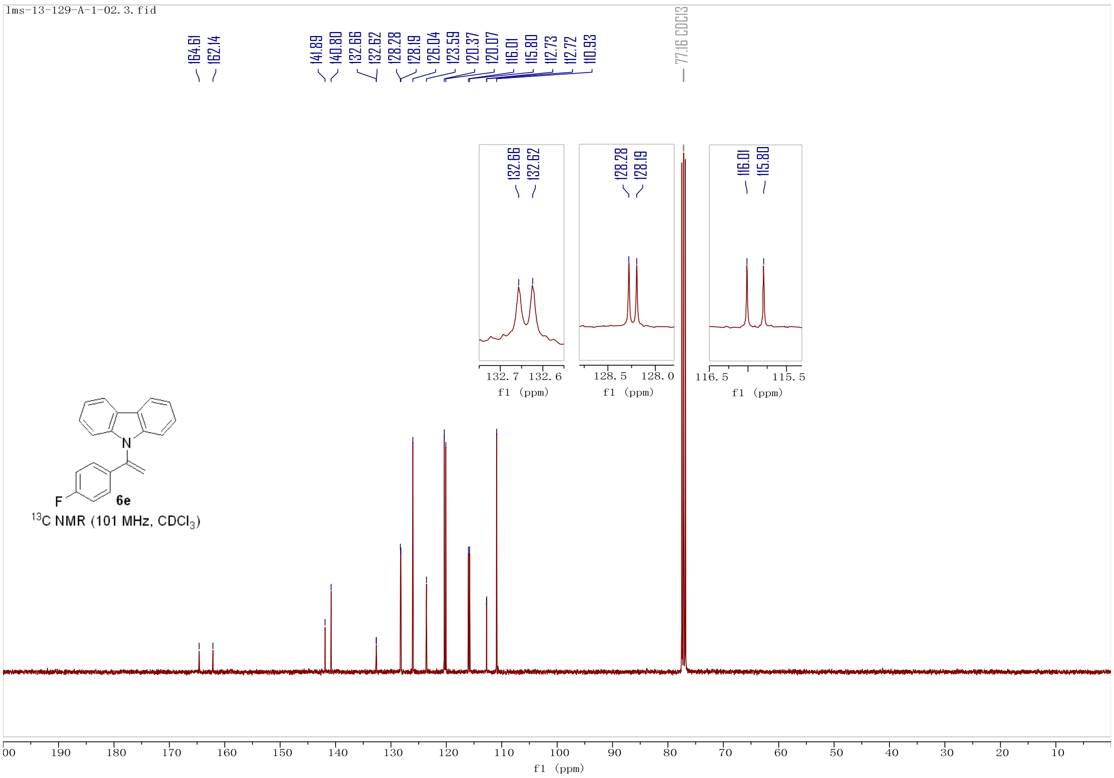
lms-13-129-A-1-02.3.fid
164.61 162.14 141.89 140.80 132.66 132.62 128.28 128.19 126.04 123.59 120.37 120.07 116.01 115.80 112.73 112.72 110.93 77.16 CDCl3
132.66 132.62
128.28 128.19
116.01 115.80
f1 (ppm)
F
N
6e
13C NMR (101 MHz, CDCl3)
f1 (ppm)

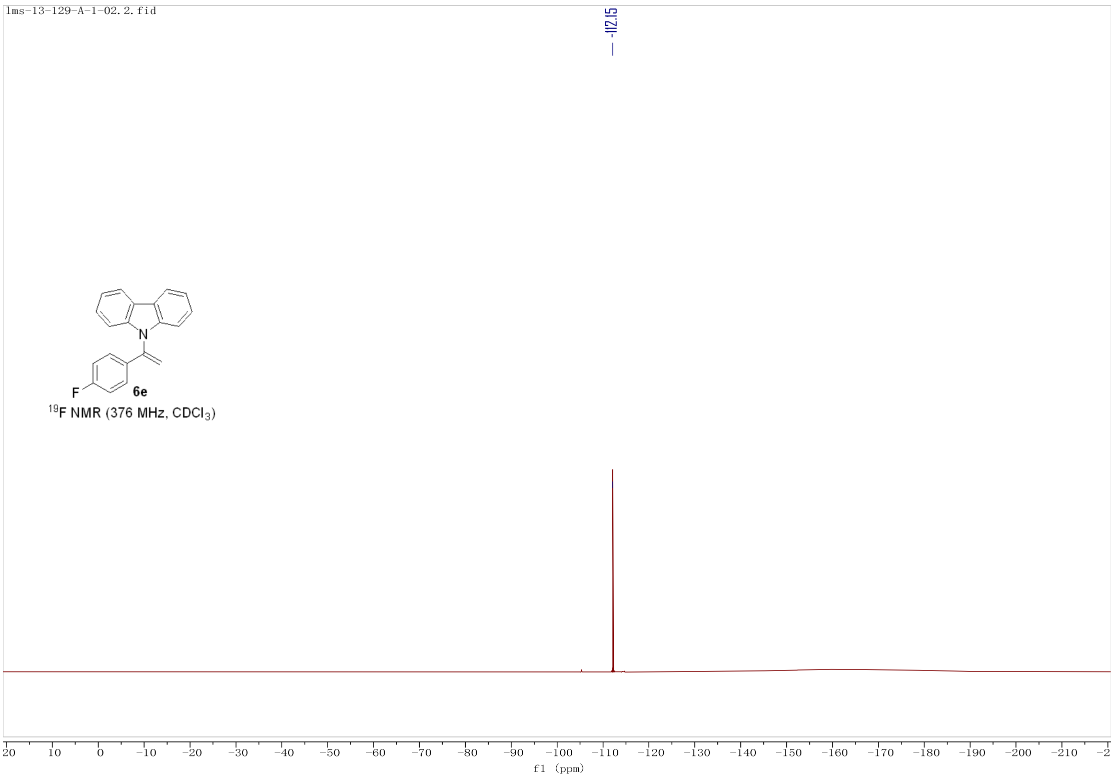
lms-13-129-A-1-02.2.fid
-112.15
F
N
6e
19F NMR (376 MHz, CDCl3)
20 10 0 −10 −20 −30 −40 −50 −60 −70 −80 −90 −100 −110 −120 −130 −140 −150 −160 −170 −180 −190 −200 −210
f1 (ppm)

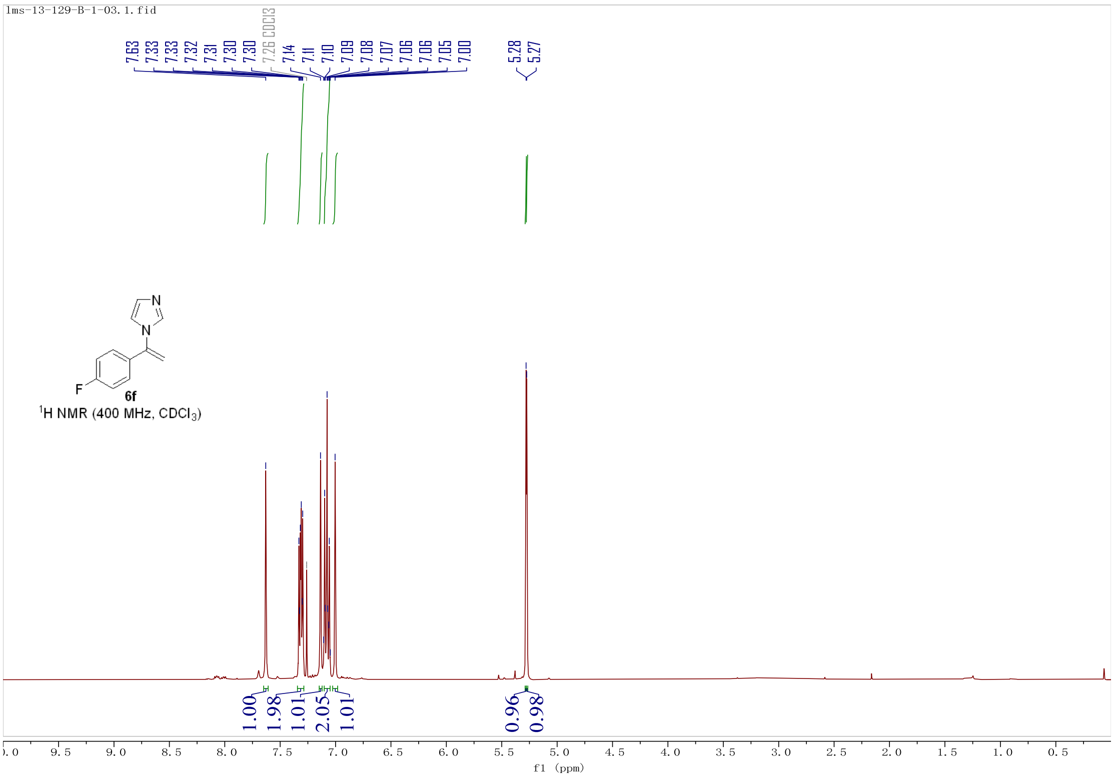
lms-13-129-B-1-03.1.fid
7.63 7.33 7.33 7.32 7.31 7.30 7.30 7.26 CDCl3 7.14 7.11 7.10 7.09 7.08 7.07 7.06 7.06 7.05 7.00
5.28 5.27
N
N
F
6f
1H NMR (400 MHz, CDCl3)
1.00 1.98 1.01 2.05 1.01
0.96 0.98
9.5 9.0 8.5 8.0 7.5 7.0 6.5 6.0 5.5 5.0 4.5 4.0 3.5 3.0 2.5 2.0 1.5 1.0 0.5
f1 (ppm)

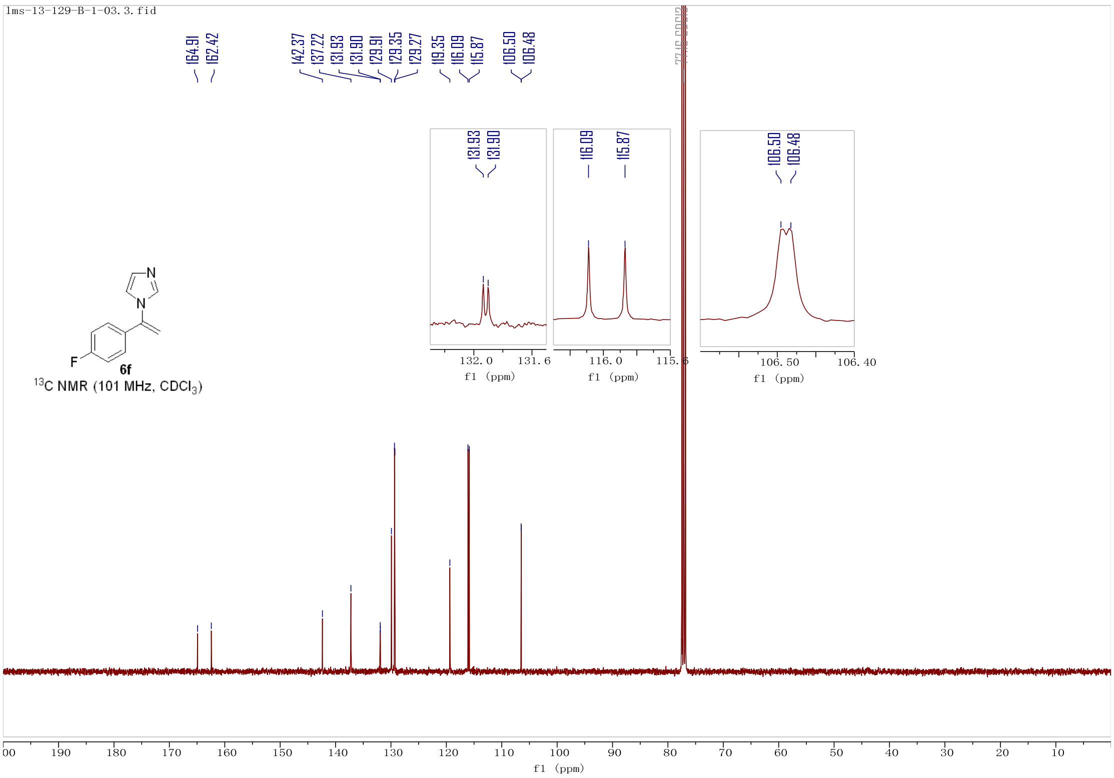
lms-13-129-B-1-03.3.fid
164.91
162.42
142.37
137.22
131.93
131.90
129.91
129.35
129.27
119.35
116.09
115.87
106.50
106.48
6f
^{13}C NMR (101 MHz, $CDCl_3$)
f1 (ppm)

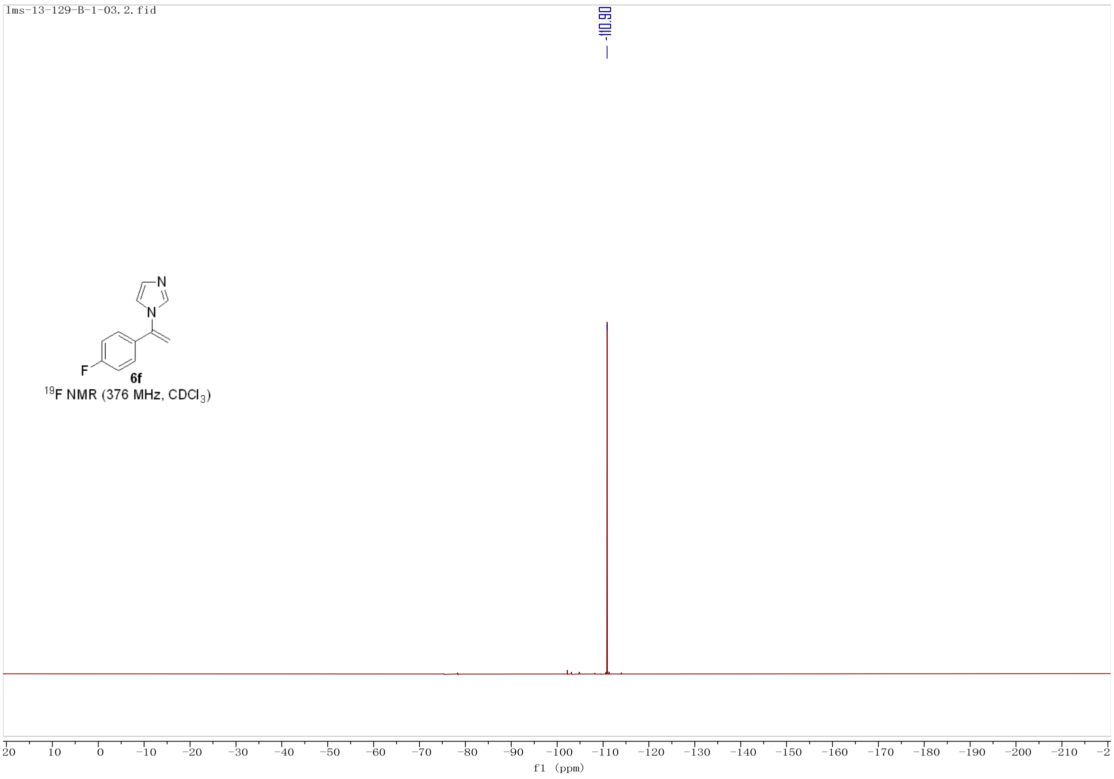
lms-13-129-B-1-03.2.fid
-110.90
6f
^{19}F NMR (376 MHz, $CDCl_3$)
f1 (ppm)

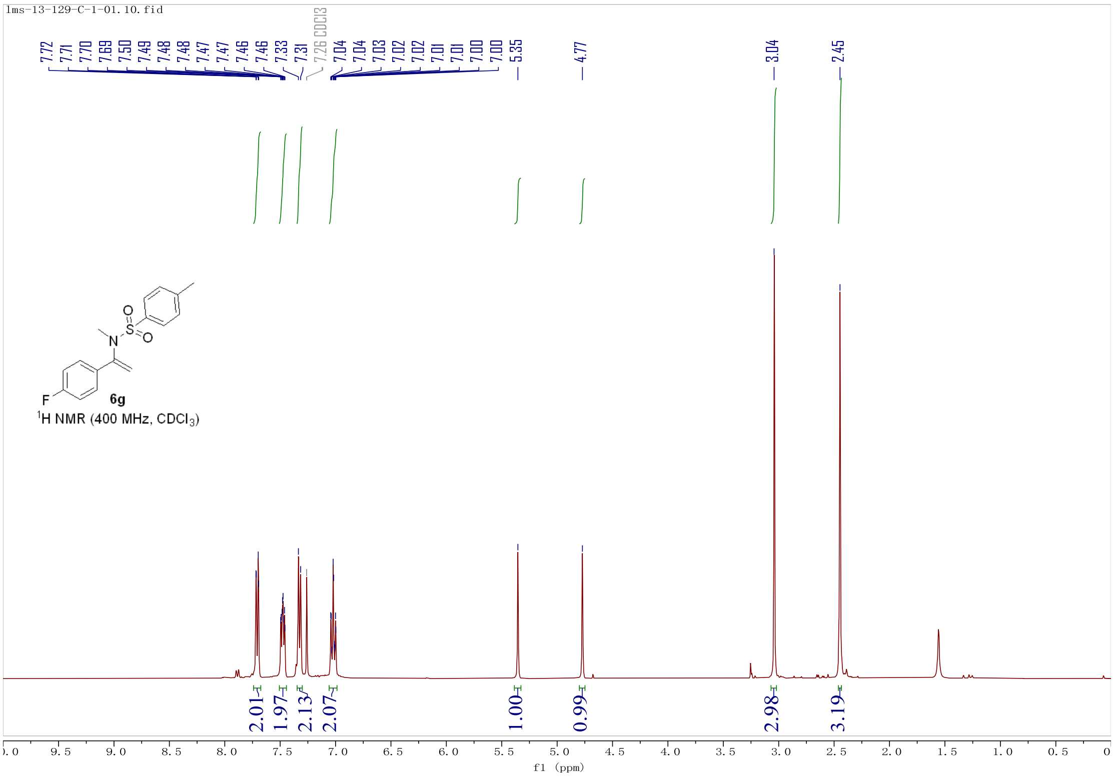
lms-13-129-C-1-01.10.fid
7.72 7.71 7.70 7.69 7.50 7.49 7.48 7.48 7.47 7.47 7.46 7.46 7.33 7.31 7.26 CDCl3 7.04 7.04 7.03 7.02 7.02 7.01 7.01 7.00 7.00 5.35 4.77 3.04 2.45
6g
1H NMR (400 MHz, CDCl3)
2.01 1.97 2.13 2.07 1.00 0.99 2.98 3.19
f1 (ppm)

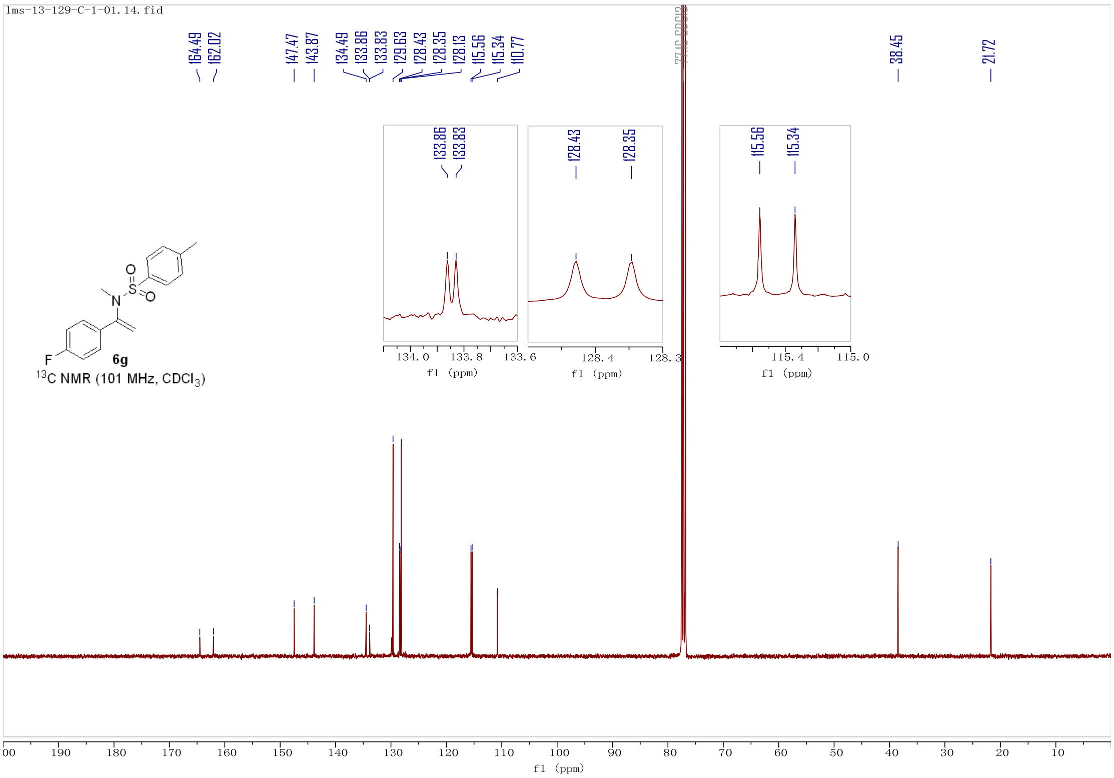
lms-13-129-C-1-01.14.fid
164.49 162.02 147.47 143.87 134.49 133.86 133.83 129.63 128.43 128.35 128.13 115.56 115.34 110.77 38.45 21.72
6g
13C NMR (101 MHz, CDCl3)
f1 (ppm)

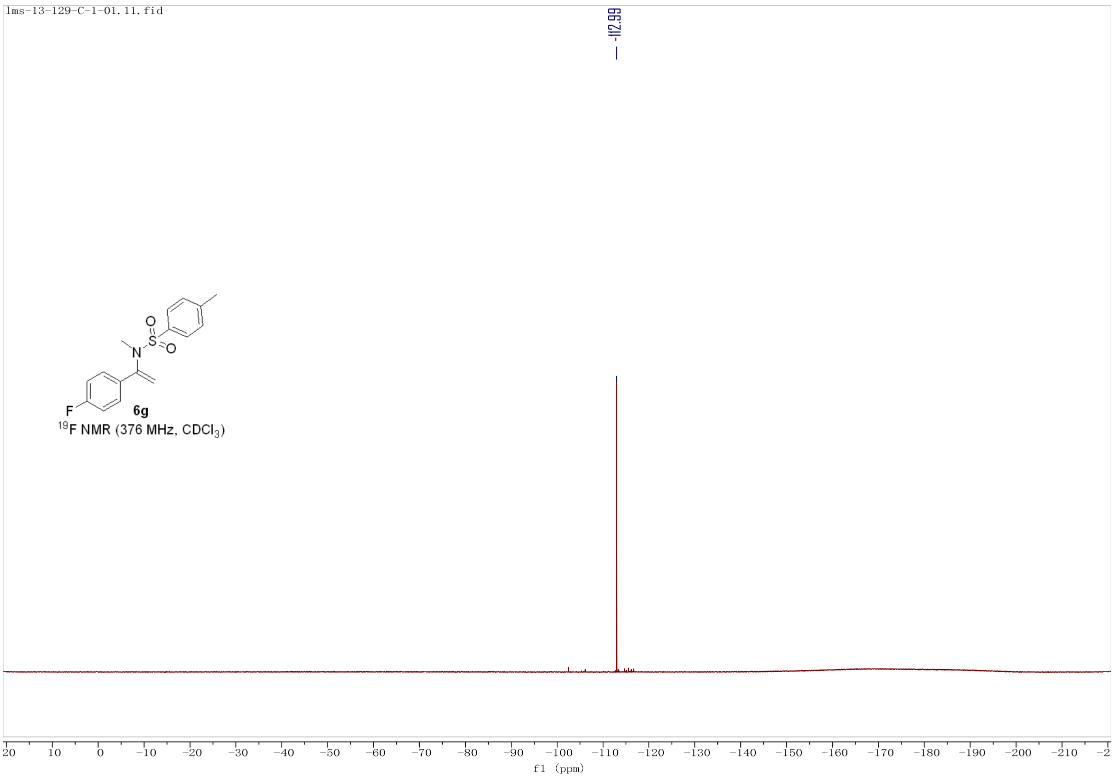

lms-13-129-C-1-01.11.fid
-112.99
F
6g
^{19}F NMR (376 MHz, CDCl$_3$)
f1 (ppm)

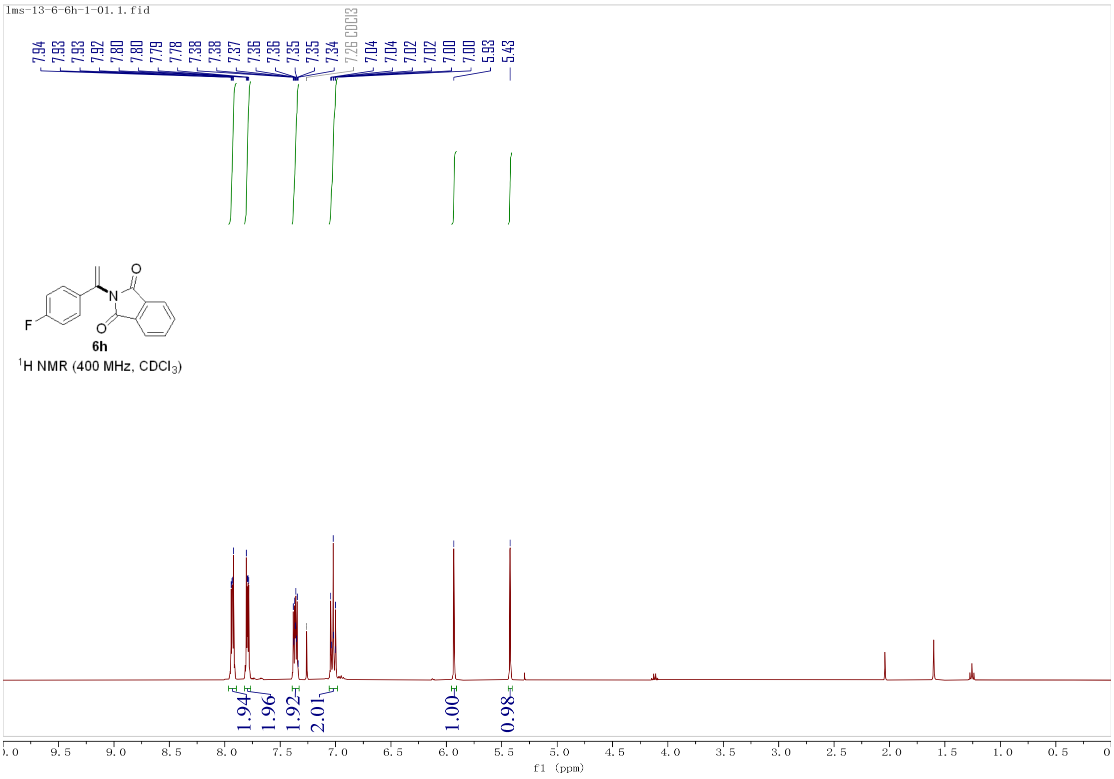

lms-13-6-6h-1-01.1.fid
7.94 7.93 7.93 7.92 7.80 7.79 7.78 7.38 7.38 7.37 7.36 7.36 7.35 7.35 7.34 7.26 CDCl3 7.04 7.04 7.02 7.02 7.00 7.00 5.93 5.43
F
6h
^{1}H NMR (400 MHz, CDCl$_3$)
1.94 1.96 1.92 2.01 1.00 0.98
f1 (ppm)

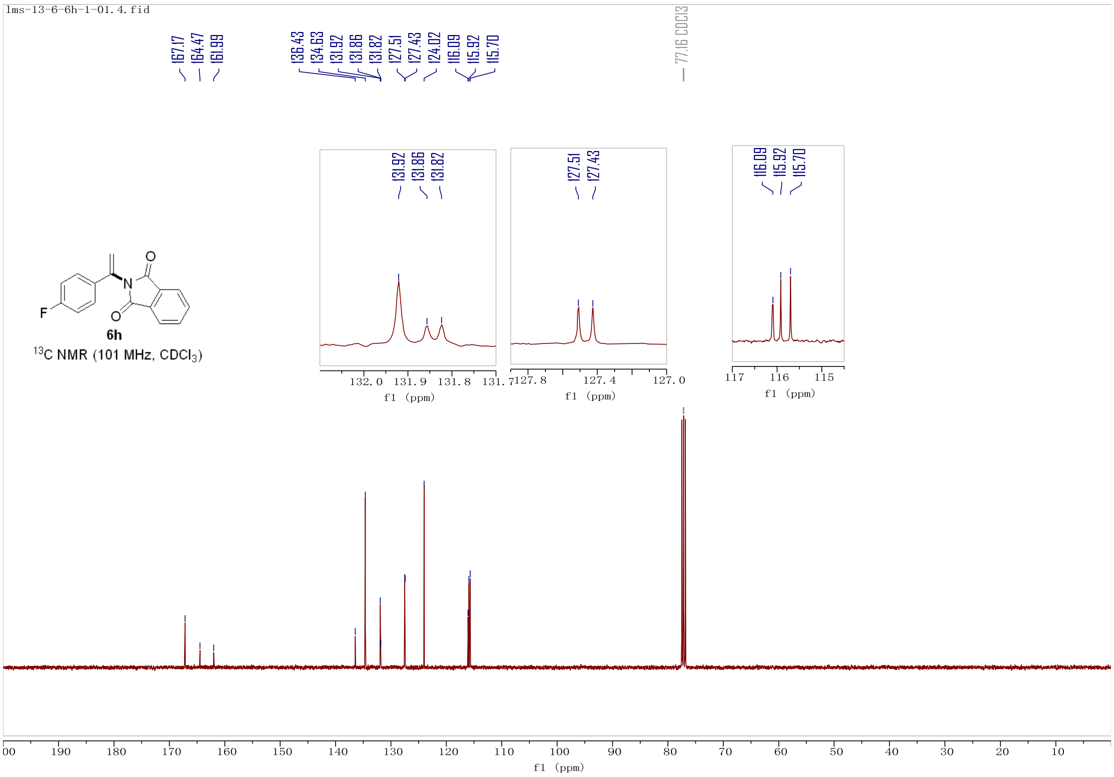
lms-13-6-6h-1-01.4.fid
167.17
164.47
161.99
136.43
134.63
131.92
131.86
131.82
127.51
127.43
124.02
116.09
115.92
115.70
77.16 CDCl3
6h
^{13}C NMR (101 MHz, $CDCl_3$)
f1 (ppm)

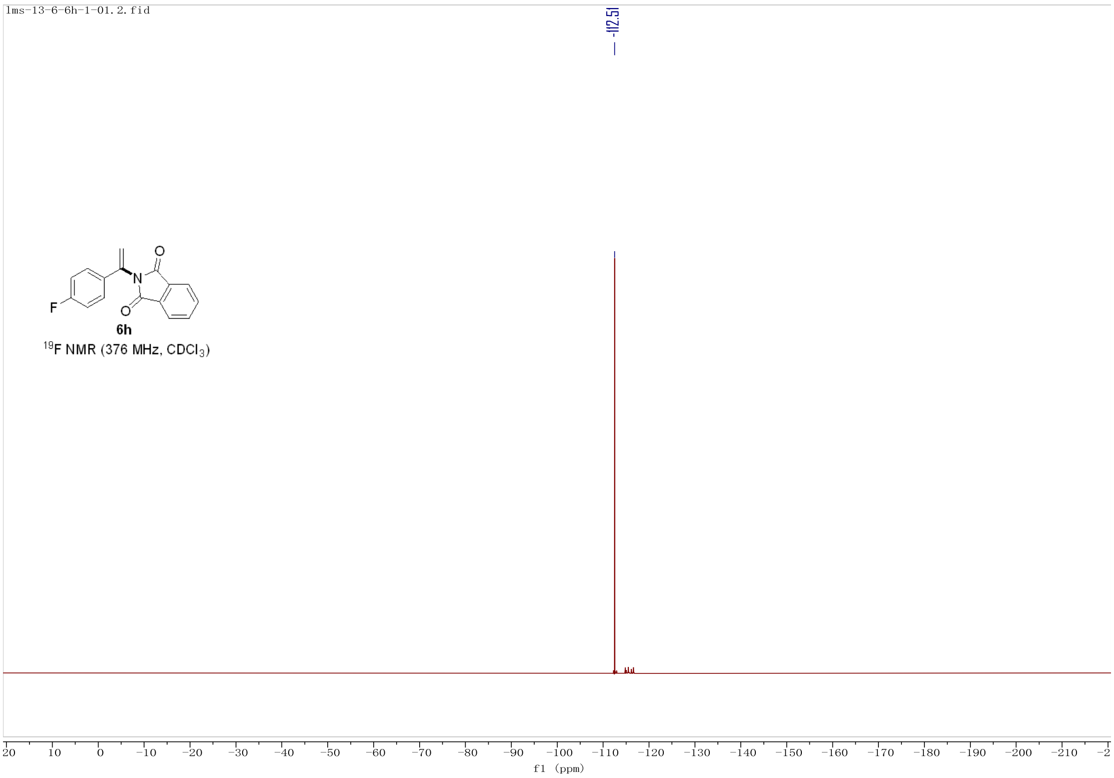
lms-13-6-6h-1-01.2.fid
-112.51
6h
^{19}F NMR (376 MHz, $CDCl_3$)
f1 (ppm)

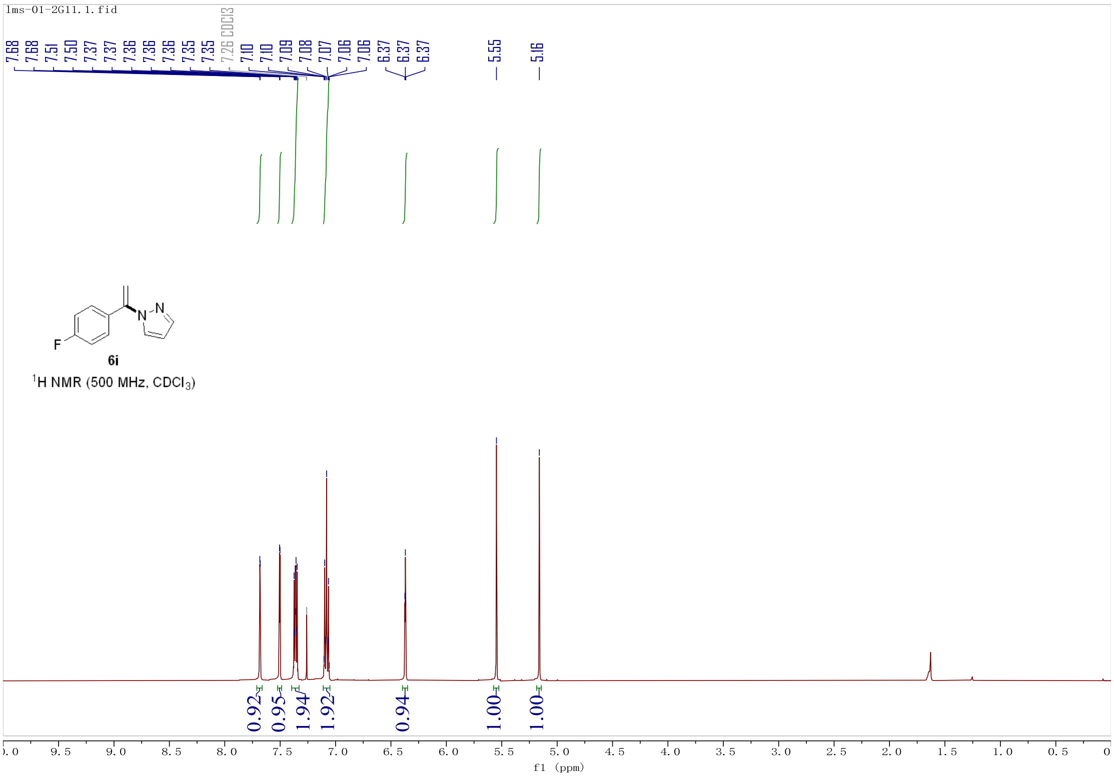
lms-01-2G11.1.fid
7.68 7.68 7.51 7.50 7.37 7.37 7.36 7.36 7.36 7.35 7.35 7.26 CDCl3 7.10 7.10 7.09 7.08 7.07 7.06 7.06 6.37 6.37 6.37 5.55 5.16
F N N
6i
^{1}H NMR (500 MHz, CDCl$_3$)
0.92 0.95 1.94 1.92 0.94 1.00 1.00
f1 (ppm)

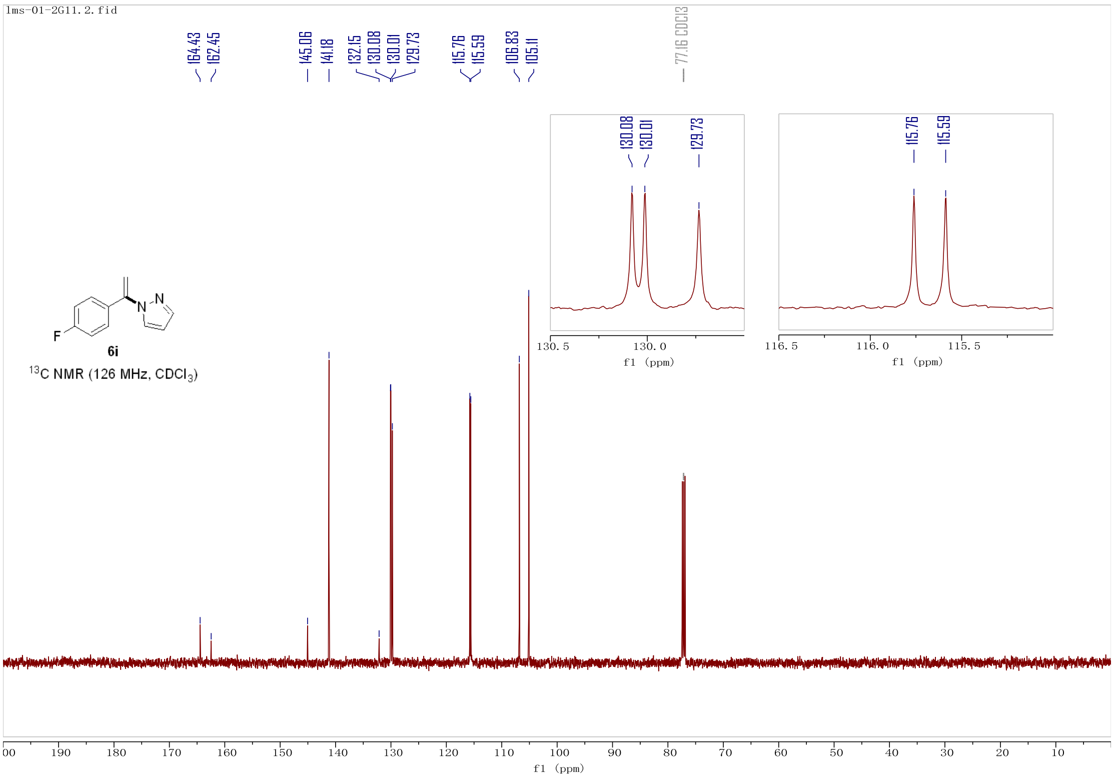
lms-01-2G11.2.fid
164.43 162.45 145.06 141.18 132.15 130.08 130.01 129.73 115.76 115.59 106.83 105.11 77.16 CDCl3
F N N
6i
^{13}C NMR (126 MHz, CDCl$_3$)
130.08 130.01 129.73
115.76 115.59
f1 (ppm)

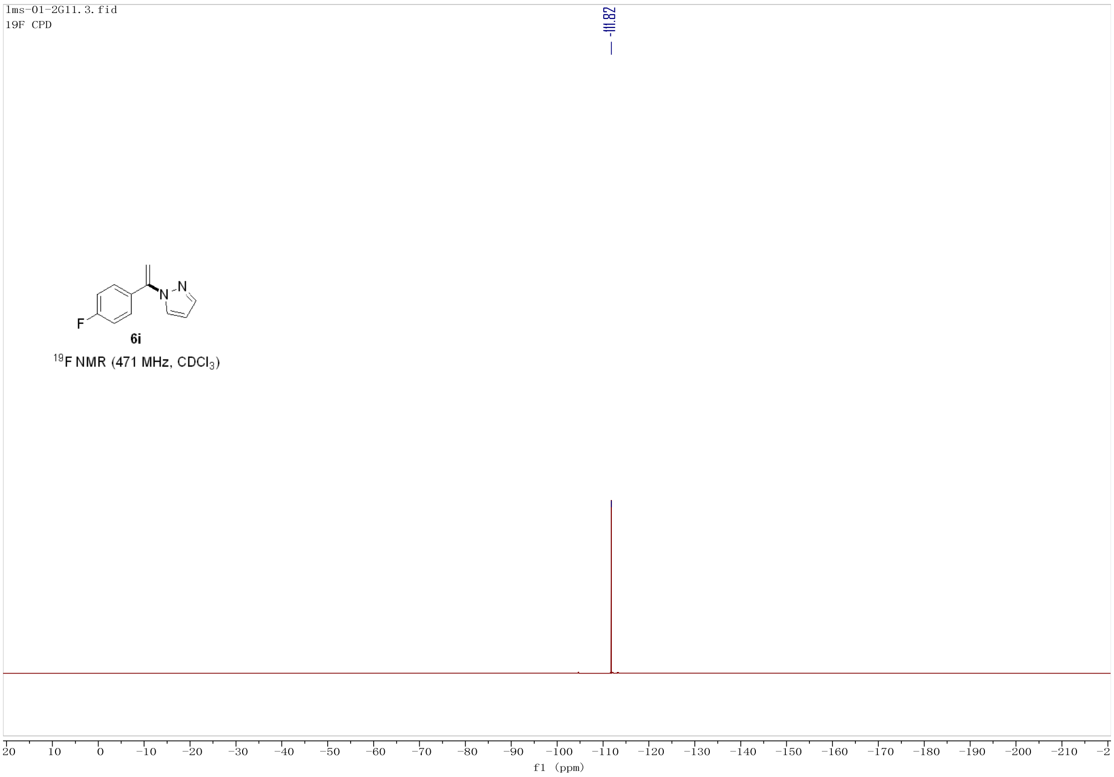
lms-01-2G11.3.fid
19F CPD
-111.82
F
N
N
6i
^{19}F NMR (471 MHz, CDCl$_3$)
f1 (ppm)

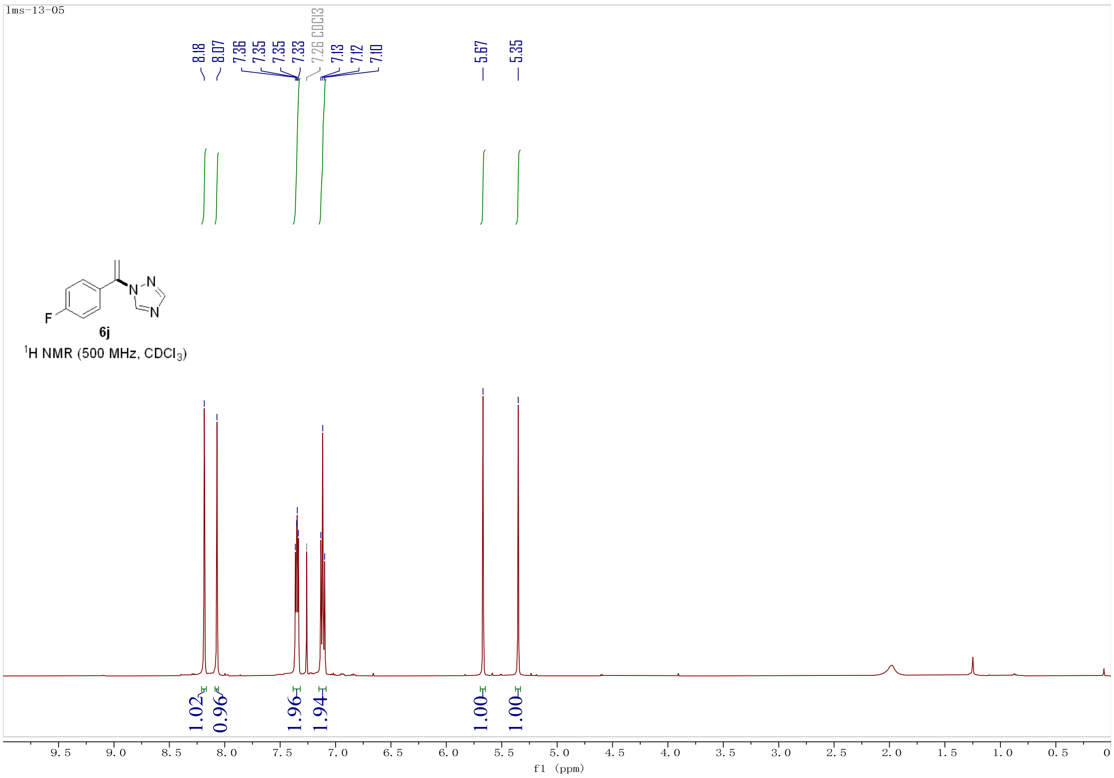
lms-13-05
8.18
8.07
7.36
7.35
7.35
7.33
7.26 CDCl3
7.13
7.12
7.10
5.67
5.35
F
N
N
N
6j
^{1}H NMR (500 MHz, CDCl$_3$)
1.02
0.96
1.96
1.94
1.00
1.00
f1 (ppm)

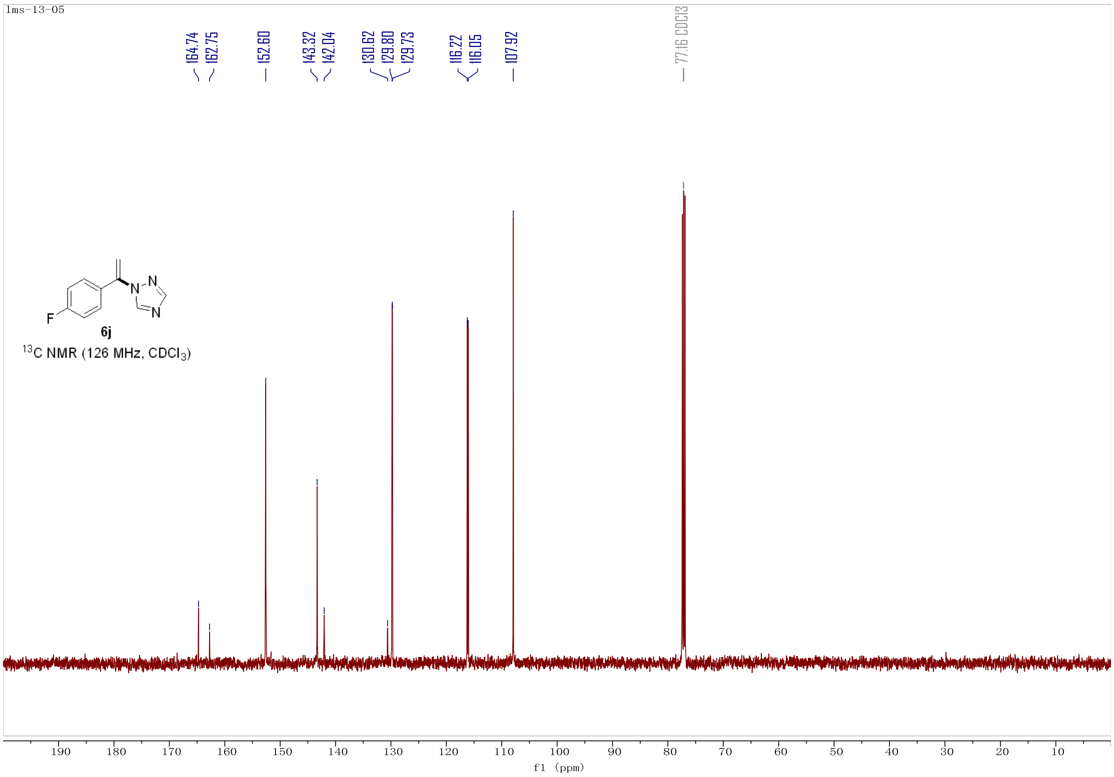

6j

^{13}C NMR (126 MHz, $CDCl_3$)

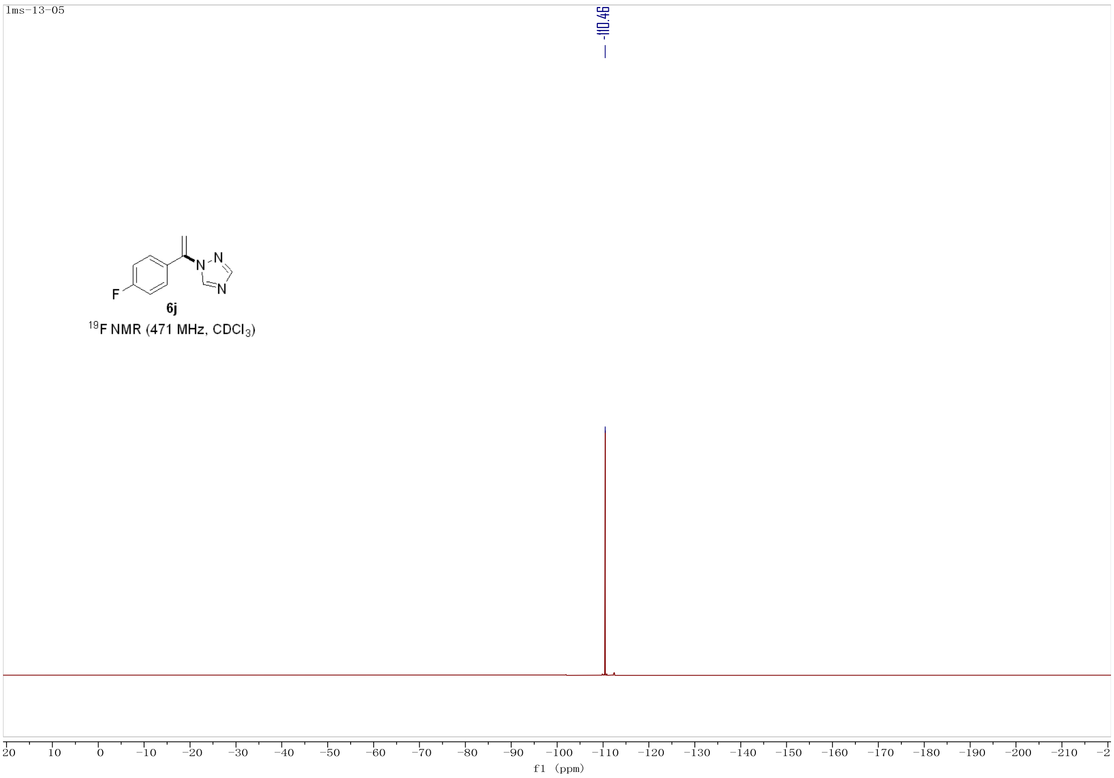

6j

^{19}F NMR (471 MHz, $CDCl_3$)

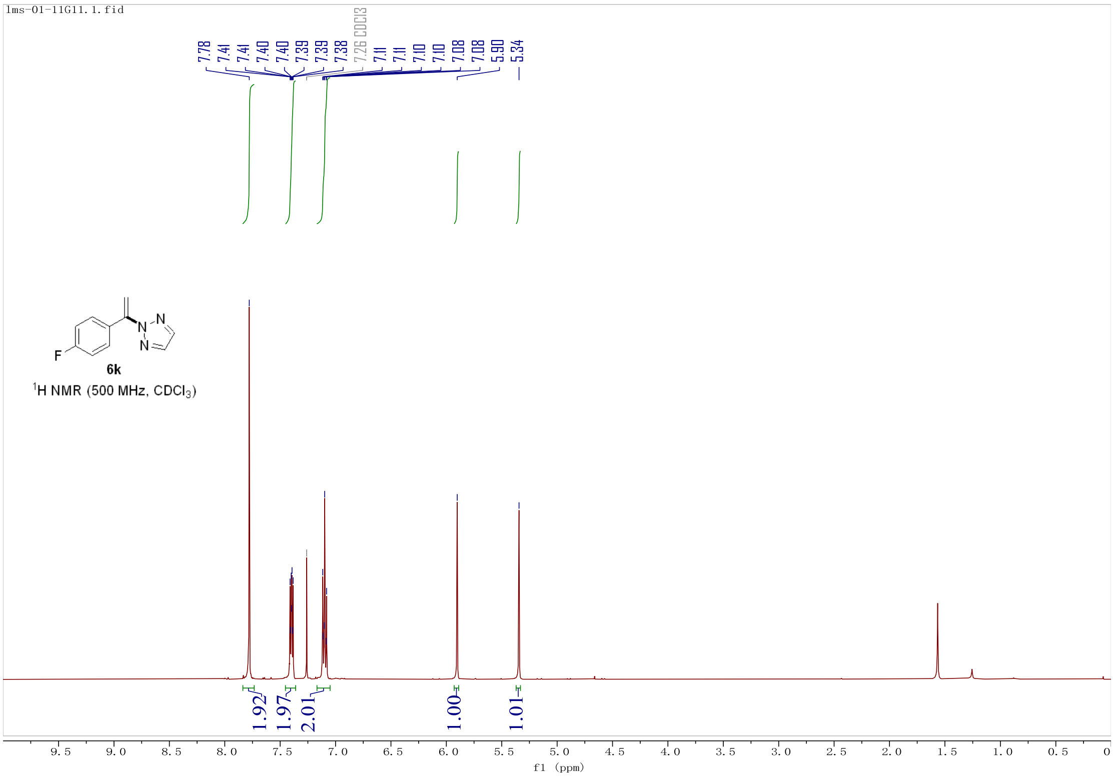
lms-01-11G11.1.fid
7.78
7.41
7.41
7.40
7.40
7.39
7.39
7.38
7.26 CDCl3
7.11
7.11
7.10
7.10
7.08
7.08
5.90
5.34
6k
1H NMR (500 MHz, CDCl3)
1.92
1.97
2.01
1.00
1.01
f1 (ppm)

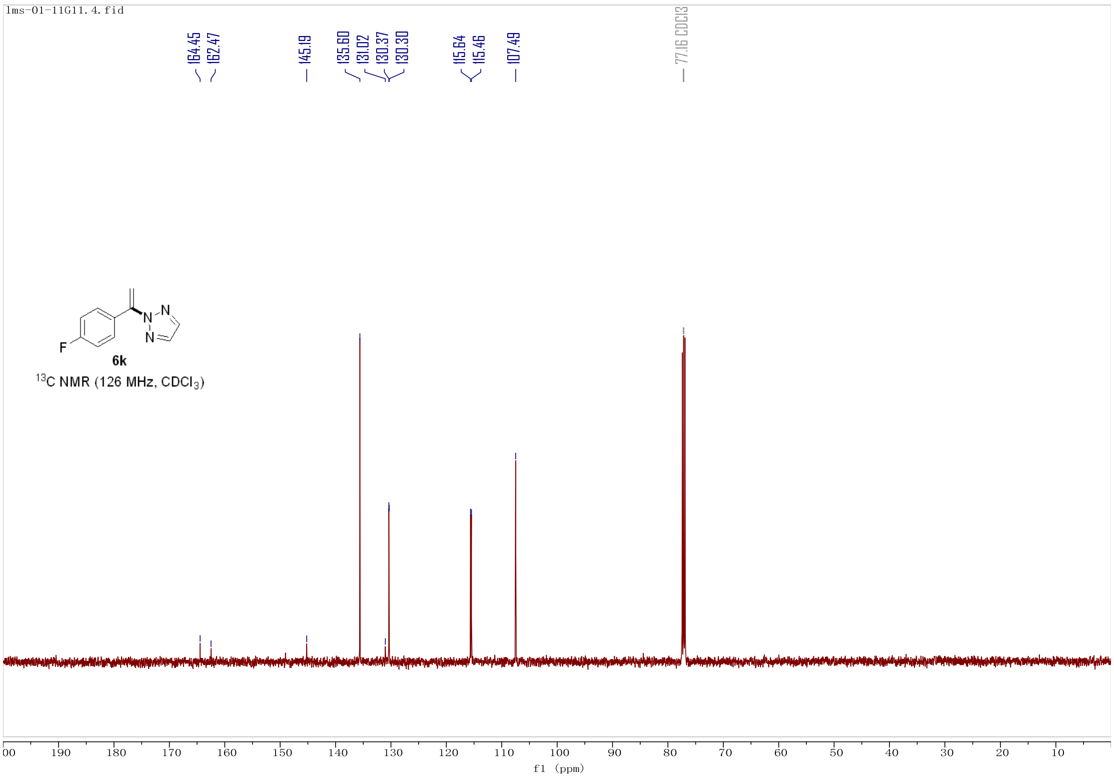
lms-01-11G11.4.fid
164.45
162.47
145.19
135.60
131.02
130.37
130.30
115.64
115.46
107.49
77.16 CDCl3
6k
13C NMR (126 MHz, CDCl3)
f1 (ppm)

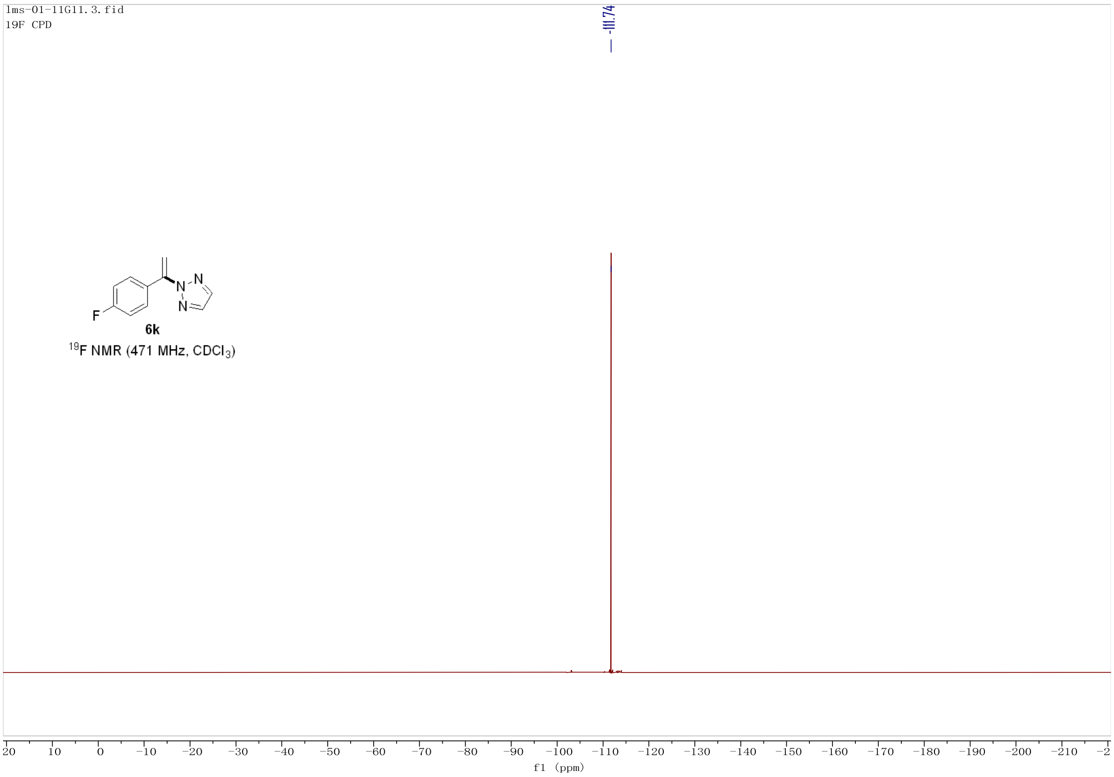
lms-01-11G11.3.fid
19F CPD
-111.74
6k
19F NMR (471 MHz, CDCl3)
f1 (ppm)

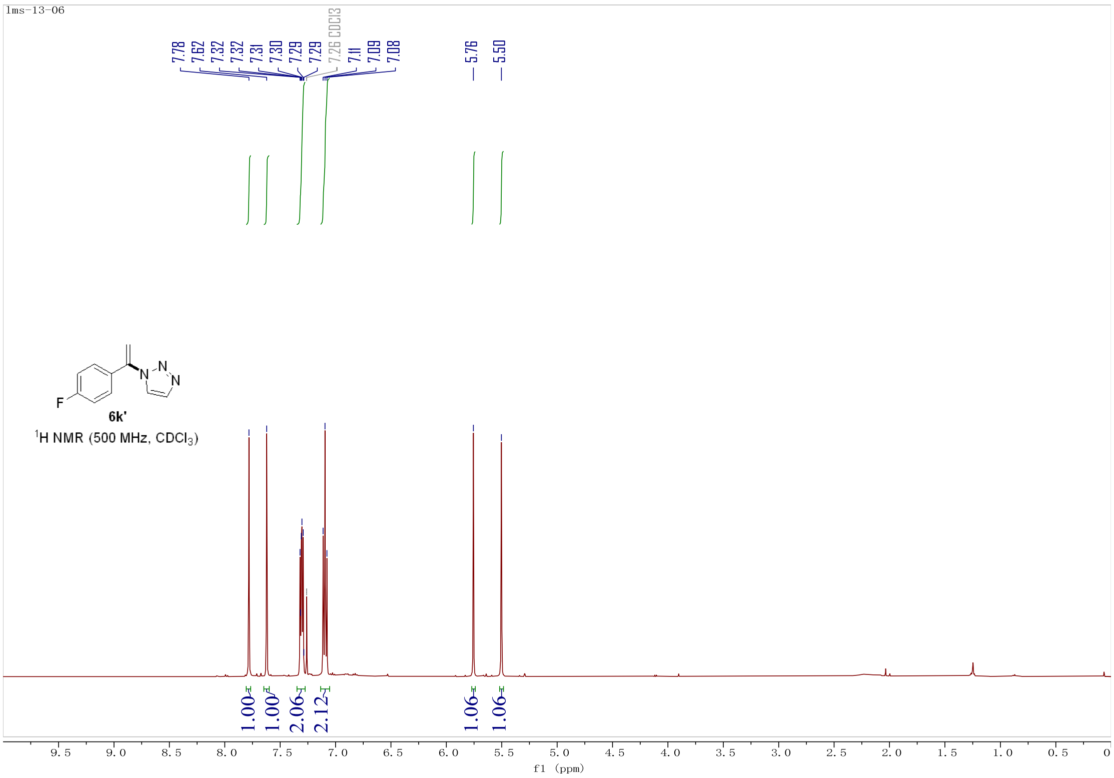
lms-13-06
7.78
7.62
7.32
7.32
7.31
7.30
7.29
7.29
7.26 CDCl3
7.11
7.09
7.08
5.76
5.50
6k'
1H NMR (500 MHz, CDCl3)
1.00
1.00
2.06
2.12
1.06
1.06
f1 (ppm)

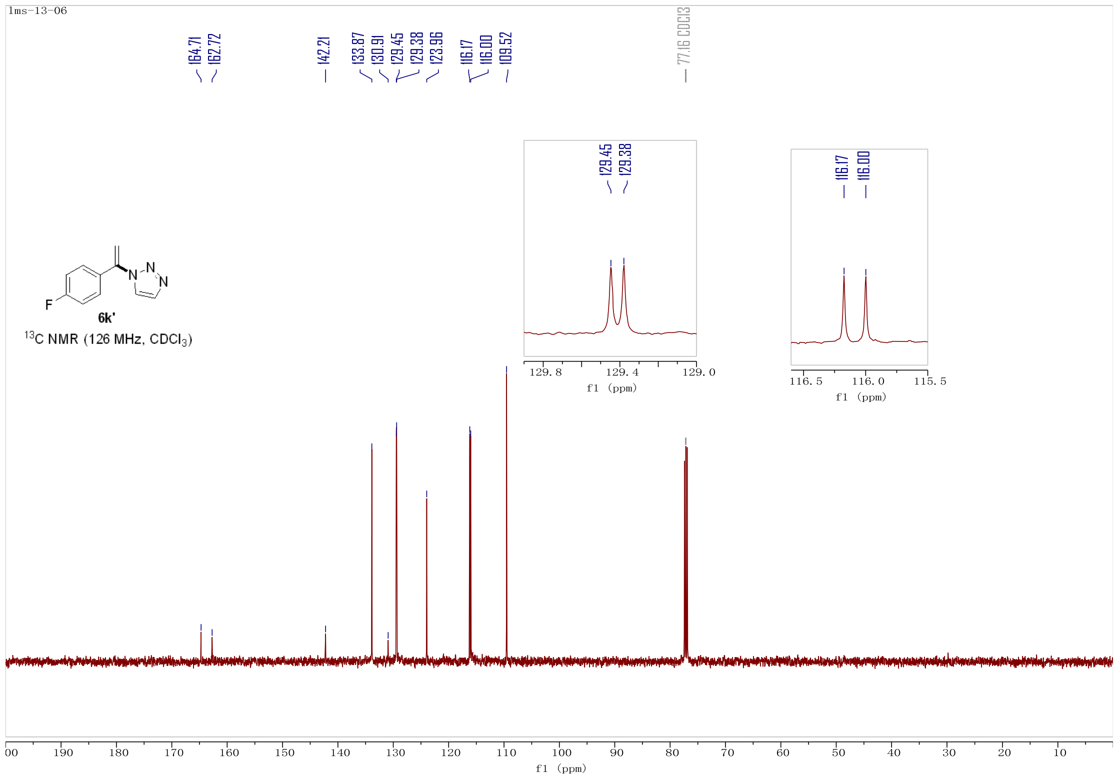

lms-13-06
164.71
162.72
142.21
133.87
130.91
129.45
129.38
123.96
116.17
116.00
109.52
77.16 CDCl3
6k'
^{13}C NMR (126 MHz, $CDCl_3$)
f1 (ppm)

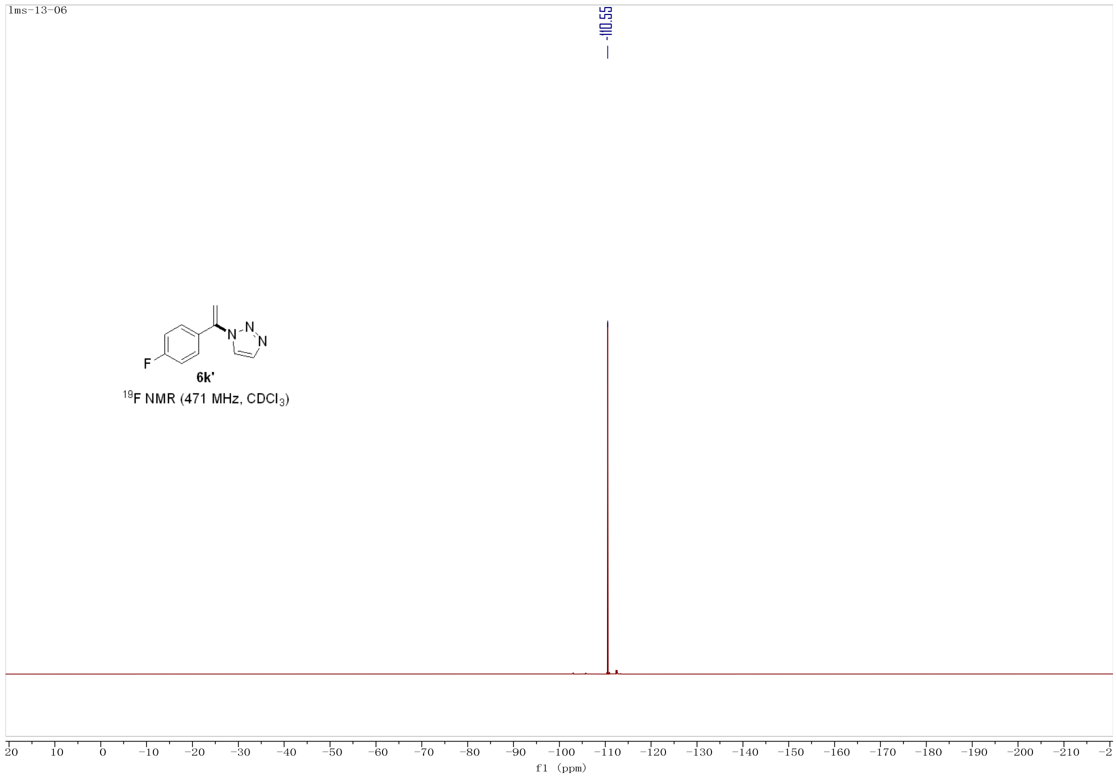

lms-13-06
-110.55
6k'
^{19}F NMR (471 MHz, $CDCl_3$)
f1 (ppm)

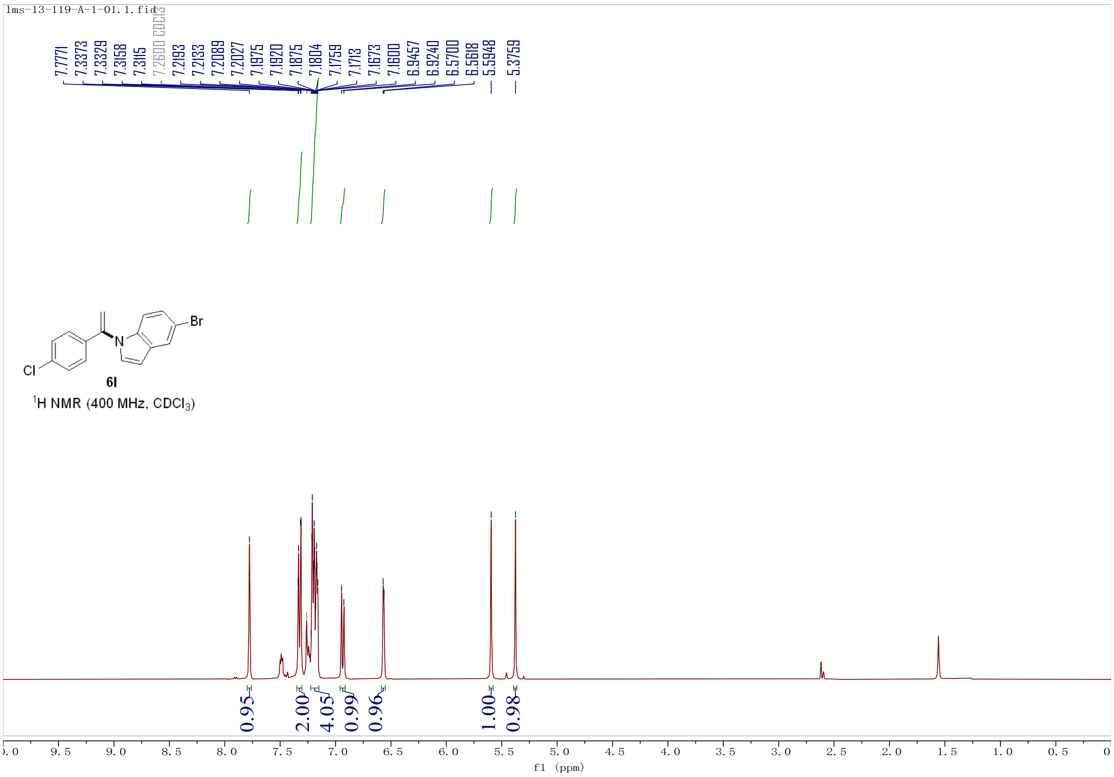
lms-13-119-A-1-01.1.fid
7.7771
7.3373
7.3329
7.3158
7.3115
7.2600 CDCl3
7.2193
7.2133
7.2089
7.2027
7.1975
7.1920
7.1875
7.1804
7.1759
7.1713
7.1673
7.1600
6.9457
6.9240
6.5700
6.5618
5.5948
5.3759
Br
N
Cl
6l
¹H NMR (400 MHz, CDCl3)
0.95
2.00
4.05
0.99
0.96
1.00
0.98
f1 (ppm)

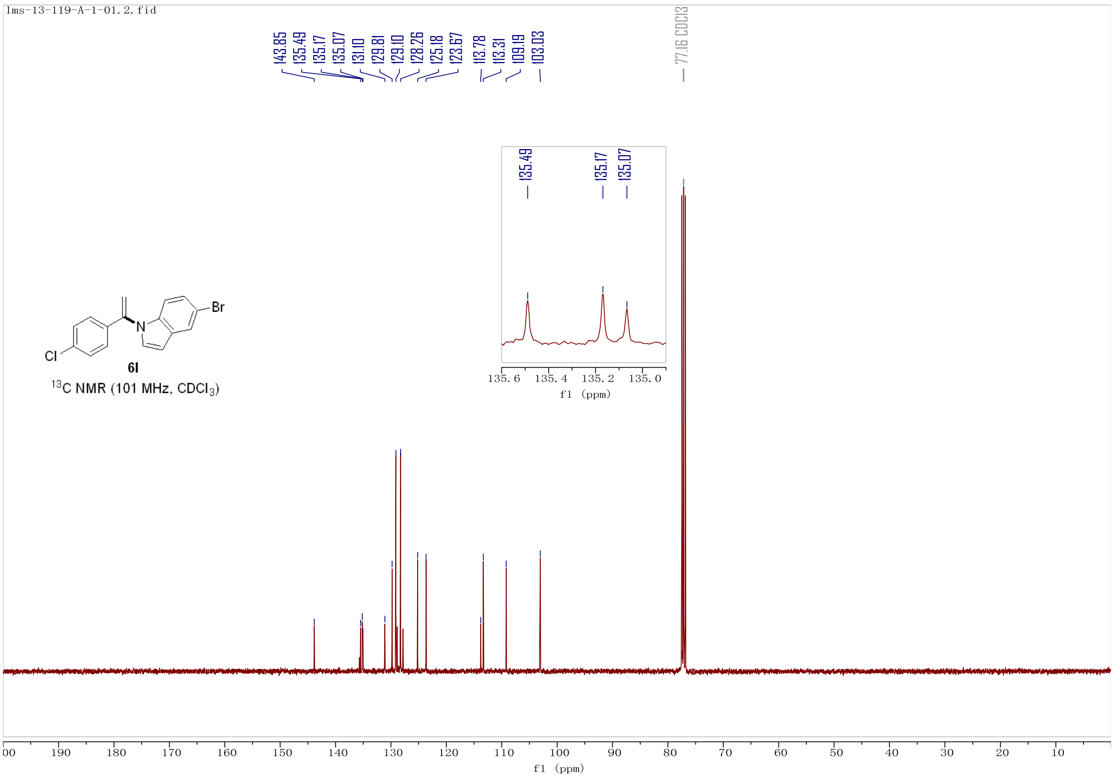
lms-13-119-A-1-01.2.fid
143.85
135.49
135.17
135.07
131.10
129.81
129.10
128.25
125.18
123.67
113.78
113.31
109.19
103.03
77.16 CDCl3
135.49
135.17
135.07
f1 (ppm)
Br
N
Cl
6l
¹³C NMR (101 MHz, CDCl3)
f1 (ppm)

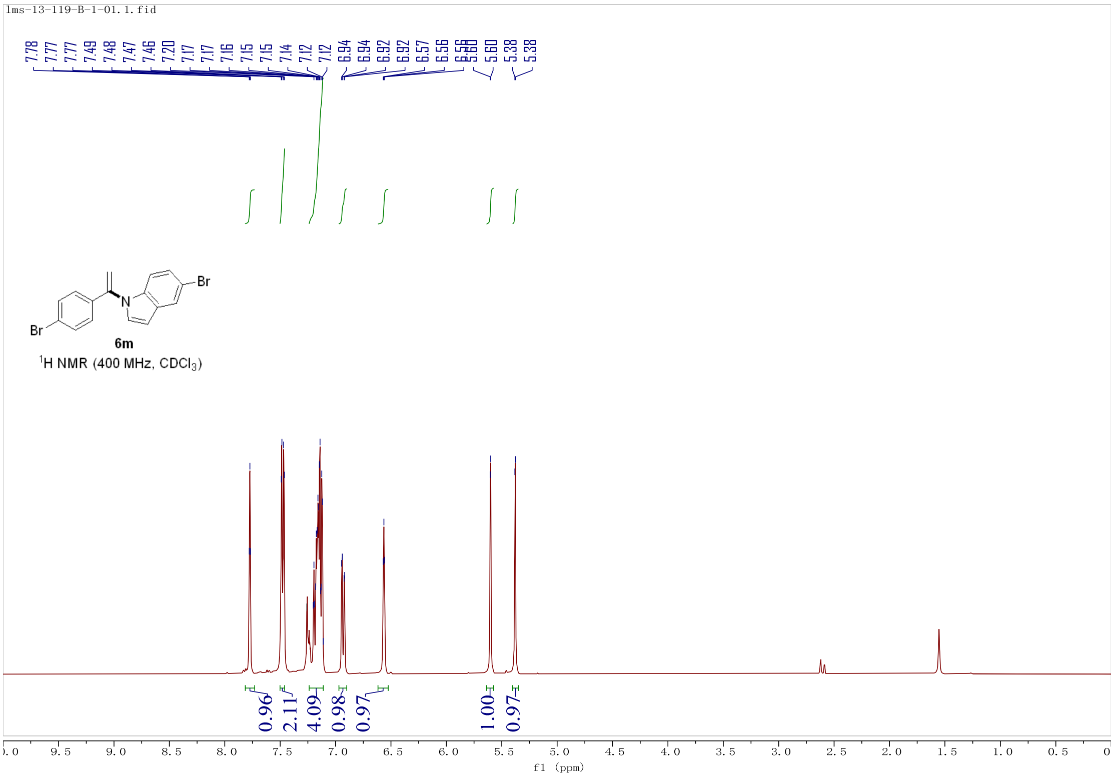
lms-13-119-B-1-01.1.fid
7.78 7.77 7.77 7.49 7.48 7.47 7.46 7.20 7.17 7.17 7.16 7.15 7.15 7.14 7.12 7.12 6.94 6.94 6.92 6.92 6.57 6.56 5.60 5.38 5.38
Br
Br
N
6m
1H NMR (400 MHz, CDCl3)
0.96 2.11 4.09 0.98 0.97 1.00 0.97
f1 (ppm)

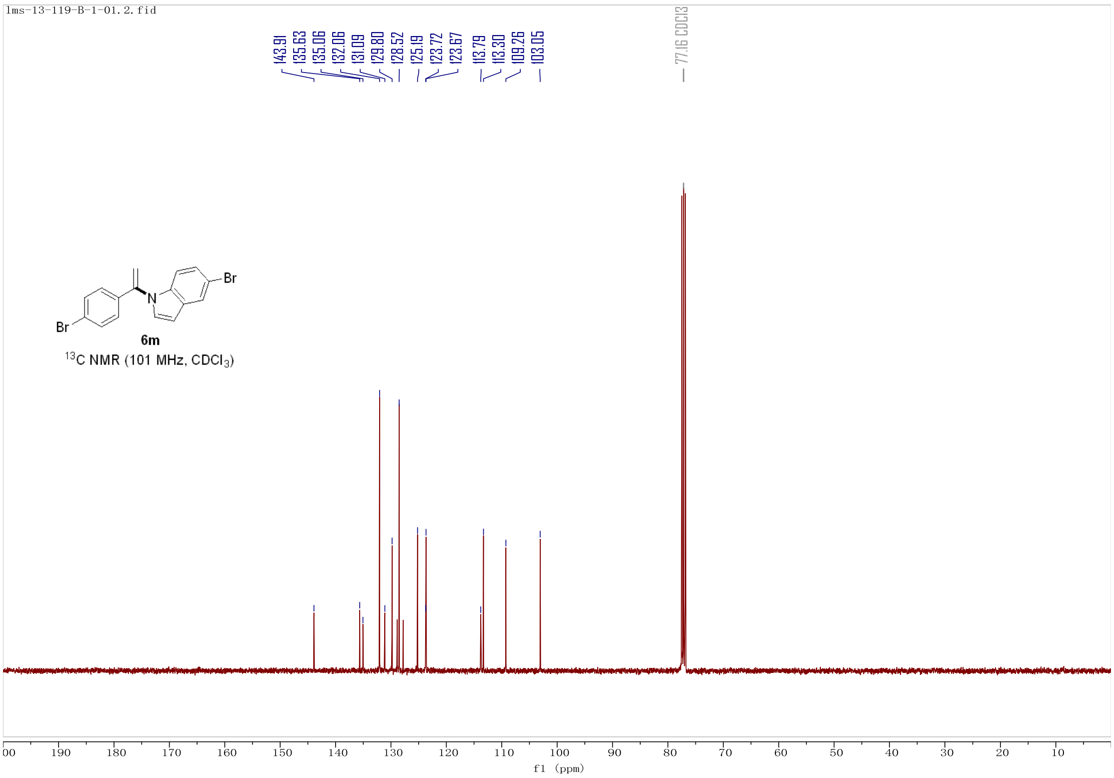
lms-13-119-B-1-01.2.fid
143.91 135.63 135.06 132.06 131.09 129.80 128.52 125.19 123.72 123.67 113.79 113.30 109.26 103.05
77.16 CDCl3
Br
Br
N
6m
13C NMR (101 MHz, CDCl3)
f1 (ppm)

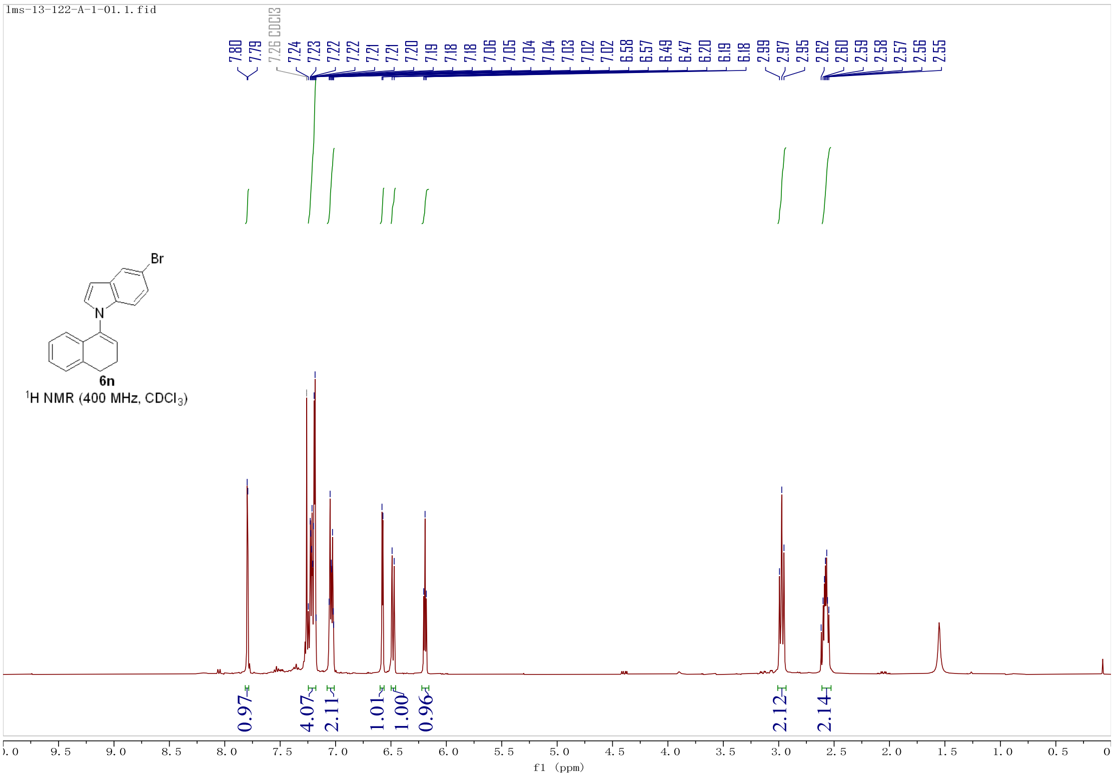
lms-13-122-A-1-01.1.fid
7.80
7.79
7.26 CDCl3
7.24
7.23
7.22
7.22
7.21
7.21
7.20
7.19
7.18
7.18
7.06
7.05
7.04
7.03
7.02
7.02
6.58
6.57
6.49
6.47
6.20
6.19
6.18
2.99
2.97
2.95
2.62
2.60
2.59
2.58
2.57
2.56
2.55
Br
N
6n
1H NMR (400 MHz, CDCl3)
0.97
4.07
2.11
1.01
1.00
0.96
2.12
2.14
f1 (ppm)

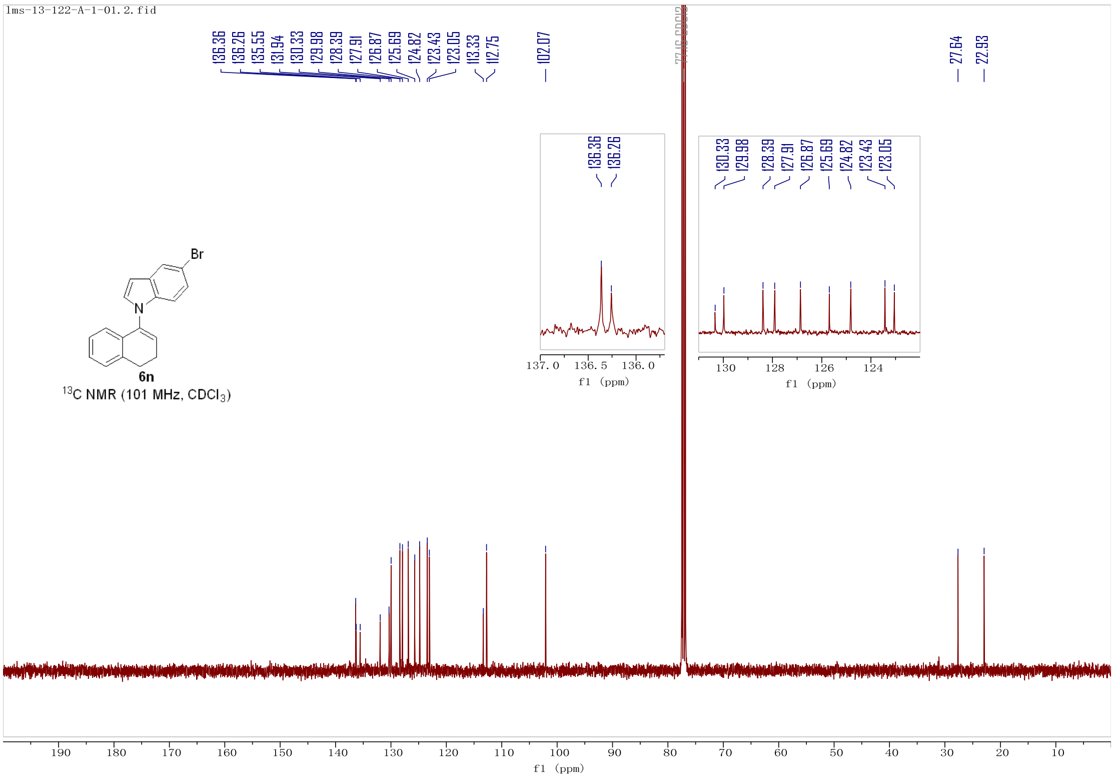
lms-13-122-A-1-01.2.fid
136.36
136.26
135.55
131.94
130.33
129.98
128.39
127.91
126.87
125.69
124.82
123.43
123.05
113.33
112.75
102.07
77.16 CDCl3
27.64
22.93
Br
N
6n
13C NMR (101 MHz, CDCl3)
f1 (ppm)

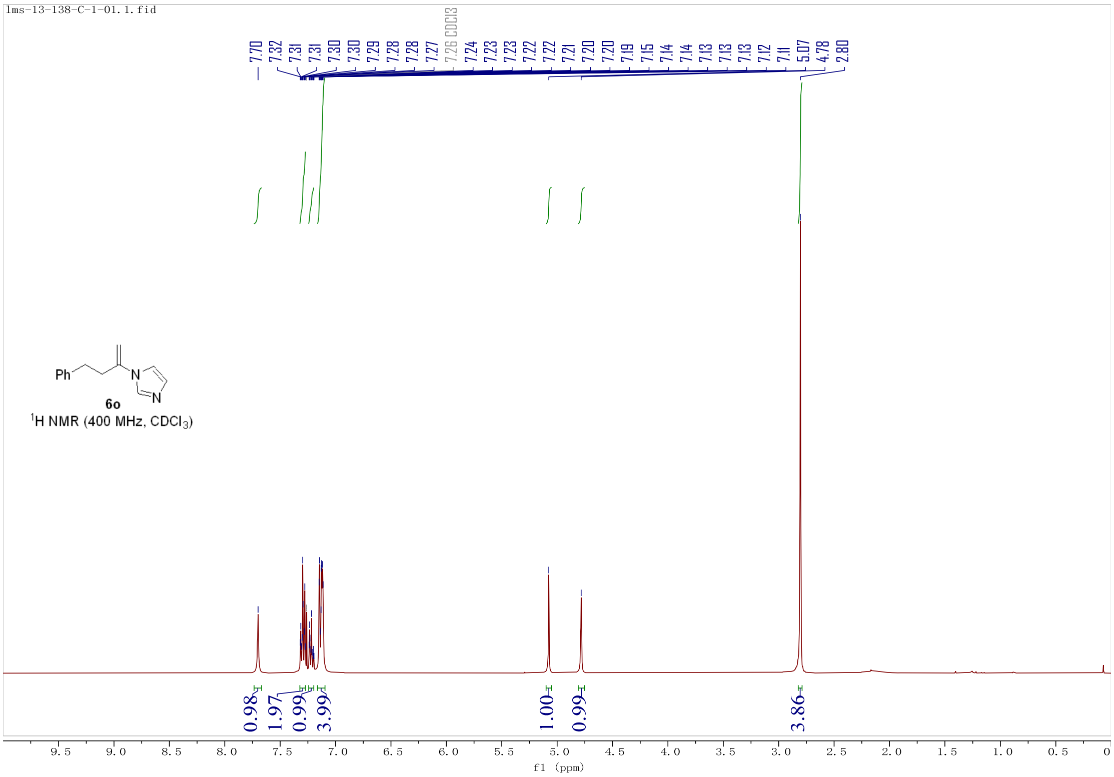
lms-13-138-C-1-01.1.fid
Ph
N
N
6o
1H NMR (400 MHz, CDCl3)
f1 (ppm)

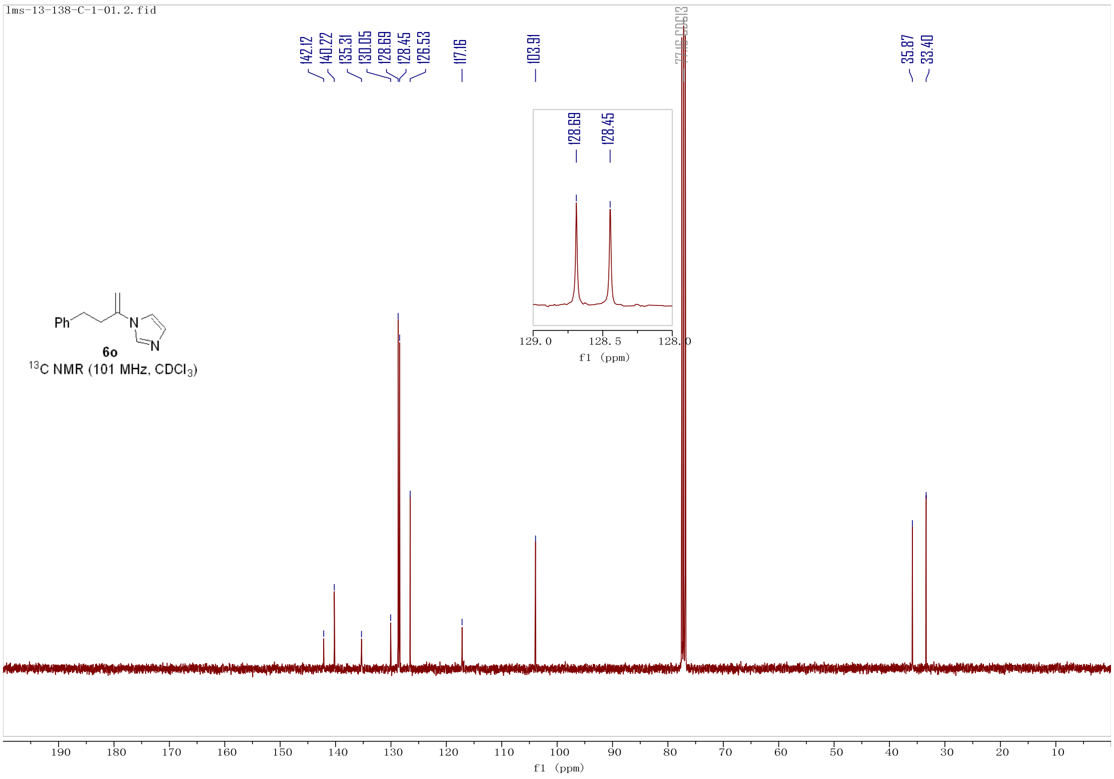
lms-13-138-C-1-01.2.fid
Ph
N
N
6o
13C NMR (101 MHz, CDCl3)
f1 (ppm)

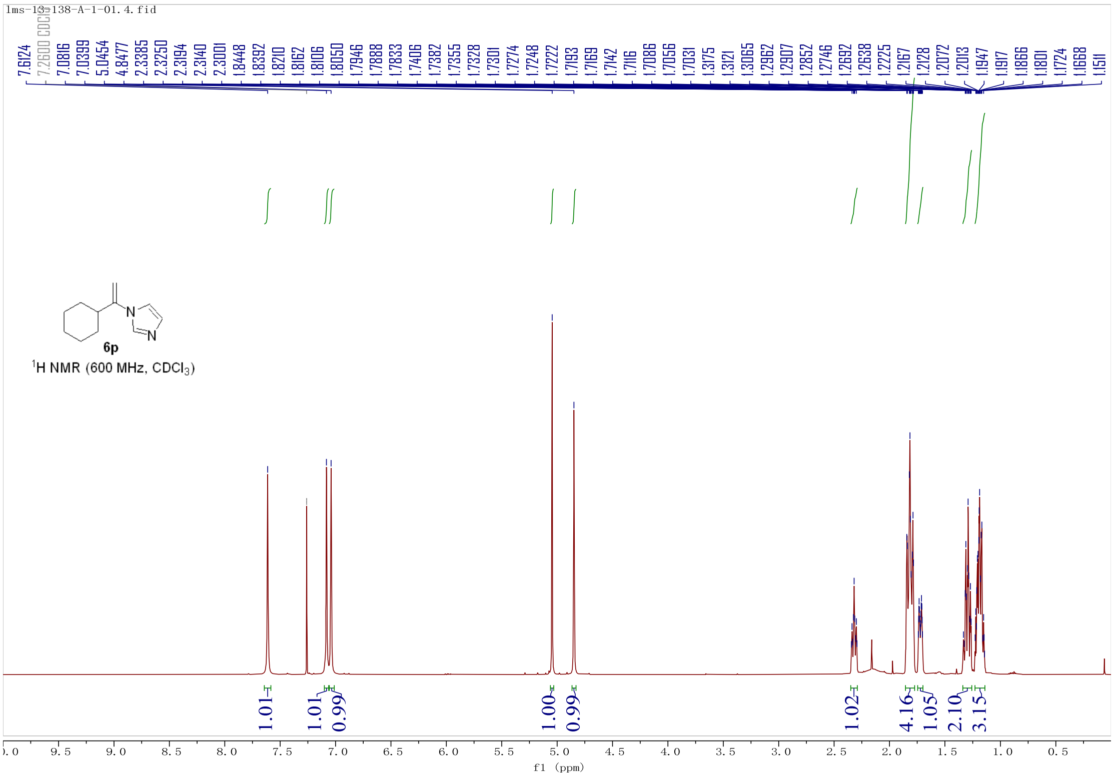
lms-13-138-A-1-01.4.fid
7.6124
7.2600 CDCl3
7.0816
7.0399
5.0454
4.8477
2.3385
2.3250
2.3194
2.3140
2.3001
1.8448
1.8392
1.8210
1.8162
1.8106
1.8050
1.7946
1.7888
1.7833
1.7406
1.7382
1.7355
1.7328
1.7301
1.7274
1.7248
1.7222
1.7193
1.7169
1.7142
1.7116
1.7086
1.7056
1.7031
1.3175
1.3121
1.3065
1.2962
1.2907
1.2852
1.2746
1.2692
1.2638
1.2225
1.2167
1.2128
1.2072
1.2013
1.1947
1.1917
1.1866
1.1801
1.1724
1.1668
1.1511
N
N
6p
^{1}H NMR (600 MHz, CDCl$_3$)
1.01
1.01
0.99
1.00
0.99
1.02
4.16
1.05
2.10
3.15
f1 (ppm)

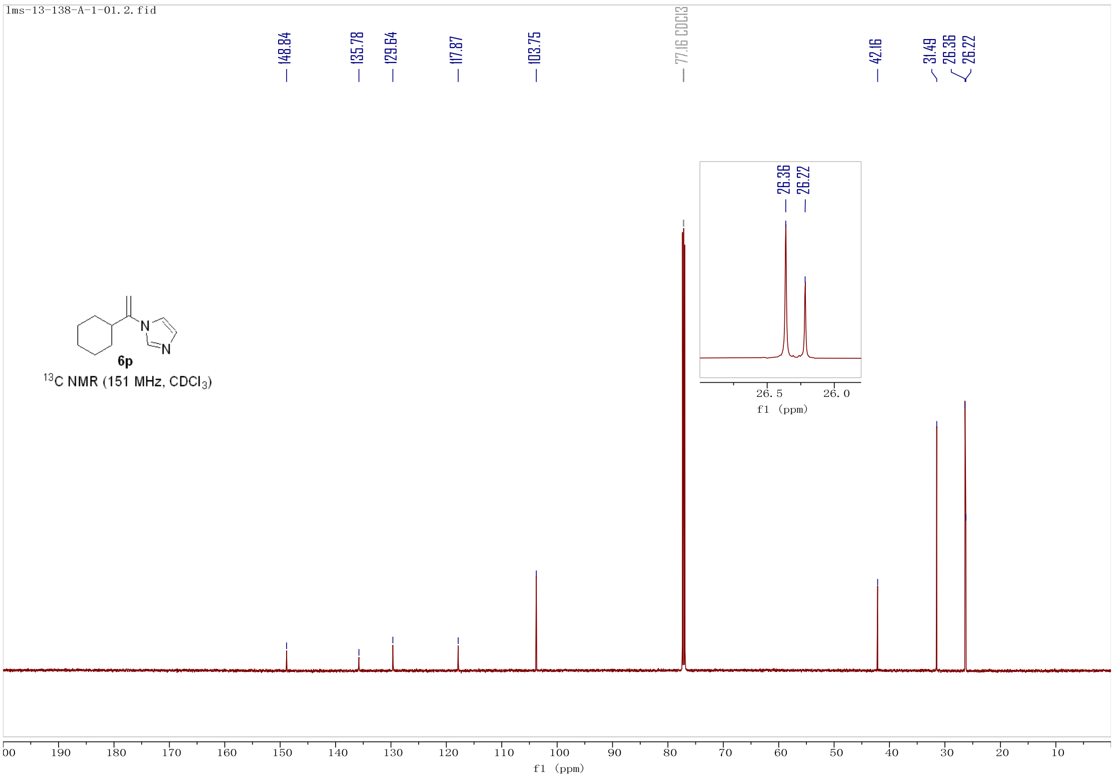
lms-13-138-A-1-01.2.fid
148.84
135.78
129.64
117.87
103.75
77.16 CDCl3
42.16
31.49
26.36
26.22
N
N
6p
^{13}C NMR (151 MHz, CDCl$_3$)
f1 (ppm)

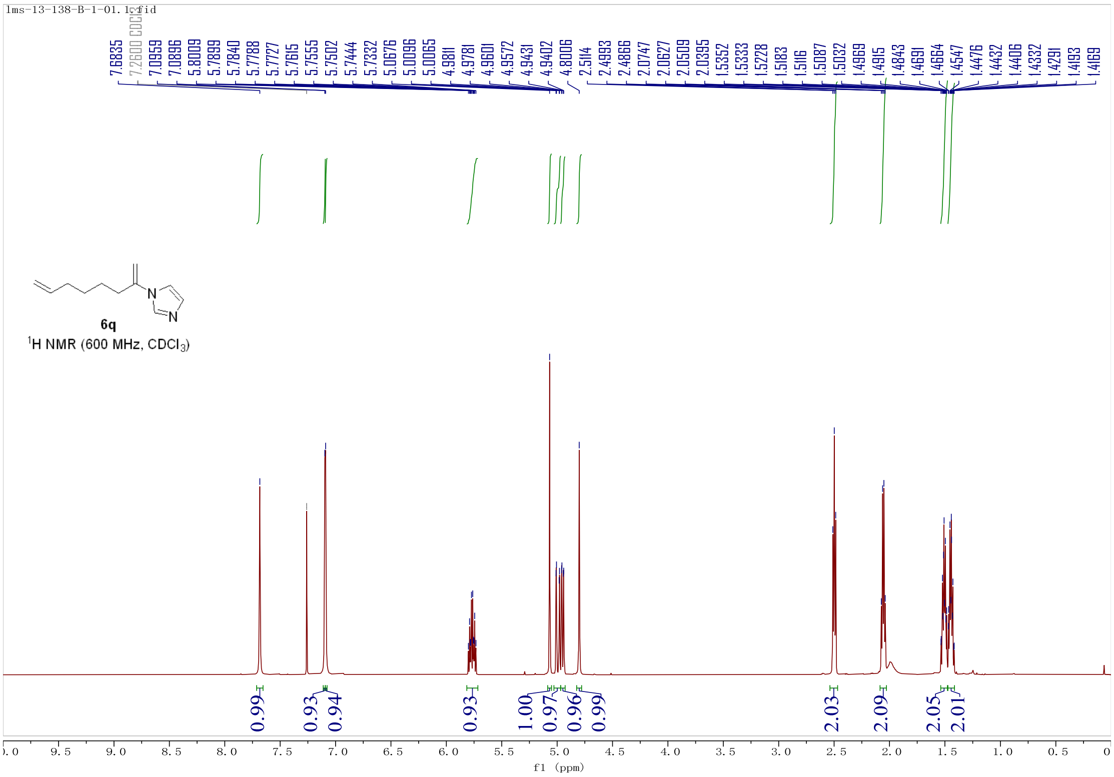
lms-13-138-B-1-01.1.fid
7.6835
7.2600 CDCl3
7.0959
7.0896
5.8009
5.7899
5.7840
5.7788
5.7727
5.7615
5.7555
5.7502
5.7444
5.7332
5.0676
5.0096
5.0065
4.9811
4.9781
4.9601
4.9572
4.9431
4.9402
4.8006
2.5114
2.4993
2.4866
2.0747
2.0627
2.0509
2.0395
1.5352
1.5333
1.5228
1.5183
1.5116
1.5087
1.5032
1.4969
1.4915
1.4843
1.4691
1.4664
1.4547
1.4476
1.4432
1.4406
1.4332
1.4291
1.4193
1.4169
N
N
6q
1H NMR (600 MHz, CDCl3)
0.99
0.93
0.94
0.93
1.00
0.97
0.96
0.99
2.03
2.09
2.05
2.01
f1 (ppm)

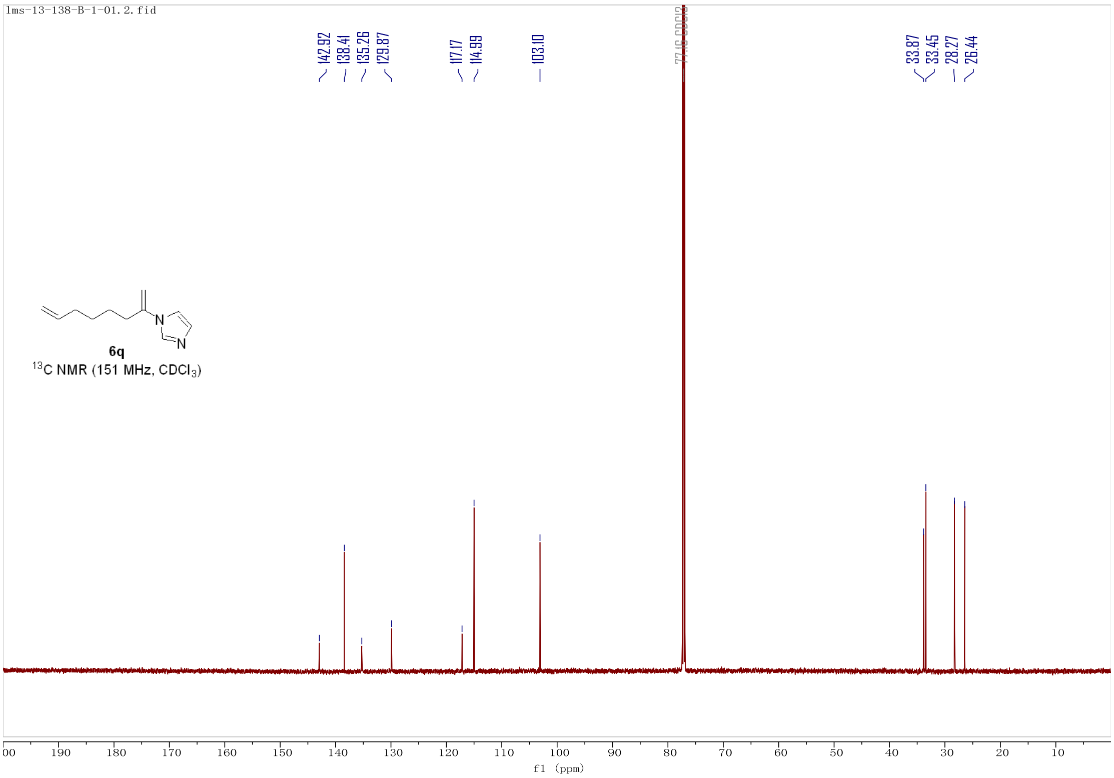
lms-13-138-B-1-01.2.fid
142.92
138.41
135.26
129.87
117.17
114.99
103.10
77.16 CDCl3
33.87
33.45
28.27
26.44
N
N
6q
13C NMR (151 MHz, CDCl3)
f1 (ppm)

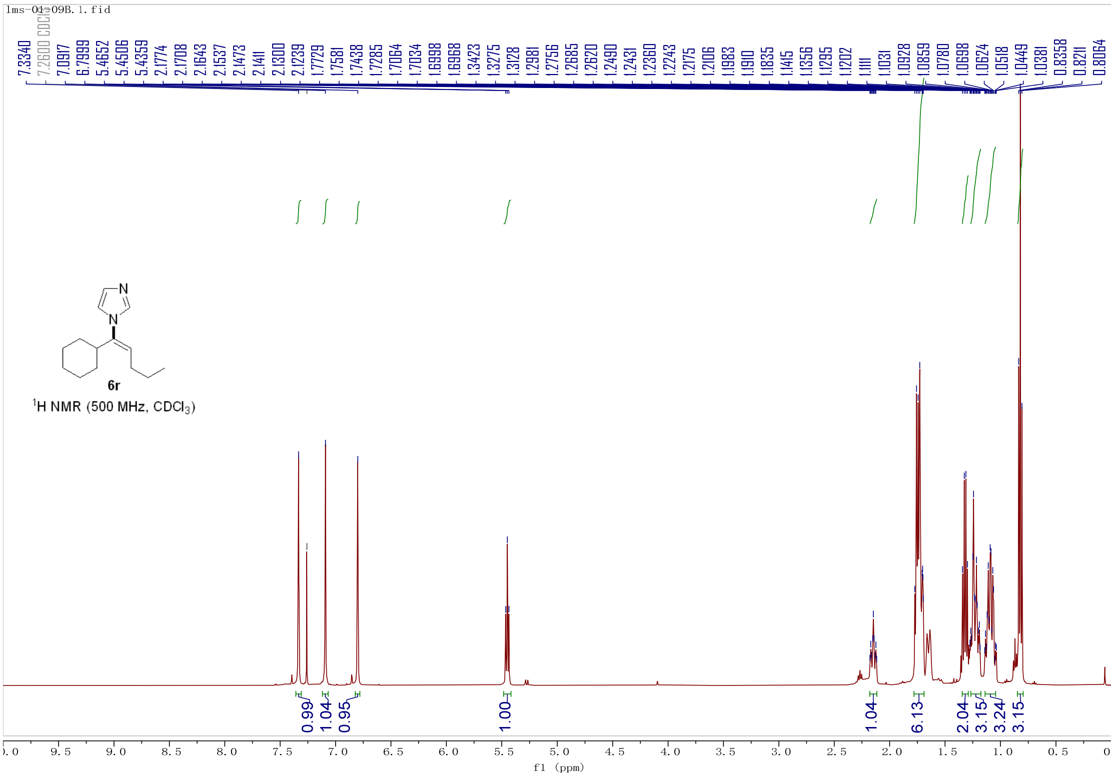
lms-01-09B.1.fid
7.3340 7.2600 CDCl3 7.0917 6.7999 5.4652 5.4506 5.4359 2.1774 2.1708 2.1643 2.1537 2.1473 2.1411 2.1300 2.1239 1.7729 1.7581 1.7438 1.7285 1.7064 1.7034 1.6998 1.6968 1.3423 1.3275 1.3128 1.2981 1.2756 1.2685 1.2620 1.2490 1.2431 1.2360 1.2243 1.2175 1.2106 1.1983 1.1910 1.1835 1.1415 1.1356 1.1295 1.1202 1.1111 1.1031 1.0928 1.0859 1.0780 1.0698 1.0624 1.0518 1.0449 1.0381 0.8358 0.8211 0.8064
6r
^{1}H NMR (500 MHz, CDCl$_3$)
0.99 1.04 0.95 1.00 1.04 6.13 2.04 3.15 3.24 3.15
f1 (ppm)

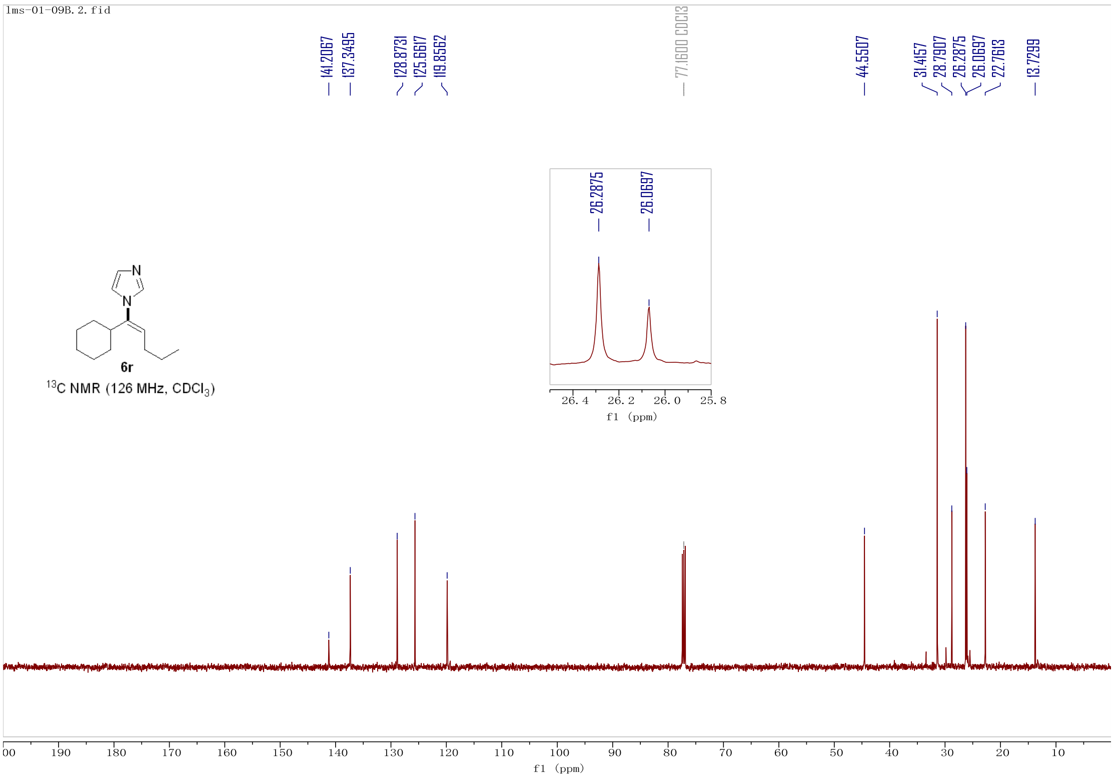
lms-01-09B.2.fid
141.2067 137.3495 128.8731 125.6617 119.8562 77.1600 CDCl3 44.5507 31.4157 28.7907 26.2875 26.0697 22.7613 13.7299
26.2875 26.0697
f1 (ppm)
6r
^{13}C NMR (126 MHz, CDCl$_3$)
f1 (ppm)

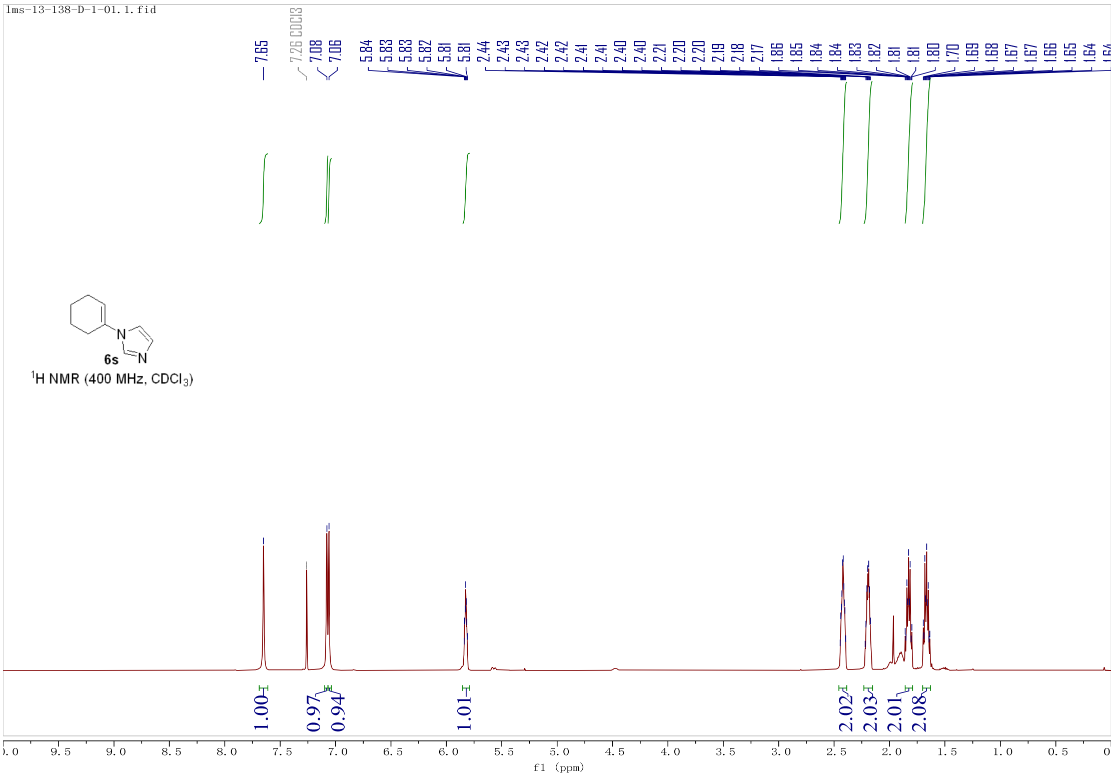
lms-13-138-D-1-01.1.fid
7.65
7.26 CDCl3
7.08
7.06
6s
^{1}H NMR (400 MHz, $CDCl_3$)
1.00
0.97
0.94
1.01
2.02
2.03
2.01
2.08
f1 (ppm)

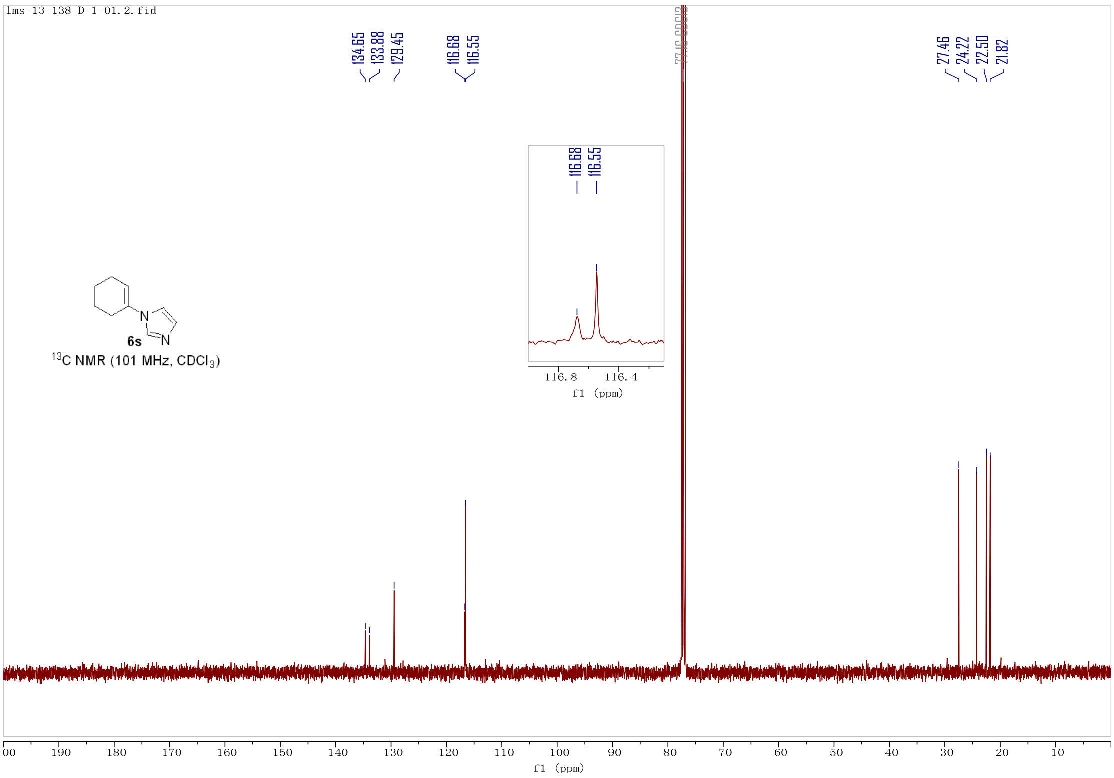
lms-13-138-D-1-01.2.fid
134.65
133.88
129.45
116.68
116.55
27.46
24.22
22.50
21.82
6s
^{13}C NMR (101 MHz, $CDCl_3$)
f1 (ppm)

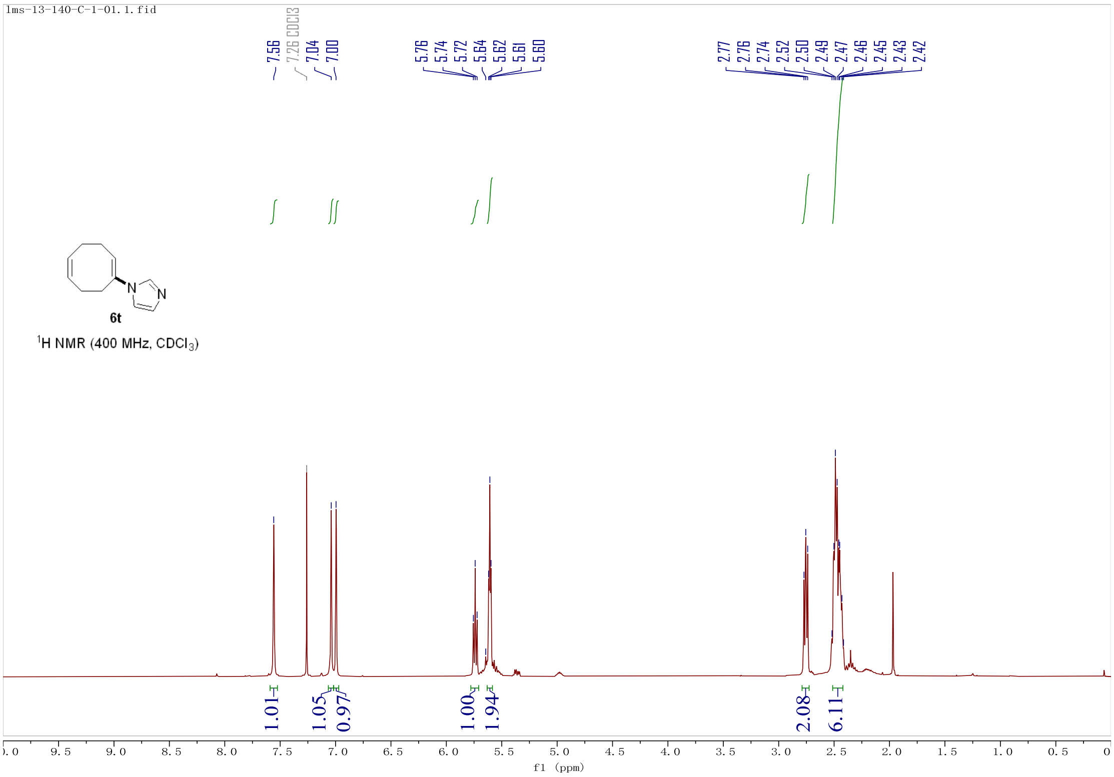
lms-13-140-C-1-01.1.fid
7.56
7.26 CDCl3
7.04
7.00
5.76
5.74
5.72
5.64
5.62
5.61
5.60
2.77
2.76
2.74
2.52
2.50
2.49
2.47
2.46
2.45
2.43
2.42
N
N
6t
1H NMR (400 MHz, CDCl3)
1.01
1.05
0.97
1.00
1.94
2.08
6.11
f1 (ppm)

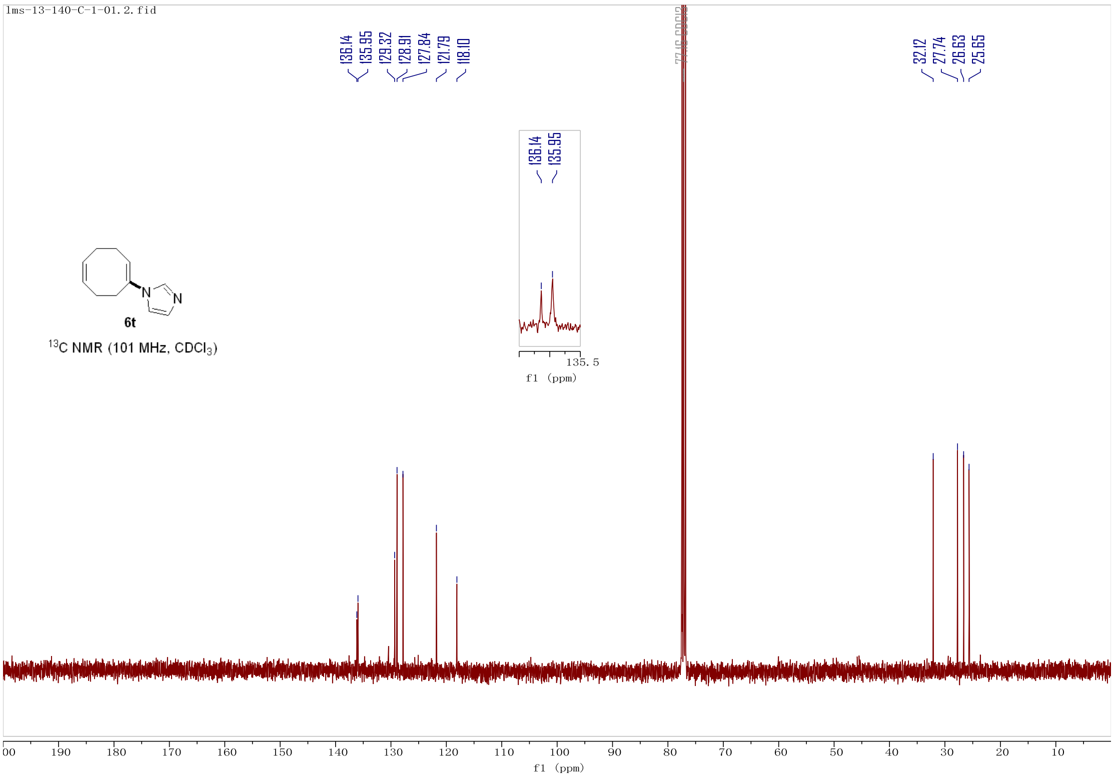
lms-13-140-C-1-01.2.fid
136.14
135.95
129.32
128.91
127.84
121.79
118.10
32.12
27.74
26.63
25.65
136.14
135.95
135.5
f1 (ppm)
N
N
6t
13C NMR (101 MHz, CDCl3)
f1 (ppm)

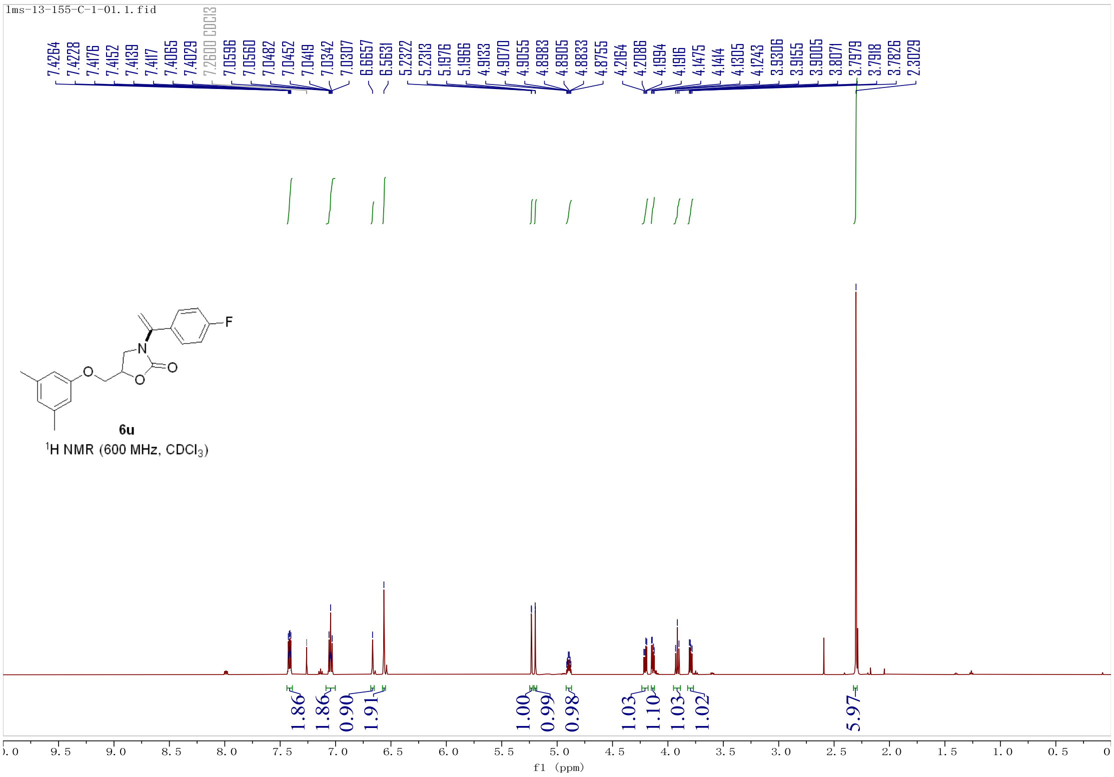
lms-13-155-C-1-01.1.fid
7.4264 7.4228 7.4176 7.4152 7.4139 7.4117 7.4065 7.4029 7.2600 CDCl3 7.0596 7.0560 7.0482 7.0452 7.0419 7.0342 7.0307 6.6657 6.5631 5.2322 5.2313 5.1976 5.1966 4.9133 4.9070 4.9055 4.8983 4.8905 4.8833 4.8755 4.2164 4.2086 4.1994 4.1916 4.1475 4.1414 4.1305 4.1243 3.9306 3.9155 3.9005 3.8071 3.7979 3.7918 3.7826 2.3029
F
N
O
O
O
6u
^{1}H NMR (600 MHz, $CDCl_3$)
1.86 1.86 0.90 1.91 1.00 0.99 0.98 1.03 1.10 1.03 1.02 5.97
f1 (ppm)

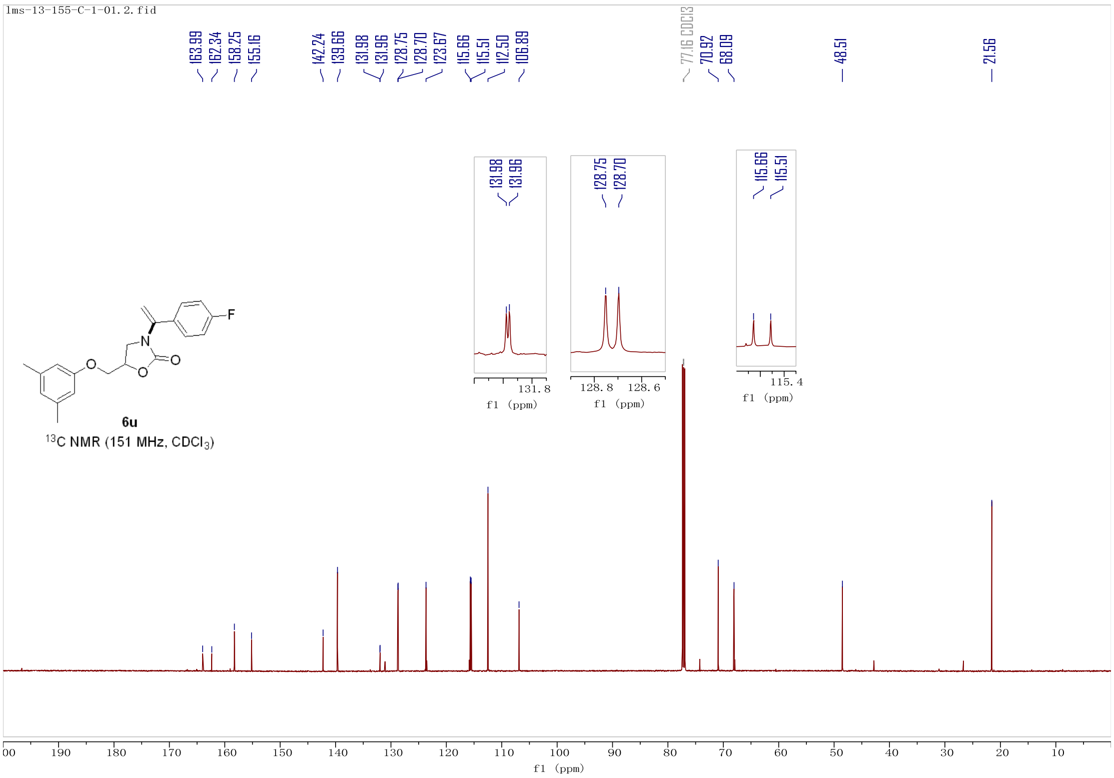
lms-13-155-C-1-01.2.fid
163.99 162.34 158.25 155.16 142.24 139.66 131.98 131.96 128.75 128.70 123.67 115.66 115.51 112.50 106.89 77.16 CDCl3 70.92 68.09 48.51 21.56
131.98 131.96
128.75 128.70
115.66 115.51
F
N
O
O
O
6u
^{13}C NMR (151 MHz, $CDCl_3$)
f1 (ppm)

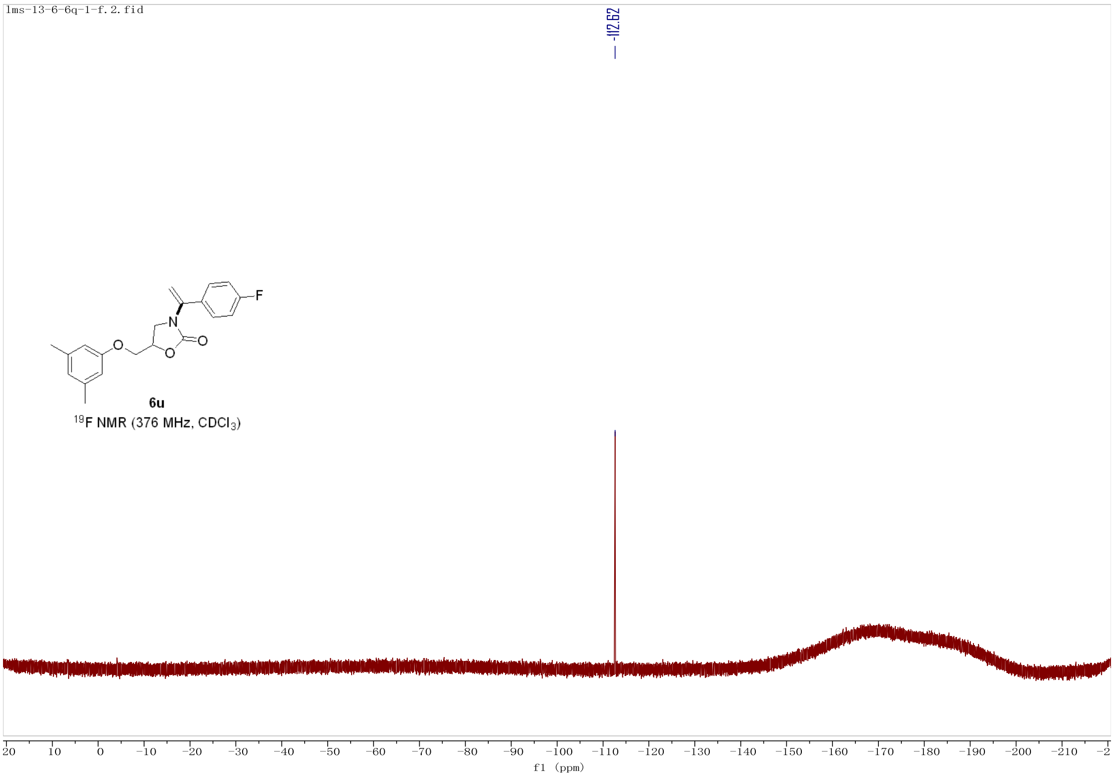
lms-13-6-6q-1-f.2.fid
-112.62
F
O
N
O
O
6u
19F NMR (376 MHz, CDCl3)
20 10 0 −10 −20 −30 −40 −50 −60 −70 −80 −90 −100 −110 −120 −130 −140 −150 −160 −170 −180 −190 −200 −210
f1 (ppm)

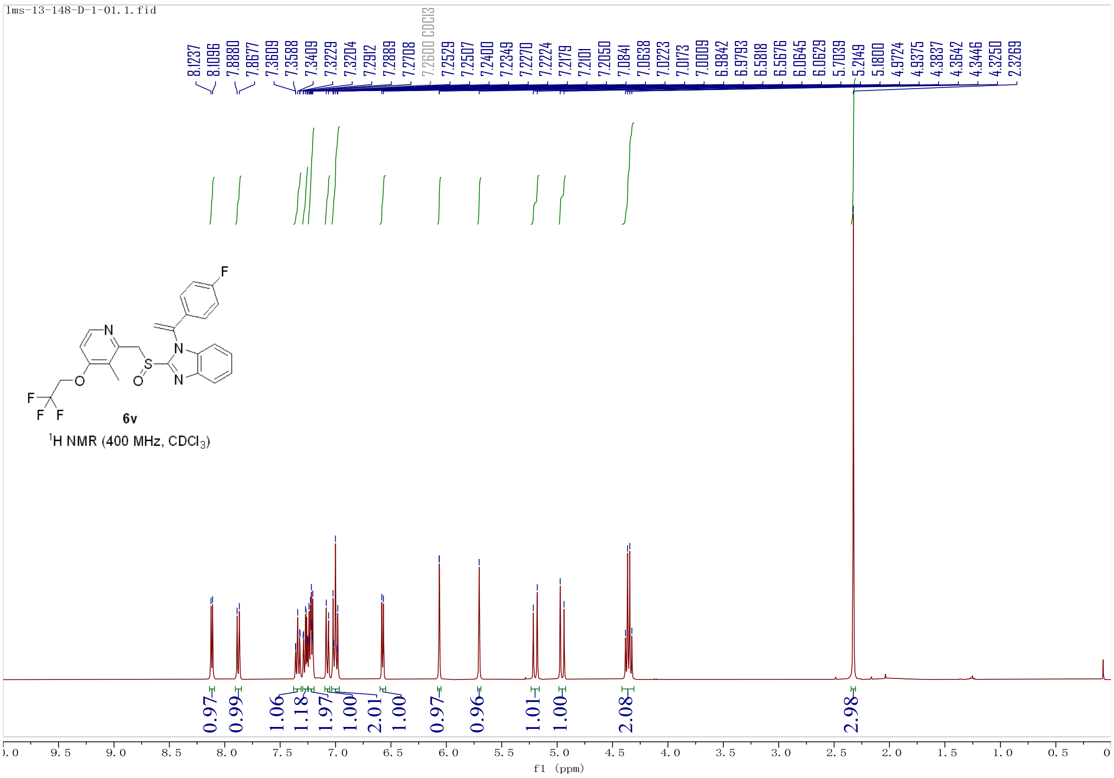
lms-13-148-D-1-01.1.fid
8.1237
8.1096
7.8880
7.8677
7.3609
7.3588
7.3409
7.3229
7.3204
7.2912
7.2889
7.2708
7.2600 CDCl3
7.2529
7.2507
7.2400
7.2349
7.2270
7.2224
7.2179
7.2101
7.2050
7.0841
7.0638
7.0223
7.0173
7.0009
6.9842
6.9793
6.5818
6.5676
6.0645
6.0629
5.7039
5.2149
5.1800
4.9724
4.9375
4.3837
4.3642
4.3446
4.3250
2.3269
F
N
N
S
O
N
O
F
F F
6v
1H NMR (400 MHz, CDCl3)
0.97
0.99
1.06
1.18
1.97
1.00
2.01
1.00
0.97
0.96
1.01
1.00
2.08
2.98
9.5 9.0 8.5 8.0 7.5 7.0 6.5 6.0 5.5 5.0 4.5 4.0 3.5 3.0 2.5 2.0 1.5 1.0 0.5 0
f1 (ppm)

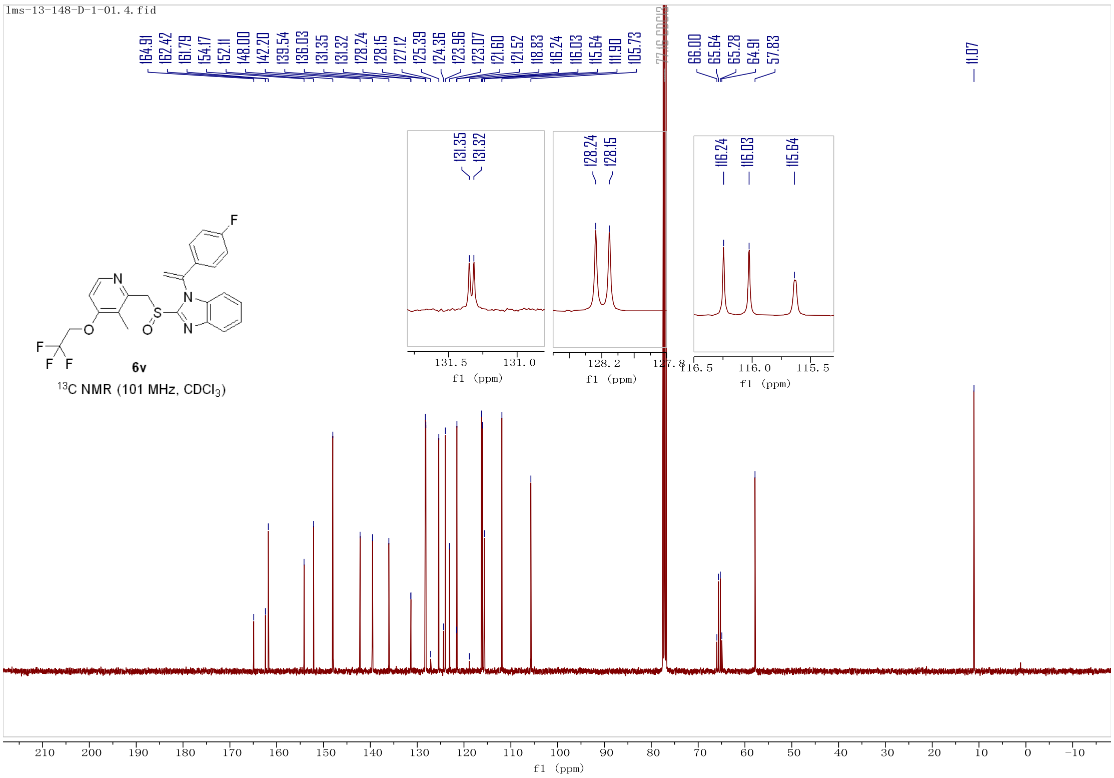
lms-13-148-D-1-01.4.fid
6v
13C NMR (101 MHz, CDCl3)
f1 (ppm)

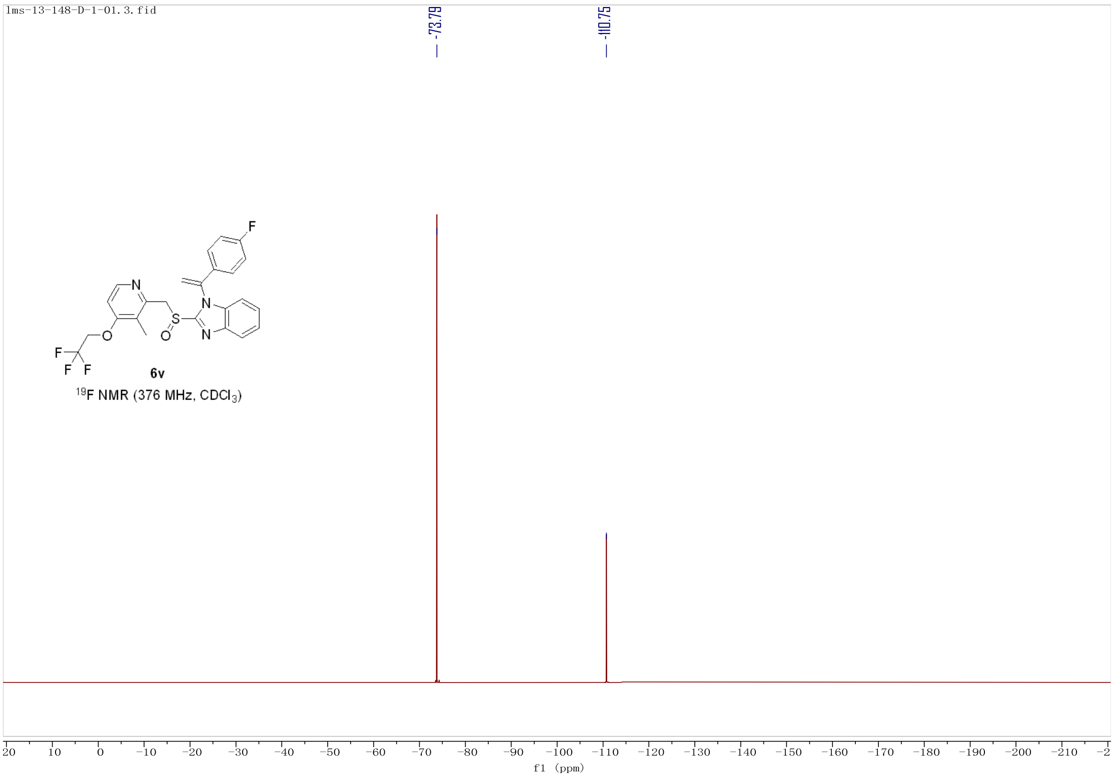
lms-13-148-D-1-01.3.fid
-73.79
-110.75
6v
19F NMR (376 MHz, CDCl3)
f1 (ppm)

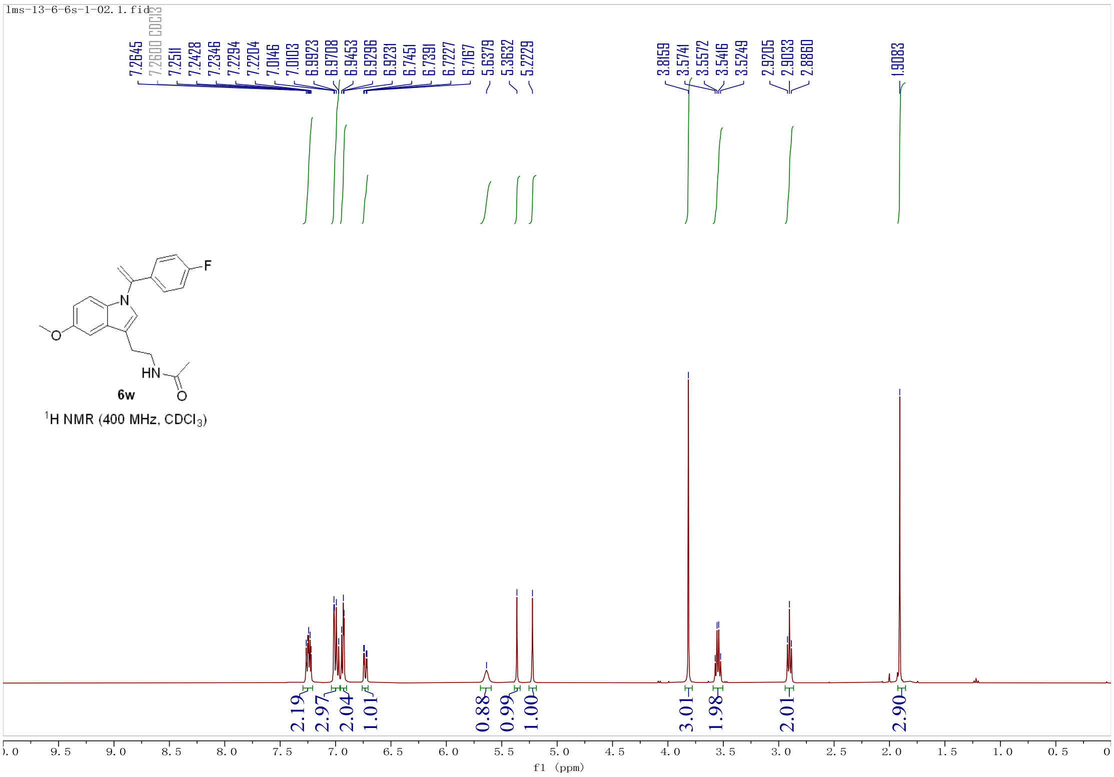
lms-13-6-6s-1-02.1.fid
7.2645
7.2600 CDCl3
7.2511
7.2428
7.2346
7.2294
7.2204
7.0146
7.0103
6.9923
6.9708
6.9453
6.9296
6.9231
6.7451
6.7391
6.7227
6.7167
5.6379
5.3632
5.2229
3.8159
3.5741
3.5572
3.5416
3.5249
2.9205
2.9033
2.8860
1.9083
6w
1H NMR (400 MHz, CDCl3)
2.19
2.97
2.04
1.01
0.88
0.99
1.00
3.01
1.98
2.01
2.90
f1 (ppm)

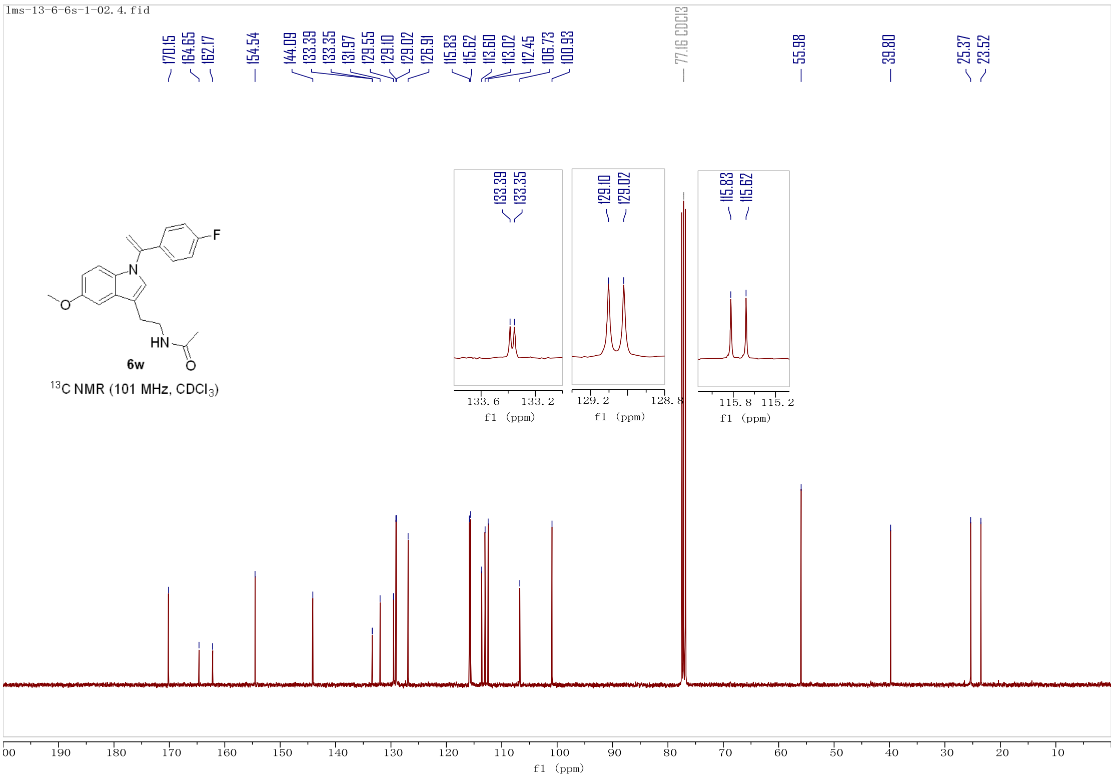
lms-13-6-6s-1-02.4.fid
170.15
164.65
162.17
154.54
144.09
133.39
133.35
131.97
129.55
129.10
129.02
126.91
115.83
115.62
113.60
113.02
112.45
106.73
100.93
77.16 CDCl3
55.98
39.80
25.37
23.52
6w
13C NMR (101 MHz, CDCl3)
f1 (ppm)

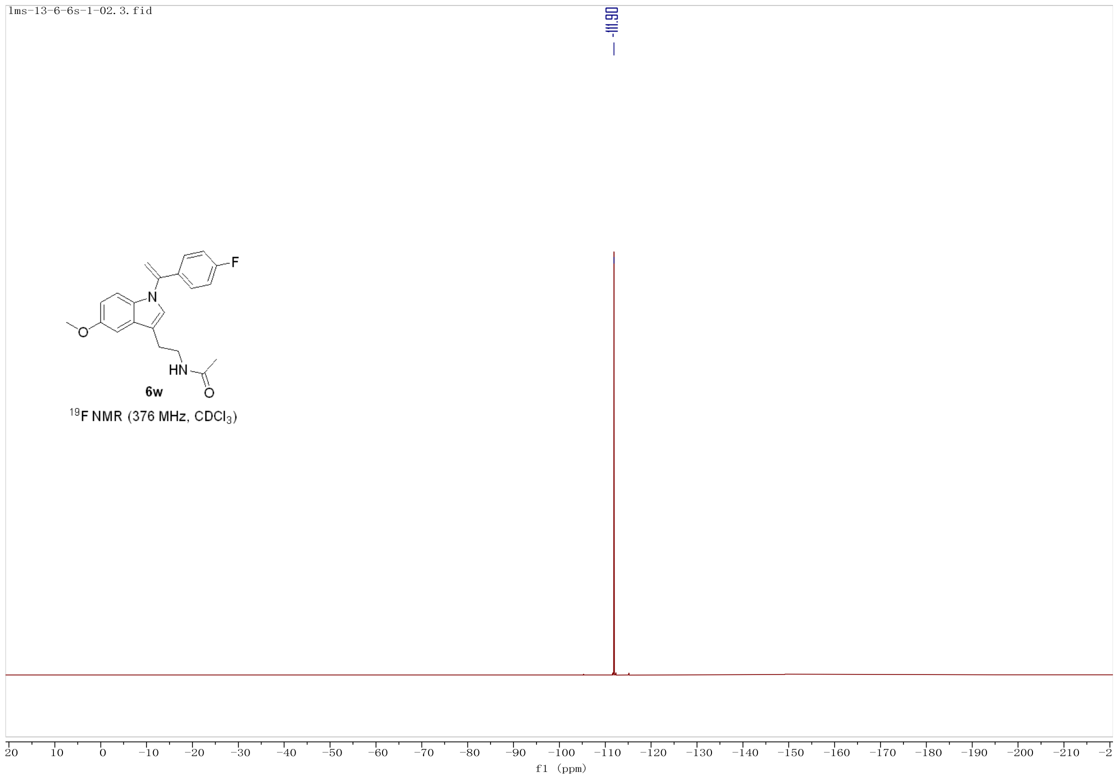
lms-13-6-6s-1-02.3.fid
-111.90
F
N
O
HN
O
6w
^{19}F NMR (376 MHz, $CDCl_3$)
f1 (ppm)

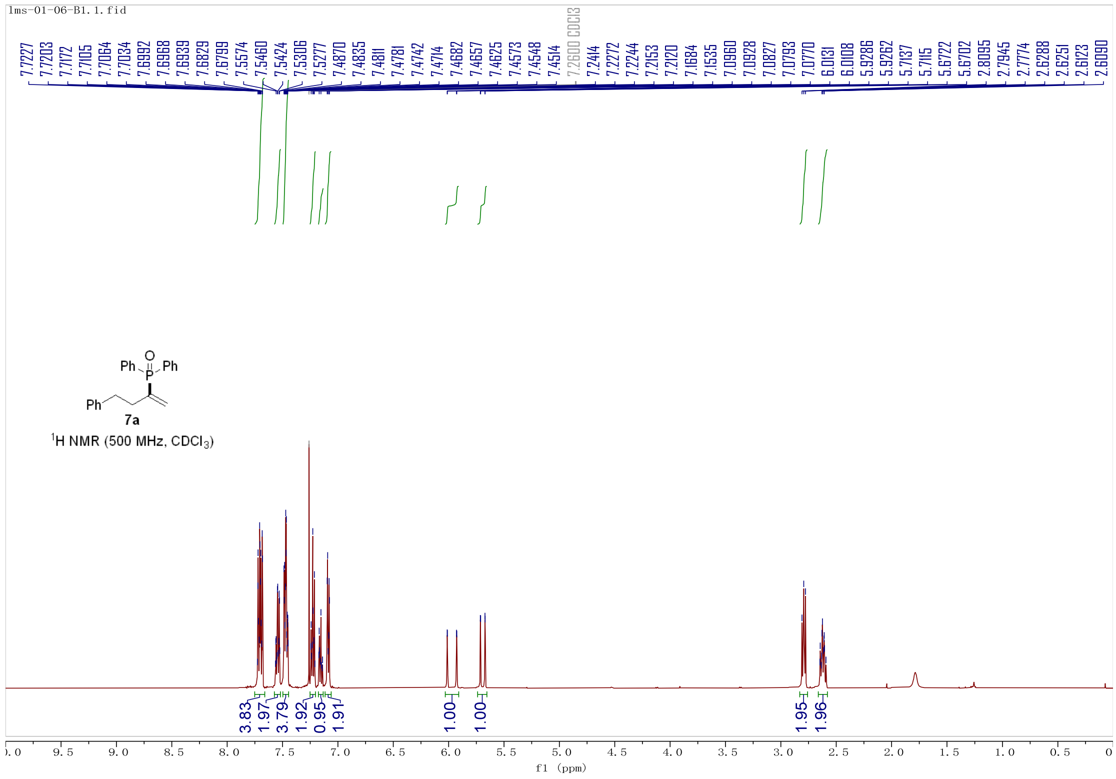
lms-01-06-B1.1.fid
7.7227 7.7203 7.7172 7.7105 7.7064 7.7034 7.6992 7.6968 7.6939 7.6829 7.6799 7.5574 7.5460 7.5424 7.5306 7.5277 7.4870 7.4835 7.4811 7.4781 7.4742 7.4714 7.4682 7.4657 7.4625 7.4573 7.4548 7.4514 7.2600 CDCl3 7.2414 7.2272 7.2244 7.2153 7.2120 7.1684 7.1535 7.0960 7.0928 7.0827 7.0793 7.0770 6.0131 6.0108 5.9286 5.9262 5.7137 5.7115 5.6722 5.6702 2.8095 2.7945 2.7774 2.6288 2.6251 2.6123 2.6090
Ph
O
P
Ph
Ph
7a
^{1}H NMR (500 MHz, $CDCl_3$)
3.83 1.97 3.79 1.92 0.95 1.91 1.00 1.00 1.95 1.96
f1 (ppm)

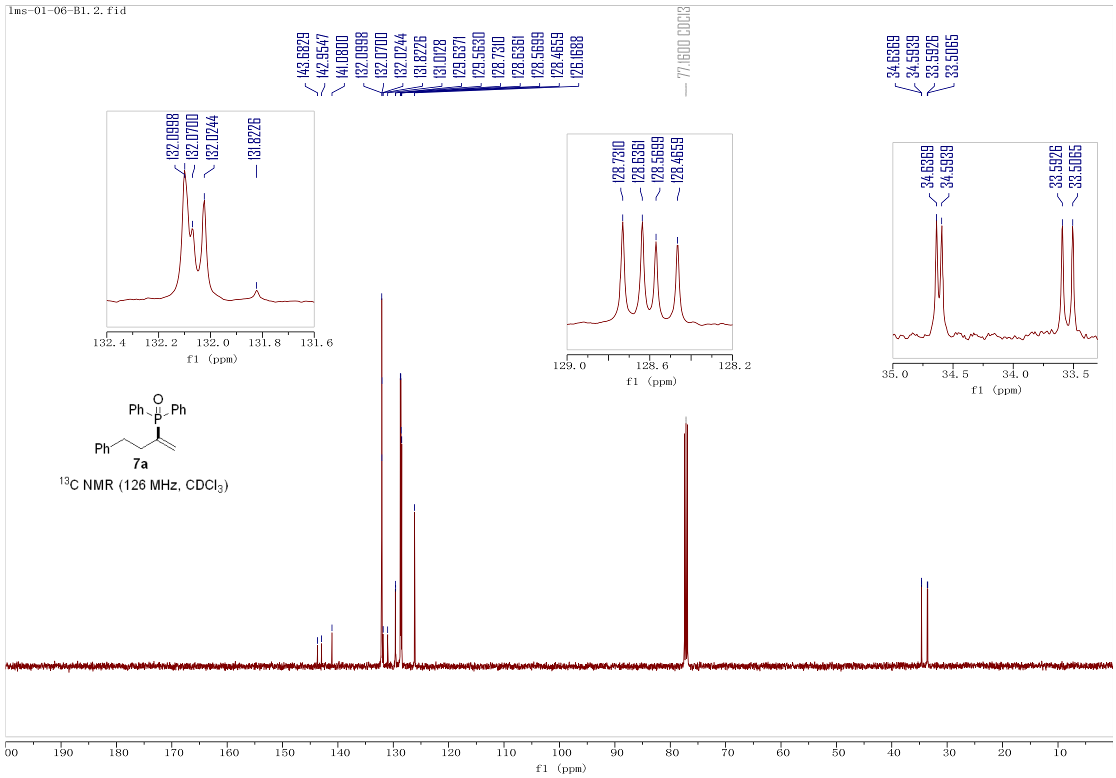
lms-01-06-B1.2.fid
143.6829
142.9547
141.0800
132.0998
132.0700
132.0244
131.8226
131.0128
129.6371
129.5630
128.7310
128.6361
128.5699
128.4659
126.1688
77.1600 CDCl3
34.6369
34.5939
33.5926
33.5065
Ph
O
P
Ph
Ph
7a
^{13}C NMR (126 MHz, CDCl$_3$)
f1 (ppm)

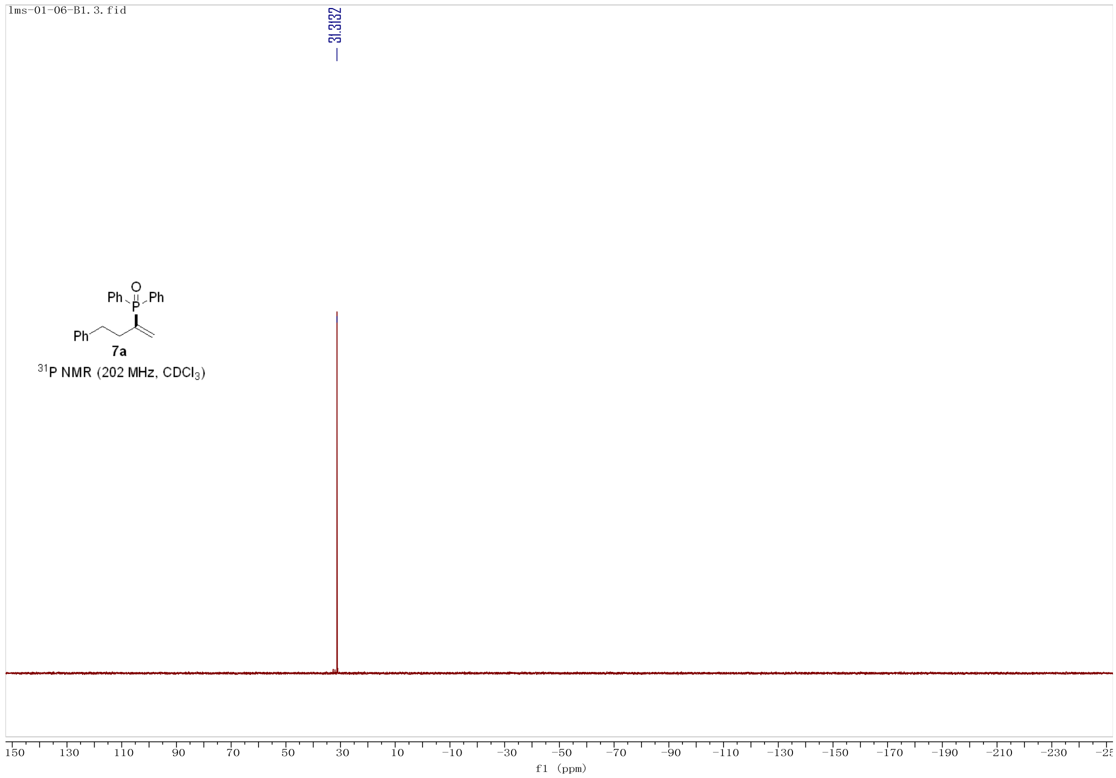
lms-01-06-B1.3.fid
31.3132
Ph
O
P
Ph
Ph
7a
^{31}P NMR (202 MHz, CDCl$_3$)
f1 (ppm)

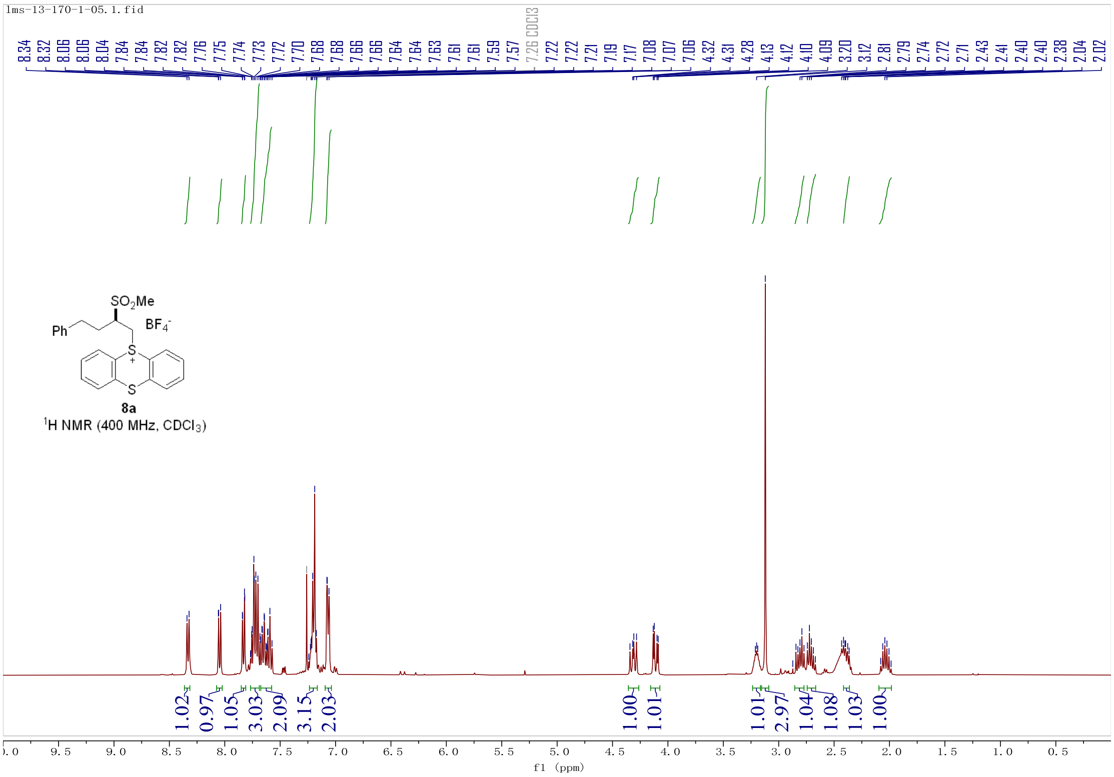
lms-13-170-1-05.1.fid
8.34 8.32 8.06 8.06 8.04 7.84 7.84 7.82 7.82 7.76 7.75 7.74 7.73 7.72 7.70 7.68 7.68 7.66 7.66 7.64 7.64 7.63 7.61 7.61 7.59 7.57 7.26 CDCl3 7.22 7.22 7.22 7.21 7.19 7.17 7.08 7.07 7.06 4.32 4.31 4.28 4.13 4.12 4.10 4.09 3.20 3.12 2.81 2.79 2.74 2.72 2.71 2.43 2.41 2.40 2.40 2.38 2.04 2.02
SO2Me
Ph
BF4-
S
S
8a
1H NMR (400 MHz, CDCl3)
1.02 0.97 1.05 3.03 2.09 3.15 2.03 1.00 1.01 1.01 2.97 1.04 1.08 1.03 1.00
f1 (ppm)

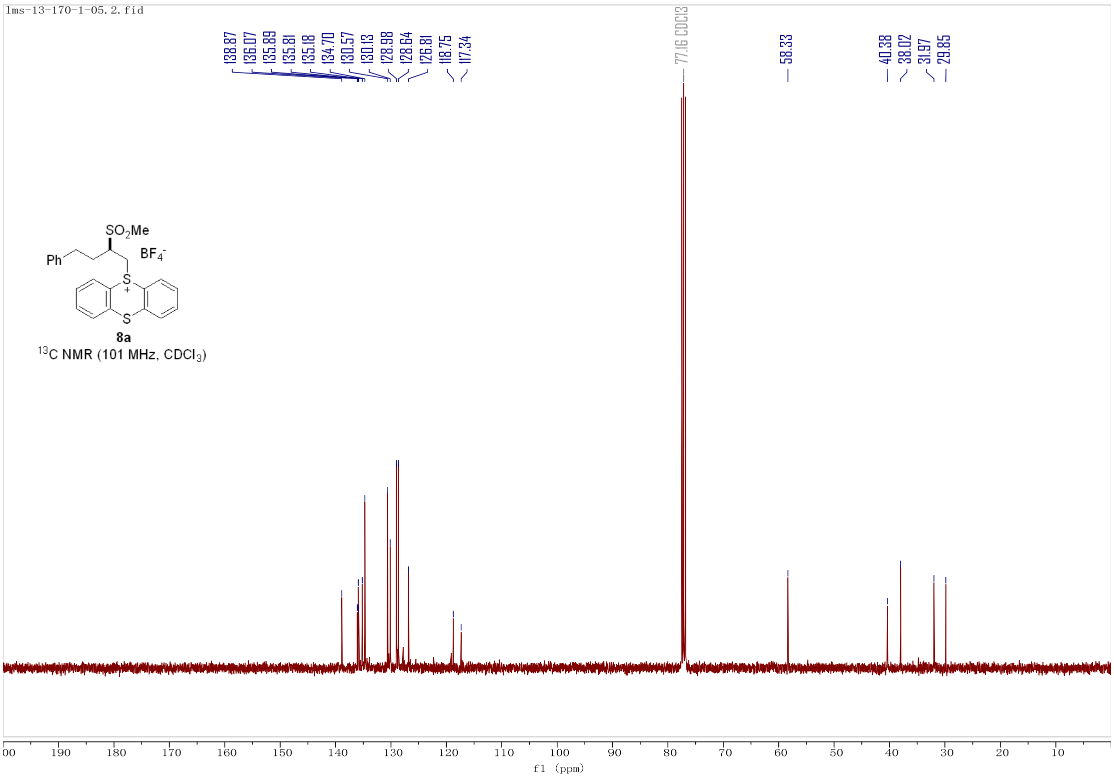
lms-13-170-1-05.2.fid
138.87 136.07 135.89 135.81 135.18 134.70 130.57 130.13 128.98 128.64 126.81 118.75 117.34
77.16 CDCl3
58.33
40.38 38.02 31.97 29.85
SO2Me
Ph
BF4-
S
S
8a
13C NMR (101 MHz, CDCl3)
f1 (ppm)

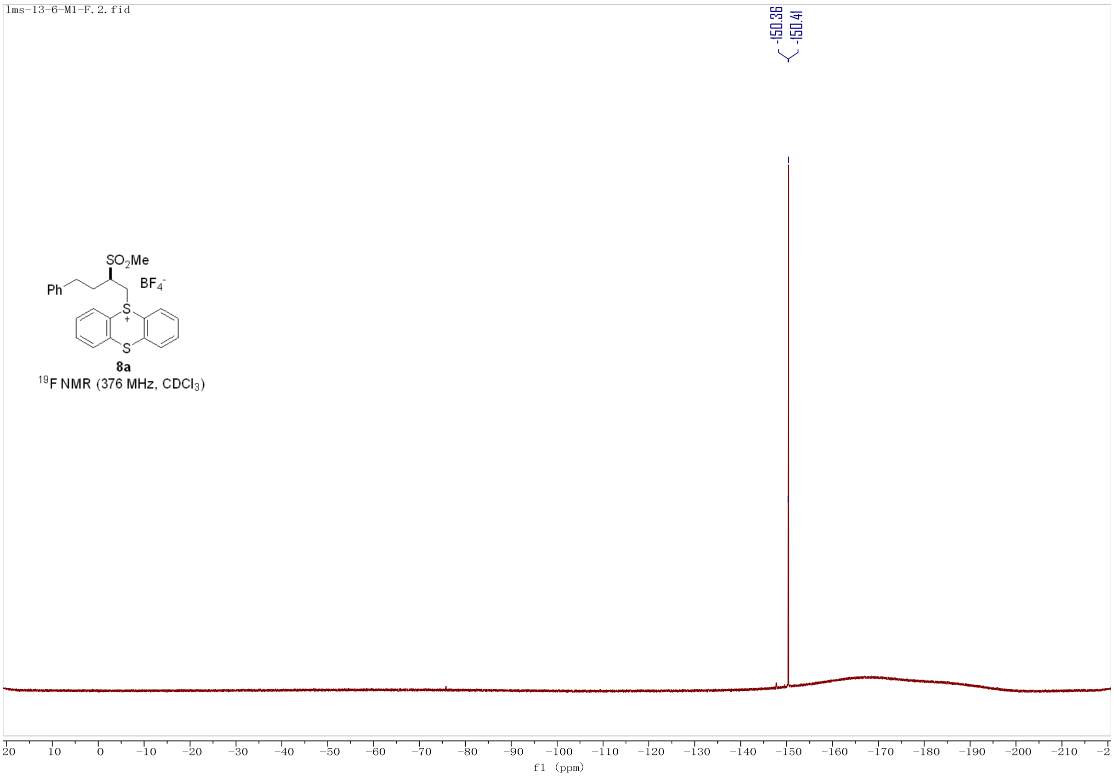
lms-13-6-M1-F.2.fid
-150.36
-150.41
SO2Me
Ph
BF4-
S
+
S
8a
19F NMR (376 MHz, CDCl3)
f1 (ppm)

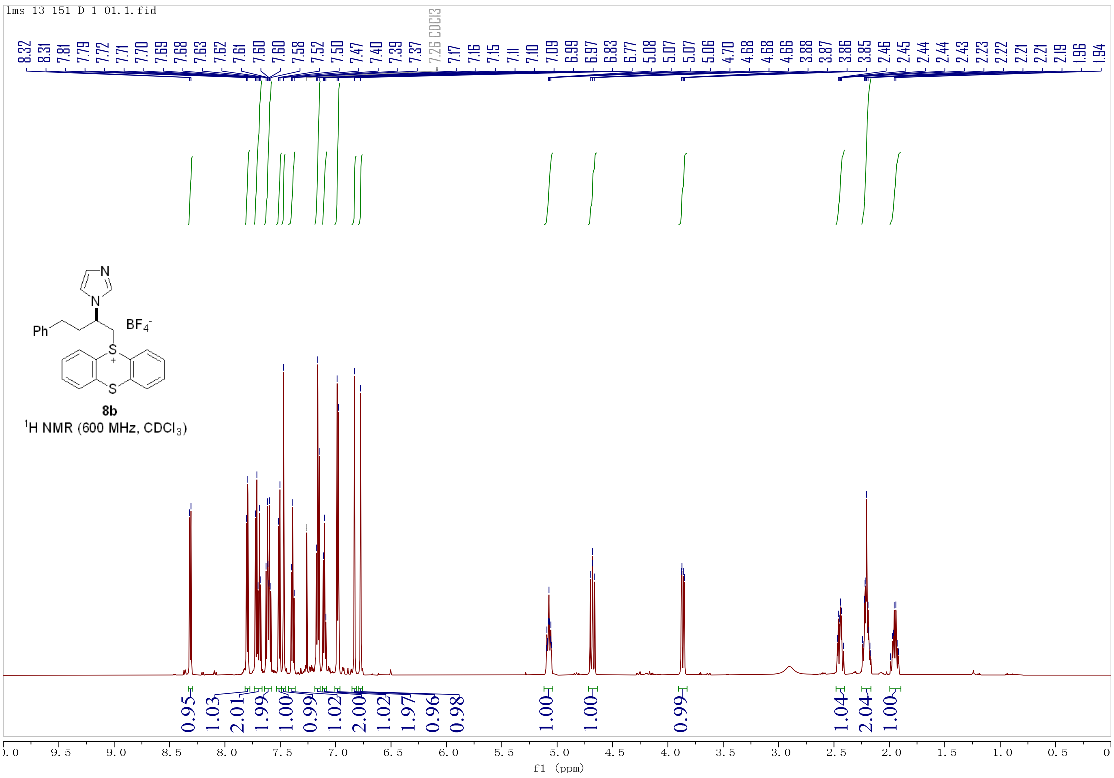
lms-13-151-D-1-01.1.fid
7.26 CDCl3
N
N
Ph
BF4-
S
+
S
8b
1H NMR (600 MHz, CDCl3)
f1 (ppm)

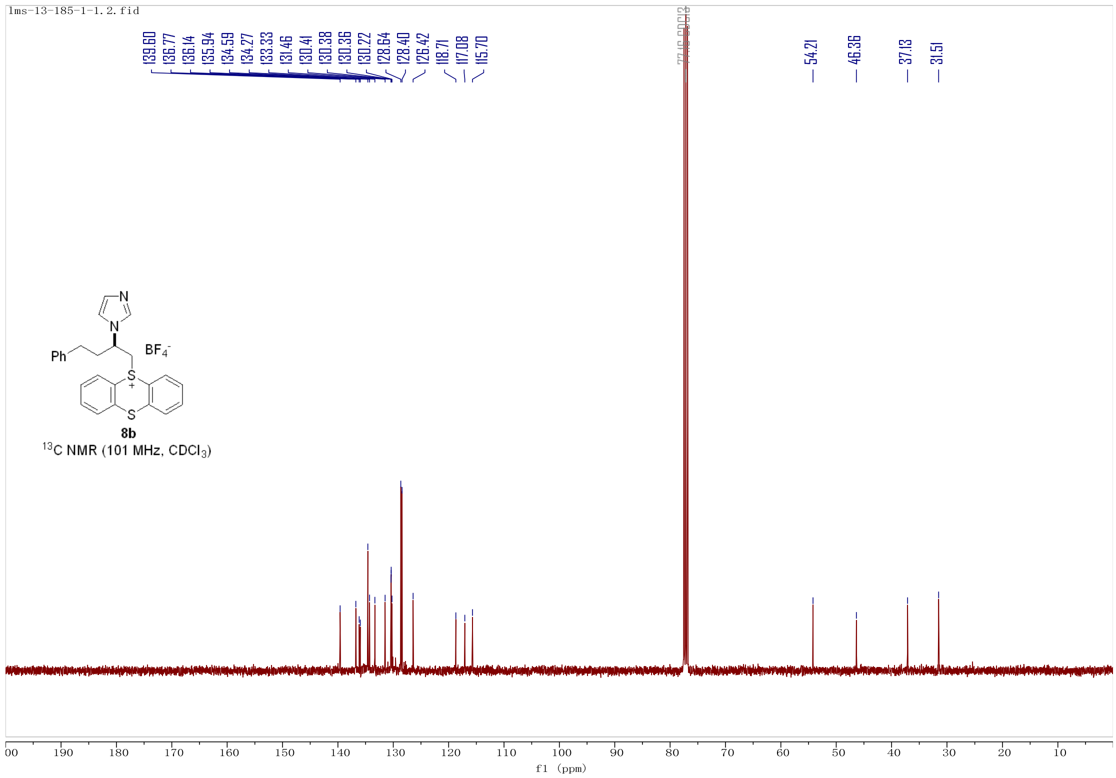
lms-13-185-1-1.2.fid
139.60
136.77
136.14
135.94
134.59
134.27
133.33
131.46
130.41
130.38
130.36
130.22
128.64
128.40
126.42
118.71
117.08
115.70
54.21
46.36
37.13
31.51
Ph
BF4-
8b
13C NMR (101 MHz, CDCl3)
f1 (ppm)

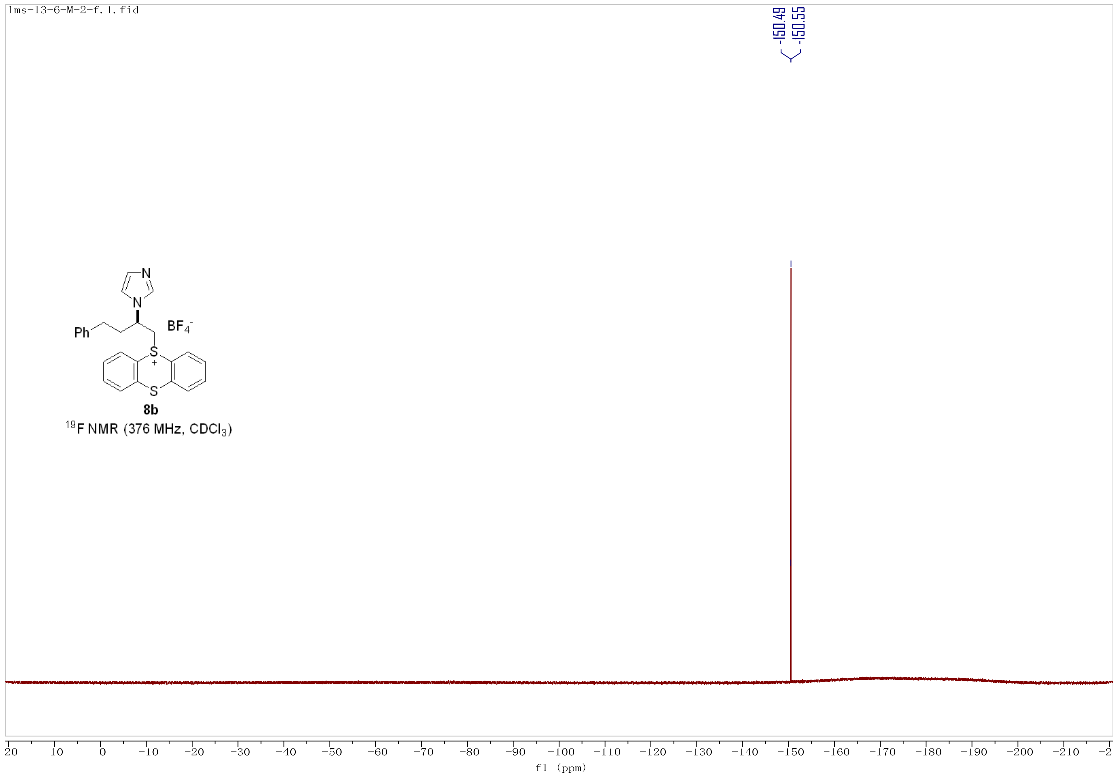
lms-13-6-M-2-f.1.fid
-150.49
-150.55
Ph
BF4-
8b
19F NMR (376 MHz, CDCl3)
f1 (ppm)

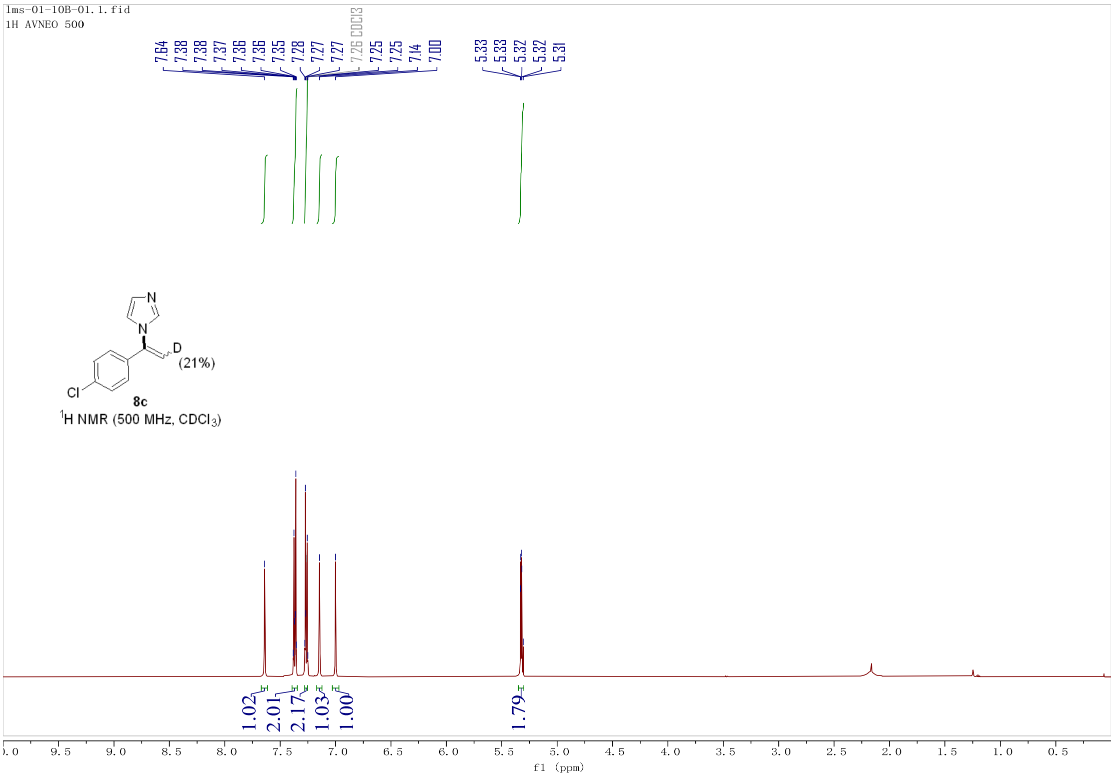

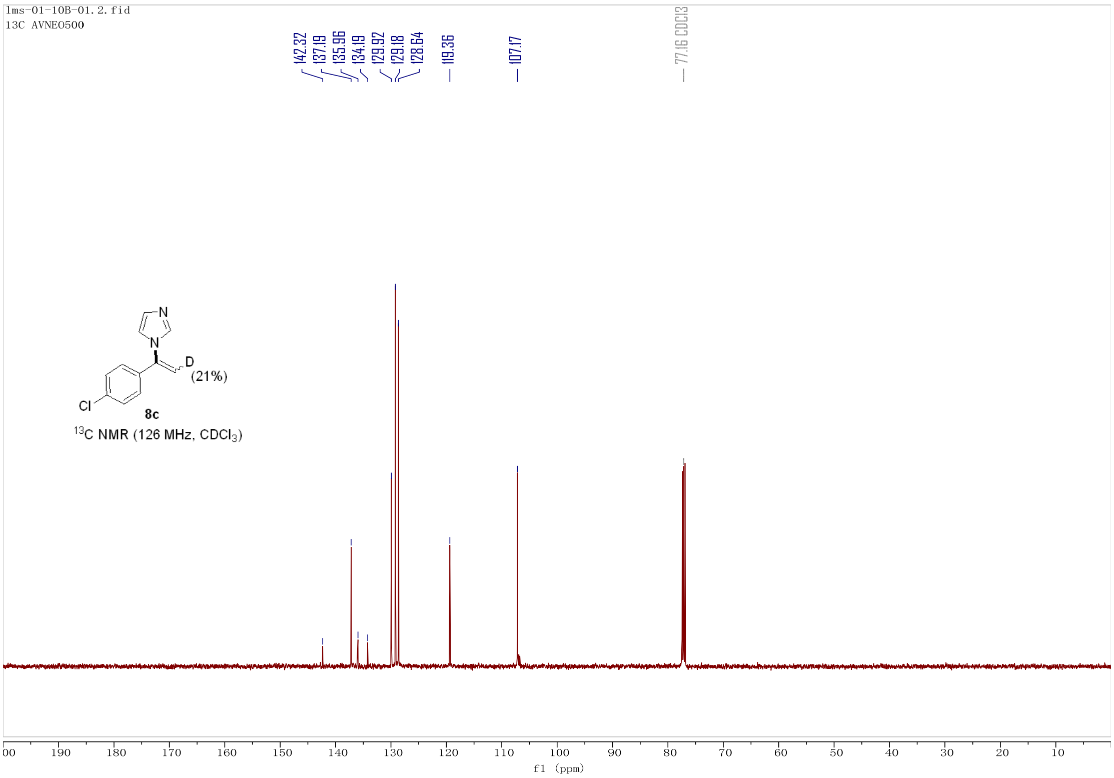

4. Supplementary References

1. Meyer, A. U.; Jäger, S.; Prasad Hari, D. & König, B. Visible Light-Mediated

Metal-Free Synthesis of Vinyl Sulfones from Aryl Sulfinates. *Adv. Synth. Catal.* **357**, 2050-2054 (2015).

2. Shi, S.-H. *et al*. Electro-Oxidation Induced O-S Cross-Coupling of Quinoxalinones with Sodium Sulfinates for Synthesizing 2-Sulfonyloxylated Quinoxalines. *Chem. Commun.* **58**, 12357-12360 (2022).

3. Gao, Y. *et al.* Development of on-DNA Vinyl Sulfone Synthesis for DNA-Encoded Chemical Libraries. *Org. Chem. Front.* **9**, 4542-4548 (2022).

4. Chen, J.; Li, J.; Plutschack, M. B.; Berger, F. & Ritter, T. Regio- and Stereoselective Thianthrenation of Olefins To Access Versatile Alkenyl Electrophiles. *Angew. Chem. Int. Ed.* **59**, 5616-5620 (2020).

5. Liu, M.-S.; Du, H.-W.; Cui, J.-F. & Shu, W. Intermolecular Metal-Free Cyclopropanation and Aziridination of Alkenes with XH_2 (X=N, C) by Thianthrenation. *Angew. Chem. Int. Ed.* **61**, e202209929 (2022).

6. Liu, M.-S.; Du, H.-W. & Shu, W. Metal-Free Allylic C-H Nitrogenation, Oxygenation, and Carbonation of Alkenes by Thianthrenation. *Chem. Sci.* **13**, 1003-1008 (2022).

7. Juliá, F.; Yan, J.; Paulus, F. & Ritter, T. Vinyl Thianthrenium Tetrafluoroborate: A Practical and Versatile Vinylating Reagent Made from Ethylene. *J. Am. Chem. Soc.* **143**, 12992-12998 (2021).

8. Xie, R.; Zhu, J. & Huang, Y. Cu-Catalyzed Highly Selective Silylation and Borylation of Alkenylsulfonium Salts. *Org. Chem. Front.* **8**, 5699-5704 (2021).
